# Alkyl sulfonyl fluorides as ambiphiles in the stereoselective palladium(II)-catalysed cyclopropanation of unactivated alkenes

**DOI:** 10.1038/s44160-025-00925-1

**Published:** 2025-12-16

**Authors:** Yilin Cao, Warabhorn Rodphon, Turki M. Alturaifi, Al Vicente Riano D. Lisboa, Zhouyang Ren, Job J. C. Struijs, Hui-Qi Ni, Taras Savchuk, Richard P. Loach, Shouliang Yang, Indrawan J. McAlpine, Donna G. Blackmond, Pavel K. Mykhailiuk, Peng Liu, K. Barry Sharpless, Keary M. Engle

**Affiliations:** 1 Department of Chemistry, The Scripps Research Institute, La Jolla, CA USA; 2 Department of Chemistry, University of Pittsburgh, Pittsburgh, PA USA; 3 Enamine Ltd., Kyiv, Ukraine; 4 Kukhar Institute of Bioorganic Chemistry and Petrochemistry NAS of Ukraine, Kyiv, Ukraine; 5 Pfizer Medicinal Sciences, Groton, CT USA; 6 Pfizer Oncology Medicinal Chemistry, San Diego, CA USA; 7 Genesis Therapeutics, San Diego, CA USA; 8 Faculty of Chemistry, Taras Shevchenko National University of Kyiv, Kyiv, Ukraine

**Keywords:** Homogeneous catalysis, Synthetic chemistry methodology

## Abstract

Here we present the ambiphilic reactivity of alkyl sulfonyl fluorides in the stereoselective synthesis of diverse cyclopropanes from olefins, under palladium(II) catalysis. The sulfonyl fluoride functionality serves as both an acidifying group and an internal oxidant within the ambiphile, enabling successive carbopalladation and oxidative addition steps in the catalytic cycle, respectively. The transformation grants access to *cis*-substituted cyclopropanes and exhibits broad compatibility with various alkyl sulfonyl fluorides, including those bearing –CN, –CO_2_R, isoxazolyl, pyrazolyl and aryl groups. With internal alkene substrates, 1,2,3-trisubstituted cyclopropanes that are otherwise challenging to synthesize are formed in good-to-moderate yields and predictable diastereoselectivity. Detailed mechanistic insights from reaction progress kinetic analysis and density functional theory calculations reveal that the S_N_2-type C–SO_2_F oxidative addition is the turnover-limiting and diastereoselectivity-determining step.

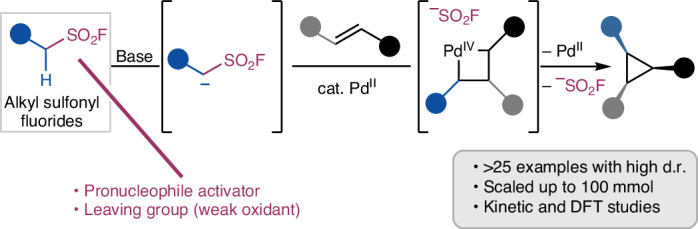

## Abstract (web summary)

The ambiphilic reactivity of alkyl sulfonyl fluorides in stereoselective cyclopropanation under Pd(II) catalysis is reported. The method provides convenient access to *cis*-disubstituted cyclopropanes that are otherwise challenging to access. A representative carboxylic acid cyclopropane building block is scaled up to 100 mmol, demonstrating the practicality of this transformation.

## Main

**Fig. 1:**
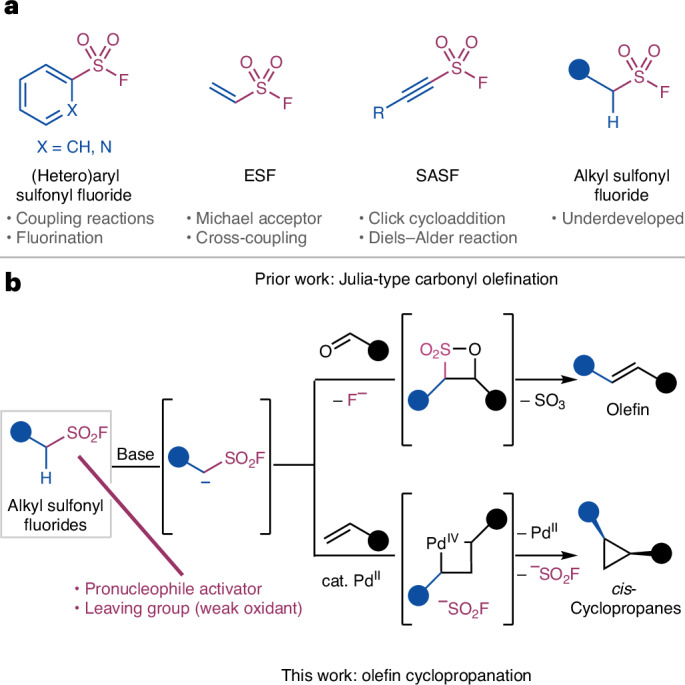
Background and this work. **a**, Modern synthetic usages of sulfonyl fluoride reagents. **b**, C–C bond formation with alkyl sulfonyl fluorides. ESF, ethenesulfonyl fluoride; SASF, 2-substituted-alkynyl-1-sulfonyl fluoride.

Organosulfonyl fluorides have emerged as central players in organic synthesis^1–3^, owing to the rise of sulfur(VI) fluoride exchange (SuFEX) chemistry, which enables rapid and reliable diversification through nucleophilic displacement of fluoride with oxygen and nitrogen nucleophiles^4–6^. The utility of organosulfonyl fluorides in this context has motivated the development of a growing number of methods to synthesize structurally diverse sulfonyl fluorides in an efficient, selective and modular manner^7–11^ (Fig. 1a).

In contrast to widely studied aryl-^12–17^, alkenyl-^18–21^ and alkynyl sulfonyl fluorides^11^, alkyl sulfonyl fluorides have been less thoroughly investigated despite offering unique opportunities as synthetic building blocks owing to the acidifying effect of the sulfonyl group on the α-protons^5^. In recent work, Bull developed an elegant series of desulfonative couplings of azetidine and oxetane sulfonyl fluorides for C–heteroatom bond formation^22,23^. Prior work has demonstrated the utility of alkyl sulfonyl fluorides in C–C bond formation through reaction with classical electrophiles, such as alkyl halides and carbonyl compounds, in the presence of base^16,17,24–30^ (Fig. 1b). We surmised that alkyl sulfonyl fluorides could exhibit unique ambiphilic reactivity in transition metal catalysis, capitalizing on the ability of the sulfonyl fluoride group to serve the dual role as a pronucleophile activator and electrophilic reactive group (internal oxidant).

Our laboratory previously developed a palladium-catalysed *anti*-cyclopropanation of olefins with various carbon pronucleophiles and I_2_ as the bystanding oxidant, where the hypothesized mechanism involves initial α-iodination of the nucleophile, directed carbopalladation, Pd^II^/Pd^IV^ oxidative addition of the newly formed C(*sp*^3^)–I bond and, finally, C(*sp*^3^)–C(*sp*^3^) reductive elimination from the palladacyclobutane intermediate^31^. The need for doubly activated carbon pronucleophiles in all cases but nitromethane limits the scope of substituted cyclopropanes that can be accessed with this method, leading us to search for alternative nucleophilic coupling partners in which a leaving group and an acidifying group could be merged into the same entity.

Here, we demonstrate alkyl sulfonyl fluorides acting as ambiphilic coupling partners in the stereoselective cyclopropanation of unactivated olefins through the Pd^II^/Pd^IV^ redox couple. The catalytic cycle leverages a novel mode of reactivity, C(*sp*^3^)–SO_2_F bonds oxidatively adding to organopalladium(II) intermediates, to deliver stereochemical outcomes that are inaccessible using conventional leaving groups^32–35^ (Fig. 1b). While this paper was in revision, Mane and Baidya reported a directed cyclopropanation reaction involving ketone-derived sulfoxonium ylides to access related *cis*-1,2-disubstituted cyclopropane products^36^.

## Results and discussion

### Reaction development

In this context, alkenyl amide **1a**, featuring the 8-aminoquinoline directing auxiliary required for reactivity^31^, and alkyl sulfonyl fluoride **2a** were selected as model substrates. After extensive screening, we identified optimal reaction conditions using a combination of Pd(OAc)_2_ (10 mol%) and Na_2_CO_3_ (1.0 equiv.) in dimethylacetamide (DMA) (0.33 M) under a nitrogen atmosphere to afford cyclopropane **3aa** in 94% yield with 98:2 diastereomeric ratio (d.r.); the unusual *cis*-configuration of the major product was confirmed by single-crystal X-ray diffraction^37–42^ (Table 1). Key findings from our optimization campaign include the important role of Na_2_CO_3_ as the base, as other carbonate salts, such as K_2_CO_3_ and Cs_2_CO_3_, led to substantially lower yields (entries 2 and 3), as did bases with other counteranions (entries 4 and 5). Other commonly used Pd^II^ sources, such as PdCl_2_ and Pd(TFA)_2_ (where TFA is trifluoroacetate), gave slightly lower yields and comparable diastereoselectivity (entries 6 and 7). Increasing the concentration of the reaction mixture decreased the yield (entry 8). The use of a polar aprotic solvent was crucial, and dimethylformamide (DMF) and dimethyl sulfoxide (DMSO) were reasonably effective alternatives to DMA (entries 9–13). We were also pleased to find that the reaction gave 92% yield even when set up and run under air with anhydrous DMA (entry 14). In control experiments, we found that changing the leaving group to sulfonyl chloride (**LG2**), phenyl sulfonyl (**LG3**) or methyl sulfonyl (**LG4**) groups led to no reaction or trace product formation (entry 15), indicating that the sulfonyl fluoride is key for the observed reaction. A representative sulfonium bromide sulfur ylide precursor (**LG5**) gave moderate yields but poor d.r. Beyond sulfur-based leaving groups, halides were also tested but found to be ineffective. An additional control experiment revealed that **2a** furnished a different product, a tri-substituted cyclopropane with the –SO_2_F group intact, when subjected to previously reported conditions for Pd^II^-catalysed cyclopropanation^31^ (see ‘Pd(0) salt screening and control experiments’ section in Supplementary Information).

**Table 1:** Reaction optimization


Entry	Deviation from standard conditions	Yield of 3aa (%)^a^	d.r.^c^
1	None	99 (94)^b^	98:2
2	K_2_CO_3_ instead of Na_2_CO_3_	34	94:6
3	Cs_2_CO_3_ instead of Na_2_CO_3_	3	–
4	NaF instead of Na_2_CO_3_	74	96:4
5	KF instead of Na_2_CO_3_	60	97:3
6	PdCl_2_ instead of Pd(OAc)_2_	95	95:5
7	Pd(TFA)_2_ instead of Pd(OAc)_2_	95	98:2
8	1.0 M DMA instead of 0.33 M	75	96:4
9	DMF instead of DMA	76	95:5
10	DMSO instead of DMA	36	97:3
11	1,2-DCE instead of DMA	n.d.	–
12	Toluene instead of DMA	n.d.	–
13	THF instead of DMA	n.d.	–
14	Air instead of N_2_	92	96:4
15	**LG2**–**LG8** instead of **LG1**	See below	


^a^Reactions performed on 0.1 mmol scale. Yields were determined by ^1^H NMR spectroscopic analysis of the crude reaction mixture with benzyl 4-fluorobenzoate as an internal standard. ^b^Isolated yield shown in parentheses. ^c^The d.r. was determined by ^1^H NMR spectroscopic analysis of the crude reaction mixture. DCE, dichloroethane; THF, tetrahydrofuran; n.d., not detected.

In seeking to rationalize the optimization data, we considered previous reports that found alkyl sulfonyl fluorides, such as phenylmethyl sulfonyl fluoride (PMSF), to be highly unstable in aqueous solutions^43^. We questioned whether yield differences could be partially attributed to the competitive decomposition of the nucleophile under different conditions (in different solvents and in the presence or absence of adventitious water). To this end, we examined the stability of alkyl sulfonyl fluoride **2a** in DMA, DMF and DMSO (see Table 2 and ‘Stability tests’ section in Supplementary Information for details). Surprisingly, alkyl sulfonyl fluoride **2a** was fully decomposed in anhydrous DMSO after 2 h but proved to be relatively stable in anhydrous DMF. Similarly, we found no decomposition of alkyl sulfonyl fluoride **2a** in anhydrous DMA under an inert atmosphere. However, under air, moderate levels of decomposition in DMA could be detected, and complete decomposition was observed in wet DMA. Under aerobic conditions, oxidation and subsequent decomposition of DMA into *N*-methylacetamide and formaldehyde has been previously documented and could be a contributing factor to these observations^44^. These results indicate that the rate of decomposition of **2a** is dependent on the solvent identity and amount of adventitious water in the solution.

**Table 2:** Nucleophile **2a** stability test


Solvent	H_2_O content	Atmosphere	Remaining 2a^a^
DMA	Anhydrous + 10% water	Air	<5%
DMA	Anhydrous	Air	83%
DMA	Anhydrous	N_2_	100%
DMF-d_7_	Anhydrous	N_2_	85%
DMSO-d_6_	Anhydrous	N_2_	<5%

^a^Reactions performed on 0.1 mmol scale. Percentages represent ^1^H NMR yields using CH_2_Br_2_ as the internal standard.

### Substrate scope

**Fig. 2:**
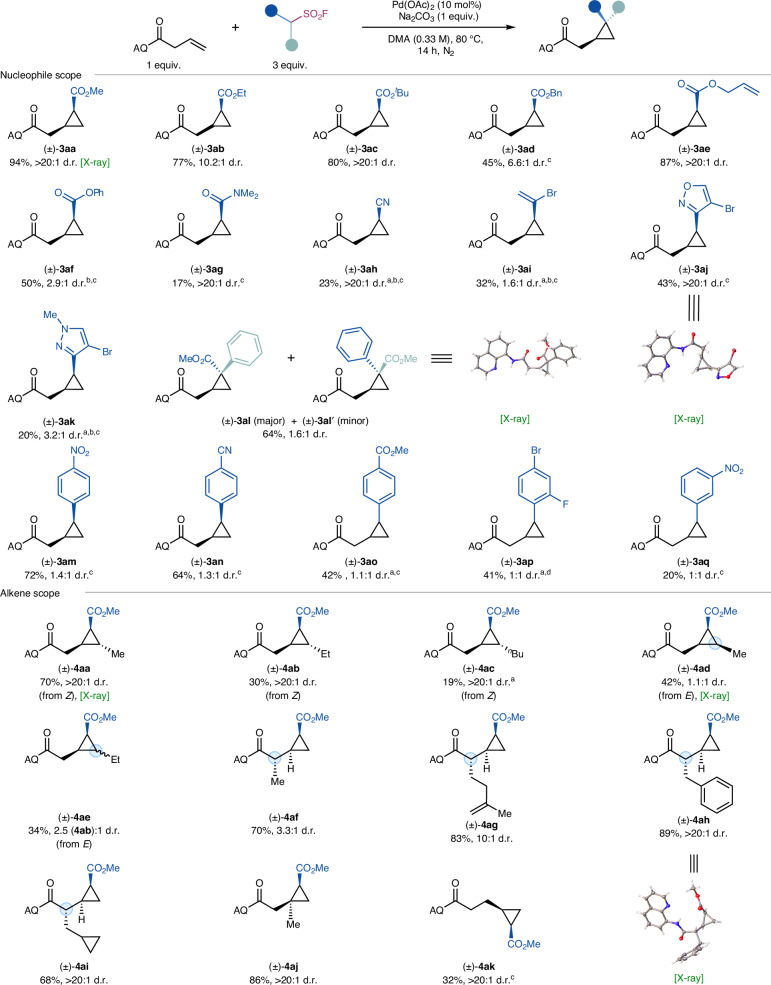
Substrate scope. Reactions performed on 0.1 mmol scale. Percentages represent isolated yields. The d.r. was determined by ^1^H NMR spectroscopy of crude or purified reaction mixture. The diastereomeric centre is highlighted with a blue circle. ^a^Reactions performed under 20 mol% catalyst loading. ^b^Reactions performed under 5 equiv. alkyl fluoride loading. ^c^Reactions performed under 24 h. ^d^Reactions performed under 36 h.

Having optimized the conditions, we began investigating the scope with respect to the alkyl sulfonyl fluoride, many of which are commercially available or readily accessible using routine procedures (see ‘General procedures for starting material preparation’ section in Supplementary Information) (Fig. 2). The reaction proceeded in moderate-to-excellent yields with various ester nucleophiles, including ethyl ester (**3ab**), *tert*-butyl ester (**3ac**), benzyl ester (**3ad**), allyl ester (**3ae**) and phenyl ester (**3af**), providing the corresponding *cis*-cyclopropanes as the major products with high diastereoselectivity and without appreciable base-mediated epimerization^45^. Amide or nitrile-based coupling partners (**3ag**–**3ah**) led to diminished yield but maintained excellent *cis*-diastereoselectivity (>20:1). We found that the reaction could tolerate weaker electron-withdrawing groups, including a 2-bromopropenyl group (**3ai**) and various heterocycles, including isoxazole (**3aj**) and 1-methyl-1*H*-pyrazole (**3ak**), which gave 32%, 43% and 20% yield, respectively. An α-aryl alkyl sulfonyl fluoride nucleophile participated in the reaction to form a fully substituted carbon centre within the cyclopropane, albeit with moderate yield and d.r. (**3al**). Aryl-substituted methylene sulfonyl fluorides were also investigated. Interestingly, the reaction did not proceed with unsubstituted, electron-neutral benzylsulfonyl fluoride, but proceeded with substituted benzylsulfonyl fluoride substrates bearing electron-withdrawing groups at the *para*-position (**3am**–**3ap**). Stronger electron-withdrawing groups led to improved yields and diastereoselectivity. A *meta*-nitro substrate was also tolerated (**3aq**).

Next, the scope of alkenes was investigated. Internal alkenes afforded good-to-moderate yields (**4aa**–**4ae**). Notably, (*Z*)-alkenes reacted with excellent diastereoselectivity (>20:1), affording a single diastereomer. To our delight, under the standard condition, the thermodynamically and kinetically unfavourable all-*cis* cyclopropane could be synthesized from an (*E*)-configured internal alkene in one step in moderate yield (**4ad**) with 1.1:1 d.r. In addition, α-substituted β,γ-unsaturated amide substrates are well tolerated, giving the desired products in moderate-to-excellent yields (**4af**–**4ai**). Specifically, a diene substrate (**4ag**) could be chemoselectively cyclopropanated at the β,γ-alkene, while leaving the δ,ε-alkene unperturbed. When the tether length between the alkene and directing group was extended and a γ,δ-unsaturated amide was tested, the cyclopropane product was furnished in moderate yield with excellent diastereoselectivity (**4ak**).

**Fig. 3:**
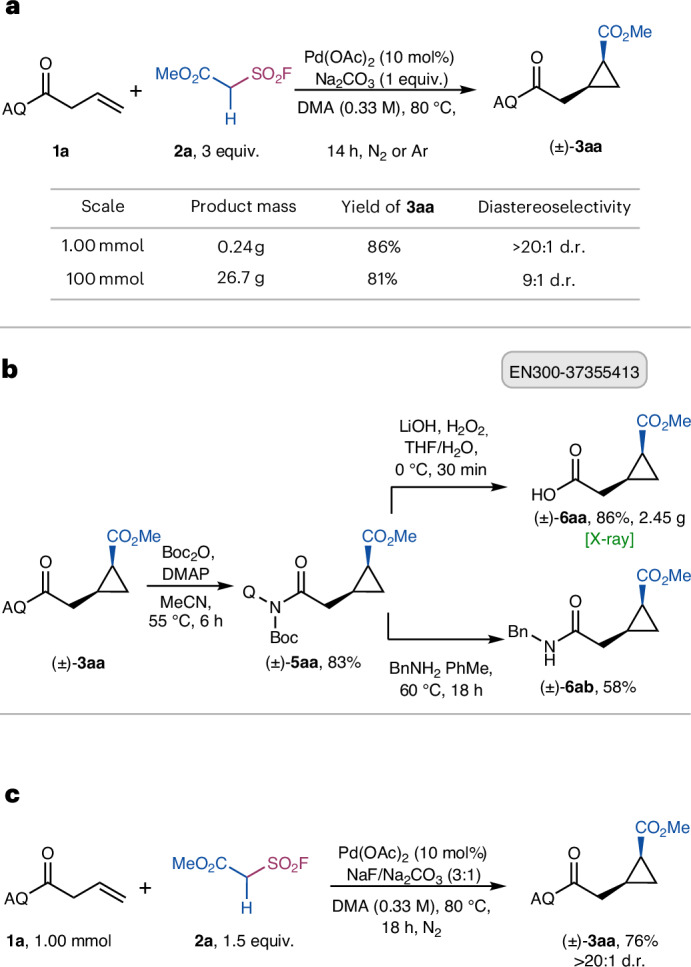
Scale-up, directing auxiliary removal and lower nucleophile loading. **a**, Large-scale synthesis. **b**, Directing auxiliary removal. **c**, Lower nucleophile loading. DMAP, 4-(dimethylamino)pyridine; Boc, *tert*-butyloxycarbonyl; Bn, benzyl.

The methodology could be easily scaled up and performed on 1.0-mmol scale under the standard reaction conditions, giving *cis*-cyclopropane **3aa** in 86% yield (Fig. 3a). Performing the reaction on 100 mmol scale resulted in 81% yield with 9:1 d.r. Removal of the directing auxiliary was possible through a two-step sequence^46^. Initial introduction of an *N*-Boc activating group proceeded in 83% yield (**5aa**). Then, hydrolysis with LiOOH/water gave carboxylic acid **6aa** in 86% yield on multigram-scale, which has been commercialized by Enamine (Fig. 3b). Alternatively, a representative amine could be used in the second step in a net transamidation (58% yield, **6ab**). If desired by the practitioner, the loading of nucleophile **2a** can be lowered to 1.5 equiv. while maintaining comparable yield and diastereoselectivity with a dual base system (0.75 equiv. NaF and 0.25 equiv. Na_2_CO_3_) (Fig. 3c; see ‘Base screening at lower nucleophile loading’ section in Supplementary Information for details).

### Kinetic studies

**Fig. 4:**
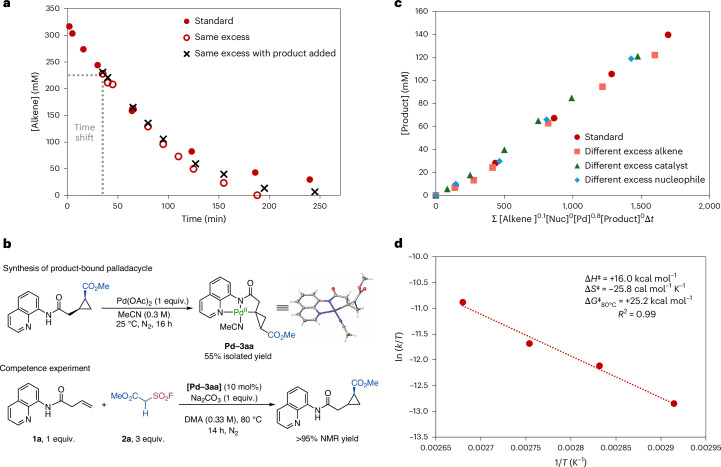
Mechanistic experiments. **a**, Reaction progress kinetic analysis: same excess. **b**, Product binding experiments. **c**, Variable time normalization analysis. [Nuc] = [SO^2^F]. **d**, Eyring analysis. Source data

The distinctive reactivity of alkyl sulfonyl fluorides in this cyclopropanation, along with the observed stereochemical outcomes, prompted us to examine the mechanism through experimental and computational techniques. We performed reaction progress kinetic analysis to determine key mechanistic features of the reaction from a minimal number of experiments^47^. First, we compared the standard reaction profile with a ‘same excess’ experiment that simulated 25% conversion (Fig. 4a). Time-shifting reveals that the two curves overlap as the reaction proceeds to approximately 50% conversion; beyond this point, a slight deviation emerges—an indication of either mild product inhibition or partial catalyst deactivation. To distinguish between the two possibilities, a third experiment was performed with product **3aa** (83 mM) as an additive, and overlay with the same excess experiment confirmed mild catalyst deactivation. The lack of product inhibition is notable given the capacity of the product to bind to the catalyst in a polydentate fashion. To explore potential coordination between the product and catalyst further, **3aa** was treated with Pd(OAc)_2_ (1 equiv.), resulting in the formation of *N,N,C-*palladacycle **Pd–3aa** in which the 3 °C (cyclopropyl)–H bond was cleaved (Fig. 4b) as characterized by X-ray crystallography^48^. Complex **Pd–3aa** proved to be catalytically competent under the standard reaction conditions, showing the reversibility of C–H activation, consistent with the lack of product inhibition. Next, to estimate the order of the reaction components, we performed a series of ‘different excess’ experiments (Fig. 4c). Considering that catalyst deactivation becomes more pronounced at higher conversion, we focused on the early stages of the reaction (<50% conversion). The variable time normalization analysis^49^ plot showed both alkene (**1a**) and nucleophile (**2a**) to be close to zero-order and Pd(OAc)_2_ to be close to first-order. Overall, the kinetic data are consistent with either oxidative addition or reductive elimination as the rate-determining step. To gain further insights into the rate-determining step, we conducted an Eyring analysis to obtain activation parameters (Fig. 4d). At temperatures close to standard conditions (70–100 °C), the experimental Δ*S*^‡^ was determined to be −25.8 e.u. and Δ*H*^‡^ to be 16.1 kcal mol^−1^ (Δ*G*^‡^_80°C_ = 25.2 kcal mol^−1^). Given that large negative Δ*S*^‡^ values are commonly observed with bimolecular associative reactions^50^, and oxidative addition and reductive elimination of the current system are unimolecular steps, the data suggest potential desolvation and coordination of a ligand to the metal centre in traversing from the catalyst resting state to the rate-limiting transition state along the potential energy surface, which informed subsequent density functional theory (DFT) calculations.

### Computational studies

**Fig. 5:**
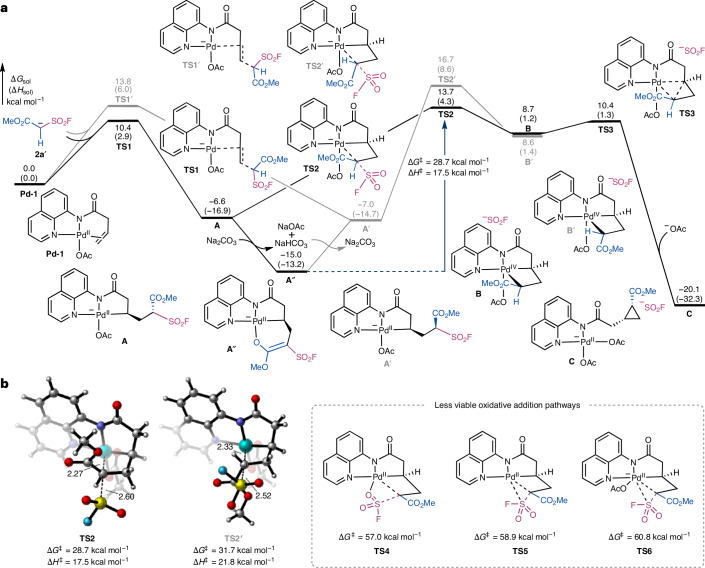
Proposed reaction pathways. **a**, DFT-computed reaction energy profile of the formation of cyclopropane **3aa**. The DFT calculations were performed at the ωB97X-D/def2-TZVP/SMD(DMA)//B3LYP-D3(BJ)/6-31G(d)–SDD(Pd) level of theory. The reaction energy profile was calculated at the experimental reaction temperature (80 °C). See the ‘Density functional theory (DFT) calculations’ section in Supplementary Information for computational details. All Gibbs free energies and enthalpies are with respect to **Pd-1**. **b**, Oxidative addition transition states. All activation barriers are with respect to the enolate resting state **Aʹʹ**.

DFT calculations with alkene **1a** and alkyl sulfonyl fluoride nucleophile **2a** as model substrates were performed to gain insights into various facets of the catalytic cycle, including the rate- and diastereoselectivity-determining steps (Fig. 5a). A particular focus was placed on the oxidative addition mechanisms and the origin of diastereoselectivity favouring the *cis*-cyclopropane product **3aa**. Ligand exchange of the precatalyst Pd(OAc)_2_ with **1a** forms π-alkene Pd^II^ complex **Pd-1** with the 8-aminoquinoline directing group binding to the Pd in a bidentate fashion. Two competing *anti*-nucleopalladation transition states with the deprotonated nucleophile **2aʹ** were considered (**TS1** and **TS1ʹ**), which lead to two different diastereomers of the palladacycle intermediate (**A** and **Aʹ**, respectively). The deprotonation of **2a** to form **2aʹ** is expected to be facile based on its predicted aqueous p*K*_a_ value of 7.75 for **2a** and the experimental p*K*_a_ of 6.83 for the analogous trifluoromethanesulfonyl^51–53^ (see Supplementary Fig. 10 for details). In the more stable transition state **TS1**, the largest substituent on the nucleophile, SO_2_F, is placed *anti*-periplanar with the alkene C=C bond, which could minimize steric strain, whereas in the less stable diastereomeric transition state **TS1ʹ**, the ester group is *anti*-periplanar with the C=C bond (Supplementary Fig. 4). This steric effect probably leads to the 3.4 kcal mol^−1^ higher energy of **TS1ʹ** than that of **TS1**. Although the formation of palladacycle **A** is kinetically favoured in the *anti*-nucleopalladation, diastereomers **A** and **Aʹ** rapidly epimerize via deprotonation of the acidic α-C–H. The Na_2_CO_3_-mediated deprotonation of **A** to form enolate **Aʹʹ** is predicted to be exergonic by 8.4 kcal mol^−1^ (see Supplementary Fig. 5 for details). This deprotonation process is in part entropy-driven as the enolate oxygen replaces the acetate ligand in **A** to form a six-membered chelation with the Pd centre.

Due to the ablation of the new α-stereocentre formed after nucleopalladation, under the Curtin–Hammett conditions, the product diastereoselectivity is determined in the subsequent steps. Our calculations indicate that the oxidative addition proceeds via an S_N_2-type stereoinvertive mechanism (**TS2** and **TS2ʹ**). Other oxidative addition pathways, including the four- and three-centred C–S oxidative addition (**TS4**–**TS6**; Fig. 5b) and the S–F oxidative addition^16,17^ (Supplementary Fig. 6), were found to be less favourable. Subsequent reductive elimination from the Pd^IV^ intermediate **B** (**TS3**) is kinetically facile with a low barrier of 1.7 kcal mol^−1^ with respect to **B**, consistent with our recent computational study on the strain-release-promoted reductive elimination from Pd^IV^ to form cyclopropane rings^31^. The liberated SO_2_F^−^ anion then decomposes to SO_2_ gas and F^−^ anion, similar to previous reports by Bull and coworkers^22,23^. In situ ^19^F NMR spectroscopy monitoring indicates that F^−^ is ultimately converted to bifluoride (FHF^−^) (see ‘Fluorine tracking experiments’ section in Supplementary Information for details). The computed reaction energy profile indicates that the irreversible intramolecular S_N_2-type oxidative addition (**TS2**) is the rate- and diastereoselectivity-determining step. The computed overall barrier from the Pd–enolate resting state **Aʹʹ** to **TS2** (Δ*G*^‡^ = 28.7 kcal mol^−1^, Δ*H*^‡^ = 17.5 kcal mol^−1^) is in qualitative agreement with the experimentally determined activation parameters. The large negative activation entropy (Δ*S*^‡^ = − 31.7 e.u. compared with the experimental value of −25.8 e.u.) is primarily attributed to entropy loss during the association of an OAc^−^ ligand upon reprotonation of the enolate resting state **Aʹʹ**. The computed diastereoselectivity indicates that the S_N_2-type oxidative addition via **TS2** leading to the *cis*-cyclopropane product is 3.0 kcal mol^−1^ more favourable than the minor pathway via **TS2ʹ** leading to the *trans* product, which is consistent with the experimentally observed diastereoselectivity (>20:1 d.r.). **TS2ʹ** is probably destabilized by steric interactions between the acetate ligand and the ester group. The S_N_2-type oxidative addition with a phenyl ester (**2f**) had an increased barrier (Δ*G*^‡^ = 30.9 kcal mol^−1^) and lower computed diastereoselectivity (ΔΔ*G*^‡^ = 1.2 kcal mol^−1^), attributed to steric repulsions between the directing group and the ester that stabilize the transition state leading to the *cis*-cyclopropane product (Supplementary Fig. 9).

## Conclusion

A highly diastereoselective Pd^II^/Pd^IV^-catalysed cyclopropanation of unactivated alkenes with alkyl sulfonyl fluorides has been developed. The synthetic versatility of this method stems from the diverse array of pronucleophiles that are compatible. With internal alkenes, all *cis*-substituted cyclopropanes that are otherwise difficult to access can be prepared in one step. Kinetic experiments and DFT calculations indicate that the S_N_2-type oxidative addition is the rate- and diastereoselectivity-determining step.

## Methods

Outside of the glovebox, the appropriate alkene substrate (0.1 mmol), Pd(OAc)_2_ (0.01 mmol, 10mol%) and Na_2_CO_3_ (0.1 mmol, 1.0 equiv.) were added to an oven-dried 1-dram (4 ml) vial equipped with a magnetic stir bar. The vial was then introduced into a nitrogen-filled glovebox antechamber. Once transferred inside the glovebox, alkyl sulfonyl fluoride (0.3 mmol, 3.0 equiv.) was added to the vial, followed by anhydrous DMA (0.3 ml, 0.33 M). The vial was sealed with a screw-top septum cap, removed from the glovebox and placed in a heating block that was preheated to 80 °C for 14 h. After this time period, the resulting mixture was filtered through a pad of celite. Saturated NaHCO_3_ solution (10 ml) was added to the filtrate, and the aqueous layer was extracted with ethyl acetate (3 × 2 ml). The combined organic layers were concentrated and purified by silica gel flash column chromatography using ethyl acetate/hexanes as the eluent.

## Supplementary information

Supplementary Information Experimental details, Supplementary Figs. 1–12 and Tables 1–36.

Supplementary Data 1 Crystallographic data for compound (±)-**3aa**, CCDC 2171740.

Supplementary Data 2 Crystallographic data for compound (±)-**3aj**, CCDC 2345363.

Supplementary Data 3 Crystallographic data for compound (±)-**3al′**, CCDC 2351469.

Supplementary Data 4 Crystallographic data for compound (±)-**4aa**, CCDC 2345089.

Supplementary Data 5 Crystallographic data for compound (±)-**4ad**, CCDC 2376390.

Supplementary Data 6 Crystallographic data for compound (±)-**4ah**, CCDC 2367118.

Supplementary Data 7 Crystallographic data for compound (±)-**6aa**, CCDC 2394329.

Supplementary Data 8 Crystallographic data for compound **Pd–3aa**, CCDC 2171739.

## Source data

Source Data Fig. 4 Source data for Fig. 4.

## Data Availability

The data that support the findings of this study are available within the article and its Supplementary Information files. Experimental procedures, characterization data for all new compounds and details for DFT calculations can be found in Supplementary Information. Crystallographic data for the structure reported in this article have been deposited at the Cambridge Crystallographic Data Centre, under deposition numbers CCDC 2171740 ((±)-**3aa**), CCDC 2345363 ((±)-**3aj**), CCDC 2351469 ((±)-**3al****′**), CCDC 2345089 ((±)-**4aa**), CCDC 2376390 ((±)-**4ad**), CCDC 2367118 ((±)-**4ah**), CCDC 2394329 ((±)-**6aa**) and CCDC 2171739 (**Pd–3aa**); copies of the data can be obtained free of charge at https://www.ccdc.cam.ac.uk/structures/.

## References

[1] Chinthakindi, P. K. & Arvidsson, P. I. Sulfonyl fluorides (SFs): more than click reagents? *Eur. J. Org. Chem.* **2018**, 3648–3666 (2018).

[2] Zhao, C., Rakesh, K. P., Ravidar, L., Fang, W.-Y. & Qin, H.-L. Pharmaceutical and medicinal significance of sulfur (S^VI^)-containing motifs for drug discovery: a critical review. *Eur. J. Med. Chem.* **162**, 679–734 (2019). 30496988 10.1016/j.ejmech.2018.11.017 PMC7111228

[3] Lou, T. S.-B. & Willis, M. C. Sulfonyl fluorides as targets and substrates in the development of new synthetic methods. *Nat. Rev. Chem.* **6**, 146–162 (2022). 37117299 10.1038/s41570-021-00352-8

[4] Narayanan, A. & Jones, L. H. Sulfonyl fluorides as privileged warheads in chemical biology. *Chem. Sci.* **6**, 2650–2659 (2015). 28706662 10.1039/c5sc00408j PMC5489032

[5] Dong, J., Krasnova, L., Finn, M. G. & Sharpless, K. B. Sulfur(VI) fluoride exchange (SuFEx): another good reaction for click chemistry. *Angew. Chem. Int. Ed.* **53**, 9430–9448 (2014). 10.1002/anie.201309399 25112519

[6] Barrow, A. S. et al. The growing applications of SuFEx click chemistry. *Chem. Soc. Rev.* **48**, 4731–4758 (2019). 31364998 10.1039/c8cs00960k

[7] Tribby, A. L., Rodríguez, I., Shariffudin, S. & Ball, N. D. Pd-catalyzed conversion of aryl iodides to sulfonyl fluorides using SO_2_ surrogate DABSO and Selectfluor. *J. Org. Chem.* **82**, 2294–2299 (2017). 28134537 10.1021/acs.joc.7b00051

[8] Kwon, J. & Kim, B. M. Synthesis of arenesulfonyl fluorides via sulfuryl fluoride incorporation from arynes. *Org. Lett.* **21**, 428–433 (2019). 30592614 10.1021/acs.orglett.8b03610

[9] Laudadio, G. et al. Sulfonyl fluoride synthesis through electrochemical oxidative coupling of thiols and potassium fluoride. *J. Am. Chem. Soc.* **141**, 11832–11836 (2019). 31303004 10.1021/jacs.9b06126 PMC6676414

[10] Lee, C., Ball, N. D. & Sammis, G. M. One-pot fluorosulfurylation of Grignard reagents using sulfuryl fluoride. *Chem. Commun.* **55**, 14753–14756 (2019). 10.1039/c9cc08487h 31754668

[11] Smedley, C. J. et al. Diversity oriented clicking (DOC): divergent synthesis of SuFExable pharmacophores from 2-substituted-alkynyl-1-sulfonyl fluoride (SASF) hubs. *Angew. Chem. Int. Ed.* **59**, 12460–12469 (2020). 10.1002/anie.202003219 PMC7572632 32301265

[12] Erchinger, J. E. et al. EnT-mediated N–S bond homolysis of a bifunctional reagent leading to aliphatic sulfonyl fluorides. *J. Am. Chem. Soc.* **145**, 2364–2374 (2023). 36652725 10.1021/jacs.2c11295

[13] Chinthakindi, P. K., Kruger, H. G., Govender, T., Naicker, T. & Arvidsson, P. I. On-water synthesis of biaryl sulfonyl fluorides. *J. Org. Chem.* **81**, 2618–2623 (2016). 26900892 10.1021/acs.joc.5b02770

[14] Cherepakha, A. Y. et al. Hetaryl bromides bearing the SO_2_F group—versatile substrates for palladium-catalyzed C–C coupling reactions. *Eur. J. Org. Chem.* **2018**, 6682–6692 (2018).

[15] Lou, T. S.-B. & Willis, M. C. Arylsulfonyl fluoride boronic acids: preparation and coupling reactivity. *Tetrahedron* **76**, 130782 (2020).

[16] Chatelain, P. et al. Desulfonative Suzuki–Miyaura coupling of sulfonyl fluorides. *Angew. Chem. Int. Ed.* **60**, 25307–25312 (2021). 10.1002/anie.202111977 34570414

[17] Zhang, G., Guan, C., Zhao, Y., Miao, H. & Ding, C. “Awaken” aryl sulfonyl fluoride: a new partner in the Suzuki–Miyaura coupling reaction. *New J. Chem.* **46**, 3560–3564 (2022).

[18] Nielsen, M. K., Ugaz, C. R., Li, W. & Doyle, A. G. PyFluor: a low-cost, stable, and selective deoxyfluorination reagent. *J. Am. Chem. Soc.* **137**, 9571–9574 (2015). 26177230 10.1021/jacs.5b06307

[19] Chen, Q., Mayer, P. & Mayr, H. Ethenesulfonyl fluoride: the most perfect Michael acceptor ever found? *Angew. Chem. Int. Ed.* **55**, 12664–12667 (2016). 10.1002/anie.201601875 27159425

[20] Qin, H.-L., Zheng, Q., Bare, G. A. L., Wu, P. & Sharpless, K. B. A Heck–Matsuda process for the synthesis of β-arylethenesulfonyl fluorides: selectively addressable bis-electrophiles for SuFEx click chemistry. *Angew. Chem. Int. Ed.* **55**, 14155–14158 (2016). 10.1002/anie.201608807 PMC5102157 27723200

[21] Zha, G.-F. et al. Palladium-catalyzed fluorosulfonylvinylation of organic iodides. *Angew. Chem. Int. Ed.* **56**, 4849–4852 (2017). 10.1002/anie.201701162 PMC5653204 28370917

[22] Rojas, J. J. et al. Amino-oxetanes as amide isosteres by an alternative defluorosulfonylative coupling of sulfonyl fluorides. *Nat. Chem.* **14**, 160–169 (2022). 35087220 10.1038/s41557-021-00856-2

[23] Symes, O. L. et al. Harnessing oxetane and azetidine sulfonyl fluorides for opportunities in drug discovery. *J. Am. Chem. Soc.* **146**, 35377–35389 (2024). 39666854 10.1021/jacs.4c14164 PMC11673132

[24] Hawkins, J. M., Lewis, T. A. & Raw, A. S. Substituent effects on sulfonate ester based olefinations. *Tetrahedron Lett.* **31**, 981–984 (1990).

[25] Kagabu, S., Hara, K. & Takahashi, J. Alkene formation through condensation of phenylmethanesulphonyl fluoride with carbonyl compounds. *J. Chem. Soc. Chem. Commun*. 408–410 (1991).

[26] Kagabu, S. et al. Reaction of phenyl- and alkoxycarbonylmethanesulfonyl fluoride with activated haloalkanes. *Bull. Soc. Chim. Fr.* **129**, 435–439 (1992).

[27] Górski, B., Talko, A., Basak, T. & Barbasiewicz, M. Olefination with sulfonyl halides and esters: scope, limitations, and mechanistic studies of the Hawkins reaction. *Org. Lett.* **19**, 1756–1759 (2017). 28300408 10.1021/acs.orglett.7b00517

[28] Górski, B. et al. Olefination with sulfonyl halides and esters: E-selective synthesis of alkenes from semistabilized carbanion precursors. *Eur. J. Org. Chem.* **2018**, 1774–1784 (2018).

[29] Tryniszewski, M., Basiak, D. & Barbasiewicz, M. Olefination with sulfonyl halides and esters: synthesis of unsaturated sulfonyl fluorides. *Org. Lett.* **24**, 4270–4274 (2022). 35653711 10.1021/acs.orglett.2c01604 PMC9490844

[30] Liang, H. et al. Amidation of β-keto sulfonyl fluorides via C–C bond cleavage. *Eur. J. Org. Chem.* **27**, e202400391 (2024).

[31] Ni, H.-Q. et al. *Anti-selective* cyclopropanation of nonconjugated alkenes with diverse pronucleophiles via directed nucleopalladation. *J. Am. Chem. Soc.* **146**, 24503–24514 (2024). 39172733 10.1021/jacs.4c07039 PMC11815279

[32] Davies, H. M. L. & Antoulinakis, E. Intermolecular metal-catalyzed carbenoid cyclopropanations. *Org. React.* **57**, 1–326 (2001).

[33] Lebel, H., Marcoux, J.-F., Molinaro, C. & Charette, A. B. Stereoselective cyclopropanation reactions. *Chem. Rev.* **103**, 977–1050 (2003). 12683775 10.1021/cr010007e

[34] Maas, G. Ruthenium-catalysed carbenoid cyclopropanation reactions with diazo compounds. *Chem. Soc. Rev.* **33**, 183–190 (2004). 15026823 10.1039/b309046a

[35] Ford, A. et al. Modern organic synthesis with α-diazocarbonyl compounds. *Chem. Rev.* **115**, 9981–10080 (2015). 26284754 10.1021/acs.chemrev.5b00121

[36] Ballav, N., Giri, C. K., Saha, S. N., Mane, M. V. & Baidya, M. Empowering diastereoselective cyclopropanation of unactivated alkenes with sulfur ylides through nucleopalladation. *J. Am. Chem. Soc.* **147**, 13017–13026 (2025). 40194297 10.1021/jacs.5c03146

[37] Lévesque, É., Goudreau, S. R. & Charette, A. B. Improved zinc-catalyzed Simmons–Smith reaction: access to various 1,2,3-trisubstituted cyclopropanes. *Org. Lett.* **16**, 1490–1493 (2014). 24555697 10.1021/ol500267w

[38] Yasui, M., Ota, R., Tsukano, C. & Takemoto, Y. Synthesis of *cis*-/all-*cis*-substituted cyclopropanes through stereocontrolled metalation and Pd-catalyzed Negishi coupling. *Org. Lett.* **20**, 7656–7660 (2018). 30462517 10.1021/acs.orglett.8b03390

[39] Piou, T., Romanov-Michailidis, F., Ashley, M. A., Romanova-Michaelides, M. & Rovis, T. Stereodivergent rhodium(III)-catalyzed *cis*-cyclopropanation enabled by multivariate optimization. *J. Am. Chem. Soc.* **140**, 9587–9593 (2018). 30033723 10.1021/jacs.8b04243 PMC6112604

[40] Phipps, E. J. T. & Rovis, T. Rh(III)-catalyzed C–H activation-initiated directed cyclopropanation of allylic alcohols. *J. Am. Chem. Soc.* **141**, 6807–6811 (2019). 30998324 10.1021/jacs.9b02156 PMC6980370

[41] Costantini, M. & Mendoza, A. Modular enantioselective synthesis of *cis*-cyclopropanes through self-sensitized stereoselective photodecarboxylation with benzothiazolines. *ACS Catal.* **11**, 13312–13319 (2021). 34765283 10.1021/acscatal.1c03949 PMC8576787

[42] Mato, M., Herlé, B. & Echavarren, A. M. Cyclopropanation by gold- or zinc-catalyzed retro-Buchner reaction at room temperature. *Org. Lett.* **20**, 4341–4345 (2018). 29975067 10.1021/acs.orglett.8b01791

[43] James, G. T. Inactivation of the protease inhibitor phenylmethylsulfonyl fluoride in buffers. *Anal. Biochem.* **86**, 574–579 (1978). 26289 10.1016/0003-2697(78)90784-4

[44] Palermo, A. F., Chiu, B. S., Patel, P. & Rousseaux, S. A. Nickel-catalyzed reductive alkyne hydrocyanation enabled by malononitrile and a formaldehyde additive. *J. Am. Chem. Soc.* **145**, 24981–24989 (2023). 10.1021/jacs.3c10165 37924301

[45] Chen, S. et al. Diastereoselective synthesis of cyclopropanes bearing trifluoromethyl-substituted all-carbon quaternary centers from 2-trifluoromethyl-1,3-enynes beyond fluorine elimination. *Chem. Commun.* **55**, 3879–3882 (2019). 10.1039/c9cc00785g 30806442

[46] Antermite, D., Affron, D. P. & Bull, J. A. Regio- and stereoselective palladium-catalyzed C(*sp*^3^)–H arylation of pyrrolidines and piperidines with C(3) directing groups. *Org. Lett.* **20**, 3948–3952 (2018). 29897773 10.1021/acs.orglett.8b01521

[47] Blackmond, D. G. Reaction progress kinetic analysis: a powerful methodology for mechanistic studies of complex catalytic reactions. *Angew. Chem. Int. Ed.* **44**, 4302–4320 (2005). 10.1002/anie.200462544 15997457

[48] Hoshiya, N., Kobayashi, T., Arisawa, M. & Shuto, S. Palladium-catalyzed arylation of cyclopropanes via directing group-mediated C(*sp*^3^)–H bond activation to construct quaternary carbon centers: synthesis of *cis*- and *trans*-1,1,2-trisubstituted chiral cyclopropanes. *Org. Lett.* **15**, 6202–6205 (2013). 24279330 10.1021/ol4030452

[49] Nielsen, C. D.-T. & Burés, J. Visual kinetic analysis. *Chem. Sci.* **10**, 348–353 (2019). 30746083 10.1039/c8sc04698k PMC6335952

[50] Anslyn, E. V. & Dougherty, D. A. *Modern Physical Organic Chemistry* (Univ. Science Books, 2006).

[51] Goumont, R., Magnier, E., Kizilian, E. & Terrier, F. Acidity inversions of α-NO_2_ and α-SO_2_CF_3_ activated carbon acids as a result of contrasting solvent effects on transfer from water to dimethyl sulfoxide solutions. *J. Org. Chem.* **68**, 6566–6570 (2003). 12919016 10.1021/jo034244o

[52] Wagen, C. C. & Wagen, A. M. Efficient and accurate p*K*_a_ prediction enabled by pre-trained machine-learned interatomic potentials. Preprint at *ChemRxiv*10.26434/chemrxiv-2024-8489b (2024).

[53] Anstine, D. M., Zubatyuk, R. & Isayev, O. AIMNet2: a neural network potential to meet your neutral, charged, organic, and elemental-organic needs. *Chem. Sci.* **16**, 10228–10244 (2025). 40342914 10.1039/d4sc08572h PMC12057637

