# Supplementary material for: Alkyl sulfonyl fluorides as ambiphiles in the stereoselective palladium(II)-catalysed cyclopropanation of unactivated alkenes
Source: Nat Synth. 2025 Dec 16;5(2):281–9. doi: 10.1038/s44160-025-00925-1 (PMC12885951; doi:10.1038/s44160-025-00925-1)
Supplement: Supplementary file 1 — Experimental details, Supplementary Figs. 1–12 and Tables 1–36. [file 44160_2025_925_MOESM1_ESM.pdf]

nature synthesis

Supplementary information https://doi.org/10.1038/s44160-025-00925-1

Alkyl sulfonyl fluorides as ambiphiles in the stereoselective palladium(II)-catalysed cyclopropanation of unactivated alkenes

In the format provided by the authors and unedited

Table of Contents

General Procedures for Starting Material Preparation 3
General Procedure C for Cyclopropanation 3
Stability Tests 5
Pd(0) Salt Screening and Control Experiments 10
Fluorine Tracking Experiments 11
General Procedures for Kinetics Experiments 12
General Procedures for Product Binding Experiments 16
Density Functional Theory (DFT) Calculations 26
Representative Procedures and Analytical Data for Sulfonyl Fluoride Nucleophiles 87
Representative Procedures and Analytical Data for Nucleophile Scope 89
Representative Procedures and Analytical Data for Alkene Scope 96
Representative Procedures and Analytical Data for Directing Auxiliary Removal 101
Scale-Up Experiments 103
Base Screening at Lower Nucleophile Loading[a] 106
X-Ray Crystallography 107
References 156

General Information

Unless otherwise stated, all materials were used as received from commercial sources without further purification. All glassware and magnetic stir bars were dried in an oven at 100 °C overnight unless otherwise stated. All solvents were purchased from MilliporeSigma (Sure/SealTM) and used as received. 1-Dram (4-mL) reaction tubes were purchased from Fisher (Cat#: 50976409). Caps with TFE septa were purchased from Chemglass (Cat#: CG-4910-16). Ambient (room) temperature refers to 21–24 °C. Elevated temperatures were maintained by an IKA heating block for 1-dram vials or a silicone oil bath for larger vessels. Thin-layer chromatography (TLC) was performed using EMD Millipore 250 mm silica gel F-254 plates (250 μm) with F-254 fluorescent indicator and visualized by UV fluorescence quenching, iodine, Seebach's stain, or potassium permanganate stain. SiliCycle SiliaFlash P60 silica gel (particle size 40–63 μm) was used for flash chromatography. Analtech thin layer chromatography products (20 cm × 20 cm, 500 micron) were used for preparative thin layer chromatography (PTLC). ^{1}H, ^{13}C, and ^{19}F NMR spectra were recorded on a Bruker DRX equipped with a 5 mm DCH cryoprobe (500 MHz or 600 MHz, 600 MHz, and 399 MHz, respectively). ^{1}H NMR spectra were reported relative to Me_4Si (δ 0.0) unless otherwise stated. ^{13}C NMR spectra were calibrated to residual solvent signals ($CDCl_3$ at 77.16 ppm). High-resolution mass spectra (HRMS) were recorded on an Agilent LC/MSD TOF mass spectrometer by electrospray ionization time of flight experiments or atmospheric pressure chemical ionization time of flight experiments.

Commercial Suppliers of Chemicals:

The following chemicals were purchased from the suppliers indicated:

methyl-2-(fluorosulfonyl)acetate: Enamine (41505-91-3)
(dimethylcarbamoyl)methanesulfonyl chloride: Enamine (100481-28-5)
cyanomethanesulfonyl fluoride: Enamine (50408-65-6)
2-bromoprop-2-ene-1-sulfonyl fluoride: Enamine (1936692-86-2)
(4-bromo-1,2-oxazol-3-yl)methanesulfonyl fluoride: Enamine (2624142-00-1)
(4-bromo-1-methyl-1*H*-pyrazol-3-yl)methanesulfonyl fluoride: Enamine (2193059-36-6)
(4-nitrophenyl)methanesulfonyl fluoride: Enamine (110661-61-5)
(4-cyanophenyl)methanesulfonyl chloride: Enamine (56105-99-8)
methyl 4-[(fluorosulfonyl)methyl]benzoate: Enamine (1955558-09-4)
(4-bromo-2-fluorophenyl)methanesulfonyl fluoride: Enamine (240528-68-0)
(3-nitrophenyl)methanesulfonyl fluoride: Enamine (2137936-30-0)
(pyridin-4-yl)methanesulfonyl fluoride hydrochloride: Enamine (2361636-41-9)
(1-methyl-1H-pyrazol-3-yl)methanesulfonyl fluoride: Enamine (2137539-20-7)
(1-methyl-1H-1,2,3-triazol-4-yl)methanesulfonyl fluoride: Enamine (2172029-58-0)
(5-methyl-1,2-oxazol-3-yl)methanesulfonyl fluoride : Enamine (1311317-32-4)

All other commercial reagents were purchased from MilliporeSigma, Alfa Aesar, Oakwood, Combi-Blocks, TCI, or Strem and used as received.

General Procedures for Starting Material Preparation

General Procedure A1 for Synthesizing Sulfonyl Chlorides

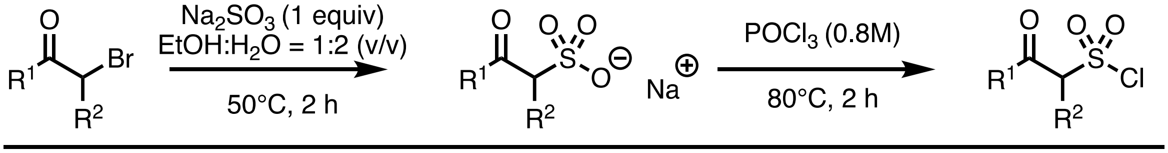

Org. Lett. **2020**, *22*, 8904–8909

The synthesis was performed according to a previous report from the literature.[1]

General Procedure A2 for Synthesizing Sulfonyl Chlorides

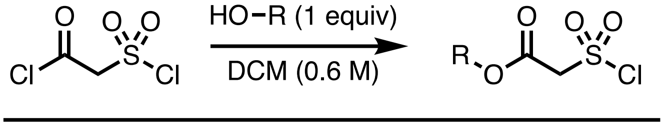

J. Org. Chem. **1997**, *62*, 8177–8181

Preparation of alkyl sulfonyl chlorides. The reaction was carried out according to a modified literature procedure.[2] To 2-(chlorosulfonyl)acetyl chloride (500 mg, 2.82 mmol) in anhydrous DCM (0.6 M) was slowly added the corresponding alcohol (1 equiv, 2.82 mmol) at 0 °C under nitrogen atmosphere. The reaction was stirred for 5 h. After this time period, the reaction was quenched with water. The aqueous solution was extracted with DCM (×3). The combined organic layers were dried over Na_2SO_4. The organic solvent was removed under reduced pressure. The crude product mixture was used without further purification.

General Procedure B for Synthesizing Sulfonyl Fluorides

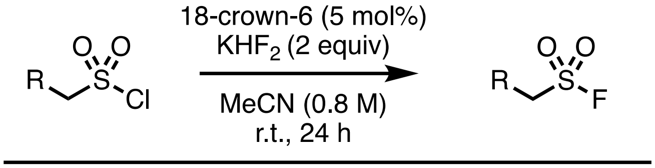

Angew. Chem. Int. Ed. **2014**, 53, 9430 – 9448

Preparation of alkyl sulfonyl fluorides. The synthesis was performed according to a previous report from the literature.[1,3] To the corresponding sulfonyl chloride in MeCN (0.8 M) were added 18-crown-6 (5 mol%) and potassium bifluoride (2 equiv). The reaction was stirred for 24 h at room temperature. After this time period, the reaction was quenched with water. The aqueous solution was extracted with ethyl acetate (×3). The combined organic layers were dried over Na_2SO_4. The organic solvent was removed under reduced pressure, and the residue was purified by column chromatography on silica gel with hexanes/ethyl acetate as the eluent to afford the product.

General Procedure C for Cyclopropanation

Outside of the glovebox, to an oven-dried 1-dram (4 mL) vial equipped with a magnetic stir bar were added the appropriate alkene substrate (0.1 mmol), $Pd(OAc)_2$ (0.01 mmol, 10 mol%), and

Na_2CO_3 (0.1 mmol, 1.0 equiv). The vial was then introduced into a nitrogen-filled glovebox antechamber. Once transferred inside the glovebox, alkyl sulfonyl fluoride (0.3 mmol, 3.0 equiv) was added to the vial followed by anhydrous DMA (0.3 mL, 0.33 M). The vial was sealed with a screw-top septum cap, removed from the glovebox, and placed in a heating block that was pre-heated to 80 °C for 14 h. After this time period, the resulting mixture was filtered through a pad of celite. Saturated $NaHCO_3$ solution (10 mL) was added to the filtrate, and the aqueous layer was extracted with ethyl acetate (3 × 2 mL). The combined organic layers were concentrated and purified by silica gel flash column chromatography with ethyl acetate/hexanes as the eluent.

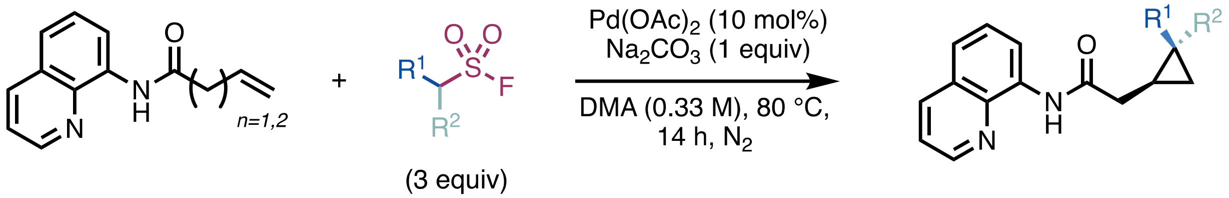

Stability Tests

(1) stability tests of 2a

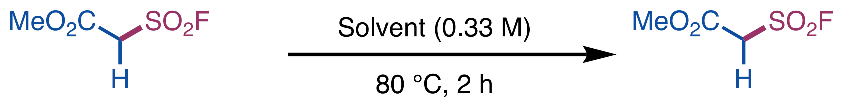

Under N_2 atmosphere. In the glovebox, to a 1-dram vial with a stir bar were added methyl-2-(fluorosulfonyl)acetate (15.6 mg, 0.1 mmol) and anhydrous or deuterated solvent. The reaction vial was capped and removed from the glovebox. 10% water was injected to the vial when necessary. The reaction vial was stirred for 2 h. After this time period, dibromomethane (7 μL, 1 equiv) was injected into the solution as the internal standard and $CDCl_3$ was added for NMR spectroscopy when appropriate.

Under Air atmosphere. In the glovebox, to a 1-dram vial with a stir bar were added methyl-2-(fluorosulfonyl)acetate (15.6 mg, 0.1 mmol) and anhydrous or deuterated solvent. The reaction vial was capped and removed from the glovebox. The cap was removed to expose the reaction mixture to air for 2 min. The vial was re-capped and stirred for an additional 2 h. After this time period, dibromomethane (7 μL, 1 equiv) was injected into the solution as the internal standard and $CDCl_3$ was added for NMR spectroscopy when appropriate.

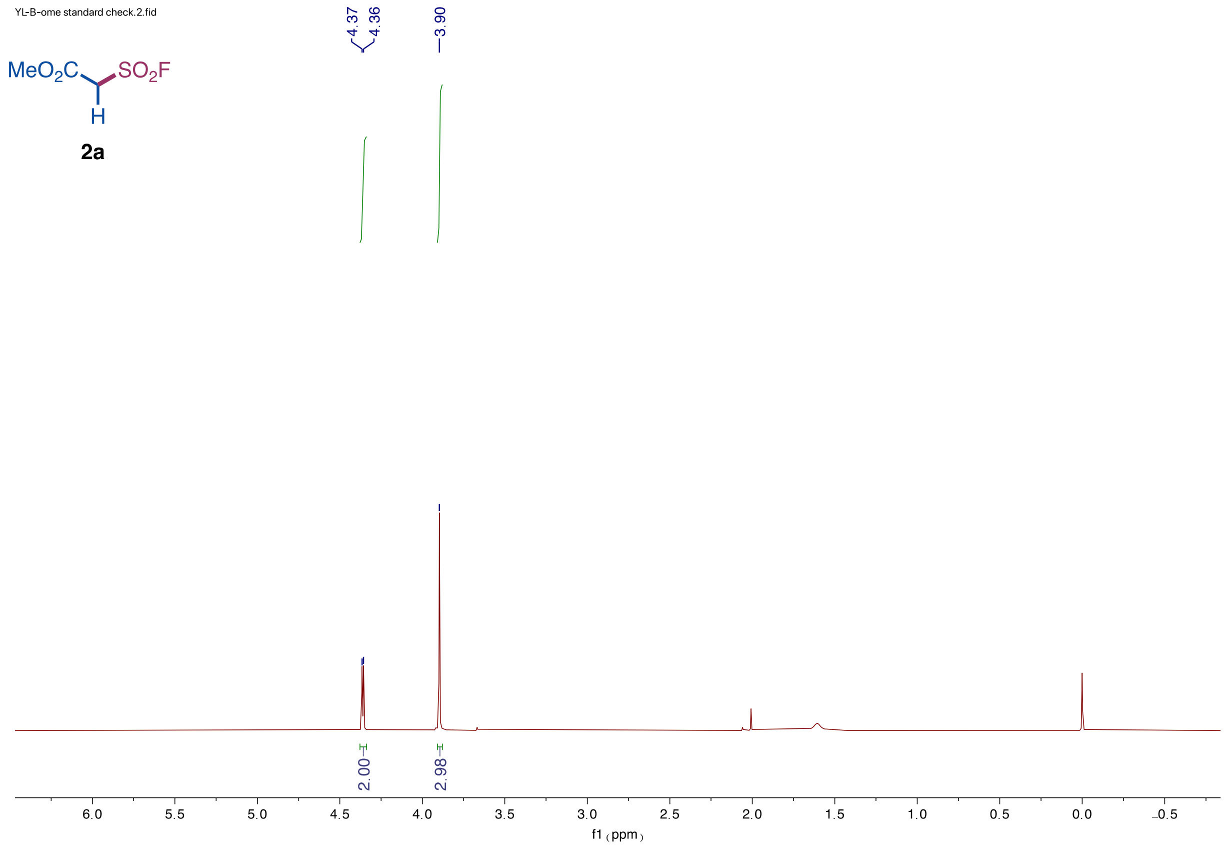

(1) **2a** in anhydrous DMA and 10% water: substrate peak not detected after the reaction

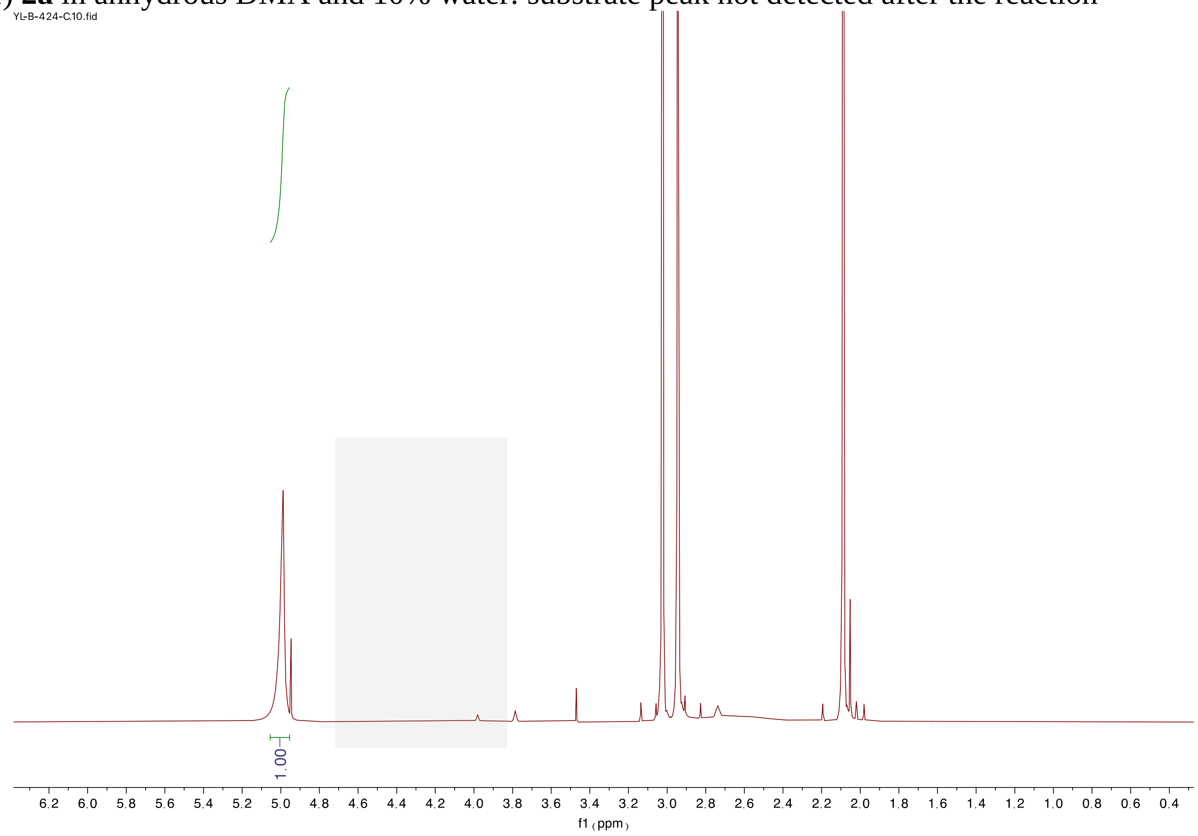

(2) **2a** in anhydrous DMA under air: 83%

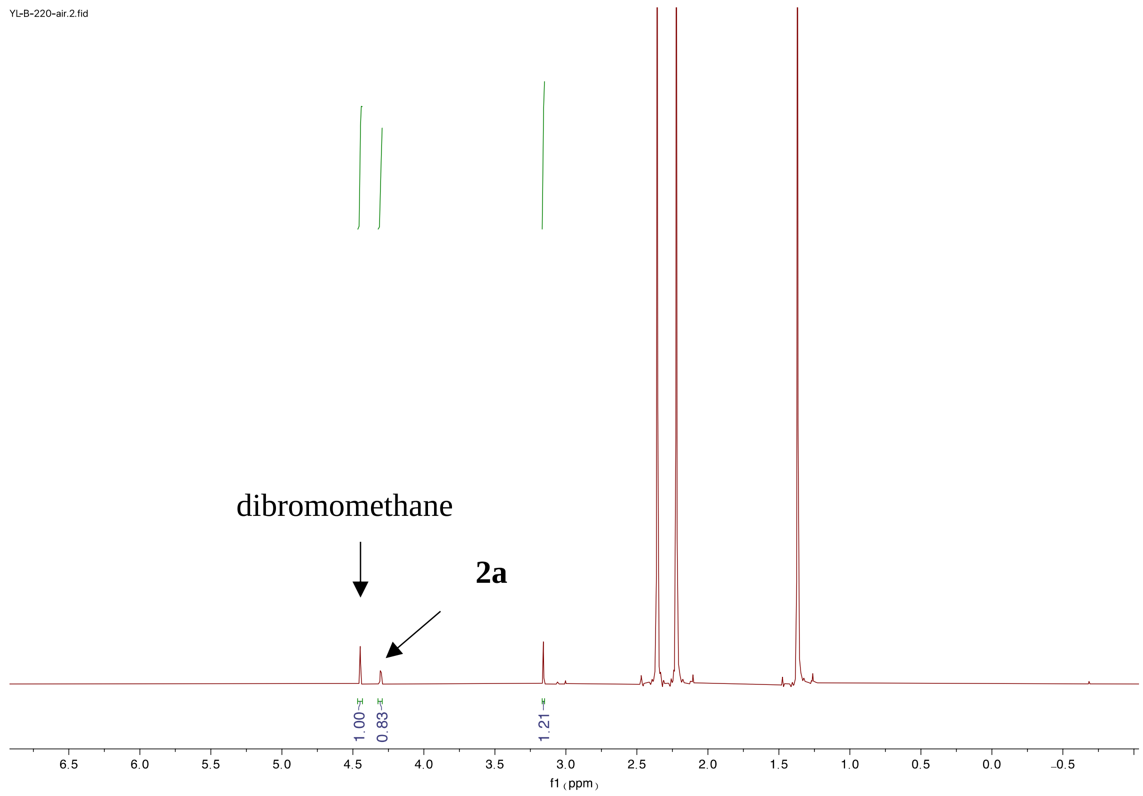

(3) **2a** in anhydrous DMA under N_2 atmosphere: 100%

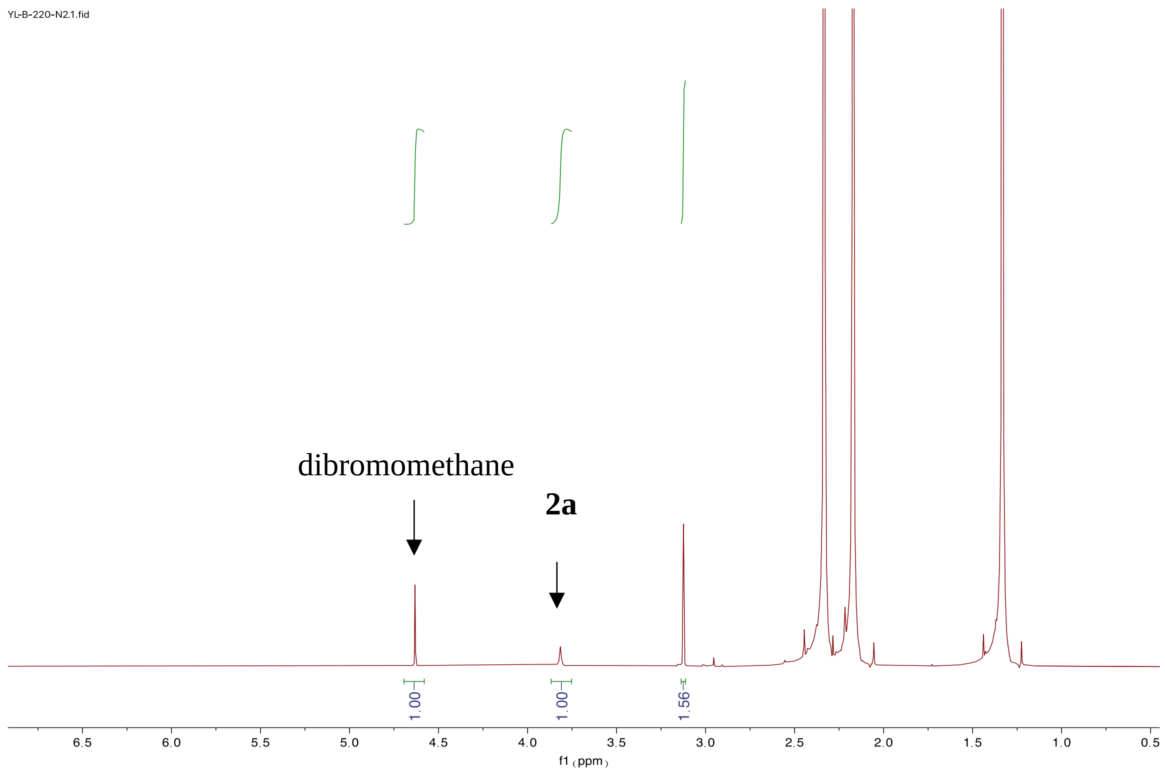

(4) **2a** in DMF-d_7 under N_2 atmosphere: 85%

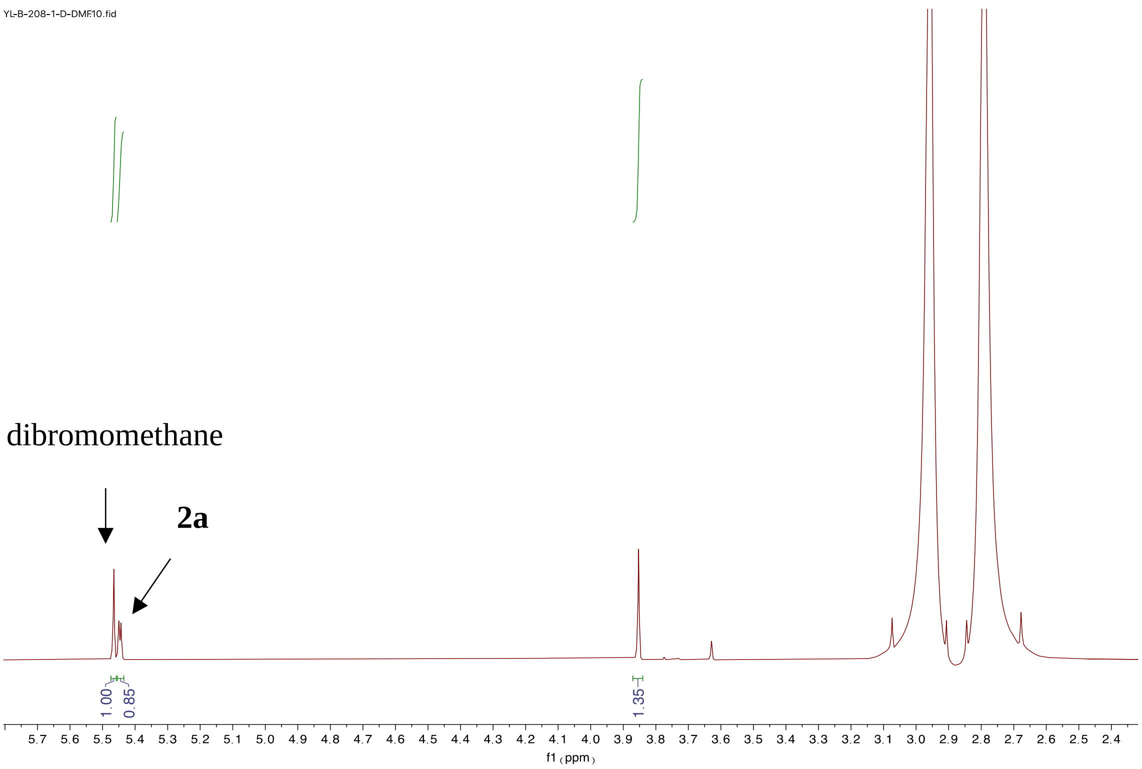

(5) **2a** in DMSO-d_6 under N_2 atmosphere: not detected

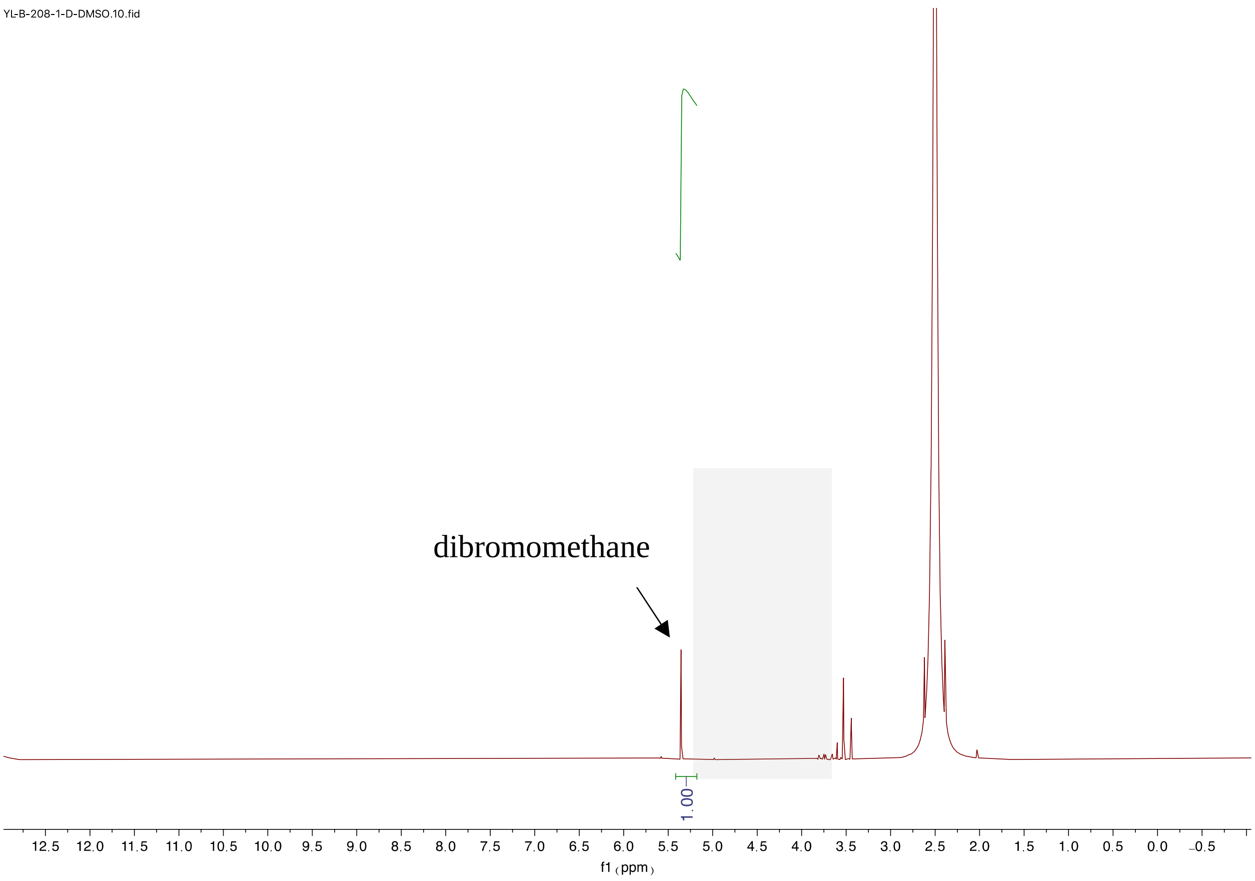

(2) stability test of methyl 2-(chlorosulfonyl)acetate

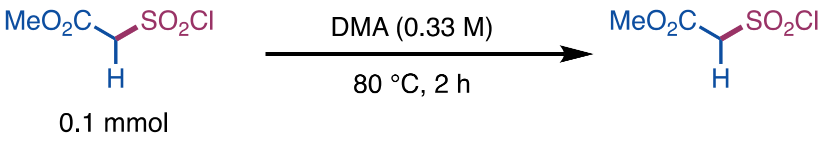

In the glovebox, to a 1-dram vial with a stir bar were added methyl 2-(chlorosulfonyl)acetate (17.2 mg, 0.1 mmol) and anhydrous DMA. The reaction vial was capped and removed from the glovebox and stirred for 2 hours. After this time, dibromomethane (7 μL, 1 equiv) was injected into the solution as the internal standard and $CDCl_3$ was added for NMR spectroscopy.

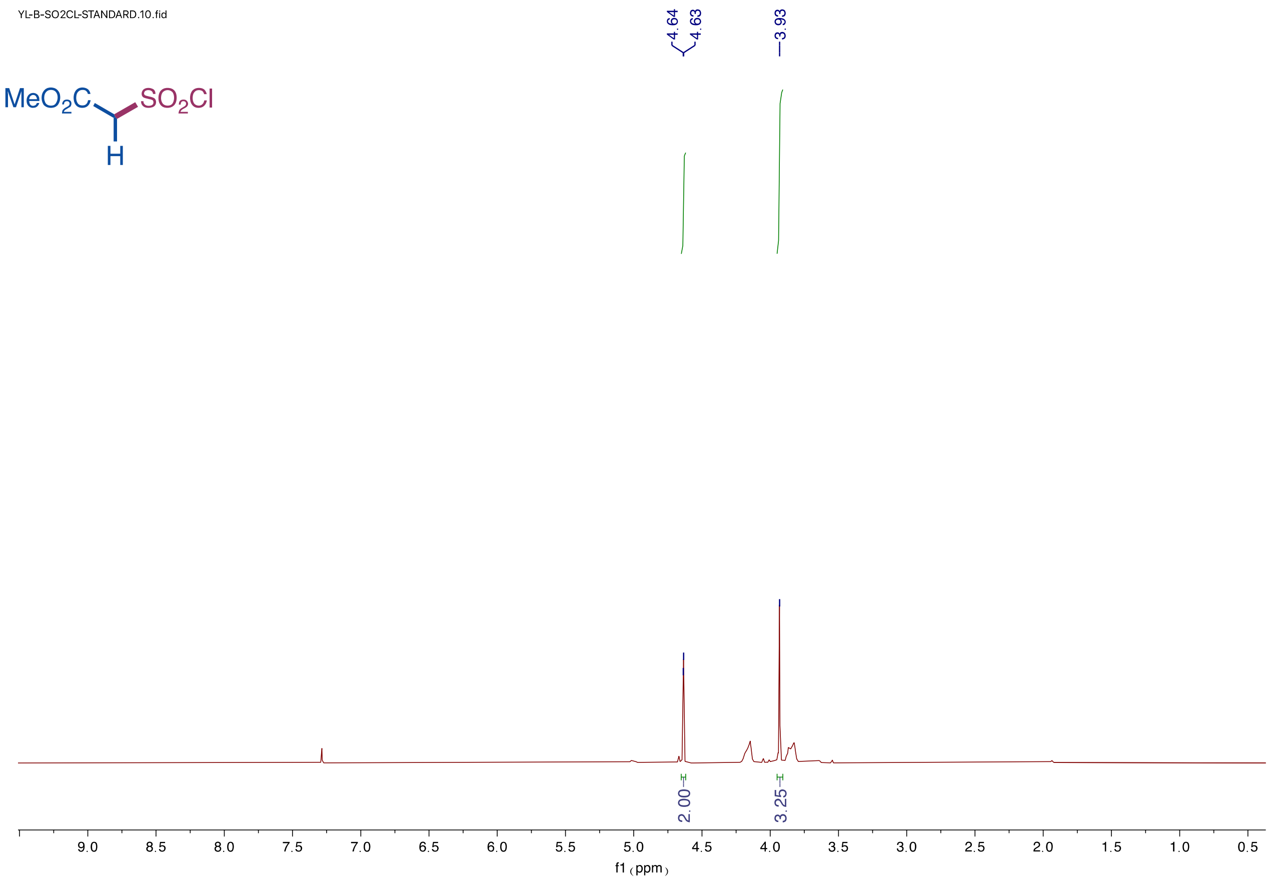

The NMR peaks of methyl 2-(chlorosulfonyl)acetate peaks were not detected.

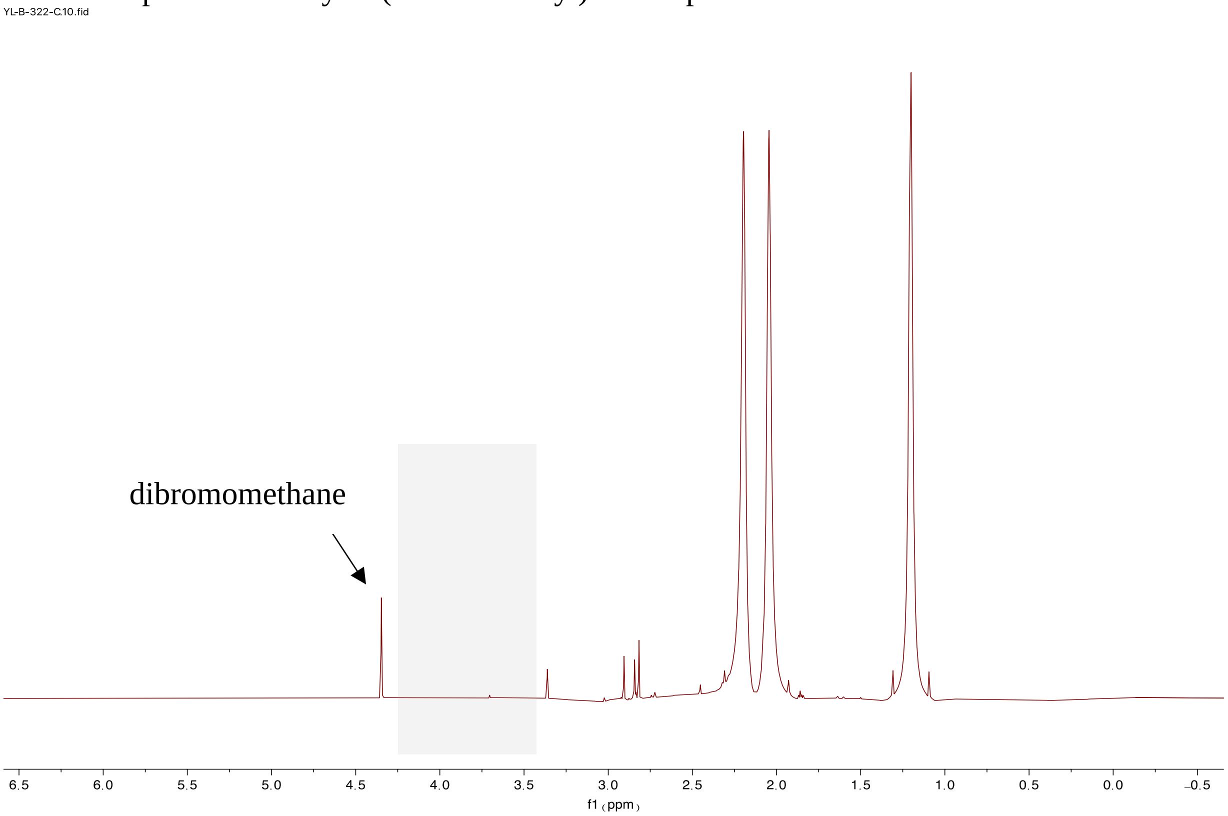

Pd(0) Salt Screening and Control Experiments

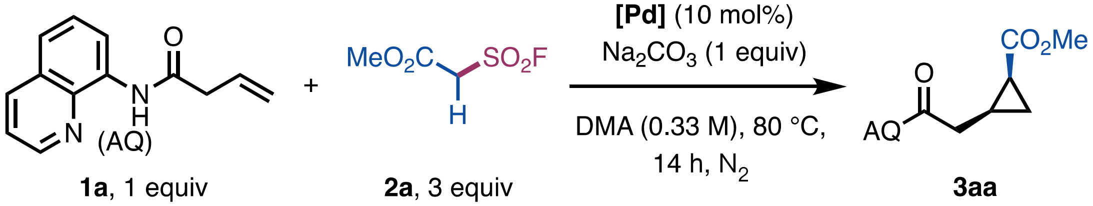

[Pd]	NMR yield	*d.r.*
Pd(COD)(DQ)	80%	96:4
$Pd_2(dba)_3$	91%	98:2
$Pd(PPh)_3$	n.d.	n.d.

[a]Reactions performed on 0.1 mmol scale. Yields were determined by ^{1}H NMR analysis of the crude reaction mixture with benzyl 4-fluorobenzoate as an internal standard. n.d. = not detected.

Control Experiment[4]

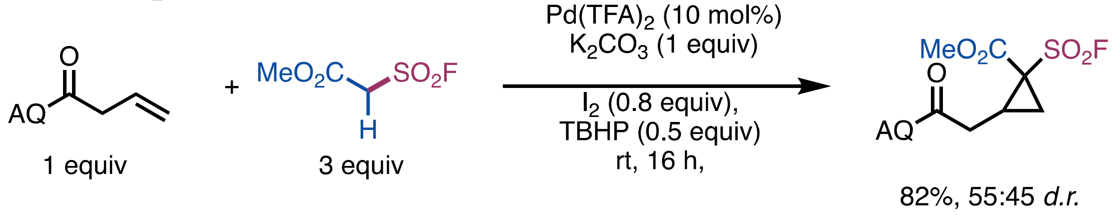

J. Am. Chem. Soc. **2024**, *146*, 24503–24514

Fluorine Tracking Experiments

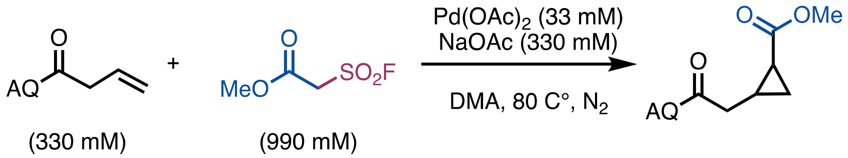

The standard reaction was tracked by ^{19}F NMR to determine the fate of the fluorine from SO_2F anion under catalytic conditions. It was observed that consumption of the nucleophile (*δ*= –57.80 ppm) was complemented by the gradual appearance of a singlet at δ= –178 ppm. This species appears in the region consistent with the ^{19}F NMR peak of HF in acetonitrile (δ = –186 ppm) [5]. We also performed an experiment where tetra-*n*-butylammonium fluoride (TBAF) was mixed with trifluoroacetic acid (TFA), which recapitulated formation of this new peak *in situ* (δ= –177 ppm). Our current hypothesis is that the SO_2F anion released from intramolecular oxidative addition decomposes into SO_2 gas and fluoride ion, with the latter speciating into bifluoride (FHF^-) under the reaction conditions.

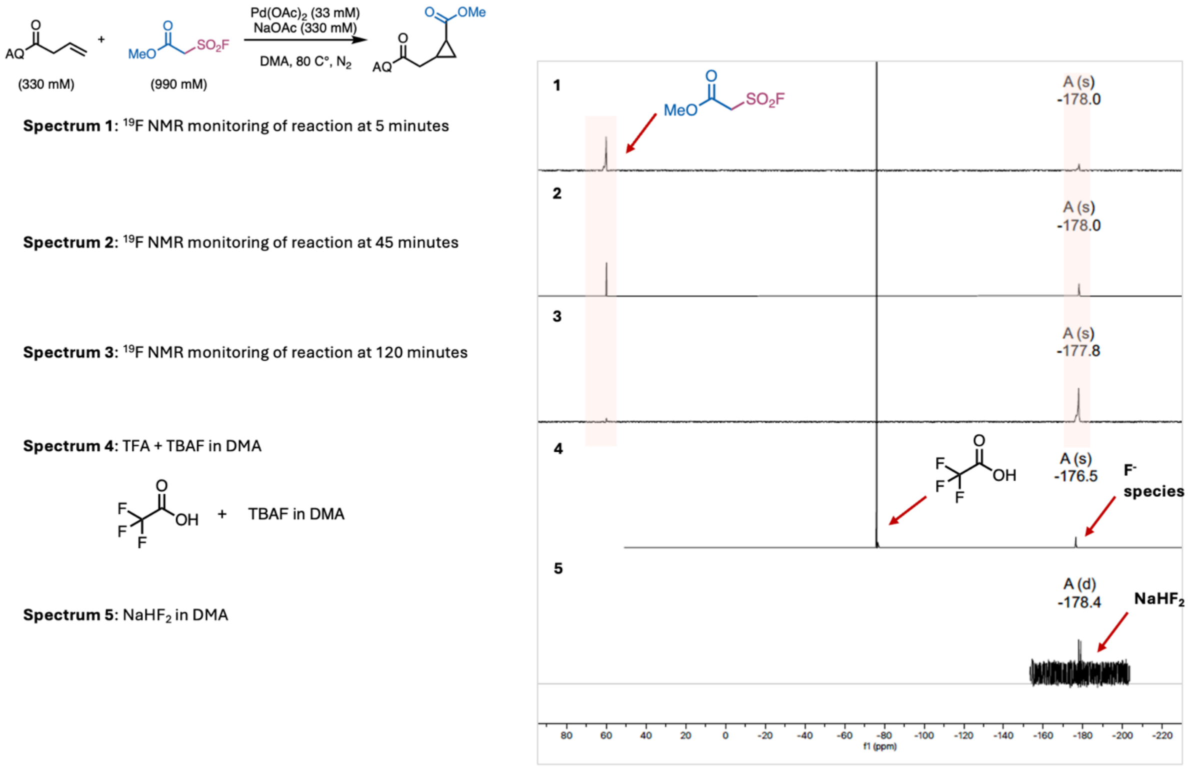

General Procedures for Kinetics Experiments

Preparation of stock solutions

1 M stock solutions of the alkene **1a** and 1,3,5-trimethoxybenzene as internal standard were prepared separately by addition of the corresponding solids (2 mmol) to 2-mL volumetric flasks and dissolution in anhydrous *N,N*-dimethylacetamide (DMA) in the glovebox.

General Kinetic Procedure

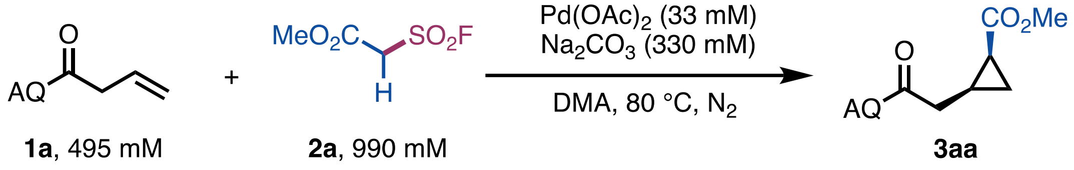

To a 13×100 mm screw thread tube were added $Pd(OAc)_2$ (11.1 mg, 0.0495 mmol, 33 mM), Na_2CO_3 (52.5 mg, 0.495 mmol, 330 mM), and a magnetic stir bar. The vial was brought into a nitrogen-filled glovebox, and the mixture was dissolved in 840 μL of anhydrous DMA. Then, the alkene substrate **1a** (495 μL, 0.495 mmol, 330 mM) and 1,3,5-trimethoxybenzene (165 μL, 0.165 mmol, 110 mM) were added successively from respective 1.0 M stock solutions. Lastly, the alkylsulfonyl fluoride nucleophile **2a** (177 μL, 1.490 mmol, 990 mM) was added. The vial is capped with a SURE-LINK open top septum cap and taped shut with electrical tape before it was brought out of the glovebox. Then, a nitrogen-filled rubber balloon was punctured into the vial and immediately placed into a thermocouple-controlled oil bath that was pre-heated to 80 °C with a stir rate of 750 rpm. This was considered t=0 min for the kinetic time course.

Reaction progress was monitored by removing an aliquot (~100 μL) from the reaction mixture. Each aliquot was acquired using a new syringe (1 mL) and a hypodermic needle. Each aliquot was quenched by injection into a screw thread tube containing $CDCl_3$ (500 μL) at room temperature. Each sample was analyzed through ^{1}H qNMR using a 600 MHz NMR spectrometer with 4 scans and a relaxation delay of 25 s.

Procedure for same excess experiments to probe catalyst robustness

Table S1. Same excess experiments were performed according to the general kinetic procedure with 33 mM $Pd(OAc)_2$ and 330 mM Na_2CO_3 in DMA.

Experiment	[Alkene 1a], mM	[Nucleophile 2a], mM	[Product 3aa], mM
Standard	330	990	-
Same excess	248	908	-
Same excess + product	248	908	83

The term "excess" refers to the difference between the initial concentration of the substrates of the reaction, particularly the alkene **1a** and the nucleophile **2a**. The same excess experiment simulates 25% conversion (83 mM) by performing the reaction at 0.75 equivalents of alkene **1a** (248 mM) and 2.75 equivalents of nucleophile **2a** (908 mM) relative to the standard conditions (330 mM and 990 mM, respectively). By keeping all else constant, the "excess" between **1a** and **2a** remains the same (660 mM) in both experiments.

From these experiments, a plot of alkene concentration (mM) versus time (min) was constructed. The same excess trace is translated (shifted) to match the standard trace on the time axis. The absence of overlay at higher conversions is indicative of either catalyst deactivation or product inhibition. To distinguish between the two possibilities, a third experiment where the product **3a** (83 mM) is added to the same excess reaction was performed, and the observed lack of overlay suggests mild catalyst deactivation.

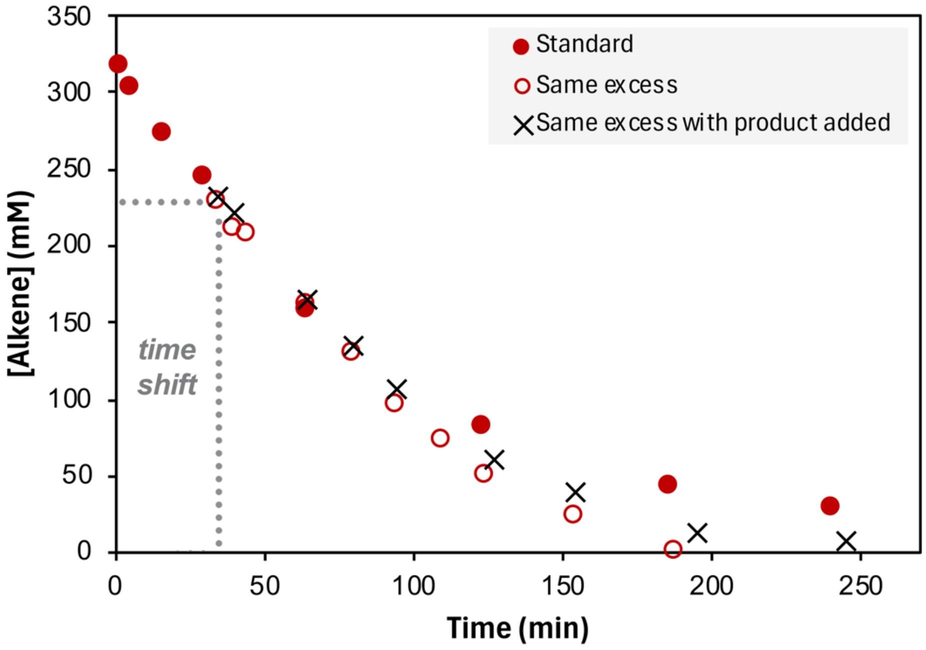

Figure S1. Same Excess Plot. The same excess traces were shifted by t=35 minutes.

Procedure for determining reaction orders using Variable Time Normalization Analysis (VTNA)

Table S2. Different excess experiments were performed according to the general kinetic procedure with 330 mM Na_2CO_3 in DMA.

Experiment	[Alkene 1a], mM	[Nucleophile 2a], mM	[Pd(OAc)$_2$], mM
Standard	330	990	33
Alkene diff excess	**231**	990	33
Nucleophile diff excess	330	**1980**	33
Catalyst diff excess	330	990	**17**

The term "excess" refers to the difference between the initial concentration of the substrates of the reaction, particularly the alkene **1a** and the nucleophile **2a**. A "different excess" experiment is performed for each component that we wish to determine the order of by changing only the concentration of that particular component and keeping all else constant. For instance, the order in the alkene **1a** was determined by performing an experiment with 0.70 equivalents of **1a** (231 mM) with respect to the standard conditions where [**1a**] = 330 mM. Similarly, the order of the

nucleophile **2a** was determined by performing an experiment with 6.0 equivalents of **2a** (1980 mM) instead of 3.0 equivalents (990 mM) under the standard conditions, and the order of the catalyst $Pd(OAc)_2$ was determined by performing an experiment with 5 mol% loading (16.5 mM) instead of 10 mol% loading (33 mM) under the standard conditions. The effects of these concentration changes are elucidated by plotting product concentration versus $\Sigma[\mathbf{1a}]^{\alpha}[\mathbf{2a}]^{\beta}[\mathbf{Pd}]^{\gamma}\Delta t$, the time-normalized scale in VTNA, wherein the exponents α, β, and γ correspond to the reaction orders in **1a**, **2a**, and $Pd(OAc)_2$, respectively.

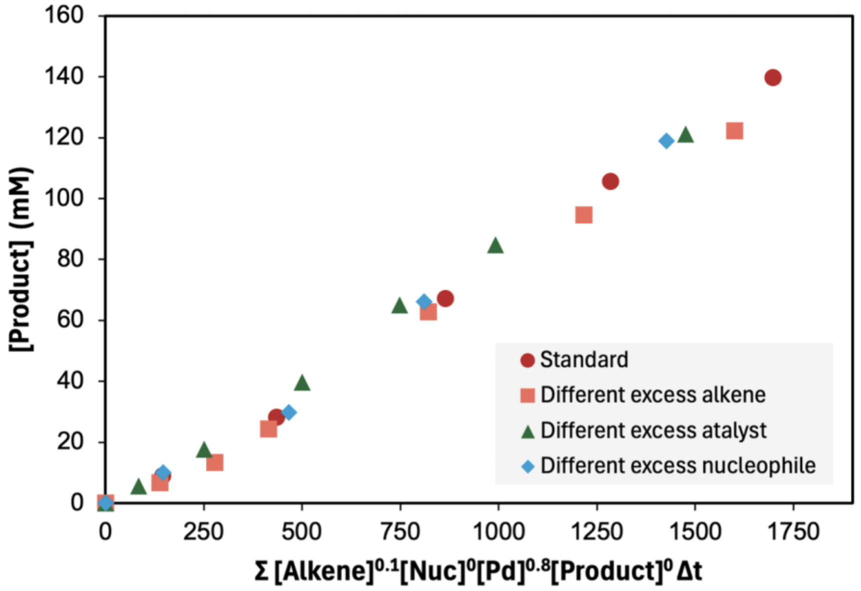

Figure S2. Different Excess Plot.

Procedure for Eyring Analysis

The experiments for Eyring analysis were performed according to the General Kinetic Procedures, with the difference being the temperatures (70 °C, 80 °C, 90 °C, and 100 °C) at which the reactions were monitored at. The oil baths were carefully controlled using a thermocouple. Initial rates were obtained from the slope of a linear-fit line monitoring the first 30% conversion of the reaction progress. The activation parameters were extracted from the slope of a plot of ln(k/T) versus 1/T.

Table S3. Initial rates for Eyring analysis were obtained by performing the experiments according to the general kinetic procedure at the stated temperature.

Initial rate (M/min)	kobs (1/s)	T, K	1/T, (K^-1)	ln(k/T)
0.001782	0.0009	343.15	0.0029142	-12.85128346
0.003828	0.0019333	353.15	0.0028317	-12.11540255
0.006072	0.0030667	363.15	0.0027537	-11.68198006
0.01386	0.007	373.15	0.0026799	-10.88382561

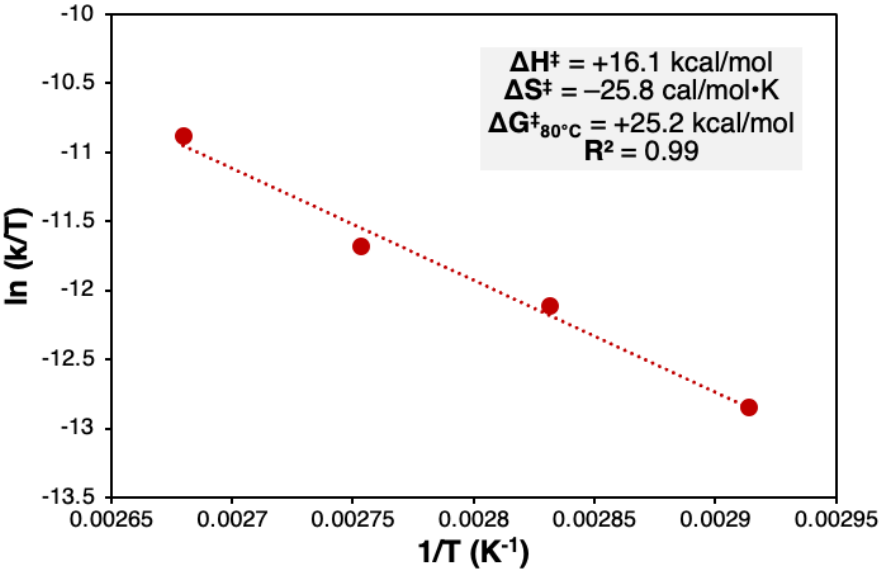

Figure S3. Eyring Plot.

Boltzmann constant (k_B) = 1.38×10^{-23} J/K
Planck constant (h) = 6.626×10^{-34} J•s
Gas constant (R) = 1.987 cal/mol•K
Linear-fitting equation = y = -8104.9x + 10.769 (R^2=0.987)
Activation enthalpy ($\Delta H^{\ddagger}$) = 16.1 kcal/mol
Activation entropy ($\Delta S^{\ddagger}$) = -25.8 cal/mol•K
Gibbs free energy of activation ($\Delta G^{\ddagger}_{353.15}$) = 25.2 kcal/mol

General Procedures for Product Binding Experiments

Procedure for the synthesis of C–H activated complex Pd-3aa.

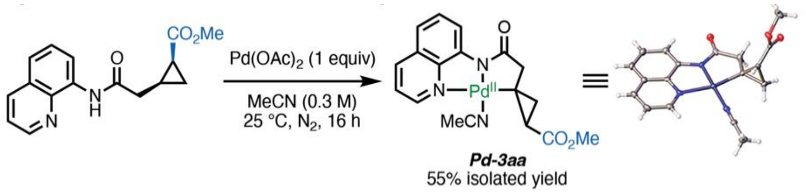

Pd-3aa: To a scintillation vial equipped with a magnetic stir bar were added $Pd(OAc)_2$ (225 mg, 1.00 mmol) and cyclopropane product **3aa** (284 mg, 1.00 mmol) inside a nitrogen-filled glovebox. The mixture was dissolved in anhydrous acetonitrile (3 mL) and stirred for 16 h at room temperature. The vial was then removed from the glovebox and triturated in diethyl ether. After vacuum filtration, the remaining solids were washed with DCM and chloroform sequentially into a clean scintillation vial to afford the final product as a green solid after concentration (237 mg, 55% yield). **^{1}H NMR** (600 MHz, $CDCl_3$) δ 9.06 (d, *J* = 7.9 Hz, 1H), 8.30–8.21 (m, 2H), 7.51 (t, *J* = 8.0 Hz, 1H), 7.36 (dd, *J* = 8.3, 4.6 Hz, 1H), 7.29 (d, *J* = 8.1 Hz, 1H), 3.68 (s, 3H), 2.97 (d, *J* = 26.8 Hz, 1H), 2.82 (d, *J* = 18.4 Hz, 1H), 1.70 (dd, *J* = 7.7, 5.1 Hz, 1H), 1.09 (dd, *J* = 7.7, 5.0 Hz, 1H). **^{13}C NMR** (151 MHz, $CDCl_3$) δ 184.50, 173.68, 147.02, 145.97, 144.94, 138.48, 129.89, 129.60, 120.91, 120.61, 118.61, 51.55, 50.81, 25.64, 23.46, 16.31, 3.61, 2.08. **HRMS** (ESI-TOF) Calcd for $C_{18}H_{18}N_3O_3$[102Pd] $[M+H]^+$ 426.0404, found 426.0397 . Single crystals of **Pd-3aa** suitable for X-ray diffraction were obtained from liquid/liquid diffusion of hexanes into a saturated solution of **Pd-3aa** in DCM (CCDC 2171739).[28]

Procedure for probing the catalytic competence of Pd-3aa.

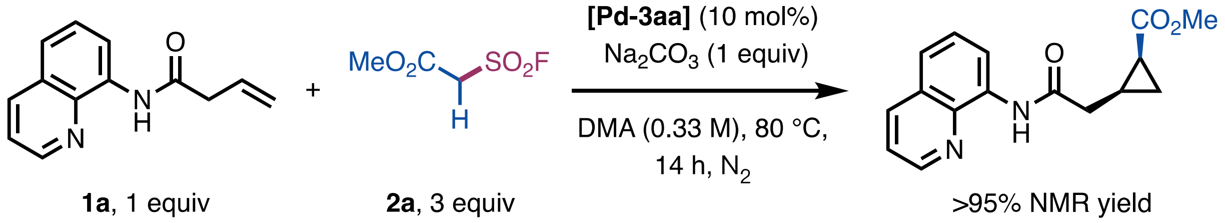

To a 1-dram clear glass vial with a magnetic stir bar were added the alkene substrate **1a** (42.5 mg, 0.20 mmol), sodium carbonate (21.2 mg, 0.20 mmol), 1,3,5-trimethoxybenzene (11.2 mg, 0.067 mmol), and complex **Pd-3aa** (8.60 mg, 0.02 mmol). The reaction vial was brought into a nitrogen-filled glovebox, and the mixture was dissolved in anhydrous DMA (606 µL). Finally, the alkylsulfonyl fluoride nucleophile **2a** (71.5 µL, 0.60 mmol) was added. The reaction vial was capped, taped shut with electrical tape, moved from the glovebox, and placed into an oil bath that was pre-heated to 80 °C with a stir rate of 750 rpm. After 16 h, the reaction vial was cooled to room temperature and diluted with $CDCl_3$ (1 mL) to ensure dissolution of reaction components. An aliquot (~100 µL) was removed from the reaction mixture which was used for NMR yield determination upon dilution to 600 µL with $CDCl_3$. The sample was analyzed through ^{1}H qNMR using a 600 MHz NMR spectrometer with 4 scans and a relaxation delay of 25 s.

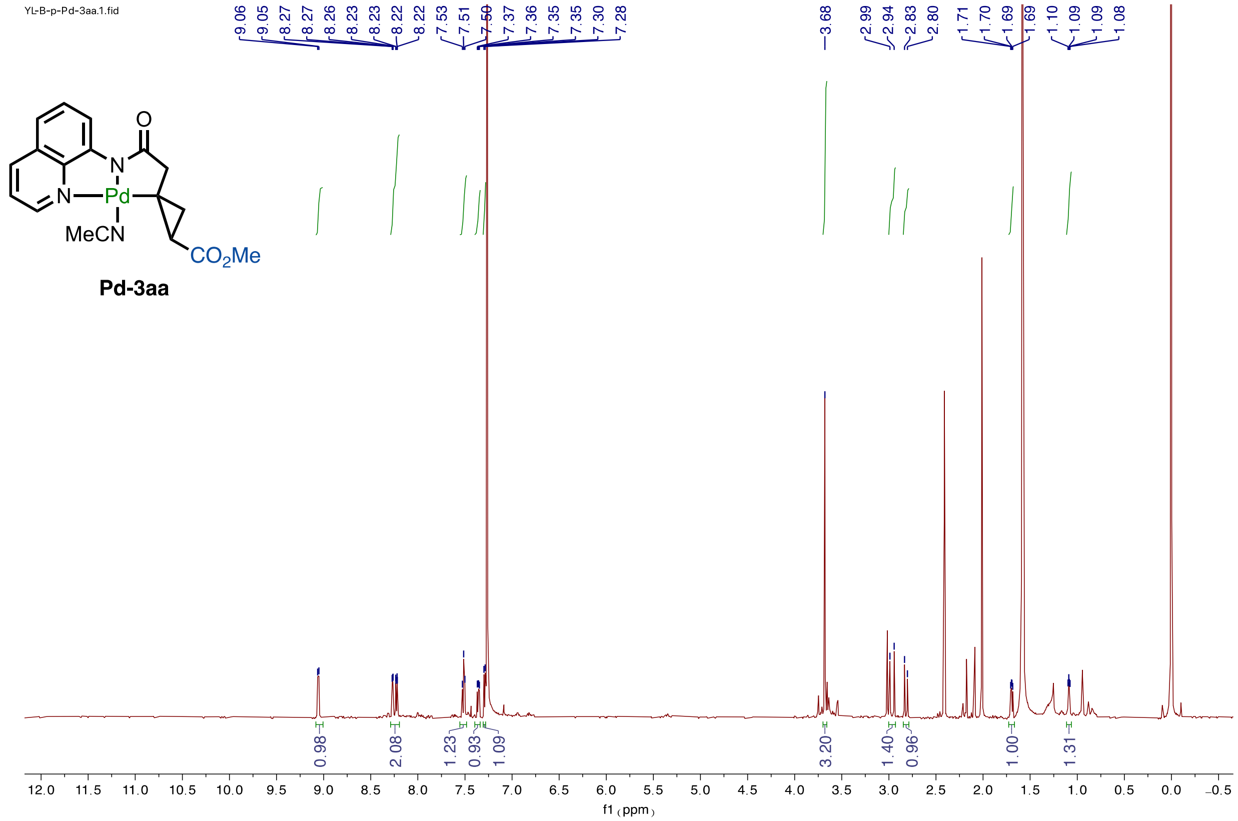
YL-B-p-Pd-3aa.1.fid
9.06 9.05 8.27 8.27 8.26 8.23 8.23 8.22 8.22 7.53 7.51 7.50 7.37 7.36 7.35 7.35 7.30 7.28
3.68
2.99 2.94 2.83 2.80
1.71 1.70 1.69 1.69
1.10 1.09 1.09 1.08
O
N
N
Pd
MeCN
CO_2Me
Pd-3aa
0.98
2.08
1.23
0.93
1.09
3.20
1.40
0.96
1.00
1.31
12.0 11.5 11.0 10.5 10.0 9.5 9.0 8.5 8.0 7.5 7.0 6.5 6.0 5.5 5.0 4.5 4.0 3.5 3.0 2.5 2.0 1.5 1.0 0.5 0.0 -0.5
f1 (ppm)

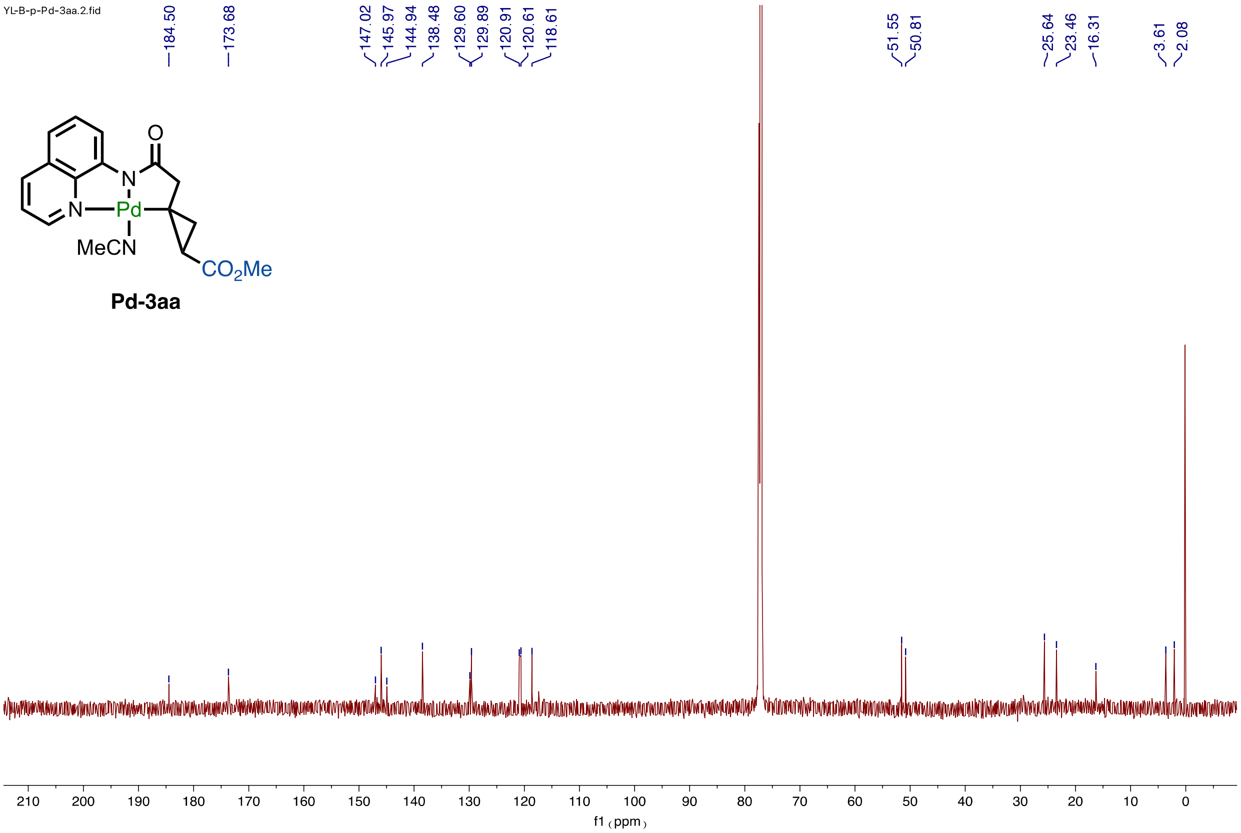
YL-B-p-Pd-3aa.2.fid
184.50
173.68
147.02 145.97 144.94 138.48 129.60 129.89 120.91 120.61 118.61
51.55 50.81
25.64 23.46 16.31
3.61 2.08
O
N
N
Pd
MeCN
CO_2Me
Pd-3aa
210 200 190 180 170 160 150 140 130 120 110 100 90 80 70 60 50 40 30 20 10 0
f1 (ppm)

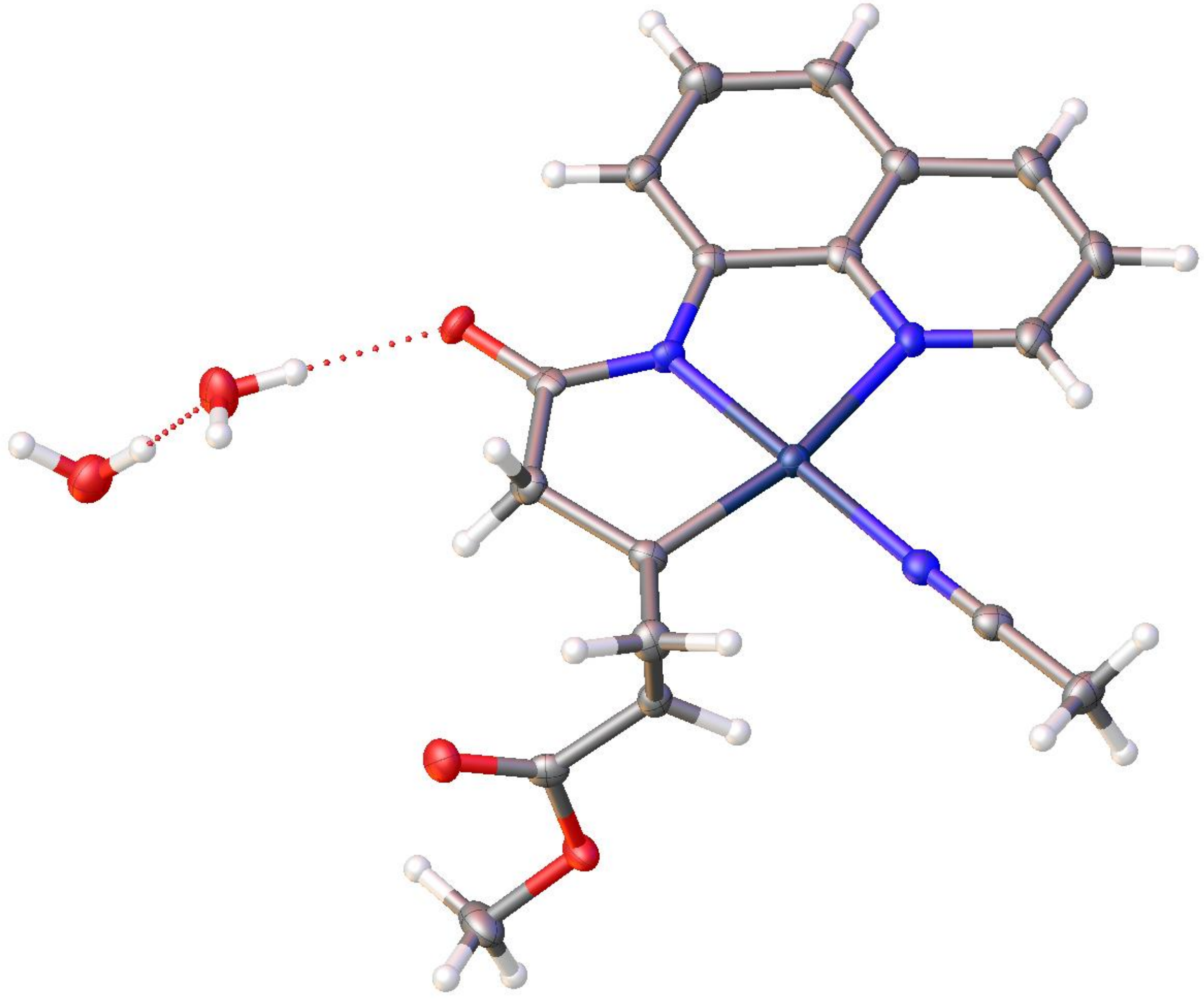

Experimental Summary for Pd-3aa

The single crystal X-ray diffraction studies were carried out on a Bruker D8-Venture 3-circle diffractometer equipped with a Photon3 detector and Mo K_{α} radiation (λ = 0.7107 Å).

Crystals of the subject compound were used as received. A 0.16 x 0.03 x 0.02 mm piece of a crystal was mounted on a Cryoloop with Paratone oil. Data were collected in a nitrogen gas stream at 100(2) K using ω scans. Crystal-to-detector distance was 50 mm and exposure time was 1 or 10 seconds using a scan width of 0.70°. Data collection was 99.7 % complete to 25.242° in θ. A total of 20507 reflections were collected covering the indices, −35<=h<=28, −32<=k<=34,

−5<=l<=5. 3811 reflections were found to be symmetry independent, with a R_{int} of 0.0579. Indexing and unit cell refinement indicated a **Tetragonal** lattice. The space group was found to be ***$P4_2/n$***. The data were integrated using the Bruker SAINT Software program and scaled using the SADABS software program. Solution by direct methods (SHELXT) produced a complete phasing model consistent with the proposed structure.

All nonhydrogen atoms were refined anisotropically by full-matrix least-squares (SHELXL-2014). All carbon bonded hydrogen atoms were placed using a riding model. Their positions were constrained relative to their parent atom using the appropriate HFIX command in SHELXL-2014. Crystallographic data are summarized in Table S4.

Notes: Great data! The sample is racemic. There is one copy of the compound, two water molecules, and a molecule of Acetonitrile in the asymmetric unit. The chemical formula for the compound is: $C_{18}H_{17}N_3O_3Pd*(H_2O)_2$

Table S4. Crystal data and structure refinement for **Pd-3aa** (CCDC 2171739).

Identification code	engle411_0m_a	
Empirical formula	C18 H21 N3 O5 Pd	
Formula weight	465.78	
Temperature	100 K	
Wavelength	0.71073 Å	
Crystal system	Tetragonal	
Space group	$P4_2/n$	
Unit cell dimensions	a = 28.171(3) Å	α= 90°.
	b = 28.171(3) Å	β= 90°.
	c = 4.7277(6) Å	γ = 90°.
Volume	3751.9(10) $Å^3$	
Z	8	
Density (calculated)	1.649 Mg/m^3	
Absorption coefficient	1.024 mm^{-1}	
F(000)	1888	
Crystal size	0.16 x 0.03 x 0.02 mm^3	
Theta range for data collection	2.892 to 26.419°.	
Index ranges	-35<=h<=28, -32<=k<=34, -5<=l<=5	
Reflections collected	20507	
Independent reflections	3811 [R(int) = 0.0579]	
Completeness to theta = 25.242°	99.7 %	
Absorption correction	Semi-empirical from equivalents	
Max. and min. transmission	0.4908 and 0.4367	
Refinement method	Full-matrix least-squares on F^2	
Data / restraints / parameters	3811 / 0 / 252	
Goodness-of-fit on F^2	1.077	
Final R indices [I>2sigma(I)]	R1 = 0.0365, wR2 = 0.0666	
R indices (all data)	R1 = 0.0512, wR2 = 0.0714	
Largest diff. peak and hole	0.543 and -0.558 e.$Å^{-3}$	

Table S5. Atomic coordinates (x 10^4) and equivalent isotropic displacement parameters (Å^{2}x 10^3) for **Pd-3aa**. U(eq) is defined as one third of the trace of the orthogonalized U^{ij} tensor.

	x	y	z	U(eq)
Pd(1)	2730(1)	5020(1)	6701(1)	14(1)
O(2S)	284(1)	4555(1)	-2631(6)	28(1)
O(3)	2140(1)	3411(1)	4766(5)	20(1)
O(1)	1478(1)	5359(1)	2893(5)	18(1)
O(2)	1499(1)	3732(1)	6884(6)	21(1)
N(3)	3196(1)	4695(1)	9293(6)	18(1)
O(1S)	599(1)	4995(1)	2380(6)	27(1)
N(1)	3189(1)	5507(1)	4698(6)	16(1)
N(2)	2263(1)	5339(1)	4279(6)	14(1)
C(8)	2440(1)	5703(1)	2521(7)	15(1)
C(10)	1806(1)	5193(1)	4375(7)	16(1)
C(12)	2179(1)	4605(1)	7765(7)	16(1)
C(9)	2943(1)	5782(1)	2782(7)	16(1)
C(5)	2906(1)	6409(1)	-759(8)	23(1)
C(11)	1719(1)	4818(1)	6573(8)	18(1)
C(2)	3911(1)	5889(1)	3305(9)	24(1)
C(13)	2155(1)	4317(1)	10381(8)	20(1)
C(6)	2424(1)	6337(1)	-948(8)	23(1)
C(14)	2274(1)	4062(1)	7649(7)	16(1)
C(15)	1923(1)	3732(1)	6468(7)	17(1)
C(7)	2190(1)	5987(1)	665(8)	18(1)
C(3)	3674(1)	6169(1)	1414(8)	24(1)
C(16)	1829(1)	3078(1)	3358(10)	30(1)
C(1)	3653(1)	5566(1)	4948(8)	21(1)
C(4)	3175(1)	6125(1)	1124(7)	18(1)
C(17)	3454(1)	4530(1)	10880(7)	18(1)
C(18)	3779(1)	4322(1)	12903(8)	24(1)

Table S6.   Bond lengths [Å] and angles [°] for **Pd-3aa**.

Pd(1)-N(3)	2.016(3)	C(6)-C(7)	1.410(5)
Pd(1)-N(1)	2.109(3)	C(14)-H(14)	1.0000
Pd(1)-N(2)	1.963(3)	C(14)-C(15)	1.468(4)
Pd(1)-C(12)	2.007(3)	C(7)-H(7)	0.9500
O(2S)-H(2SA)	0.8497	C(3)-H(3)	0.9500
O(2S)-H(2SB)	0.8508	C(3)-C(4)	1.420(5)
O(3)-C(15)	1.355(4)	C(16)-H(16A)	0.9800
O(3)-C(16)	1.446(4)	C(16)-H(16B)	0.9800
O(1)-C(10)	1.252(4)	C(16)-H(16C)	0.9800
O(2)-C(15)	1.211(4)	C(1)-H(1)	0.9500
N(3)-C(17)	1.142(4)	C(17)-C(18)	1.448(5)
O(1S)-H(1SA)	0.8496	C(18)-H(18A)	0.9800
O(1S)-H(1SB)	0.8504	C(18)-H(18B)	0.9800
N(1)-C(9)	1.378(4)	C(18)-H(18C)	0.9800
N(1)-C(1)	1.323(4)		
N(2)-C(8)	1.409(4)	N(3)-Pd(1)-N(1)	99.75(11)
N(2)-C(10)	1.353(4)	N(2)-Pd(1)-N(3)	178.23(12)
C(8)-C(9)	1.441(4)	N(2)-Pd(1)-N(1)	81.36(11)
C(8)-C(7)	1.379(5)	N(2)-Pd(1)-C(12)	83.94(12)
C(10)-C(11)	1.502(5)	C(12)-Pd(1)-N(3)	95.03(12)
C(12)-C(11)	1.533(4)	C(12)-Pd(1)-N(1)	164.77(12)
C(12)-C(13)	1.481(5)	H(2SA)-O(2S)-H(2SB)	104.4
C(12)-C(14)	1.554(4)	C(15)-O(3)-C(16)	115.7(3)
C(9)-C(4)	1.405(5)	C(17)-N(3)-Pd(1)	175.9(3)
C(5)-H(5)	0.9500	H(1SA)-O(1S)-H(1SB)	104.5
C(5)-C(6)	1.378(5)	C(9)-N(1)-Pd(1)	110.7(2)
C(5)-C(4)	1.415(5)	C(1)-N(1)-Pd(1)	130.3(2)
C(11)-H(11A)	0.9900	C(1)-N(1)-C(9)	118.9(3)
C(11)-H(11B)	0.9900	C(8)-N(2)-Pd(1)	116.2(2)
C(2)-H(2)	0.9500	C(10)-N(2)-Pd(1)	118.5(2)
C(2)-C(3)	1.366(5)	C(10)-N(2)-C(8)	125.3(3)
C(2)-C(1)	1.401(5)	N(2)-C(8)-C(9)	114.2(3)
C(13)-H(13A)	0.9900	C(7)-C(8)-N(2)	128.0(3)
C(13)-H(13B)	0.9900	C(7)-C(8)-C(9)	117.8(3)
C(13)-C(14)	1.515(5)	O(1)-C(10)-N(2)	124.8(3)
C(6)-H(6)	0.9500	O(1)-C(10)-C(11)	122.0(3)

N(2)-C(10)-C(11)	113.1(3)
C(11)-C(12)-Pd(1)	109.5(2)
C(11)-C(12)-C(14)	121.1(3)
C(13)-C(12)-Pd(1)	124.3(2)
C(13)-C(12)-C(11)	118.8(3)
C(13)-C(12)-C(14)	59.8(2)
C(14)-C(12)-Pd(1)	115.5(2)
N(1)-C(9)-C(8)	117.6(3)
N(1)-C(9)-C(4)	121.4(3)
C(4)-C(9)-C(8)	121.0(3)
C(6)-C(5)-H(5)	120.5
C(6)-C(5)-C(4)	119.0(3)
C(4)-C(5)-H(5)	120.5
C(10)-C(11)-C(12)	113.0(3)
C(10)-C(11)-H(11A)	109.0
C(10)-C(11)-H(11B)	109.0
C(12)-C(11)-H(11A)	109.0
C(12)-C(11)-H(11B)	109.0
H(11A)-C(11)-H(11B)	107.8
C(3)-C(2)-H(2)	120.5
C(3)-C(2)-C(1)	119.0(3)
C(1)-C(2)-H(2)	120.5
C(12)-C(13)-H(13A)	117.5
C(12)-C(13)-H(13B)	117.5
C(12)-C(13)-C(14)	62.5(2)
H(13A)-C(13)-H(13B)	114.6
C(14)-C(13)-H(13A)	117.5
C(14)-C(13)-H(13B)	117.5
C(5)-C(6)-H(6)	119.1
C(5)-C(6)-C(7)	121.9(3)
C(7)-C(6)-H(6)	119.1
C(12)-C(14)-H(14)	115.7
C(13)-C(14)-C(12)	57.7(2)
C(13)-C(14)-H(14)	115.7
C(15)-C(14)-C(12)	121.4(3)
C(15)-C(14)-C(13)	118.3(3)
C(15)-C(14)-H(14)	115.7
O(3)-C(15)-C(14)	110.2(3)
O(2)-C(15)-O(3)	122.7(3)
O(2)-C(15)-C(14)	127.1(3)
C(8)-C(7)-C(6)	120.8(3)
C(8)-C(7)-H(7)	119.6
C(6)-C(7)-H(7)	119.6
C(2)-C(3)-H(3)	120.1
C(2)-C(3)-C(4)	119.8(3)
C(4)-C(3)-H(3)	120.1
O(3)-C(16)-H(16A)	109.5
O(3)-C(16)-H(16B)	109.5
O(3)-C(16)-H(16C)	109.5
H(16A)-C(16)-H(16B)	109.5
H(16A)-C(16)-H(16C)	109.5
H(16B)-C(16)-H(16C)	109.5
N(1)-C(1)-C(2)	123.1(3)
N(1)-C(1)-H(1)	118.5
C(2)-C(1)-H(1)	118.5
C(9)-C(4)-C(5)	119.4(3)
C(9)-C(4)-C(3)	117.8(3)
C(5)-C(4)-C(3)	122.8(3)
N(3)-C(17)-C(18)	179.7(4)
C(17)-C(18)-H(18A)	109.5
C(17)-C(18)-H(18B)	109.5
C(17)-C(18)-H(18C)	109.5
H(18A)-C(18)-H(18B)	109.5
H(18A)-C(18)-H(18C)	109.5
H(18B)-C(18)-H(18C)	109.5

Symmetry transformations used to generate equivalent atoms:

Table S7. Anisotropic displacement parameters ($Å^2$x 10^3) for **Pd-3aa**. The anisotropic displacement factor exponent takes the form: $-2\pi^2[h^2 a^{*2}U^{11} + ... + 2 h k a^* b^* U^{12}]$

	U^{11}	U^{22}	U^{33}	U^{23}	U^{13}	U^{12}
Pd(1)	15(1)	14(1)	14(1)	-1(1)	-2(1)	1(1)
O(2S)	25(1)	31(2)	27(2)	4(1)	-3(1)	2(1)
O(3)	17(1)	17(1)	27(2)	-6(1)	3(1)	-3(1)
O(1)	14(1)	20(1)	21(1)	3(1)	-3(1)	5(1)
O(2)	18(1)	24(1)	22(1)	1(1)	3(1)	-3(1)
N(3)	19(2)	16(1)	18(2)	-3(1)	-1(1)	-1(1)
O(1S)	20(1)	35(2)	26(2)	9(1)	1(1)	-6(1)
N(1)	14(1)	15(1)	20(2)	-5(1)	-3(1)	0(1)
N(2)	17(1)	14(1)	13(2)	2(1)	-1(1)	1(1)
C(8)	18(2)	14(2)	12(2)	-3(1)	-1(1)	0(1)
C(10)	19(2)	12(2)	17(2)	-5(1)	5(2)	3(1)
C(12)	20(2)	15(2)	12(2)	0(1)	0(1)	-2(1)
C(9)	18(2)	17(2)	12(2)	-6(1)	-1(1)	0(1)
C(5)	32(2)	19(2)	18(2)	3(2)	2(2)	-6(2)
C(11)	16(2)	19(2)	19(2)	0(2)	4(2)	1(1)
C(2)	17(2)	28(2)	29(2)	-6(2)	-1(2)	-6(1)
C(13)	22(2)	23(2)	14(2)	2(2)	-1(2)	-1(1)
C(6)	27(2)	23(2)	20(2)	3(2)	-1(2)	2(2)
C(14)	16(2)	17(2)	14(2)	2(1)	-1(1)	-1(1)
C(15)	23(2)	15(2)	12(2)	3(1)	3(2)	1(1)
C(7)	18(2)	18(2)	20(2)	-2(1)	-1(2)	2(1)
C(3)	22(2)	24(2)	26(2)	-4(2)	2(2)	-8(1)
C(16)	27(2)	24(2)	40(3)	-12(2)	3(2)	-7(2)
C(1)	19(2)	20(2)	23(2)	-6(2)	-3(2)	1(1)
C(4)	21(2)	17(2)	17(2)	-2(1)	0(1)	-1(1)
C(17)	23(2)	16(2)	15(2)	-3(1)	-1(2)	-1(1)
C(18)	24(2)	25(2)	21(2)	-1(2)	-6(2)	6(1)

Table S8. Hydrogen coordinates (x 10^4) and isotropic displacement parameters ($Å^2$x 10^3) for **Pd-3aa**.

	x	y	z	U(eq)
H(2SA)	1	4657	-2487	42
H(2SB)	437	4706	-1365	42
H(1SA)	880	5106	2394	41
H(1SB)	572	4854	3962	41
H(5)	3057	6645	-1876	27
H(11A)	1527	4560	5728	22
H(11B)	1534	4957	8145	22
H(2)	4246	5914	3504	29
H(13A)	1837	4260	11212	24
H(13B)	2413	4357	11782	24
H(6)	2243	6529	-2202	28
H(14)	2614	3967	7417	19
H(7)	1857	5945	472	22
H(3)	3844	6392	296	28
H(16A)	2014	2881	2056	45
H(16B)	1586	3253	2295	45
H(16C)	1675	2875	4770	45
H(1)	3818	5380	6304	25
H(18A)	3991	4100	11929	35
H(18B)	3598	4152	14356	35
H(18C)	3967	4574	13794	35

Density Functional Theory (DFT) Calculations

Computational Methods

DFT calculations were performed using the Gaussian 16 program.[6] Geometries of all stationary points were optimized in the gas phase with the dispersion-corrected[7,8] B3LYP-D3(BJ) functional[9,10], using the SDD basis set for palladium[11,12] and the 6-31G(d) basis set for all other atoms. Vibrational frequency calculations were conducted at the same level of theory to confirm whether each structure is a local minimum or a transition state. Single-point energy calculations were carried out using the ωB97X-D functional[13,14] with the def2-TZVP basis set.[15] Solvation energy corrections were calculated using the SMD solvation model[16] in single point energy calculations with *N*,*N*-dimethylacetamide (DMA, ε = 37.78) as the solvent. In the benchmark studies (Tables S1 and S2), DLPNO-CCSD(T)[17–20] single-point energy calculations were performed using the ORCA 5.0 program[19] with the def2-TZVP basis set[15] and the def2-TZVP/C auxiliary basis set, based on the B3LYP-D3(BJ)-optimized geometries. RIJCOSX approximations[22] with the def2/J auxiliary basis set were applied during the HF step. The SMD solvation model with DMA as the solvent was used in the DLPNO-CCSD(T) calculations. Translational entropies in solution were calculated using the free-volume theory proposed by Whitesides[23], implemented in a modified version of the GoodVibes 3.2 package[24], where the molarity (10.8 mol/L) and the molecular volume (133.9 Å^3) of DMA computed at the single-point level of theory using the "volume=tight" keyword, were incorporated into the *thermo.py* script. Gibbs free energies were computed at a temperature of 80 C° and standard concentration (1 mol/L).

Benchmark studies

We tested geometry optimizations with B3LYP-D3(BJ) and ωB97X-D using the 6-31G(d)–SDD(Pd) basis set and the def2-TZVP basis set, where single-point energy calculations were performed at the DLPNO-CCSD(T)/def2-TZVP–SMD(DMA) level of theory (Table S9). The B3LYP-D3(BJ)/6-31G(d)–SDD(Pd) geometry optimization method combined with DLPNO-CCSD(T) single point energy calculations yielded activation enthalpies and free energies in best agreement with experimental values, with $\Delta H^{\ddagger}$ and $\Delta G^{\ddagger}$ deviations of 0.9 and 3.1 kcal/mol, respectively. Other geometry optimization methods showed varied impacts on the activation barriers. Notably, ωB97X-D/6-31G(d)–SDD(Pd) resulted in a significant deviation of 7.9 kcal/mol in $\Delta H^{\ddagger}$ from the experimental value, despite showing good agreement for $T\Delta S^{\ddagger}$ (deviation of 0.1 kcal/mol). B3LYP-D3(BJ)/def2-TZVP and ωB97X-D/def2-TZVP optimizations yielded intermediate results with $\Delta H^{\ddagger}$ deviations of 3.7 and 3.8 kcal/mol from the experimental value, respectively.

Table S9. Overall activation enthalpy ($\Delta H^{\ddagger}$), activation entropy ($\Delta S^{\ddagger}$), and activation Gibbs free energy ($\Delta G^{\ddagger}$) were computed using different levels of theories for geometry optimizations. All values are in kcal/mol and were computed at 80 °C from the catalyst resting state **A''** to the turnover-limiting transition state (**TS2**).

DLPNO-CCSD(T)/def2-TZVP–SMD(DMA)//**optimization**

level of theory in geometry optimization	$\Delta H^{\ddagger}$	$T\Delta S^{\ddagger}$	$\Delta G^{\ddagger}$	$\Delta H^{\ddagger}_{(comp)}-\Delta H^{\ddagger}_{(exp)}$	$T\Delta S^{\ddagger}_{(comp)}-T\Delta S^{\ddagger}_{(exp)}$	$\Delta G^{\ddagger}_{(comp)}-\Delta G^{\ddagger}_{(exp)}$
B3LYP-D3(BJ)/6-31G(d)/SDD(Pd)	17.0	-11.3	28.3	0.9	-2.2	3.1
ωB97X-D/6-31G(d)/SDD(Pd)	24.0	-9.2	33.2	7.9	-0.1	8.0
B3LYP-D3(BJ)/def2-TZVP	19.8	-6.4	26.2	3.7	2.7	1.0
ωB97X-D/def2-TZVP	19.9	-7.2	27.1	3.8	1.9	1.9
experimental value	16.1	-9.1	25.2			

Benchmarking various single-point energy methods with geometries optimized with B3LYP-D3(BJ)/6-31G(d)–SDD(Pd) revealed that Pople basis sets underestimated activation enthalpies, exhibiting deviations of up to −20.2 kcal/mol for B3LYP/6-311+G(d,p)–SDD(Pd) (Table S10). The ωB97X-D/def2-TZVP method showed good agreement with experimental values, with deviations in $\Delta H^{\ddagger}$ and $\Delta G^{\ddagger}$ of 1.4 and 3.5 kcal/mol, respectively. Among the DFT methods, ωB97X-D/def2-TZVP aligned most closely with DLPNO-CCSD(T)-computed values and experimental results. Based on these benchmark results, the ωB97X-D/def2-TZVP/SMD(DMA)//B3LYP-D3(BJ)/6-31G(d)–SDD(Pd) level of theory was chosen to study the reaction energy profiles and the competing pathways reported in the main text.

Table S10. Single point benchmark results. All values are reported in kcal/mol at 80 °C.

single point–SMD(DMA)//B3LYP-D3(BJ)/6-31G(d)/SDD(Pd)

single point level of theory	$\Delta H^{\ddagger}$	$T\Delta S^{\ddagger}$	$\Delta G^{\ddagger}$	$\Delta H^{\ddagger}_{(comp)}-\Delta H^{\ddagger}_{(exp)}$	$T\Delta S^{\ddagger}_{(comp)}-T\Delta S^{\ddagger}_{(exp)}$	$\Delta G^{\ddagger}_{(comp)}-\Delta G^{\ddagger}_{(exp)}$
DLPNO-CCSD(T)/def2-TZVP	17.0	-11.3	28.3	0.9	-2.2	3.1
ωB97X-D/6-311+G(d,p)/SDD(Pd)	7.8	-11.3	19.1	-8.3	-2.2	-6.1
M06/6-311+G(d,p)/SDD(Pd)	7.4	-11.2	18.6	-8.7	-2.1	-6.6
B3LYP-D3(BJ)/6-311+G(d,p)/SDD(Pd)	-4.1	-11.2	7.1	-20.2	-2.1	-18.1
**ωB97X-D/def2-TZVP**	**17.5**	**-11.2**	**28.7**	**1.4**	**-2.1**	**3.5**
M06/def2-TZVP	18.3	-11.2	29.5	2.2	-2.1	4.3
ωB97X-D/def2-TZVPP	18.1	-11.2	29.3	2.0	-2.1	4.1
experimental value	16.1	-9.1	25.2			

In the computational results reported in the main text, all geometry optimizations were performed in gas phase followed by single point energy calculations in DMA using the SMD solvation model. We compared these results with those using geometries optimized in DMA solution for key intermediates and transition states involved in the oxidative addition step (Table S11). The overall barriers (**A''**→**TS2**) are comparable (30.5 vs 28.7 kcal/mol) and the oxidative addition transition state leading to the cis-cyclopropane product (**TS2**) is still more favorable than **TS2'** leading to the trans-product, although the computed selectivity is lower when using geometries optimized in solution. The Gibbs free energies of the Pd(IV) complexes with SO_2F^- (**B** and **B'**) were 5–7 kcal/mol lower in energy when compared to results using gas phase geometries. The S–F bond in the structures optimized in solution is significantly longer than the gas-phase optimized geometries (1.77 vs 1.71 Å, respectively). In addition, the SO_2F^- is further away from the Pd(IV) complex when the geometry was optimized in solution. These differences could lead to the greater stabilities of **B** and **B'** when using geometries optimized in solution.

Table S11. Gibbs free energies of key intermediates and transition states in the oxidative addition step computed using geometries optimized in gas phase and implicit solvent. All values were computed at 80 °C and are with respect to **A''** in kcal/mol.

Compound	Method of geometry optimization[a]	
	B3LYP-D3(BJ)/ 6-31G(d)–SDD(Pd) (gas phase)	B3LYP-D3(BJ)/ 6-31G(d)–SDD(Pd) SMD(DMA)
**A''**	0.0	0.0
**A**	8.5	8.4
**A'**	8.1	7.3
**TS2**	28.7	30.5
**TS2'**	31.7	31.8
**B**	23.7	16.8
**B'**	23.6	18.0

[a]Single point energies were calculated at the ωB97X-D/def2-TZVP/SMD(DMA) level of theory in all calculations.

Additional computational results

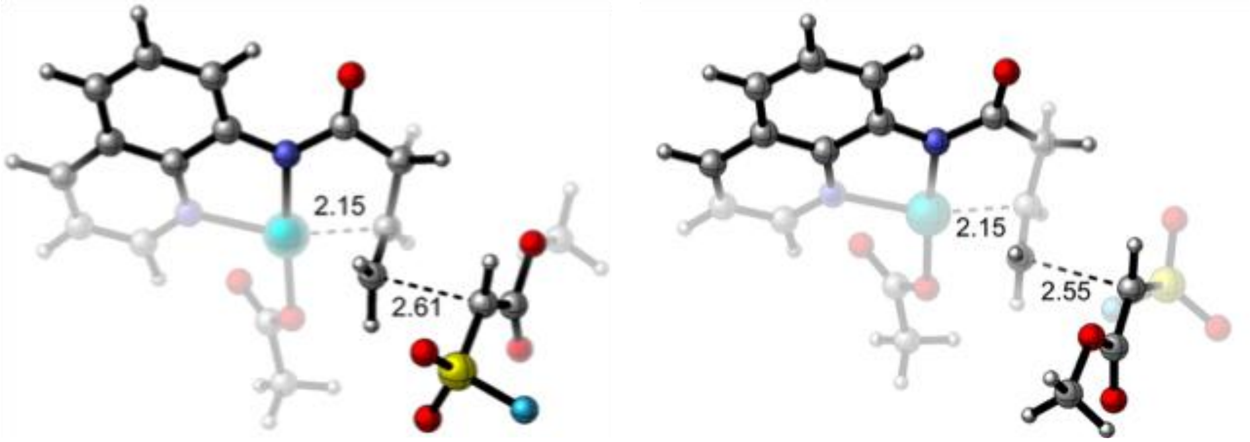

TS1
$\Delta\Delta G^{\ddagger}$ = 0.0 kcal/mol

TS1'
$\Delta\Delta G^{\ddagger}$ = 3.4 kcal/mol

Figure S4. *Anti*-nucleopalladation transition states. Gibbs free energies are reported with respect to **TS1**.

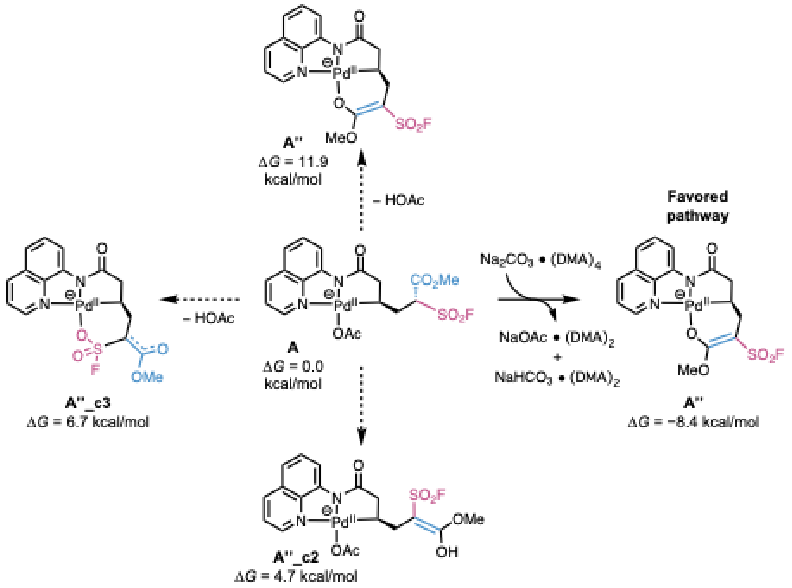

Figure S5. Possible resting states for the Pd complex. Several intermediates, including **A''_c3** (Δ*G* = 6.7 kcal/mol), **A''_c2** (Δ*G* = 4.7 kcal/mol), and **A''** (Δ*G* = 11.9 kcal/mol), are shown, with varying degrees of stability. The favored pathway leads to the most stable resting state **A''** and is calculated using DMA-solvated sodium carbonate as the base (Δ*G* = −8.4 kcal/mol), indicating this as the most likely resting state in the equilibrium. All Gibbs free energies are with respect to **A**.

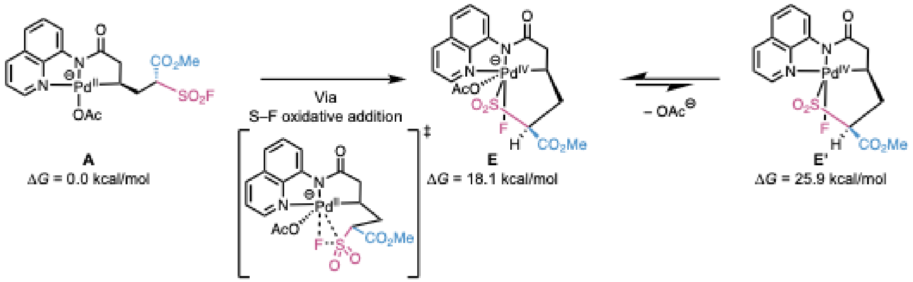

Figure S6. The S–F oxidative addition pathway can be excluded because the 6-coordinated intermediate **E** (Δ*G* = 18.1 kcal/mol) and the 5-coordinated intermediate **E'** (Δ*G* = 25.9 kcal/mol) are much higher in energy than complex **A** (Δ*G* = 0.0 kcal/mol). Although the transition state of S–F oxidative addition could not be calculated, the significantly higher energy intermediates suggest that this pathway is unlikely.

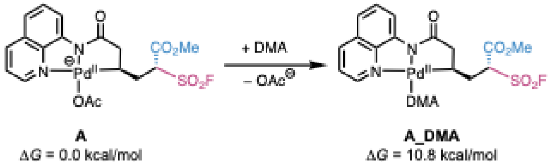

Figure S7. The acetate anionic ligand binding to the Pd center is thermodynamically more favorable than the binding of the neutral ligand DMA by more than 10 kcal/mol.

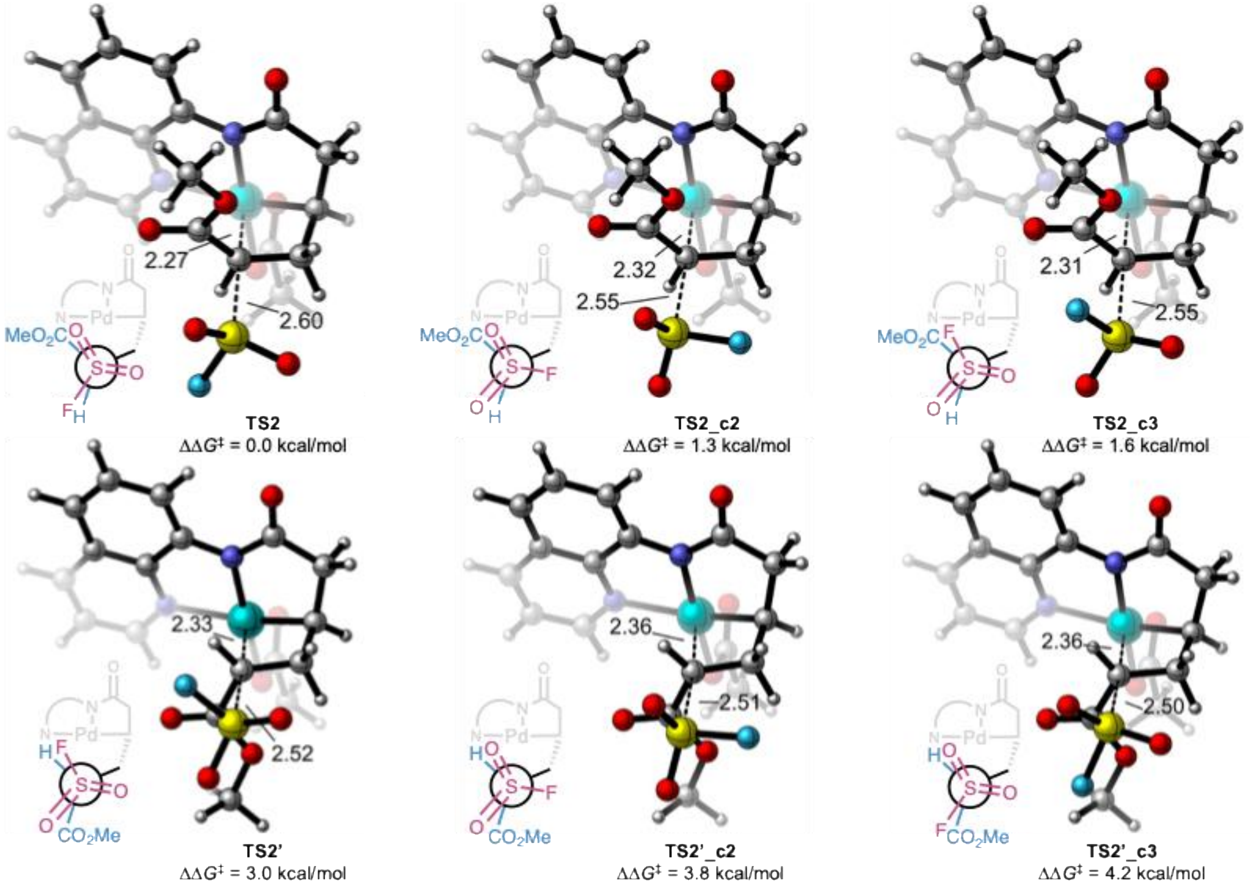

Figure S8. Conformations of the sulfonyl fluoride leaving group in the S_N2-type oxidative addition transition states. The most stable conformer has the fluoride *syn* to the *α*-C–H.

Next, we computed the activation free energies of the SN2-type oxidative addition with a phenyl ester substrate (**2f'**) and a substrate with a sulfonyl chloride leaving group (LG2) (Figure S9). The S_N2-type oxidative addition with a phenyl ester (**2f'**) had an increased barrier ($\Delta G^‡$ = 30.9 kcal/mol) and lower computed diastereoselectivity ($\Delta\Delta G^‡$ = 1.2 kcal/mol) due to steric repulsions between the directing group and the ester stabilizing the transition state leading to the cis-cyclopropane product. The computed OA barrier with LG2 (SO_2Cl^-) is 1.9 kcal/mol higher than that with LG1 (SO_2F^-). Additionally, the experimental stability test showed that the sulfonyl chloride pronucleophile is unstable (Pages S8–S9). These results suggest that the weaker LG ability and low stability contribute to the low yield with LG2. The weaker LG ability of LG2 leads to an earlier TS, evidenced by the longer forming Pd–C bond distance (2.48 Å in **TS2_LG2** with LG2 compared to 2.27 Å in **TS2** with LG1), resulting in a diminished computed cis/trans selectivity in the OA transition states with LG2 (0.2 kcal/mol), which is consistent with the experimentally observed low diastereoselectivity.

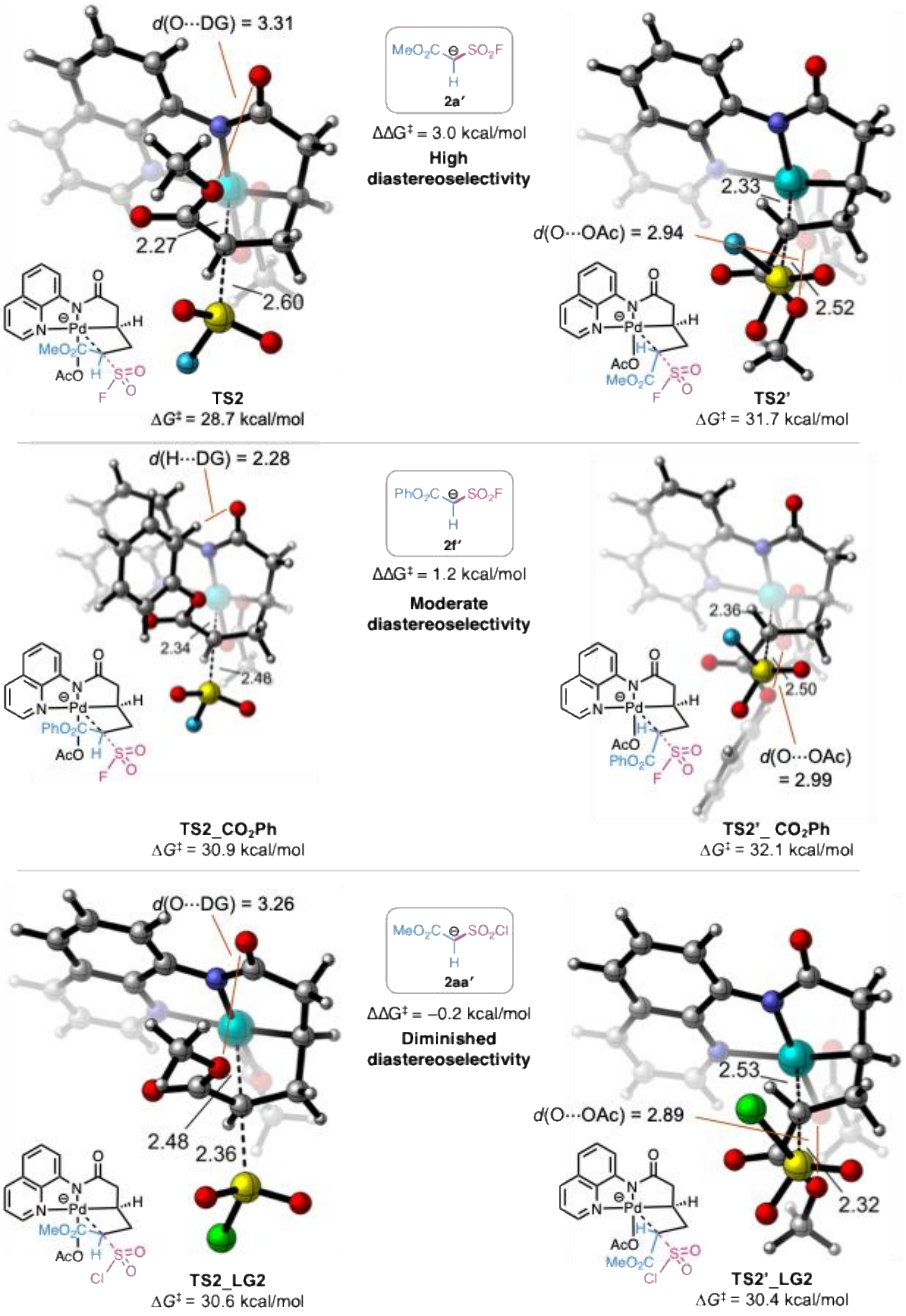

Figure S9. Oxidative addition transition states with a phenyl ester nucleophile **2f'** and sulfonyl chloride **2aa'** show decreased diastereoselectivity. All activation free energies are with respect to the Pd-enolate resting state (**A″**).

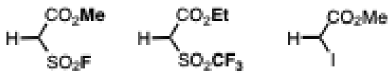

	2a	2a''	2ab
Experimental[1] pK_a (Water)	–	6.83	–
Predicted[2] pK_a	7.75	7.35	15.74

[1] *J. Org. Chem.* **2003**, *68*, 6566
[2] *computed using AIMNet2*

Figure S10. Predicted pK_a values support facile deprotonation of the sulfonyl fluoride nucleophile[25] **2a** and indicate that methyl 2-iodoacetate (**2ab**) is significantly less acidic, which may account for its lack of reactivity under the same conditions. Calculations were performed using Rowan's pK_a prediction workflow[26] that utilizes the atoms-in-molecules neural network potential (AIMNet2).[27]

ΔG‡ (kcal/mol) **A''** = 0.0 kcal/mol	MeO_2C–C⊖(H)–SO_2F **2a′** LG1	MeO_2C–C⊖(H)–SO_2Cl **2aa′** LG2	MeO_2C–C⊖(H)–I **2ab′** LG6
**TS2**	28.7	30.6	4.5
**TS2′**	31.7	30.4	7.8

Figure S11. Overall reaction barriers of the rate-determining oxidative addition step from the catalyst resting state **A′′** to the turnover-limiting transition states (**TS2**/**TS2′**) with LG2 (–SO_2Cl) and LG6 (–I). While the iodide is an excellent leaving group and the oxidative addition barrier with the **2ab′** nucleophile is low, the low reactivity of LG6 is likely attributed to the difficulty in the deprotonation of α-C–H of the 2-iodoacetate nucleophile.

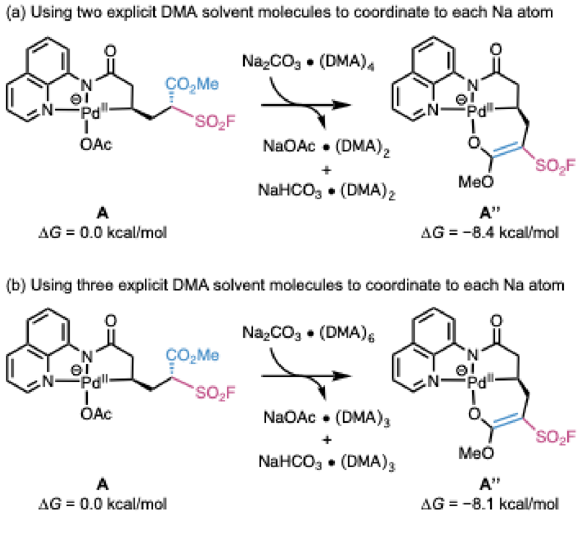

Figure S12. Computed Gibbs free energies of the deprotonation of the α-C–H in palladacycle **A** using either two or three DMA molecules per sodium atom.

Cartesian Coordinates

Pd-1

```
B3LYP-D3(BJ)/6-31G(d)-SDD(Pd) SCF energy in gas phase (au):  -1043.287645
B3LYP-D3(BJ)/6-31G(d)-SDD(Pd) enthalpy in gas phase (au):    -1042.992759
B3LYP-D3(BJ)/6-31G(d)-SDD(Pd) free energy in gas phase (au): -1043.072137
ωB97X-D/def2-TZVP SCF energy in solution (au):               -1043.301137
ωB97X-D/def2-TZVP enthalpy in solution (au):                 -1043.006251
ωB97X-D/def2-TZVP free energy in solution (au):              -1043.085629

Cartesian coordinates
ATOM         X            Y            Z
Pd       0.890700     0.370870    -0.214960
C       -1.716960    -0.820460    -0.030170
C       -0.112310    -2.476780    -0.466940
C       -1.964880     0.575180     0.134430
C       -2.760160    -1.785800     0.040180
C       -1.091570    -3.487090    -0.410850
H        0.932330    -2.679690    -0.672580
C       -3.278960     0.972260     0.372670
C       -4.080210    -1.338120     0.291560
C       -2.401450    -3.142060    -0.155240
H       -0.798500    -4.519020    -0.568770
C       -4.312180     0.011560     0.450540
H       -3.495720     2.023000     0.490870
H       -4.887060    -2.061930     0.352350
H       -3.174770    -3.903860    -0.105420
H       -5.322250     0.362360     0.641900
N       -0.423490    -1.203540    -0.276650
N       -0.831360     1.388030     0.032300
C       -0.890950     2.759190    -0.013500
O       -1.908400     3.438200     0.048020
C        0.493330     3.414690    -0.110010
H        0.401280     4.275380    -0.778260
H        0.725930     3.807270     0.887790
C        1.598910     2.498170    -0.572290
C        2.465550     1.873450     0.293910
H        1.805420     2.470100    -1.640950
H        3.360690     1.382140    -0.067310
H        2.391680     2.022080     1.367730
O        2.808140    -0.934460     1.620420
C        3.088870    -1.285500     0.476570
O        2.471410    -0.868900    -0.606600
C        4.207840    -2.275310     0.182470
H        4.737580    -2.524200     1.103480
H        3.792320    -3.189320    -0.257040
H        4.904330    -1.854510    -0.549720
```

TS1

```
B3LYP-D3(BJ)/6-31G(d)-SDD(Pd) SCF energy in gas phase (au):  -1958.967992
B3LYP-D3(BJ)/6-31G(d)-SDD(Pd) enthalpy in gas phase (au):    -1958.578324
B3LYP-D3(BJ)/6-31G(d)-SDD(Pd) free energy in gas phase (au): -1958.692885
ωB97X-D/def2-TZVP SCF energy in solution (au):               -1959.156736
ωB97X-D/def2-TZVP enthalpy in solution (au):                 -1958.767068
```

ωB97X-D/def2-TZVP free energy in solution (au): -1958.881630
imaginary frequency (cm^{-1}): -46.43

Cartesian coordinates

ATOM	X	Y	Z
Pd	-0.845850	0.556770	0.297630
C	0.996430	-0.149110	1.149510
O	-0.795520	-3.360410	1.559240
C	-0.573090	-2.175800	1.316820
C	0.681810	-1.466740	1.834550
C	1.316700	-0.111650	-0.205960
N	-1.416800	-1.328450	0.635400
C	-2.681600	-1.662580	0.176690
C	-3.268690	-2.928480	0.169210
C	-4.581170	-3.104820	-0.316800
C	-5.338530	-2.054450	-0.792170
C	-4.786100	-0.749650	-0.794650
C	-3.457040	-0.560950	-0.318160
N	-2.880970	0.685680	-0.295160
C	-3.564900	1.746790	-0.694250
C	-4.882800	1.638360	-1.186390
C	-5.482600	0.400880	-1.241070
H	0.519580	-1.298690	2.906780
H	1.535740	-2.147270	1.753520
H	-2.698460	-3.762840	0.550040
H	-5.000310	-4.107940	-0.310860
H	-6.348560	-2.205730	-1.162680
H	-3.064130	2.699710	-0.578210
H	-5.402980	2.534610	-1.507900
H	-6.497450	0.289020	-1.615530
H	1.373550	0.657990	1.772960
H	1.724660	0.781010	-0.664000
C	3.763140	-1.009630	-0.300510
H	1.150790	-0.968280	-0.849330
O	-1.776810	3.475080	1.074640
C	-0.713750	3.475420	0.444470
O	-0.123240	2.435180	-0.065340
C	0.054340	4.768970	0.185020
H	-0.514760	5.627730	0.548370
H	1.023380	4.726590	0.694140
H	0.260680	4.877570	-0.884720
H	3.588380	-2.065260	-0.147480
C	4.163120	-0.184010	0.792800
O	4.607430	0.954890	0.802020
O	3.904040	-0.855130	1.988210
C	4.191200	-0.095920	3.154360
H	3.928440	-0.737400	3.999530
H	5.250890	0.177400	3.203330
H	3.602590	0.827680	3.186120
S	4.021640	-0.592070	-1.913900
O	3.419110	-1.615250	-2.773540
O	3.809360	0.824240	-2.200810
F	5.649060	-0.785920	-2.184500

TS1'

B3LYP-D3(BJ)/6-31G(d)-SDD(Pd) SCF energy in gas phase (au): -1958.965996
B3LYP-D3(BJ)/6-31G(d)-SDD(Pd) enthalpy in gas phase (au): -1958.576417

B3LYP-D3(BJ)/6-31G(d)-SDD(Pd) free energy in gas phase (au): -1958.690705
ωB97X-D/def2-TZVP SCF energy in solution (au): -1959.151629
ωB97X-D/def2-TZVP enthalpy in solution (au): -1958.762050
ωB97X-D/def2-TZVP free energy in solution (au): -1958.876338
imaginary frequency (cm^{-1}): -123.69

Cartesian coordinates

ATOM	X	Y	Z
Pd	0.813620	0.597390	-0.301530
C	-1.034080	-0.064600	-1.172860
O	0.758880	-3.232040	-1.807850
C	0.542110	-2.060690	-1.503220
C	-0.713030	-1.317650	-1.966130
C	-1.340540	-0.145280	0.185740
N	1.397970	-1.251690	-0.788430
C	2.668530	-1.609640	-0.367870
C	3.263580	-2.869170	-0.459130
C	4.583200	-3.071380	-0.004120
C	5.341010	-2.053290	0.536660
C	4.780910	-0.756010	0.641140
C	3.444160	-0.541690	0.197450
N	2.860380	0.699030	0.274280
C	3.544340	1.733110	0.739450
C	4.870130	1.597960	1.203520
C	5.477410	0.363740	1.160290
H	-0.548140	-1.055390	-3.018560
H	-1.565460	-2.005690	-1.953880
H	2.693110	-3.677140	-0.892840
H	5.008350	-4.068440	-0.089250
H	6.357190	-2.223990	0.881120
H	3.035340	2.688260	0.698260
H	5.390480	2.472200	1.580810
H	6.498370	0.231610	1.510960
H	-1.451560	0.773530	-1.722240
H	-1.742650	0.710070	0.711590
C	-3.694510	-1.107280	0.296010
H	-1.137060	-1.039060	0.764150
O	1.701890	3.601660	-0.799230
C	0.634820	3.521600	-0.181040
O	0.067250	2.428290	0.234210
C	-0.169430	4.770280	0.173010
H	0.397120	5.670330	-0.076650
H	-1.112000	4.764370	-0.385550
H	-0.427170	4.768100	1.237030
H	-3.302040	-2.030490	-0.102720
S	-4.494000	-0.164480	-0.862990
O	-4.052450	-0.601120	-2.191910
O	-5.906600	0.118890	-0.624860
F	-3.803690	1.331110	-0.688550
C	-3.851040	-0.893210	1.694450
O	-4.381470	0.043650	2.278430
O	-3.209020	-1.907510	2.399340
C	-3.234180	-1.736560	3.810250
H	-2.685490	-2.586980	4.223390
H	-2.754020	-0.798070	4.108150
H	-4.260510	-1.728660	4.193670

A

B3LYP-D3(BJ)/6-31G(d)-SDD(Pd) SCF energy in gas phase (au): -1959.010210
B3LYP-D3(BJ)/6-31G(d)-SDD(Pd) enthalpy in gas phase (au): -1958.617308
B3LYP-D3(BJ)/6-31G(d)-SDD(Pd) free energy in gas phase (au): -1958.727337
ωB97X-D/def2-TZVP SCF energy in solution (au): -1959.191549
ωB97X-D/def2-TZVP enthalpy in solution (au): -1958.798646
ωB97X-D/def2-TZVP free energy in solution (au): -1958.908675

Cartesian coordinates

ATOM	X	Y	Z
Pd	0.940000	-0.307170	-0.136250
C	-0.687740	0.812590	0.343590
O	1.733720	3.580930	0.916500
C	1.253550	2.501000	0.563590
C	-0.253910	2.291080	0.386110
C	-1.876750	0.555060	-0.580870
N	1.963770	1.371400	0.229560
C	3.337760	1.270870	0.194290
C	4.267690	2.283580	0.457340
C	5.650100	2.031920	0.365130
C	6.158200	0.794200	0.019820
C	5.259910	-0.266410	-0.251430
C	3.855530	-0.028790	-0.164380
N	2.952260	-1.028020	-0.414800
C	3.368130	-2.239600	-0.741980
C	4.738930	-2.558020	-0.851750
C	5.672990	-1.576020	-0.607960
H	-0.766850	2.873930	1.163650
H	-0.501280	2.778320	-0.570580
H	3.893620	3.258620	0.734260
H	6.336010	2.849280	0.577750
H	7.228730	0.617600	-0.043860
H	2.586140	-2.972450	-0.919280
H	5.036330	-3.566280	-1.123700
H	6.737290	-1.788510	-0.682840
H	-0.910450	0.453530	1.353710
H	-1.963260	-0.502570	-0.830100
C	-3.212150	1.000260	0.060690
H	-1.749030	1.102590	-1.521530
O	-0.102110	-2.055910	-0.572420
C	-0.854590	-2.597450	0.329980
O	-0.942510	-2.265530	1.520680
C	-1.752350	-3.713400	-0.212960
H	-1.289200	-4.225270	-1.060890
H	-1.994270	-4.429790	0.577760
H	-2.687340	-3.257630	-0.562820
C	-3.520340	0.195930	1.318350
O	-3.410360	0.640460	2.437140
O	-3.876230	-1.055340	1.003340
C	-3.993630	-1.983230	2.102280
H	-4.490130	-1.503100	2.947710
H	-4.590160	-2.810470	1.715810
H	-2.984390	-2.313540	2.360080
S	-4.596080	0.842950	-1.108240
O	-4.491670	-0.319230	-1.971580
O	-5.854690	1.208260	-0.473540
F	-4.190690	2.122870	-2.045580

H   -3.207890   2.058090   0.331240

A'

B3LYP-D3(BJ)/6-31G(d)-SDD(Pd) SCF energy in gas phase (au): -1959.001119
B3LYP-D3(BJ)/6-31G(d)-SDD(Pd) enthalpy in gas phase (au):   -1958.608366
B3LYP-D3(BJ)/6-31G(d)-SDD(Pd) free energy in gas phase (au): -1958.722645
ωB97X-D/def2-TZVP SCF energy in solution (au):   -1959.187796
ωB97X-D/def2-TZVP enthalpy in solution (au):   -1958.795043
ωB97X-D/def2-TZVP free energy in solution (au):   -1958.909322

Cartesian coordinates

ATOM	X	Y	Z
Pd	-0.920570	0.369700	0.117880
C	0.823750	-0.678220	0.275220
O	-1.203620	-3.706200	-0.350360
C	-0.876960	-2.518470	-0.272600
C	0.582260	-2.065600	-0.339920
C	1.985960	0.073320	-0.353900
N	-1.727940	-1.444580	-0.177170
C	-3.106370	-1.522600	-0.187730
C	-3.881870	-2.683430	-0.276290
C	-5.288620	-2.609620	-0.289540
C	-5.963990	-1.407890	-0.221290
C	-5.222200	-0.203630	-0.134970
C	-3.796890	-0.259300	-0.112030
N	-3.039410	0.881860	-0.032890
C	-3.628010	2.068200	0.002980
C	-5.034170	2.206070	-0.010290
C	-5.821400	1.080060	-0.073360
H	1.205870	-2.851360	0.107180
H	0.831670	-2.041170	-1.413920
H	-3.371660	-3.633720	-0.334700
H	-5.852320	-3.537880	-0.356800
H	-7.050170	-1.364810	-0.233800
H	-2.951230	2.917600	0.005470
H	-5.470310	3.199950	0.024230
H	-6.907030	1.154160	-0.085660
H	0.979830	-0.774350	1.355820
H	2.094520	1.060820	0.095980
C	3.362010	-0.646370	-0.323440
H	1.784530	0.228280	-1.422840
O	-1.098260	3.637940	-0.578920
C	-0.154700	3.298340	0.148650
O	0.105340	2.117740	0.600880
C	0.876070	4.340810	0.600450
H	0.583220	5.340700	0.270140
H	0.977770	4.322560	1.691060
H	1.858690	4.091280	0.182920
H	3.286990	-1.654240	-0.740770
S	4.003830	-0.963830	1.345550
O	3.202040	-1.966260	2.023860
O	5.455700	-1.071070	1.311440
F	3.671040	0.450750	2.054450
C	4.399240	0.156460	-1.086260
O	4.747140	1.282230	-0.815320
O	4.840370	-0.536850	-2.157350
C	5.778850	0.168810	-2.981920

H	6.020000	-0.511700	-3.799020
H	5.333680	1.091120	-3.364770
H	6.675190	0.418340	-2.407890

A''

B3LYP-D3(BJ)/6-31G(d)-SDD(Pd) SCF energy in gas phase (au): -1729.885930
B3LYP-D3(BJ)/6-31G(d)-SDD(Pd) enthalpy in gas phase (au): -1729.565128
B3LYP-D3(BJ)/6-31G(d)-SDD(Pd) free energy in gas phase (au): -1729.656811
ωB97X-D/def2-TZVP SCF energy in solution (au): -1730.048047
ωB97X-D/def2-TZVP enthalpy in solution (au): -1729.727245
ωB97X-D/def2-TZVP free energy in solution (au): -1729.818928

Cartesian coordinates

ATOM	X	Y	Z
Pd	-0.362980	-0.002250	-0.135700
C	0.916520	-1.546960	0.072890
O	-2.086430	-3.697400	0.217090
C	-1.366560	-2.713340	0.035720
C	0.125560	-2.814230	-0.290790
C	2.182850	-1.342040	-0.757010
N	-1.781860	-1.398010	0.035860
C	-3.083510	-0.963480	0.131290
C	-4.223550	-1.754830	0.317310
C	-5.502510	-1.170810	0.382790
C	-5.701860	0.192030	0.272100
C	-4.582250	1.037430	0.081500
C	-3.276090	0.464030	0.009010
N	-2.168340	1.247320	-0.182110
C	-2.293800	2.558680	-0.295890
C	-3.545680	3.207310	-0.230030
C	-4.678260	2.447070	-0.043810
H	0.529820	-3.723640	0.174180
H	0.182590	-2.973010	-1.379390
H	-4.090470	-2.823250	0.408650
H	-6.359230	-1.825000	0.530120
H	-6.696110	0.627080	0.329950
H	-1.370460	3.111870	-0.444600
H	-3.598440	4.287390	-0.328260
H	-5.659790	2.913050	0.009740
H	1.176960	-1.559270	1.141750
H	1.900530	-1.267720	-1.819990
C	2.992190	-0.128530	-0.344600
H	2.830730	-2.223170	-0.677130
C	2.401420	1.121420	-0.158580
O	1.166880	1.404200	-0.311070
O	3.219190	2.143280	0.214930
C	2.593330	3.387250	0.492640
H	2.109110	3.800880	-0.398590
H	1.843160	3.290920	1.284820
H	3.400680	4.047370	0.817730
S	4.658520	-0.344470	-0.013230
O	5.040240	-1.742580	-0.223120
O	5.538420	0.710200	-0.511680
F	4.759970	-0.160110	1.632450

TS2

B3LYP-D3(BJ)/6-31G(d)-SDD(Pd) SCF energy in gas phase (au): -1958.987892

B3LYP-D3(BJ)/6-31G(d)-SDD(Pd) enthalpy in gas phase (au): -1958.597858
B3LYP-D3(BJ)/6-31G(d)-SDD(Pd) free energy in gas phase (au): -1958.709518
ωB97X-D/def2-TZVP SCF energy in solution (au): -1959.154751
ωB97X-D/def2-TZVP enthalpy in solution (au): -1958.764717
ωB97X-D/def2-TZVP free energy in solution (au): -1958.876377
imaginary frequency (cm^{-1}): -54.70

Cartesian coordinates

ATOM	X	Y	Z
Pd	-0.311220	1.132340	0.564280
C	1.095320	0.991310	2.028330
C	1.563430	0.064690	-0.133920
C	-2.478410	-0.523890	-0.547410
C	-2.178830	1.262470	-2.018430
C	-2.078640	-1.159320	0.681280
C	-3.463910	-1.113330	-1.390910
C	-3.143240	0.748320	-2.910490
H	-1.639740	2.182710	-2.223550
C	-2.697000	-2.363280	1.021100
C	-4.065260	-2.336040	-1.002870
C	-3.779080	-0.430550	-2.592300
H	-3.364180	1.281640	-3.829410
C	-3.676980	-2.930250	0.179340
H	-2.400050	-2.850680	1.938210
H	-4.815220	-2.789090	-1.645410
H	-4.524260	-0.858160	-3.259000
H	-4.131250	-3.871030	0.480990
N	-1.870250	0.652000	-0.890200
N	-1.102240	-0.500380	1.409330
C	-0.500730	-0.966540	2.556640
O	-0.782460	-2.010390	3.142250
C	0.544310	0.023160	3.081330
H	0.042140	0.594880	3.874040
H	1.343260	-0.555240	3.563210
C	2.243980	0.450280	1.161710
H	1.295090	1.987270	2.427890
H	2.988590	1.220650	0.953100
H	2.758140	-0.402480	1.614360
C	-0.242090	3.851500	0.157840
O	0.481550	2.860820	-0.268190
C	0.208670	5.217530	-0.344510
H	1.196300	5.450730	0.069270
H	0.310820	5.204840	-1.434720
H	-0.503440	5.988980	-0.043440
S	3.770400	-0.928670	-1.078300
O	3.799050	-2.332520	-1.556250
O	4.962150	-0.471490	-0.322350
F	3.862720	-0.022490	-2.518910
C	1.028190	-1.294400	-0.414890
O	0.412190	-1.596280	-1.418700
O	1.309790	-2.153050	0.585550
C	0.901070	-3.507810	0.363750
H	1.522890	-3.944290	-0.422330
H	1.055470	-4.014080	1.316550
H	-0.152020	-3.545760	0.077900
H	1.630680	0.713500	-0.997130
O	-1.215410	3.729740	0.914920

TS2'
B3LYP-D3(BJ)/6-31G(d)-SDD(Pd) SCF energy in gas phase (au): -1958.981882
B3LYP-D3(BJ)/6-31G(d)-SDD(Pd) enthalpy in gas phase (au): -1958.591895
B3LYP-D3(BJ)/6-31G(d)-SDD(Pd) free energy in gas phase (au): -1958.705571
ωB97X-D/def2-TZVP SCF energy in solution (au): -1959.147985
ωB97X-D/def2-TZVP enthalpy in solution (au): -1958.757998
ωB97X-D/def2-TZVP free energy in solution (au): -1958.871674
imaginary frequency (cm^{-1}): -150.45

Cartesian coordinates

ATOM	X	Y	Z
Pd	-0.370180	0.850280	0.740530
C	0.905950	0.465050	2.277920
C	1.446040	-0.467810	0.111930
C	-2.511990	-0.589270	-0.678510
C	-2.011510	1.303220	-1.949510
C	-2.235570	-1.357160	0.508360
C	-3.466050	-1.039330	-1.637250
C	-2.930450	0.926440	-2.951830
H	-1.407690	2.201510	-2.031190
C	-2.946680	-2.542850	0.696690
C	-4.163450	-2.249940	-1.402100
C	-3.652720	-0.234100	-2.789280
H	-3.051070	1.549980	-3.831810
C	-3.896960	-2.968080	-0.254850
H	-2.747740	-3.124110	1.585470
H	-4.889760	-2.597250	-2.131860
H	-4.369160	-0.553820	-3.542470
H	-4.425750	-3.900310	-0.071400
N	-1.823870	0.579210	-0.863570
N	-1.274370	-0.828770	1.354290
C	-0.886840	-1.345500	2.568860
O	-1.325960	-2.368670	3.091450
C	0.140360	-0.434230	3.251970
H	-0.428350	0.187040	3.957610
H	0.813530	-1.063370	3.850970
C	2.024140	-0.243220	1.491520
H	1.236640	1.402920	2.728170
H	2.915390	0.380730	1.410600
H	2.322000	-1.198190	1.939100
C	-0.252070	3.600300	0.655000
O	0.490320	2.654080	0.168530
C	0.232860	5.005970	0.315070
H	1.220650	5.171550	0.759700
H	0.344710	5.112470	-0.769500
H	-0.468030	5.754130	0.691810
S	3.340260	-1.954740	-0.625230
O	4.150600	-2.480550	0.498290
O	4.074200	-1.609720	-1.864750
F	2.398630	-3.291640	-1.082060
O	-1.267560	3.417780	1.340630
C	1.768820	0.357100	-1.084820
O	1.181550	0.280180	-2.147680
O	2.816370	1.170620	-0.862030
C	3.235340	1.927030	-1.998620
H	4.083850	2.521030	-1.656210

H	3.537620	1.249500	-2.801160
H	2.424570	2.576300	-2.339170
H	0.821180	-1.326940	-0.084570

B

B3LYP-D3(BJ)/6-31G(d)-SDD(Pd) SCF energy in gas phase (au): -1958.988007
B3LYP-D3(BJ)/6-31G(d)-SDD(Pd) enthalpy in gas phase (au): -1958.596813
B3LYP-D3(BJ)/6-31G(d)-SDD(Pd) free energy in gas phase (au): -1958.711512
ωB97X-D/def2-TZVP SCF energy in solution (au): -1959.160915
ωB97X-D/def2-TZVP enthalpy in solution (au): -1958.769721
ωB97X-D/def2-TZVP free energy in solution (au): -1958.884420

Cartesian coordinates

ATOM	X	Y	Z
Pd	-0.447600	1.175910	0.470620
C	0.957270	1.309960	1.941450
C	1.438450	0.275140	-0.140310
C	-2.367850	-0.829200	-0.523140
C	-2.278890	0.857380	-2.132800
C	-1.912630	-1.305760	0.756350
C	-3.242870	-1.614320	-1.328000
C	-3.142610	0.146140	-2.991700
H	-1.864370	1.822910	-2.408030
C	-2.365010	-2.553670	1.184570
C	-3.678710	-2.874620	-0.849840
C	-3.619270	-1.079800	-2.585270
H	-3.410670	0.567990	-3.954870
C	-3.238550	-3.315570	0.380010
H	-2.024380	-2.922290	2.140960
H	-4.343680	-3.476580	-1.462950
H	-4.281860	-1.658550	-3.224440
H	-3.564240	-4.284470	0.750610
N	-1.918070	0.388090	-0.953710
N	-1.049390	-0.465420	1.441430
C	-0.424670	-0.749720	2.635990
O	-0.603540	-1.756890	3.315770
C	0.487230	0.399490	3.078250
H	-0.096280	0.985620	3.801420
H	1.334020	-0.033880	3.625870
C	2.135110	0.784150	1.106000
H	1.058760	2.355470	2.236940
H	2.832480	1.578550	0.834590
H	2.700980	-0.004850	1.607470
C	-0.767200	3.806630	-0.038130
O	0.106470	2.949060	-0.466320
C	-0.591230	5.221830	-0.565750
H	0.347650	5.638580	-0.184170
H	-0.519430	5.210640	-1.658280
H	-1.424740	5.853140	-0.250800
S	3.882030	-0.692420	-0.993560
O	4.045270	-2.135050	-1.318590
O	4.985670	-0.089570	-0.199790
F	4.067190	0.064250	-2.520560
C	1.122960	-1.163100	-0.367180
O	0.571390	-1.589440	-1.364190
O	1.491140	-1.932230	0.676480
C	1.341620	-3.343620	0.477790

H	2.049340	-3.667550	-0.289140
H	1.573460	-3.794530	1.443010
H	0.317220	-3.579550	0.180910
H	1.520310	0.845100	-1.058340
O	-1.681280	3.514890	0.751500

B'

B3LYP-D3(BJ)/6-31G(d)-SDD(Pd) SCF energy in gas phase (au): -1958.983177
B3LYP-D3(BJ)/6-31G(d)-SDD(Pd) enthalpy in gas phase (au): -1958.591977
B3LYP-D3(BJ)/6-31G(d)-SDD(Pd) free energy in gas phase (au): -1958.707017
ωB97X-D/def2-TZVP SCF energy in solution (au): -1959.160681
ωB97X-D/def2-TZVP enthalpy in solution (au): -1958.769481
ωB97X-D/def2-TZVP free energy in solution (au): -1958.884521

Cartesian coordinates

ATOM	X	Y	Z
Pd	-0.495280	0.934330	0.618140
C	0.717900	0.841980	2.256690
C	1.251570	-0.226000	0.210370
C	-2.411860	-0.865500	-0.721290
C	-1.945820	0.851570	-2.231960
C	-2.177150	-1.425900	0.583470
C	-3.244930	-1.533470	-1.665500
C	-2.745190	0.252120	-3.228040
H	-1.402620	1.774150	-2.410580
C	-2.809110	-2.627860	0.900270
C	-3.867140	-2.751900	-1.298010
C	-3.391720	-0.928170	-2.939130
H	-2.832690	0.722660	-4.201810
C	-3.643770	-3.268760	-0.039310
H	-2.640310	-3.054870	1.878120
H	-4.501410	-3.264210	-2.016120
H	-4.014680	-1.417910	-3.683830
H	-4.113460	-4.206460	0.246860
N	-1.801440	0.317930	-1.035090
N	-1.328720	-0.702810	1.409580
C	-0.982330	-1.019160	2.703890
O	-1.396590	-1.984410	3.340830
C	-0.049070	0.038790	3.304900
H	-0.686420	0.708860	3.897790
H	0.630700	-0.463540	4.006220
C	1.869790	0.100280	1.557820
H	0.967840	1.855700	2.573130
H	2.744230	0.737450	1.426080
H	2.190010	-0.807390	2.079520
C	-0.704370	3.560640	0.333300
O	0.217320	2.762540	-0.098880
C	-0.521380	5.026790	-0.015770
H	0.391460	5.403940	0.458380
H	-0.395380	5.140070	-1.097650
H	-1.379310	5.610690	0.323710
S	3.624100	-1.716960	-0.327260
O	4.347950	-1.966840	0.949800
O	4.496920	-1.369690	-1.485110
F	3.063100	-3.286440	-0.743510
O	-1.681340	3.163860	0.998960
C	1.736380	0.364720	-1.074610

O	1.258250	0.094590	-2.161780
O	2.739370	1.242070	-0.894080
C	3.389220	1.670690	-2.096640
H	4.124850	2.411020	-1.778700
H	3.881290	0.809010	-2.553760
H	2.666680	2.115830	-2.785680
H	0.828620	-1.213120	0.069580

TS3

B3LYP-D3(BJ)/6-31G(d)-SDD(Pd) SCF energy in gas phase (au): -1958.986683
B3LYP-D3(BJ)/6-31G(d)-SDD(Pd) enthalpy in gas phase (au): -1958.596608
B3LYP-D3(BJ)/6-31G(d)-SDD(Pd) free energy in gas phase (au): -1958.708656
ωB97X-D/def2-TZVP SCF energy in solution (au): -1959.159690
ωB97X-D/def2-TZVP enthalpy in solution (au): -1958.769616
ωB97X-D/def2-TZVP free energy in solution (au): -1958.881663
imaginary frequency (cm^{-1}): -123.24

Cartesian coordinates

ATOM	X	Y	Z
Pd	-0.308380	0.336720	0.901630
C	0.651240	-1.131570	2.205440
C	1.601680	-0.749490	0.454970
C	-2.857070	0.619940	-0.458560
C	-1.761520	2.691260	-0.478920
C	-2.816890	-0.744090	-0.016190
C	-3.929890	1.121910	-1.248840
C	-2.778660	3.258970	-1.275190
H	-0.882330	3.239070	-0.152800
C	-3.889430	-1.565160	-0.367700
C	-4.991670	0.248210	-1.588620
C	-3.852470	2.480240	-1.648780
H	-2.700200	4.297030	-1.581090
C	-4.953020	-1.058330	-1.144330
H	-3.888280	-2.590480	-0.028820
H	-5.814540	0.616410	-2.194910
H	-4.651680	2.894160	-2.259160
H	-5.764990	-1.733830	-1.402290
N	-1.817460	1.429490	-0.090540
N	-1.695240	-1.097150	0.721760
C	-1.594550	-2.241020	1.475690
O	-2.457050	-3.112450	1.568690
C	-0.260520	-2.351620	2.207560
H	-0.481130	-2.629390	3.246680
H	0.255650	-3.201910	1.751810
C	2.063420	-1.361930	1.722020
H	0.579500	-0.426230	3.032350
H	2.808840	-0.755830	2.229790
H	2.356890	-2.407570	1.630920
H	2.044540	0.186260	0.135830
C	1.196120	-1.661420	-0.660400
O	1.091890	-2.870370	-0.572060
O	0.940280	-0.942410	-1.766540
C	0.899980	-1.648380	-3.012080
H	0.552940	-2.672360	-2.859830
H	0.209370	-1.095720	-3.652610
H	1.910430	-1.616780	-3.427560
C	1.771600	2.195760	1.899140

O	0.834980	2.020200	1.001920
C	2.762330	3.276820	1.494610
H	3.371540	2.857950	0.685010
H	2.244650	4.156820	1.099560
H	3.394900	3.554490	2.341080
O	1.906290	1.543380	2.935300
S	4.337180	-0.115530	-1.706340
O	3.877650	-1.234020	-0.819230
O	3.742970	-0.169700	-3.071480
F	3.419240	1.244790	-0.973060

C

B3LYP-D3(BJ)/6-31G(d)-SDD(Pd) SCF energy in gas phase (au): -2187.496029
B3LYP-D3(BJ)/6-31G(d)-SDD(Pd) enthalpy in gas phase (au): -2187.046222
B3LYP-D3(BJ)/6-31G(d)-SDD(Pd) free energy in gas phase (au): -2187.183243
ωB97X-D/def2-TZVP SCF energy in solution (au): -2187.850766
ωB97X-D/def2-TZVP enthalpy in solution (au): -2187.400959
ωB97X-D/def2-TZVP free energy in solution (au): -2187.537980

Cartesian coordinates

ATOM	X	Y	Z
Pd	-1.952760	-0.061590	0.084120
C	0.376420	-2.214550	-0.294990
O	0.185090	-0.293860	-3.464240
C	-0.085590	-0.582210	-2.299660
C	0.193740	-1.998590	-1.790210
C	1.644290	-2.770500	0.285750
N	-0.592610	0.332450	-1.386220
C	-0.190520	1.643400	-1.397760
C	0.601910	2.300900	-2.352710
C	1.033530	3.620380	-2.136620
C	0.709920	4.344490	-1.001040
C	-0.087050	3.729770	-0.008050
C	-0.539800	2.394730	-0.223240
N	-1.308450	1.751810	0.707150
C	-1.654120	2.349330	1.839080
C	-1.243010	3.665940	2.124280
C	-0.471210	4.347830	1.208420
H	-0.633120	-2.636820	-2.124130
H	1.087530	-2.298560	-2.341830
H	0.888450	1.760670	-3.242350
H	1.654810	4.083780	-2.900800
H	1.060180	5.362970	-0.855100
H	-2.282530	1.764860	2.501070
H	-1.539970	4.122690	3.063730
H	-0.140500	5.365170	1.407770
H	-0.533810	-2.497780	0.222730
H	2.523870	-2.877570	-0.342380
C	1.264580	-1.335190	0.577960
H	1.579800	-3.469980	1.115060
H	0.881110	-1.091940	1.561530
C	2.114520	-0.296430	-0.044790
O	2.603670	-0.357690	-1.157800
O	2.270700	0.771100	0.771730
C	3.289760	1.688630	0.346460
H	3.038680	2.113130	-0.629200
H	3.300850	2.474290	1.106380

H	4.252120	1.169100	0.305210
C	-3.083790	-1.157400	2.495000
O	-3.313720	-0.220120	1.622180
C	-4.255800	-1.343250	3.469710
H	-4.600090	-0.375460	3.851180
H	-5.097960	-1.800230	2.936170
H	-3.961090	-1.990920	4.299840
O	-2.078800	-1.861070	2.587900
C	-3.659210	-1.725730	-1.526930
O	-2.639900	-1.806540	-0.726470
C	-4.053580	-3.101960	-2.082110
H	-3.247510	-3.482660	-2.720570
H	-4.185270	-3.817870	-1.263540
H	-4.974380	-3.026890	-2.667130
O	-4.276140	-0.714110	-1.862890
S	5.670510	-1.455210	1.057810
O	6.201750	-0.267560	0.312320
O	4.462800	-1.135710	1.876510
F	4.953480	-2.399600	-0.236610

2a'

B3LYP-D3(BJ)/6-31G(d) SCF energy in gas phase (au): -915.652514
B3LYP-D3(BJ)/6-31G(d) enthalpy in gas phase (au): -915.558714
B3LYP-D3(BJ)/6-31G(d) free energy in gas phase (au): -915.605925
ωB97X-D/def2-TZVP SCF energy in solution (au): -915.859205
ωB97X-D/def2-TZVP enthalpy in solution (au): -915.765404
ωB97X-D/def2-TZVP free energy in solution (au): -915.812615

Cartesian coordinates

ATOM	X	Y	Z
C	-0.078640	0.726020	0.143430
H	-0.110530	1.793990	-0.014390
S	1.463970	0.081280	0.145880
O	2.450830	1.152340	0.344870
O	1.612460	-1.184230	0.865360
F	1.784380	-0.388410	-1.432940
C	-1.257270	-0.056330	0.062840
O	-1.420200	-1.272470	0.060700
O	-2.376630	0.795580	-0.011750
C	-3.609790	0.106690	-0.075770
H	-4.385170	0.878330	-0.132050
H	-3.670000	-0.544590	-0.956750
H	-3.774830	-0.520630	0.809180

$Na_2CO_3\cdot(DMA)_4$

B3LYP-D3(BJ)/6-31G(d) SCF energy in gas phase (au): -1740.056843
B3LYP-D3(BJ)/6-31G(d) enthalpy in gas phase (au): -1739.449139
B3LYP-D3(BJ)/6-31G(d) free energy in gas phase (au): -1739.596388
ωB97X-D/def2-TZVP SCF energy in solution (au): -1740.085197
ωB97X-D/def2-TZVP enthalpy in solution (au): -1739.477493
ωB97X-D/def2-TZVP free energy in solution (au): -1739.624742

Cartesian coordinates

ATOM	X	Y	Z
C	0.792330	0.825580	-0.090890
O	0.763360	1.008080	-1.369790
O	1.475070	1.577020	0.702740

O	0.109040	-0.185230	0.415080
Na	0.465120	-1.264670	-1.525430
Na	0.287930	0.741550	2.477820
C	3.373480	-0.832370	-1.130950
O	2.688750	-1.808030	-1.515100
N	3.681020	0.200530	-1.947380
C	3.864250	-0.760630	0.297750
H	3.744180	-1.746100	0.747040
H	4.912050	-0.454580	0.370020
H	3.250780	-0.025540	0.835440
C	3.095950	0.237550	-3.283600
H	3.782250	0.757300	-3.959110
H	2.946440	-0.783610	-3.634230
H	2.135340	0.763530	-3.243010
C	4.089640	1.497760	-1.395920
H	3.266190	1.938980	-0.822080
H	4.970420	1.389070	-0.758210
H	4.356960	2.151190	-2.229200
C	-2.461800	-0.895850	-2.152660
O	-1.627650	-1.822900	-2.176200
N	-3.685750	-1.084680	-1.586340
C	-2.074680	0.434280	-2.758290
H	-1.159950	0.777210	-2.247910
H	-1.813970	0.256170	-3.807130
H	-2.824620	1.222240	-2.714810
C	-4.006050	-2.375250	-0.987030
H	-4.864060	-2.825250	-1.501710
H	-3.141290	-3.027780	-1.078150
H	-4.261910	-2.244820	0.070820
C	-4.750650	-0.099850	-1.476220
H	-5.688540	-0.533690	-1.843510
H	-4.896090	0.193970	-0.428810
H	-4.536530	0.790350	-2.060960
C	-0.489290	-2.063820	2.523240
O	-0.314420	-1.251480	3.450390
N	0.503840	-2.909670	2.147490
C	-1.818180	-2.130170	1.802530
H	-2.560630	-1.642900	2.437290
H	-1.724710	-1.565390	0.869670
H	-2.140240	-3.152100	1.583500
C	1.776970	-2.803940	2.848870
H	2.361870	-1.954490	2.476530
H	1.596750	-2.662420	3.914590
H	2.344760	-3.724820	2.688750
C	0.547320	-3.600210	0.865080
H	1.354090	-3.186370	0.245960
H	0.736540	-4.670970	1.010930
H	-0.394180	-3.482590	0.330260
C	-0.742140	3.349410	1.640310
O	-0.946490	2.654860	2.655400
N	-1.474280	3.172790	0.510020
C	0.356140	4.388310	1.630330
H	0.687070	4.534910	2.659540
H	1.185030	3.980410	1.040910
H	0.042870	5.347760	1.208190
C	-2.424530	2.063510	0.482780
H	-1.919890	1.122040	0.231930

H	-2.883390	1.965360	1.466910
H	-3.195160	2.283390	-0.261980
C	-1.041300	3.680890	-0.792330
H	-0.598200	4.671460	-0.688840
H	-0.314120	2.998130	-1.249800
H	-1.919610	3.771780	-1.439690

$NaHCO_3 \cdot (DMA)_2$

B3LYP-D3(BJ)/6-31G(d) SCF energy in gas phase (au): -1002.531604
B3LYP-D3(BJ)/6-31G(d) enthalpy in gas phase (au): -1002.205081
B3LYP-D3(BJ)/6-31G(d) free energy in gas phase (au): -1002.296237
ωB97X-D/def2-TZVP SCF energy in solution (au): -1002.579017
ωB97X-D/def2-TZVP enthalpy in solution (au): -1002.252494
ωB97X-D/def2-TZVP free energy in solution (au): -1002.343650

Cartesian coordinates

ATOM	X	Y	Z
C	2.345260	-1.536010	-0.364050
C	-2.872790	-0.554820	0.622220
O	2.148990	-1.868020	0.842840
O	3.664340	-1.420380	-0.764080
O	1.478870	-1.269800	-1.230570
Na	-0.046360	-1.445810	0.485640
O	-2.264450	-1.630020	0.773520
C	0.674280	1.421780	-0.136080
O	-0.316760	0.918790	0.433430
H	4.178100	-1.693260	0.014950
N	-3.146180	-0.068880	-0.616260
C	-2.591020	-0.781400	-1.764560
H	-1.542520	-0.504860	-1.936150
H	-2.643960	-1.854370	-1.582030
H	-3.176740	-0.532740	-2.653310
C	-3.505570	1.326160	-0.836250
H	-4.197460	1.676820	-0.070080
H	-2.612820	1.964240	-0.824960
H	-4.000840	1.417640	-1.806540
C	-3.309260	0.250410	1.827070
H	-3.174050	-0.372690	2.711760
H	-2.670290	1.136170	1.915210
H	-4.352090	0.576530	1.769120
N	1.827610	1.658390	0.535380
C	0.618270	1.752410	-1.611740
H	1.179540	0.981470	-2.151440
H	-0.424630	1.709810	-1.927640
H	1.026210	2.737960	-1.853020
C	1.929050	1.231760	1.931090
H	2.622490	1.897560	2.453400
H	0.943940	1.294440	2.392220
H	2.288150	0.197080	1.989440
C	3.098670	1.934250	-0.129550
H	2.940890	2.459940	-1.069940
H	3.707430	2.570560	0.520260
H	3.639320	1.003110	-0.337080

$NaOAc \cdot (DMA)_2$

B3LYP-D3(BJ)/6-31G(d) SCF energy in gas phase (au): -966.616388

B3LYP-D3(BJ)/6-31G(d) enthalpy in gas phase (au): -966.266387
B3LYP-D3(BJ)/6-31G(d) free energy in gas phase (au): -966.360297
ωB97X-D/def2-TZVP SCF energy in solution (au): -966.640430
ωB97X-D/def2-TZVP enthalpy in solution (au): -966.290428
ωB97X-D/def2-TZVP free energy in solution (au): -966.384338

Cartesian coordinates

ATOM	X	Y	Z
C	0.000160	1.160620	-0.341570
C	2.769220	-0.979280	-0.161760
O	0.000320	1.044440	0.921190
O	-0.000030	0.186060	-1.153680
Na	-0.000490	-1.258080	0.686960
O	2.188720	-1.810900	0.563530
C	-2.769570	-0.979210	-0.161050
O	-2.189620	-1.810470	0.565110
N	3.276470	0.166110	0.358420
C	3.084590	0.442230	1.781170
H	3.926200	1.043340	2.139610
H	2.143170	0.983270	1.932490
H	3.049850	-0.501490	2.324090
C	3.699440	1.309340	-0.434900
H	2.985380	2.133350	-0.312210
H	4.686640	1.654050	-0.104390
H	3.751970	1.057900	-1.491940
C	2.884270	-1.225870	-1.651210
H	2.680520	-2.283390	-1.824900
H	2.108960	-0.635580	-2.153510
H	3.865070	-0.974100	-2.063960
N	-3.276650	0.166760	0.358050
C	-2.884330	-1.226890	-1.650350
H	-2.109590	-0.636350	-2.153210
H	-2.679800	-2.284380	-1.823330
H	-3.865390	-0.976160	-2.063130
C	-3.084750	0.443970	1.780610
H	-3.051900	-0.499250	2.324540
H	-2.142470	0.983550	1.931730
H	-3.925440	1.046920	2.138110
C	-3.698560	1.309590	-0.436440
H	-3.751950	1.056900	-1.493140
H	-4.685190	1.655830	-0.105860
H	-2.983460	2.132910	-0.315140
C	0.000560	2.569770	-0.940650
H	0.880550	2.698860	-1.581160
H	-0.875830	2.697420	-1.586300
H	-0.002100	3.336540	-0.162510

OAc^-

B3LYP-D3(BJ)/6-31G(d) SCF energy in gas phase (au): -228.499351
B3LYP-D3(BJ)/6-31G(d) enthalpy in gas phase (au): -228.444243
B3LYP-D3(BJ)/6-31G(d) free energy in gas phase (au): -228.474181
ωB97X-D/def2-TZVP SCF energy in solution (au): -228.632944
ωB97X-D/def2-TZVP enthalpy in solution (au): -228.577835
ωB97X-D/def2-TZVP free energy in solution (au): -228.607774

Cartesian coordinates

ATOM	X	Y	Z

C	0.220410	0.001790	-0.000130
O	0.807950	-1.108820	-0.000140
O	0.695580	1.166340	0.000230
C	-1.353440	-0.055050	0.000000
H	-1.748960	0.472220	0.880630
H	-1.749120	0.472310	-0.880510
H	-1.731990	-1.085130	-0.000020

TS4

B3LYP-D3(BJ)/6-31G(d)-SDD(Pd) SCF energy in gas phase (au): -1730.347948
B3LYP-D3(BJ)/6-31G(d)-SDD(Pd) enthalpy in gas phase (au): -1730.017523
B3LYP-D3(BJ)/6-31G(d)-SDD(Pd) free energy in gas phase (au): -1730.111555
ωB97X-D/def2-TZVP SCF energy in solution (au): -1730.436084
ωB97X-D/def2-TZVP enthalpy in solution (au): -1730.105659
ωB97X-D/def2-TZVP free energy in solution (au): -1730.199691
imaginary frequency (cm^{-1}): -140.18

Cartesian coordinates

ATOM	X	Y	Z
Pd	-0.131350	0.044240	-0.660600
C	1.020630	1.732500	-0.815550
C	1.858270	0.663230	1.244910
C	-2.840860	-0.595000	0.209800
C	-1.773660	-2.630940	-0.220170
C	-2.756710	0.839320	0.186410
C	-4.021350	-1.254000	0.653700
C	-2.902540	-3.358990	0.209250
H	-0.869920	-3.119710	-0.571740
C	-3.863430	1.568640	0.619210
C	-5.122420	-0.471700	1.084580
C	-4.015740	-2.671530	0.641630
H	-2.878120	-4.443080	0.192220
C	-5.027940	0.904040	1.059950
H	-3.816550	2.647640	0.596760
H	-6.023800	-0.967620	1.431900
H	-4.901160	-3.204610	0.977640
H	-5.869990	1.505830	1.389240
N	-1.750720	-1.307780	-0.214440
N	-1.562390	1.357400	-0.304900
C	-1.307540	2.697870	-0.564370
O	-2.085400	3.620100	-0.360810
C	0.070140	2.863750	-1.204870
H	-0.082610	2.846790	-2.291920
H	0.463190	3.856590	-0.949750
C	1.645790	1.964210	0.586260
H	1.818750	1.541150	-1.534640
H	2.560720	2.560470	0.541700
H	0.892950	2.501380	1.190470
C	3.161550	0.187800	1.765750
O	3.249060	-0.669750	2.624400
O	4.198700	0.852780	1.230790
C	5.497470	0.388630	1.638820
H	6.209650	1.004610	1.091840
H	5.622500	0.507380	2.717770
H	5.614020	-0.665950	1.374690
S	2.664760	-1.102090	-0.807860
O	1.203570	-1.469820	-1.151480

O	3.372080	-0.418200	-1.899150
F	3.290350	-2.638170	-0.777480
H	1.010920	0.136060	1.668100

TS5

B3LYP-D3(BJ)/6-31G(d)-SDD(Pd) SCF energy in gas phase (au): -1730.338109
B3LYP-D3(BJ)/6-31G(d)-SDD(Pd) enthalpy in gas phase (au): -1730.006599
B3LYP-D3(BJ)/6-31G(d)-SDD(Pd) free energy in gas phase (au): -1730.098692
ωB97X-D/def2-TZVP SCF energy in solution (au): -1730.436031
ωB97X-D/def2-TZVP enthalpy in solution (au): -1730.104521
ωB97X-D/def2-TZVP free energy in solution (au): -1730.196614
imaginary frequency (cm^{-1}): -257.68

Cartesian coordinates

ATOM	X	Y	Z
Pd	-0.021810	0.303840	-0.171660
C	0.884840	2.097160	-0.515550
C	2.228980	0.434350	0.666100
C	-2.764960	-0.681800	0.206940
C	-1.391180	-2.574570	0.208370
C	-2.867100	0.748560	0.119710
C	-3.934300	-1.481930	0.365160
C	-2.498630	-3.431760	0.369310
H	-0.381210	-2.960530	0.129530
C	-4.133350	1.324740	0.187470
C	-5.202220	-0.853050	0.433010
C	-3.759300	-2.885440	0.445710
H	-2.338910	-4.502960	0.426210
C	-5.281450	0.519280	0.344470
H	-4.217190	2.399660	0.122470
H	-6.091970	-1.464190	0.551300
H	-4.634610	-3.518360	0.566420
H	-6.250470	1.007780	0.393320
N	-1.518550	-1.257640	0.132690
N	-1.660880	1.430470	-0.011340
C	-1.493280	2.804810	0.009950
O	-2.396660	3.628770	0.040620
C	-0.005140	3.182650	0.073210
H	0.133840	4.152030	-0.419900
H	0.226880	3.333300	1.137230
C	2.268830	1.836230	0.091700
H	0.921090	2.144910	-1.606690
H	3.069220	1.913620	-0.649810
H	2.520580	2.530090	0.907990
C	3.465150	-0.266800	1.165730
O	3.437120	-1.309450	1.780420
O	4.561370	0.461290	0.916570
C	5.798380	-0.143140	1.337640
H	6.573420	0.581290	1.090600
H	5.778530	-0.345430	2.410940
H	5.950690	-1.078590	0.793590
S	1.905780	-0.956040	-1.140110
O	1.611700	-2.337050	-0.761940
O	1.549860	-0.400120	-2.451560
F	3.560560	-0.942120	-1.249660
H	1.462780	0.273230	1.435570

TS6

B3LYP-D3(BJ)/6-31G(d)-SDD(Pd) SCF energy in gas phase (au): -1958.934070
B3LYP-D3(BJ)/6-31G(d)-SDD(Pd) enthalpy in gas phase (au): -1958.545456
B3LYP-D3(BJ)/6-31G(d)-SDD(Pd) free energy in gas phase (au): -1958.655129
ωB97X-D/def2-TZVP SCF energy in solution (au): -1959.080242
ωB97X-D/def2-TZVP enthalpy in solution (au): -1958.691628
ωB97X-D/def2-TZVP free energy in solution (au): -1958.801301
imaginary frequency (cm^{-1}): -162.51

Cartesian coordinates

ATOM	X	Y	Z
Pd	-0.044140	0.745500	0.227820
C	1.132490	0.850260	1.897360
C	1.774460	-1.341100	0.855600
C	-2.540290	-0.702970	-0.545890
C	-1.853580	0.519290	-2.413760
C	-2.295740	-1.060380	0.829420
C	-3.666680	-1.227470	-1.244960
C	-2.943280	0.043850	-3.175590
H	-1.096700	1.176250	-2.826980
C	-3.196180	-1.930170	1.448010
C	-4.549290	-2.111650	-0.574910
C	-3.839750	-0.821980	-2.592410
H	-3.053170	0.363330	-4.207050
C	-4.304110	-2.441500	0.741570
H	-3.021690	-2.196030	2.480630
H	-5.404790	-2.515490	-1.109980
H	-4.688670	-1.208460	-3.152160
H	-4.978390	-3.119430	1.259680
N	-1.669430	0.160210	-1.155350
N	-1.179730	-0.481650	1.405520
C	-0.882130	-0.490440	2.756620
O	-1.441620	-1.171200	3.615130
C	0.204420	0.534100	3.074580
H	-0.327810	1.456600	3.338200
H	0.758720	0.201710	3.964270
C	2.239440	-0.169760	1.647200
H	1.536050	1.859160	1.981770
H	3.069240	0.299120	1.107580
H	2.672680	-0.533010	2.603440
C	2.686260	-2.328340	0.281770
O	2.343500	-3.433980	-0.110180
O	3.969270	-1.887660	0.242790
C	4.834270	-2.655840	-0.589990
H	5.815930	-2.185520	-0.511730
H	4.876960	-3.699030	-0.262570
H	4.470130	-2.610650	-1.620830
S	1.872370	0.317560	-1.449650
O	1.158910	0.799950	-2.653930
O	3.125730	1.008940	-1.084190
F	2.439960	-1.174470	-2.074930
H	0.759240	-1.700140	0.954120
O	-0.584060	3.450790	1.578550
C	-0.092090	3.625180	0.461180
O	0.270030	2.705940	-0.385370
C	0.163960	5.034830	-0.076770
H	-0.184440	5.782350	0.640090

H	1.235470	5.168070	-0.261830
H	-0.348630	5.169720	-1.035250

A_DMA

B3LYP-D3(BJ)/6-31G(d)-SDD(Pd) SCF energy in gas phase (au): -2018.284195
B3LYP-D3(BJ)/6-31G(d)-SDD(Pd) enthalpy in gas phase (au): -2017.803574
B3LYP-D3(BJ)/6-31G(d)-SDD(Pd) free energy in gas phase (au): -2017.923787
ωB97X-D/def2-TZVP SCF energy in solution (au): -2018.404176
ωB97X-D/def2-TZVP enthalpy in solution (au): -2017.923555
ωB97X-D/def2-TZVP free energy in solution (au): -2018.043768

Cartesian coordinates

ATOM	X	Y	Z
Pd	1.134190	0.266020	0.091610
C	-0.475080	-0.987060	-0.125640
O	2.005830	-3.724120	-0.116660
C	1.494770	-2.614980	0.008440
C	-0.004830	-2.409140	0.219990
C	-1.695290	-0.543020	0.676460
N	2.174550	-1.415980	0.039310
C	3.554960	-1.271580	0.009360
C	4.499500	-2.294520	-0.084740
C	5.878830	-1.999800	-0.084050
C	6.357750	-0.709140	0.006120
C	5.437290	0.362410	0.101420
C	4.039340	0.078680	0.100340
N	3.113130	1.087320	0.189130
C	3.507160	2.347320	0.280830
C	4.869170	2.712140	0.289280
C	5.823110	1.722730	0.198960
H	-0.544880	-3.188220	-0.331900
H	-0.180910	-2.605480	1.288990
H	4.149800	-3.313940	-0.156120
H	6.579680	-2.826950	-0.158740
H	7.423210	-0.499050	0.004340
H	2.717180	3.089420	0.350760
H	5.144230	3.758740	0.367020
H	6.881640	1.969950	0.202430
H	-0.704020	-0.937500	-1.200380
H	-1.956380	0.491570	0.461960
H	-1.490820	-0.608360	1.750390
O	0.022600	2.102330	0.210200
C	-0.925460	2.376710	-0.568920
C	-0.873460	1.967910	-2.023740
H	0.103050	1.522200	-2.215280
H	-1.647240	1.223680	-2.233130
H	-1.023100	2.821900	-2.691970
N	-2.008280	3.029710	-0.110200
C	-3.218000	3.253790	-0.896700
H	-3.973910	2.505960	-0.633850
H	-3.609040	4.250900	-0.671750
H	-3.009050	3.199780	-1.963380
C	-2.137030	3.333910	1.315630
H	-2.357190	4.399420	1.440980
H	-2.954430	2.741870	1.738790
H	-1.203540	3.087720	1.816040
C	-2.954840	-1.386880	0.379050

C	-3.241920	-1.514660	-1.110260
O	-3.130030	-2.538760	-1.735980
O	-3.603520	-0.326880	-1.641800
C	-3.937490	-0.364650	-3.043300
H	-4.757700	-1.065040	-3.213690
H	-4.238240	0.651550	-3.296290
H	-3.069150	-0.673680	-3.629900
S	-4.415020	-0.660400	1.190300
O	-4.379530	0.794820	1.183820
O	-5.616730	-1.390250	0.832440
F	-4.029210	-1.097030	2.706610
H	-2.884540	-2.398930	0.781510

DMA

B3LYP-D3(BJ)/6-31G(d) SCF energy in gas phase (au): -287.843979
B3LYP-D3(BJ)/6-31G(d) enthalpy in gas phase (au): -287.701586
B3LYP-D3(BJ)/6-31G(d) free energy in gas phase (au): -287.741732
ωB97X-D/def2-TZVP SCF energy in solution (au): -287.862259
ωB97X-D/def2-TZVP enthalpy in solution (au): -287.719865
ωB97X-D/def2-TZVP free energy in solution (au): -287.760011

Cartesian coordinates

ATOM	X	Y	Z
C	-0.728410	-0.294420	-0.000010
O	-1.067120	-1.473480	-0.000200
N	0.595250	0.083050	0.000170
C	-1.774140	0.814300	0.000140
H	-1.693620	1.454060	0.886110
H	-1.693550	1.454170	-0.885760
H	-2.752100	0.332690	0.000030
C	1.622930	-0.944520	0.000120
H	2.259470	-0.852410	-0.890160
H	2.260200	-0.851620	0.889790
H	1.132510	-1.916680	0.000680
C	1.079930	1.448970	-0.000170
H	1.698920	1.639550	-0.888200
H	0.256730	2.161150	0.000220
H	1.699830	1.639600	0.887200

E

B3LYP-D3(BJ)/6-31G(d)-SDD(Pd) SCF energy in gas phase (au): -1730.403875
B3LYP-D3(BJ)/6-31G(d)-SDD(Pd) enthalpy in gas phase (au): -1730.069661
B3LYP-D3(BJ)/6-31G(d)-SDD(Pd) free energy in gas phase (au): -1730.163496
ωB97X-D/def2-TZVP SCF energy in solution (au): -1959.165160
ωB97X-D/def2-TZVP enthalpy in solution (au): -1958.830945
ωB97X-D/def2-TZVP free energy in solution (au): -1958.924780

Cartesian coordinates

ATOM	X	Y	Z
Pd	-0.115900	-0.027020	-1.216010
C	1.362050	1.384970	-1.555110
C	2.781570	-0.304490	-0.300400
C	-2.636500	-0.277750	0.247890
C	-2.235480	-2.354040	-0.759950
C	-2.205860	1.079460	0.418680
C	-3.778420	-0.778530	0.932950
C	-3.354560	-2.923630	-0.120760

H	-1.586720	-2.927060	-1.415360
C	-2.933110	1.895460	1.279030
C	-4.496920	0.090460	1.792450
C	-4.116970	-2.137400	0.717100
H	-3.594190	-3.967450	-0.290410
C	-4.069910	1.391320	1.949850
H	-2.618300	2.919680	1.416700
H	-5.368070	-0.284340	2.321170
H	-4.982120	-2.551290	1.228240
H	-4.613350	2.058750	2.612170
N	-1.905510	-1.085240	-0.583950
N	-1.090520	1.448090	-0.333080
C	-0.503960	2.701540	-0.370180
O	-0.915370	3.690570	0.217360
C	0.680490	2.741930	-1.348570
H	0.275030	3.111310	-2.299260
H	1.385540	3.501980	-0.992290
C	2.570270	1.167310	-0.639530
H	1.616170	1.181150	-2.598740
H	3.477290	1.540900	-1.135010
H	2.457770	1.727960	0.293120
S	1.176490	-0.787660	0.516410
O	0.927750	0.054180	1.694830
O	1.056700	-2.245630	0.618720
F	0.742860	-1.392680	-2.336050
C	3.974160	-0.629910	0.582980
O	4.799050	-1.470470	0.309840
O	3.992610	0.141540	1.679790
C	5.074600	-0.111870	2.594940
H	4.913690	0.571220	3.427770
H	5.045660	-1.150730	2.931750
H	6.033800	0.083070	2.109070
H	2.797240	-0.946270	-1.183760

E'

B3LYP-D3(BJ)/6-31G(d)-SDD(Pd) SCF energy in gas phase (au): -1959.008327
B3LYP-D3(BJ)/6-31G(d)-SDD(Pd) enthalpy in gas phase (au): -1958.616230
B3LYP-D3(BJ)/6-31G(d)-SDD(Pd) free energy in gas phase (au): -1958.722988
ωB97X-D/def2-TZVP SCF energy in solution (au): -1730.500037
ωB97X-D/def2-TZVP enthalpy in solution (au): -1730.107939
ωB97X-D/def2-TZVP free energy in solution (au): -1730.214698

Cartesian coordinates

ATOM	X	Y	Z
Pd	-0.134280	-0.005300	-0.332970
C	0.973670	1.580100	-1.034170
C	2.926250	0.011560	-1.140680
C	-3.051450	-0.567820	-0.031810
C	-1.956780	-2.616520	0.213600
C	-2.939350	0.853720	-0.269650
C	-4.330550	-1.181640	0.127640
C	-3.180300	-3.304040	0.371000
H	-1.001600	-3.134060	0.227870
C	-4.121420	1.597460	-0.318140
C	-5.498670	-0.382550	0.068710
C	-4.352980	-2.584720	0.333420
H	-3.178230	-4.379390	0.518650

C	-5.373540	0.974720	-0.146580
H	-4.044340	2.661420	-0.491360
H	-6.471940	-0.850740	0.190200
H	-5.313840	-3.080100	0.455300
H	-6.266630	1.594040	-0.193170
N	-1.908890	-1.312270	0.032550
N	-1.665590	1.346660	-0.422860
C	-1.287660	2.656660	-0.566640
O	-2.051120	3.619900	-0.657030
C	0.240490	2.820850	-0.544090
H	0.499160	3.696550	-1.155040
H	0.533590	3.025660	0.491250
C	2.440080	1.395090	-0.701030
H	0.785720	1.405920	-2.096620
H	3.026480	2.159070	-1.237870
H	2.608670	1.534820	0.367120
S	1.822510	-1.257260	-0.239950
O	2.381320	-1.362780	1.136210
O	1.779010	-2.502820	-1.046630
F	-0.342300	-0.444390	-2.233450
O	-0.099170	0.166060	1.683530
C	0.689660	0.855810	2.460460
O	1.388200	1.821350	2.160990
C	0.682500	0.297700	3.880280
H	1.330670	-0.585890	3.885450
H	1.074080	1.040370	4.579680
H	-0.322160	-0.017530	4.175810
C	4.357870	-0.326850	-0.836470
O	5.032880	-1.120970	-1.459830
O	4.809280	0.331870	0.253750
C	6.104610	-0.064790	0.704100
H	6.859530	0.116300	-0.067520
H	6.305450	0.540740	1.588920
H	6.107120	-1.128290	0.958810
H	2.729070	-0.189570	-2.195910

A''_c2
B3LYP-D3(BJ)/6-31G(d)-SDD(Pd) SCF energy in gas phase (au): -1958.986844
B3LYP-D3(BJ)/6-31G(d)-SDD(Pd) enthalpy in gas phase (au): -1958.594928
B3LYP-D3(BJ)/6-31G(d)-SDD(Pd) free energy in gas phase (au): -1958.705341
ωB97X-D/def2-TZVP SCF energy in solution (au): -1959.171285
ωB97X-D/def2-TZVP enthalpy in solution (au): -1958.779368
ωB97X-D/def2-TZVP free energy in solution (au): -1958.889781

Cartesian coordinates

ATOM	X	Y	Z
Pd	-1.007260	0.476500	-0.227190
C	0.840530	-0.319440	0.036710
O	-0.889110	-3.512020	0.723350
C	-0.673260	-2.358470	0.347530
C	0.706800	-1.850340	-0.055190
C	1.928300	0.244560	-0.883470
N	-1.631180	-1.377740	0.178030
C	-2.989650	-1.554310	0.321380
C	-3.654570	-2.739360	0.661380
C	-5.058910	-2.776800	0.751160
C	-5.849210	-1.667470	0.517280

C	-5.227410	-0.443050	0.173530
C	-3.803900	-0.386300	0.077050
N	-3.164640	0.778370	-0.251030
C	-3.856340	1.879740	-0.486440
C	-5.265610	1.910890	-0.412970
C	-5.940280	0.755910	-0.085060
H	1.469810	-2.385700	0.522590
H	0.850610	-2.164460	-1.099580
H	-3.058380	-3.619760	0.852160
H	-5.530400	-3.720410	1.017710
H	-6.932320	-1.714920	0.593810
H	-3.267650	2.757310	-0.739540
H	-5.795240	2.837240	-0.614470
H	-7.026170	0.744830	-0.019490
H	1.065980	-0.040860	1.075770
H	1.802020	1.323020	-1.001610
C	3.330530	-0.009530	-0.355140
H	1.840960	-0.187360	-1.882690
O	-0.565830	2.477010	-0.666460
C	0.255940	3.294460	-0.136460
O	1.229840	3.042660	0.608890
C	-0.029430	4.763180	-0.457010
H	-0.904430	5.092430	0.116890
H	0.824220	5.390760	-0.191340
H	-0.270470	4.879610	-1.518140
C	3.773880	0.622090	0.779530
O	3.150460	1.598400	1.402890
O	4.920240	0.244210	1.365290
C	5.395890	1.017020	2.465920
H	4.696930	0.980290	3.306400
H	6.345440	0.556250	2.742560
H	5.552140	2.061130	2.178270
S	4.270090	-1.284380	-1.039440
O	3.600850	-1.806670	-2.226170
O	5.703530	-1.016970	-1.070680
F	4.123330	-2.494700	0.070250
H	2.310380	1.979770	0.966090

A''_c3

B3LYP-D3(BJ)/6-31G(d)-SDD(Pd) SCF energy in gas phase (au): -1729.866482
B3LYP-D3(BJ)/6-31G(d)-SDD(Pd) enthalpy in gas phase (au): -1729.546036
B3LYP-D3(BJ)/6-31G(d)-SDD(Pd) free energy in gas phase (au): -1729.636500
ωB97X-D/def2-TZVP SCF energy in solution (au): -1730.024748
ωB97X-D/def2-TZVP enthalpy in solution (au): -1729.704302
ωB97X-D/def2-TZVP free energy in solution (au): -1729.794767

Cartesian coordinates

ATOM	X	Y	Z
Pd	-0.112840	0.064410	0.132990
C	1.048810	1.758950	0.130370
O	-2.119990	3.647300	0.078520
C	-1.317420	2.718520	0.191840
C	0.144600	2.913510	0.580200
C	2.373090	1.670870	0.900650
N	-1.612660	1.379050	0.046050
C	-2.872570	0.854280	-0.157380
C	-4.065220	1.570760	-0.309950

C	-5.289900	0.899740	-0.492830
C	-5.382830	-0.477810	-0.533150
C	-4.205190	-1.249820	-0.384570
C	-2.955230	-0.586440	-0.199400
N	-1.794610	-1.296520	-0.050630
C	-1.808530	-2.617910	-0.073390
C	-3.001260	-3.351620	-0.252380
C	-4.186490	-2.668140	-0.407310
H	0.481910	3.892020	0.210910
H	0.159000	2.976890	1.679920
H	-4.018080	2.649550	-0.281250
H	-6.190700	1.498960	-0.607140
H	-6.336430	-0.978990	-0.676760
H	-0.844480	-3.102010	0.055440
H	-2.967910	-4.436720	-0.265160
H	-5.123490	-3.202730	-0.547600
H	1.280780	1.862340	-0.938620
H	2.185380	1.643280	1.979960
C	3.197670	0.466900	0.471700
H	2.979170	2.565640	0.699230
C	3.816560	0.523110	-0.816770
O	4.053700	1.558510	-1.438830
O	4.191070	-0.703630	-1.331460
C	4.806420	-0.642080	-2.608100
H	4.138900	-0.195950	-3.353600
H	5.728730	-0.050910	-2.580710
H	5.031470	-1.677230	-2.878120
S	2.511900	-0.950920	1.064370
O	2.232620	-0.900340	2.499290
O	1.379050	-1.529180	0.234510
F	3.626690	-2.116460	0.868280

HOAc

B3LYP-D3(BJ)/6-31G(d) SCF energy in gas phase (au): -229.084610
B3LYP-D3(BJ)/6-31G(d) enthalpy in gas phase (au): -229.015532
B3LYP-D3(BJ)/6-31G(d) free energy in gas phase (au): -229.045022
ωB97X-D/def2-TZVP SCF energy in solution (au): -229.121897
ωB97X-D/def2-TZVP enthalpy in solution (au): -229.052819
ωB97X-D/def2-TZVP free energy in solution (au): -229.082309

Cartesian coordinates

ATOM	X	Y	Z
C	-0.092370	0.125670	-0.000020
O	-0.646030	1.201670	0.000000
O	-0.777520	-1.046940	-0.000010
C	1.396890	-0.109230	0.000010
H	1.683610	-0.690960	0.882160
H	1.683760	-0.691240	-0.881930
H	1.916290	0.849070	-0.000160
H	-1.722460	-0.803340	0.000040

TS2_c2

B3LYP-D3(BJ)/6-31G(d)-SDD(Pd) SCF energy in gas phase (au): -1958.987696
B3LYP-D3(BJ)/6-31G(d)-SDD(Pd) enthalpy in gas phase (au): -1958.597628
B3LYP-D3(BJ)/6-31G(d)-SDD(Pd) free energy in gas phase (au): -1958.709630
ωB97X-D/def2-TZVP SCF energy in solution (au): -1959.152414
ωB97X-D/def2-TZVP enthalpy in solution (au): -1958.762345

ωB97X-D/def2-TZVP free energy in solution (au): -1958.874347
imaginary frequency (cm^{-1}): -120.52

Cartesian coordinates

ATOM	X	Y	Z
Pd	-0.329090	1.103660	0.584230
C	1.128540	0.929700	1.992560
C	1.597870	0.097950	-0.216860
C	-2.518800	-0.532230	-0.510670
C	-2.295160	1.316350	-1.916920
C	-2.066590	-1.211190	0.676120
C	-3.531070	-1.097940	-1.338810
C	-3.289320	0.828320	-2.790930
H	-1.774830	2.251000	-2.104140
C	-2.660710	-2.434560	0.990250
C	-4.105320	-2.341870	-0.978090
C	-3.900180	-0.370050	-2.497670
H	-3.552500	1.396990	-3.676970
C	-3.666250	-2.978410	0.163760
H	-2.324900	-2.954440	1.875560
H	-4.875370	-2.776920	-1.609220
H	-4.667250	-0.778200	-3.151670
H	-4.099810	-3.935400	0.444290
N	-1.935250	0.663230	-0.828360
N	-1.070260	-0.570260	1.392390
C	-0.419850	-1.076280	2.494840
O	-0.668280	-2.146960	3.047370
C	0.638930	-0.100720	3.017950
H	0.170280	0.422410	3.863090
H	1.465480	-0.693740	3.430310
C	2.278680	0.469220	1.082040
H	1.310720	1.911900	2.433150
H	2.979640	1.283320	0.887780
H	2.841800	-0.373500	1.490760
C	-0.291410	3.849260	0.218470
O	0.428010	2.859740	-0.219160
C	0.163070	5.215810	-0.282390
H	1.154710	5.442930	0.125220
H	0.257810	5.207070	-1.373340
H	-0.543150	5.989630	0.026600
S	3.713660	-0.781790	-1.346530
O	4.213210	0.100310	-2.425130
O	3.805370	-2.241770	-1.581570
F	4.848130	-0.532390	-0.093730
C	1.048640	-1.253750	-0.504990
O	0.419890	-1.541890	-1.505520
O	1.352500	-2.122560	0.477080
C	0.942690	-3.476630	0.254010
H	1.575540	-3.915750	-0.521670
H	1.080910	-3.979690	1.210730
H	-0.106160	-3.511950	-0.046620
H	1.581870	0.794780	-1.043750
O	-1.259740	3.730940	0.980570

TS2_c3
B3LYP-D3(BJ)/6-31G(d)-SDD(Pd) SCF energy in gas phase (au): -1958.988403
B3LYP-D3(BJ)/6-31G(d)-SDD(Pd) enthalpy in gas phase (au): -1958.598355

B3LYP-D3(BJ)/6-31G(d)-SDD(Pd) free energy in gas phase (au): -1958.709677
ωB97X-D/def2-TZVP SCF energy in solution (au): -1959.152610
ωB97X-D/def2-TZVP enthalpy in solution (au): -1958.762562
ωB97X-D/def2-TZVP free energy in solution (au): -1958.873884
imaginary frequency (cm^{-1}): -115.25

Cartesian coordinates

ATOM	X	Y	Z
Pd	-0.377390	1.116250	0.576710
C	1.073870	1.036020	1.999470
C	1.602000	0.200840	-0.196530
C	-2.472910	-0.642490	-0.511370
C	-2.325860	1.192290	-1.945500
C	-1.999230	-1.280140	0.689830
C	-3.449610	-1.269140	-1.338380
C	-3.287820	0.643400	-2.819400
H	-1.849370	2.147800	-2.143730
C	-2.536590	-2.525680	1.018690
C	-3.966770	-2.533410	-0.962290
C	-3.843110	-0.578130	-2.511690
H	-3.570160	1.184760	-3.716570
C	-3.507580	-3.130170	0.193010
H	-2.184060	-3.014840	1.914940
H	-4.709740	-3.014660	-1.592350
H	-4.584060	-1.032900	-3.165060
H	-3.897290	-4.102330	0.485510
N	-1.944600	0.574450	-0.843730
N	-1.041230	-0.580800	1.403880
C	-0.374990	-1.039600	2.517620
O	-0.577020	-2.112550	3.084460
C	0.634970	-0.009530	3.032320
H	0.141870	0.496310	3.874040
H	1.489430	-0.558610	3.448350
C	2.252990	0.632530	1.098420
H	1.199720	2.030850	2.431280
H	2.901070	1.487890	0.895390
H	2.869580	-0.167030	1.518690
C	-0.477340	3.854680	0.160380
O	0.301640	2.896760	-0.244560
C	-0.085210	5.234070	-0.358000
H	0.884420	5.521100	0.064580
H	0.032160	5.210460	-1.446540
H	-0.837380	5.974870	-0.077920
S	3.829690	-0.525700	-1.207490
O	4.940050	-0.478830	-0.227360
O	4.112550	0.012800	-2.556550
F	3.650040	-2.203780	-1.488020
C	1.129060	-1.183770	-0.464340
O	0.515630	-1.520660	-1.458530
O	1.470900	-2.017690	0.536680
C	1.137170	-3.394400	0.331780
H	1.792160	-3.804350	-0.441590
H	1.306820	-3.878070	1.293450
H	0.091300	-3.493150	0.034780
H	1.554960	0.874780	-1.041170
O	-1.453040	3.699530	0.906100

TS2'_c2

B3LYP-D3(BJ)/6-31G(d)-SDD(Pd) SCF energy in gas phase (au): -1958.981222
B3LYP-D3(BJ)/6-31G(d)-SDD(Pd) enthalpy in gas phase (au): -1958.591213
B3LYP-D3(BJ)/6-31G(d)-SDD(Pd) free energy in gas phase (au): -1958.704548
ωB97X-D/def2-TZVP SCF energy in solution (au): -1959.147013
ωB97X-D/def2-TZVP enthalpy in solution (au): -1958.757005
ωB97X-D/def2-TZVP free energy in solution (au): -1958.870339
imaginary frequency (cm^{-1}): -168.50

Cartesian coordinates

ATOM	X	Y	Z
Pd	-0.381390	0.807680	0.740780
C	0.919420	0.366090	2.242700
C	1.434270	-0.524420	0.047620
C	-2.554700	-0.581520	-0.679310
C	-2.066180	1.343370	-1.905930
C	-2.262310	-1.382910	0.481370
C	-3.526960	-1.000690	-1.633760
C	-3.002730	0.997640	-2.903270
H	-1.460140	2.241490	-1.972000
C	-2.977170	-2.569310	0.650110
C	-4.226950	-2.213660	-1.419510
C	-3.728140	-0.163560	-2.760420
H	-3.134600	1.645430	-3.763910
C	-3.945660	-2.963670	-0.296300
H	-2.767190	-3.174950	1.519910
H	-4.967030	-2.537690	-2.146120
H	-4.458420	-0.459270	-3.510130
H	-4.476630	-3.897690	-0.129120
N	-1.864360	0.588980	-0.843310
N	-1.283570	-0.882870	1.324060
C	-0.892240	-1.421840	2.527620
O	-1.335110	-2.450120	3.036930
C	0.146020	-0.527420	3.216250
H	-0.413730	0.097640	3.925650
H	0.812650	-1.167930	3.810760
C	2.009510	-0.365790	1.437780
H	1.282740	1.288240	2.700200
H	2.933720	0.209900	1.383230
H	2.251110	-1.347020	1.860070
C	-0.255070	3.562120	0.744060
O	0.465270	2.628460	0.203310
C	0.228100	4.975250	0.432600
H	1.231360	5.120690	0.848770
H	0.304340	5.115620	-0.651230
H	-0.454490	5.715310	0.856050
S	3.255560	-2.003060	-0.841900
O	4.111050	-1.458000	-1.920280
O	2.824220	-3.411580	-0.973500
F	4.313780	-2.046320	0.493110
O	-1.248530	3.364110	1.456850
C	1.743630	0.371540	-1.099350
O	1.166570	0.334830	-2.170840
O	2.770950	1.191330	-0.823610
C	3.175560	2.027990	-1.907490
H	4.011330	2.615420	-1.524960
H	3.493000	1.410610	-2.751490

H	2.351260	2.681240	-2.204760
H	0.781170	-1.354650	-0.179140

TS2'_c3

B3LYP-D3(BJ)/6-31G(d)-SDD(Pd) SCF energy in gas phase (au): -1958.981783
B3LYP-D3(BJ)/6-31G(d)-SDD(Pd) enthalpy in gas phase (au): -1958.591790
B3LYP-D3(BJ)/6-31G(d)-SDD(Pd) free energy in gas phase (au): -1958.704573
ωB97X-D/def2-TZVP SCF energy in solution (au): -1959.146886
ωB97X-D/def2-TZVP enthalpy in solution (au): -1958.756892
ωB97X-D/def2-TZVP free energy in solution (au): -1958.869676
imaginary frequency (cm^{-1}): -168.88

Cartesian coordinates

ATOM	X	Y	Z
Pd	-0.384820	0.831860	0.728730
C	0.864280	0.414460	2.280270
C	1.419980	-0.563610	0.129810
C	-2.544430	-0.562020	-0.707290
C	-1.982460	1.311620	-1.980890
C	-2.301130	-1.330360	0.486630
C	-3.497370	-0.993650	-1.675480
C	-2.897130	0.951310	-2.993260
H	-1.356520	2.195060	-2.058290
C	-3.044190	-2.496630	0.672860
C	-4.227450	-2.185260	-1.442470
C	-3.649610	-0.190360	-2.834010
H	-2.991380	1.572760	-3.877920
C	-3.993330	-2.903190	-0.287950
H	-2.871130	-3.077290	1.567490
H	-4.952920	-2.518810	-2.179420
H	-4.364090	-0.496540	-3.594640
H	-4.547600	-3.820810	-0.106130
N	-1.826330	0.588990	-0.888950
N	-1.336890	-0.821410	1.340680
C	-0.988030	-1.329340	2.570310
O	-1.460750	-2.335390	3.097350
C	0.045270	-0.430220	3.260200
H	-0.523830	0.229620	3.929510
H	0.681370	-1.061010	3.897070
C	1.958040	-0.368290	1.529790
H	1.237340	1.342020	2.718420
H	2.897260	0.183390	1.479900
H	2.168310	-1.338710	1.994060
C	-0.202020	3.582300	0.648380
O	0.512930	2.617450	0.157710
C	0.319960	4.974660	0.307070
H	1.312750	5.113490	0.749780
H	0.432450	5.078150	-0.777740
H	-0.359590	5.741590	0.685210
S	3.248650	-2.127830	-0.544530
O	2.767370	-3.396760	-1.131190
O	4.336500	-2.220670	0.455000
F	4.011190	-1.353070	-1.860600
O	-1.218930	3.427860	1.338560
C	1.782990	0.287900	-1.035790
O	1.232300	0.235540	-2.119830
O	2.819630	1.095480	-0.753680

C	3.286960	1.871460	-1.856150
H	4.110280	2.469690	-1.463270
H	3.638170	1.205450	-2.648600
H	2.487840	2.516060	-2.230530
H	0.752120	-1.382820	-0.094440

A''_CO_2Ph

B3LYP-D3(BJ)/6-31G(d)-SDD(Pd) SCF energy in gas phase (au): -1921.645247
B3LYP-D3(BJ)/6-31G(d)-SDD(Pd) enthalpy in gas phase (au): -1921.267947
B3LYP-D3(BJ)/6-31G(d)-SDD(Pd) free energy in gas phase (au): -1921.370524
ωB97X-D/def2-TZVP SCF energy in solution (au): -1921.787243
ωB97X-D/def2-TZVP enthalpy in solution (au): -1921.409943
ωB97X-D/def2-TZVP free energy in solution (au): -1921.512520

Cartesian coordinates

ATOM	X	Y	Z
Pd	-0.711740	-0.507120	-0.337160
C	0.244950	-2.231490	0.093470
O	-3.099100	-3.641970	0.763140
C	-2.213400	-2.874300	0.381920
C	-0.809480	-3.343530	-0.015950
C	1.487590	-2.431920	-0.775180
N	-2.347600	-1.514040	0.199740
C	-3.515290	-0.792360	0.307090
C	-4.767780	-1.274020	0.704250
C	-5.881950	-0.415410	0.766710
C	-5.801490	0.927320	0.451390
C	-4.556990	1.464850	0.041920
C	-3.418430	0.607640	-0.039860
N	-2.200070	1.086540	-0.445270
C	-2.051950	2.366010	-0.744710
C	-3.122910	3.283330	-0.678620
C	-4.364530	2.829750	-0.293770
H	-0.557410	-4.240150	0.566950
H	-0.886520	-3.668140	-1.065760
H	-4.851400	-2.319930	0.962790
H	-6.835450	-0.832950	1.083020
H	-6.669520	1.578200	0.514890
H	-1.051810	2.672430	-1.038950
H	-2.954410	4.325530	-0.932090
H	-5.212690	3.508580	-0.236950
H	0.555390	-2.115210	1.142910
H	1.182690	-2.488010	-1.831690
C	2.515060	-1.331120	-0.603280
H	1.968600	-3.389060	-0.539660
C	2.167980	0.004580	-0.740120
O	1.014590	0.476730	-0.974820
O	3.200820	0.920240	-0.632360
S	4.066370	-1.735390	0.014770
O	4.120990	-3.157490	0.352430
O	5.201260	-1.124620	-0.670920
F	4.080040	-0.975140	1.498730
C	2.900130	2.153380	-0.083240
C	2.284780	3.152240	-0.837290
C	3.290730	2.382470	1.235190
C	2.055210	4.398860	-0.254050
H	1.989870	2.936560	-1.857460

C	3.059270	3.633890	1.807230
H	3.761330	1.571100	1.780170
C	2.440380	4.643810	1.066680
H	1.573210	5.181890	-0.834340
H	3.360440	3.818120	2.835240
H	2.259150	5.616900	1.515630

TS2_CO_2Ph

B3LYP-D3(BJ)/6-31G(d)-SDD(Pd) SCF energy in gas phase (au): -2150.748468
B3LYP-D3(BJ)/6-31G(d)-SDD(Pd) enthalpy in gas phase (au): -2150.301737
B3LYP-D3(BJ)/6-31G(d)-SDD(Pd) free energy in gas phase (au): -2150.423307
ωB97X-D/def2-TZVP SCF energy in solution (au): -2150.891761
ωB97X-D/def2-TZVP enthalpy in solution (au): -2150.445031
ωB97X-D/def2-TZVP free energy in solution (au): -2150.566600
imaginary frequency (cm^{-1}): -158.17

Cartesian coordinates

ATOM	X	Y	Z
Pd	-1.668910	-0.230210	0.567280
C	-1.373120	1.055840	2.115050
C	-0.399590	1.640720	-0.022000
C	-0.292590	-2.523520	-0.654220
C	-1.987440	-1.889660	-2.128240
C	0.363170	-2.294540	0.606260
C	0.170390	-3.532090	-1.548100
C	-1.587790	-2.856890	-3.074440
H	-2.821800	-1.218650	-2.310030
C	1.437440	-3.118490	0.938820
C	1.277390	-4.332690	-1.173490
C	-0.517840	-3.671190	-2.779290
H	-2.123420	-2.942700	-4.014320
C	1.878960	-4.119080	0.048420
H	1.926270	-2.967880	1.889560
H	1.636210	-5.098720	-1.855370
H	-0.182580	-4.427320	-3.485340
H	2.728480	-4.730210	0.342860
N	-1.367480	-1.739250	-0.973460
N	-0.153390	-1.258550	1.371120
C	0.314820	-0.839600	2.589090
O	1.259100	-1.335560	3.210300
C	-0.521980	0.312470	3.152410
H	-1.180980	-0.139930	3.906200
H	0.155640	0.987500	3.692520
C	-0.662010	2.194240	1.360460
H	-2.342180	1.365330	2.510840
H	-1.299060	3.076370	1.268670
H	0.271700	2.503310	1.839700
C	-4.370730	0.236030	0.124900
O	-3.266470	0.821970	-0.231750
C	-5.630000	0.926030	-0.388380
H	-5.718420	1.915950	0.073460
H	-5.562490	1.080650	-1.470420
H	-6.514930	0.332200	-0.149100
S	0.603590	3.739040	-0.892540
O	1.893770	3.709880	-1.621400
O	0.367810	4.903120	-0.010790
F	-0.534470	3.947870	-2.129950

C	0.845610	0.937120	-0.427280
O	1.001390	0.362920	-1.480890
O	1.809100	1.057060	0.534680
H	-1.123250	1.785040	-0.812060
O	-4.432760	-0.783920	0.822540
C	3.095290	0.593770	0.246500
C	3.637710	-0.361470	1.099280
C	3.817150	1.140170	-0.812910
C	4.945510	-0.794450	0.871790
H	3.039490	-0.746230	1.917880
C	5.120160	0.691390	-1.026320
H	3.361210	1.906030	-1.430900
C	5.686590	-0.275970	-0.191390
H	5.381110	-1.541850	1.529990
H	5.696550	1.106240	-1.849100
H	6.703330	-0.618850	-0.366340

TS2'_CO_2Ph

B3LYP-D3(BJ)/6-31G(d)-SDD(Pd) SCF energy in gas phase (au): -2150.742381
B3LYP-D3(BJ)/6-31G(d)-SDD(Pd) enthalpy in gas phase (au): -2150.295839
B3LYP-D3(BJ)/6-31G(d)-SDD(Pd) free energy in gas phase (au): -2150.418807
ωB97X-D/def2-TZVP SCF energy in solution (au): -2150.888213
ωB97X-D/def2-TZVP enthalpy in solution (au): -2150.441671
ωB97X-D/def2-TZVP free energy in solution (au): -2150.564638
imaginary frequency (cm^{-1}): -179.68

Cartesian coordinates

ATOM	X	Y	Z
Pd	-0.817940	-0.592010	-1.053530
C	-0.524400	0.742960	-2.559790
C	0.369810	1.343760	-0.397300
C	-2.578160	-0.860230	1.285550
C	-0.905100	-2.472810	1.507580
C	-3.131500	0.206910	0.492630
C	-3.154700	-1.217510	2.538940
C	-1.406130	-2.882750	2.761170
H	-0.019840	-2.921700	1.067740
C	-4.260940	0.863520	0.981770
C	-4.302210	-0.522250	2.994120
C	-2.525230	-2.259730	3.265550
H	-0.903130	-3.673780	3.307750
C	-4.828410	0.489250	2.217800
H	-4.684030	1.664530	0.392910
H	-4.743650	-0.790350	3.950090
H	-2.937300	-2.550770	4.228980
H	-5.705910	1.029580	2.564550
N	-1.475000	-1.512750	0.805760
N	-2.470100	0.476530	-0.695000
C	-2.829280	1.416080	-1.633380
O	-3.801240	2.166370	-1.566510
C	-1.889680	1.376640	-2.845510
H	-2.413320	0.786600	-3.610170
H	-1.797580	2.398000	-3.240460
C	0.489280	1.670120	-1.868850
H	-0.100550	0.232400	-3.426710
H	1.507220	1.456570	-2.198350
H	0.291700	2.734490	-2.044010

C	0.366650	-2.819670	-2.154090
O	0.825840	-1.792660	-1.505350
C	1.431140	-3.843350	-2.535570
H	2.239560	-3.355600	-3.090640
H	1.872580	-4.273180	-1.628580
H	0.994190	-4.641770	-3.138950
S	1.565320	3.399750	0.369720
O	1.638190	4.447880	-0.672790
O	2.735870	3.285290	1.272180
F	0.337610	3.951050	1.395220
O	-0.824810	-2.991470	-2.443960
C	1.350680	0.557710	0.394910
O	1.166630	0.165370	1.526140
O	2.505580	0.369500	-0.301410
H	-0.463250	1.723590	0.175910
C	3.535860	-0.312940	0.344780
C	4.347990	0.372180	1.243810
C	3.751110	-1.646450	0.013380
C	5.418220	-0.311870	1.821560
H	4.122670	1.409750	1.470230
C	4.827740	-2.314670	0.598870
H	3.064920	-2.120500	-0.679080
C	5.662070	-1.650410	1.501200
H	6.063790	0.205130	2.526740
H	5.012640	-3.356840	0.350170
H	6.499390	-2.174550	1.955160

A (optimized in DMA)

B3LYP-D3(BJ)/6-31G(d)-SDD(Pd) SCF energy in solution (au): -1959.094334
B3LYP-D3(BJ)/6-31G(d)-SDD(Pd) enthalpy in solution (au): -1958.701391
B3LYP-D3(BJ)/6-31G(d)-SDD(Pd) free energy in solution (au): -1958.811479
ωB97X-D/def2-TZVP SCF energy in solution (au): -1959.192859
ωB97X-D/def2-TZVP enthalpy in solution (au): -1958.799916
ωB97X-D/def2-TZVP free energy in solution (au): -1958.910005

Cartesian coordinates

ATOM	X	Y	Z
Pd	-0.932190	0.288250	-0.164610
C	0.692310	-0.842790	0.315880
O	-1.737610	-3.573720	0.963080
C	-1.255040	-2.504850	0.570800
C	0.247940	-2.316400	0.359450
C	1.895260	-0.594140	-0.593010
N	-1.962360	-1.381610	0.222970
C	-3.347440	-1.266540	0.203130
C	-4.277900	-2.267700	0.487340
C	-5.664350	-2.012950	0.406780
C	-6.161810	-0.777020	0.049740
C	-5.257200	0.274440	-0.244930
C	-3.855060	0.030880	-0.166860
N	-2.947480	1.025610	-0.439590
C	-3.371510	2.232650	-0.781430
C	-4.741260	2.553610	-0.884660
C	-5.675440	1.578520	-0.617260
H	0.778290	-2.904420	1.120850
H	0.472590	-2.798450	-0.604720

H	-3.915090	-3.244730	0.771960
H	-6.351540	-2.823300	0.636410
H	-7.230070	-0.590250	-0.008100
H	-2.601410	2.972250	-0.979470
H	-5.035350	3.558030	-1.170140
H	-6.738950	1.791130	-0.685650
H	0.915720	-0.492640	1.329520
H	1.970100	0.459590	-0.863470
C	3.223550	-1.001020	0.085730
H	1.791920	-1.170560	-1.519010
O	0.151720	2.008900	-0.610550
C	0.817690	2.592610	0.335910
O	0.777880	2.304970	1.542590
C	1.742960	3.700510	-0.159780
H	1.282400	4.266610	-0.975360
H	2.012420	4.377440	0.655160
H	2.661720	3.245600	-0.550410
C	3.500000	-0.141530	1.314710
O	3.341080	-0.532020	2.449630
O	3.880570	1.088340	0.960510
C	4.029570	2.050510	2.031300
H	4.649800	1.637150	2.829140
H	4.516320	2.913120	1.577490
H	3.036400	2.315780	2.398090
S	4.616760	-0.853870	-1.072330
O	4.487260	0.263210	-1.993150
O	5.880150	-1.141070	-0.407760
F	4.270480	-2.183110	-1.957020
H	3.229950	-2.051400	0.384760

A' (optimized in DMA)

B3LYP-D3(BJ)/6-31G(d)-SDD(Pd) SCF energy in solution (au): -1959.089966
B3LYP-D3(BJ)/6-31G(d)-SDD(Pd) enthalpy in solution (au): -1958.697301
B3LYP-D3(BJ)/6-31G(d)-SDD(Pd) free energy in solution (au): -1958.811179
ωB97X-D/def2-TZVP SCF energy in solution (au): -1959.190623
ωB97X-D/def2-TZVP enthalpy in solution (au): -1958.797958
ωB97X-D/def2-TZVP free energy in solution (au): -1958.911836

Cartesian coordinates

ATOM	X	Y	Z
Pd	0.947800	0.372070	-0.182850
C	-0.788170	-0.704380	-0.176880
O	1.304600	-3.610570	0.753290
C	0.953760	-2.444010	0.539290
C	-0.508160	-2.002500	0.598820
C	-1.974890	0.087170	0.351630
N	1.786640	-1.385040	0.282950
C	3.175510	-1.418750	0.297230
C	3.986870	-2.528740	0.538810
C	5.393970	-2.411010	0.540340
C	6.028140	-1.209140	0.303830
C	5.247630	-0.052300	0.051720
C	3.826320	-0.157090	0.047810
N	3.035590	0.940080	-0.192940
C	3.592590	2.118170	-0.426940
C	4.990660	2.305880	-0.440610
C	5.810110	1.225650	-0.202260

H	-1.140390	-2.840160	0.276080
H	-0.728920	-1.844760	1.666570
H	3.515070	-3.482490	0.726400
H	5.985650	-3.302270	0.733410
H	7.111170	-1.129050	0.304760
H	2.910360	2.942710	-0.610740
H	5.396030	3.292780	-0.637090
H	6.891330	1.334210	-0.205240
H	-0.943340	-0.943900	-1.236490
H	-2.085960	1.023830	-0.195510
C	-3.346050	-0.630920	0.348160
H	-1.810470	0.347640	1.404570
O	0.192120	2.938600	1.357730
C	-0.143330	3.041540	0.168770
O	0.057470	2.161690	-0.761540
C	-0.908120	4.274230	-0.308070
H	-0.780540	5.104960	0.391370
H	-0.587620	4.579810	-1.309140
H	-1.977350	4.031130	-0.364780
H	-3.314620	-1.569220	0.908180
S	-3.875150	-1.172360	-1.305390
O	-3.081290	-2.296000	-1.775670
O	-5.327280	-1.200140	-1.404730
F	-3.397310	0.109470	-2.186930
C	-4.434750	0.283160	0.901480
O	-4.633500	1.411150	0.502640
O	-5.097680	-0.313690	1.893190
C	-6.132080	0.477990	2.521880
H	-6.552390	-0.160190	3.298760
H	-5.702340	1.382320	2.959180
H	-6.897380	0.746530	1.789840

A'' (optimized in DMA)
B3LYP-D3(BJ)/6-31G(d)-SDD(Pd) SCF energy in solution (au): -1729.966295
B3LYP-D3(BJ)/6-31G(d)-SDD(Pd) enthalpy in solution (au): -1729.645496
B3LYP-D3(BJ)/6-31G(d)-SDD(Pd) free energy in solution (au): -1729.737310
ωB97X-D/def2-TZVP SCF energy in solution (au): -1730.048715
ωB97X-D/def2-TZVP enthalpy in solution (au): -1729.727916
ωB97X-D/def2-TZVP free energy in solution (au): -1729.819730

Cartesian coordinates

ATOM	X	Y	Z
Pd	-0.351870	0.007270	-0.195050
C	0.947470	-1.529640	-0.022240
O	-2.022180	-3.709550	0.167570
C	-1.316540	-2.712070	-0.016180
C	0.164900	-2.799710	-0.386910
C	2.197120	-1.293460	-0.868570
N	-1.742280	-1.405810	0.021670
C	-3.055240	-0.974860	0.162370
C	-4.175810	-1.773630	0.394330
C	-5.462980	-1.203530	0.501680
C	-5.671860	0.154690	0.386260
C	-4.564550	1.008530	0.150110
C	-3.258740	0.447260	0.037690
N	-2.162590	1.242690	-0.195390
C	-2.313920	2.553050	-0.314380

C	-3.569960	3.186990	-0.212340
C	-4.686540	2.416050	0.017970
H	0.593370	-3.708230	0.058010
H	0.195870	-2.945880	-1.478110
H	-4.038660	-2.841270	0.488180
H	-6.306520	-1.864420	0.683730
H	-6.664850	0.585580	0.473780
H	-1.410910	3.127960	-0.497830
H	-3.635820	4.264620	-0.317910
H	-5.670640	2.869300	0.101190
H	1.229840	-1.561130	1.041770
H	1.900350	-1.175070	-1.922280
C	3.009450	-0.091380	-0.424520
H	2.845830	-2.176910	-0.839130
C	2.403720	1.163950	-0.236320
O	1.175360	1.419650	-0.442310
O	3.192680	2.178550	0.174420
C	2.551030	3.431180	0.450490
H	2.103800	3.854170	-0.453370
H	1.781520	3.320250	1.219940
H	3.346800	4.085110	0.811170
S	4.611180	-0.347480	0.079020
O	5.015840	-1.739080	-0.145000
O	5.563840	0.714850	-0.245100
F	4.542360	-0.253630	1.751290

TS2 (optimized in DMA)

B3LYP-D3(BJ)/6-31G(d)-SDD(Pd) SCF energy in solution (au): -1959.075947
B3LYP-D3(BJ)/6-31G(d)-SDD(Pd) enthalpy in solution (au): -1958.685955
B3LYP-D3(BJ)/6-31G(d)-SDD(Pd) free energy in solution (au): -1958.798688
ωB97X-D/def2-TZVP SCF energy in solution (au): -1959.152095
ωB97X-D/def2-TZVP enthalpy in solution (au): -1958.762103
ωB97X-D/def2-TZVP free energy in solution (au): -1958.874836
imaginary frequency (cm^{-1}): -297.45

Cartesian coordinates

ATOM	X	Y	Z
Pd	-0.220060	0.980760	0.680390
C	1.150320	0.511970	2.115830
C	1.774490	-0.185180	-0.125990
C	-2.598670	-0.209190	-0.569630
C	-2.110360	1.730920	-1.774940
C	-2.258380	-1.069770	0.532270
C	-3.683720	-0.525000	-1.436870
C	-3.168150	1.492020	-2.676960
H	-1.467750	2.600580	-1.876460
C	-3.027470	-2.217670	0.721570
C	-4.438020	-1.703180	-1.203830
C	-3.947910	0.370280	-2.505430
H	-3.348740	2.190340	-3.487030
C	-4.103000	-2.518640	-0.143920
H	-2.783220	-2.875650	1.542660
H	-5.264620	-1.943780	-1.865640
H	-4.769970	0.153890	-3.182210
H	-4.674790	-3.424190	0.040970
N	-1.840670	0.916490	-0.768190
N	-1.179380	-0.655560	1.307340

C	-0.647750	-1.326050	2.380370
O	-1.062180	-2.393070	2.840800
C	0.508910	-0.553110	3.015630
H	0.085350	-0.063350	3.903690
H	1.245880	-1.279700	3.381850
C	2.333320	0.029710	1.260690
H	1.408570	1.417730	2.668910
H	3.109990	0.795210	1.202400
H	2.787370	-0.887570	1.650360
C	0.207450	3.756460	0.500550
O	0.781290	2.684710	0.042140
C	0.843580	5.053780	0.025210
H	1.896880	5.086990	0.326760
H	0.817130	5.104990	-1.069410
H	0.320550	5.918250	0.440850
S	3.774160	-0.924070	-1.156740
O	3.791020	-2.222920	-1.862640
O	5.002100	-0.554340	-0.421410
F	3.740740	0.204360	-2.430050
C	1.036570	-1.413730	-0.547070
O	0.442850	-1.522460	-1.604130
O	1.174020	-2.397860	0.352850
C	0.498670	-3.631010	0.042480
H	0.946380	-4.088350	-0.843780
H	0.642100	-4.267770	0.914430
H	-0.564370	-3.451730	-0.125710
H	1.759340	0.630650	-0.834970
O	-0.773550	3.759990	1.259780

TS2' (optimized in DMA)

B3LYP-D3(BJ)/6-31G(d)-SDD(Pd) SCF energy in solution (au): -1959.070712
B3LYP-D3(BJ)/6-31G(d)-SDD(Pd) enthalpy in solution (au): -1958.680838
B3LYP-D3(BJ)/6-31G(d)-SDD(Pd) free energy in solution (au): -1958.795769
ωB97X-D/def2-TZVP SCF energy in solution (au): -1959.147656
ωB97X-D/def2-TZVP enthalpy in solution (au): -1958.757782
ωB97X-D/def2-TZVP free energy in solution (au): -1958.872713
imaginary frequency (cm^{-1}): -295.51

Cartesian coordinates

ATOM	X	Y	Z
Pd	-0.426990	0.770670	0.797080
C	1.022060	0.539440	2.215420
C	1.679660	-0.281610	0.034200
C	-2.431610	-0.904540	-0.552090
C	-2.472740	1.175050	-1.612870
C	-1.882260	-1.718660	0.500400
C	-3.398390	-1.432440	-1.455500
C	-3.431060	0.730410	-2.547570
H	-2.080500	2.187140	-1.644140
C	-2.327120	-3.035530	0.604460
C	-3.821380	-2.778300	-1.309280
C	-3.888300	-0.565940	-2.466650
H	-3.790790	1.411900	-3.311000
C	-3.287800	-3.546890	-0.296740
H	-1.926790	-3.658540	1.391330
H	-4.556660	-3.181370	-1.999300
H	-4.625480	-0.942100	-3.170760

H	-3.606210	-4.579340	-0.179380
N	-1.996420	0.391420	-0.661000
N	-0.955010	-1.086890	1.324460
C	-0.339650	-1.625400	2.425450
O	-0.492860	-2.773350	2.851910
C	0.537580	-0.588910	3.132510
H	-0.083020	-0.165900	3.935040
H	1.373490	-1.110040	3.619480
C	2.243380	0.165810	1.360510
H	1.178480	1.479120	2.749790
H	2.895830	1.026260	1.203540
H	2.836820	-0.637560	1.813340
C	-0.821810	3.534430	0.793250
O	0.056560	2.722660	0.290250
C	-0.624280	4.988210	0.391740
H	0.384540	5.320520	0.661440
H	-0.719510	5.088110	-0.695960
H	-1.361520	5.631040	0.878490
S	3.592790	-1.445910	-0.780910
O	4.572450	-1.833710	0.257590
O	4.145090	-1.074360	-2.101030
F	2.784650	-2.910390	-1.103260
O	-1.757700	3.190760	1.533920
C	1.614580	0.566760	-1.193240
O	0.976500	0.266510	-2.187760
O	2.400600	1.644020	-1.092780
C	2.362520	2.547490	-2.209190
H	3.044020	3.358390	-1.951340
H	2.698290	2.043640	-3.119220
H	1.349600	2.932600	-2.344490
H	1.138950	-1.215020	-0.028390

B (optimized in DMA)

B3LYP-D3(BJ)/6-31G(d)-SDD(Pd) SCF energy in solution (au): -1959.087546
B3LYP-D3(BJ)/6-31G(d)-SDD(Pd) enthalpy in solution (au): -1958.696452
B3LYP-D3(BJ)/6-31G(d)-SDD(Pd) free energy in solution (au): -1958.811295
ωB97X-D/def2-TZVP SCF energy in solution (au): -1959.172880
ωB97X-D/def2-TZVP enthalpy in solution (au): -1958.781787
ωB97X-D/def2-TZVP free energy in solution (au): -1958.896629

Cartesian coordinates

ATOM	X	Y	Z
Pd	-0.742070	1.148960	0.330580
C	0.614600	1.731000	1.754020
C	1.188280	0.639680	-0.237270
C	-2.155730	-1.321120	-0.461050
C	-2.536680	0.215430	-2.177690
C	-1.568600	-1.582060	0.823910
C	-2.844600	-2.346760	-1.169260
C	-3.240860	-0.739840	-2.939460
H	-2.390710	1.228650	-2.541020
C	-1.686110	-2.866710	1.348210
C	-2.946430	-3.638740	-0.593930
C	-3.389420	-2.012740	-2.435610
H	-3.647580	-0.460210	-3.905170
C	-2.372860	-3.876180	0.636200
H	-1.245070	-3.079530	2.310600

H	-3.471110	-4.420470	-1.134790
H	-3.919660	-2.776320	-2.997920
H	-2.442170	-4.864260	1.082670
N	-2.018220	-0.067210	-0.994630
N	-0.923840	-0.503500	1.431030
C	-0.254540	-0.521050	2.633030
O	-0.205770	-1.477610	3.403890
C	0.361900	0.838710	2.965090
H	-0.341360	1.334600	3.647160
H	1.289050	0.669030	3.527040
C	1.848290	1.396840	0.908650
H	0.470040	2.793770	1.948330
H	2.331950	2.300640	0.531860
H	2.592070	0.800070	1.441080
C	-1.859570	3.432010	-0.187860
O	-0.750940	2.978480	-0.679080
C	-2.358430	4.755870	-0.703050
H	-1.538280	5.478750	-0.753180
H	-2.746850	4.621680	-1.720000
H	-3.157800	5.141360	-0.066520
S	4.257880	-0.104430	-0.910470
O	4.734380	-1.509760	-1.089020
O	5.115110	0.691710	0.018350
F	4.722710	0.595390	-2.465680
C	1.328860	-0.841540	-0.368670
O	1.004570	-1.462160	-1.366390
O	1.834260	-1.416010	0.735640
C	2.030910	-2.838770	0.671100
H	2.825790	-3.061190	-0.044600
H	2.327870	-3.135620	1.677370
H	1.105900	-3.343570	0.385270
H	1.219610	1.113150	-1.216420
O	-2.496180	2.766650	0.667520

B' (optimized in DMA)

B3LYP-D3(BJ)/6-31G(d)-SDD(Pd) SCF energy in solution (au): -1959.083810
B3LYP-D3(BJ)/6-31G(d)-SDD(Pd) enthalpy in solution (au): -1958.692665
B3LYP-D3(BJ)/6-31G(d)-SDD(Pd) free energy in solution (au): -1958.808354
ωB97X-D/def2-TZVP SCF energy in solution (au): -1959.170244
ωB97X-D/def2-TZVP enthalpy in solution (au): -1958.779099
ωB97X-D/def2-TZVP free energy in solution (au): -1958.894788

Cartesian coordinates

ATOM	X	Y	Z
Pd	-0.644850	0.915700	0.596070
C	0.586710	1.063330	2.229650
C	1.174780	-0.008000	0.232800
C	-2.294680	-1.172130	-0.677310
C	-2.214900	0.601610	-2.194240
C	-1.935050	-1.675540	0.620030
C	-3.037750	-1.974690	-1.589990
C	-2.949030	-0.125460	-3.154040
H	-1.872240	1.612580	-2.394100
C	-2.330940	-2.968220	0.952900
C	-3.421940	-3.284810	-1.207140
C	-3.353450	-1.406310	-2.850900
H	-3.179950	0.331520	-4.110160

C	-3.067980	-3.754740	0.038810
H	-2.071860	-3.359480	1.925810
H	-3.986910	-3.896560	-1.903920
H	-3.916970	-1.996990	-3.567800
H	-3.356740	-4.758050	0.339530
N	-1.902680	0.096680	-1.013740
N	-1.223520	-0.797330	1.439920
C	-0.779700	-1.036210	2.718650
O	-0.991300	-2.061890	3.365250
C	-0.039540	0.172280	3.295180
H	-0.776990	0.743090	3.875170
H	0.719810	-0.187040	4.001800
C	1.819620	0.492940	1.518710
H	0.667380	2.112580	2.514190
H	2.556180	1.268740	1.309330
H	2.310030	-0.313460	2.073830
C	-1.343020	3.385510	0.284230
O	-0.262050	2.830200	-0.157400
C	-1.597370	4.820450	-0.092430
H	-0.670750	5.400060	-0.046330
H	-1.964840	4.857240	-1.125440
H	-2.350130	5.263250	0.563690
S	4.067200	-1.210750	-0.315010
O	4.784890	-1.249100	0.994530
O	4.952440	-0.835560	-1.459420
F	3.851190	-2.939340	-0.619560
O	-2.158680	2.724650	0.977890
C	1.620570	0.468870	-1.111990
O	1.264410	-0.047390	-2.158330
O	2.447470	1.524070	-1.048920
C	3.000600	1.953410	-2.304860
H	3.622270	2.817770	-2.068460
H	3.609570	1.151660	-2.728600
H	2.206970	2.236740	-3.000930
H	0.946690	-1.068870	0.198900

Na_2CO_3 · $(DMA)_4$ (optimized in DMA)

B3LYP-D3(BJ)/6-31G(d) SCF energy in solution (au): -1740.085597
B3LYP-D3(BJ)/6-31G(d) enthalpy in solution (au): -1739.479130
B3LYP-D3(BJ)/6-31G(d) free energy in solution (au): -1739.631249
ωB97X-D/def2-TZVP SCF energy in solution (au): -1740.090003
ωB97X-D/def2-TZVP enthalpy in solution (au): -1739.483536
ωB97X-D/def2-TZVP free energy in solution (au): -1739.635655

Cartesian coordinates

ATOM	X	Y	Z
C	0.778820	1.121720	-0.400630
O	1.115290	0.669300	-1.563600
O	1.019110	2.334020	-0.027720
O	0.183650	0.303510	0.447870
Na	0.974190	-1.445800	-0.641070
Na	-0.259550	1.989860	1.836550
C	3.736020	-0.586800	-0.388320
O	3.207780	-1.692300	-0.127940
N	4.136700	-0.277790	-1.644340
C	3.931520	0.440860	0.702270
H	3.764120	-0.047190	1.663250

H	4.933870	0.879730	0.691530
H	3.197890	1.248400	0.580440
C	3.800450	-1.199380	-2.723280
H	4.506150	-1.056040	-3.545800
H	3.864710	-2.226400	-2.364470
H	2.782170	-1.008950	-3.089860
C	4.364830	1.104510	-2.058610
H	3.400890	1.589630	-2.253180
H	4.903150	1.660530	-1.290830
H	4.971610	1.104730	-2.968020
C	-1.759120	-2.278350	-1.663770
O	-0.725300	-2.804890	-1.191680
N	-2.987050	-2.638850	-1.230130
C	-1.626280	-1.221480	-2.740020
H	-0.739210	-0.618330	-2.517650
H	-1.474010	-1.714890	-3.708440
H	-2.483690	-0.555260	-2.824510
C	-3.126510	-3.592590	-0.135120
H	-3.538230	-4.539790	-0.505090
H	-2.152170	-3.772520	0.313180
H	-3.810430	-3.185290	0.617360
C	-4.246610	-2.163370	-1.794310
H	-4.949770	-3.001500	-1.828690
H	-4.682700	-1.371040	-1.173690
H	-4.113900	-1.796090	-2.810260
C	-0.671700	-0.618320	3.100540
O	-0.883820	0.542490	3.515250
N	0.533660	-1.204430	3.277870
C	-1.761120	-1.392540	2.391890
H	-2.700010	-0.849450	2.515330
H	-1.526910	-1.448830	1.324790
H	-1.878740	-2.408150	2.781060
C	1.597600	-0.421460	3.894240
H	2.050960	0.264320	3.166760
H	1.199930	0.161470	4.725910
H	2.368500	-1.102520	4.263760
C	0.961220	-2.432230	2.618740
H	1.777870	-2.212590	1.919580
H	1.328280	-3.151440	3.360690
H	0.140540	-2.890290	2.069750
C	-1.909190	3.440280	-0.059540
O	-2.034850	3.310070	1.178100
N	-2.345650	2.487810	-0.917400
C	-1.233950	4.665280	-0.632310
H	-1.100650	5.389010	0.174220
H	-0.249040	4.369310	-1.009920
H	-1.802430	5.129520	-1.443690
C	-2.869960	1.234730	-0.374060
H	-2.049960	0.530150	-0.190300
H	-3.385430	1.436850	0.565140
H	-3.576190	0.809870	-1.091780
C	-1.927390	2.442330	-2.316690
H	-1.843700	3.446430	-2.732140
H	-0.962450	1.928900	-2.413160
H	-2.682290	1.899150	-2.891760

$NaHCO_3 \cdot (DMA)_2$ (optimized in DMA)

B3LYP-D3(BJ)/6-31G(d) SCF energy in solution (au): -1002.554199
B3LYP-D3(BJ)/6-31G(d) enthalpy in solution (au): -1002.228573
B3LYP-D3(BJ)/6-31G(d) free energy in solution (au): -1002.320895
ωB97X-D/def2-TZVP SCF energy in solution (au): -1002.581786
ωB97X-D/def2-TZVP enthalpy in solution (au): -1002.256160
ωB97X-D/def2-TZVP free energy in solution (au): -1002.348481

Cartesian coordinates

ATOM	X	Y	Z
C	2.228840	-1.686940	-0.364040
C	-2.835830	-0.410550	0.671110
O	2.248380	-1.937550	0.876530
O	3.450340	-1.632880	-1.007890
O	1.223750	-1.471490	-1.082650
Na	0.038930	-1.288460	0.904400
O	-2.184870	-1.441630	0.952690
C	0.775260	1.464080	0.091120
O	0.111500	1.007130	1.048110
H	4.113630	-1.805610	-0.316120
N	-3.159600	-0.107210	-0.605250
C	-2.801190	-1.008490	-1.693750
H	-2.024630	-0.557750	-2.323250
H	-2.434760	-1.946950	-1.281370
H	-3.684460	-1.200630	-2.313070
C	-3.894790	1.085280	-1.008900
H	-3.905120	1.831400	-0.216820
H	-3.407580	1.523570	-1.886100
H	-4.928040	0.831260	-1.277620
C	-3.264010	0.540020	1.767320
H	-3.030490	0.075940	2.726950
H	-2.706960	1.479600	1.688170
H	-4.333450	0.768010	1.727290
N	2.097690	1.713990	0.219360
C	0.104040	1.726430	-1.238910
H	0.440640	0.983580	-1.969820
H	-0.972160	1.618050	-1.096230
H	0.319180	2.724680	-1.632090
C	2.753940	1.384470	1.483140
H	3.669840	1.975090	1.564950
H	2.090590	1.625620	2.314510
H	3.000250	0.316870	1.527330
C	2.988700	1.969720	-0.906690
H	2.431200	2.306280	-1.778840
H	3.708250	2.747790	-0.632350
H	3.536570	1.055970	-1.169380

NaOAc · $(DMA)_2$ (optimized in DMA)
B3LYP-D3(BJ)/6-31G(d) SCF energy in solution (au): -966.633016
B3LYP-D3(BJ)/6-31G(d) enthalpy in solution (au): -966.284016
B3LYP-D3(BJ)/6-31G(d) free energy in solution (au): -966.382563
ωB97X-D/def2-TZVP SCF energy in solution (au): -966.641329
ωB97X-D/def2-TZVP enthalpy in solution (au): -966.292329
ωB97X-D/def2-TZVP free energy in solution (au): -966.390875

Cartesian coordinates

ATOM	X	Y	Z
C	-0.000020	1.421080	-0.211240

C	2.677540	-1.105090	-0.058080
O	-0.000130	1.447210	1.061370
O	0.000280	0.364310	-0.907870
Na	0.000220	-0.844240	1.090760
O	2.094180	-1.687020	0.884250
C	-2.677370	-1.105130	-0.058120
O	-2.093660	-1.687020	0.884000
N	3.341970	0.055700	0.141240
C	3.298290	0.677760	1.462940
H	4.189600	1.298270	1.588510
H	2.400040	1.298580	1.567200
H	3.284300	-0.097200	2.229680
C	3.800640	0.916090	-0.942560
H	3.058020	1.695960	-1.157910
H	4.736740	1.399190	-0.646760
H	3.981010	0.344490	-1.851330
C	2.635550	-1.686060	-1.453640
H	2.178310	-2.675490	-1.395290
H	2.017650	-1.048070	-2.094510
H	3.629080	-1.774960	-1.903690
N	-3.341940	0.055510	0.141450
C	-2.635490	-1.685830	-1.453790
H	-2.018530	-1.047220	-2.094950
H	-2.177370	-2.674860	-1.395830
H	-3.629180	-1.775540	-1.903350
C	-3.298050	0.677530	1.463170
H	-3.283480	-0.097490	2.229850
H	-2.399980	1.298650	1.567190
H	-4.189550	1.297680	1.589120
C	-3.801630	0.915580	-0.942160
H	-3.982630	0.343700	-1.850650
H	-4.737600	1.398540	-0.645740
H	-3.059370	1.695590	-1.158310
C	-0.000210	2.770250	-0.930540
H	0.880940	3.350930	-0.631780
H	-0.000420	2.649530	-2.017150
H	-0.881120	3.351040	-0.631320

A''_LG2
B3LYP-D3(BJ)/6-31G(d)-SDD(Pd) SCF energy in gas phase (au): -2090.246158
B3LYP-D3(BJ)/6-31G(d)-SDD(Pd) enthalpy in gas phase (au): -2089.926106
B3LYP-D3(BJ)/6-31G(d)-SDD(Pd) free energy in gas phase (au): -2090.020352
ωB97X-D/def2-TZVP SCF energy in solution (au): -2090.395012
ωB97X-D/def2-TZVP enthalpy in solution (au): -2090.074959
ωB97X-D/def2-TZVP free energy in solution (au): -2090.169205

Cartesian coordinates

ATOM	X	Y	Z
Pd	-0.512580	0.034700	-0.254330
C	0.816730	-1.477270	-0.141840
O	-2.117240	-3.707060	0.143080
C	-1.432310	-2.703420	-0.060120
C	0.041160	-2.762600	-0.471260
C	2.015670	-1.223450	-1.054380
N	-1.879550	-1.398830	-0.014230
C	-3.184270	-0.999190	0.164860
C	-4.289390	-1.822630	0.410190

C	-5.576800	-1.273780	0.561270
C	-5.817670	0.084200	0.479960
C	-4.734240	0.961050	0.231630
C	-3.420610	0.423480	0.072090
N	-2.347410	1.238400	-0.176950
C	-2.514500	2.547060	-0.266360
C	-3.776250	3.161220	-0.115670
C	-4.874760	2.368730	0.130200
H	0.495450	-3.662220	-0.035090
H	0.040000	-2.915210	-1.562250
H	-4.123520	-2.888030	0.479830
H	-6.405310	-1.952200	0.752860
H	-6.817270	0.492080	0.604230
H	-1.617640	3.127570	-0.464640
H	-3.862960	4.240460	-0.196680
H	-5.862890	2.807490	0.249970
H	1.148920	-1.493720	0.905690
H	1.661140	-1.109390	-2.091040
C	2.839590	-0.008670	-0.676530
H	2.679850	-2.095610	-1.059830
C	2.225170	1.226560	-0.390120
O	0.986240	1.475070	-0.507920
O	3.024090	2.236810	0.016560
C	2.374240	3.431900	0.435000
H	1.854860	3.914870	-0.399250
H	1.652100	3.230430	1.232210
H	3.174740	4.076070	0.803280
S	4.470870	-0.265130	-0.266860
O	4.907650	-1.602110	-0.689880
O	5.361350	0.880160	-0.466360
Cl	4.480540	-0.462690	2.015920

A''_LG6

B3LYP-D3(BJ)/6-31G(d)-SDD(Pd) SCF energy in gas phase (au): -1092.853115
B3LYP-D3(BJ)/6-31G(d)-SDD(Pd) enthalpy in gas phase (au): -1092.549315
B3LYP-D3(BJ)/6-31G(d)-SDD(Pd) free energy in gas phase (au): -1092.635002
ωB97X-D/def2-TZVP SCF energy in solution (au): -1379.279249
ωB97X-D/def2-TZVP enthalpy in solution (au): -1378.975449
ωB97X-D/def2-TZVP free energy in solution (au): -1379.061135

Cartesian coordinates

ATOM	X	Y	Z
Pd	0.645340	-0.010790	-0.051950
C	-0.610620	-1.558750	-0.347670
O	2.408770	-3.703970	-0.229830
C	1.674000	-2.719450	-0.112990
C	0.157500	-2.827180	0.067570
C	-1.949680	-1.399120	0.373440
N	2.082870	-1.405110	-0.065080
C	3.385380	-0.966670	-0.043390
C	4.542910	-1.753130	-0.104760
C	5.818570	-1.160870	-0.055030
C	5.999230	0.205330	0.052000
C	4.861860	1.046330	0.116600
C	3.558440	0.464630	0.070060
N	2.432870	1.242340	0.133290
C	2.536510	2.556550	0.234290

C	3.785190	3.213100	0.283410
C	4.935800	2.458950	0.225570
H	-0.194160	-3.733440	-0.445470
H	0.000570	-3.003400	1.143620
H	4.424800	-2.823770	-0.191650
H	6.689530	-1.811120	-0.106600
H	6.992300	0.645930	0.085050
H	1.597670	3.102020	0.276810
H	3.821650	4.295260	0.366650
H	5.914860	2.932010	0.262180
H	-0.788500	-1.564200	-1.433210
H	-1.780390	-1.470480	1.464630
C	-2.668330	-0.127740	0.016140
H	-2.592520	-2.255510	0.118150
C	-2.116170	1.115400	-0.086350
O	-0.848880	1.403740	-0.067870
O	-2.958480	2.198100	-0.228380
C	-2.341060	3.418690	-0.592230
H	-1.642770	3.772020	0.175040
H	-1.794980	3.333600	-1.540100
H	-3.159700	4.136400	-0.703080
I	-4.849300	-0.281110	0.114510

TS2_LG2

B3LYP-D3(BJ)/6-31G(d)-SDD(Pd) SCF energy in gas phase (au): -2319.358733
B3LYP-D3(BJ)/6-31G(d)-SDD(Pd) enthalpy in gas phase (au): -2318.968977
B3LYP-D3(BJ)/6-31G(d)-SDD(Pd) free energy in gas phase (au): -2319.082773
ωB97X-D/def2-TZVP SCF energy in solution (au): -2319.501644
ωB97X-D/def2-TZVP enthalpy in solution (au): -2319.111888
ωB97X-D/def2-TZVP free energy in solution (au): -2319.225684
imaginary frequency (cm^{-1}): -211.87

Cartesian coordinates

ATOM	X	Y	Z
Pd	-0.357520	0.876700	0.995890
C	0.861520	0.011350	2.374750
C	1.598970	-0.341300	0.076050
C	-2.692020	0.103920	-0.642700
C	-2.041030	2.219940	-1.379550
C	-2.503230	-0.958300	0.309380
C	-3.686870	0.004010	-1.657600
C	-2.995700	2.195220	-2.419270
H	-1.406390	3.079270	-1.196620
C	-3.332840	-2.076600	0.213290
C	-4.498570	-1.156000	-1.723460
C	-3.807680	1.093410	-2.556000
H	-3.075070	3.044710	-3.090000
C	-4.312550	-2.160740	-0.798610
H	-3.200430	-2.875800	0.927670
H	-5.253190	-1.233940	-2.501490
H	-4.552930	1.042480	-3.346590
H	-4.933530	-3.052460	-0.841200
N	-1.891910	1.210670	-0.539010
N	-1.497200	-0.754290	1.241300
C	-1.085270	-1.671210	2.179480
O	-1.583690	-2.780970	2.373940
C	0.068260	-1.128980	3.026580

H	-0.389370	-0.763550	3.956660
H	0.708090	-1.977220	3.302210
C	2.059260	-0.427630	1.513360
H	1.154920	0.782960	3.089430
H	2.901490	0.254960	1.638900
H	2.400370	-1.439210	1.754700
C	0.530700	3.654350	0.728960
O	0.982190	2.441320	0.828800
C	1.645400	4.689590	0.587880
H	2.270220	4.447700	-0.278510
H	1.223780	5.691260	0.476870
H	2.294010	4.658410	1.470380
S	3.563950	-1.209070	-0.910160
O	3.365030	-2.304280	-1.877040
O	4.651490	-1.361560	0.074610
C	0.752760	-1.378490	-0.587470
O	0.183600	-1.227040	-1.648260
O	0.761400	-2.525690	0.119530
C	0.012400	-3.609430	-0.448920
H	0.558260	-4.014700	-1.305700
H	-0.079810	-4.344940	0.348990
H	-0.973610	-3.264400	-0.762320
H	1.722700	0.583420	-0.472280
O	-0.658110	3.992590	0.736280
Cl	4.160030	0.644090	-2.175010

TS2_LG6

B3LYP-D3(BJ)/6-31G(d)-SDD(Pd) SCF energy in gas phase (au): -1322.007059
B3LYP-D3(BJ)/6-31G(d)-SDD(Pd) enthalpy in gas phase (au): -1321.632117
B3LYP-D3(BJ)/6-31G(d)-SDD(Pd) free energy in gas phase (au): -1321.736353
ωB97X-D/def2-TZVP SCF energy in solution (au): -1608.430418
ωB97X-D/def2-TZVP enthalpy in solution (au): -1608.055476
ωB97X-D/def2-TZVP free energy in solution (au): -1608.159712
imaginary frequency (cm^{-1}): -100.50

Cartesian coordinates

ATOM	X	Y	Z
Pd	-0.554690	1.031380	0.690920
C	0.758050	0.749400	2.219900
C	1.536270	0.078960	0.023170
C	-2.683580	-0.470860	-0.682390
C	-2.312260	1.461260	-1.935150
C	-2.342690	-1.238320	0.487870
C	-3.649190	-0.947000	-1.616100
C	-3.252260	1.064010	-2.909550
H	-1.762970	2.394020	-2.016400
C	-2.999750	-2.455360	0.679660
C	-4.286890	-2.190100	-1.379980
C	-3.913300	-0.132150	-2.745460
H	-3.437350	1.700860	-3.768630
C	-3.956400	-2.910670	-0.251560
H	-2.750250	-3.039230	1.553570
H	-5.021360	-2.556560	-2.092010
H	-4.642330	-0.470600	-3.478140
H	-4.441150	-3.866070	-0.064600
N	-2.045900	0.723630	-0.874350
N	-1.384920	-0.678100	1.315010

C	-0.850450	-1.264750	2.437930
O	-1.171050	-2.360740	2.899570
C	0.177770	-0.354560	3.114850
H	-0.347540	0.104470	3.964190
H	0.961720	-0.995740	3.538130
C	2.020570	0.374360	1.424970
H	0.874760	1.698940	2.747200
H	2.715920	1.214020	1.375680
H	2.549330	-0.486410	1.844420
C	-0.486150	3.810450	0.346300
O	0.309460	2.815920	0.089690
C	0.084420	5.169090	-0.052360
H	0.956610	5.396170	0.571320
H	0.429380	5.145840	-1.091460
H	-0.667320	5.951220	0.076310
C	0.984540	-1.239700	-0.396850
O	0.417110	-1.452970	-1.451320
O	1.149880	-2.170770	0.569660
C	0.595350	-3.459030	0.289160
H	1.229180	-3.986440	-0.431000
H	0.571460	-3.979030	1.246360
H	-0.414490	-3.361980	-0.112890
H	1.545840	0.847020	-0.734510
O	-1.602900	3.711620	0.868860
I	4.072030	-0.567680	-1.059110

TS2'_LG2

B3LYP-D3(BJ)/6-31G(d)-SDD(Pd) SCF energy in gas phase (au): -2319.351339
B3LYP-D3(BJ)/6-31G(d)-SDD(Pd) enthalpy in gas phase (au): -2318.961961
B3LYP-D3(BJ)/6-31G(d)-SDD(Pd) free energy in gas phase (au): -2319.079049
ωB97X-D/def2-TZVP SCF energy in solution (au): -2319.500162
ωB97X-D/def2-TZVP enthalpy in solution (au): -2319.110784
ωB97X-D/def2-TZVP free energy in solution (au): -2319.227872
imaginary frequency (cm^{-1}): -230.62

Cartesian coordinates

ATOM	X	Y	Z
Pd	-0.640360	0.794560	0.754600
C	0.795780	0.749230	2.197400
C	1.641140	-0.032680	0.031630
C	-2.415770	-1.130640	-0.578090
C	-2.597480	0.883340	-1.743280
C	-1.824190	-1.837870	0.527920
C	-3.295070	-1.790390	-1.485750
C	-3.468460	0.306110	-2.691490
H	-2.285900	1.921410	-1.804780
C	-2.153490	-3.183990	0.690750
C	-3.598470	-3.158870	-1.280430
C	-3.813900	-1.019810	-2.556810
H	-3.849620	0.910230	-3.508600
C	-3.032080	-3.820500	-0.210250
H	-1.717160	-3.722160	1.519820
H	-4.265150	-3.666920	-1.971910
H	-4.483140	-1.495710	-3.269900
H	-3.257680	-4.872160	-0.050830
N	-2.103470	0.192950	-0.733980
N	-0.975190	-1.092250	1.328270

C	-0.392650	-1.509800	2.502220
O	-0.506450	-2.623890	3.010130
C	0.381400	-0.364880	3.166650
H	-0.293890	0.049170	3.928130
H	1.239480	-0.794570	3.702700
C	2.077120	0.440510	1.401120
H	0.853760	1.727540	2.679440
H	2.698070	1.329830	1.284600
H	2.685780	-0.333530	1.883730
C	-1.259180	3.532180	0.716550
O	-0.350110	2.771810	0.185090
C	-1.124000	5.003640	0.326080
H	-0.152430	5.387170	0.657300
H	-1.157370	5.107180	-0.764600
H	-1.924840	5.594460	0.776140
S	3.688370	-0.820390	-0.715280
O	4.776930	-0.595180	0.253630
O	3.974330	-0.557580	-2.135970
O	-2.163330	3.150790	1.467620
C	1.461130	0.875330	-1.143340
O	0.822180	0.596300	-2.138630
O	2.175970	2.006020	-0.992980
C	2.006350	2.968370	-2.037050
H	2.690070	3.783120	-1.795330
H	2.255980	2.527130	-3.005300
H	0.972800	3.320950	-2.034150
H	1.187320	-1.009060	-0.077300
Cl	3.213660	-3.078480	-0.574740

TS2'_LG6
B3LYP-D3(BJ)/6-31G(d)-SDD(Pd) SCF energy in gas phase (au): -1322.000612
B3LYP-D3(BJ)/6-31G(d)-SDD(Pd) enthalpy in gas phase (au): -1321.625770
B3LYP-D3(BJ)/6-31G(d)-SDD(Pd) free energy in gas phase (au): -1321.729989
ωB97X-D/def2-TZVP SCF energy in solution (au): -1608.425173
ωB97X-D/def2-TZVP enthalpy in solution (au): -1608.050331
ωB97X-D/def2-TZVP free energy in solution (au): -1608.154550
imaginary frequency (cm^{-1}): -131.26

Cartesian coordinates

ATOM	X	Y	Z
Pd	-0.747790	0.812610	0.702480
C	0.578040	0.793300	2.247650
C	1.449270	-0.099080	0.145050
C	-2.463400	-1.150530	-0.665360
C	-2.458650	0.778160	-1.979240
C	-2.001740	-1.795600	0.537270
C	-3.272990	-1.848950	-1.608080
C	-3.250540	0.157770	-2.968990
H	-2.103820	1.798930	-2.081450
C	-2.393340	-3.116820	0.758400
C	-3.643360	-3.190090	-1.340510
C	-3.656090	-1.143690	-2.777110
H	-3.524840	0.710390	-3.861890
C	-3.206100	-3.789720	-0.177330
H	-2.056320	-3.606880	1.660500
H	-4.258330	-3.726790	-2.057840
H	-4.268220	-1.651110	-3.519250

H	-3.484450	-4.820040	0.031190
N	-2.094140	0.149920	-0.878690
N	-1.204440	-1.022040	1.362810
C	-0.732800	-1.377530	2.604790
O	-0.914390	-2.457690	3.165520
C	0.011200	-0.207840	3.259980
H	-0.722610	0.295260	3.904930
H	0.788900	-0.619180	3.919040
C	1.860210	0.309880	1.543000
H	0.679760	1.802180	2.653110
H	2.610320	1.098980	1.477650
H	2.311910	-0.544970	2.056910
C	-1.320970	3.531810	0.539560
O	-0.386070	2.760060	0.077130
C	-1.170650	4.992880	0.120230
H	-0.218420	5.388190	0.491240
H	-1.147780	5.068040	-0.972930
H	-1.995420	5.591350	0.513430
O	-2.262080	3.163610	1.253420
C	1.505460	0.806670	-1.037550
O	0.952420	0.589190	-2.100870
O	2.226020	1.917660	-0.783040
C	2.197760	2.899120	-1.818000
H	2.855430	3.700860	-1.477770
H	2.561910	2.477840	-2.759350
H	1.177280	3.268600	-1.942090
H	0.986150	-1.056430	-0.031260
I	3.814760	-1.387390	-0.583150

Na_2CO_3 · (DMA)$_6$

B3LYP-D3(BJ)/6-31G(d) SCF energy in gas phase (au): -2315.812007
B3LYP-D3(BJ)/6-31G(d) enthalpy in gas phase (au): -2314.913871
B3LYP-D3(BJ)/6-31G(d) free energy in gas phase (au): -2315.117907
ωB97X-D/def2-TZVP SCF energy in solution (au): -2315.839907
ωB97X-D/def2-TZVP enthalpy in solution (au): -2314.941771
ωB97X-D/def2-TZVP free energy in solution (au): -2315.145807

Cartesian coordinates

ATOM	X	Y	Z
C	0.034760	0.759250	-0.446730
O	0.025510	-0.383020	0.205480
O	1.165980	1.284090	-0.774960
O	-1.094770	1.321320	-0.729940
Na	-2.210140	-0.284050	0.456230
Na	2.278750	-0.561380	0.002490
C	-1.055110	1.289710	2.682050
O	-2.182440	0.756920	2.594510
N	0.030170	0.602540	3.101900
C	-0.867400	2.733310	2.268440
H	-1.794120	3.272710	2.473870
H	-0.035910	3.234420	2.768440
H	-0.699780	2.740110	1.183790
C	-0.102760	-0.828400	3.358730
H	-1.116740	-1.035000	3.699710
H	0.093240	-1.384960	2.435390
H	0.615080	-1.123240	4.129160
C	1.387180	1.102270	2.873440

H	1.473900	1.542570	1.877420
H	1.679730	1.840170	3.631320
H	2.076860	0.258140	2.916010
C	-4.023270	1.530560	-1.252930
O	-4.486810	0.477490	-0.773600
N	-3.982160	2.677060	-0.527930
C	-3.472450	1.577840	-2.660590
H	-3.750190	0.653370	-3.169410
H	-3.847570	2.435380	-3.228710
H	-2.381050	1.643540	-2.587330
C	-4.452060	2.621100	0.854500
H	-5.375320	2.040650	0.893970
H	-3.717850	2.149380	1.518730
H	-4.654580	3.639660	1.196610
C	-3.072070	3.762870	-0.903360
H	-3.328890	4.164540	-1.888670
H	-3.178360	4.569310	-0.174930
H	-2.039270	3.397130	-0.909770
C	4.077670	1.385450	-1.436270
O	4.419510	0.589940	-0.540770
N	3.733900	2.670840	-1.172700
C	4.042210	0.926860	-2.880900
H	4.445400	-0.085400	-2.922600
H	3.008230	0.908790	-3.236630
H	4.622820	1.580570	-3.540260
C	3.547650	3.069410	0.217230
H	2.488900	2.965070	0.477480
H	4.141900	2.419820	0.857030
H	3.866140	4.109830	0.343230
C	2.947710	3.458450	-2.121220
H	1.895870	3.157210	-2.044170
H	3.055750	4.517810	-1.869340
H	3.307830	3.317290	-3.140820
C	-4.463020	-2.297280	0.053840
O	-3.248770	-2.346150	0.319960
N	-5.372700	-1.863430	0.965680
C	-4.958660	-2.716120	-1.314350
H	-4.147390	-3.239490	-1.821320
H	-5.837030	-3.366910	-1.274020
H	-5.210090	-1.814000	-1.881830
C	-4.877970	-1.327360	2.230760
H	-5.649880	-1.455540	2.995450
H	-3.978410	-1.865030	2.526130
H	-4.633130	-0.262440	2.141940
C	-6.706720	-1.418380	0.584550
H	-7.374110	-1.513670	1.446310
H	-6.678210	-0.371710	0.257480
H	-7.107860	-2.028650	-0.224120
C	0.779220	-2.476440	-1.685980
O	2.006910	-2.288330	-1.583000
N	0.045910	-1.827540	-2.625090
C	0.077170	-3.455190	-0.767660
H	-0.495140	-2.898870	-0.019790
H	-0.605960	-4.123810	-1.300170
H	0.844730	-4.044670	-0.263400
C	0.719580	-0.824280	-3.445070
H	0.820720	0.118390	-2.892900

H	1.711040	-1.188380	-3.715040
H	0.128220	-0.661330	-4.351440
C	-1.401710	-1.680050	-2.545740
H	-1.858080	-1.893270	-3.520940
H	-1.827710	-2.345620	-1.798640
H	-1.641500	-0.650870	-2.255800
C	4.710920	-1.134300	1.760690
O	3.486580	-1.330360	1.852120
N	5.491070	-1.856400	0.915090
C	5.374930	-0.064120	2.605140
H	5.689890	0.762880	1.960410
H	4.640220	0.304470	3.321690
H	6.250520	-0.434760	3.147330
C	4.844150	-2.776370	-0.016570
H	3.922390	-3.152520	0.421660
H	4.594490	-2.268340	-0.954900
H	5.523830	-3.609140	-0.222760
C	6.819750	-1.414610	0.515130
H	7.403870	-2.284370	0.199740
H	6.749250	-0.705350	-0.319700
H	7.343550	-0.937890	1.343430

$NaHCO_3$ · $(DMA)_3$

B3LYP-D3(BJ)/6-31G(d) SCF energy in gas phase (au): -1290.409373
B3LYP-D3(BJ)/6-31G(d) enthalpy in gas phase (au): -1289.937858
B3LYP-D3(BJ)/6-31G(d) free energy in gas phase (au): -1290.058571
ωB97X-D/def2-TZVP SCF energy in solution (au): -1290.456267
ωB97X-D/def2-TZVP enthalpy in solution (au): -1289.984752
ωB97X-D/def2-TZVP free energy in solution (au): -1290.105465

Cartesian coordinates

ATOM	X	Y	Z
C	1.921580	1.270330	-0.770920
C	-2.640810	-1.830710	-0.325020
O	1.415060	0.869620	-1.858130
O	2.970600	2.173260	-0.875080
O	1.593280	0.952420	0.393410
Na	-0.022170	-0.545490	-0.730160
O	-1.874650	-1.787460	-1.299730
C	-1.112440	1.748230	0.985510
O	-1.665970	0.746510	0.495480
H	3.087480	2.302800	-1.831620
N	-3.882910	-1.271510	-0.374140
C	-4.218020	-0.440370	-1.522530
H	-3.623990	-0.763950	-2.375030
H	-5.284580	-0.540980	-1.748380
H	-3.991920	0.613390	-1.311260
C	-4.667580	-1.022000	0.825450
H	-4.347140	-0.093220	1.317710
H	-5.721990	-0.929490	0.547990
H	-4.576280	-1.847260	1.531830
C	-2.212760	-2.504730	0.965030
H	-2.183880	-1.770050	1.776340
H	-2.895020	-3.312150	1.252980
H	-1.208320	-2.904910	0.821190
N	-0.921610	2.876570	0.253270
C	-0.637310	1.733030	2.422850

H	-0.974270	2.605410	2.991850
H	-1.022360	0.825930	2.890650
H	0.456170	1.700620	2.427080
C	-1.274820	2.849490	-1.164940
H	-1.504010	3.868730	-1.490150
H	-0.450570	2.447330	-1.766990
H	-2.149680	2.215210	-1.302430
C	0.020070	3.920640	0.641060
H	-0.335780	4.882940	0.259110
H	0.096300	3.991410	1.725000
H	1.018760	3.717950	0.235090
C	2.268260	-1.856710	0.727040
O	1.193850	-2.267450	0.253620
N	3.374490	-1.722100	-0.051670
C	2.369170	-1.492710	2.193590
H	2.438480	-0.404100	2.278850
H	1.453440	-1.830170	2.680850
H	3.232650	-1.949480	2.687450
C	3.248990	-1.989030	-1.483420
H	2.904080	-1.097070	-2.020940
H	4.225290	-2.296300	-1.870530
H	2.525430	-2.789840	-1.632940
C	4.525530	-0.916190	0.336950
H	5.431880	-1.350910	-0.097530
H	4.417030	0.117000	-0.016670
H	4.640420	-0.899920	1.419680

NaOAc · (DMA)$_3$

B3LYP-D3(BJ)/6-31G(d) SCF energy in gas phase (au):	-1254.484914
B3LYP-D3(BJ)/6-31G(d) enthalpy in gas phase (au):	-1253.989968
B3LYP-D3(BJ)/6-31G(d) free energy in gas phase (au):	-1254.115252
ωB97X-D/def2-TZVP SCF energy in solution (au):	-1254.512733
ωB97X-D/def2-TZVP enthalpy in solution (au):	-1254.017786
ωB97X-D/def2-TZVP free energy in solution (au):	-1254.143070

Cartesian coordinates

ATOM	X	Y	Z
C	2.319440	0.359250	0.780070
C	-3.260720	-1.014170	0.211630
O	2.064050	0.565730	-0.443520
O	1.463240	0.008710	1.645730
Na	-0.220720	-0.092990	0.031140
O	-2.264980	-0.618300	0.831850
C	1.070030	-2.740970	-0.110280
O	0.087190	-2.318070	-0.747600
N	-4.314340	-0.180610	-0.007130
C	-4.216880	1.197380	0.469790
H	-4.859100	1.826570	-0.154000
H	-4.549500	1.280260	1.513540
H	-3.184140	1.539080	0.392140
C	-5.618540	-0.604490	-0.485900
H	-6.377030	-0.470560	0.297660
H	-5.919680	-0.009060	-1.356560
H	-5.607210	-1.655120	-0.770970
C	-3.303760	-2.430650	-0.335080
H	-2.273400	-2.790320	-0.358880
H	-3.899060	-3.080850	0.317460

H	-3.724020	-2.483620	-1.343810
N	2.306430	-2.765620	-0.670330
C	0.904210	-3.199720	1.324990
H	1.290620	-2.415030	1.986180
H	-0.164730	-3.306880	1.513650
H	1.410070	-4.145180	1.541940
C	2.482730	-2.195360	-2.004500
H	1.564630	-2.335360	-2.573260
H	2.692730	-1.122820	-1.921260
H	3.310990	-2.710460	-2.501830
C	3.536840	-2.981090	0.073740
H	3.330970	-3.350680	1.076030
H	4.167540	-3.711450	-0.447640
H	4.095490	-2.041170	0.162010
C	3.756020	0.589740	1.263370
H	3.821900	1.581630	1.728510
H	4.029890	-0.143030	2.028230
H	4.465290	0.555840	0.431480
C	-0.077730	2.845720	0.072080
O	-0.981020	2.129490	-0.398810
N	0.874110	3.393680	-0.727290
C	0.886550	3.007100	-2.135500
H	1.341290	3.813700	-2.719430
H	1.466520	2.084890	-2.256170
H	-0.135930	2.838560	-2.469360
C	2.161870	3.857140	-0.229110
H	2.901020	3.053250	-0.328990
H	2.489930	4.725080	-0.811670
H	2.097630	4.149130	0.817770
C	-0.022880	3.093490	1.566830
H	-1.020790	2.903990	1.965830
H	0.660780	2.364780	2.020190
H	0.290940	4.104810	1.837840

Table of Nucleophile Scope

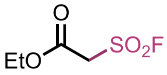
2b

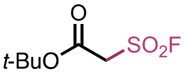
2c

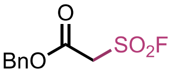
2d

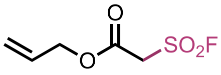
2e

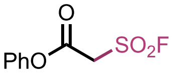
2f

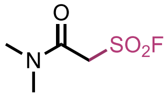
2g

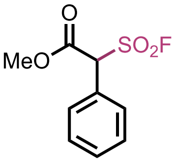
2h

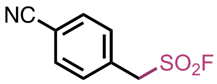
2i

Unsuccessful Nucleophiles

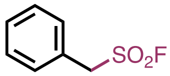
n.d.

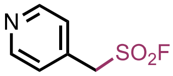
trace

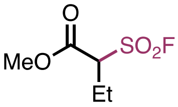
trace

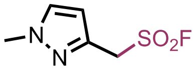
6% (*trans*)

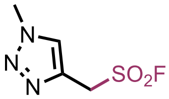
n.d.

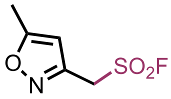
n.d.

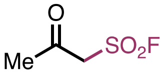
trace

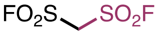
n.r.

Representative Procedures and Analytical Data for Sulfonyl Fluoride Nucleophiles

(yields are not optimized)

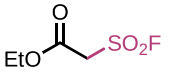

2b

ethyl 2-(fluorosulfonyl)acetate (2b): The reaction was carried out according to General Procedure A1 starting from ethyl 2-bromoacetate (1.7g, 10 mol) and sodium sulfate (1.4 g, 10 mol) to afford crude ethyl 2-(chlorosulfonyl)acetate, which was directly carried forward to General Procedure B. The reaction mixture was purified by silica gel flash column chromatography (10–20% ethyl acetate: hexanes) to afford **2b** as colorless oil. **^{1}H NMR** (500 MHz, $CDCl_3$) δ 4.37–4.31 (m, 4H), 1.35 (t, J = 7.1 Hz, 3H). **^{13}C NMR** (151 MHz, $CDCl_3$) δ 160.07, 63.72, 54.81 (d, $J_{C–F}$ = 20.1 Hz),13.99. **^{19}F NMR** (376 MHz, $CDCl_3$) δ 57.75. **HRMS** (ESI-TOF) Calcd for $C_4H_6FO_4S$ $[M{-}H]^-$ 168.9971, found 168.9970.

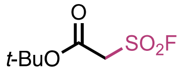

2c

***tert*-butyl 2-(fluorosulfonyl)acetate (2c):** The reaction was carried out according to General Procedure A2 starting from 2-(chlorosulfonyl)acetyl chloride (500 mg, 2.82 mol) and 2-methylpropan-2-ol (266 μL, 10 mol) in anhydrous DCM (10 mL) to afford crude *tert*-butyl 2-(chlorosulfonyl)acetate, which was directly carried forward to General Procedure B. The reaction mixture was purified by silica gel flash column chromatography (10–20% ethyl acetate: hexanes) to afford **2c** as colorless oil. **^{1}H NMR** (600 MHz, $CDCl_3$) δ 4.26 (d, J = 3.3 Hz, 2H), 1.53 (d, J = 0.7 Hz, 9H). **^{13}C NMR** (151 MHz, $CDCl_3$) δ 158.93, 85.75, 56.01 (d, $J_{C–F}$ = 18.8 Hz). **^{19}F NMR** (376 MHz, $CDCl_3$) δ 57.33. HRMS (ESI-TOF) Calcd for $C_6H_{10}FO_4S$ $[M{-}H]^-$ 197.0284, found 197.0282.

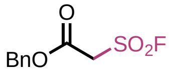

2d

benzyl 2-(fluorosulfonyl)acetate (2d): The reaction was carried out according to General Procedure A1 starting from benzyl 2-bromoacetate (6.9 g, 30 mol) and sodium sulfate (4.3 g, 30 mol) to get benzyl 2-(chlorosulfonyl)acetate. Then, followed by General Procedure B, the reaction mixture was purified by silica gel flash column chromatography (10% – 20% ethyl acetate: hexanes) to afford **2d** as colorless oil. **^{1}H NMR** (600 MHz, $CDCl_3$) δ 7.42–7.34 (m, 5H), 5.29 (s, 2H), 4.37 (d, J = 3.5 Hz, 2H). **^{13}C NMR** (151 MHz, $CDCl_3$) δ 159.98, 134.05, 129.19, 128.95, 128.75, 69.22, 54.73 (d, $J_{C–F}$ = 20.2 Hz). **^{19}F NMR** (376 MHz, CDC13) δ 57.99. **HRMS** (ESI-TOF) Calcd for $C_9H_8FO_4S$ $[M{-}H]^-$ 231.0127, found 231.0125.

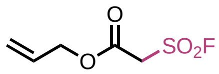

2e

allyl 2-(fluorosulfonyl)acetate (2e): The reaction was carried out according to General Procedure A1 starting from benzyl 2-bromoacetate (4.44 g, 24.8 mol) and sodium sulfate (3.13 g, 24.8 mol) to get allyl 2-(chlorosulfonyl)acetate. Then, followed by General Procedure B, the reaction mixture was purified by silica gel flash column chromatography (10% – 50% ethyl acetate: hexanes) to afford **2e** as colorless oil. **^{1}H NMR** (600 MHz, $CDCl_3$) δ 5.93 (ddt, J = 17.1, 10.4, 5.9 Hz, 1H), 5.46–5.28 (m, 2H), 4.76 (dt, J = 5.9, 1.3 Hz, 2H), 4.37 (d, $J_{C–F}$ = 3.4 Hz, 2H). **^{13}C NMR** (151 MHz, $CDCl_3$) δ 163.40 – 116.17 (m), 67.94, 54.71 (d, J = 20.4 Hz). **^{19}F NMR** (376 MHz, CDC13) δ 57.90. **HRMS** (ESI-TOF) Calcd for $C_5H_6FO_4S$ $[M{-}H]^-$ 180.9971, found 180.9969.

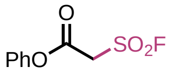

2f

phenyl 2-(fluorosulfonyl)acetate (2f): The reaction was carried out according to General Procedure A1 starting from phenyl 2-bromoacetate (3.3 g, 15 mol) and sodium sulfate (1.81 g, 15 mol) to get phenyl 2-(chlorosulfonyl)acetate. Then, followed by General Procedure B, the reaction mixture was purified by silica gel

flash column chromatography (10% – 30% ethyl acetate: hexanes) to afford **2f** as white solid. **^{1}H NMR** (600 MHz, $CDCl_3$) δ 7.46–7.39 (m, 2H), 7.35–7.29 (m, 1H), 7.20–7.12 (m, 2H), 4.58 (d, *J* = 3.7 Hz, 2H). **^{13}C NMR** (151 MHz, $CDCl_3$) δ 158.68, 149.95, 129.94, 127.20, 121.03, 54.97 (d, $J_{C–F}$ = 20.8 Hz). **^{19}F NMR** (376 MHz, $CDCl_3$) δ 58.52. **HRMS** (ESI-TOF) Calcd for $C_8H_6FO_4S$ $[M-H]^-$ 216.9971, found 216.9973.

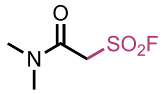

2g

2-(dimethylamino)-2-oxoethane-1-sulfonyl fluoride (2g): The reaction was carried out according to General Procedure B using 2-(dimethylamino)-2-oxoethane-1-sulfonyl chloride (1g, 5.3 mmol), KHF_2 (841.5 mg, 10.8 mmol), and 18-crown-6 (71.2 mg, 0.3 mmol) in 6.7 mL acetonitrile. The reaction was run for 24 h at room temperature, and the product was purified by recrystallization with DCM and hexanes to afford **2g** as a white crystal. **^{1}H NMR** (400 MHz, $CDCl_3$) δ 4.47 (d, *J* = 2.4 Hz, 2H), 3.14 (s, 3H), 3.06 (s, 3H). **^{13}C NMR** (151 MHz, $CDCl_3$) δ 158.81, 54.28 (d, $J_{C–F}$ = 17.4 Hz), 38.42, 36.35. ^{19}F NMR (376 MHz, CDCl3) δ 61.27. **HRMS** (ESI-TOF) Calcd for $C_4H_9FNO_3S$ $[M+H]^-$ 170.0287, found 170.0287.

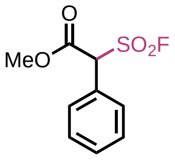

2h

methyl 2-(fluorosulfonyl)-2-phenylacetate (2h): The reaction was carried out according to General Procedure A1 starting from methyl 2-bromo-2-(4-fluorophenyl)acetate (3.6 g, 14.4 mol) and sodium sulfate (2.1 g, 14.4 mol) to get 2-bromo-2-(4-chlorophenyl)acetate. Then, followed by General Procedure B, the reaction mixture was purified by silica gel flash column chromatography (10% – 30% ethyl acetate: hexanes) to afford **2h** as light brown solid. **^{1}H NMR** (600 MHz, $CDCl_3$) δ 7.6 –7.55 (m, 2H), 7.52–7.44 (m, 3H), 5.41 (s, 1H), 3.89 (s, 3H). **^{13}C NMR** (151 MHz, $CDCl_3$) δ 163.39 (d, *J* = 2.2 Hz), 130.94, 130.11, 129.61, 126.13, 71.06 (d, $J_{C–F}$ = 17.2 Hz), 54.25. **^{19}F NMR** (376 MHz, CDCl3) δ 48.47. **HRMS** (ESI-TOF) Calcd for $C_9H_8FO_4S$ $[M-H]^-$ 231.0127, found 231.0128.

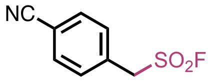

2i

(4-cyanophenyl)methanesulfonyl fluoride (2i): The reaction was carried out according to General Procedure B using (4-cyanophenyl)methanesulfonyl fluoride (500 mg), KHF_2 (841.5 mg, 10.8 mmol), and 18-crown-6 (71.2 mg, 0.3 mmol) in 6.7 mL acetonitrile. The reaction was run for 24 h at room temperature, and the crude was purified by recrystallization with DCM and hexanes to afford 329 mg (71%) of **2i** as a white crystal. **^{1}H NMR** (400 MHz, $CDCl_3$) δ 7.76 (d, *J* = 8.4 Hz, 1H), 7.58 (d, *J* = 8.2 Hz, 2H), 4.65 (d, *J* = 3.1 Hz, 2H). **^{13}C NMR** (151 MHz, $CDCl_3$) δ 133.18, 131.59, 130.68, 117.90, 114.35, 56.40 (d, $J_{C–F}$ = 19.1 Hz).**^{19}F NMR** (376 MHz, $CDCl_3$) δ 53.03. **HRMS** (ESI-TOF) Calcd for $C_8H_5FNO_2S$ $[M-H]^-$ 198.0025, found 198.0024.

Representative Procedures and Analytical Data for Nucleophile Scope

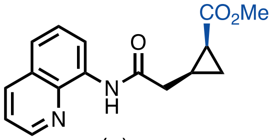

(±)-3aa

(±)-**methyl 2-(2-oxo-2-(quinolin-8-ylamino)ethyl)cyclopropane-1-carboxylate (3aa):** The reaction was carried out according to General Procedure C with **1a** (21.2 mg, 0.1 mmol), methyl 2-(fluorosulfonyl)acetate **2a** (46.8 mg, 0.3 mmol), Na_2CO_3 (10.6 mg, 0.1 mmol), $Pd(OAc)_2$ (2.3 mg, 0.01 mmol), and anhydrous DMA (0.3 mL). The reaction was run for 14 h at 80 °C, and the crude residue was purified by silica gel flash column chromatography (10–20% ethyl acetate: hexanes) to afford 26.6 mg (94%) of **(±)-3aa** as a white solid. **^{1}H NMR** (600 MHz, $CDCl_3$) δ 9.91 (s, 1H), 8.83–8.76 (m, 2H), 8.15 (dd, *J* = 8.2, 1.7 Hz, 1H), 7.56–7.48 (m, 1H), 7.45 (dd, *J* = 8.2, 4.2 Hz, 1H), 3.68 (s, 3H), 2.95–2.78 (m, 2H), 1.93 (td, *J* = 8.4, 5.5 Hz, 1H), 1.85–1.77 (m, 1H), 1.29 (td, *J* = 8.3, 4.7 Hz, 1H), 1.14 (dt, *J* = 7.1, 5.1 Hz, 1H). **^{13}C NMR** (151 MHz, $CDCl_3$) δ 173.75, 170.77, 148.29, 138.52, 136.42, 134.68, 128.05, 127.53, 121.73, 121.59, 116.58, 51.98, 35.88, 17.73, 17.71, 13.94. **HRMS** (ESI-TOF) Calcd for $C_{16}H_{17}N_2O_3^+$ $[M+H]^+$ 285.1239, found 285.1249. **X-ray** (single-crystal) Single crystals of **3aa** suitable for X-ray diffraction were obtained from liquid/liquid diffusion of hexanes into a saturated solution of **3aa** in DCM (CCDC 2171740).[28]

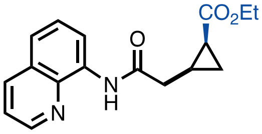

(±)-3ab

(±)-**ethyl 2-(2-oxo-2-(quinolin-8-ylamino)ethyl)cyclopropane-1-carboxylate (3ab):** The reaction was carried out according to General Procedure C with **1a** (21.2 mg, 0.1 mmol), ethyl 2-(fluorosulfonyl)acetate (51.1 mg, 0.3 mmol), Na_2CO_3 (10.6 mg, 0.1 mmol), $Pd(OAc)_2$ (2.3 mg, 0.01 mmol), and anhydrous DMA (0.3 mL). The reaction was run for 14 h at 80 °C, and the crude residue was purified by silica gel flash column chromatography (10–20% ethyl acetate: hexanes) to afford 22.9 mg (77%) of **(±)-3ab** as a white solid. **^{1}H NMR** (600 MHz, $CDCl_3$) δ 9.95 (s, 1H), 8.94–8.71 (m, 2H), 8.16 (dd, *J* = 8.3, 1.7 Hz, 1H), 7.57–7.49 (m, 2H), 7.46 (dd, *J* = 8.2, 4.2 Hz, 1H), 4.14 (td, *J* = 7.1, 3.1 Hz, 2H), 2.98–2.86 (m, 2H), 1.93 (td, *J* = 8.3, 5.5 Hz, 1H), 1.82 (tq, *J* = 8.6, 7.2 Hz, 1H), 1.29 (ddd, *J* = 11.5, 8.1, 3.9 Hz, 1H), 1.20 (t, *J* = 7.1 Hz, 3H), 1.15 (dt, *J* = 7.1, 5.1 Hz, 1H). **^{13}C NMR** (151 MHz, $CDCl_3$) δ 173.19, 170.78, 148.25, 138.49, 136.38, 134.66, 128.01, 127.49, 121.68, 121.54, 116.52, 60.72, 35.87, 17.93, 17.56, 14.32, 13.77. **HRMS** (ESI-TOF) Calcd for $C_{17}H_{19}N_2O_3^+$ $[M+H]^+$ 299.1396, found 299.1392.

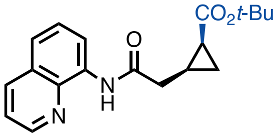

(±)-3ac

(±)-***tert*-butyl 2-(2-oxo-2-(quinolin-8-ylamino)ethyl)cyclopropane-1-carboxylate (3ac):** The reaction was carried out according to General Procedure C with **1a** (21.2mg, 0.1 mmol), *tert*-butyl 2-(fluorosulfonyl)acetate (59.5 mg, 0.3 mmol), Na_2CO_3 (10.6 mg, 0.1 mmol), $Pd(OAc)_2$ (2.3 mg, 0.01 mmol), and anhydrous DMA (0.3 mL). The reaction was run for 14 h at 80 °C, and the crude residue was purified by silica gel flash column chromatography (10–20% ethyl acetate: hexanes) to afford 26.0 mg (80%) of **(±)-3ac** as a colorless oil. **^{1}H NMR** (600 MHz, $CDCl_3$) δ 10.00 (s, 1H), 8.92–8.54 (m, 2H), 8.27–7.87 (m, 1H), 7.64–7.47 (m, 2H), 7.44 (dd, *J* = 8.3, 4.2 Hz, 1H), 2.89 (qd, *J* = 16.1, 7.2 Hz, 2H), 1.87 (td, *J* = 8.3, 5.6 Hz, 1H), 1.75 (q, *J* = 7.9 Hz, 1H), 1.38 (s, 9H), 1.21 (td, *J* = 8.2, 4.7 Hz, 1H), 1.08 (q, *J* = 5.7 Hz, 1H). **^{13}C NMR** (151 MHz, $CDCl_3$) δ 172.25, 170.92, 148.27, 138.54, 136.36, 134.72, 128.04, 127.52, 121.68, 121.51, 116.49, 80.79, 35.87, 28.23, 19.00, 17.09, 13.29. **HRMS** (ESI-TOF) Calcd for $C_{19}H_{23}N_2O_3^+$ $[M+H]^+$ 327.1709, found 327.1703.

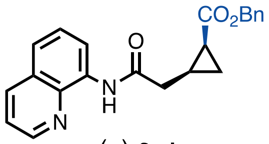

(±)-3ad

(±)-**benzyl 2-(2-oxo-2-(quinolin-8-ylamino)ethyl)cyclopropane-1-carboxylate (3ad):** The reaction was carried out according to General Procedure C using **1a** (21.2mg, 0.1 mmol), benzyl 2-(fluorosulfonyl)acetate (69.7 mg, 0.3 mmol), Na_2CO_3 (10.6 mg, 0.1 mmol), $Pd(OAc)_2$ (2.3 mg, 0.01 mmol), and anhydrous DMA (0.3 mL). The reaction was run for 24 h at 80 °C, and the crude residue was purified by silica gel flash column chromatography (10% – 20% ethyl acetate: hexanes) to afford 16.2 mg (45%) of **(±)-3ad** as a colorless oil. **^{1}H NMR** (600 MHz, $CDCl_3$) δ 9.92 (s, 1H), 8.94 – 8.63 (m, 2H), 8.14 (dd, J = 8.2, 1.7 Hz, 1H), 7.72–7.48 (m, 2H), 7.42 (dd, J = 8.2, 4.2 Hz, 1H), 7.32–7.14 (m, 5H), 5.11 (d, J = 3.2 Hz, 2H), 3.02–2.77 (m, 2H), 1.98 (td, J = 8.3, 5.5 Hz, 1H), 1.84 (q, J = 7.8 Hz, 1H), 1.30 (dt, J = 8.3, 4.1 Hz, 1H), 1.17 (dt, J = 7.2, 5.1 Hz, 1H). **^{13}C NMR** (151 MHz, $CDCl_3$) δ 173.12, 170.70, 148.29, 128.54, 128.24, 128.18, 127.52, 121.71, 121.59, 116.60, 66.62, 35.89, 17.94, 17.90, 14.08. **HRMS** (ESI-TOF) Calcd for $C_{20}H_{22}N_2O_3^+$ $[M+H]^+$ 361.1552, found 361.1559.

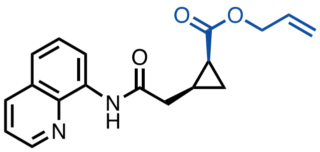

(±)-3ae

(±)-**allyl 2-(2-oxo-2-(quinolin-8-ylamino)ethyl)cyclopropane-1-carboxylate (3ae):** The reaction was carried out according to General Procedure C using **1a** (21.2mg, 0.1 mmol), allyl 2-(fluorosulfonyl)acetate (54.7 mg, 0.3 mmol), Na_2CO_3 (10.6 mg, 0.1 mmol), $Pd(OAc)_2$ (2.3 mg, 0.01 mmol), and anhydrous DMA (0.3 mL). The reaction was run for 14 h at 80 °C, and the crude residue was purified by silica gel flash column chromatography (10% – 20% ethyl acetate: hexanes) to afford 27 mg (87%) of **(±)-3ae** as a colorless oil. **^{1}H NMR** (600 MHz, $CDCl_3$) δ 9.92 (s, 1H), 8.92–8.70 (m, 2H), 8.15 (dd, J = 8.2, 1.7 Hz, 1H), 7.62–7.48 (m, 2H), 7.44 (dd, J = 8.3, 4.2 Hz, 1H), 5.84 (ddt, J = 17.3, 10.4, 5.8 Hz, 1H), 5.23 (dq, J = 17.2, 1.5 Hz, 1H), 5.10 (dq, J = 10.4, 1.3 Hz, 1H), 4.66–4.53 (m, 2H), 2.90 (qd, J = 15.9, 7.2 Hz, 2H), 1.96 (td, J = 8.4, 5.5 Hz, 1H), 1.84 (ddt, J = 15.8, 8.6, 7.2 Hz, 1H), 1.30 (td, J = 8.3, 4.7 Hz, 1H), 1.15 (dt, J = 7.1, 5.2 Hz, 1H). **^{13}C NMR** (151 MHz, $CDCl_3$) δ 172.93, 170.69, 148.27, 138.49, 136.39, 134.65, 132.31, 128.01, 127.49, 121.69, 121.56, 118.26, 116.54, 65.49, 35.85, 17.83, 17.79, 14.01. **HRMS** (ESI-TOF) Calcd for $C_{18}H_{19}N_2O_3^+$ $[M+H]^+$ 311.1396, found 311.1043.

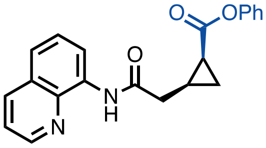

(±)-3af

(±)-**phenyl 2-(2-oxo-2-(quinolin-8-ylamino)ethyl)cyclopropane-1-carboxylate (3af):** The reaction was carried out according to General Procedure C using **1a** (21.2mg, 0.1 mmol), phenyl 2-(fluorosulfonyl)acetate (118 mg, 0.5 mmol), Na_2CO_3 (10.6 mg, 0.1 mmol), $Pd(OAc)_2$ (4.5 mg, 0.02 mmol), and anhydrous DMA (0.3 mL). The reaction was run for 24 h at 80 °C, and the crude residue was purified by silica gel flash column chromatography (15% – 35% ethyl acetate: hexanes) to afford 12.9 mg of **(±)-3af** from the mixture of the two diastereomers (17.3 mg, 50%, (±)-**3af/3af'**=2.9:1 *d.r.*) as a yellow oil. The reported *d.r.* was determined by ^{1}H NMR analysis of purified (±)-**3af/3af'**. The following analytical data correspond to the major diastereomer, as drawn. **^{1}H NMR** (600 MHz, $CDCl_3$) δ 9.96 (s, 1H), 8.93–8.80 (m, 1H), 8.76 (dd, J = 4.2, 1.7 Hz, 1H), 8.18 (dd, J = 8.2, 1.7 Hz, 1H), 7.68–7.50 (m, 2H), 7.46 (dd, J = 8.3, 4.2 Hz, 1H), 7.27 (dd, J = 13.9, 6.2 Hz, 2H), 7.17 (t, J = 7.4 Hz, 1H), 7.11–7.00 (m, 2H), 3.03 (dd, J = 15.9, 6.6 Hz, 1H), 2.96 (dd, J = 15.9, 8.0 Hz, 1H), 2.21 (td, J = 8.3, 5.5 Hz, 1H), 2.00 (h, J = 7.8 Hz, 1H), 1.45 (td, J = 8.3, 4.8 Hz, 1H), 1.28 (dt, J = 7.3, 5.1 Hz, 1H). **^{13}C NMR** (151 MHz, $CDCl_3$) δ 172.04, 170.55, 150.91, 148.33, 138.47, 136.38, 134.63, 129.36, 128.04, 127.51, 125.77, 121.82,

121.72, 121.65, 116.56, 35.75, 18.57, 17.94, 14.60. **HRMS** (ESI-TOF) Calcd for $C_{18}H_{19}N_2O_3^+$ $[M+H]^+$ 347.1396, found 347.1393.

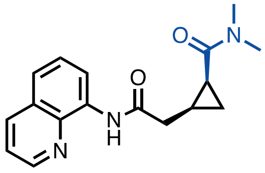

(±)-**3ag**

(±) *N*,*N*-dimethyl-2-(2-oxo-2-(quinolin-8-ylamino)ethyl)cyclopropane-1-carboxamide (3ag): The reaction was carried out according to General Procedure C using **1a** (21.2mg, 0.1 mmol), 2-(dimethylamino)-2-oxoethane-1-sulfonyl fluoride (50.8 mg, 0.3 mmol), Na_2CO_3 (10.6 mg, 0.1 mmol), $Pd(OAc)_2$ (2.3 mg, 0.01 mmol), and anhydrous DMA (0.3 mL). The reaction was run for 24 h at 80°C, and the crude residue was purified by PTLC (100% ethyl acetate: hexanes) to afford 5.2 mg (17%) of **(±)-3ag** as a yellow oil. **^{1}H NMR** (600 MHz, $CDCl_3$) δ 9.85 (s, 1H), 8.94–8.73 (m, 2H), 8.15 (dd, *J* = 8.2, 1.7 Hz, 1H), 7.60–7.49 (m, 2H), 7.45 (dd, *J* = 8.3, 4.2 Hz, 1H), 2.81 (dd, *J* = 15.6, 6.3 Hz, 1H), 2.63 (dd, *J* = 15.7, 8.1 Hz, 1H), 1.99 (td, *J* = 8.2, 5.2 Hz, 1H), 1.84–1.76 (m, 1H), 1.28 (dt, *J* = 6.3, 5.0 Hz, 1H), 1.10 (td, *J* = 8.2, 4.6 Hz, 1H). **^{13}C NMR** (151 MHz, $CDCl_3$) δ 171.29, 171.04, 148.35, 136.40, 128.05, 127.48, 121.77, 121.60, 116.48, 37.56, 36.46, 35.86, 29.84, 17.64, 16.24, 11.30. **HRMS** (ESI-TOF) Calcd for $C_{17}H_{20}N_3O^+$ $[M+H]^+$ 298.1556, found 298.1555.

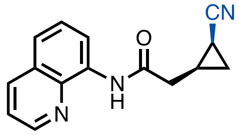

(±)-**3ah**

(±)**2-((2-cyanocyclopropyl)-*N*-(quinolin-8-yl)acetamide (3ah):** The reaction was carried out according to General Procedure C using **1a** (21.2mg, 0.1 mmol), cyanomethanesulfonyl fluoride (61.6 mg, 0.5 mmol), Na_2CO_3 (10.6 mg, 0.1 mmol), $Pd(OAc)_2$ (4.5 mg, 0.02 mmol), and anhydrous DMA (0.3 mL). The reaction was run for 24 h at 80 °C, and the crude residue was purified by silica gel flash column chromatography (10% – 20% ethyl acetate: hexanes) to afford 6.4 mg (23%) of **(±)-3ah** as a yellow oil. **^{1}H NMR** (600 MHz, CDC13) δ 9.98 (s, 1H), 8.87–8.71 (m, 2H), 8.23–8.11 (m, 1H), 7.59–7.49 (m, 2H), 7.47–7.43 (m, 1H), 2.95 (dd, *J* = 16.1, 6.6 Hz, 1H), 2.73 (dd, *J* = 16.0, 7.6 Hz, 1H), 1.87 (h, *J* = 7.5 Hz, 1H), 1.68 (td, *J* = 8.4, 5.4 Hz, 1H), 1.40 (td, *J* = 8.5, 5.4 Hz, 1H), 1.06 (q, *J* = 5.8 Hz, 1H). **^{13}C NMR** (151 MHz, $CDCl_3$) δ 168.87, 148.45, 138.45, 136.51, 134.34, 128.07, 127.48, 121.95, 121.86, 120.26, 116.68, 38.79, 14.81, 13.72, 2.83. **HRMS** (ESI-TOF) Calcd for $C_{15}H_{14}N_3O^+$ $[M+H]^+$ 252.1137, found 252.1136.

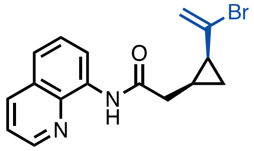

(±)-**3ai**

(±)**2-(2-(1-bromovinyl)cyclopropyl)-*N*-(quinolin-8-yl)acetamide (3ai):** The reaction was carried out according to General Procedure C using **1a** (21.2mg, 0.1 mmol), 2-bromoprop-2-ene-1-sulfonyl fluoride (107 mg, 0.5 mmol), Na_2CO_3 (10.6 mg, 0.1 mmol), $Pd(OAc)_2$ (4.5 mg, 0.02 mmol), and anhydrous DMA (0.3 mL). The reaction was run for 24 h at 80 °C, and the crude residue was purified by silica gel flash column chromatography (10% – 20% ethyl acetate: hexanes) to afford an inseparable mixture of **(±)-3ai** and (*E*)-*N*-(quinolin-8-yl)but-2-enamide (23%, 7.5 mg, 100:16 *ratio*), and pure **(±)-3ai'** (12%, 4.0 mg). The reported *d.r.* was determined by ^{1}H NMR analysis of purified **(±)-3ai/3ai'**. The corresponding analytical data corresponding to the major product, as drawn. **^{1}H NMR** (600 MHz, $CDCl_3$) δ 10.06 (s, 1H), 8.85–8.74 (m, 2H), 8.16 (dd, *J* = 8.2, 1.7 Hz, 1H), 7.55–7.49 (m, 2H), 7.46 (dd, *J* = 8.2, 4.2 Hz, 1H), 5.38 (s, 1H), 5.22 (t, *J* = 1.5 Hz, 1H), 2.86 (dd, *J* = 16.2, 5.8 Hz, 1H), 2.38 (dd, J = 16.2, 8.9 Hz, 1H), 2.01 (q, *J* = 7.8 Hz, 1H), 1.65 (dq, *J* = 9.2, 6.1 Hz, 1H), 1.23 (td, *J* = 8.3 Hz, 1H), 0.84 (q, *J* = 5.8 Hz, 1H). **^{13}C NMR** (151 MHz, $CDCl_3$) δ 170.92, 148.31, 140.94, 138.56, 136.49, 134.63, 128.08, 127.58, 121.73, 121.62, 116.59, 114.12, 36.76, 24.25, 14.91, 12.18. **HRMS** (ESI-TOF) Calcd for $C_{16}H_{16}BrN_2O^+$ $[M+H]^+$ 331.0446, found 331.0450.

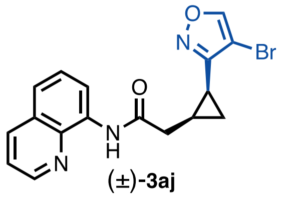

(±)-**3aj**

(±)**2-(2-(4-bromoisoxazol-3-yl)cyclopropyl)-*N*-(quinolin-8-yl)acetamide (3aj):** The reaction was carried out according to General Procedure C using **1a** (21.2mg, 0.1 mmol), (4-bromoisoxazol-3-yl)methanesulfonyl fluoride (77.1 mg, 0.3 mmol), Na_2CO_3 (10.6 mg, 0.1 mmol), $Pd(OAc)_2$ (2.2 mg, 0.01 mmol), and anhydrous DMA (0.3 mL). The reaction was run for 24 h at 80 °C, and the crude residue was purified by silica gel flash column chromatography (5% – 15% ethyl acetate: hexanes) to afford 15.9 mg (43%) of **(±)-3aj** as white solid. **^{1}H NMR** (600 MHz, $CDCl_3$) δ 9.82 (s, 1H), 8.83–8.73 (m, 2H), 8.30 (s, 1H), 8.15 (dd, J = 8.2, 1.7 Hz, 1H), 7.54–7.48 (m, 2H), 7.44 (dd, J = 8.3, 4.1 Hz, 1H), 2.65 (dd, J = 15.9, 6.9 Hz, 1H), 2.55 (dd, J = 15.9, 7.6 Hz, 1H), 2.19 (td, J = 8.5, 5.9 Hz, 1H), 2.01–1.92 (m, 1H), 1.45 (td, J = 8.4, 5.1 Hz, 1H), 1.31 (q, J = 5.9 Hz, 1H). **^{13}C NMR** (151 MHz, $CDCl_3$) δ 170.47, 161.24, 156.88, 148.25, 138.51, 136.42, 134.59, 128.02, 127.53, 121.72, 121.59, 116.57, 96.54, 36.87, 16.63, 11.81, 11.03. **HRMS** (ESI-TOF) Calcd for $C_{17}H_{15}BrN_3O_2^+$ $[M+H]^+$ 372.0348, found 372.0345. **X-ray** (single-crystal) Single crystals for X-ray crystallography were obtained from liquid diffusion of saturated DCM in hexanes (CCDC 2345363).[28]

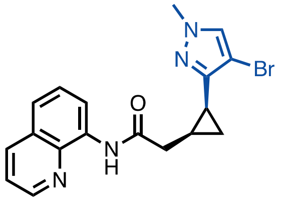

(±)-**3ak**

(±)**2-(2-(4-bromo-1-methyl-1H-pyrazol-3-yl)cyclopropyl)-N-(quinolin-8-yl)acetamide (3ak):** The reaction was carried out according to General Procedure C using **1a** (21.2mg, 0.1 mmol), ((4-bromo-1-methyl-1H-pyrazol-3-yl)methanesulfonyl fluoride (135 mg, 0.5 mmol), Na_2CO_3 (10.6 mg, 0.1 mmol), $Pd(OAc)_2$ (4.5 mg, 0.02 mmol), and anhydrous DMA (0.3 mL). The reaction was run for 24 h at 80 °C, and the crude residue was purified by silica gel flash column chromatography (30% – 50% ethyl acetate: hexanes) to afford **(±)-3ak** from two inseparable diastereomeric mixtures (5.7 mg, 15%, **(±)-3ak/3ak'**=85:15 and 1.9 mg, 5%, **(±)-3ak/3ak'**=50:50). The reported *d.r.* was determined by ^{1}H NMR analysis of purified **(±)-3ak/3ak'**. The following analytical data correspond to the major diastereomer, as drawn. **^{1}H NMR** (600 MHz, $CDCl_3$) δ 9.99 (s, 1H), 8.89–8.66 (m, 2H), 8.15 (d, J = 8.2 Hz, 1H), 7.57–7.47 (m, 2H), 7.46–7.41 (m, 1H), 3.84 (d, J = 1.7 Hz, 3H), 2.70 (dd, J = 16.1, 6.4 Hz, 1H), 2.38 (dd, J = 16.1, 8.2 Hz, 1H), 2.21 (t, J = 7.9 Hz, 1H), 1.79–1.70 (m, 1H), 1.36 (tt, J = 8.6, 3.4 Hz, 1H), 1.19 (q, J = 5.9 Hz, 1H). **^{13}C NMR** (151 MHz, $CDCl_3$) δ 171.62, 148.74, 148.18, 138.65, 136.44, 134.81, 130.85, 128.06, 127.59, 121.63, 121.46, 116.62, 94.98, 39.60, 37.28, 15.30, 12.63, 10.90. **HRMS** (ESI-TOF) Calcd for $C_{17}H_{15}BrN_3O_2^+$ $[M+H]^+$ 385.0664, found 385.0667.

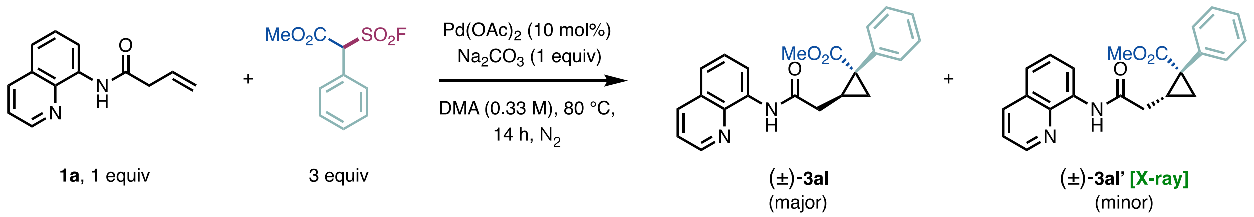

(±)**methyl 2-(2-oxo-2-(quinolin-8-ylamino)ethyl)-1-phenylcyclopropane-1-carboxylate (3al):** The reaction was carried out according to General Procedure C using **1a** (21.2mg, 0.1 mmol), methyl 2-(fluorosulfonyl)-2-phenylacetate (69.7 mg, 0.3 mmol), Na_2CO_3 (10.6 mg, 0.1 mmol), $Pd(OAc)_2$ (2.2 mg, 0.01 mmol), and anhydrous DMA (0.3 mL). The reaction was run for 14 h at 80 °C, and the crude residue was purified by silica gel flash column chromatography (5% – 20% ethyl acetate: hexanes) to afford two pure diastereomers **(±)-3al** (14.2 mg, 40%) as yellow oil and

(±)-3al' (8.7 mg, 24%) as the white solid, respectively. The reported *d.r.* was determined by ^{1}H NMR analysis of purified **(±)-3al/3al'**. The following analytical data correspond to major diastereomer **(±)-3al**. **^{1}H NMR** (600 MHz, $CDCl_3$) δ 9.83 (s, 1H), 8.76 (dt, *J* = 5.5, 1.6 Hz, 2H), 8.15 (dd, *J* = 8.2, 1.7 Hz, 1H), 7.55–7.48 (m, 2H), 7.44 (dd, *J* = 8.3, 4.2 Hz, 1H), 7.36–7.26 (m, 5H), 3.66 (s, 3H), 2.49–2.41 (m, 2H), 2.08–2.01 (m, 1H), 1.99–1.94 (m, 1H), 1.44–1.38 (m, 1H). **^{13}C NMR** (151 MHz, $CDCl_3$) δ 174.48, 169.90, 148.27, 138.47, 136.48, 135.56, 134.46, 131.44, 128.49, 128.06, 127.69, 127.54, 121.75, 121.70, 116.62, 52.68, 38.89, 33.66, 24.13, 20.93. **HRMS** (ESI-TOF) Calcd for $C_{22}H_{21}N_2O_3^+$ [M+H]$^+$ 361.1552, found 361.1553.

The following analytical data correspond to minor diastereomer **(±)-3al'**. **^{1}H NMR** (600 MHz, $CDCl_3$) δ 9.89 (s, 1H), 8.85–8.79 (m, 2H), 8.16 (dd, *J* = 8.2, 1.7 Hz, 1H), 7.60–7.39 (m, 5H), 7.35–7.30 (m, 2H), 7.26 (s, 1H), 3.58 (s, 3H), 3.08–2.97 (m, 2H), 2.14 (tt, *J* = 8.7, 7.0 Hz, 1H), 1.75 (dd, *J* = 7.3, 4.6 Hz, 1H), 1.52 (dd, *J* = 8.9, 4.6 Hz, 1H). **^{13}C NMR** (151 MHz, $CDCl_3$) δ 173.55, 170.63, 148.30, 140.50, 138.51, 136.46, 134.68, 130.68, 128.32, 128.09, 127.56, 127.32, 121.76, 121.62, 116.68, 52.68, 36.43, 34.10, 25.57, 21.14. **HRMS** (ESI-TOF) Calcd for $C_{22}H_{21}N_2O_3^+$ [M+H]$^+$ 361.1552, found 361.1552. **X-ray** (single-crystal) Single crystals for X-ray crystallography were obtained from liquid diffusion of saturated DCM in hexanes (CCDC 2351469).[28]

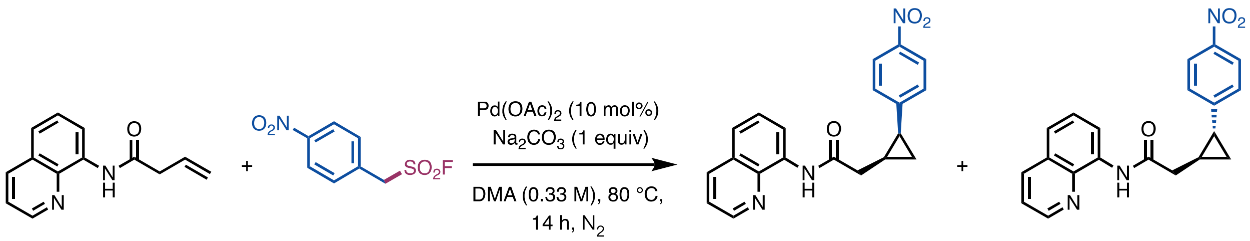

1a, 1 equiv 3 equiv (±)-**3am** (±)-**3am'**

(±)2-(2-(4-nitrophenyl)cyclopropyl)-*N*-(quinolin-8-yl)acetamide (3am): The reaction was carried out according to General Procedure C using **1a** (21.2mg, 0.1 mmol), (4-nitrophenyl)methanesulfonyl fluoride (69.2 mg, 0.3 mmol), Na_2CO_3 (10.6 mg, 0.1 mmol), $Pd(OAc)_2$ (2.2 mg, 0.01 mmol), and anhydrous DMA (0.3 mL). The reaction was run for 14 h at 80 °C, and the crude residue was purified by silica gel flash column chromatography (5% – 20% ethyl acetate: hexanes) to afford a mixture of the two diastereomers of **(±)-3am** and **(±)-3am'** from two inseparable mixtures (14.4 mg, 42%, **(±)-3am**:**(±)-3am'** = 91:9 and 10.7 mg, 31% **(±)-3am**:**(±)-3am'**=20:80) as white solids. The following analytical data correspond to major diastereomer **(±)-3am**, as drawn. **^{1}H NMR** (600 MHz, $CDCl_3$) δ 9.65 (s, 1H), 8.71 (ddd, *J* = 8.9, 5.6, 1.8 Hz, 2H), 8.16–8.13 (m, 1H), 8.10–8.04 (m, 2H), 7.54–7.48 (m, 2H), 7.44 (dd, J = 8.2, 4.2 Hz, 1H), 7.41 (d, *J* = 8.6 Hz, 2H), 2.47 (td, *J* = 8.6, 6.1 Hz, 1H), 2.41 (dd, *J* = 16.0, 6.9 Hz, 1H), 2.24–2.15 (m, 1H), 1.85 (dtd, *J* = 14.6, 8.4, 6.6 Hz, 1H), 1.40 (td, *J* = 8.3, 5.7 Hz, 1H), 1.10 (q, *J* = 5.9 Hz, 1H). **^{13}C NMR** (151 MHz, $CDCl_3$) δ 170.28, 148.26, 146.75, 138.30, 136.52, 134.28, 129.83, 128.04, 127.48, 123.53, 121.80, 121.77, 116.48, 37.35, 21.17, 16.45, 10.62. **HRMS** (ESI-TOF) Calcd for $C_{20}H_{18}N_3O_3^+$ [M+H]$^+$ 348.1348, found 348.1353.

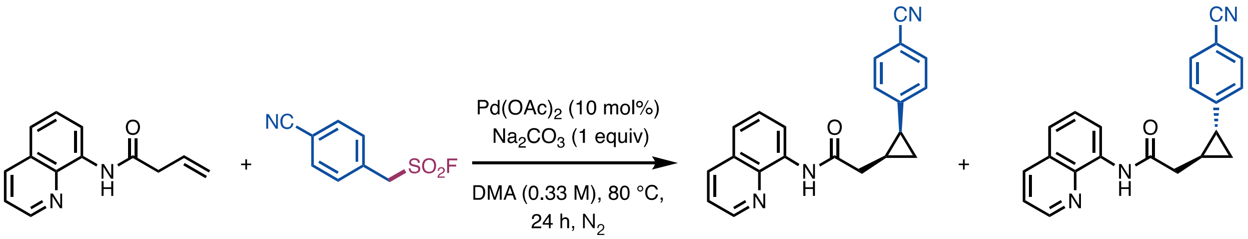

1a, 1 equiv **2i**, 3 equiv (±)-**3an** (±)-**3an'**

(±)2-(-2-(4-cyanophenyl)cyclopropyl)-*N*-(quinolin-8-yl)acetamide (3an): The reaction was carried out according to General Procedure C using **1a** (21.2mg, 0.1 mmol), (4-cyanophenyl)methanesulfonyl fluoride (59.8 mg, 0.3 mmol), Na_2CO_3 (10.6 mg, 0.1 mmol), $Pd(OAc)_2$ (2.2 mg, 0.01 mmol), and anhydrous DMA (0.3 mL). The reaction was run for 24 h at 80 °C, and the crude residue was purified by silica gel flash column chromatography (20% – 30% ethyl acetate: hexanes) to afford a mixture of the two diastereomers of **(±)-3an** and **(±)-3an'** as a white solid (20.8 mg, 64%, **(±)-3an**:**(±)-3an'** =57:43). The mixture was purified by prep-TLC using 30% ethyl acetate: hexanes and only the pure **(±)-3an** were collected for analytical purposes. The following analytical data correspond to the major diastereomer, as drawn. **^{1}H NMR** (600 MHz, $CDCl_3$) δ 9.70 (s, 1H), 8.79 (dd, *J* = 4.2, 1.8 Hz, 1H), 8.72 (dd, *J* = 7.0, 2.0 Hz, 1H), 8.16 (dd, *J* = 8.2, 1.7 Hz, 1H), 7.56–7.43 (m, 5H), 7.36 (d, *J* = 8.3 Hz, 2H), 2.43 (td, *J* = 8.6, 6.2 Hz, 1H), 2.35 (dd, *J* = 16.0, 7.1 Hz, 1H), 2.22 (dd, *J* = 16.0, 7.6 Hz, 1H), 1.86–1.75 (m, 1H), 1.37 (td, *J* = 8.4, 5.7 Hz, 1H), 1.05 (q, *J* = 5.9 Hz, 1H). **^{13}C NMR** (151 MHz, $CDCl_3$) δ 170.42, 148.32, 144.43, 138.34, 136.54, 134.32, 132.08, 129.91, 128.05, 127.50, 121.84, 121.75, 119.08, 116.48, 110.05, 37.37, 21.26, 16.14, 10.24. **HRMS** (ESI-TOF) Calcd for $C_{21}H_{18}N_3O^+$ $[M+H]^+$ 328.1450, found 328.1454.

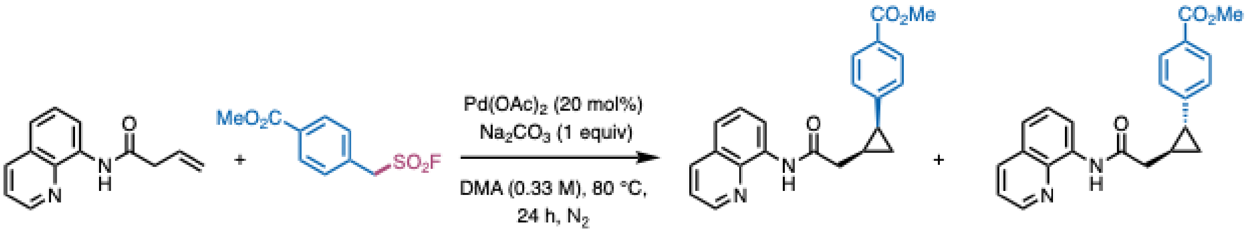

1a, 1 equiv 3 equiv (±)-**3ao** (±)-**3ao'**

(±)-methyl 4-(-2-(2-oxo-2-(quinolin-8-ylamino)ethyl)cyclopropyl)benzoate (3ao): The reaction was carried out according to General Procedure C using **1a** (21.2mg, 0.1 mmol), methyl 4-(fluorosulfonyl)methyl)benzoate (69.7 mg, 0.3 mmol), Na_2CO_3 (10.6 mg, 0.1 mmol), $Pd(OAc)_2$ (4.5 mg, 0.02 mmol), and anhydrous DMA (0.3 mL). The reaction was run for 24 h at 80 °C, and the crude residue was purified by silica gel flash column chromatography (20% – 30% ethyl acetate: hexanes) to afford a mixture of the two diastereomers of **(±)-3ao** and **(±)-3ao'**as white solids. (15.0 mg, 42%, **(±)-3ao**:**(±)-3ao'** = 52:48). The mixture was purified by prep-TLC using 30% ethyl acetate: hexanes and only the pure products were collected for analytical purposes. The following analytical data correspond to the major diastereomer, as drawn. **^{1}H NMR** (600 MHz, $CDCl_3$) δ 9.79 (s, 1H), 8.78–8.69 (m, 2H), 8.15 (dd, *J* = 8.3, 1.7 Hz, 1H), 7.94–7.85 (m, 2H), 7.54–7.47 (m, 2H), 7.44 (dd, *J* = 8.3, 4.2 Hz, 1H), 7.34 (d, *J* = 8.1 Hz, 2H), 3.86 (s, 3H), 2.44 (td, *J* = 8.6, 6.2 Hz, 1H), 2.34–2.22 (m, 2H), 1.77 (td, *J* = 8.2, 5.9 Hz, 1H), 1.35 (td, *J* = 8.4, 5.6 Hz, 1H), 1.07 (q, *J* = 5.9 Hz, 1H). **^{13}C NMR** (151 MHz, $CDCl_3$) δ 170.82, 167.13, 148.26, 144.09, 136.44, 134.50, 129.63, 129.17, 128.22, 128.04, 127.54, 121.69, 121.60, 116.49, 52.11, 37.48, 29.85, 21.13, 15.85, 10.18. **HRMS** (ESI-TOF) Calcd for $C_{22}H_{21}N_2O_3^+$ $[M+H]^+$ 361.1552, found 361.1557.

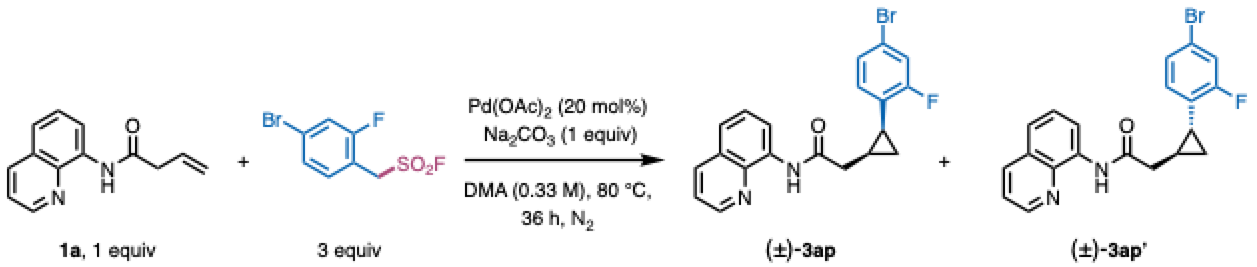

(±)2-(2-(4-bromo-2-fluorophenyl)cyclopropyl)-*N*-(quinolin-8-yl)acetamide (3ap): The reaction was carried out according to General Procedure C using **1a** (21.2mg, 0.1 mmol), (4-bromo-2-fluorophenyl)methanesulfonyl fluoride (81.3 mg, 0.3 mmol), Na_2CO_3 (10.6 mg, 0.1 mmol), $Pd(OAc)_2$ (4.5 mg, 0.02 mmol), and anhydrous DMA (0.3 mL). The reaction was run for 36 h at 80 °C, and the crude residue was purified by silica gel flash column chromatography (20% – 30% ethyl acetate: hexanes) to afford two inseparable mixtures of **(±)-3ap** and **(±)-3ap'** as colorless oil (7.9 mg, 20%, **(±)-3ap**:**(±)-3ap'** = 90:10; 8.3 mg, 21%, **(±)-3ap**:**(±)-3ap'** =5: 95). The following analytical data correspond to the major diastereomer, as drawn. **^{1}H NMR** (600 MHz, $CDCl_3$) δ 9.86 (s, 1H), 8.19–8.11 (m, 1H), 7.56–7.41 (m, 4H), 7.25–7.21 (m, 1H), 7.14 (dd, J = 9.4, 1.9 Hz, 1H), 7.05 (t, J = 8.0 Hz, 1H), 2.50 (dd, J = 16.0, 5.8 Hz, 1H), 2.28 (q, J = 8.0 Hz, 1H), 2.02–1.95 (m, 1H), 1.79 (dtd, J = 14.6, 8.8, 5.9 Hz, 1H), 1.37 (td, J = 8.4, 5.6 Hz, 1H), 0.97 (q, J = 5.8 Hz, 1H). **^{13}C NMR** (151 MHz, $CDCl_3$) δ 170.75, 148.33, 138.47, 136.47, 134.52, 131.53, 131.50, 128.05, 127.54, 127.23, 127.21, 121.73, 121.64, 119.09, 118.92, 116.55, 37.85, 14.93, 14.91, 14.67, 9.68. **HRMS** (ESI-TOF) Calcd for $C_{20}H_{17}BrFN_2O^+$ $[M+H]^+$ 399.0508, found 399.0505.

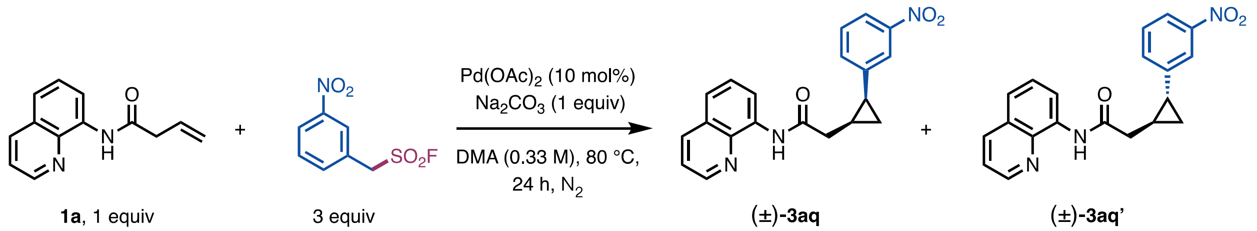

(±)2-(2-(3-nitrophenyl)cyclopropyl)-*N*-(quinolin-8-yl)acetamide (3aq): The reaction was carried out according to General Procedure C using **1a** (21.2 mg, 0.1 mmol), (3-nitrophenyl)methanesulfonyl fluoride (65.8 mg, 0.3 mmol), Na_2CO_3 (10.6 mg, 0.1 mmol), $Pd(OAc)_2$ (2.3 mg, 0.01 mmol), and anhydrous DMA (0.3 mL). The reaction was run for 24 h at 80 °C, and the crude residue was purified by prep-TLC using 30% ethyl acetate: hexanes to afford 3.7 mg (11%) of **(±)-3aq** as the colorless oil and 3.2 mg (9%) of **(±)-3aq'** as light yellow solid. The following analytical data correspond to the major diastereomer, as drawn. **^{1}H NMR** (600 MHz, $CDCl_3$) δ 9.77 (s, 1H), 8.77 (dd, J = 4.2, 1.7 Hz, 1H), 8.71 (dd, J = 7.1, 2.0 Hz, 1H), 8.23–8.12 (m, 2H), 7.98 (dt, J = 8.3, 1.7 Hz, 1H), 7.62 (d, J = 7.7 Hz, 1H), 7.53 – 7.48 (m, 2H), 7.46 (dd, J = 8.3, 4.2 Hz, 1H), 7.37 (t, J = 7.9 Hz, 1H), 2.49 (q, J = 8.3 Hz, 1H), 2.34 (dd, J = 16.2, 7.2 Hz, 1H), 2.27 (dd, J = 16.2, 7.5 Hz, 1H), 1.81 (q, J = 7.6 Hz, 1H), 1.41 (td, J = 8.4, 5.8 Hz, 1H), 1.09 (q, J = 5.9 Hz, 1H). **^{13}C NMR** (151 MHz, $CDCl_3$) δ 170.37, 148.31, 140.77, 138.38, 136.53, 135.66, 134.32, 129.17, 128.04, 127.52, 123.91, 121.78, 121.74, 121.56, 116.50, 37.53, 20.74, 15.69, 10.26. **HRMS** (ESI-TOF) Calcd for $C_{20}H_{18}N_3O_3^+$ $[M+H]^+$ 348.1348, found 348.1348.

Representative Procedures and Analytical Data for Alkene Scope

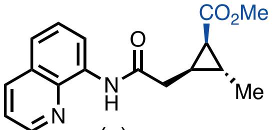

(±)-4aa

(±)-methyl 2-methyl-3-(2-oxo-2-(quinolin-8-ylamino)ethyl)cyclopropane-1-carboxylate (4aa): The reaction was carried out according to General Procedure C with (*Z*)-*N*-(quinolin-8-yl)pent-3-enamide (22.6 mg, 0.1 mmol), **2a** (46.8 mg, 0.3 mmol), Na_2CO_3 (10.6 mg, 0.1 mmol), $Pd(OAc)_2$ (2.3 mg, 0.01 mmol), and anhydrous DMA (0.3 mL). The reaction was run for 14 h at 80 °C, and the crude residue was purified by silica gel flash column chromatography (10–30% ethyl acetate:hexanes) to afford 21 mg (70%) of **(±)-4aa** as a colorless solid. **^{1}H NMR** (600 MHz, $CDCl_3$) δ 9.92 (s, 1H), 8.79 (ddd, *J* = 9.0, 5.8, 1.6 Hz, 2H), 8.15 (dd, *J* = 8.3, 1.7 Hz, 1H), 7.57–7.47 (m, 2H), 7.44 (dd, *J* = 8.2, 4.2 Hz, 1H), 2.95 (dd, *J* = 16.0, 7.1 Hz, 1H), 2.87 (dd, *J* = 15.9, 6.9 Hz, 1H), 1.64 (dd, *J* = 8.7, 4.8 Hz, 1H), 1.60 (dq, *J* = 8.7, 6.9 Hz, 1H), 1.52 (pd, *J* = 6.1, 4.6 Hz, 1H), 1.27 (d, *J* = 6.0 Hz, 3H). **^{13}C NMR** (151 MHz, $CDCl_3$) δ 173.49, 170.85, 148.21, 138.50, 136.41, 134.69, 128.04, 127.52, 121.70, 121.54, 116.56, 51.84, 35.71, 26.08, 25.68, 23.07, 17.81. **HRMS** (ESI-TOF) Calcd for $C_{17}H_{19}N_2O_3^+$ [M+H]$^+$ 299.1396, found 299.1391. **X-ray** (single-crystal) Single crystals suitable for X-ray diffraction were obtained from liquid diffusion of hexanes into a saturated solution of **4aa** in DCM (CCDC 2345089).[28]

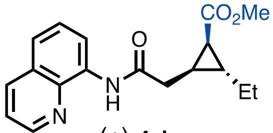

(±)-4ab

(±)-methyl 2-ethyl-3-(2-oxo-2-(quinolin-8-ylamino)ethyl)cyclopropane-1-carboxylate (4ab): The reaction was carried out according to General Procedure C with (*Z*)-*N*-(quinolin-8-yl)hex-3-enamide (24.0 mg, 0.1 mmol), **2a** (46.8 mg, 0.3 mmol), Na_2CO_3 (10.6 mg, 0.1 mmol), $Pd(OAc)_2$ (2.3 mg, 0.01 mmol), and anhydrous DMA (0.3 mL). The reaction was run for 14 h at 80 °C, and the crude residue was purified by silica gel flash column chromatography (5–20% ethyl acetate:hexanes) to afford 9.5 mg (30%) of **(±)-4ab** as colorless oil. **^{1}H NMR** (600 MHz, $CDCl_3$) δ 9.88 (s, 1H), 8.79 (dd, *J* = 13.1, 5.7 Hz, 2H), 8.15 (dd, *J* = 8.2, 1.6 Hz, 1H), 7.59–7.47 (m, 2H), 7.45 (dd, *J* = 8.2, 4.2 Hz, 1H), 3.67 (s, 3H), 3.05–2.80 (m, 2H), 1.66 (td, *J* = 8.8, 5.7 Hz, 2H), 1.56 (dq, *J* = 14.2, 7.1 Hz, 1H), 1.48 (p, *J* = 6.2 Hz, 1H), 1.40 (dp, *J* = 14.4, 7.2 Hz, 1H), 1.00 (t, *J* = 7.3 Hz, 3H). **^{13}C NMR** (151 MHz, $CDCl_3$) δ 173.68, 170.91, 148.23, 138.47, 136.42, 134.67, 128.03, 127.52, 121.72, 121.56, 116.56, 51.89, 35.83, 30.39, 26.04, 24.95, 24.33, 13.13. **HRMS** (ESI-TOF) Calcd for $C_{18}H_{21}N_2O_3^+$ [M+H]$^+$ 313.1552, found 313.1553.

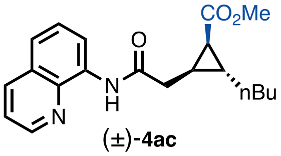

(±)-4ac

(±)-methyl 2-butyl-3-(2-oxo-2-(quinolin-8-ylamino)ethyl)cyclopropane-1-carboxylate (4ac): The reaction was carried out according to General Procedure C with (*Z*)-*N*-(quinolin-8-yl)oct-3-enamide (24.0 mg, 0.1 mmol), **2a** (46.8 mg, 0.3 mmol), Na_2CO_3 (10.6 mg, 0.1 mmol), $Pd(OAc)_2$ (4.5 mg, 0.02 mmol), and anhydrous DMA (0.3 mL). The reaction was run for 24 h at 80 °C, and the crude residue was purified by prep-TLC (30% ethyl acetate:hexanes) to afford 6.5 mg (19%) of **(±)-4ac** as a colorless oil. **^{1}H NMR** (600 MHz, $CDCl_3$) δ 9.88 (s, 1H), 8.98–8.38 (m, 2H), 8.15 (dd, *J* = 8.3, 1.7 Hz, 1H), 7.56–7.48 (m, 2H), 7.45 (dd, *J* = 8.3, 4.2 Hz, 1H), 3.67 (s, 3H), 2.91 (qd, *J* = 15.7, 6.8 Hz, 2H), 1.68–1.62 (m, 2H), 1.56–1.44 (m, 2H), 1.36 (ddq, *J* = 42.6, 14.4, 7.9 Hz, 5H), 0.86 (t, *J* = 7.3 Hz, 3H). **^{13}C NMR** (151 MHz, $CDCl_3$) δ 173.69, 170.93, 148.23, 138.48, 136.43, 134.67, 128.03, 127.52,

121.73, 121.57, 116.58, 51.90, 35.84, 32.66, 31.13, 28.78, 25.16, 24.62, 22.51, 14.15. **HRMS** (ESI-TOF) Calcd for $C_{20}H_{25}N_2O_3^+$ $[M+H]^+$ 341.1865, found 341.1866.

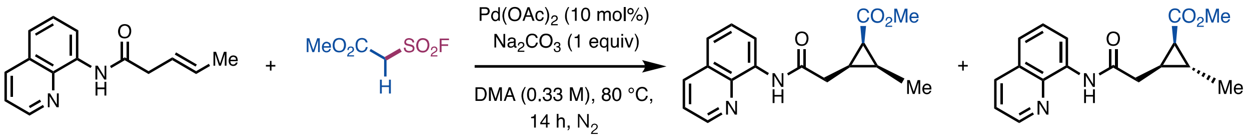

1 equiv 2a, 3 equiv (±)-4ad (±)-4ad’

(±) all-*cis*- methyl 2-methyl-3-(2-oxo-2-(quinolin-8-ylamino)ethyl)cyclopropane-1-carboxylate (4ad): The reaction was carried out according to General Procedure C using (*E*)-*N*-(quinolin-8-yl)pent-3-enamide (22.6 mg, 0.1 mmol), **2a** (46.8 mg, 0.3 mmol), Na_2CO_3 (10.6 mg, 0.1 mmol), $Pd(OAc)_2$ (2.3 mg, 0.01 mmol), and anhydrous DMA (0.3 mL). The reaction was run for 14 h at 80 °C, and the crude residue was purified by silica gel flash column chromatography (10% – 30% ethyl acetate: hexanes) to afford a mixture of the two diastereomers (12.5 mg, 42%, **(±)-4ad:(±)-4ad’**=1.14:1 *d.r.*) as colorless oil. The reported *d.r.* was determined by ^1H NMR analysis of purified **(±)-4ad**. The two diastereomers were separated for characterization by SFC using Phenomenex Lux Cellulose-2 4.6 x 100mm 3u as the column and 0% MeOH +10 mM NH_3 as the mobile phase. The following analytical data correspond to the major diastereomer, as drawn (with a trace amount of acetone). **^{1}H NMR** (600 MHz, $CDCl_3$) δ 9.97 (s, 1H), 8.96–8.48 (m, 2H), 8.15 (dd, J = 8.3, 1.7 Hz, 1H), 7.56–7.47 (m, 2H), 7.45 (dd, J = 8.2, 4.2 Hz, 1H), 3.67 (s, 3H), 3.10–3.01 (m, 1H), 3.02–2.91 (m, 1H), 1.92–1.85 (m, 2H), 1.71–1.64 (m, 1H), 1.31 (d, J = 6.5 Hz, 3H). **^{13}C NMR** (151 MHz, $CDCl_3$) δ 172.69, 171.27, 148.27, 138.60, 136.42, 134.82, 128.08, 127.55, 121.71, 121.52, 116.55, 51.54, 31.46, 20.99, 20.54, 18.89, 7.52. **HRMS** (ESI-TOF) Calcd for $C_{17}H_{19}N_2O_3^+$ $[M+H]^+$ 299.1396, found 299.1404. **X-ray** (single-crystal) Single crystals suitable for X-ray diffraction experiments were obtained from liquid diffusion of hexanes into a saturated solution of **4ad** in DCM (CCDC 2376390).[28]

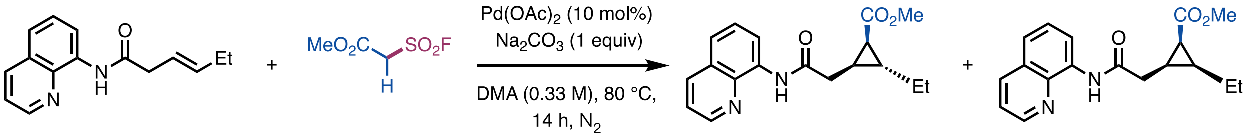

1 equiv 2a, 3 equiv (±)-4ab (±)-4ae’

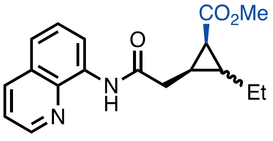

(±)-4ae

(±)-methyl -2-ethyl-3-(2-oxo-2-(quinolin-8-ylamino)ethyl)cyclopropane-1-carboxylate (4ae): The reaction was carried out according to General Procedure C with (*E*)-*N*-(quinolin-8-yl)hex-3-enamide (24.0 mg, 0.1 mmol), **2a** (46.8 mg, 0.3 mmol), Na_2CO_3 (10.6 mg, 0.1 mmol), $Pd(OAc)_2$ (2.3 mg, 0.01 mmol), and anhydrous DMA (0.3 mL). The reaction was run for 14 h at 80 °C, and the crude residue was purified by silica gel flash column chromatography (5–20% ethyl acetate:hexanes) to afford a mixture of the two diastereomers of **(±)-4ab (major)** and **(±)-4ae’** (minor) as a white solid (10.7 mg, 34%, **(±)-4ab**:**(±)-4ae’**= 2.5:1). The mixture was re-purified by prep-TLC (20% ethyl acetate:hexanes) and only the pure fraction of **(±)-4ab** (2.9 mg) and **(±)-4ae’** (3.5 mg) were collected.

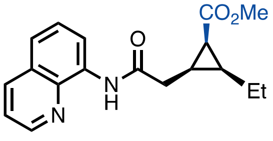

(±)-**4ae'**

The following analytical data correspond to the minor diastereomer **(±)-4ae'**. **^{1}H NMR** (600 MHz, $CDCl_3$) δ 9.96 (s, 1H), 8.87–8.71 (m, 2H), 8.16 (dd, J = 8.3, 1.7 Hz, 1H), 7.57–7.48 (m, 2H), 7.45 (dd, J = 8.2, 4.2 Hz, 1H), 3.15–3.07 (m, 1H), 3.02–2.96 (m, 1H), 1.95–1.88 (m, 2H), 1.82–1.66 (m, 2H), 1.56–1.49 (m, 1H), 0.98 (t, J = 7.4 Hz, 3H). **^{13}C NMR** (151 MHz, CDCl3) δ 171.88, 170.30, 147.26, 137.57, 135.41, 133.82, 127.07, 126.55, 120.71, 120.51, 115.55, 50.60, 30.64, 25.76, 20.13, 19.06, 14.93, 12.92. **HRMS** (ESI-TOF) Calcd for $C_{18}H_{21}N_2O_3^+$ $[M+H]^+$ 313.1552, found 313.1555.

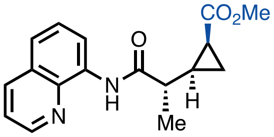

(±)-**4af**

(±)-methyl 2-(1-oxo-1-(quinolin-8-ylamino)propan-2-yl)cyclopropane-1-carboxylate (4af): The reaction was carried out according to General Procedure C with (2-methyl-*N*-(quinolin-8-yl)but-3-enamide (22.6 mg, 0.1 mmol), **2a** (46.8 mg, 0.3 mmol), Na_2CO_3 (10.6 mg, 0.1 mmol), $Pd(OAc)_2$ (2.3 mg, 0.01 mmol), and anhydrous DMA (0.3 mL). The reaction was run for 14 h at 80 °C, and the crude residue was purified by silica gel flash column chromatography (5–15% ethyl acetate:hexanes) to afford a mixture of two diastereomers (20.8 mg, 70%, **(±)-4af:(±)-4af'**=3.3:1 *d.r.*) as a yellow oil. The reported *d.r.* was determined by ^{1}H NMR analysis of purified **(±)-4af**. The following analytical data correspond to the major diastereomer, as drawn. **^{1}H NMR** (600 MHz, $CDCl_3$) δ 9.80 (s, 1H), 8.83–8.79 (m, 2H), 8.14 (dd, J = 8.2, 1.7 Hz, 1H), 7.57–7.49 (m, 2H), 7.44 (dd, J = 8.3, 4.2 Hz, 1H), 3.55 (s, 3H), 2.87 (dq, J = 10.4, 6.9 Hz, 1H), 1.89 (td, J = 8.3, 5.6 Hz, 1H), 1.72–1.68 (m, 1H), 1.46 (d, J = 6.9 Hz, 3H), 1.23 (dt, J = 8.3, 4.2 Hz, 1H), 1.07 (dt, J = 7.3, 5.1 Hz, 1H). **^{13}C NMR** (151 MHz, $CDCl_3$) δ 174.44, 174.20, 148.25, 138.62, 136.36, 134.81, 128.04, 127.52, 121.68, 121.59, 116.72, 51.92, 40.17, 25.80, 18.75, 18.01, 13.57. **HRMS** (ESI-TOF) Calcd for $C_{17}H_{19}N_2O_3^+$ $[M+H]^+$ 299.1396, found 299.1397.

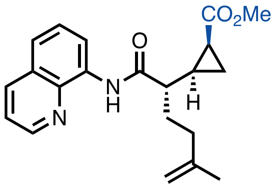

(±)-**4ag**

(±) methyl 2-(5-methyl-1-oxo-1-(quinolin-8-ylamino)hex-5-en-2-yl)cyclopropane-1-carboxylate (4ag): The reaction was carried out according to General Procedure C with 5-methyl-*N*-(quinolin-8-yl)-2-vinylhex-5-enamide (28.0 mg, 0.1 mmol), **2a** (46.8 mg, 0.3 mmol), Na_2CO_3 (10.6 mg, 0.1 mmol), $Pd(OAc)_2$ (2.3 mg, 0.01 mmol), and anhydrous DMA (0.3 mL). The reaction was run for 14 h at 80 °C, and the crude residue was purified by prep-TLC (20% ethyl acetate:hexanes) to afford a mixture of two diastereomers (29.2 mg, 83%, **(±)-4ag:(±)-4ag'**=10:1 *d.r.*) as a light-yellow oil. **^{1}H NMR** (600 MHz, $CDCl_3$) δ 9.75 (s, 1H), 8.87 – 8.80 (m, 2H), 8.15 (dd, J = 8.3, 1.7 Hz, 1H), 7.58 –7.48 (m, 2H), 7.44 (dd, J = 8.2, 4.2 Hz, 1H), 4.74 (d, J = 12.7 Hz, 2H), 3.48 (s, 3H), 2.74 (td, J = 9.1, 4.6 Hz, 1H), 2.20 –2.13 (m, 3H), 1.93 –1.80 (m, 2H), 1.76 – 1.68 (m, 4H), 1.26 (td, J = 8.3, 4.9 Hz, 1H), 1.12 (dt, J = 7.3, 5.1 Hz, 1H). **^{13}C NMR** (151 MHz, $CDCl_3$) δ 174.16, 173.81, 148.29, 145.18, 138.53, 136.31, 134.66, 128.01, 127.47, 121.69, 121.67, 116.71, 110.68, 51.86, 45.70, 35.63, 31.19, 24.84, 22.47, 18.01, 13.99. **HRMS** (ESI-TOF) Calcd for $C_{21}H_{25}N_2O_3^+$ $[M+H]^+$ 353.1865, found 353.1875.

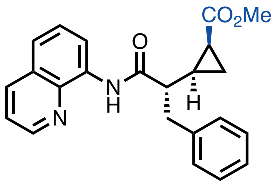

(±)-**4ah**

(±)-methyl 2-(1-oxo-3-phenyl-1-(quinolin-8-ylamino)propan-2-yl)cyclopropane-1-carboxylate (4ah): The reaction was carried out according to General Procedure C with 2-benzyl-*N*-(quinolin-8-yl)but-3-enamide (30.2 mg, 0.1 mmol), **2a** (46.8 mg, 0.3 mmol), Na_2CO_3 (10.6 mg, 0.1 mmol), $Pd(OAc)_2$ (2.3 mg, 0.01 mmol), and anhydrous DMA (0.3 mL). The reaction was run for 14 h at 80 °C, and the crude residue was purified by silica gel flash column chromatography (5–15% ethyl acetate:hexanes) to afford 33.5 mg (89%) of **(±)-4ah** as the colorless solid. **^{1}H NMR** (600 MHz, $CDCl_3$) δ 9.61 (s, 1H), 8.80 (dd, *J* = 7.5, 1.5 Hz, 1H), 8.75 (dd, *J* = 4.2, 1.7 Hz, 1H), 8.09 (dd, *J* = 8.2, 1.7 Hz, 1H), 7.52–7.42 (m, 2H), 7.38 (dd, *J* = 8.2, 4.2 Hz, 1H), 7.32–7.26 (m, 2H), 7.21 (t, *J* = 7.6 Hz, 2H), 7.15–7.07 (m, 1H), 3.48 (s, 3H), 3.33 (td, *J* = 10.3, 3.5 Hz, 1H), 3.10–3.02 (m, 2H), 1.82 (td, *J* = 8.3, 5.6 Hz, 1H), 1.75 (dtd, *J* = 10.1, 8.6, 7.2 Hz, 1H), 1.16 (td, *J* = 8.3, 4.8 Hz, 1H), 0.93 (dt, *J* = 7.3, 5.2 Hz, 1H). **^{13}C NMR** (151 MHz, $CDCl_3$) δ 174.15, 173.13, 148.14, 139.26, 138.45, 136.21, 134.57, 129.29, 128.44, 127.91, 127.40, 126.36, 121.64, 121.61, 116.69, 51.86, 48.05, 39.25, 24.66, 17.88, 14.33. **HRMS** (ESI-TOF) Calcd for $C_{23}H_{23}N_2O_3^+$ $[M+H]^+$ 375.1709, found 375.1705. **X-ray** (single-crystal) Single crystals suitable for X-ray diffraction experiments were obtained from liquid diffusion of hexanes into a saturated solution of **4ah** in DCM (CCDC 2367118).[28]

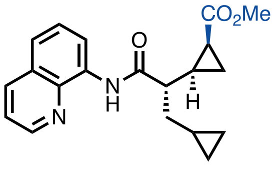

(±)-**4ai**

(±)-methyl 2-(1-oxo-3-phenyl-1-(quinolin-8-ylamino)propan-2-yl)cyclopropane-1-carboxylate (4ai): The reaction was carried out according to General Procedure C with 2-(cyclopropylmethyl)-*N*-(quinolin-8-yl)but-3-enamide (26.6 mg, 0.1 mmol), **2a** (46.8 mg, 0.3 mmol), Na_2CO_3 (10.6 mg, 0.1 mmol), $Pd(OAc)_2$ (2.3 mg, 0.01 mmol), and anhydrous DMA (0.3 mL). The reaction was run for 14 h at 80 °C, and the crude residue was purified by silica gel flash column chromatography (5–15% ethyl acetate:hexanes) to afford 22.9 mg (68%) of **(±)-4ai** as a light-yellow oil. **^{1}H NMR** (600 MHz, $CDCl_3$) δ 9.81 (s, 1H), 8.89–8.81 (m, 2H), 8.14 (dd, *J* = 8.3, 1.7 Hz, 1H), 7.56–7.47 (m, 2H), 7.44 (dd, *J* = 8.2, 4.2 Hz, 1H), 3.49 (s, 3H), 2.83 (ddd, *J* = 10.4, 8.9, 5.5 Hz, 1H), 1.92 (ddd, *J* = 13.7, 8.9, 7.0 Hz, 1H), 1.85 (td, *J* = 8.3, 5.5 Hz, 1H), 1.76–1.68 (m, 1H), 1.65 (ddd, *J* = 13.5, 7.1, 5.5 Hz, 1H), 1.25 (td, *J* = 8.3, 4.6 Hz, 1H), 1.13 (dt, *J* = 7.2, 5.1 Hz, 1H), 0.83 (tq, *J* = 8.1, 3.7 Hz, 1H), 0.47 (tt, *J* = 8.7, 4.2 Hz, 1H), 0.40 (tt, *J* = 8.5, 4.4 Hz, 1H), 0.14 (ddp, *J* = 18.3, 9.2, 4.7 Hz, 2H). **^{13}C NMR** (151 MHz, $CDCl_3$) δ 174.19, 174.12, 148.29, 138.57, 136.32, 134.79, 128.03, 127.52, 121.68, 121.58, 116.68, 51.86, 46.77, 38.37, 24.53, 18.08, 14.05, 9.35, 4.96, 4.75. **HRMS** (ESI-TOF) Calcd for $C_{20}H_{23}N_2O_3^+$ $[M+H]^+$ 339.1709, found 339.1711.

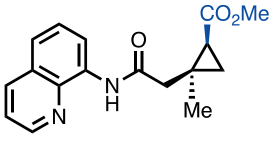

(±)-**4aj**

(±)-methyl 2-methyl-2-(2-oxo-2-(quinolin-8-ylamino)ethyl)cyclopropane-1-carboxylate (4aj): The reaction was carried out according to General Procedure C with 3-methyl-*N*-(quinolin-8-yl)but-3-enamide (22.6 mg, 0.1 mmol), **2a** (46.8 mg, 0.3 mmol), Na_2CO_3 (10.6 mg, 0.1 mmol), $Pd(OAc)_2$ (2.3 mg, 0.01 mmol), and anhydrous DMA (0.3 mL). The reaction was run for 14 h at 80 °C, and the crude residue was purified by silica gel flash column chromatography (5–15% ethyl acetate:hexanes) to afford 25.7 mg (86%) of **(±)-4ai** as a colorless oil. **^{1}H NMR** (600 MHz, $CDCl_3$) δ 9.89 (s, 1H), 8.86–8.72 (m, 2H), 8.14 (dd, *J* = 8.3, 1.7 Hz, 1H), 7.58–7.47 (m, 2H), 7.44 (dd, *J* = 8.3, 4.2 Hz, 1H), 3.70 (s, 3H), 2.91 (d, *J* = 1.4 Hz, 2H), 1.75 (dd, *J* = 8.1, 5.6 Hz, 1H), 1.38–1.34 (m, 1H), 1.34 (s, 3H), 1.11 (dd, *J* = 8.1, 4.5 Hz, 1H). **^{13}C NMR** (151 MHz, $CDCl_3$) δ 173.59, 170.10, 148.25, 138.50, 136.38, 134.70, 128.02,

127.49, 121.66, 121.47, 116.55, 51.97, 40.84, 25.93, 24.53, 24.48, 21.87. **HRMS** (ESI-TOF) Calcd for $C_{17}H_{19}N_2O_3^+$ [M+H]$^+$ 299.1396, found 299.1397.

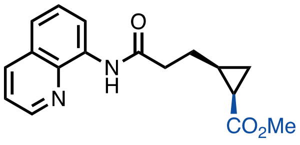

(±)-**4ak**

(±)-methyl 2-(3-oxo-3-(quinolin-8-ylamino)propyl)cyclopropane-1-carboxylate (4ak): The reaction was carried out according to General Procedure C with *N*-(quinolin-8-yl)pent-4-enamide (22.6 mg, 0.1 mmol), **2a** (46.8 mg, 0.3 mmol), Na_2CO_3 (10.6 mg, 0.1 mmol), $Pd(OAc)_2$ (2.3 mg, 0.01 mmol), and anhydrous DMA (0.3 mL). The reaction was run for 24 h at 80 °C, and the crude residue was purified by prep-TLC (20% ethyl acetate:hexanes) to afford 9.6 mg (32%) of **(±)-4ak** as colorless oil. **^{1}H NMR** (600 MHz, $CDCl_3$) δ 9.82 (s, 1H), 8.87–8.74 (m, 2H), 8.16 (dd, *J* = 8.2, 1.7 Hz, 1H), 7.58–7.48 (m, 2H), 7.46 (dd, *J* = 8.2, 4.2 Hz, 1H), 3.70 (s, 3H), 2.65–2.57 (m, 2H), 2.12 (dq, *J* = 14.5, 7.3 Hz, 1H), 2.03 (dq, *J* = 14.4, 7.3 Hz, 1H), 1.76 (td, *J* = 8.3, 5.5 Hz, 1H), 1.45 (h, *J* = 7.8 Hz, 1H), 1.09 (td, *J* = 8.2, 4.5 Hz, 1H), 1.04 (dt, *J* = 7.2, 5.1 Hz, 1H). **^{13}C NMR** (151 MHz, $CDCl_3$) δ 173.51, 171.31, 148.27, 138.45, 136.51, 134.62, 128.06, 127.56, 121.73, 121.54, 116.55, 51.84, 37.97, 23.35, 21.25, 18.27, 13.75. **HRMS** (ESI-TOF) Calcd for $C_{17}H_{19}N_2O_3^+$ [M+H]$^+$ 299.1396, found 299.1400.

Representative Procedures and Analytical Data for Directing Auxiliary Removal

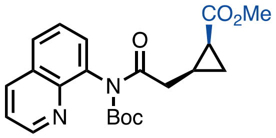

(±)-**5aa**

(±)-**methyl 2-(2-((*tert*-butoxycarbonyl)(quinolin-8-yl)amino)-2-oxoethyl)cyclopropane-1-carboxylate (5aa):** To a 8-mL vial equipped with a Teflon-coated magnetic stir bar, **1a** (56.9 mg, 0.2 mmol), DMAP (48.9 mg, 2 equiv) and Boc_2O (184 μL, 4.0 equiv) were added. The reaction vessel was sealed and then evacuated and backfilled with N_2 (×3). Anhydrous MeCN (400 μL, 0.5 M) was subsequently added by syringe. The reaction mixture was stirred at 55 ºC for 6 h. The reaction mixture was then allowed to cool to room temperature, and sat. aq. NH_4Cl (1 mL) and CH_2Cl_2 (1 mL) were added. The phases were separated, and the aqueous layer was extracted with CH_2Cl_2 (3 × 5 mL). The combined organic extracts were dried over Na_2SO_4, and the solvent was removed under reduced pressure. The crude residue was purified by silica gel flash column chromatography (30–50% ethyl acetate:hexanes) as the eluent to furnish (±)-**5aa** (63.4 mg, 83%) as the white solid. **^{1}H NMR** (600 MHz, $CDCl_3$) δ 8.87 (dd, *J* = 4.2, 1.7 Hz, 1H), 8.14 (dd, *J* = 8.3, 1.7 Hz, 1H), 7.80 (dd, *J* = 8.1, 1.5 Hz, 1H), 7.56–7.47 (m, 1H), 7.50 (dd, *J* = 7.3, 1.5 Hz, 1H), 7.38 (dd, *J* = 8.3, 4.2 Hz, 1H), 3.67 (s, 3H), 3.56 (dd, *J* = 18.0, 6.6 Hz, 1H), 3.39 (dd, *J* = 18.1, 7.7 Hz, 1H), 1.82 (td, *J* = 8.3, 5.6 Hz, 1H), 1.76–1.68 (m, 1H), 1.24 (d, *J* = 1.8 Hz, 9H), 1.16 (td, *J* = 8.3, 4.6 Hz, 1H), 1.07–1.02 (m, 1H). **^{13}C NMR** (151 MHz, $CDCl_3$) δ 175.66, 173.75, 152.86, 150.43, 144.26, 136.99, 136.00, 129.06, 128.96, 128.08, 126.14, 121.52, 82.73, 51.73, 36.27, 27.73, 17.64, 17.46, 13.72. **HRMS** (ESI-TOF) Calcd for $C_{12}H_{25}N_2O_5^+$ $[M+H]^+$ 385.1763, found 385.1771.

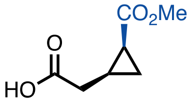

(±)-**6aa**

(±)-**2-(2-(methoxycarbonyl)cyclopropyl)acetic acid (6aa):** A solution of H_2O_2 (61.3 μL, 0.60 mmol, 30 wt.% in H_2O) in THF (300 μL) was added to a solution of $LiOH{\cdot}H_2O$ (16.8 mg, 0.40 mmol) in H_2O (300 μL) at 0 °C. The resulting mixture was added dropwise to a solution of Boc-protected amide (±)-**5aa** (38.4 mg, 0.10 mmol) in THF (400 μL) at 0 °C. The reaction was then stirred at room temperature for 14 h. Sat. aq. $Na_2S_2O_3$ (1 mL) and EtOAc (1 mL) were then added, and the reaction mixture was acidified to pH ~ 2 with 1 N aq. HCl. The phases were separated, and the aqueous layer was extracted with EtOAc (3 × 5 mL). The combined organic extracts were dried over Na_2SO_4, and the solvent was removed under reduced pressure. The crude residue was purified by silica gel flash column chromatography (30–50% ethyl acetate:hexanes) as the eluent to afford 13.4 mg (85%) of (±)-**6aa** as light-yellow solid. **^{1}H NMR** (600 MHz, $CDCl_3$) δ 3.69 (s, 3H), 2.76 (dd, *J* = 17.2, 6.6 Hz, 1H), 2.67 (dd, *J* = 17.3, 8.0 Hz, 1H), 1.83 (td, *J* = 8.3, 5.6 Hz, 1H), 1.61–1.51 (m, 1H), 1.18–1.15 (m, 1H), 1.00 (dt, *J* = 7.0, 5.2 Hz, 1H). **^{13}C NMR** (151 MHz, $CDCl_3$) δ 178.49, 173.47, 51.95, 32.07, 17.58, 16.54, 13.59. **HRMS** (ESI-TOF) Calcd for $C_7H_{10}O_4Na^+$ $[M+Na]^+$ 181.0477, found 181.0480. Single crystals of (±)-**6aa** suitable for X-ray diffraction were obtained from slow evaporation of hexanes into a saturated solution of (±)-**6aa** in DCM (CCDC 2394329). The compound is commercially available at Enamine (https://enaminestore.com/search) under the code EN300-37355413.[28]

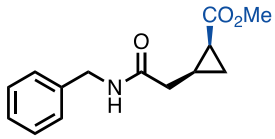

(±)-**6ab**

(±)-**methyl 2-(2-(benzylamino)-2-oxoethyl)cyclopropane-1-carboxylate (6ab):** To a 8-mL vial equipped with a Teflon-coated magnetic stir bar, **5aa** (38.4 mg, 0.10 mmol) was added benzylamine (21.8 μL, 0.20 mmol) in DMF (0.2 mL). The reaction was stirred at 60 °C for 24 h. After this time period, the crude reaction mixture was diluted with EtOAc (5 mL), washed with brine (10 mL), and extracted with EtOAc (3 × 5 mL). The combined organic extracts were dried over Na_2SO_4 and the solvent was removed under reduced pressure. The crude was purified by silica gel flash column chromatography (20–80% ethyl acetate:hexanes) as the eluent to afford 14.4 mg (58%) of **(±)-6ab** as colorless oil. **^{1}H NMR** (600 MHz, $CDCl_3$) δ 7.37–7.21 (m, 5H), 5.87 (s, 1H), 4.48 (dd, *J* = 14.8, 6.1 Hz, 1H), 4.37 (dd, *J* = 14.7, 5.5 Hz, 1H), 3.55 (s, 3H), 2.61 (dd, *J* = 15.2, 6.1 Hz, 1H), 2.50 (dd, *J* = 15.3, 8.6 Hz, 1H), 1.81 (td, *J* = 8.3, 5.6 Hz, 1H), 1.75–1.65 (m, 1H), 1.18 (td, *J* = 8.4, 4.7 Hz, 1H), 0.98 (q, *J* = 5.7 Hz, 1H). **^{13}C NMR** (151 MHz, $CDCl_3$) δ 174.50, 172.32, 138.83, 129.26, 128.32, 128.06, 52.34, 44.07, 34.98, 18.48, 18.03, 14.49. **HRMS** (ESI-TOF) Calcd for $C_{14}H_{18}NO_3^+$ [M+H]$^+$ 248.1287, found 248.1286.

Scale-Up Experiments

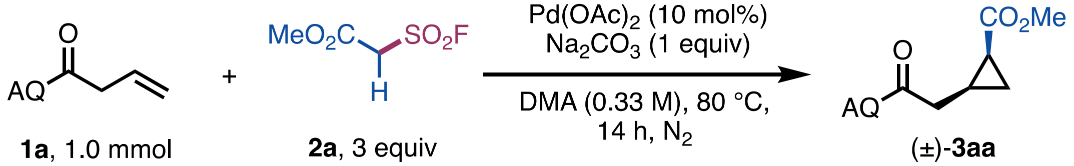

1-mmol scale of making (±)-3aa using glovebox: Outside of the glovebox, to 1.00-mmol scale in a 13 × 100 mm reaction tube with rubber-lined cap and equipped with a magnetic stir bar were added the *N*-(quinolin-8-yl)but-3-enamide **1a** (212.3 mg, 1.00 mmol), $Pd(OAc)_2$ (23 mg, 0.1 mmol, 10 mol%), and Na_2CO_3 (106 mg, 1.00 mmol). The vial was then introduced into a nitrogen-filled glovebox antechamber. Once transferred inside the glovebox, methyl 2-(fluorosulfonyl)acetate **2a** (468.4 mg, 3 mmol) was added to the vial followed by anhydrous DMA (3.0 mL, 0.33 M). The vial was sealed with a screw-top septum cap, removed from the glovebox, and placed in a heating block that was pre-heated to 80 °C for 14 h. After this time period, the resulting mixture was filtered through a pad of celite. Saturated Na_2HCO_3 solution (20 mL) was added to the filtrate, and the aqueous layer was extracted with ethyl acetate (3 × 5 mL). The crude was purified by silica gel flash column chromatography (10–20% ethyl acetate:hexanes) to afford **(±)-3aa** (245.1 mg, 86%) as a white solid.

1-mmol 10-mmol and 100-mmol scale of making (±)-3aa without using glovebox: In a 1000 mL round bottom flask equipped with a magnetic stir bar were added *N*-(quinolin-8-yl)but-3-enamide **1a** (21.20 g, 0.10 mol, 1.00 equiv), $Pd(OAc)_2$ (2.30 g, 0.01 mol, 10 mol%), Na_2CO_3 (10.60 g, 0.10 mol, 1.00 equiv) under argon atmosphere. Once transferred inside this flask, methyl 2-(fluorosulfonyl)acetate **2a** (46.80 g, 0.30 mol, 3.00 equiv) were added to the flask followed by anhydrous DMA (300 mL, 0.33M). The flask was sealed with a septum and heated at 80 °C (in an oil bath with a thermocouple) for 48 h. Then, the reaction mixture was poured into water (1000 mL) and EtOAc (300 mL); and was left at rt for 24 h. During that time, a lot of black insoluble amorphous precipitate was formed. The water-organic layer was filtered off from the precipitate. The transparent biphase filtrate was separated in separating flask, and the aqueous layer was extracted additionally with EtOAc (3 × 300 mL). The combined organic layer was dried under Na_2SO_4 and concentrated to give **(±)-3aa** (26.7 g, ca. 90% purity by ^1H NMR, 90% yield) which was used in the next step without further purification.

Different runs:

1-mmol scale: 86% conversion, 0.24 g of pure product (85% yield), run for 14 h.
10-mmol scale*: 75% conversion 1.75 g of pure product (62% yield), run for 48 h.
100-mmol scale: 26.7 g of pure product (81%), run for 48 h.

* *Note:* Several mixed fractions with impurities were observed and obtained during the purification using column chromatography. Only the pure fractions of **(±)-3aa** are collected when calculating the yields, which accounts for the lower yield.

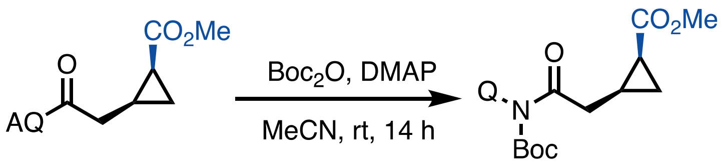

(±)-**3aa** (±)-**5aa**

Scale-up of (±)-5aa: In a 1000 mL round bottom flask equipped with a magnetic stir bar and anhydrous MeCN (260 mL, 0.5M) were added methyl (26 g, 90.40 mmol, 1.00 equiv), DMAP (22.37 g, 180.80 mmol, 2.00 equiv) and Boc_2O (79.77 g, 361.60 mmol, 4.00 equiv). The reaction mixture was stirred at room temperature for 14 h. The solution was concentrated under reduced pressure and sat. aq. solution of NH_4Cl (300 mL) and CH_2Cl_2 (300 mL) were added. The layers were separated and the aqueous layer was extracted with CH_2Cl_2 (3 × 100 mL). The combined organic extracts were dried over Na_2SO_4, filtered, and the solvent was removed under reduced pressure. The residue was purified by flash column chromatography (SiO_2, 30% – 50% EtOAc/hexanes, (EtOAc/Hexane, 3:7, R_f = 0.25). Yield: 22.60 g, 0.0588 mol, 58.8%, orange solid, m.p. = 111-112 °C after 2 steps.

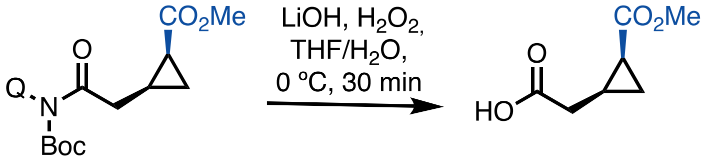

(±)-**5aa** (±)-**6aa**

Scale-up of (±)-6aa: A solution of H_2O_2 (3 mL, 28 mmol, 30 wt.% in H_2O, ~1.56 equiv) in THF (50 mL) was added to a solution of LiOH (0.50 g, 21 mmol, 1.17 equiv) in H_2O (50 mL) at 0 °C. The resulting mixture was added dropwise to a solution of *N*-Boc-protected amide (6.90 g, 18 mmol, 1.00 equiv) in THF (100 mL) at 0 °C. The reaction was then stirred at 0 -10 °C for 30 min (monitored by TLC). A solution of sat. aq. $Na_2S_2O_3$ (50 mL) and EtOAc (50 mL) was then added. The aqueous solution was washed with EtOAc (1 × 50 mL) and acified to pH ~ 2 with 1M HCl. Then the aqueous layer was extracted with EtOAc (3 × 50 mL). The combined organic extracts were dried over Na_2SO_4, filtered and the solvent was removed under reduced pressure. Yield: 2.45 g, 15.50 mmol, 86%, light yellow solid.

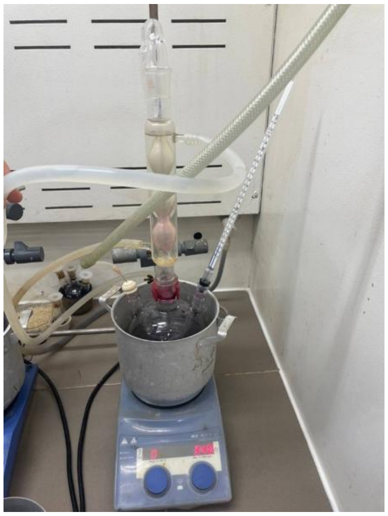

Reaction mixture

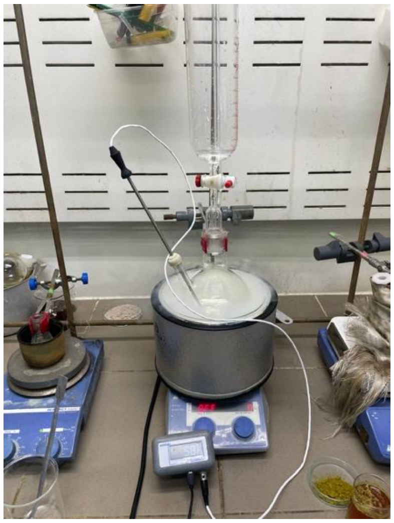

H_2O_2 solution addition

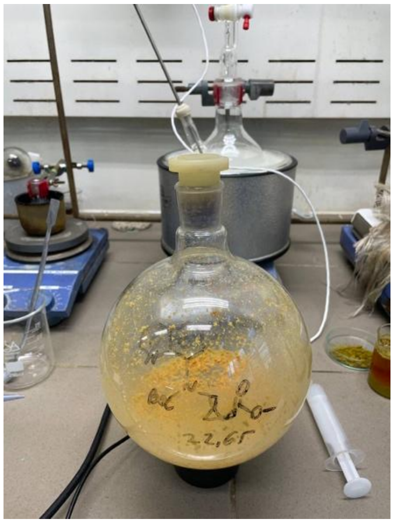

Flask with the starting material

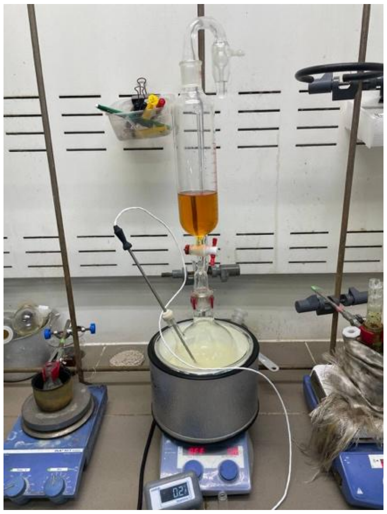

Addition of SM to mixture

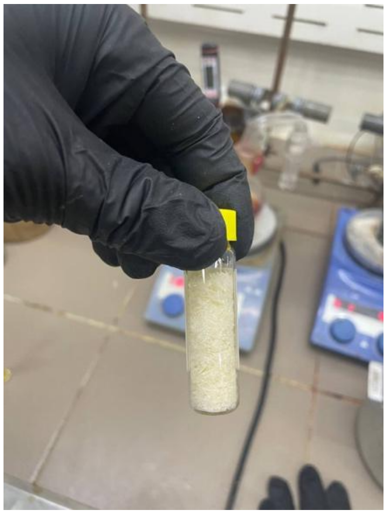

Product 6aa

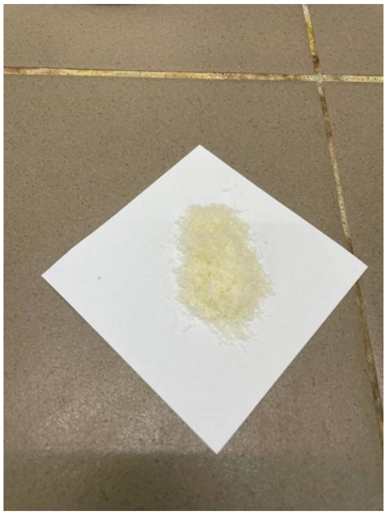

Product 6aa

The compound **6aa** is commercially available at Enamine (https://enaminestore.com/search) under the code EN300-37355413.

Base Screening at Lower Nucleophile Loading[a]

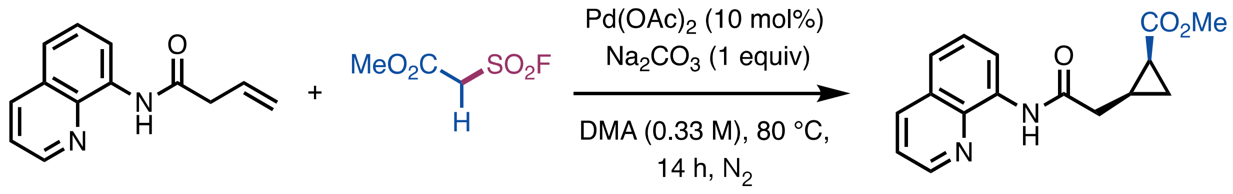

1a, 1 equiv **2a**, 1.5 equiv **(±)-3aa**

Entry	Base	Yield %[b]
1	Na_2CO_3	30
2	NaF	76
3	NaOBz	45
4	NaOAc	40
5	DBU	Trace
**6**	**NaF/Na_2CO_3 (3:1)**	**95**
7	NaF/Na_2CO_3 (1:1)	83
8	NaF/Na_2CO_3 (1:3)	55

[a]General Procedure C. [b]Quantitative ^{1}H NMR yield determined using 1,3,5-trimethoxybenzene as internal standard with a relaxation delay of 25 s.

***Lower nucleophile loading* (*1-mmol*)**: Outside of the glovebox, to 1.00-mmol scale in a 13 × 100 mm reaction tube with rubber-lined cap and equipped with a magnetic stir bar were added the *N*-(quinolin-8-yl)but-3-enamide **1a** (212.3 mg, 1.00 mmol) and $Pd(OAc)_2$ (23 mg, 0.1 mmol, 10 mol%), and Na_2CO_3 (106 mg, 1.00 mmol). The vial was then introduced into a nitrogen-filled glovebox antechamber. Once transferred inside the glovebox, NaF (31.5 mg, 0.75 mmol), Na_2CO_3 (26.5mg, 0.25mmol), and methyl 2-(fluorosulfonyl)acetate **2a** (246.5 mg, 1.55 mmol) were added to the vial followed by anhydrous DMA (3.0 mL, 0.33 M). The vial was sealed with a screw-top septum cap, removed from the glovebox, and placed in a heating block that was pre-heated to 80 °C for 18 h. After this time period, the resulting mixture was filtered through a pad of celite. Saturated Na_2HCO_3 solution (20 mL) was added to the filtrate, and the aqueous layer was extracted with ethyl acetate (3 × 5 mL). The crude was purified by silica gel flash column chromatography (10–20% ethyl acetate:hexanes) to afford 214.9 mg (76%) of ***(±)*-3aa** as a white solid.

X-Ray Crystallography
General Methods

The structures and their respective CCDC number are summarized in the table below. These data can be obtained free of charge from The Cambridge Crystallographic Data Centre via www.ccdc.cam.ac.uk/data_request/cif.

In all structures below, ellipsoids are colored according to their atom types.

dark blue: palladium

light blue: nitrogen

red: oxygen

grey: carbon

dark red: bromide

white: hydrogen

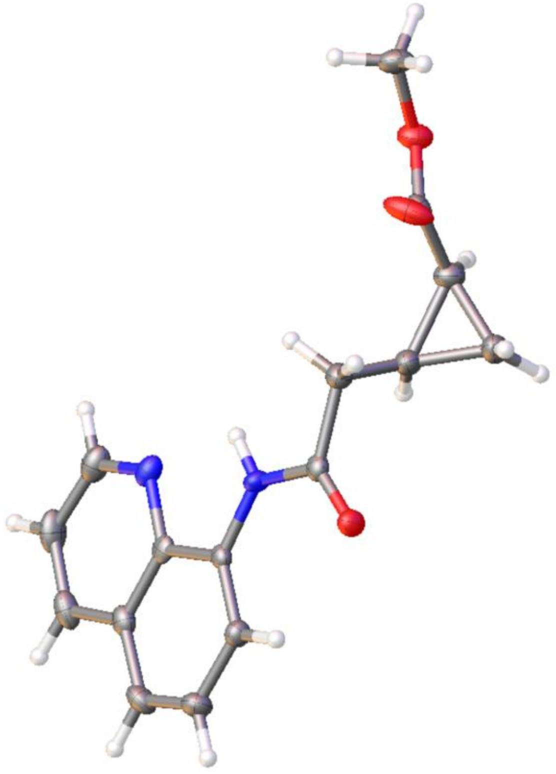

Experimental Summary for (±)-3aa

The single crystal X-ray diffraction studies were carried out on a Bruker D8-Venture 3-circle diffractometer equipped with a Photon3 detector and Mo K_{α} radiation (λ = 0.7107 Å).

Crystals of the subject compound were used as received. A 0.2 x 0.2 x 0.2 mm piece of a crystal was mounted on a Cryoloop with Paratone oil. Data were collected in a nitrogen gas stream at 100(2) K using ω scans. Crystal-to-detector distance was 50 mm and exposure time was 1 or 2 seconds depending on the 2θ range per frame using a scan width of 0.70°. Data collection was 99.3 % complete to 25.242 in θ. A total of 9167 reflections were collected covering the indices, −15<=h<=15, −15<=k<=15, −12<=l<=12. 2801 reflections were found to be symmetry independent, with a R_{int} of 0.0497. Indexing and unit cell refinement indicated a **P**rimitive, **Monoclinic** lattice. The space group was found to be ***P*2₁/*c***. The data were integrated using the

Bruker SAINT Software program and scaled using the SADABS software program. Solution by direct methods (SHELXT) produced a complete phasing model consistent with the proposed structure.

All nonhydrogen atoms were refined anisotropically by full-matrix least-squares (SHELXL-2014). All carbon bonded hydrogen atoms were placed using a riding model. Their positions were constrained relative to their parent atom using the appropriate HFIX command in SHELXL-2014. Crystallographic data are summarized in Table S12.

Notes: Great data! The sample is racemic. There is one copy of the compound in the asymmetric unit. There is a minor positional disorder in the cycloproply ring. The chemical formula for the compound is: $C_{16}H_{16}N_2O_3$

Table S12. Crystal data and structure refinement for **(±)-3aa** (CCDC 2171740).

Identification code	engle413_a	
Empirical formula	C16 H16 N2 O3	
Formula weight	284.31	
Temperature	100 K	
Wavelength	0.71073 Å	
Crystal system	Monoclinic	
Space group	P 1 21/c 1	
Unit cell dimensions	a = 12.3067(16) Å	a= 90°.
	b = 12.5895(16) Å	b= 101.701(4)°.
	c = 9.0721(12) Å	g = 90°.
Volume	1376.4(3) $Å^3$	
Z	4	
Density (calculated)	1.372 Mg/m^3	
Absorption coefficient	0.096 mm^{-1}	
F(000)	600	
Crystal size	0.19 x 0.15 x 0.12 mm^3	
Theta range for data collection	2.806 to 26.416°.	
Index ranges	-15<=h<=15, -15<=k<=15, -10<=l<=11	
Reflections collected	9167	
Independent reflections	2801 [R(int) = 0.0497]	
Completeness to theta = 25.242°	99.3 %	
Absorption correction	Semi-empirical from equivalents	
Max. and min. transmission	0.4908 and 0.4391	
Refinement method	Full-matrix least-squares on F^2	
Data / restraints / parameters	2801 / 52 / 232	
Goodness-of-fit on F^2	1.115	
Final R indices [I>2sigma(I)]	R1 = 0.0535, wR2 = 0.1005	
R indices (all data)	R1 = 0.0759, wR2 = 0.1070	
Largest diff. peak and hole	0.192 and -0.269 e.$Å^{-3}$	

Table S13. Atomic coordinates (x 10^4) and equivalent isotropic displacement parameters ($Å^2$x 10^3) for **(±)-3aa**. U(eq) is defined as one third of the trace of the orthogonalized U^{ij} tensor.

	x	y	z	U(eq)
O(1)	7376(1)	2253(1)	4822(2)	23(1)
N(1)	5360(1)	4692(1)	7500(2)	22(1)
N(2)	6630(1)	3379(1)	6318(2)	19(1)
C(1)	5008(2)	2731(2)	4478(2)	20(1)
C(2)	3851(2)	2789(2)	3926(2)	26(1)
C(3)	3196(2)	3472(2)	4530(2)	26(1)
C(4)	3676(2)	4140(2)	5741(2)	21(1)
C(5)	3060(2)	4869(2)	6436(2)	28(1)
C(6)	3592(2)	5470(2)	7613(3)	32(1)
C(7)	4743(2)	5358(2)	8101(2)	29(1)
C(8)	4829(2)	4088(1)	6318(2)	18(1)
C(9)	5498(2)	3365(2)	5656(2)	16(1)
C(10)	7487(2)	2862(2)	5899(2)	18(1)
C(11)	8600(2)	3094(2)	6915(2)	23(1)
O(2)	10397(1)	4885(1)	7826(2)	30(1)
O(3)	11630(1)	3768(1)	9187(2)	28(1)
C(12)	9563(2)	2697(2)	6289(2)	21(1)
C(13)	10079(2)	3376(2)	5271(3)	28(1)
C(14)	10726(2)	3173(2)	6836(2)	25(1)
C(15)	10880(3)	4042(2)	7960(3)	21(1)
C(16)	11852(3)	4552(2)	10387(3)	30(1)
O(2A)	11059(14)	2986(10)	8480(20)	40(5)
O(3A)	11348(12)	4700(11)	9205(16)	28(1)
C(12A)	9060(13)	3940(20)	6200(20)	28(5)
C(13A)	10002(18)	3820(20)	5420(20)	28(1)
C(14A)	10211(14)	4406(13)	6881(17)	26(3)
C(15A)	10870(30)	3926(12)	8270(30)	21(1)
C(16A)	12130(30)	4340(30)	10540(30)	30(1)

Table S14. Bond lengths [Å] and angles [°] for **(±)-3aa**.

O(1)-C(10)	1.228(2)	C(13)-H(13A)	0.9900
N(1)-C(7)	1.321(3)	C(13)-H(13B)	0.9900
N(1)-C(8)	1.367(2)	C(13)-C(14)	1.502(3)
N(2)-H(2)	0.8800	C(14)-H(14)	1.0000
N(2)-C(9)	1.401(2)	C(14)-C(15)	1.482(3)
N(2)-C(10)	1.358(2)	C(16)-H(16A)	0.9800
C(1)-H(1)	0.9500	C(16)-H(16B)	0.9800
C(1)-C(2)	1.413(3)	C(16)-H(16C)	0.9800
C(1)-C(9)	1.372(3)	O(2A)-C(15A)	1.213(10)
C(2)-H(2A)	0.9500	O(3A)-C(15A)	1.345(9)
C(2)-C(3)	1.367(3)	O(3A)-C(16A)	1.461(10)
C(3)-H(3)	0.9500	C(12A)-H(12A)	1.0000
C(3)-C(4)	1.415(3)	C(12A)-C(13A)	1.487(10)
C(4)-C(5)	1.417(3)	C(12A)-C(14A)	1.539(10)
C(4)-C(8)	1.410(3)	C(13A)-H(13C)	0.9900
C(5)-H(5)	0.9500	C(13A)-H(13D)	0.9900
C(5)-C(6)	1.363(3)	C(13A)-C(14A)	1.497(10)
C(6)-H(6)	0.9500	C(14A)-H(14A)	1.0000
C(6)-C(7)	1.403(3)	C(14A)-C(15A)	1.485(10)
C(7)-H(7)	0.9500	C(16A)-H(16D)	0.9800
C(8)-C(9)	1.438(2)	C(16A)-H(16E)	0.9800
C(10)-C(11)	1.516(3)	C(16A)-H(16F)	0.9800
C(11)-H(11A)	0.9900		
C(11)-H(11B)	0.9900	C(7)-N(1)-C(8)	116.99(18)
C(11)-H(11C)	0.9900	C(9)-N(2)-H(2)	115.3
C(11)-H(11D)	0.9900	C(10)-N(2)-H(2)	115.3
C(11)-C(12)	1.499(3)	C(10)-N(2)-C(9)	129.46(16)
C(11)-C(12A)	1.42(2)	C(2)-C(1)-H(1)	120.1
O(2)-C(15)	1.210(3)	C(9)-C(1)-H(1)	120.1
O(3)-C(15)	1.340(3)	C(9)-C(1)-C(2)	119.86(18)
O(3)-C(16)	1.454(3)	C(1)-C(2)-H(2A)	119.2
C(12)-H(12)	1.0000	C(3)-C(2)-C(1)	121.61(19)
C(12)-C(13)	1.491(3)	C(3)-C(2)-H(2A)	119.2
C(12)-C(14)	1.538(3)	C(2)-C(3)-H(3)	120.0

C(2)-C(3)-C(4)	119.91(19)	C(11)-C(12)-H(12)	115.1
C(4)-C(3)-H(3)	120.0	C(11)-C(12)-C(14)	120.22(18)
C(3)-C(4)-C(5)	123.62(19)	C(13)-C(12)-C(11)	120.8(2)
C(8)-C(4)-C(3)	119.39(17)	C(13)-C(12)-H(12)	115.1
C(8)-C(4)-C(5)	116.99(18)	C(13)-C(12)-C(14)	59.42(15)
C(4)-C(5)-H(5)	120.2	C(14)-C(12)-H(12)	115.1
C(6)-C(5)-C(4)	119.5(2)	C(12)-C(13)-H(13A)	117.6
C(6)-C(5)-H(5)	120.2	C(12)-C(13)-H(13B)	117.6
C(5)-C(6)-H(6)	120.5	C(12)-C(13)-C(14)	61.85(14)
C(5)-C(6)-C(7)	119.03(19)	H(13A)-C(13)-H(13B)	114.7
C(7)-C(6)-H(6)	120.5	C(14)-C(13)-H(13A)	117.6
N(1)-C(7)-C(6)	124.1(2)	C(14)-C(13)-H(13B)	117.6
N(1)-C(7)-H(7)	117.9	C(12)-C(14)-H(14)	115.7
C(6)-C(7)-H(7)	117.9	C(13)-C(14)-C(12)	58.73(14)
N(1)-C(8)-C(4)	123.33(17)	C(13)-C(14)-H(14)	115.7
N(1)-C(8)-C(9)	117.14(17)	C(15)-C(14)-C(12)	119.3(2)
C(4)-C(8)-C(9)	119.53(17)	C(15)-C(14)-C(13)	119.6(2)
N(2)-C(9)-C(8)	114.66(16)	C(15)-C(14)-H(14)	115.7
C(1)-C(9)-N(2)	125.64(17)	O(2)-C(15)-O(3)	123.5(2)
C(1)-C(9)-C(8)	119.70(17)	O(2)-C(15)-C(14)	125.9(2)
O(1)-C(10)-N(2)	123.70(18)	O(3)-C(15)-C(14)	110.6(2)
O(1)-C(10)-C(11)	123.00(17)	O(3)-C(16)-H(16A)	109.5
N(2)-C(10)-C(11)	113.28(16)	O(3)-C(16)-H(16B)	109.5
C(10)-C(11)-H(11A)	109.0	O(3)-C(16)-H(16C)	109.5
C(10)-C(11)-H(11B)	109.0	H(16A)-C(16)-H(16B)	109.5
C(10)-C(11)-H(11C)	110.7	H(16A)-C(16)-H(16C)	109.5
C(10)-C(11)-H(11D)	110.7	H(16B)-C(16)-H(16C)	109.5
H(11A)-C(11)-H(11B)	107.8	C(15A)-O(3A)-C(16A)	115.2(15)
H(11C)-C(11)-H(11D)	108.8	C(11)-C(12A)-H(12A)	113.9
C(12)-C(11)-C(10)	113.06(16)	C(11)-C(12A)-C(13A)	124(2)
C(12)-C(11)-H(11A)	109.0	C(11)-C(12A)-C(14A)	121.4(15)
C(12)-C(11)-H(11B)	109.0	C(13A)-C(12A)-H(12A)	113.9
C(12A)-C(11)-C(10)	105.2(7)	C(13A)-C(12A)-C(14A)	59.3(5)
C(12A)-C(11)-H(11C)	110.7	C(14A)-C(12A)-H(12A)	113.9
C(12A)-C(11)-H(11D)	110.7	C(12A)-C(13A)-H(13C)	117.6
C(15)-O(3)-C(16)	116.1(2)	C(12A)-C(13A)-H(13D)	117.6

C(12A)-C(13A)-C(14A)	62.1(6)	O(2A)-C(15A)-C(14A)	125.6(12)
H(13C)-C(13A)-H(13D)	114.6	O(3A)-C(15A)-C(14A)	109.5(11)
C(14A)-C(13A)-H(13C)	117.6	O(3A)-C(16A)-H(16D)	109.5
C(14A)-C(13A)-H(13D)	117.6	O(3A)-C(16A)-H(16E)	109.5
C(12A)-C(14A)-H(14A)	115.4	O(3A)-C(16A)-H(16F)	109.5
C(13A)-C(14A)-C(12A)	58.6(5)	H(16D)-C(16A)-H(16E)	109.5
C(13A)-C(14A)-H(14A)	115.4	H(16D)-C(16A)-H(16F)	109.5
C(15A)-C(14A)-C(12A)	119.2(14)	H(16E)-C(16A)-H(16F)	109.5
C(15A)-C(14A)-C(13A)	121.2(14)		
C(15A)-C(14A)-H(14A)	115.4		
O(2A)-C(15A)-O(3A)	124.5(12)		

Symmetry transformations used to generate equivalent atoms:

Table S15. Anisotropic displacement parameters ($Å^2$x 10^3) for Engle413. The anisotropic displacement factor exponent takes the form: $-2\pi^2[h^2 a^{*2}U^{11} + ... + 2 h k a^* b^* U^{12}]$

	U^{11}	U^{22}	U^{33}	U^{23}	U^{13}	U^{12}
O(1)	19(1)	26(1)	23(1)	-6(1)	6(1)	1(1)
N(1)	31(1)	18(1)	18(1)	0(1)	8(1)	2(1)
N(2)	18(1)	24(1)	15(1)	-5(1)	1(1)	0(1)
C(1)	21(1)	21(1)	20(1)	-2(1)	6(1)	0(1)
C(2)	22(1)	30(1)	25(1)	-4(1)	1(1)	-6(1)
C(3)	16(1)	29(1)	33(1)	2(1)	4(1)	-2(1)
C(4)	22(1)	19(1)	25(1)	4(1)	9(1)	-1(1)
C(5)	24(1)	24(1)	41(1)	5(1)	17(1)	3(1)
C(6)	38(1)	22(1)	42(1)	2(1)	25(1)	7(1)
C(7)	42(1)	21(1)	27(1)	-2(1)	15(1)	0(1)
C(8)	24(1)	14(1)	16(1)	5(1)	7(1)	-2(1)
C(9)	15(1)	18(1)	16(1)	3(1)	5(1)	-1(1)
C(10)	20(1)	20(1)	17(1)	4(1)	6(1)	0(1)
C(11)	20(1)	31(1)	20(1)	-1(1)	4(1)	-2(1)
O(2)	31(1)	24(1)	32(1)	-3(1)	1(1)	2(1)
O(3)	23(1)	28(1)	29(1)	-3(1)	-2(1)	0(1)
C(12)	16(1)	22(1)	25(1)	-3(1)	3(1)	-1(1)
C(13)	25(1)	36(2)	24(1)	-5(1)	8(1)	-7(1)
C(14)	18(1)	28(1)	30(1)	-6(1)	5(1)	0(1)
C(15)	14(1)	25(1)	25(1)	0(1)	6(1)	-3(1)
C(16)	29(2)	33(2)	25(1)	-7(1)	-4(1)	-2(1)
O(2A)	25(9)	25(2)	60(11)	1(3)	-14(8)	-5(3)
O(3A)	23(1)	28(1)	29(1)	-3(1)	-2(1)	0(1)
C(12A)	22(4)	42(12)	20(6)	-4(8)	4(3)	-5(5)
C(13A)	25(1)	36(2)	24(1)	-5(1)	8(1)	-7(1)
C(14A)	23(5)	29(6)	24(2)	-3(2)	1(3)	-1(4)
C(15A)	14(1)	25(1)	25(1)	0(1)	6(1)	-3(1)
C(16A)	29(2)	33(2)	25(1)	-7(1)	-4(1)	-2(1)

Table S16. Hydrogen coordinates (x 10^4) and isotropic displacement parameters ($Å^2$x 10^{3}) for **(±)-3aa**.

	x	y	z	U(eq)
H(2)	6812	3782	7123	23
H(1)	5447	2254	4033	24
H(2A)	3519	2342	3115	31
H(3)	2420	3499	4136	31
H(5)	2282	4937	6085	34
H(6)	3189	5958	8095	38
H(7)	5102	5789	8916	35
H(11A)	8676	3871	7071	28
H(11B)	8622	2760	7908	28
H(11C)	8504	3308	7931	28
H(11D)	9087	2462	7009	28
H(12)	9549	1920	6060	26
H(13A)	9771	4097	5040	34
H(13B)	10335	3022	4428	34
H(14)	11359	2663	6917	30
H(16A)	12047	5231	9980	45
H(16B)	12471	4309	11173	45
H(16C)	11189	4643	10817	45
H(12A)	8504	4487	5736	34
H(13C)	9981	4236	4487	34
H(13D)	10333	3101	5406	34
H(14A)	10276	5195	6800	31
H(16D)	11722	4126	11318	45
H(16E)	12643	4915	10929	45
H(16F)	12550	3729	10281	45

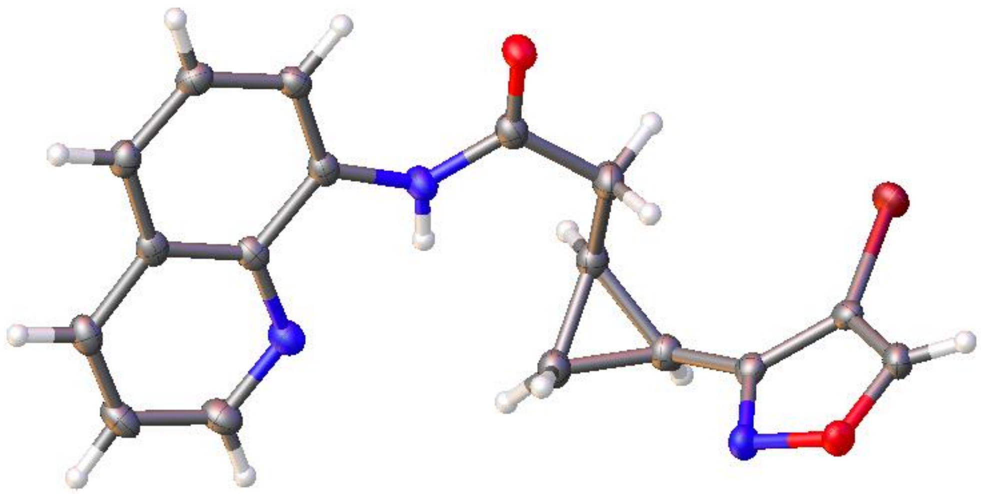

<u>Experimental Summary for (±)-3aj</u>

The single crystal X-ray diffraction studies were carried out on a Bruker D8 Venture Ultra diffractometer equipped with Mo K_α radiation (λ = 0.71073 Å). Crystals of the subject compound were used as received (grown from DCM/Hexanes).

A 0.200 x 0.080 x 0.060 mm crystal was mounted on a Cryoloop with Paratone oil.

Data were collected in a nitrogen gas stream at 100(2) K using ϕ and ϖ scans. Crystal-to-detector distance was 50 mm using exposure time 3.0s with a scan width of 0.70°. Data collection was 100.0 % complete to 25.242° in θ.

A total of 34805 reflections were collected. 2937 reflections were found to be symmetry independent, with a R_{int} of 0.0385. Indexing and unit cell refinement indicated a **Monoclinic** lattice. The space group was found to be ***P* 2_1/c**. The data were integrated using the Bruker SAINT Software program and scaled using the SADABS software program. Solution by direct methods (SHELXT) produced a complete phasing model consistent with the proposed structure.

All nonhydrogen atoms were refined anisotropically by full-matrix least-squares (SHELXL-2014). All carbon bonded hydrogen atoms were placed using a riding model. Their

positions were constrained relative to their parent atom using the appropriate HFIX command in SHELXL-2014.

Crystallographic data are summarized in Table S17.

Notes: Excellent data and refinement
Centrosymmetric space group, sample is “racemic”

Table S17. Crystal data and structure refinement for **(±)-3aj** (CCDC 2345363).

Identification code	Engle574	
Empirical formula	C17 H14 Br N3 O2	
Formula weight	372.22	
Temperature	100.00 K	
Wavelength	0.71073 Å	
Crystal system	Monoclinic	
Space group	P 1 21/c 1	
Unit cell dimensions	a = 8.0839(4) Å	α= 90°.
	b = 7.5263(3) Å	β= 95.830(2)°.
	c = 24.6522(11) Å	γ = 90°.
Volume	1492.13(12) $Å^3$	
Z	4	
Density (calculated)	1.657 Mg/m^3	
Absorption coefficient	2.769 mm^{-1}	
F(000)	752	
Crystal size	0.2 x 0.08 x 0.06 mm^3	
Theta range for data collection	2.533 to 26.014°.	
Index ranges	-9<=h<=9, -9<=k<=9, -30<=l<=30	
Reflections collected	34805	
Independent reflections	2937 [R(int) = 0.0385]	
Completeness to theta = 25.242°	100.0 %	
Absorption correction	Semi-empirical from equivalents	
Max. and min. transmission	0.6465 and 0.5728	
Refinement method	Full-matrix least-squares on F^2	
Data / restraints / parameters	2937 / 0 / 211	
Goodness-of-fit on F^2	1.109	
Final R indices [I>2sigma(I)]	R1 = 0.0265, wR2 = 0.0624	
R indices (all data)	R1 = 0.0299, wR2 = 0.0641	
Largest diff. peak and hole	0.438 and -0.288 e.$Å^{-3}$	

Table S18. Atomic coordinates (x 10^4) and equivalent isotropic displacement parameters ($Å^2$x 10^3) for **(±)-3aj**. U(eq) is defined as one third of the trace of the orthogonalized U^{ij} tensor.

	x	y	z	U(eq)
Br(1)	9187(1)	1065(1)	8008(1)	26(1)
O(1)	11539(2)	1388(2)	6650(1)	24(1)
O(2)	5289(2)	6480(2)	6898(1)	25(1)
N(1)	9927(2)	963(3)	6406(1)	23(1)
N(2)	4268(2)	4598(3)	6220(1)	22(1)
N(3)	3315(2)	3245(3)	5252(1)	23(1)
C(1)	11502(3)	1442(3)	7195(1)	24(1)
C(2)	9960(3)	1075(3)	7318(1)	20(1)
C(3)	9001(3)	769(3)	6808(1)	19(1)
C(4)	7224(3)	259(3)	6704(1)	22(1)
C(5)	6219(3)	948(3)	6202(1)	24(1)
C(6)	5862(3)	1607(3)	6755(1)	23(1)
C(7)	6285(3)	3463(3)	6941(1)	26(1)
C(8)	5214(3)	4997(3)	6690(1)	22(1)
C(9)	3368(3)	5751(3)	5848(1)	19(1)
C(10)	2973(3)	7490(3)	5947(1)	20(1)
C(11)	2088(3)	8507(3)	5532(1)	23(1)
C(12)	1597(3)	7802(3)	5029(1)	22(1)
C(13)	1980(3)	6015(3)	4916(1)	20(1)
C(14)	1533(3)	5168(3)	4409(1)	23(1)
C(15)	1987(3)	3452(3)	4334(1)	27(1)
C(16)	2886(3)	2535(3)	4769(1)	26(1)
C(17)	2875(3)	4977(3)	5326(1)	19(1)

Table S19. Bond lengths [Å] and angles [°] for **(±)-3aj**.

Br(1)-C(2)	1.871(2)	C(14)-C(15)	1.359(3)
O(1)-N(1)	1.415(2)	C(15)-H(15)	0.9500
O(1)-C(1)	1.348(3)	C(15)-C(16)	1.412(3)
O(2)-C(8)	1.227(3)	C(16)-H(16)	0.9500
N(1)-C(3)	1.309(3)		
N(2)-H(2)	0.83(3)	C(1)-O(1)-N(1)	108.48(16)
N(2)-C(8)	1.355(3)	C(3)-N(1)-O(1)	106.07(17)
N(2)-C(9)	1.411(3)	C(8)-N(2)-H(2)	118.2(19)
N(3)-C(16)	1.319(3)	C(8)-N(2)-C(9)	129.0(2)
N(3)-C(17)	1.368(3)	C(9)-N(2)-H(2)	111.6(19)
C(1)-H(1)	0.9500	C(16)-N(3)-C(17)	117.3(2)
C(1)-C(2)	1.341(3)	O(1)-C(1)-H(1)	125.2
C(2)-C(3)	1.427(3)	C(2)-C(1)-O(1)	109.53(19)
C(3)-C(4)	1.484(3)	C(2)-C(1)-H(1)	125.2
C(4)-H(4)	1.0000	C(1)-C(2)-Br(1)	127.57(17)
C(4)-C(5)	1.501(3)	C(1)-C(2)-C(3)	105.58(19)
C(4)-C(6)	1.512(3)	C(3)-C(2)-Br(1)	126.80(17)
C(5)-H(5A)	0.9900	N(1)-C(3)-C(2)	110.34(19)
C(5)-H(5B)	0.9900	N(1)-C(3)-C(4)	121.1(2)
C(5)-C(6)	1.506(3)	C(2)-C(3)-C(4)	128.6(2)
C(6)-H(6)	1.0000	C(3)-C(4)-H(4)	115.3
C(6)-C(7)	1.498(3)	C(3)-C(4)-C(5)	119.1(2)
C(7)-H(7A)	0.9900	C(3)-C(4)-C(6)	120.8(2)
C(7)-H(7B)	0.9900	C(5)-C(4)-H(4)	115.3
C(7)-C(8)	1.534(3)	C(5)-C(4)-C(6)	59.96(15)
C(9)-C(10)	1.375(3)	C(6)-C(4)-H(4)	115.3
C(9)-C(17)	1.431(3)	C(4)-C(5)-H(5A)	117.7
C(10)-H(10)	0.9500	C(4)-C(5)-H(5B)	117.7
C(10)-C(11)	1.413(3)	C(4)-C(5)-C(6)	60.36(15)
C(11)-H(11)	0.9500	H(5A)-C(5)-H(5B)	114.9
C(11)-C(12)	1.370(3)	C(6)-C(5)-H(5A)	117.7
C(12)-H(12)	0.9500	C(6)-C(5)-H(5B)	117.7
C(12)-C(13)	1.414(3)	C(4)-C(6)-H(6)	114.7
C(13)-C(14)	1.418(3)	C(5)-C(6)-C(4)	59.68(15)
C(13)-C(17)	1.416(3)	C(5)-C(6)-H(6)	114.7
C(14)-H(14)	0.9500	C(7)-C(6)-C(4)	120.4(2)

C(7)-C(6)-C(5)	121.6(2)
C(7)-C(6)-H(6)	114.7
C(6)-C(7)-H(7A)	107.7
C(6)-C(7)-H(7B)	107.7
C(6)-C(7)-C(8)	118.48(19)
H(7A)-C(7)-H(7B)	107.1
C(8)-C(7)-H(7A)	107.7
C(8)-C(7)-H(7B)	107.7
O(2)-C(8)-N(2)	123.9(2)
O(2)-C(8)-C(7)	120.9(2)
N(2)-C(8)-C(7)	115.1(2)
N(2)-C(9)-C(17)	114.4(2)
C(10)-C(9)-N(2)	125.7(2)
C(10)-C(9)-C(17)	119.9(2)
C(9)-C(10)-H(10)	120.1
C(9)-C(10)-C(11)	119.8(2)
C(11)-C(10)-H(10)	120.1
C(10)-C(11)-H(11)	119.2
C(12)-C(11)-C(10)	121.6(2)
C(12)-C(11)-H(11)	119.2
C(11)-C(12)-H(12)	120.1
C(11)-C(12)-C(13)	119.8(2)
C(13)-C(12)-H(12)	120.1
C(12)-C(13)-C(14)	124.0(2)
C(12)-C(13)-C(17)	119.4(2)
C(17)-C(13)-C(14)	116.6(2)
C(13)-C(14)-H(14)	120.1
C(15)-C(14)-C(13)	119.8(2)
C(15)-C(14)-H(14)	120.1
C(14)-C(15)-H(15)	120.4
C(14)-C(15)-C(16)	119.2(2)
C(16)-C(15)-H(15)	120.4
N(3)-C(16)-C(15)	123.6(2)
N(3)-C(16)-H(16)	118.2
C(15)-C(16)-H(16)	118.2
N(3)-C(17)-C(9)	117.12(19)
N(3)-C(17)-C(13)	123.4(2)
C(13)-C(17)-C(9)	119.4(2)

Symmetry transformations used to generate equivalent atoms:

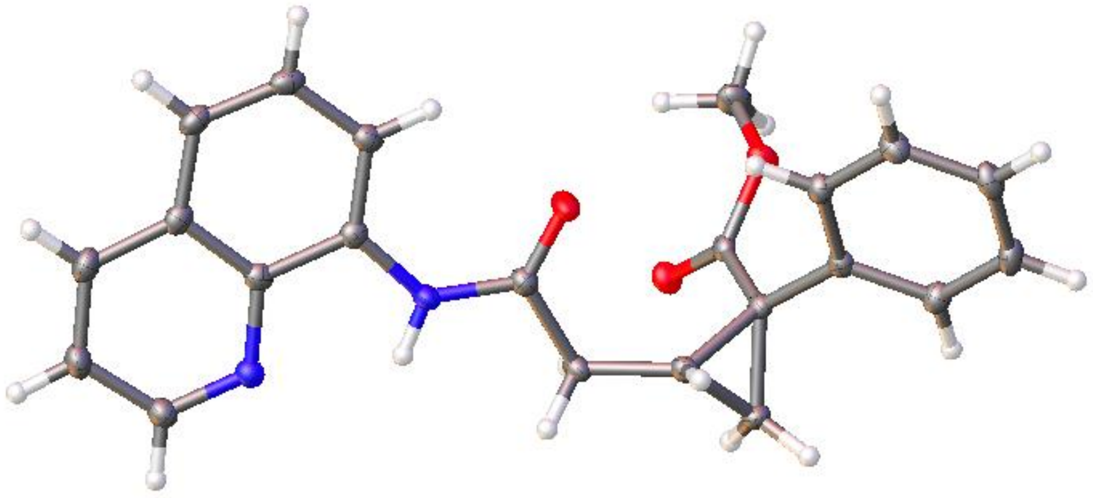

Experimental Summary for (±)-3al’

The single crystal X-ray diffraction studies were carried out on a Bruker SMART Pt135 diffractometer equipped with Cu K_α radiation (λ = 1.54178 Å). Crystals of the subject compound were used as received.

A 0.175 x 0.160 x 0.12 mm crystal was mounted on a Cryoloop with Paratone oil. Data were collected in a nitrogen gas stream at 100(2) K using ϕ and ω scans. Crystal-to-detector distance was 45 mm using exposure time 1.0, 2.0 and 4.0s with a scan width of 1.40°. Data collection was 98.5 % complete to 67.679° in θ.

A total of 23049 reflections were collected. 3281 reflections were found to be symmetry independent, with a Rint of 0.0403. Indexing and unit cell refinement indicated a Triclinic lattice. The space group was found to be P-1. The data were integrated using the Bruker SAINT Software program and scaled using the SADABS software program. Solution by direct methods (SHELXT) produced a complete phasing model consistent with the proposed structure.

All nonhydrogen atoms were refined anisotropically by full-matrix least-squares (SHELXL-2014). All carbon bonded hydrogen atoms were placed using a riding model. Their positions were constrained relative to their parent atom using the appropriate HFIX command in SHELXL-2014.

Crystallographic data are summarized in Table S20.

Notes: Excellent data and refinement

Centrosymmetric space group, sample is “racemic”

Table S20. Crystal data and structure refinement for **(±)-3al'** (CCDC 2351469).

Identification code	Engle583	
Empirical formula	C22 H20 N2 O3	
Formula weight	360.40	
Temperature	100.15 K	
Wavelength	1.54178 Å	
Crystal system	Triclinic	
Space group	P-1	
Unit cell dimensions	a = 8.8972(4) Å	α= 64.6640(10)°.
	b = 10.2905(5) Å	β= 89.7170(10)°.
	c = 10.8737(5) Å	γ = 79.6050(10)°.
Volume	882.10(7) $Å^3$	
Z	2	
Density (calculated)	1.357 Mg/m^3	
Absorption coefficient	0.737 mm^{-1}	
F(000)	380	
Crystal size	0.175 x 0.16 x 0.12 mm^3	
Theta range for data collection	4.514 to 70.071°.	
Index ranges	-10<=h<=10, -12<=k<=12, -13<=l<=13	
Reflections collected	23049	
Independent reflections	3281 [R(int) = 0.0403]	
Completeness to theta = 67.679°	98.5 %	
Absorption correction	Semi-empirical from equivalents	
Max. and min. transmission	0.7533 and 0.6809	
Refinement method	Full-matrix least-squares on F^2	
Data / restraints / parameters	3281 / 0 / 248	
Goodness-of-fit on F^2	1.035	
Final R indices [I>2sigma(I)]	R1 = 0.0336, wR2 = 0.0857	
R indices (all data)	R1 = 0.0342, wR2 = 0.0864	
Largest diff. peak and hole	0.283 and -0.255 e.$Å^{-3}$	

Table S21. Atomic coordinates (x 10^4) and equivalent isotropic displacement parameters (Å^{2}x 10^3) for Engle583. U(eq) is defined as one third of the trace of the orthogonalized U^{ij} tensor.

	x	y	z	U(eq)
O(1)	8042(1)	8924(1)	7767(1)	19(1)
O(2)	5547(1)	9954(1)	7163(1)	19(1)
O(3)	6186(1)	6259(1)	7074(1)	26(1)
N(1)	8426(1)	4693(1)	7212(1)	17(1)
N(2)	10625(1)	2694(1)	7022(1)	17(1)
C(2)	6155(1)	7772(1)	9206(1)	14(1)
C(1)	6706(1)	8909(1)	7990(1)	14(1)
C(14)	8228(1)	4368(1)	6105(1)	16(1)
C(15)	6995(1)	4990(1)	5136(1)	18(1)
C(16)	6953(1)	4584(1)	4050(1)	20(1)
C(9)	3411(1)	7822(1)	8623(1)	17(1)
C(10)	1854(1)	8086(1)	8799(1)	20(1)
C(8)	4476(1)	8026(1)	9433(1)	15(1)
C(13)	3960(1)	8466(1)	10434(1)	18(1)
C(6)	7462(1)	5585(1)	7630(1)	17(1)
C(3)	7326(1)	6976(1)	10442(1)	17(1)
C(21)	11751(1)	1720(1)	6940(1)	20(1)
C(20)	11809(1)	1272(1)	5877(1)	22(1)
C(5)	8152(1)	5696(1)	8850(1)	17(1)
C(18)	9392(1)	2916(1)	4912(1)	18(1)
C(19)	10635(1)	1861(1)	4872(1)	21(1)
C(11)	1347(1)	8530(1)	9796(1)	21(1)
C(7)	6010(1)	11128(1)	5994(1)	23(1)
C(22)	9448(1)	3299(1)	6017(1)	15(1)
C(12)	2398(1)	8711(1)	10620(1)	21(1)
C(4)	6966(1)	6172(1)	9646(1)	16(1)
C(17)	8115(1)	3591(1)	3923(1)	20(1)

Table S22. Bond lengths [Å] and angles [°] for **(±)-3al'**.

O(1)-C(1)	1.2129(13)	C(5)-H(5B)	0.9900
O(2)-C(1)	1.3457(13)	C(5)-C(4)	1.5065(14)
O(2)-C(7)	1.4510(13)	C(18)-C(19)	1.4179(16)
O(3)-C(6)	1.2239(13)	C(18)-C(22)	1.4203(15)
N(1)-H(1)	0.877(15)	C(18)-C(17)	1.4205(16)
N(1)-C(14)	1.3996(14)	C(19)-H(19)	0.9500
N(1)-C(6)	1.3617(14)	C(11)-H(11)	0.9500
N(2)-C(21)	1.3160(14)	C(11)-C(12)	1.3876(16)
N(2)-C(22)	1.3683(14)	C(7)-H(7A)	0.9800
C(2)-C(1)	1.4951(14)	C(7)-H(7B)	0.9800
C(2)-C(8)	1.5066(14)	C(7)-H(7C)	0.9800
C(2)-C(3)	1.5249(14)	C(12)-H(12)	0.9500
C(2)-C(4)	1.5369(14)	C(4)-H(4)	1.0000
C(14)-C(15)	1.3800(15)	C(17)-H(17)	0.9500
C(14)-C(22)	1.4333(15)		
C(15)-H(15)	0.9500	C(1)-O(2)-C(7)	115.06(8)
C(15)-C(16)	1.4139(15)	C(14)-N(1)-H(1)	111.8(9)
C(16)-H(16)	0.9500	C(6)-N(1)-H(1)	118.7(9)
C(16)-C(17)	1.3675(16)	C(6)-N(1)-C(14)	129.54(9)
C(9)-H(9)	0.9500	C(21)-N(2)-C(22)	117.57(9)
C(9)-C(10)	1.3917(15)	C(1)-C(2)-C(8)	118.05(8)
C(9)-C(8)	1.3984(15)	C(1)-C(2)-C(3)	114.47(8)
C(10)-H(10)	0.9500	C(1)-C(2)-C(4)	116.63(8)
C(10)-C(11)	1.3894(16)	C(8)-C(2)-C(3)	119.02(9)
C(8)-C(13)	1.3945(15)	C(8)-C(2)-C(4)	116.56(8)
C(13)-H(13)	0.9500	C(3)-C(2)-C(4)	58.63(7)
C(13)-C(12)	1.3967(16)	O(1)-C(1)-O(2)	122.86(9)
C(6)-C(5)	1.5210(14)	O(1)-C(1)-C(2)	124.79(9)
C(3)-H(3A)	0.9900	O(2)-C(1)-C(2)	112.33(8)
C(3)-H(3B)	0.9900	N(1)-C(14)-C(22)	114.59(9)
C(3)-C(4)	1.4991(14)	C(15)-C(14)-N(1)	125.51(10)
C(21)-H(21)	0.9500	C(15)-C(14)-C(22)	119.89(10)
C(21)-C(20)	1.4133(16)	C(14)-C(15)-H(15)	120.1
C(20)-H(20)	0.9500	C(14)-C(15)-C(16)	119.77(10)
C(20)-C(19)	1.3672(16)	C(16)-C(15)-H(15)	120.1
C(5)-H(5A)	0.9900	C(15)-C(16)-H(16)	119.2

C(17)-C(16)-C(15)	121.65(10)	C(4)-C(5)-H(5A)	108.9
C(17)-C(16)-H(16)	119.2	C(4)-C(5)-H(5B)	108.9
C(10)-C(9)-H(9)	119.9	C(19)-C(18)-C(22)	117.08(10)
C(10)-C(9)-C(8)	120.21(10)	C(19)-C(18)-C(17)	123.86(10)
C(8)-C(9)-H(9)	119.9	C(22)-C(18)-C(17)	119.06(10)
C(9)-C(10)-H(10)	119.8	C(20)-C(19)-C(18)	119.32(10)
C(11)-C(10)-C(9)	120.33(10)	C(20)-C(19)-H(19)	120.3
C(11)-C(10)-H(10)	119.8	C(18)-C(19)-H(19)	120.3
C(9)-C(8)-C(2)	119.62(9)	C(10)-C(11)-H(11)	120.1
C(13)-C(8)-C(2)	121.21(9)	C(12)-C(11)-C(10)	119.85(10)
C(13)-C(8)-C(9)	119.16(9)	C(12)-C(11)-H(11)	120.1
C(8)-C(13)-H(13)	119.8	O(2)-C(7)-H(7A)	109.5
C(8)-C(13)-C(12)	120.40(10)	O(2)-C(7)-H(7B)	109.5
C(12)-C(13)-H(13)	119.8	O(2)-C(7)-H(7C)	109.5
O(3)-C(6)-N(1)	124.24(10)	H(7A)-C(7)-H(7B)	109.5
O(3)-C(6)-C(5)	122.91(9)	H(7A)-C(7)-H(7C)	109.5
N(1)-C(6)-C(5)	112.84(9)	H(7B)-C(7)-H(7C)	109.5
C(2)-C(3)-H(3A)	117.7	N(2)-C(22)-C(14)	117.42(9)
C(2)-C(3)-H(3B)	117.7	N(2)-C(22)-C(18)	123.04(10)
H(3A)-C(3)-H(3B)	114.8	C(18)-C(22)-C(14)	119.53(10)
C(4)-C(3)-C(2)	61.08(7)	C(13)-C(12)-H(12)	120.0
C(4)-C(3)-H(3A)	117.7	C(11)-C(12)-C(13)	120.03(10)
C(4)-C(3)-H(3B)	117.7	C(11)-C(12)-H(12)	120.0
N(2)-C(21)-H(21)	118.1	C(2)-C(4)-H(4)	113.8
N(2)-C(21)-C(20)	123.75(10)	C(3)-C(4)-C(2)	60.29(7)
C(20)-C(21)-H(21)	118.1	C(3)-C(4)-C(5)	121.28(9)
C(21)-C(20)-H(20)	120.4	C(3)-C(4)-H(4)	113.8
C(19)-C(20)-C(21)	119.24(10)	C(5)-C(4)-C(2)	123.75(9)
C(19)-C(20)-H(20)	120.4	C(5)-C(4)-H(4)	113.8
C(6)-C(5)-H(5A)	108.9	C(16)-C(17)-C(18)	120.08(10)
C(6)-C(5)-H(5B)	108.9	C(16)-C(17)-H(17)	120.0
H(5A)-C(5)-H(5B)	107.7	C(18)-C(17)-H(17)	120.0
C(4)-C(5)-C(6)	113.27(9)		

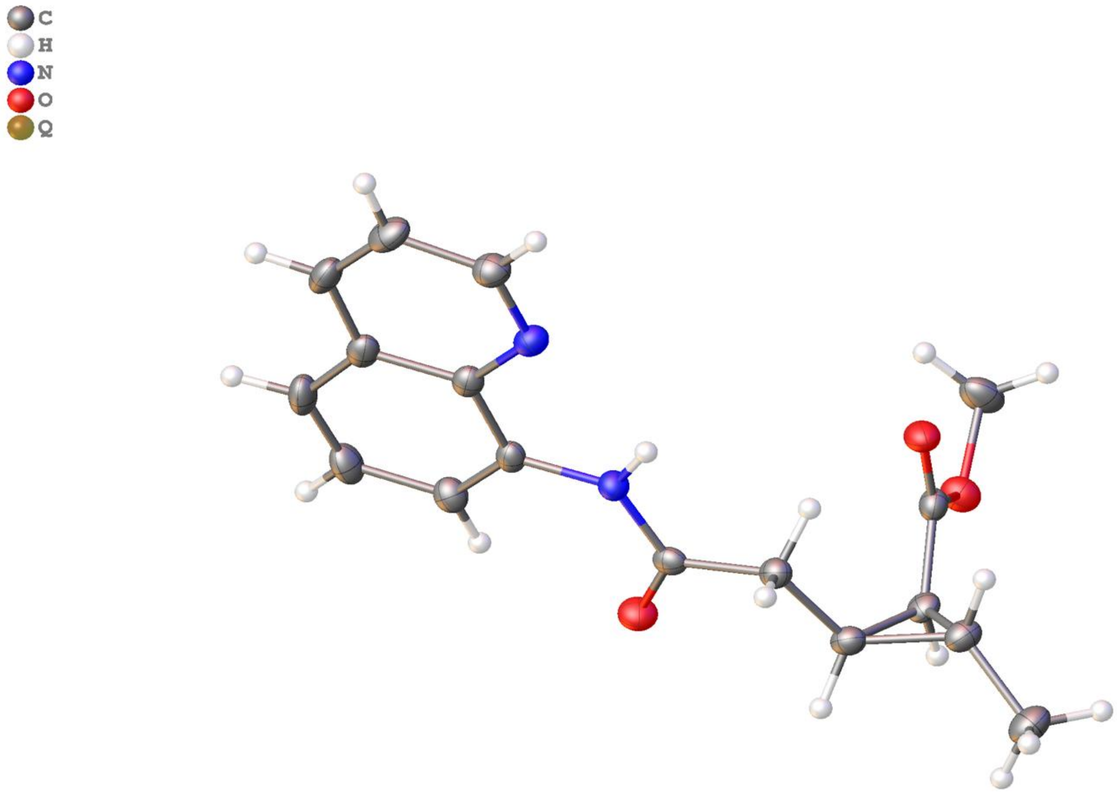
C
H
N
O
Q

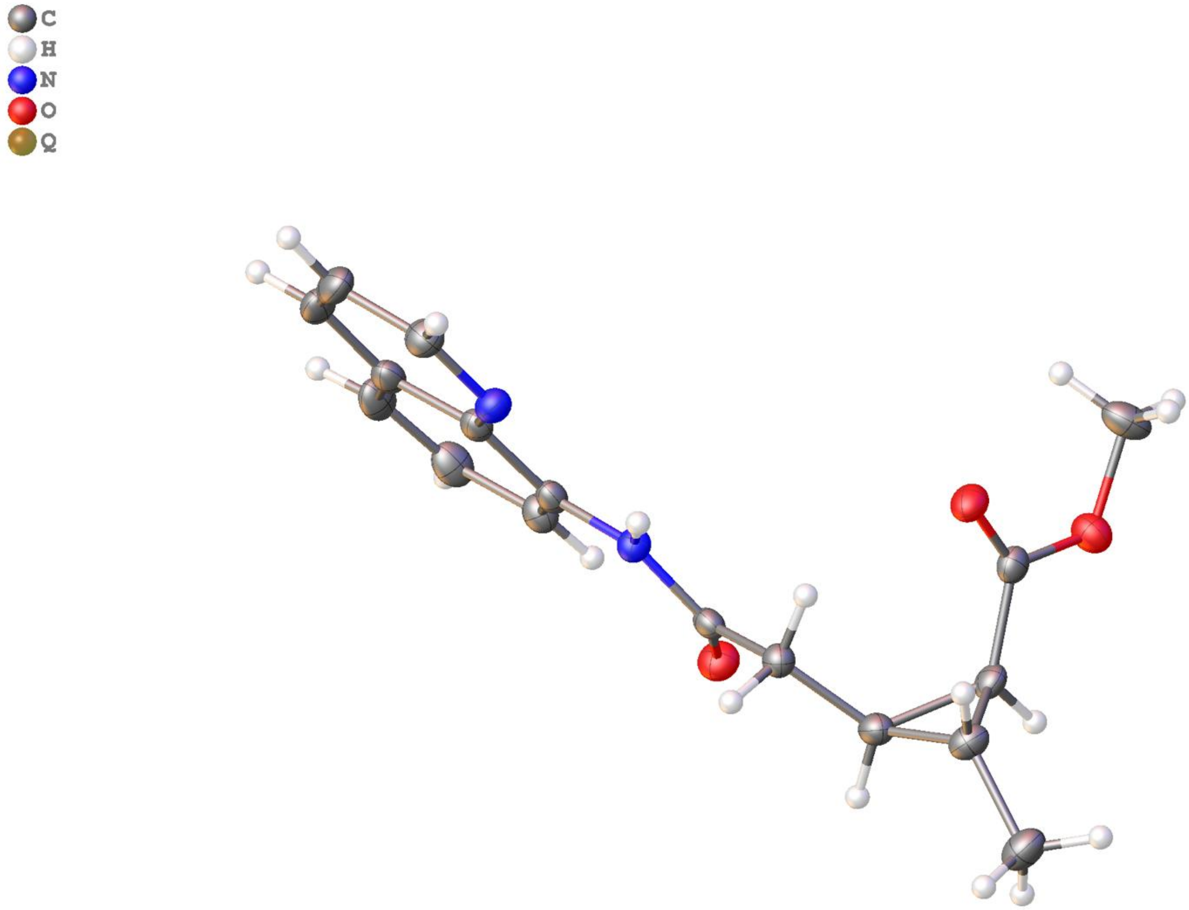
C
H
N
O
Q

Experimental Summary for (±)-4aa

The single crystal X-ray diffraction studies were carried out on a Bruker D8 Venture Ultra diffractometer equipped with Mo K_α radiation (λ = 0.71073 Å). Crystals of the subject compound were used as received (grown from DCM/Hexanes).

A 0.200 x 0.100 x 0.100 mm crystal was mounted on a Cryoloop with Paratone oil.

Data were collected in a nitrogen gas stream at 100(2) K using ϕ and ϖ scans. Crystal-to-detector distance was 40 mm using exposure time 10.0s with a scan width of 0.70°. Data collection was 100.0 % complete to 25.242° in θ.

A total of 11177 reflections were collected. 2827 reflections were found to be symmetry independent, with a R_{int} of 0.0492. Indexing and unit cell refinement indicated a **Monoclinic** lattice. The space group was found to be ***P* 2$_1$/c**. The data were integrated using the Bruker SAINT Software program and scaled using the SADABS software program. Solution by direct methods (SHELXT) produced a complete phasing model consistent with the proposed structure.

All nonhydrogen atoms were refined anisotropically by full-matrix least-squares (SHELXL-2014). All carbon bonded hydrogen atoms were placed using a riding model. Their positions were constrained relative to their parent atom using the appropriate HFIX command in SHELXL-2014.

Crystallographic data are summarized in Table S23.

Notes: Good data and refinement
Centrosymmetric space group, sample is "racemic"

Table S23. Crystal data and structure refinement for **(±)-4aa** (CCDC 2345089).

Identification code	Engle572	
Empirical formula	C17 H18 N2 O3	
Formula weight	298.33	
Temperature	100.15 K	
Wavelength	0.71073 Å	
Crystal system	Monoclinic	
Space group	P 1 21/c 1	
Unit cell dimensions	a = 10.7445(8) Å	α= 90°.
	b = 9.1571(7) Å	β= 96.035(3)°.
	c = 15.1984(14) Å	γ= 90°.
Volume	1487.1(2) Å^3	
Z	4	
Density (calculated)	1.333 Mg/m^3	
Absorption coefficient	0.092 mm^{-1}	
F(000)	632	
Crystal size	0.2 x 0.1 x 0.1 mm^3	
Theta range for data collection	1.906 to 25.682°.	
Index ranges	-12<=h<=13, -11<=k<=11, -14<=l<=18	
Reflections collected	11177	
Independent reflections	2827 [R(int) = 0.0492]	
Completeness to theta = 25.242°	100.0 %	
Absorption correction	Semi-empirical from equivalents	
Max. and min. transmission	0.7453 and 0.6624	
Refinement method	Full-matrix least-squares on F^2	
Data / restraints / parameters	2827 / 0 / 204	
Goodness-of-fit on F^2	1.025	
Final R indices [I>2sigma(I)]	R1 = 0.0421, wR2 = 0.0948	
R indices (all data)	R1 = 0.0663, wR2 = 0.1051	
Largest diff. peak and hole	0.209 and -0.220 e.Å^{-3}	

Table S24. Atomic coordinates (x 10^4) and equivalent isotropic displacement parameters ($Å^2$x 10^3) for **(±)-4aa**. U(eq) is defined as one third of the trace of the orthogonalized U^{ij} tensor.

	x	y	z	U(eq)
O(1)	3010(1)	8777(1)	4056(1)	26(1)
O(2)	3651(1)	4142(1)	4669(1)	25(1)
O(3)	1801(1)	3674(1)	3890(1)	26(1)
N(1)	7632(1)	8470(2)	4372(1)	22(1)
N(2)	5114(1)	8354(2)	4335(1)	18(1)
C(1)	3893(2)	8202(2)	4502(1)	18(1)
C(2)	3748(2)	7269(2)	5306(1)	20(1)
C(3)	2434(2)	6826(2)	5415(1)	21(1)
C(4)	2205(2)	5560(2)	5998(1)	24(1)
C(5)	1224(2)	5585(2)	6637(1)	32(1)
C(6)	1830(2)	5429(2)	5004(1)	20(1)
C(7)	2543(2)	4386(2)	4522(1)	20(1)
C(8)	2414(2)	2519(2)	3441(1)	32(1)
C(9)	5609(2)	9205(2)	3688(1)	19(1)
C(10)	4902(2)	9946(2)	3021(1)	23(1)
C(11)	5486(2)	10818(2)	2420(1)	28(1)
C(12)	6757(2)	10949(2)	2471(1)	28(1)
C(13)	7520(2)	10165(2)	3122(1)	23(1)
C(14)	8841(2)	10177(2)	3193(1)	27(1)
C(15)	9516(2)	9361(2)	3821(1)	29(1)
C(16)	8863(2)	8523(2)	4398(1)	26(1)
C(17)	6948(2)	9276(2)	3734(1)	19(1)

Table S25. Bond lengths [Å] and angles [°] for (±)**-4aa**.

O(1)-C(1)	1.226(2)	C(13)-C(17)	1.422(2)
O(2)-C(7)	1.208(2)	C(14)-H(14)	0.9500
O(3)-C(7)	1.350(2)	C(14)-C(15)	1.360(3)
O(3)-C(8)	1.453(2)	C(15)-H(15)	0.9500
N(1)-C(16)	1.320(2)	C(15)-C(16)	1.408(3)
N(1)-C(17)	1.369(2)	C(16)-H(16)	0.9500
N(2)-H(2)	0.859(19)		
N(2)-C(1)	1.370(2)	C(7)-O(3)-C(8)	114.95(14)
N(2)-C(9)	1.402(2)	C(16)-N(1)-C(17)	117.59(15)
C(1)-C(2)	1.512(2)	C(1)-N(2)-H(2)	114.7(12)
C(2)-H(2A)	0.9900	C(1)-N(2)-C(9)	128.85(15)
C(2)-H(2B)	0.9900	C(9)-N(2)-H(2)	116.4(12)
C(2)-C(3)	1.495(2)	O(1)-C(1)-N(2)	123.68(16)
C(3)-H(3)	1.0000	O(1)-C(1)-C(2)	123.38(16)
C(3)-C(4)	1.495(2)	N(2)-C(1)-C(2)	112.93(14)
C(3)-C(6)	1.537(2)	C(1)-C(2)-H(2A)	108.5
C(4)-H(4)	1.0000	C(1)-C(2)-H(2B)	108.5
C(4)-C(5)	1.506(2)	H(2A)-C(2)-H(2B)	107.5
C(4)-C(6)	1.528(2)	C(3)-C(2)-C(1)	114.98(14)
C(5)-H(5A)	0.9800	C(3)-C(2)-H(2A)	108.5
C(5)-H(5B)	0.9800	C(3)-C(2)-H(2B)	108.5
C(5)-H(5C)	0.9800	C(2)-C(3)-H(3)	114.4
C(6)-H(6)	1.0000	C(2)-C(3)-C(4)	119.50(15)
C(6)-C(7)	1.468(2)	C(2)-C(3)-C(6)	122.94(15)
C(8)-H(8A)	0.9800	C(4)-C(3)-H(3)	114.4
C(8)-H(8B)	0.9800	C(4)-C(3)-C(6)	60.51(11)
C(8)-H(8C)	0.9800	C(6)-C(3)-H(3)	114.4
C(9)-C(10)	1.379(2)	C(3)-C(4)-H(4)	114.1
C(9)-C(17)	1.435(2)	C(3)-C(4)-C(5)	122.70(17)
C(10)-H(10)	0.9500	C(3)-C(4)-C(6)	61.09(12)
C(10)-C(11)	1.410(3)	C(5)-C(4)-H(4)	114.1
C(11)-H(11)	0.9500	C(5)-C(4)-C(6)	120.64(15)
C(11)-C(12)	1.365(3)	C(6)-C(4)-H(4)	114.1
C(12)-H(12)	0.9500	C(4)-C(5)-H(5A)	109.5
C(12)-C(13)	1.413(2)	C(4)-C(5)-H(5B)	109.5
C(13)-C(14)	1.412(3)	C(4)-C(5)-H(5C)	109.5

H(5A)-C(5)-H(5B)	109.5	C(16)-C(15)-H(15)	120.9
H(5A)-C(5)-H(5C)	109.5	N(1)-C(16)-C(15)	124.35(17)
H(5B)-C(5)-H(5C)	109.5	N(1)-C(16)-H(16)	117.8
C(3)-C(6)-H(6)	115.9	C(15)-C(16)-H(16)	117.8
C(4)-C(6)-C(3)	58.40(11)	N(1)-C(17)-C(9)	118.07(15)
C(4)-C(6)-H(6)	115.9	N(1)-C(17)-C(13)	122.32(16)
C(7)-C(6)-C(3)	121.64(15)	C(13)-C(17)-C(9)	119.60(15)
C(7)-C(6)-C(4)	116.56(15)		
C(7)-C(6)-H(6)	115.9		
O(2)-C(7)-O(3)	122.64(17)		
O(2)-C(7)-C(6)	125.73(16)		
O(3)-C(7)-C(6)	111.60(15)		
O(3)-C(8)-H(8A)	109.5		
O(3)-C(8)-H(8B)	109.5		
O(3)-C(8)-H(8C)	109.5		
H(8A)-C(8)-H(8B)	109.5		
H(8A)-C(8)-H(8C)	109.5		
H(8B)-C(8)-H(8C)	109.5		
N(2)-C(9)-C(17)	116.29(15)		
C(10)-C(9)-N(2)	124.66(16)		
C(10)-C(9)-C(17)	119.04(16)		
C(9)-C(10)-H(10)	119.8		
C(9)-C(10)-C(11)	120.43(17)		
C(11)-C(10)-H(10)	119.8		
C(10)-C(11)-H(11)	119.2		
C(12)-C(11)-C(10)	121.60(17)		
C(12)-C(11)-H(11)	119.2		
C(11)-C(12)-H(12)	120.1		
C(11)-C(12)-C(13)	119.87(17)		
C(13)-C(12)-H(12)	120.1		
C(12)-C(13)-C(17)	119.35(16)		
C(14)-C(13)-C(12)	123.57(17)		
C(14)-C(13)-C(17)	117.06(16)		
C(13)-C(14)-H(14)	119.8		
C(15)-C(14)-C(13)	120.40(17)		
C(15)-C(14)-H(14)	119.8		
C(14)-C(15)-H(15)	120.9		
C(14)-C(15)-C(16)	118.28(17)		

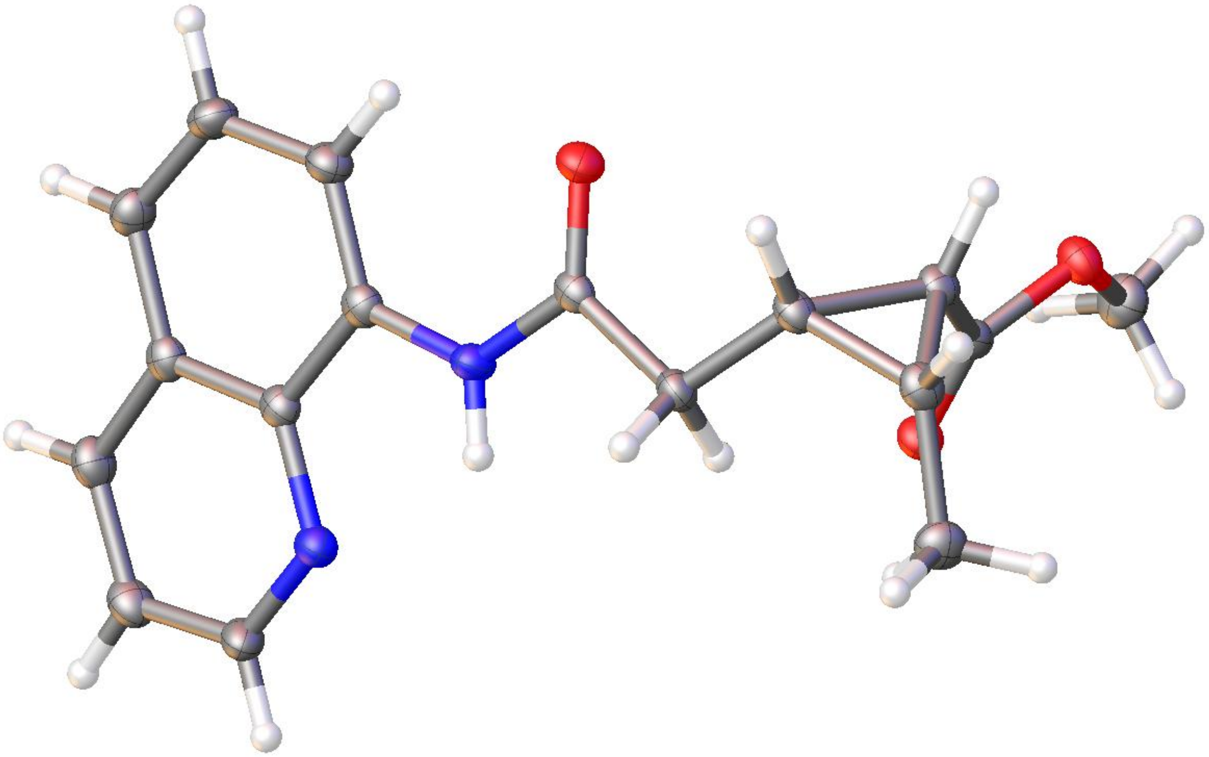

Experimental Summary for (±)-4ad

The single crystal X-ray diffraction studies were carried out on a Bruker SMART Pt135 CCD diffractometer equipped with Cu K_α radiation (λ = 1.54178 Å).

Crystals of the subject compound were used as received. A 0.2 x 0.15 x 0.15 mm piece of a crystal was mounted on a Cryoloop with Paratone oil. Data were collected in a nitrogen gas stream at 100(2) K using ϕ and ω scans. Crystal-to-detector distance was 45 mm and exposure time was 1, 2, 3, or 5 seconds depending on the 2θ range per frame using a scan width of 1.25°. Data collection was 98.8 % complete to 67.679° in θ. A total of 33503 reflections were collected covering the indices, −9<=h<=10, −12<=k<=12, −19<=l<=19. 2775 reflections were found to be symmetry independent, with a R_{int} of 0.0376. Indexing and unit cell refinement indicated a **P**rimitive, **Orthorhombic** lattice. The space group was found to be $\boldsymbol{P2_12_12_1}$. The data were integrated using the Bruker SAINT Software program and scaled using the SADABS

software program. Solution by direct methods (SHELXT) produced a complete phasing model consistent with the proposed structure.

All nonhydrogen atoms were refined anisotropically by full-matrix least-squares (SHELXL-2014). All carbon bonded hydrogen atoms were placed using a riding model. Their positions were constrained relative to their parent atom using the appropriate HFIX command in SHELXL-2014. Crystallographic data are summarized in Table S26.

Notes: Great data! Absolute stereochemistry was conclusively assigned (Absolute Structure Parameter = 0.02(3)). There is one copy of the compound in the asymmetric unit. The chemical formula for the compound is: $C_{17}H_{18}N_2O_3$

Table S26. Crystal data and structure refinement for **(±)-4ad** (CCDC 2376390).

Identification code	engle622_0m_a	
Empirical formula	C17 H18 N2 O3	
Formula weight	298.33	
Temperature	100.15 K	
Wavelength	1.54178 Å	
Crystal system	Orthorhombic	
Space group	$P2_12_12_1$	
Unit cell dimensions	a = 9.0977(4) Å	α= 90°.
	b = 10.3108(5) Å	β= 90°.
	c = 15.6663(7) Å	γ = 90°.
Volume	1469.57(12) $Å^3$	
Z	4	
Density (calculated)	1.348 Mg/m^3	
Absorption coefficient	0.762 mm^{-1}	
F(000)	632	
Crystal size	0.2 x 0.15 x 0.15 mm^3	
Theta range for data collection	5.135 to 70.520°.	
Index ranges	-9<=h<=10, -12<=k<=12, -19<=l<=19	
Reflections collected	33503	
Independent reflections	2775 [R(int) = 0.0376]	
Completeness to theta = 67.679°	98.8 %	
Absorption correction	Semi-empirical from equivalents	
Max. and min. transmission	0.5220 and 0.4464	
Refinement method	Full-matrix least-squares on F^2	
Data / restraints / parameters	2775 / 0 / 201	
Goodness-of-fit on F^2	1.057	
Final R indices [I>2sigma(I)]	R1 = 0.0249, wR2 = 0.0663	
R indices (all data)	R1 = 0.0249, wR2 = 0.0663	
Absolute structure parameter	0.02(3)	
Largest diff. peak and hole	0.223 and -0.150 e.$Å^{-3}$	

Table S27. Atomic coordinates (x 10^4) and equivalent isotropic displacement parameters ($Å^2$x 10^3) for **(±)-4ad**. U(eq) is defined as one third of the trace of the orthogonalized U^{ij} tensor.

	x	y	z	U(eq)
O(1)	3626(1)	-792(1)	6591(1)	21(1)
O(2)	4288(1)	1134(1)	7148(1)	20(1)
O(3)	8843(1)	574(1)	6479(1)	22(1)
N(1)	8622(1)	2727(1)	6797(1)	19(1)
N(2)	8611(1)	5316(1)	6732(1)	20(1)
C(1)	2477(2)	-126(2)	6127(1)	25(1)
C(2)	4478(2)	-27(1)	7088(1)	16(1)
C(3)	5618(2)	-816(1)	7512(1)	18(1)
C(4)	5937(2)	-671(1)	8473(1)	20(1)
C(5)	5108(2)	266(2)	9034(1)	25(1)
C(6)	7067(2)	-239(1)	7839(1)	18(1)
C(7)	7444(2)	1182(1)	7731(1)	19(1)
C(8)	8365(2)	1435(1)	6939(1)	16(1)
C(9)	9431(2)	3303(2)	6134(1)	18(1)
C(10)	10220(2)	2638(2)	5521(1)	21(1)
C(11)	11018(2)	3328(2)	4891(1)	23(1)
C(12)	11004(2)	4654(2)	4852(1)	22(1)
C(13)	10174(2)	5365(2)	5457(1)	18(1)
C(14)	10042(2)	6736(2)	5444(1)	22(1)
C(15)	9236(2)	7355(2)	6062(1)	23(1)
C(16)	8552(2)	6597(2)	6700(1)	22(1)
C(17)	9400(2)	4693(1)	6112(1)	17(1)

Table S28. Bond lengths [Å] and angles [°] for **(±)-4ad**.

O(1)-C(1)	1.4463(18)	C(12)-C(13)	1.417(2)
O(1)-C(2)	1.3523(17)	C(13)-C(14)	1.418(2)
O(2)-C(2)	1.2127(17)	C(13)-C(17)	1.425(2)
O(3)-C(8)	1.2232(18)	C(14)-H(14)	0.9500
N(1)-H(1)	0.8800	C(14)-C(15)	1.372(2)
N(1)-C(8)	1.3710(18)	C(15)-H(15)	0.9500
N(1)-C(9)	1.4051(19)	C(15)-C(16)	1.414(2)
N(2)-C(16)	1.3230(19)	C(16)-H(16)	0.9500
N(2)-C(17)	1.3668(19)		
C(1)-H(1A)	0.9800	C(2)-O(1)-C(1)	115.22(11)
C(1)-H(1B)	0.9800	C(8)-N(1)-H(1)	115.8
C(1)-H(1C)	0.9800	C(8)-N(1)-C(9)	128.33(12)
C(2)-C(3)	1.476(2)	C(9)-N(1)-H(1)	115.8
C(3)-H(3)	1.0000	C(16)-N(2)-C(17)	117.65(13)
C(3)-C(4)	1.5408(19)	O(1)-C(1)-H(1A)	109.5
C(3)-C(6)	1.535(2)	O(1)-C(1)-H(1B)	109.5
C(4)-H(4)	1.0000	O(1)-C(1)-H(1C)	109.5
C(4)-C(5)	1.509(2)	H(1A)-C(1)-H(1B)	109.5
C(4)-C(6)	1.497(2)	H(1A)-C(1)-H(1C)	109.5
C(5)-H(5A)	0.9800	H(1B)-C(1)-H(1C)	109.5
C(5)-H(5B)	0.9800	O(1)-C(2)-C(3)	109.86(11)
C(5)-H(5C)	0.9800	O(2)-C(2)-O(1)	122.63(13)
C(6)-H(6)	1.0000	O(2)-C(2)-C(3)	127.51(13)
C(6)-C(7)	1.5138(19)	C(2)-C(3)-H(3)	114.4
C(7)-H(7A)	0.9900	C(2)-C(3)-C(4)	121.22(13)
C(7)-H(7B)	0.9900	C(2)-C(3)-C(6)	122.66(12)
C(7)-C(8)	1.519(2)	C(4)-C(3)-H(3)	114.4
C(9)-C(10)	1.382(2)	C(6)-C(3)-H(3)	114.4
C(9)-C(17)	1.4340(19)	C(6)-C(3)-C(4)	58.24(9)
C(10)-H(10)	0.9500	C(3)-C(4)-H(4)	113.7
C(10)-C(11)	1.416(2)	C(5)-C(4)-C(3)	122.57(13)
C(11)-H(11)	0.9500	C(5)-C(4)-H(4)	113.7
C(11)-C(12)	1.369(2)	C(6)-C(4)-C(3)	60.67(9)
C(12)-H(12)	0.9500	C(6)-C(4)-H(4)	113.7

C(6)-C(4)-C(5)	122.65(12)	C(14)-C(13)-C(17)	116.91(14)
C(4)-C(5)-H(5A)	109.5	C(13)-C(14)-H(14)	120.0
C(4)-C(5)-H(5B)	109.5	C(15)-C(14)-C(13)	119.96(14)
C(4)-C(5)-H(5C)	109.5	C(15)-C(14)-H(14)	120.0
H(5A)-C(5)-H(5B)	109.5	C(14)-C(15)-H(15)	120.8
H(5A)-C(5)-H(5C)	109.5	C(14)-C(15)-C(16)	118.49(14)
H(5B)-C(5)-H(5C)	109.5	C(16)-C(15)-H(15)	120.8
C(3)-C(6)-H(6)	114.1	N(2)-C(16)-C(15)	124.07(14)
C(4)-C(6)-C(3)	61.08(9)	N(2)-C(16)-H(16)	118.0
C(4)-C(6)-H(6)	114.1	C(15)-C(16)-H(16)	118.0
C(4)-C(6)-C(7)	121.22(12)	N(2)-C(17)-C(9)	117.58(13)
C(7)-C(6)-C(3)	122.20(12)	N(2)-C(17)-C(13)	122.86(13)
C(7)-C(6)-H(6)	114.1	C(13)-C(17)-C(9)	119.55(13)
C(6)-C(7)-H(7A)	109.1		
C(6)-C(7)-H(7B)	109.1		
C(6)-C(7)-C(8)	112.52(11)		
H(7A)-C(7)-H(7B)	107.8		
C(8)-C(7)-H(7A)	109.1		
C(8)-C(7)-H(7B)	109.1		
O(3)-C(8)-N(1)	123.27(13)		
O(3)-C(8)-C(7)	123.55(13)		
N(1)-C(8)-C(7)	113.18(12)		
N(1)-C(9)-C(17)	115.45(13)		
C(10)-C(9)-N(1)	125.20(13)		
C(10)-C(9)-C(17)	119.35(14)		
C(9)-C(10)-H(10)	120.0		
C(9)-C(10)-C(11)	120.08(14)		
C(11)-C(10)-H(10)	120.0		
C(10)-C(11)-H(11)	119.1		
C(12)-C(11)-C(10)	121.89(14)		
C(12)-C(11)-H(11)	119.1		
C(11)-C(12)-H(12)	120.3		
C(11)-C(12)-C(13)	119.48(14)		
C(13)-C(12)-H(12)	120.3		
C(12)-C(13)-C(14)	123.49(14)		
C(12)-C(13)-C(17)	119.60(14)		

Symmetry transformations used to generate equivalent atoms:

Table S29. Anisotropic displacement parameters ($Å^2$x 10^3) for **(±)-4ad**. The anisotropic displacement factor exponent takes the form: $-2\pi^2$[h^2 $a^{*2}U^{11}$ + ... + 2 h k a* b* U^{12}]

	U^{11}	U^{22}	U^{33}	U^{23}	U^{13}	U^{12}
O(1)	21(1)	18(1)	25(1)	-1(1)	-5(1)	-1(1)
O(2)	22(1)	14(1)	24(1)	1(1)	0(1)	2(1)
O(3)	24(1)	15(1)	26(1)	-2(1)	1(1)	2(1)
N(1)	22(1)	15(1)	19(1)	-2(1)	2(1)	1(1)
N(2)	19(1)	18(1)	24(1)	-2(1)	1(1)	0(1)
C(1)	21(1)	30(1)	25(1)	2(1)	-5(1)	1(1)
C(2)	18(1)	15(1)	16(1)	0(1)	3(1)	-2(1)
C(3)	20(1)	12(1)	21(1)	1(1)	-1(1)	-1(1)
C(4)	23(1)	17(1)	20(1)	4(1)	-2(1)	-1(1)
C(5)	28(1)	26(1)	20(1)	1(1)	1(1)	1(1)
C(6)	19(1)	15(1)	22(1)	0(1)	-4(1)	0(1)
C(7)	19(1)	15(1)	22(1)	-2(1)	-1(1)	-2(1)
C(8)	15(1)	16(1)	19(1)	-1(1)	-5(1)	1(1)
C(9)	17(1)	18(1)	18(1)	0(1)	-3(1)	-1(1)
C(10)	23(1)	17(1)	23(1)	-2(1)	0(1)	2(1)
C(11)	23(1)	26(1)	20(1)	-4(1)	3(1)	2(1)
C(12)	22(1)	24(1)	19(1)	1(1)	1(1)	-2(1)
C(13)	17(1)	20(1)	19(1)	1(1)	-4(1)	-2(1)
C(14)	24(1)	19(1)	24(1)	4(1)	-2(1)	-2(1)
C(15)	23(1)	17(1)	30(1)	1(1)	-3(1)	1(1)
C(16)	20(1)	15(1)	31(1)	-2(1)	4(1)	0(1)
C(17)	15(1)	17(1)	19(1)	0(1)	-3(1)	0(1)

Table S30. Hydrogen coordinates (x 10^4) and isotropic displacement parameters (Å^{2}x 10^3) for **(±)-4ad**.

	x	y	z	U(eq)
H(1)	8231	3265	7170	23
H(1A)	2020	-726	5721	38
H(1B)	2900	610	5817	38
H(1C)	1733	189	6529	38
H(3)	5701	-1724	7292	21
H(4)	6186	-1507	8762	24
H(5A)	4959	1083	8727	37
H(5B)	5675	431	9555	37
H(5C)	4153	-107	9186	37
H(6)	7929	-835	7788	22
H(7A)	7991	1483	8240	22
H(7B)	6524	1690	7693	22
H(10)	10227	1716	5521	25
H(11)	11578	2856	4484	28
H(12)	11548	5094	4422	26
H(14)	10511	7224	5008	27
H(15)	9139	8272	6060	28
H(16)	8017	7035	7134	26

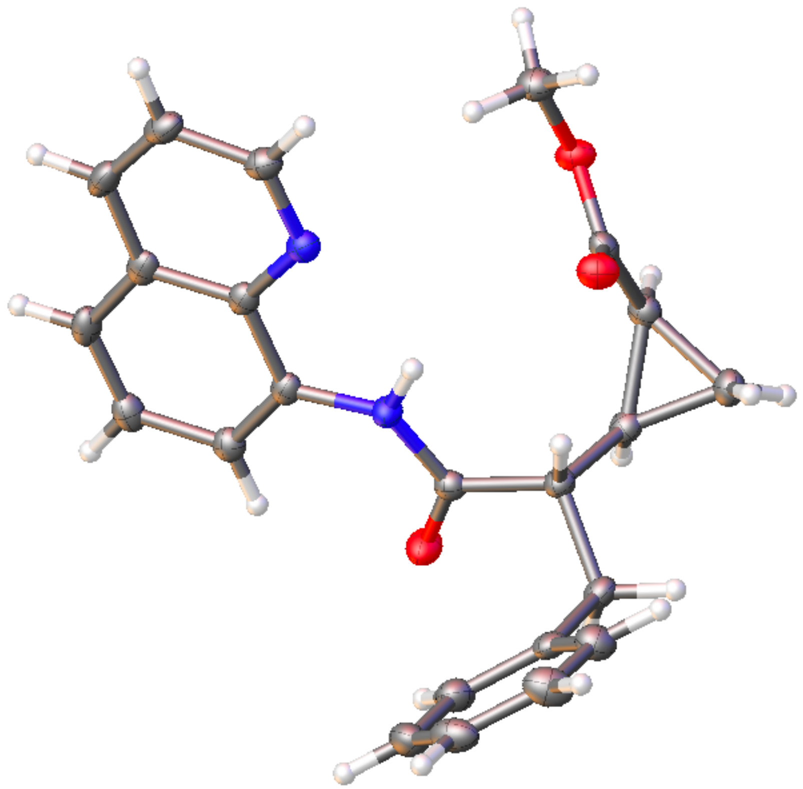

Experimental Summary for (±)-4ah

The single crystal X-ray diffraction studies were carried out on a Bruker SMART Pt 135 diffractometer equipped with Cu K_α radiation (λ = 1.54178 Å). Crystals of the subject compound were used as received (grown from DCM).

A 0.350 x 0.350 x 0.300 mm crystal was mounted on a Cryoloop with Paratone oil.

Data were collected in a nitrogen gas stream at 100(2) K using ϕ and ϖ scans. Crystal-to-detector distance was 45 mm using exposure time 2.0, 5.0 and 10.0s with a scan width of 1.25°. Data collection was 98.1 % complete to 67.679° in θ.

A total of 27745 reflections were collected. 3428 reflections were found to be symmetry independent, with a R_{int} of 0.0397. Indexing and unit cell refinement indicated a **Triclinic** lattice. The space group was found to be ***P*-1**. The data were integrated using the Bruker SAINT Software

program and scaled using the SADABS software program. Solution by direct methods (SHELXT) produced a complete phasing model consistent with the proposed structure.

All nonhydrogen atoms were refined anisotropically by full-matrix least-squares (SHELXL-2014). All carbon bonded hydrogen atoms were placed using a riding model. Their positions were constrained relative to their parent atom using the appropriate HFIX command in SHELXL-2014.

Crystallographic data are summarized in Table S31.

Notes: Excellent data and refinement
Centrosymmetric (Racemic) space group

Table S31. Crystal data and structure refinement for (±)-**4ah** (CCDC 2367118).

Identification code	engle604	
Empirical formula	C23 H22 N2 O3	
Formula weight	374.42	
Temperature	100.15 K	
Wavelength	1.54178 Å	
Crystal system	Triclinic	
Space group	P-1	
Unit cell dimensions	a = 8.0823(7) Å	α= 84.354(2)°.
	b = 10.9527(10) Å	β= 74.498(2)°.
	c = 11.3301(10) Å	γ = 80.946(2)°.
Volume	952.77(15) Å^3	
Z	2	
Density (calculated)	1.305 Mg/m^3	
Absorption coefficient	0.701 mm^{-1}	
F(000)	396	
Crystal size	0.35 x 0.35 x 0.3 mm^3	
Theta range for data collection	4.056 to 68.235°.	
Index ranges	-9<=h<=9, -13<=k<=13, -13<=l<=13	
Reflections collected	27745	
Independent reflections	3428 [R(int) = 0.0397]	
Completeness to theta = 67.679°	98.1 %	
Absorption correction	Semi-empirical from equivalents	
Max. and min. transmission	0.6617 and 0.5856	
Refinement method	Full-matrix least-squares on F^2	
Data / restraints / parameters	3428 / 0 / 257	
Goodness-of-fit on F^2	1.069	
Final R indices [I>2sigma(I)]	R1 = 0.0359, wR2 = 0.0915	
R indices (all data)	R1 = 0.0364, wR2 = 0.0919	
Largest diff. peak and hole	0.301 and -0.156 e.Å^{-3}	

Table S32. Atomic coordinates (x 10^4) and equivalent isotropic displacement parameters ($Å^2$x 10^3) for **(±)-4ah**. U(eq) is defined as one third of the trace of the orthogonalized U^{ij} tensor.

	x	y	z	U(eq)
O(1)	7481(1)	5343(1)	5952(1)	26(1)
N(1)	6234(1)	6729(1)	7420(1)	19(1)
C(1)	7435(1)	6330(1)	6388(1)	18(1)
O(2)	8773(1)	8987(1)	7792(1)	25(1)
N(2)	4446(1)	7796(1)	9473(1)	20(1)
C(2)	8796(1)	7194(1)	5834(1)	18(1)
O(3)	9424(1)	7778(1)	9374(1)	24(1)
C(3)	10411(1)	6682(1)	6267(1)	20(1)
C(4)	11796(2)	7463(1)	6164(1)	25(1)
C(5)	10738(1)	7083(1)	7433(1)	21(1)
C(6)	9540(1)	8056(1)	8176(1)	20(1)
C(7)	9224(1)	7249(1)	4422(1)	21(1)
C(8)	7763(1)	7958(1)	3926(1)	21(1)
C(9)	6372(2)	7393(1)	3838(1)	26(1)
C(10)	5005(2)	8077(1)	3429(1)	30(1)
C(11)	5004(2)	9331(1)	3103(1)	32(1)
C(12)	6390(2)	9900(1)	3173(1)	34(1)
C(13)	7757(2)	9216(1)	3582(1)	28(1)
C(14)	8323(2)	8667(1)	10202(1)	26(1)
C(15)	4933(1)	6092(1)	8195(1)	18(1)
C(16)	4532(1)	4976(1)	7970(1)	21(1)
C(17)	3213(1)	4413(1)	8834(1)	24(1)
C(18)	2313(1)	4954(1)	9900(1)	24(1)
C(19)	2685(1)	6106(1)	10149(1)	21(1)
C(20)	1797(2)	6740(1)	11220(1)	26(1)
C(21)	2227(2)	7855(1)	11382(1)	26(1)
C(22)	3576(2)	8342(1)	10487(1)	23(1)
C(23)	4002(1)	6687(1)	9296(1)	19(1)

Table S33. Bond lengths [Å] and angles [°] for **(±)-4ah**.

O(1)-C(1)	1.2234(13)	C(15)-C(16)	1.3758(16)
N(1)-H(1)	0.875(15)	C(15)-C(23)	1.4321(15)
N(1)-C(1)	1.3642(14)	C(16)-H(16)	0.9500
N(1)-C(15)	1.4079(14)	C(16)-C(17)	1.4161(16)
C(1)-C(2)	1.5296(15)	C(17)-H(17)	0.9500
O(2)-C(6)	1.2129(13)	C(17)-C(18)	1.3690(17)
N(2)-C(22)	1.3204(15)	C(18)-H(18)	0.9500
N(2)-C(23)	1.3663(15)	C(18)-C(19)	1.4126(17)
C(2)-H(2)	1.0000	C(19)-C(20)	1.4190(16)
C(2)-C(3)	1.5219(15)	C(19)-C(23)	1.4202(16)
C(2)-C(7)	1.5414(14)	C(20)-H(20)	0.9500
O(3)-C(6)	1.3431(14)	C(20)-C(21)	1.3637(18)
O(3)-C(14)	1.4434(13)	C(21)-H(21)	0.9500
C(3)-H(3)	1.0000	C(21)-C(22)	1.4093(17)
C(3)-C(4)	1.4870(15)	C(22)-H(22)	0.9500
C(3)-C(5)	1.5316(15)		
C(4)-H(4A)	0.9900	C(1)-N(1)-H(1)	118.1(9)
C(4)-H(4B)	0.9900	C(1)-N(1)-C(15)	127.81(9)
C(4)-C(5)	1.5193(16)	C(15)-N(1)-H(1)	113.8(9)
C(5)-H(5)	1.0000	O(1)-C(1)-N(1)	123.64(10)
C(5)-C(6)	1.4825(15)	O(1)-C(1)-C(2)	121.73(9)
C(7)-H(7A)	0.9900	N(1)-C(1)-C(2)	114.57(9)
C(7)-H(7B)	0.9900	C(22)-N(2)-C(23)	117.24(10)
C(7)-C(8)	1.5137(15)	C(1)-C(2)-H(2)	109.6
C(8)-C(9)	1.3962(17)	C(1)-C(2)-C(7)	110.39(9)
C(8)-C(13)	1.3934(17)	C(3)-C(2)-C(1)	107.54(8)
C(9)-H(9)	0.9500	C(3)-C(2)-H(2)	109.6
C(9)-C(10)	1.3930(17)	C(3)-C(2)-C(7)	110.13(9)
C(10)-H(10)	0.9500	C(7)-C(2)-H(2)	109.6
C(10)-C(11)	1.3870(19)	C(6)-O(3)-C(14)	115.81(9)
C(11)-H(11)	0.9500	C(2)-C(3)-H(3)	114.2
C(11)-C(12)	1.3881(19)	C(2)-C(3)-C(5)	122.64(9)
C(12)-H(12)	0.9500	C(4)-C(3)-C(2)	120.70(9)
C(12)-C(13)	1.3929(17)	C(4)-C(3)-H(3)	114.2
C(13)-H(13)	0.9500	C(4)-C(3)-C(5)	60.42(7)
C(14)-H(14A)	0.9800	C(5)-C(3)-H(3)	114.2
C(14)-H(14B)	0.9800	C(3)-C(4)-H(4A)	117.6
C(14)-H(14C)	0.9800	C(3)-C(4)-H(4B)	117.6

C(3)-C(4)-C(5)	61.24(7)
H(4A)-C(4)-H(4B)	114.7
C(5)-C(4)-H(4A)	117.6
C(5)-C(4)-H(4B)	117.6
C(3)-C(5)-H(5)	115.5
C(4)-C(5)-C(3)	58.34(7)
C(4)-C(5)-H(5)	115.5
C(6)-C(5)-C(3)	121.70(9)
C(6)-C(5)-C(4)	118.23(10)
C(6)-C(5)-H(5)	115.5
O(2)-C(6)-O(3)	123.22(10)
O(2)-C(6)-C(5)	126.70(10)
O(3)-C(6)-C(5)	110.06(9)
C(2)-C(7)-H(7A)	109.1
C(2)-C(7)-H(7B)	109.1
H(7A)-C(7)-H(7B)	107.8
C(8)-C(7)-C(2)	112.59(9)
C(8)-C(7)-H(7A)	109.1
C(8)-C(7)-H(7B)	109.1
C(9)-C(8)-C(7)	121.58(10)
C(13)-C(8)-C(7)	120.07(10)
C(13)-C(8)-C(9)	118.32(11)
C(8)-C(9)-H(9)	119.7
C(10)-C(9)-C(8)	120.60(11)
C(10)-C(9)-H(9)	119.7
C(9)-C(10)-H(10)	119.7
C(11)-C(10)-C(9)	120.53(12)
C(11)-C(10)-H(10)	119.7
C(10)-C(11)-H(11)	120.3
C(10)-C(11)-C(12)	119.38(12)
C(12)-C(11)-H(11)	120.3
C(11)-C(12)-H(12)	120.0
C(11)-C(12)-C(13)	120.06(12)
C(13)-C(12)-H(12)	120.0
C(8)-C(13)-H(13)	119.4
C(12)-C(13)-C(8)	121.11(11)
C(12)-C(13)-H(13)	119.4
O(3)-C(14)-H(14A)	109.5
O(3)-C(14)-H(14B)	109.5
O(3)-C(14)-H(14C)	109.5
H(14A)-C(14)-H(14B)	109.5
H(14A)-C(14)-H(14C)	109.5
H(14B)-C(14)-H(14C)	109.5
N(1)-C(15)-C(23)	114.89(9)
C(16)-C(15)-N(1)	125.18(10)
C(16)-C(15)-C(23)	119.92(10)
C(15)-C(16)-H(16)	120.0
C(15)-C(16)-C(17)	119.92(10)
C(17)-C(16)-H(16)	120.0
C(16)-C(17)-H(17)	119.3
C(18)-C(17)-C(16)	121.48(11)
C(18)-C(17)-H(17)	119.3
C(17)-C(18)-H(18)	120.1
C(17)-C(18)-C(19)	119.84(11)
C(19)-C(18)-H(18)	120.1
C(18)-C(19)-C(20)	123.52(11)
C(18)-C(19)-C(23)	119.64(10)
C(20)-C(19)-C(23)	116.83(11)
C(19)-C(20)-H(20)	120.2
C(21)-C(20)-C(19)	119.68(11)
C(21)-C(20)-H(20)	120.2
C(20)-C(21)-H(21)	120.5
C(20)-C(21)-C(22)	118.93(11)
C(22)-C(21)-H(21)	120.5
N(2)-C(22)-C(21)	124.11(11)
N(2)-C(22)-H(22)	117.9
C(21)-C(22)-H(22)	117.9
N(2)-C(23)-C(15)	117.62(10)
N(2)-C(23)-C(19)	123.19(10)
C(19)-C(23)-C(15)	119.19(10)

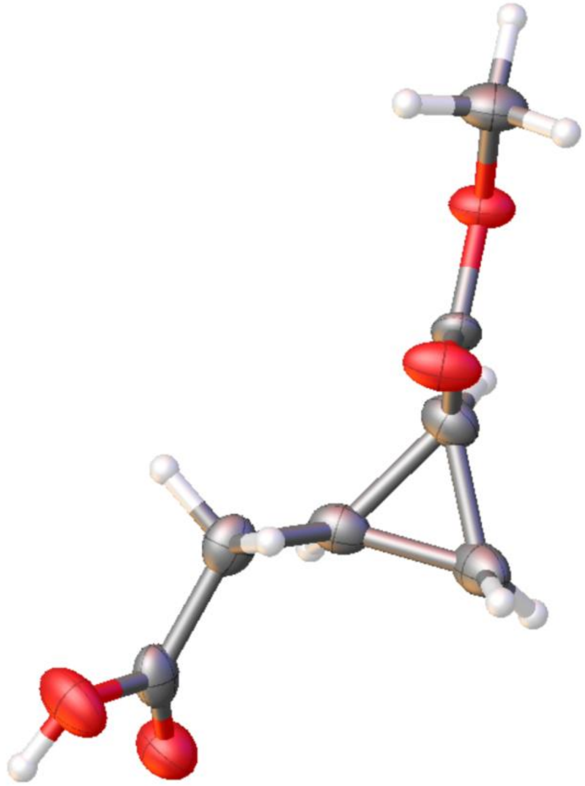

Experimental Summary for (±)-6aa

The single crystal X-ray diffraction studies were carried out on a “Bruker APEX-II CCD” 3-circle diffractometer equipped with Mo K_α radiation (λ = 0.7107 Å).

Crystals of the subject compound were used as received. A 0.2 x 0.1 x 0.07 mm piece of a crystal was mounted on a glass capillary. Data were collected in a nitrogen gas stream at 173(2) K using ω scans. Crystal-to-detector distance was 40 mm and exposure time was 10 seconds per frame using a scan width of 1.5°. Data collection was 99.7 % complete to 25.0 in θ. A total of 5943 reflections were collected covering the indices, −6<=h<=7, −7<=k<=7, −6<=l<=12. 1600 reflections were found to be symmetry independent, with a R_{int} of 0.0731. Indexing and unit cell refinement indicated a **P**rimitive, **Triclinic** lattice. The space group was found to be $\boldsymbol{P}\bar{1}$. The data were integrated using the Bruker SAINT Software program and scaled

using the SADABS software program. Solution by direct methods (SHELXT) produced a complete phasing model consistent with the proposed structure.

All nonhydrogen atoms were refined anisotropically by full-matrix least-squares (SHELXL-2016). All hydrogen atoms were placed using a riding model. Their positions were constrained relative to their parent atom using the appropriate HFIX command in SHELXL-2016. Crystallographic data are summarized in Table 34.

Attention! The structure was refined as a two-component twin with ratio of components 34 : 66 %. Twinning causes high enough R-factor and low precision on C-C bonds.

The chemical formula for the compound is: $C_7H_{10}O_4$

Table 34. Crystal data and structure refinement for **(±)-6aa (**CCDC 2394329)

Identification code	vd510	
Empirical formula	C7 H10 O4	
Formula weight	158.15	
Temperature	172.65 K	
Wavelength	0.71073 Å	
Crystal system	Triclinic	
Space group	P-1	
Unit cell dimensions	a = 5.9719(19) Å	α= 76.78(2)°.
	b = 6.108(2) Å	β= 84.19(2)°.
	c = 10.902(3) Å	γ= 87.80(2)°.
Volume	385.1(2) $Å^3$	
Z	2	
Density (calculated)	1.364 Mg/m^3	
Absorption coefficient	0.113 mm^{-1}	
F(000)	168	
Crystal size	0.2 x 0.1 x 0.07 mm^3	
Theta range for data collection	1.928 to 24.992°.	
Index ranges	-6<=h<=7, -7<=k<=7, -6<=l<=12	
Reflections collected	1353	
Independent reflections	1353 [R(int) = ?]	
Completeness to theta = 24.992°	99.7 %	
Absorption correction	None	
Refinement method	Full-matrix least-squares on F^2	
Data / restraints / parameters	1353 / 0 / 101	
Goodness-of-fit on F^2	1.259	
Final R indices [I>2sigma(I)]	R1 = 0.1159, wR2 = 0.3393	
R indices (all data)	R1 = 0.1411, wR2 = 0.3510	
Extinction coefficient	n/a	
Largest diff. peak and hole	0.546 and -0.607 e.$Å^{-3}$	

Table 35. Atomic coordinates (x 10^4) and equivalent isotropic displacement parameters ($Å^2$x 10^3) for **(±)-6aa**. U(eq) is defined as one third of the trace of the orthogonalized U^{ij} tensor.

	x	y	z	U(eq)
O(1)	4242(9)	7631(9)	5899(5)	40(2)
O(2)	7808(10)	8482(10)	5199(6)	47(2)
O(3)	9873(9)	1644(9)	8348(6)	38(2)
O(4)	8468(8)	-1710(8)	8398(5)	31(1)
C(1)	5955(13)	1248(13)	8226(7)	31(2)
C(2)	5174(14)	3319(13)	8670(8)	37(2)
C(3)	5348(12)	3410(12)	7296(7)	30(2)
C(4)	7124(13)	4825(13)	6400(7)	31(2)
C(5)	6223(13)	7103(13)	5831(7)	32(2)
C(6)	8296(12)	481(11)	8322(7)	24(2)
C(7)	10726(14)	-2700(13)	8513(9)	38(2)

Table 36. Bond lengths [Å] and angles [°] for **(±)-6aa**.

O(1)-C(5)	1.214(9)	C(1)-C(2)-H(2B)	117.6
O(2)-H(2)	0.8400	H(2A)-C(2)-H(2B)	114.7
O(2)-C(5)	1.320(10)	C(3)-C(2)-C(1)	61.7(5)
O(3)-C(6)	1.207(8)	C(3)-C(2)-H(2A)	117.6
O(4)-C(6)	1.322(8)	C(3)-C(2)-H(2B)	117.6
O(4)-C(7)	1.462(9)	C(1)-C(3)-H(3)	114.6
C(1)-H(1)	1.0000	C(2)-C(3)-C(1)	59.7(5)
C(1)-C(2)	1.497(11)	C(2)-C(3)-H(3)	114.6
C(1)-C(3)	1.526(11)	C(2)-C(3)-C(4)	120.8(6)
C(1)-C(6)	1.463(11)	C(4)-C(3)-C(1)	121.8(6)
C(2)-H(2A)	0.9900	C(4)-C(3)-H(3)	114.6
C(2)-H(2B)	0.9900	C(3)-C(4)-H(4A)	109.3
C(2)-C(3)	1.480(12)	C(3)-C(4)-H(4B)	109.3
C(3)-H(3)	1.0000	H(4A)-C(4)-H(4B)	108.0
C(3)-C(4)	1.517(10)	C(5)-C(4)-C(3)	111.5(7)
C(4)-H(4A)	0.9900	C(5)-C(4)-H(4A)	109.3
C(4)-H(4B)	0.9900	C(5)-C(4)-H(4B)	109.3
C(4)-C(5)	1.492(11)	O(1)-C(5)-O(2)	123.0(7)
C(7)-H(7A)	0.9800	O(1)-C(5)-C(4)	124.2(7)
C(7)-H(7B)	0.9800	O(2)-C(5)-C(4)	112.8(7)
C(7)-H(7C)	0.9800	O(3)-C(6)-O(4)	123.6(7)
C(5)-O(2)-H(2)	109.5	O(3)-C(6)-C(1)	125.9(6)
C(6)-O(4)-C(7)	116.1(6)	O(4)-C(6)-C(1)	110.5(6)
C(2)-C(1)-H(1)	115.3	O(4)-C(7)-H(7A)	109.5
C(2)-C(1)-C(3)	58.6(5)	O(4)-C(7)-H(7B)	109.5
C(3)-C(1)-H(1)	115.3	O(4)-C(7)-H(7C)	109.5
C(6)-C(1)-H(1)	115.3	H(7A)-C(7)-H(7B)	109.5
C(6)-C(1)-C(2)	119.4(7)	H(7A)-C(7)-H(7C)	109.5
C(6)-C(1)-C(3)	121.3(6)	H(7B)-C(7)-H(7C)	109.5
C(1)-C(2)-H(2A)	117.6		

Symmetry transformations used to generate equivalent atoms:

References

1. Fang, W.-Y., Wang, S.-M., Zhang, Z.-W.& Qin, H.-L. Clickable Transformation of Nitriles (RCN) to Oxazolyl Sulfonyl Fluoride Warheads. *Org. Lett.* **22**, 8904–8909 (2020).

2. Gordeev, M. F., Gordon, E. M. & Patel, D. V. Solid-Phase Synthesis of β-Sultams. *J. Org. Chem.* **62**, 8177–8181 (1997).

3. Dong, J., Krasnova, L., Finn, M. G.& Sharpless, K. B. Sulfur(VI) Fluoride Exchange (SuFEx): Another Good Reaction for Click Chemistry. *Angew. Chem., Int. Ed.* **53**, 9430– 9448 (2014).

4. Ni, H.-Q. et al. *Anti*-selective Cyclopropanation of Nonconjugated Alkenes with Diverse Pronucleophiles via Directed Nucleopalladation. *J. Am. Chem. Soc.* **146**, 24503–24514 (2024).

5. Christe, K. O. & Wilson, W. W. Nuclear magnetic resonance spectrum of the fluoride anion. *J. Fluor. Chem.* **46,** 339–342 (1990).

6. Frisch, M. J. et al. Gaussian 16 Rev. C.01 (Wallingford, CT, 2016).

7. Grimme, S., Antony, J., Ehrlich, S. & Krieg, H. A Consistent and Accurate Ab Initio Parametrization of Density Functional Dispersion Correction (DFT-D) for the 94 Elements H-Pu. *J. Chem. Phys*. **132**, 154104 (2010).

8. Grimme, S., Ehrlich, S. & Goerigk, L. Effect of the Damping Function in Dispersion Corrected Density Functional Theory. *J. Comput. Chem.* **32**, 1456–1465 (2011).

9. Lee, C., Yang, W. & Parr, R. G. Development of the Colle-Salvetti Correlation-Energy Formula into a Functional of the Electron Density. *Phys. Rev. B* **37**, 785–789 (1988).

10. Becke, A. D. Density-functional Thermochemistry. III. The Role of Exact Exchange. *J. Chem. Phys.* **98**, 5648–5652 (1993).

11. Andrae, D., Häußermann, U., Dolg, M., Stoll, H. & Preuß, H. Energy-Adjustedab Initio Pseudopotentials for the Second and Third Row Transition Elements. *Theor. Chim. Acta*, **77**, 123–141 (1990).

12. Martin, J. M. L. & Sundermann, A. Correlation Consistent Valence Basis Sets for Use with the Stuttgart–Dresden–Bonn Relativistic Effective Core Potentials: The Atoms Ga–Kr and In–Xe. *J. Chem. Phys.* **114**, 3408–3420 (2001).

13. Chai, J.-D. & Head-Gordon, M. Long-Range Corrected Hybrid Density Functionals with Damped Atom–Atom Dispersion Corrections. *Phys. Chem. Chem. Phys.* **10**, 6615–6620 (2008).

14. Chai, J.-D. & Head-Gordon, M. Systematic Optimization of Long-Range Corrected Hybrid Density Functionals. *J. Chem. Phys.* **128**, 084106 (2008).

15. Weigend, F. & Ahlrichs, R. Balanced Basis Sets of Split Valence, Triple Zeta Valence and Quadruple Zeta Valence Quality for H to Rn: Design and Assessment of Accuracy. *Phys. Chem. Chem. Phys.* **7**, 3297–3305 (2005).

16. Marenich, A. V., Cramer, C. J. & Truhlar, D. G. Universal Solvation Model Based on Solute Electron Density and on a Continuum Model of the Solvent Defined by the Bulk Dielectric Constant and Atomic Surface Tensions. *J. Phys. Chem. B* **113**, 6378–6396 (2009).

17. Riplinger, C. & Neese, F. An Efficient and near Linear Scaling Pair Natural Orbital Based Local Coupled Cluster Method. *J. Chem. Phys.* **138**, 034106 (2013).

18. Riplinger, C., Sandhoefer, B., Hansen, A. & Neese, F. Natural Triple Excitations in Local Coupled Cluster Calculations with Pair Natural Orbitals. *J. Chem. Phys.* **139**, 134101 (2013).

19. Liakos, D. G., Sparta, M., Kesharwani, M. K., Martin, J. M. L. & Neese, F. Exploring the Accuracy Limits of Local Pair Natural Orbital Coupled-Cluster Theory. *J. Chem. Theory Comput.* **11**, 1525–1539 (2015).

20. Liakos, D. G. & Neese, F. Is It Possible To Obtain Coupled Cluster Quality Energies at near Density Functional Theory Cost? Domain-Based Local Pair Natural Orbital Coupled Cluster vs Modern Density Functional Theory. *J. Chem. Theory Comput.* **11**, 4054–4063 (2015).

21. Neese, F., Wennmohs, F., Becker, U. & Riplinger, C. The ORCA Quantum Chemistry Program Package. *J. Chem. Phys.* **152**, 224108 (2020).

22. Neese, F., Wennmohs, F., Hansen, A. & Becker, U. Efficient, Approximate and Parallel Hartree–Fock and Hybrid DFT Calculations. A 'Chain-of-Spheres' Algorithm for the Hartree–Fock Exchange. *Mov. Front. Quantum Chem.* **356**, 98–109 (2009).

23. Mammen, M., Shakhnovich, E. I., Deutch, J. M. & Whitesides, G. M. Estimating the Entropic Cost of Self-Assembly of Multiparticle Hydrogen-Bonded Aggregates Based on the Cyanuric Acid·Melamine Lattice. *J. Org. Chem.* **63**, 3821–3830 (1998).

24. Luchini, G., Alegre-Requena, J., Funes-Ardoiz, I. & Paton, R. GoodVibes: Automated Thermochemistry for Heterogeneous Computational Chemistry Data. *F1000Research* **9**, 291(2020).

25. Goumont, R., Magnier, E., Kizilian, E. & Terrier, F. Acidity inversions of α-NO_2 and α-SO_2CF_3 activated carbon acids as a result of contrasting solvent effects on transfer from water to dimethyl sulfoxide solutions. *The Journal of Organic Chemistry* **68**, 6566–6570 (2003).

26. Wagen, C. C. & Wagen, A. M. Efficient and Accurate pKa Prediction Enabled by Pre-Trained Machine-Learned Interatomic Potentials. *ChemRxiv* (2024). DOI: https://doi.org/10.26434/chemrxiv-2024-8489b.

27. Anstine, D. M., Zubatyuk, R. & Isayev, O. AIMNet2: A Neural Network Potential to Meet Your Neutral, Charged, Organic, and Elemental-Organic Needs. *ChemRxiv* (2024), DOI: 10.26434/chemrxiv-2023-296ch-v2.

28. CCDC 2171740 (**(±)-3aa**), CCDC 2345363 (**(±)-3aj**), CCDC 2351469 (**(±)-3al'**), CCDC 2345089 (**(±)-4aa**), CCDC 2376390 (**(±)-4ad**), CCDC 2367118 (**(±)-4ah**), CCDC 2394329 (**(±)-6aa**) and CCDC 2171739 (**Pd-3aa**) contains the supplementary crystallographic data for this paper. These data can be obtained free of charge from The Cambridge Crystallographic Data Centre via www.ccdc.cam.ac.uk/data_request/cif. The software Olex2 has been used for the analysis.

NMR Spectra

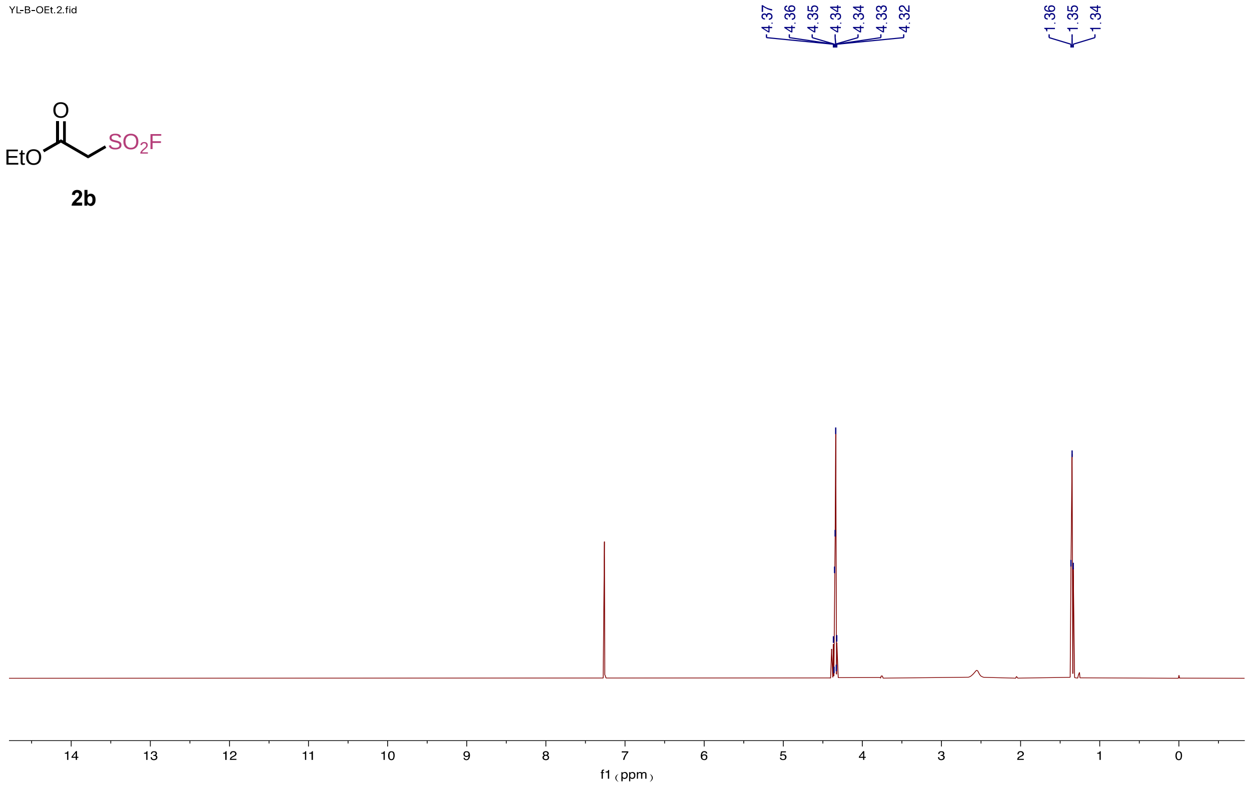
YL-B-OEt.2.fid
4.37
4.36
4.35
4.34
4.34
4.33
4.32
1.36
1.35
1.34
EtO
O
SO2F
2b
14 13 12 11 10 9 8 7 6 5 4 3 2 1 0
f1 (ppm)

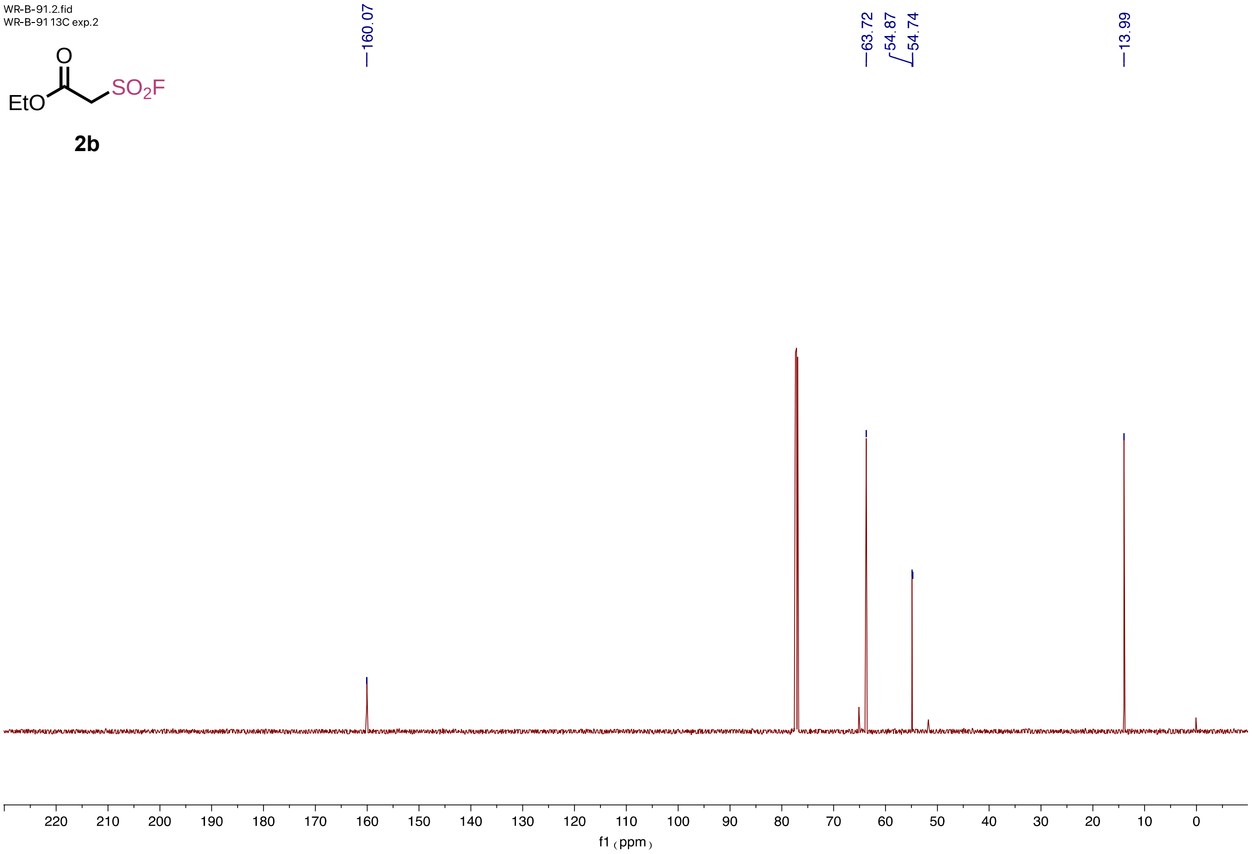
WR-B-91.2.fid
WR-B-91 13C exp.2
160.07
63.72
54.87
54.74
13.99
EtO
O
SO2F
2b
220 210 200 190 180 170 160 150 140 130 120 110 100 90 80 70 60 50 40 30 20 10 0
f1 (ppm)

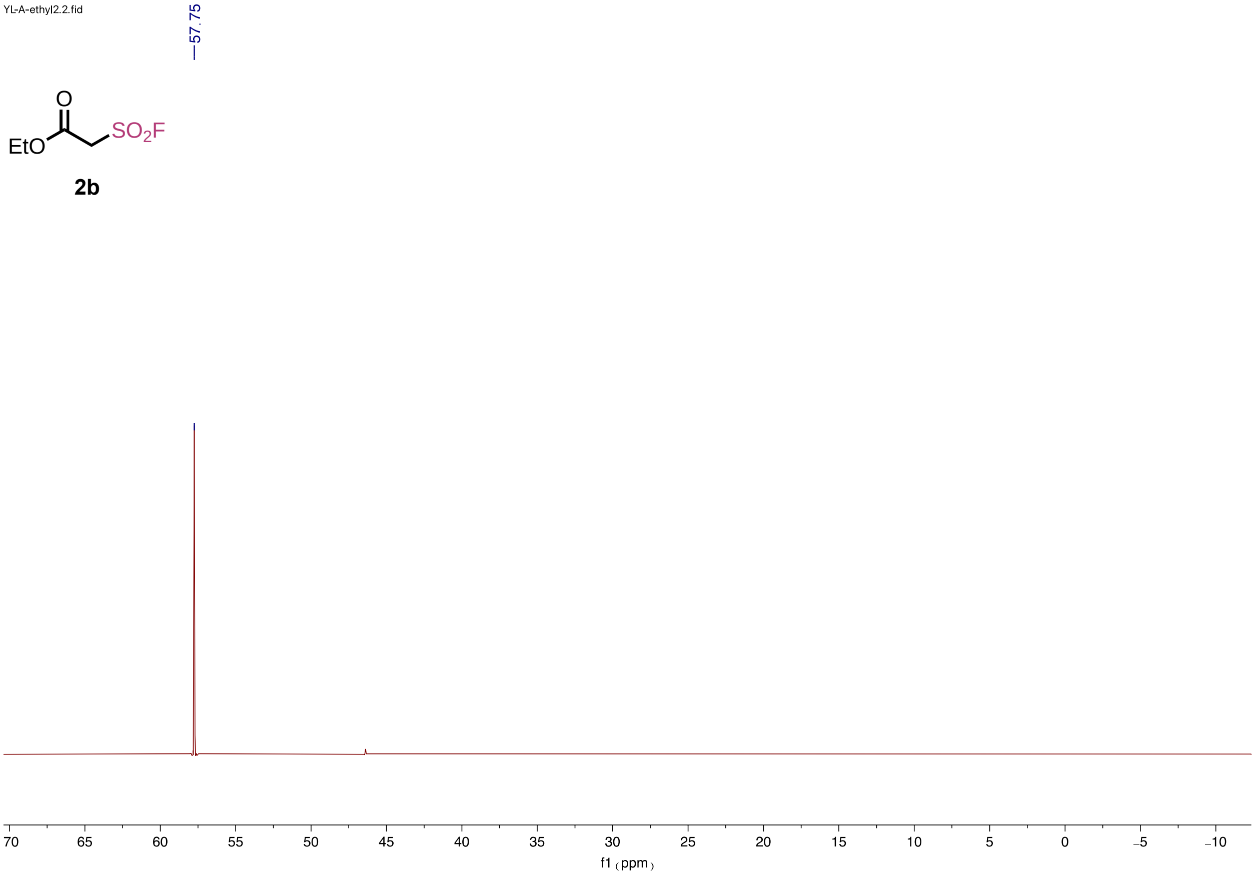
YL-A-ethyl2.2.fid
57.75
O
EtO
SO2F
2b
70 65 60 55 50 45 40 35 30 25 20 15 10 5 0 -5 -10
f1 (ppm)

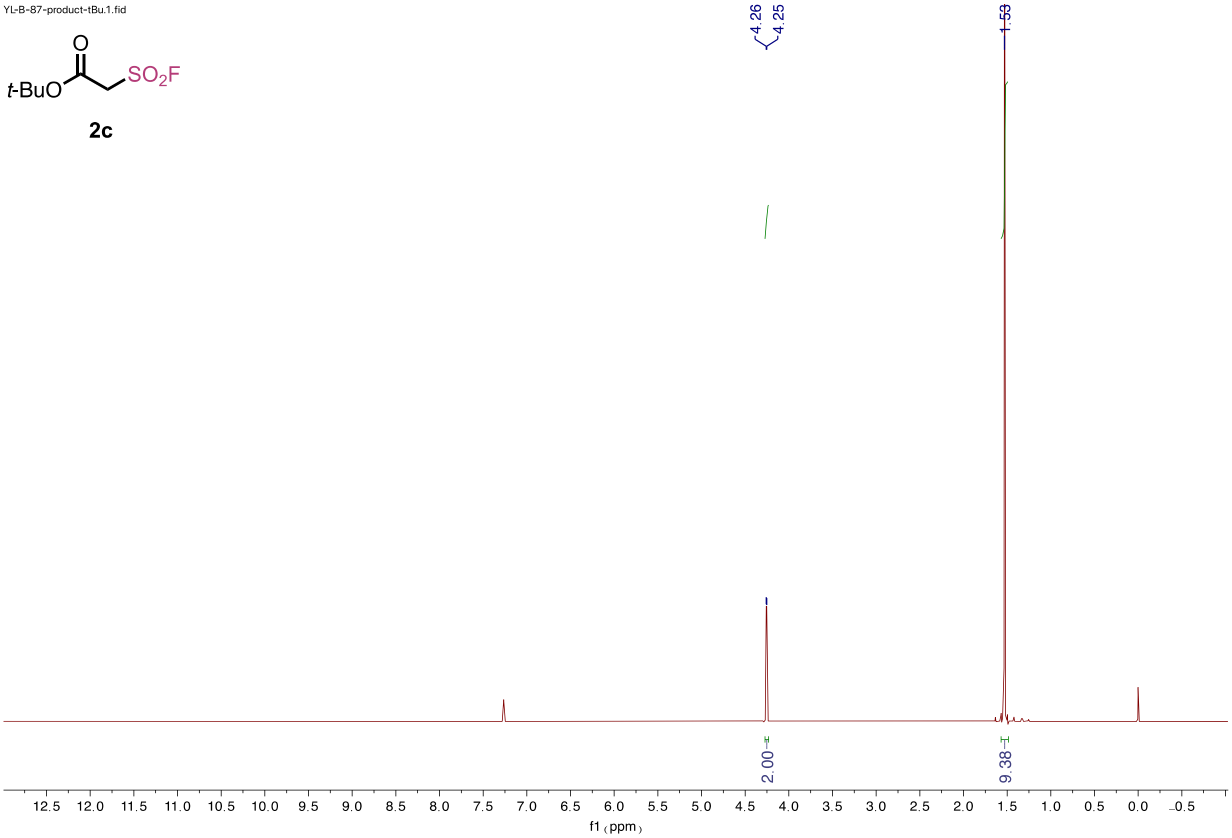
YL-B-87-product-tBu.1.fid
4.26
4.25
1.53
O
t-BuO
SO2F
2c
2.00
9.38
12.5 12.0 11.5 11.0 10.5 10.0 9.5 9.0 8.5 8.0 7.5 7.0 6.5 6.0 5.5 5.0 4.5 4.0 3.5 3.0 2.5 2.0 1.5 1.0 0.5 0.0 -0.5
f1 (ppm)

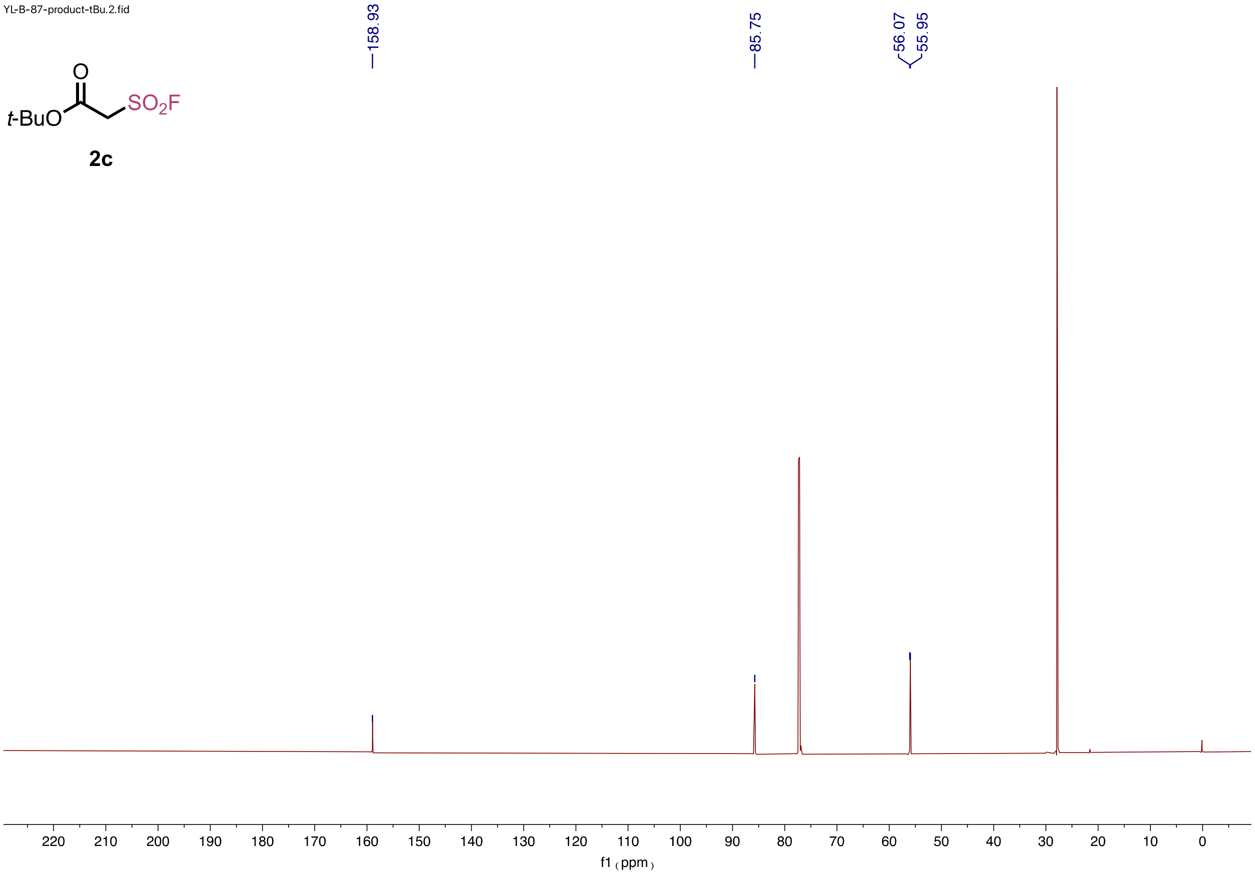
YL-B-87-product-tBu.2.fid
158.93
85.75
56.07
55.95
t-BuO
O
SO2F
2c
220 210 200 190 180 170 160 150 140 130 120 110 100 90 80 70 60 50 40 30 20 10 0
f1 (ppm)

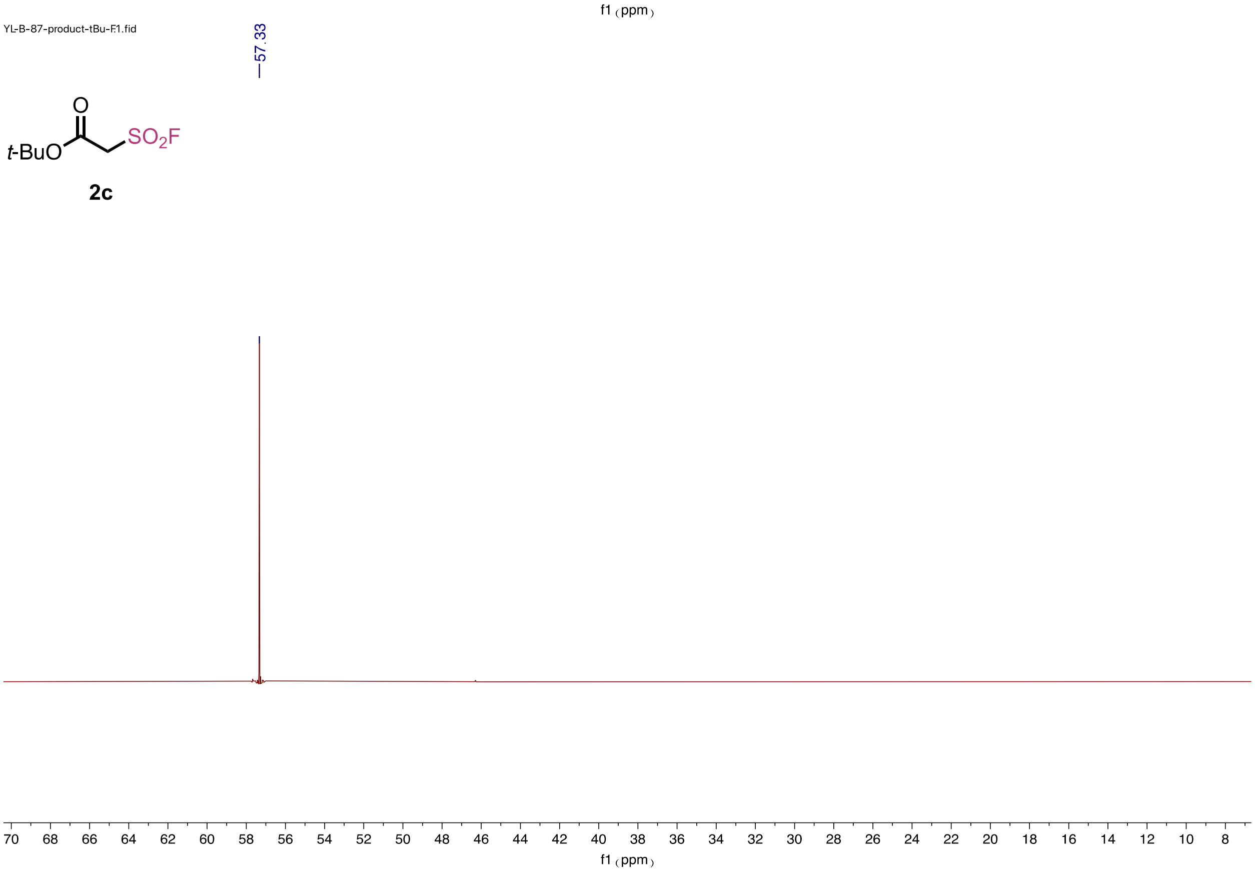
YL-B-87-product-tBu-F1.fid
-57.33
t-BuO
O
SO2F
2c
70 68 66 64 62 60 58 56 54 52 50 48 46 44 42 40 38 36 34 32 30 28 26 24 22 20 18 16 14 12 10 8
f1 (ppm)

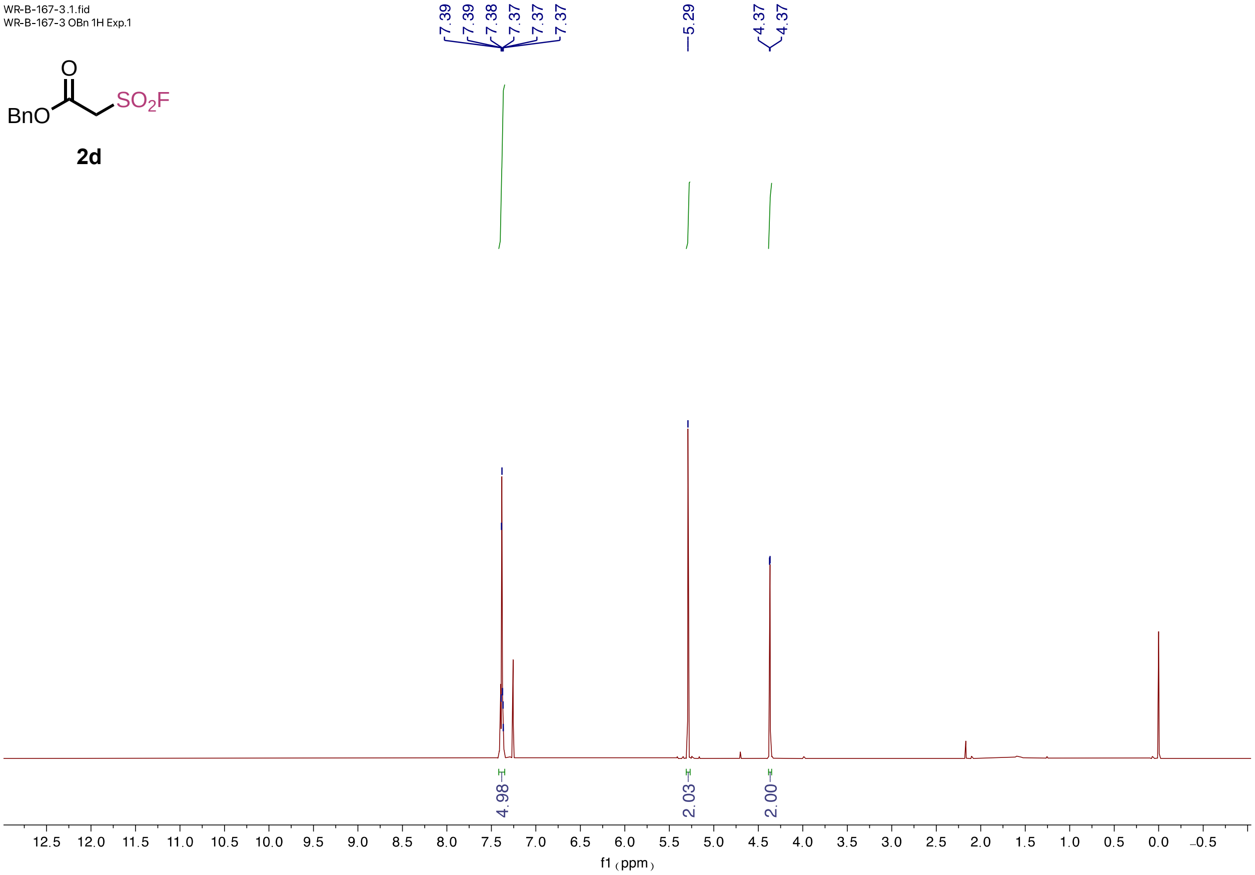
WR-B-167-3.1.fid
WR-B-167-3 OBn 1H Exp.1
BnO
O
SO2F
2d
7.39
7.39
7.38
7.37
7.37
7.37
5.29
4.37
4.37
4.98
2.03
2.00
12.5 12.0 11.5 11.0 10.5 10.0 9.5 9.0 8.5 8.0 7.5 7.0 6.5 6.0 5.5 5.0 4.5 4.0 3.5 3.0 2.5 2.0 1.5 1.0 0.5 0.0 -0.5
f1 (ppm)

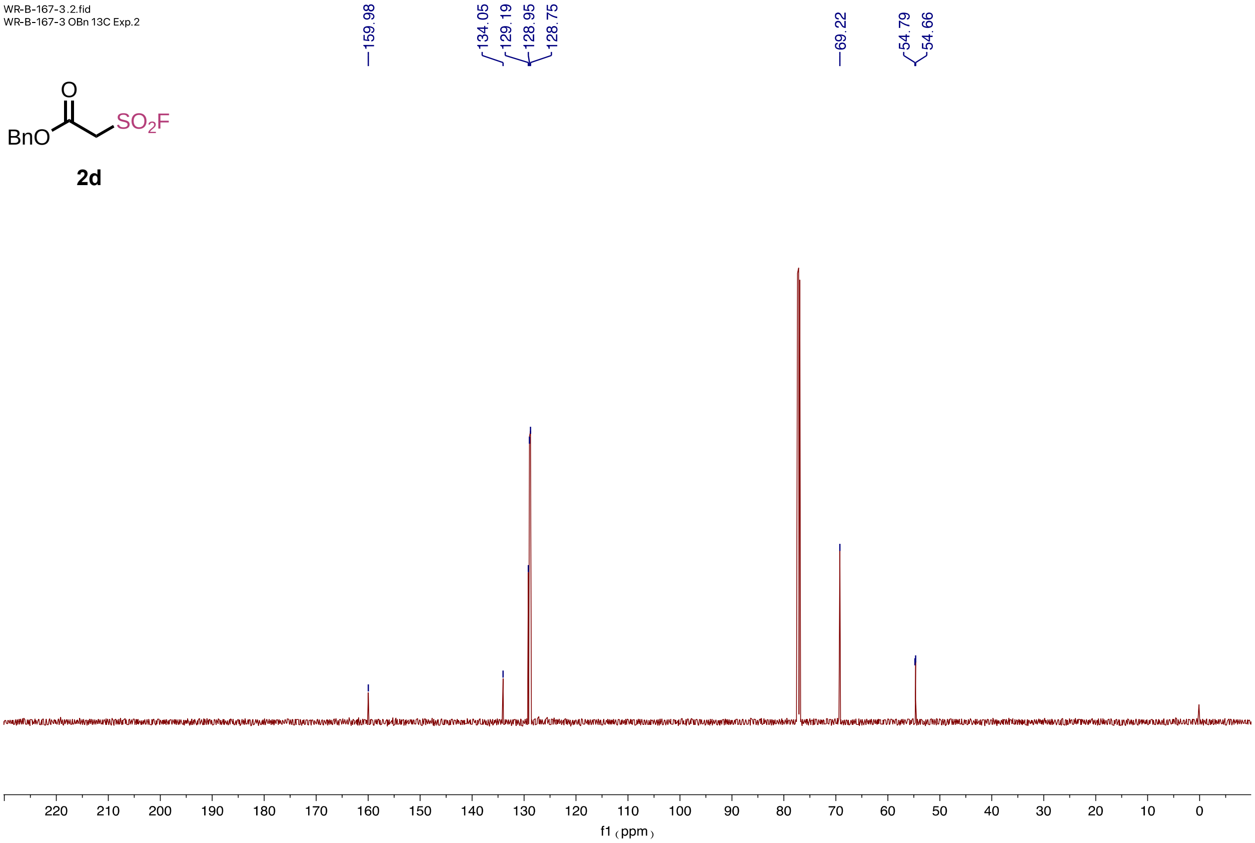
WR-B-167-3.2.fid
WR-B-167-3 OBn 13C Exp.2
BnO
O
SO2F
2d
159.98
134.05
129.19
128.95
128.75
69.22
54.79
54.66
220 210 200 190 180 170 160 150 140 130 120 110 100 90 80 70 60 50 40 30 20 10 0
f1 (ppm)

WR-B-167-3.4.fid
WR-B-167 F1 19F exp.4

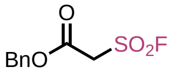

2d

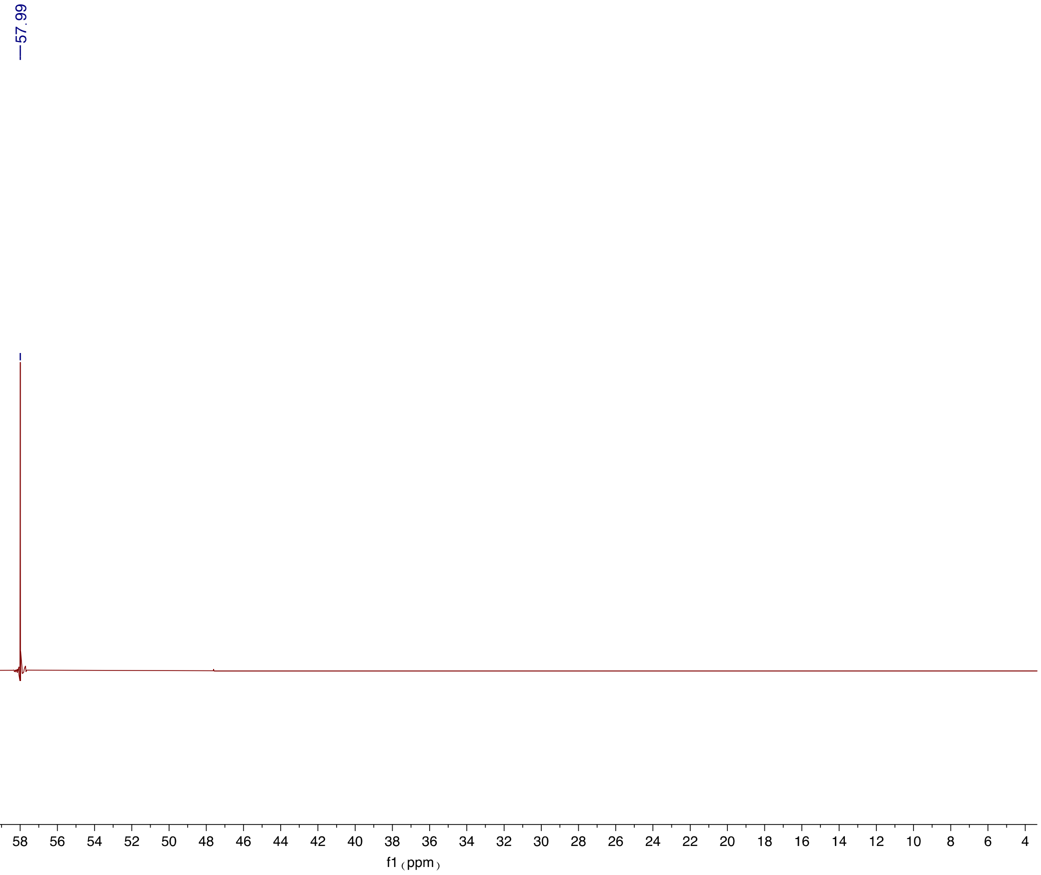

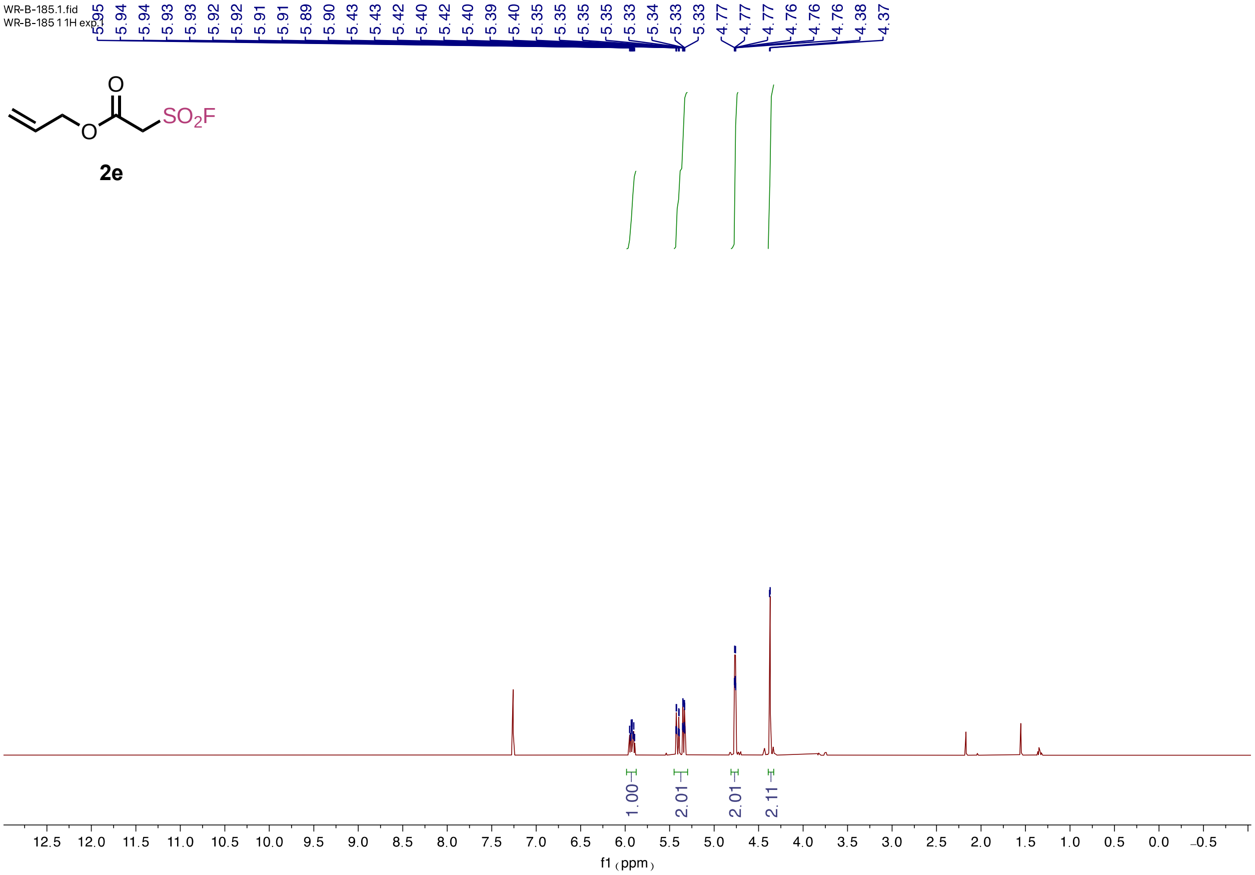
WR-B-185.1.fid
WR-B-185 1 1H exp.1
5.95 5.94 5.94 5.93 5.93 5.92 5.92 5.91 5.91 5.89 5.90 5.43 5.42 5.40 5.42 5.40 5.39 5.40 5.35 5.35 5.35 5.35 5.33 5.34 5.33 5.33 4.77 4.77 4.77 4.76 4.76 4.76 4.38 4.37
O
O
SO2F
2e
1.00 2.01 2.01 2.11
12.5 12.0 11.5 11.0 10.5 10.0 9.5 9.0 8.5 8.0 7.5 7.0 6.5 6.0 5.5 5.0 4.5 4.0 3.5 3.0 2.5 2.0 1.5 1.0 0.5 0.0 -0.5
f1 (ppm)

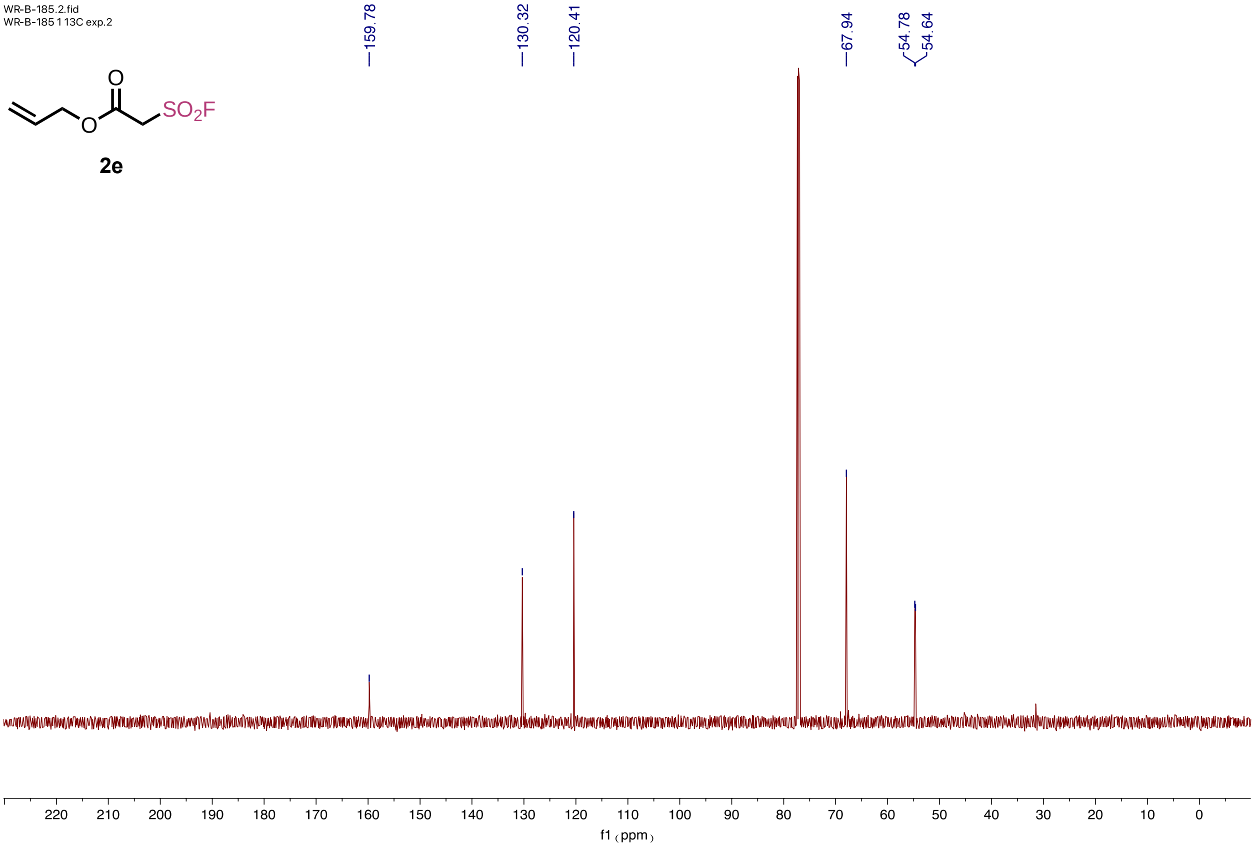
WR-B-185.2.fid
WR-B-185 1 13C exp.2
159.78
130.32
120.41
67.94
54.78
54.64
O
O
SO2F
2e
220 210 200 190 180 170 160 150 140 130 120 110 100 90 80 70 60 50 40 30 20 10 0
f1 (ppm)

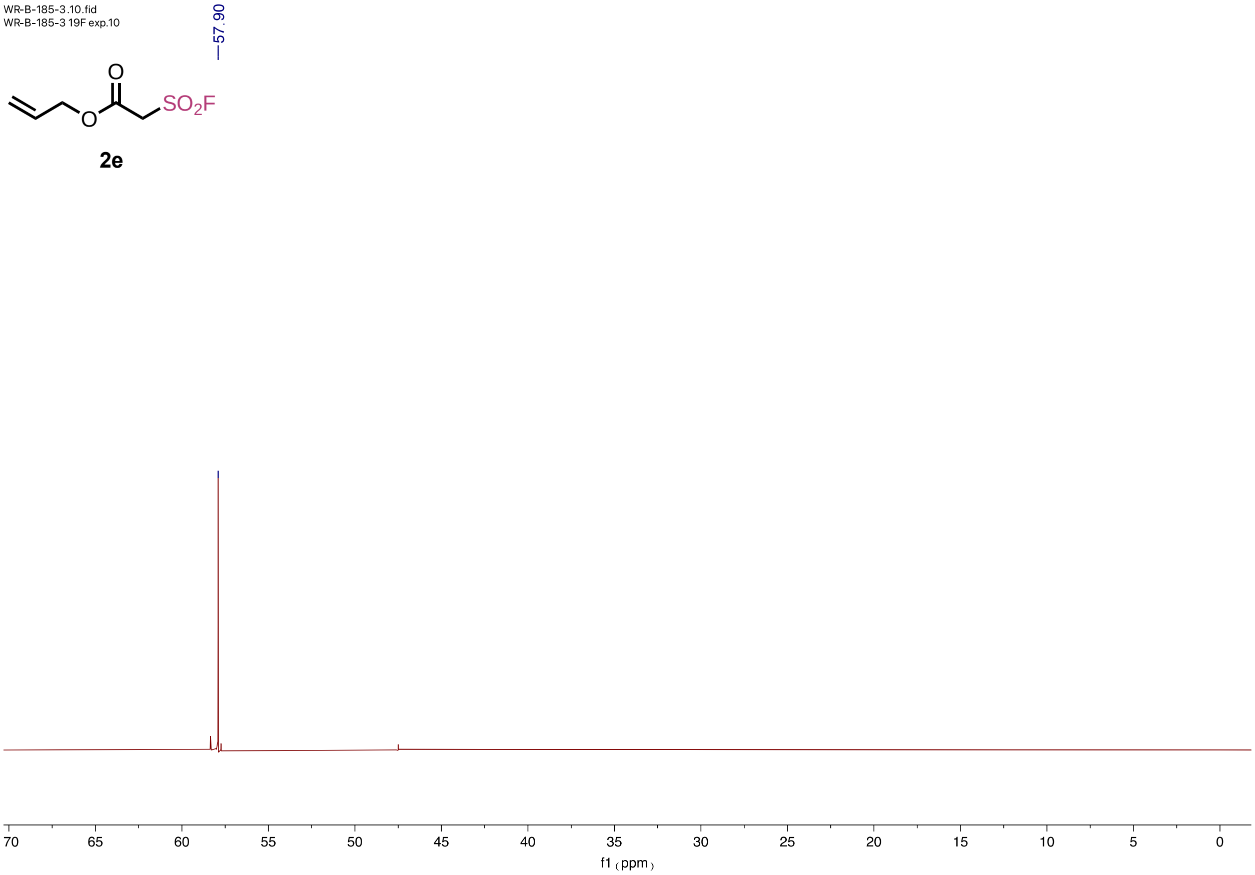
WR-B-185-3.10.fid
WR-B-185-3 19F exp.10
—57.90
2e
70 65 60 55 50 45 40 35 30 25 20 15 10 5 0
f1 (ppm)

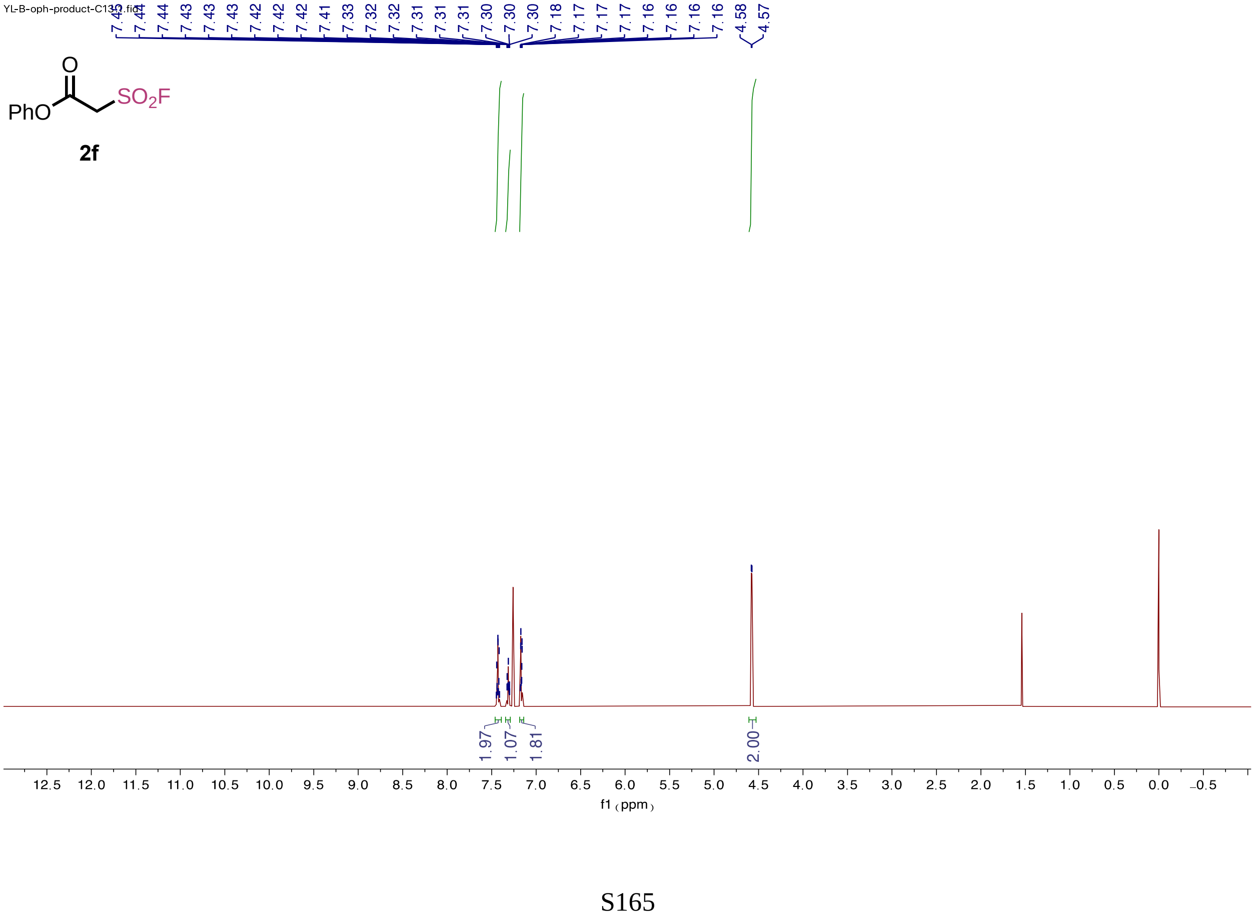
YL-B-oph-product-C13?.fid
7.45 7.44 7.44 7.43 7.43 7.43 7.42 7.42 7.42 7.41 7.33 7.32 7.32 7.31 7.31 7.31 7.30 7.30 7.30 7.18 7.17 7.17 7.17 7.16 7.16 7.16 4.58 4.57
PhO
2f
1.97 1.07 1.81 2.00
12.5 12.0 11.5 11.0 10.5 10.0 9.5 9.0 8.5 8.0 7.5 7.0 6.5 6.0 5.5 5.0 4.5 4.0 3.5 3.0 2.5 2.0 1.5 1.0 0.5 0.0 -0.5
f1 (ppm)

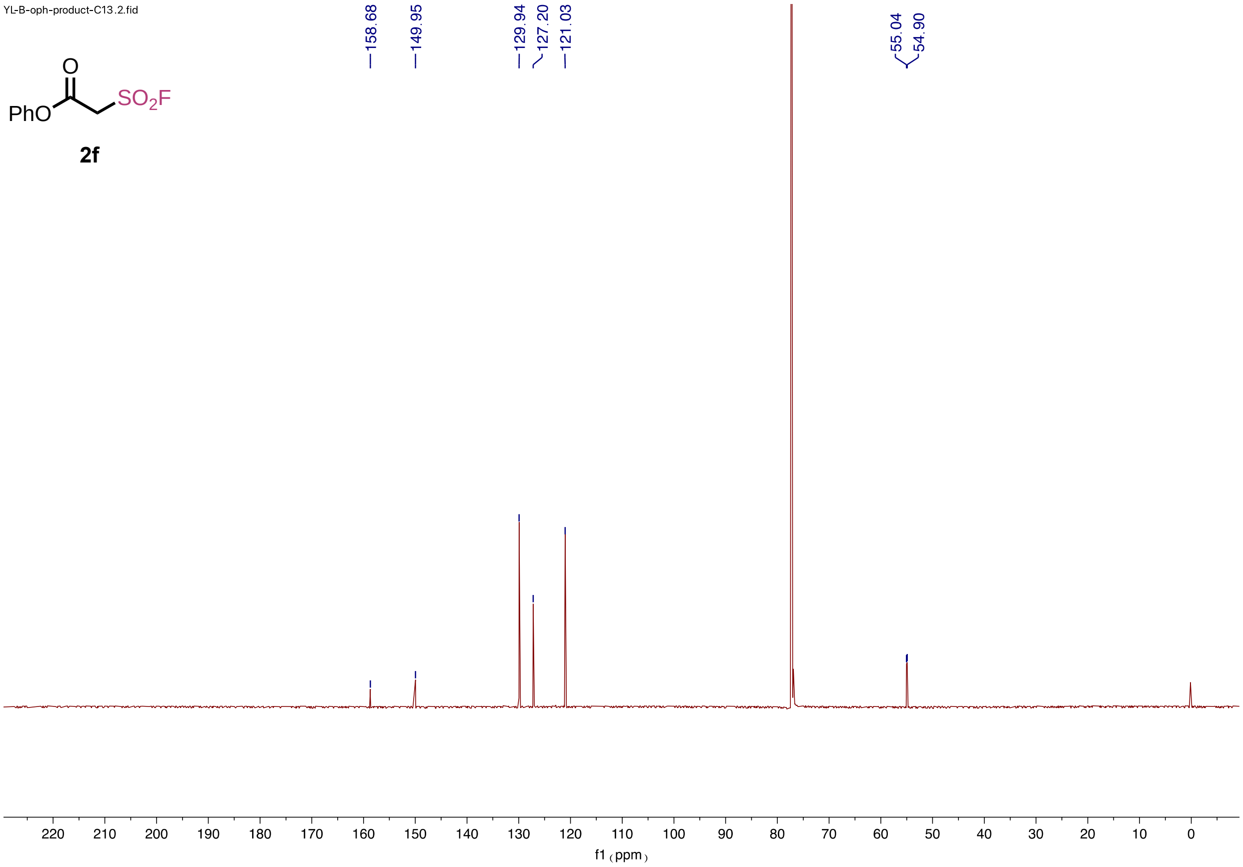
YL-B-oph-product-C13.2.fid
158.68
149.95
129.94
127.20
121.03
55.04
54.90
PhO
O
SO2F
2f
220
210
200
190
180
170
160
150
140
130
120
110
100
90
80
70
60
50
40
30
20
10
0
f1 (ppm)

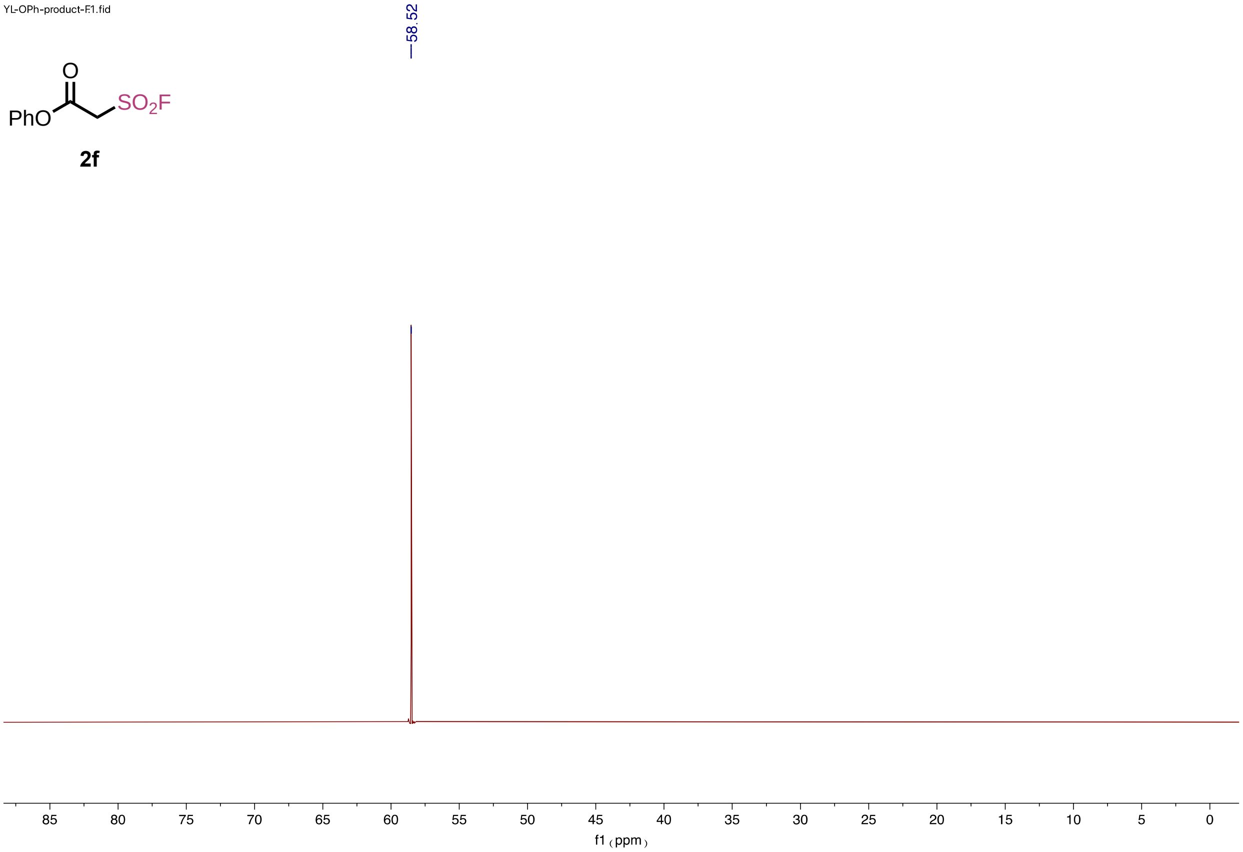
YL-OPh-product-F1.fid
58.52
PhO
O
SO2F
2f
85
80
75
70
65
60
55
50
45
40
35
30
25
20
15
10
5
0
f1 (ppm)

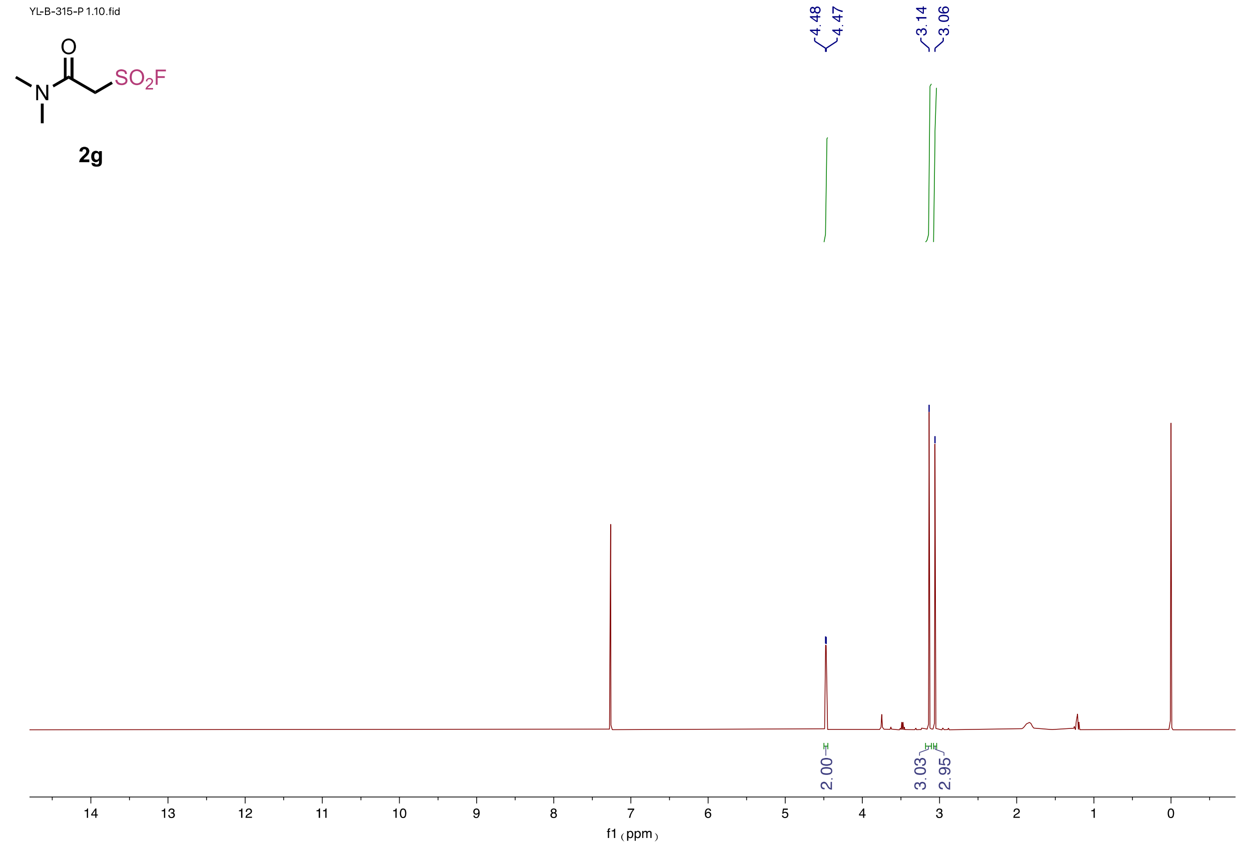
YL-B-315-P 1.10.fid
O
N
SO2F
2g
4.48
4.47
3.14
3.06
2.00
3.03
2.95
14 13 12 11 10 9 8 7 6 5 4 3 2 1 0
f1 (ppm)

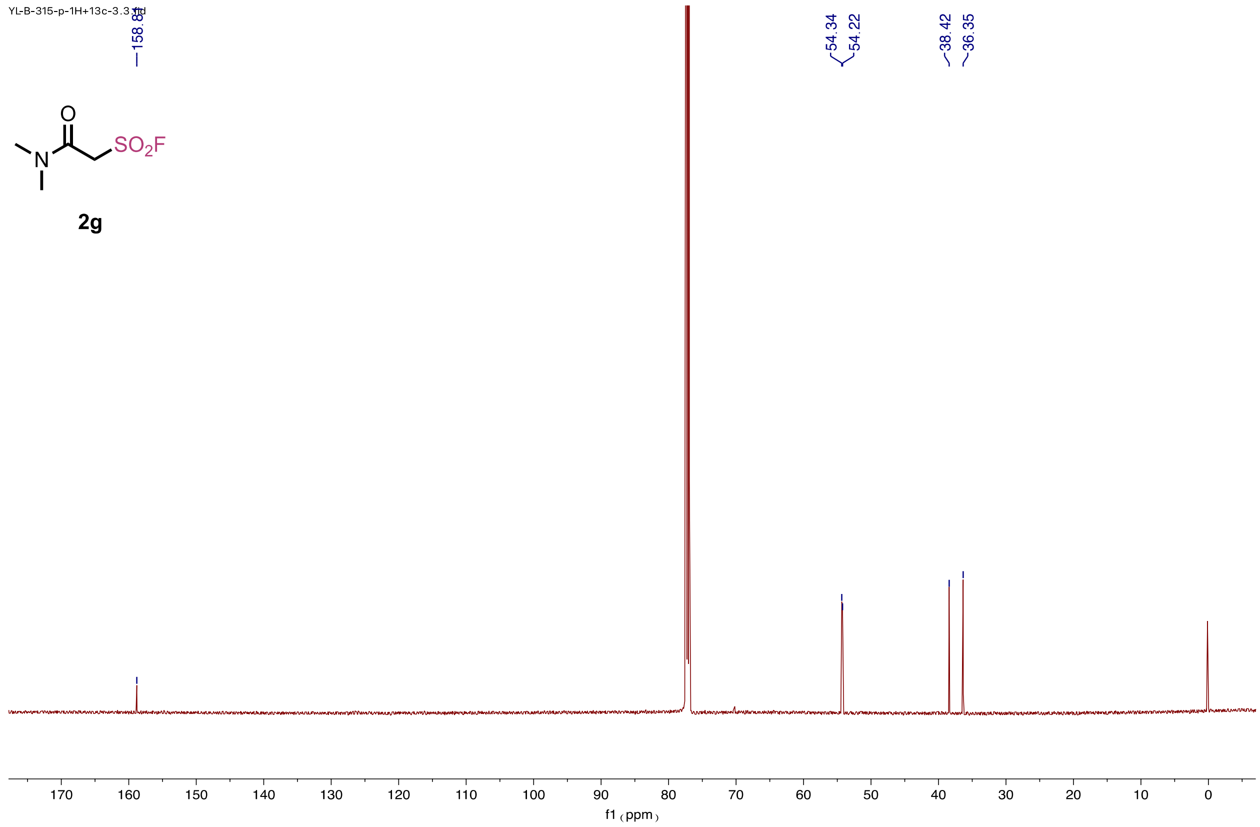
YL-B-315-p-1H+13c-3.3.fid
158.84
54.34
54.22
38.42
36.35
O
N
SO2F
2g
170 160 150 140 130 120 110 100 90 80 70 60 50 40 30 20 10 0
f1 (ppm)

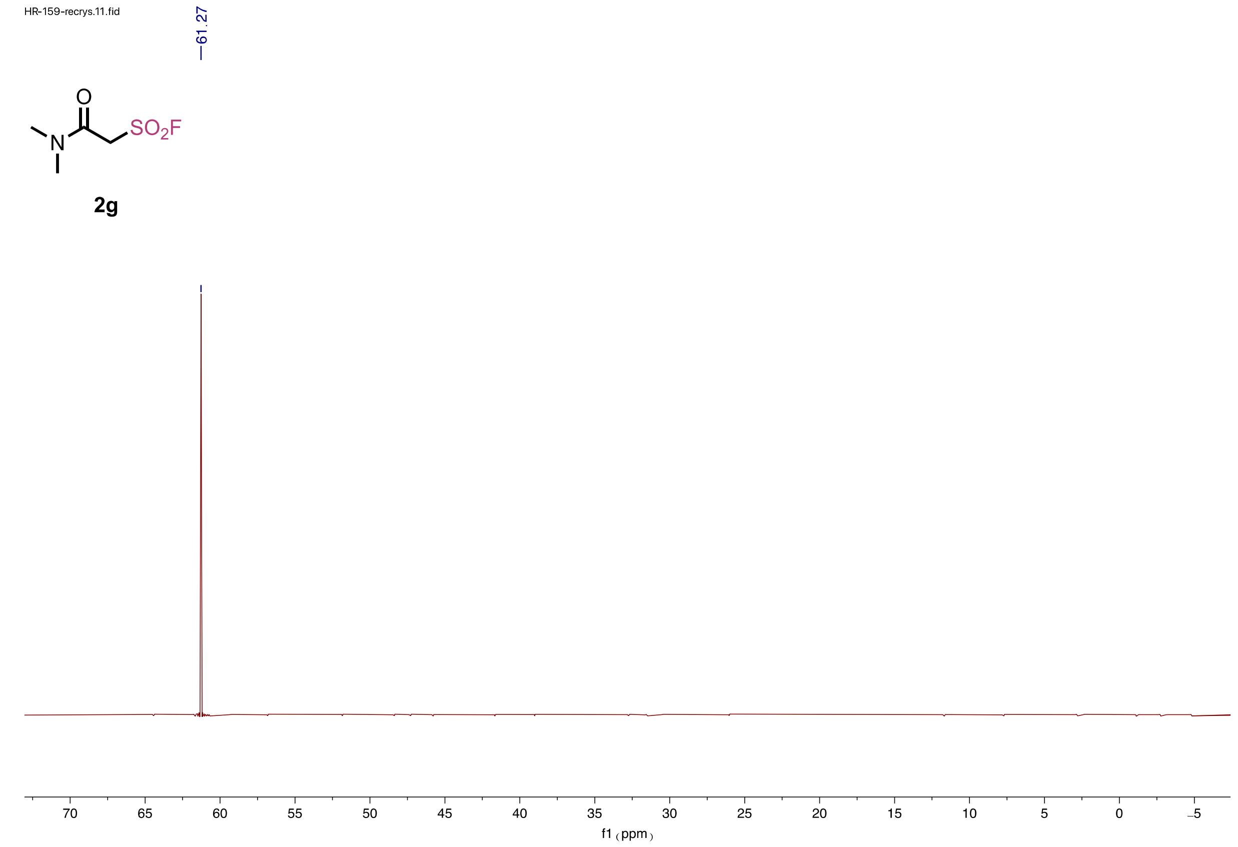
HR-159-recrys.11.fid
61.27
O
N
SO2F
2g
70 65 60 55 50 45 40 35 30 25 20 15 10 5 0 -5
f1 (ppm)

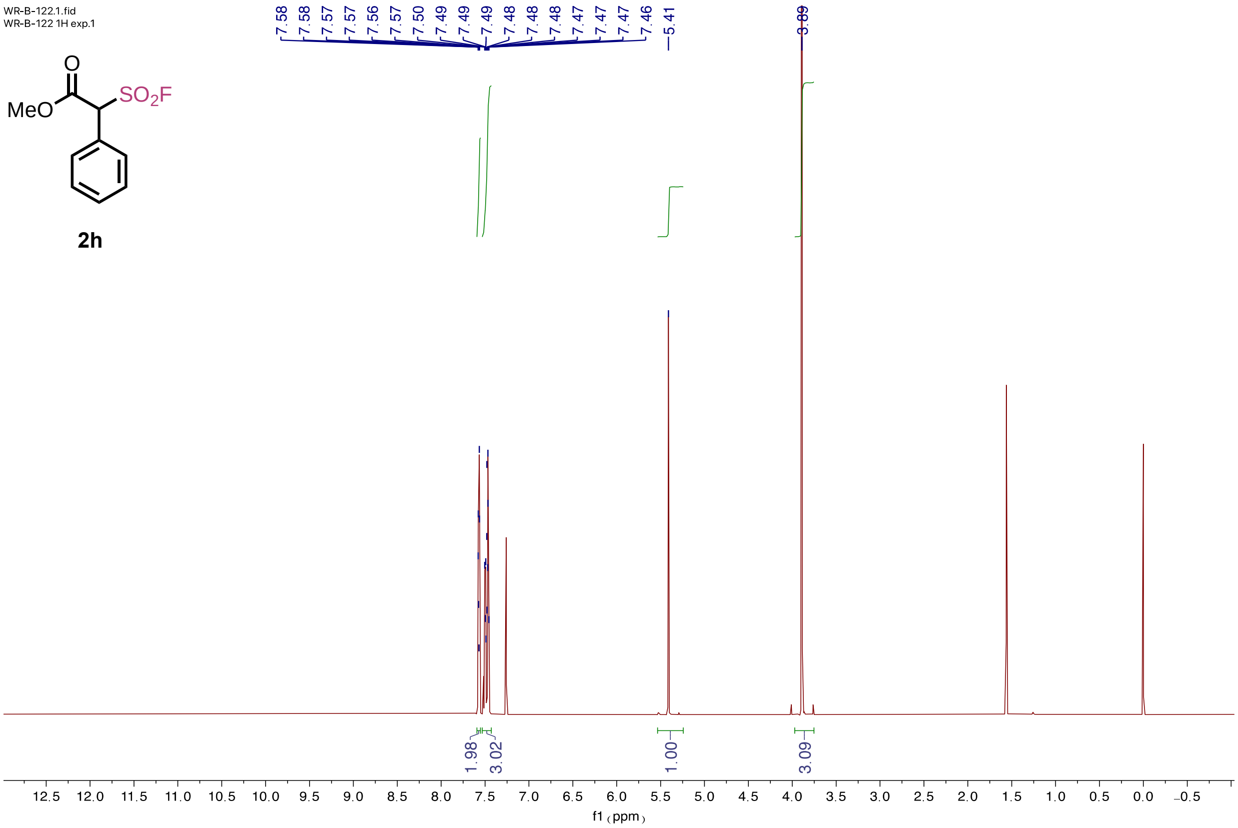
WR-B-122.1.fid
WR-B-122 1H exp.1
7.58 7.58 7.57 7.57 7.56 7.57 7.50 7.49 7.49 7.49 7.48 7.48 7.48 7.47 7.47 7.47 7.46
5.41
3.89
O
MeO
SO2F
2h
1.98
3.02
1.00
3.09
12.5 12.0 11.5 11.0 10.5 10.0 9.5 9.0 8.5 8.0 7.5 7.0 6.5 6.0 5.5 5.0 4.5 4.0 3.5 3.0 2.5 2.0 1.5 1.0 0.5 0.0 -0.5
f1 (ppm)

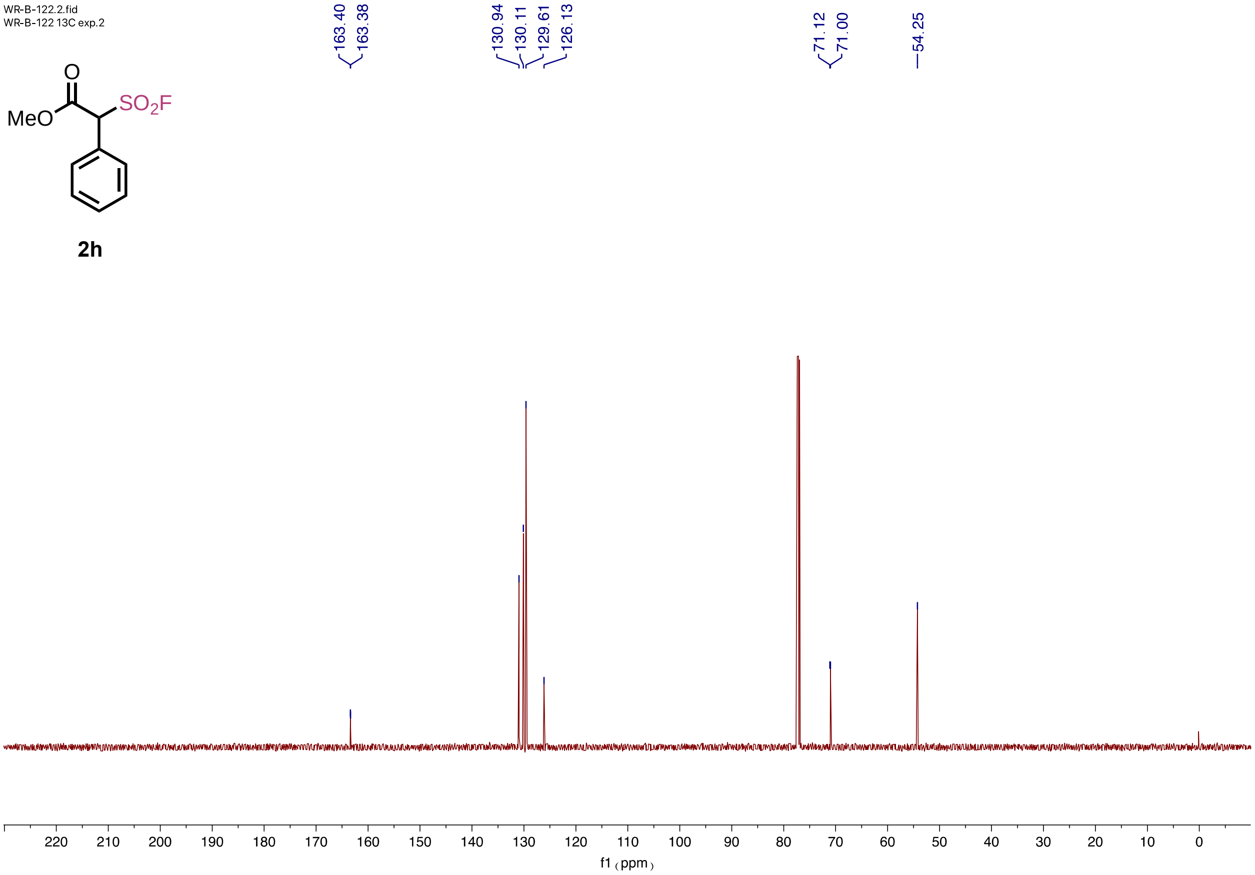
WR-B-122.2.fid
WR-B-122 13C exp.2
163.40
163.38
130.94
130.11
129.61
126.13
71.12
71.00
54.25
MeO
O
SO2F
2h
220 210 200 190 180 170 160 150 140 130 120 110 100 90 80 70 60 50 40 30 20 10 0
f1 (ppm)

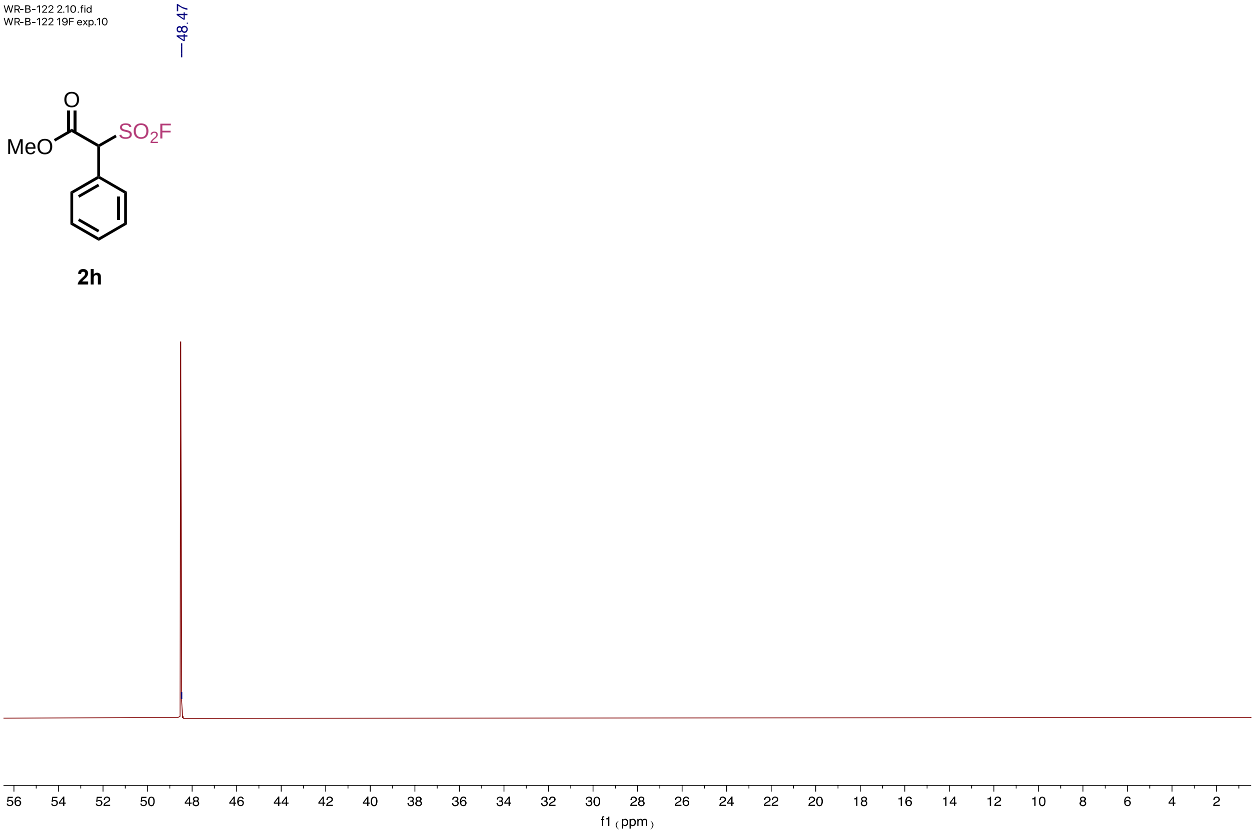
WR-B-122 2.10.fid
WR-B-122 19F exp.10
48.47
MeO
O
SO2F
2h
56 54 52 50 48 46 44 42 40 38 36 34 32 30 28 26 24 22 20 18 16 14 12 10 8 6 4 2
f1 (ppm)

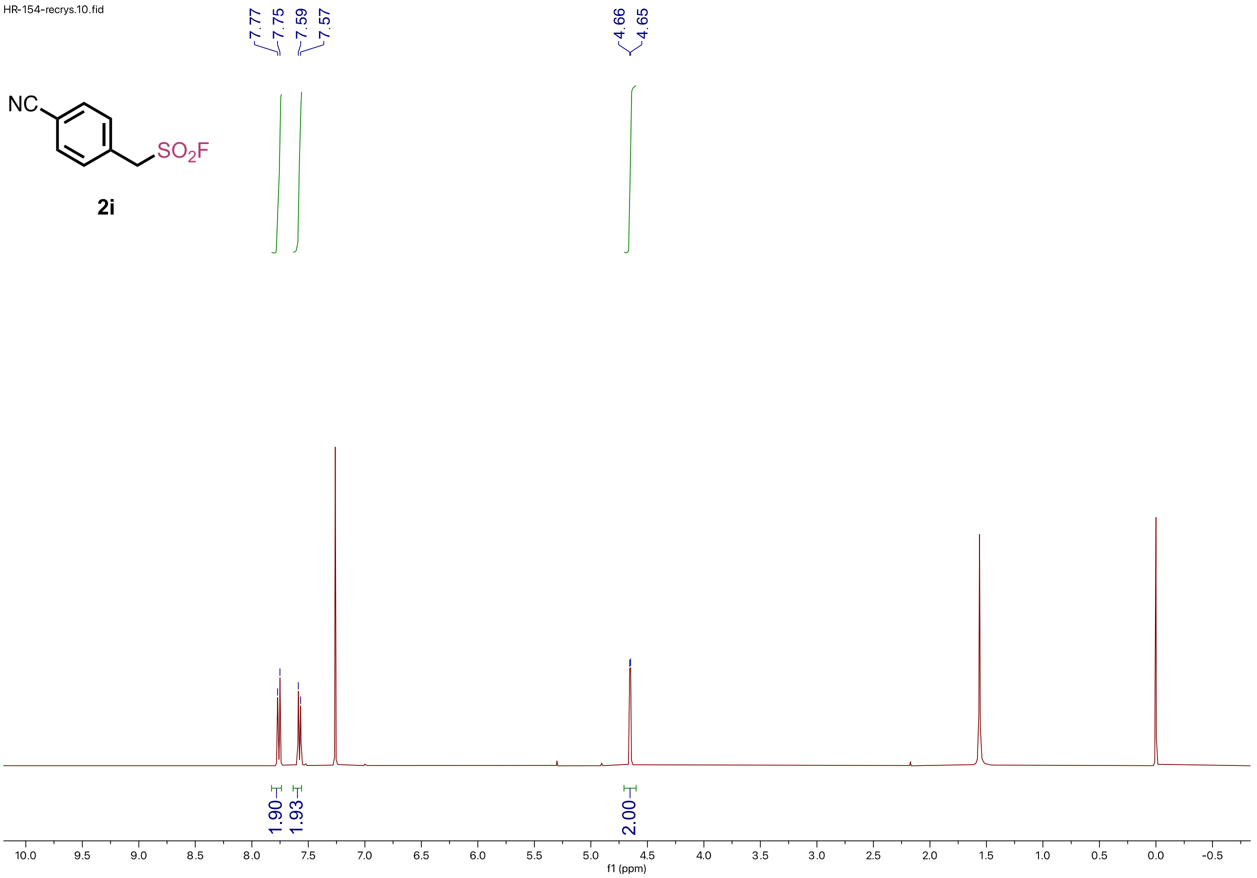
HR-154-recrys.10.fid
NC
SO2F
2i
7.77
7.75
7.59
7.57
4.66
4.65
1.90
1.93
2.00
10.0 9.5 9.0 8.5 8.0 7.5 7.0 6.5 6.0 5.5 5.0 4.5 4.0 3.5 3.0 2.5 2.0 1.5 1.0 0.5 0.0 -0.5
f1 (ppm)

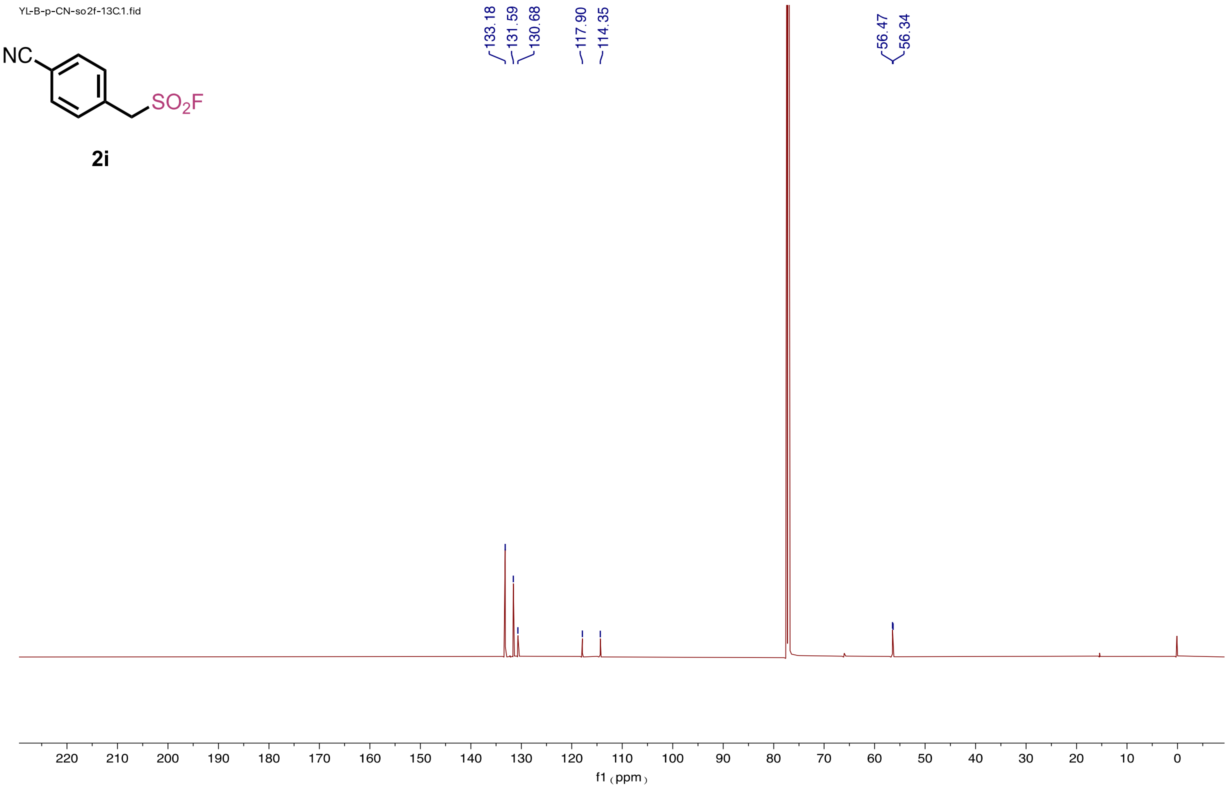
YL-B-p-CN-so2f-13C.1.fid
NC
SO2F
2i
133.18
131.59
130.68
117.90
114.35
56.47
56.34
220 210 200 190 180 170 160 150 140 130 120 110 100 90 80 70 60 50 40 30 20 10 0
f1 (ppm)

HR-154-recrys.11.fid

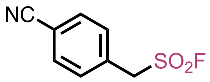

2i

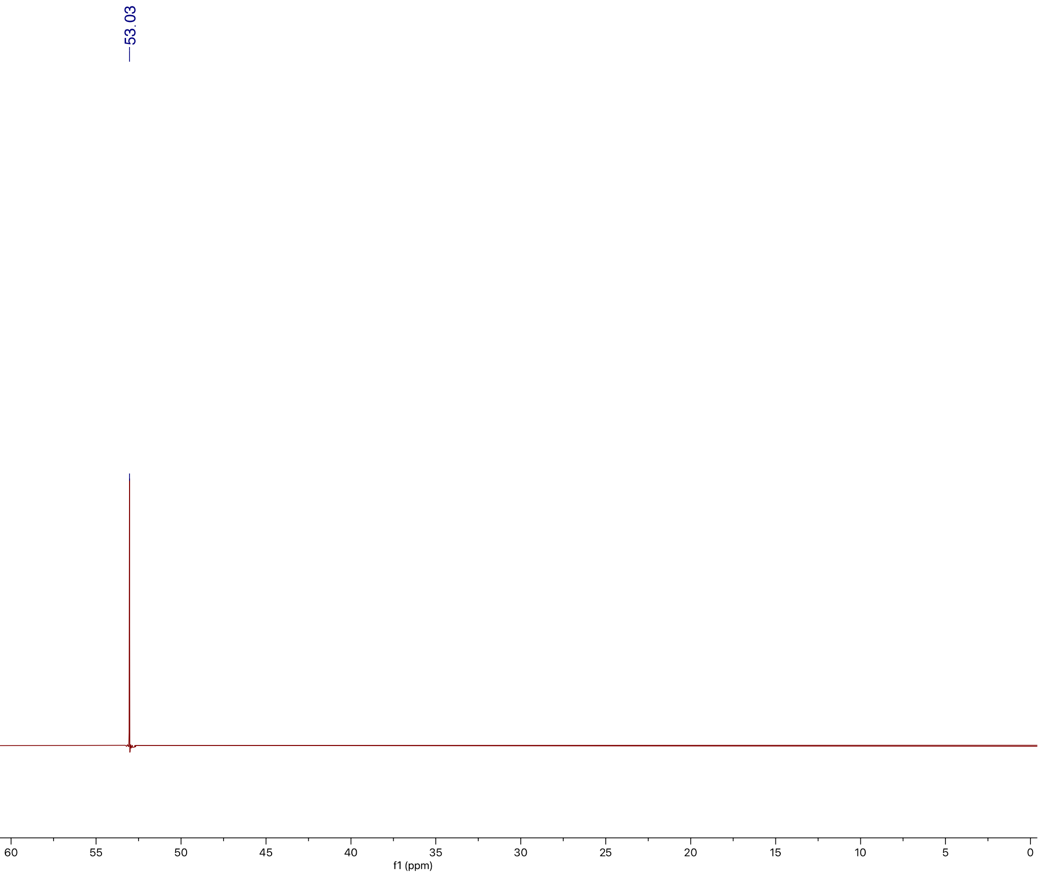

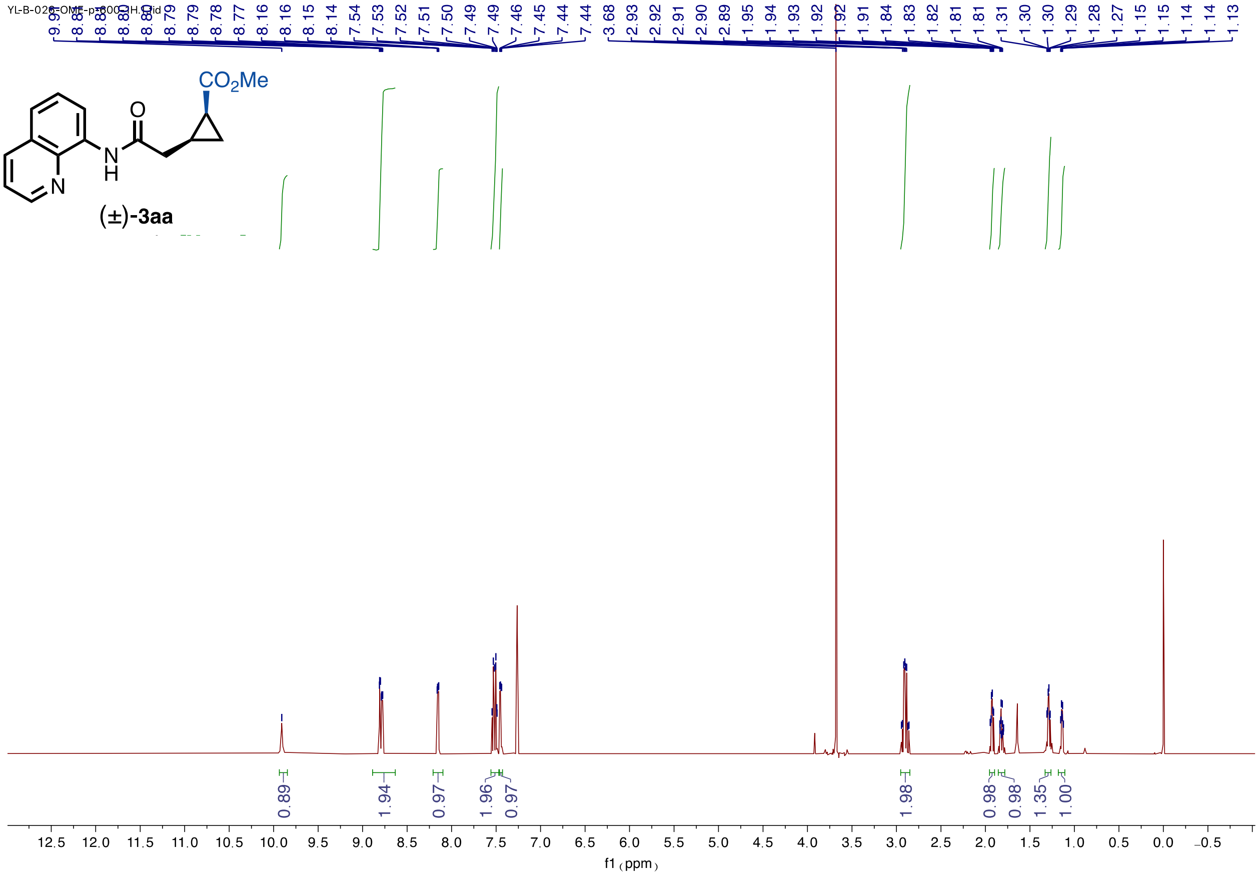
YL-B-026-OME-p-600-H.1.fid
CO2Me
O
N
H
N
(±)-3aa
0.89
1.94
0.97
1.96
0.97
1.98
0.98
0.98
1.35
1.00
f1 (ppm)

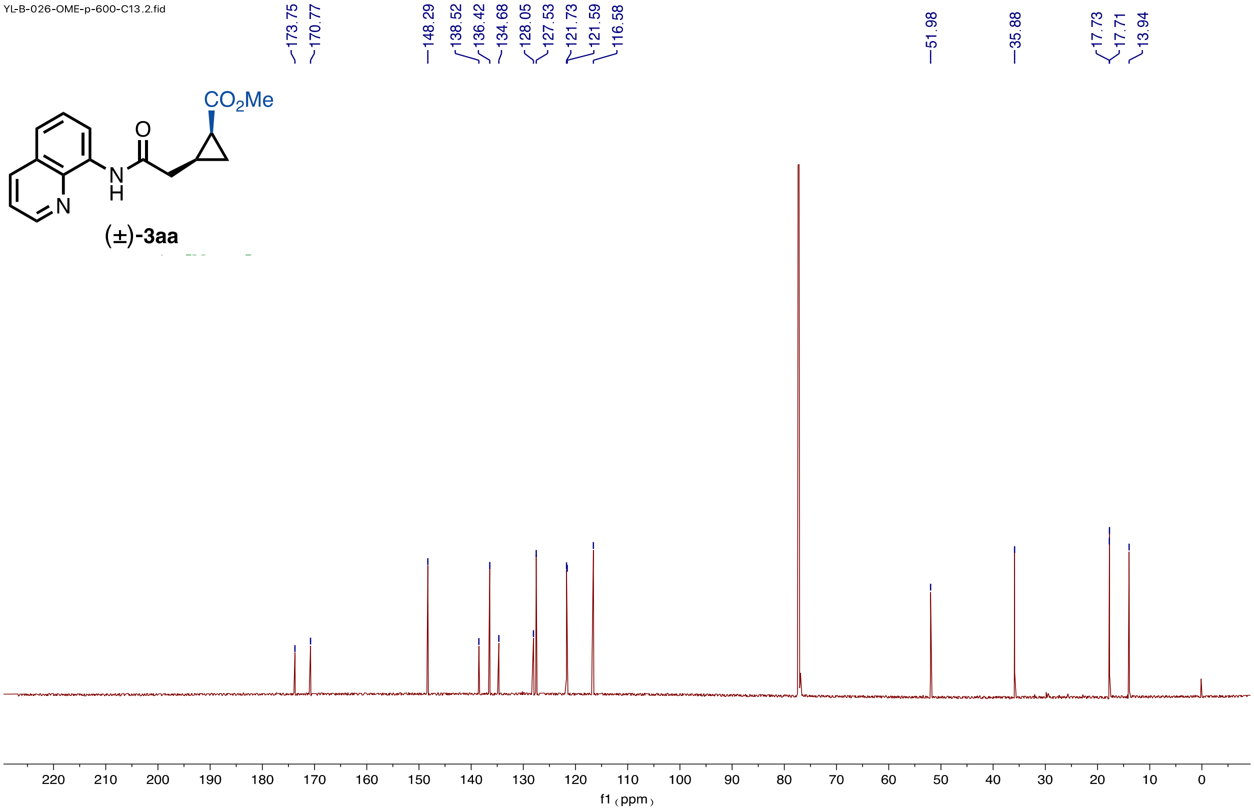
YL-B-026-OME-p-600-C13.2.fid
173.75
170.77
148.29
138.52
136.42
134.68
128.05
127.53
121.73
121.59
116.58
51.98
35.88
17.73
17.71
13.94
CO2Me
O
N
H
N
(±)-3aa
f1 (ppm)

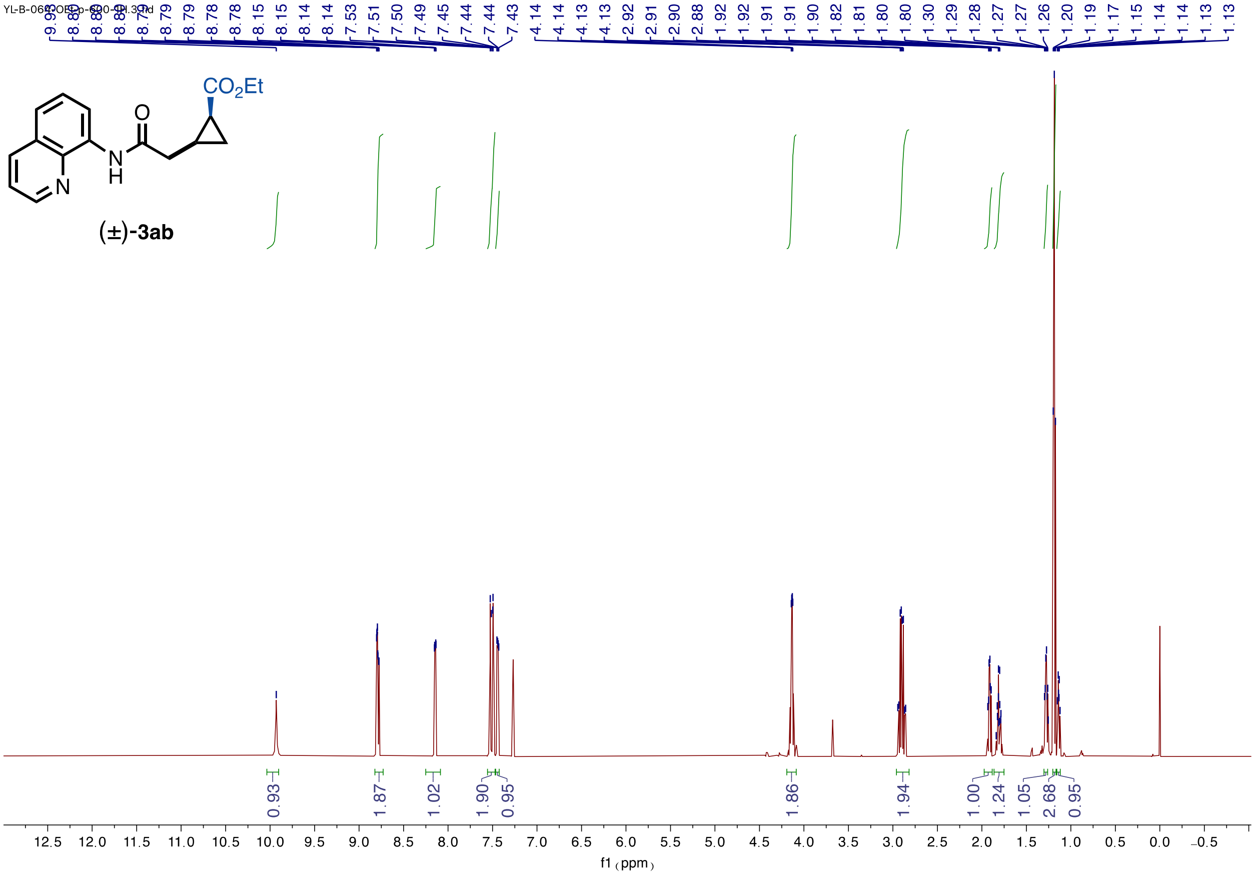

(±)-3ab
CO2Et
O
N
H
N
8.79 8.79 8.78 8.78 8.15 8.15 8.14 8.14 7.53 7.51 7.50 7.49 7.45 7.44 7.44 7.43 4.14 4.14 4.13 4.13 2.92 2.91 2.90 2.88 1.92 1.92 1.91 1.91 1.90 1.82 1.81 1.80 1.80 1.30 1.29 1.28 1.27 1.27 1.26 1.20 1.19 1.17 1.15 1.14 1.14 1.13 1.13
0.93 1.87 1.02 1.90 0.95 1.86 1.94 1.00 1.24 1.05 2.68 0.95
12.5 12.0 11.5 11.0 10.5 10.0 9.5 9.0 8.5 8.0 7.5 7.0 6.5 6.0 5.5 5.0 4.5 4.0 3.5 3.0 2.5 2.0 1.5 1.0 0.5 0.0 -0.5
f1 (ppm)

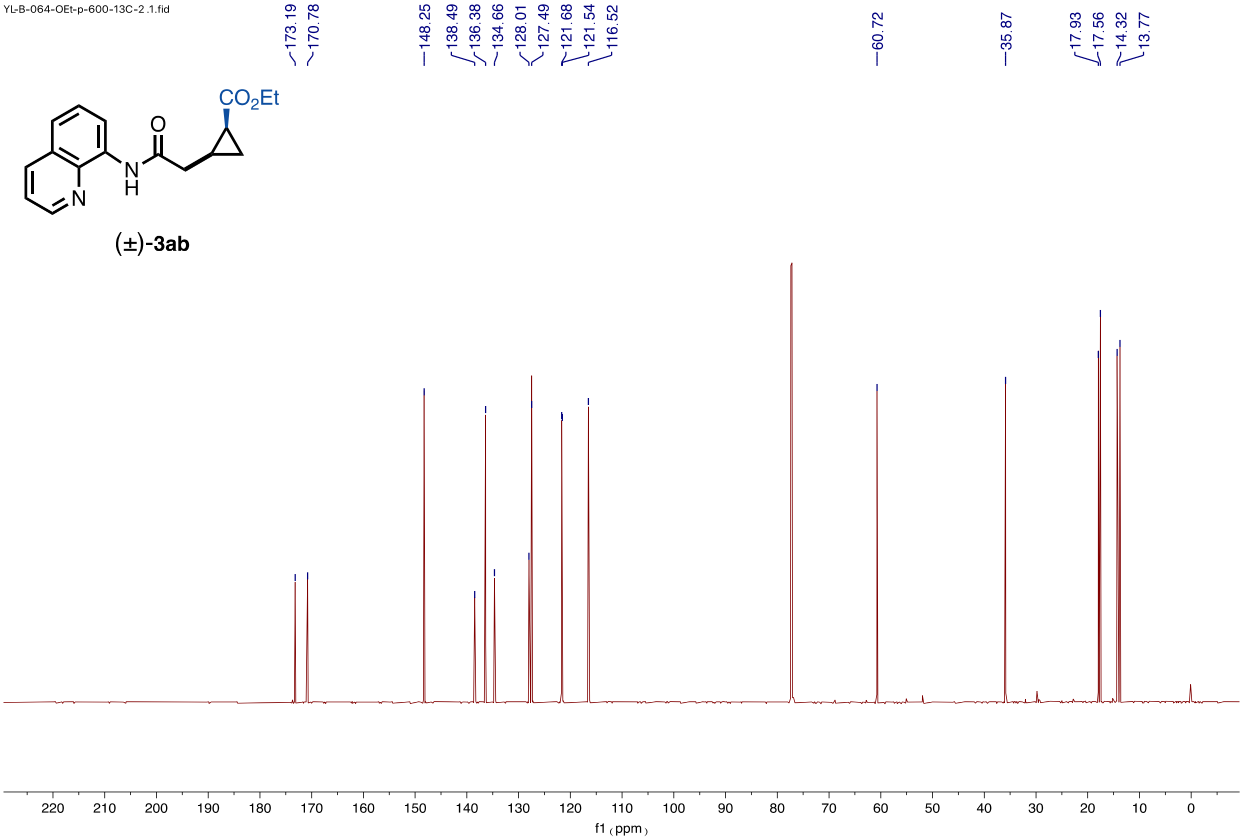

YL-B-064-OEt-p-600-13C-2.1.fid
173.19 170.78 148.25 138.49 136.38 134.66 128.01 127.49 121.68 121.54 116.52 60.72 35.87 17.93 17.56 14.32 13.77
CO2Et
O
N
H
N
(±)-3ab
220 210 200 190 180 170 160 150 140 130 120 110 100 90 80 70 60 50 40 30 20 10 0
f1 (ppm)

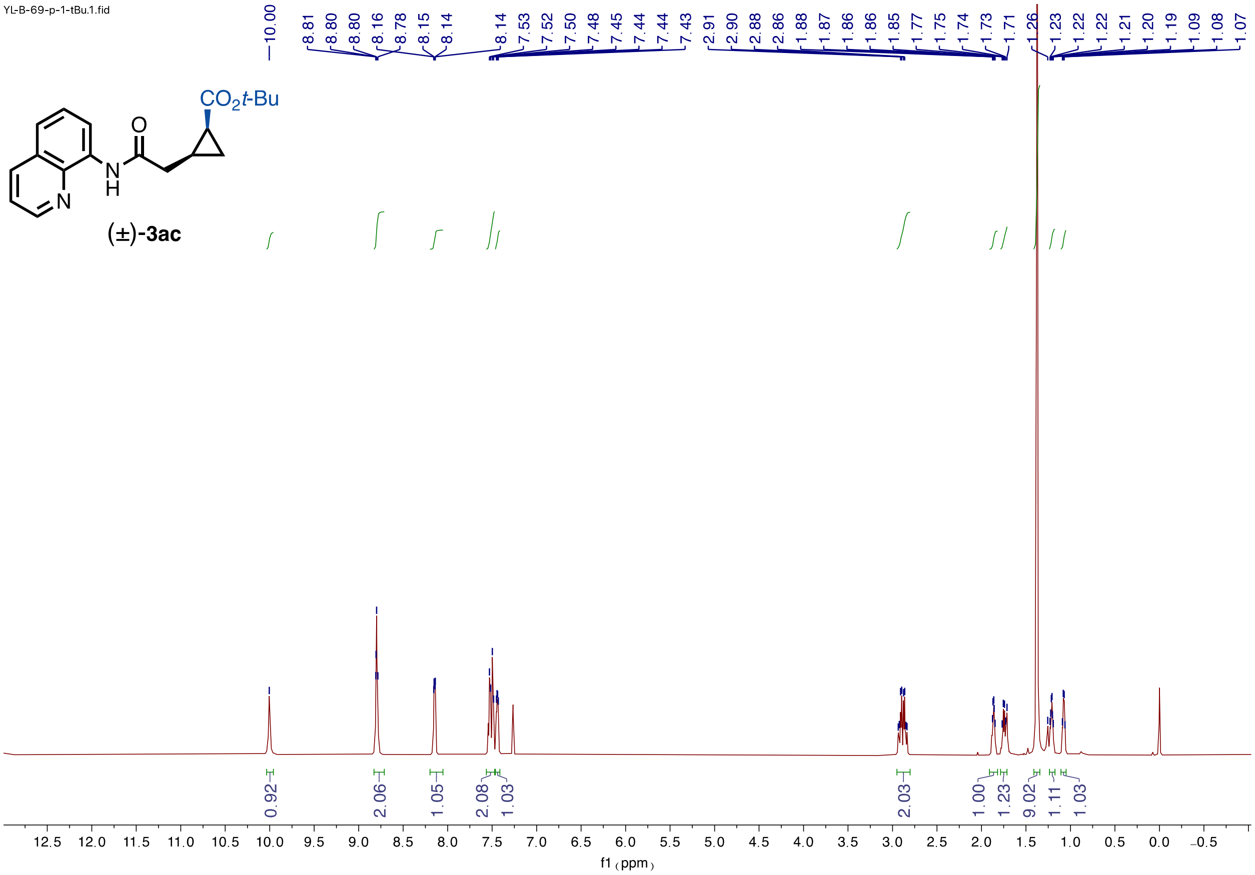

YL-B-69-p-1-tBu.1.fid
CO2t-Bu
O
N
H
N
(±)-3ac
f1 (ppm)

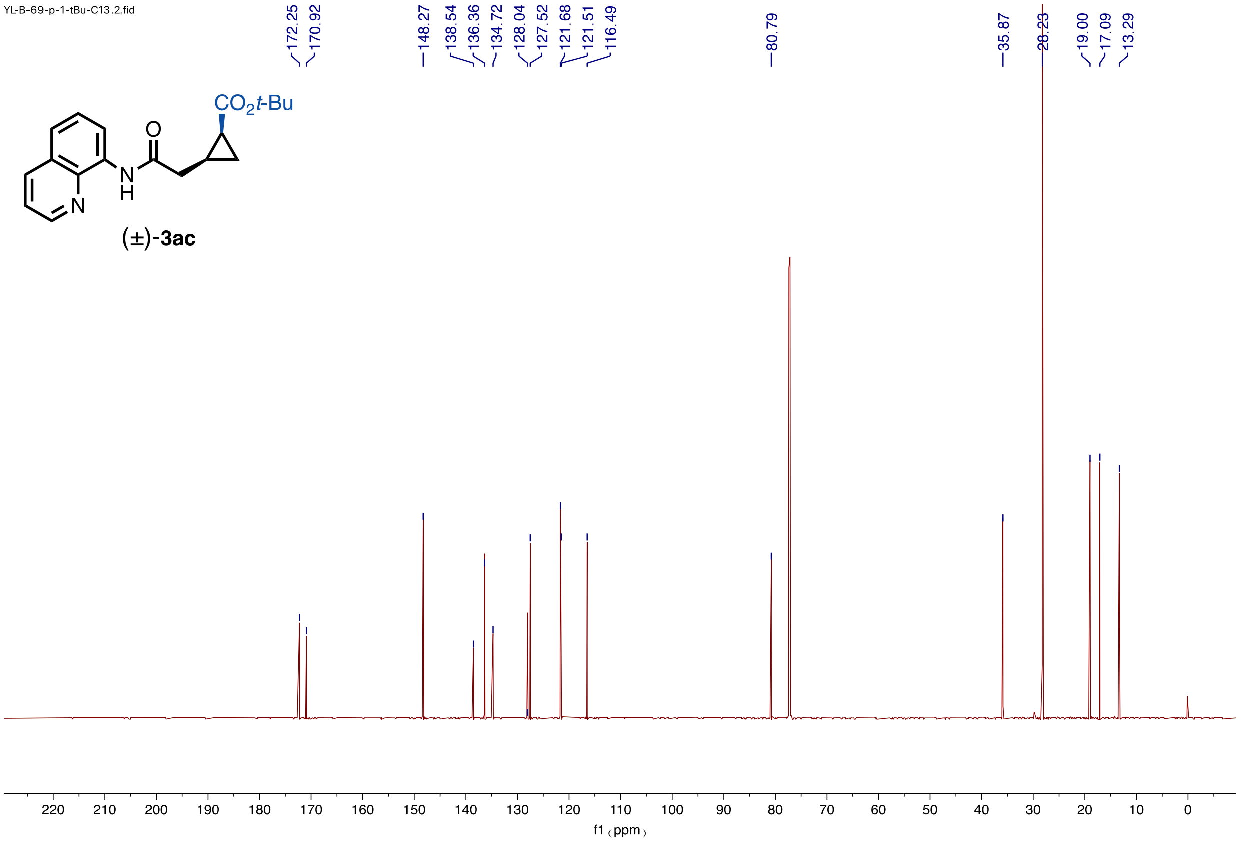

YL-B-69-p-1-tBu-C13.2.fid
CO2t-Bu
O
N
H
N
(±)-3ac
f1 (ppm)

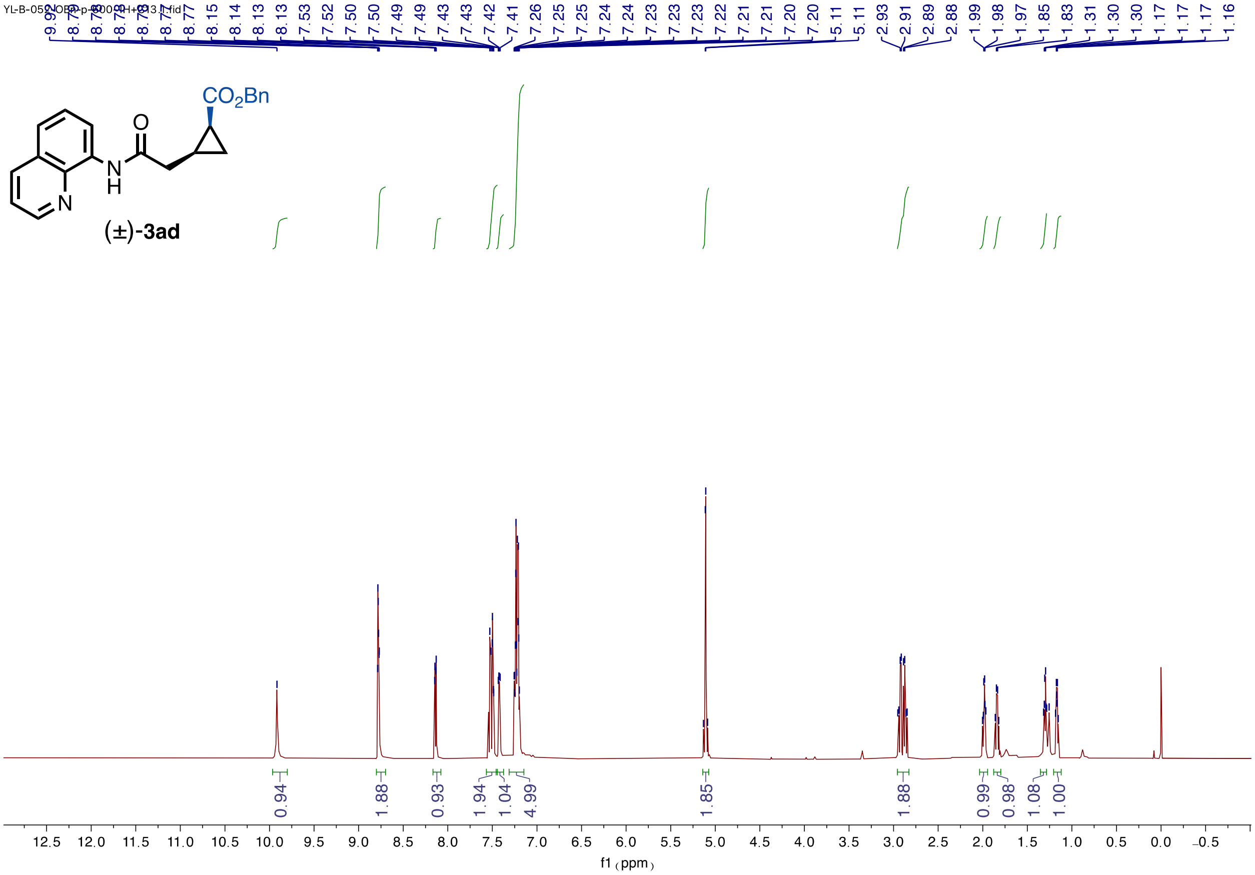
8.77 8.15 8.14 8.13 8.13 7.53 7.52 7.50 7.50 7.49 7.49 7.43 7.43 7.42 7.41 7.26 7.25 7.25 7.24 7.24 7.23 7.23 7.23 7.22 7.21 7.21 7.20 7.20 5.11 5.11 2.93 2.91 2.89 2.88 1.99 1.98 1.97 1.85 1.83 1.31 1.30 1.30 1.17 1.17 1.17 1.16
CO2Bn
O
N
H
N
(±)-3ad
0.94 1.88 0.93 1.94 1.04 4.99 1.85 1.88 0.99 0.98 1.08 1.00
f1 (ppm)

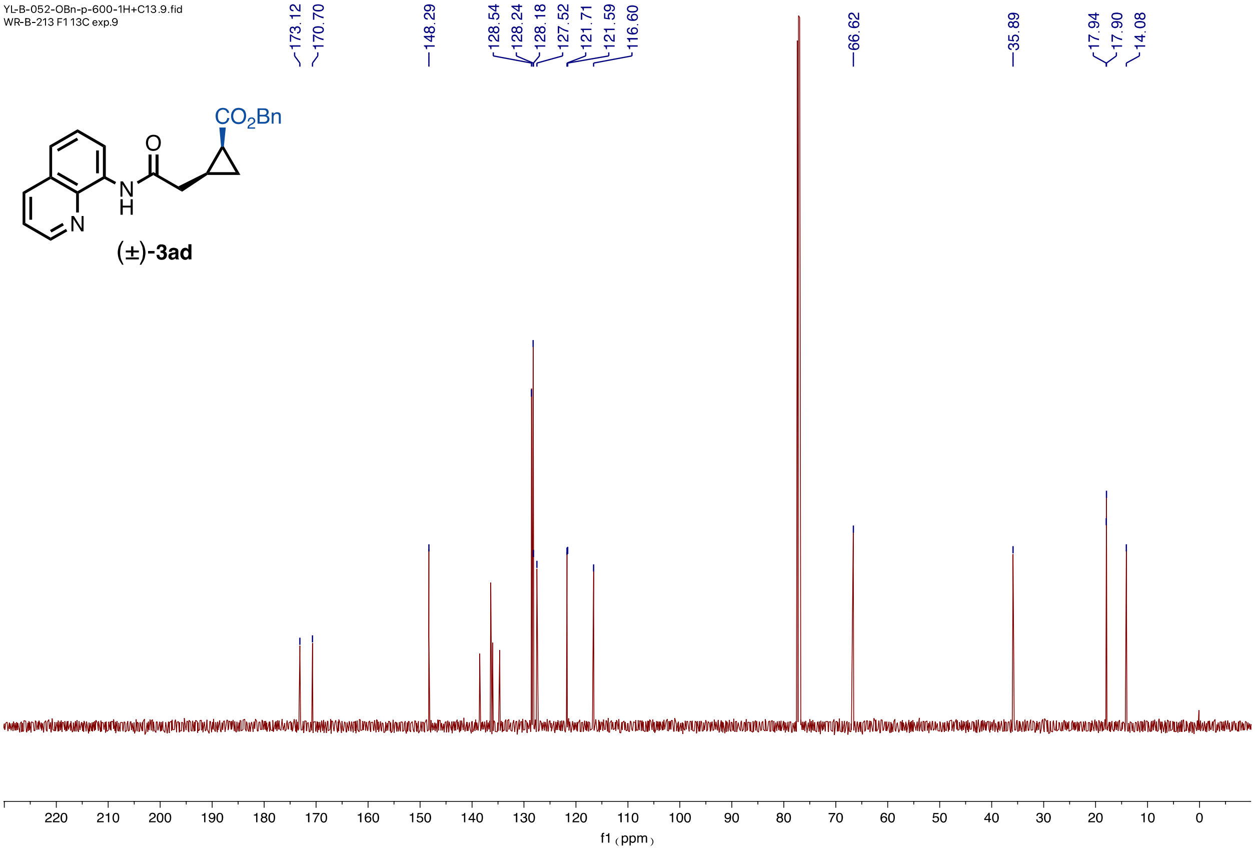
YL-B-052-OBn-p-600-1H+C13.9.fid
WR-B-213 F1 13C exp.9
173.12 170.70 148.29 128.54 128.24 128.18 127.52 121.71 121.59 116.60 66.62 35.89 17.94 17.90 14.08
CO2Bn
O
N
H
N
(±)-3ad
f1 (ppm)

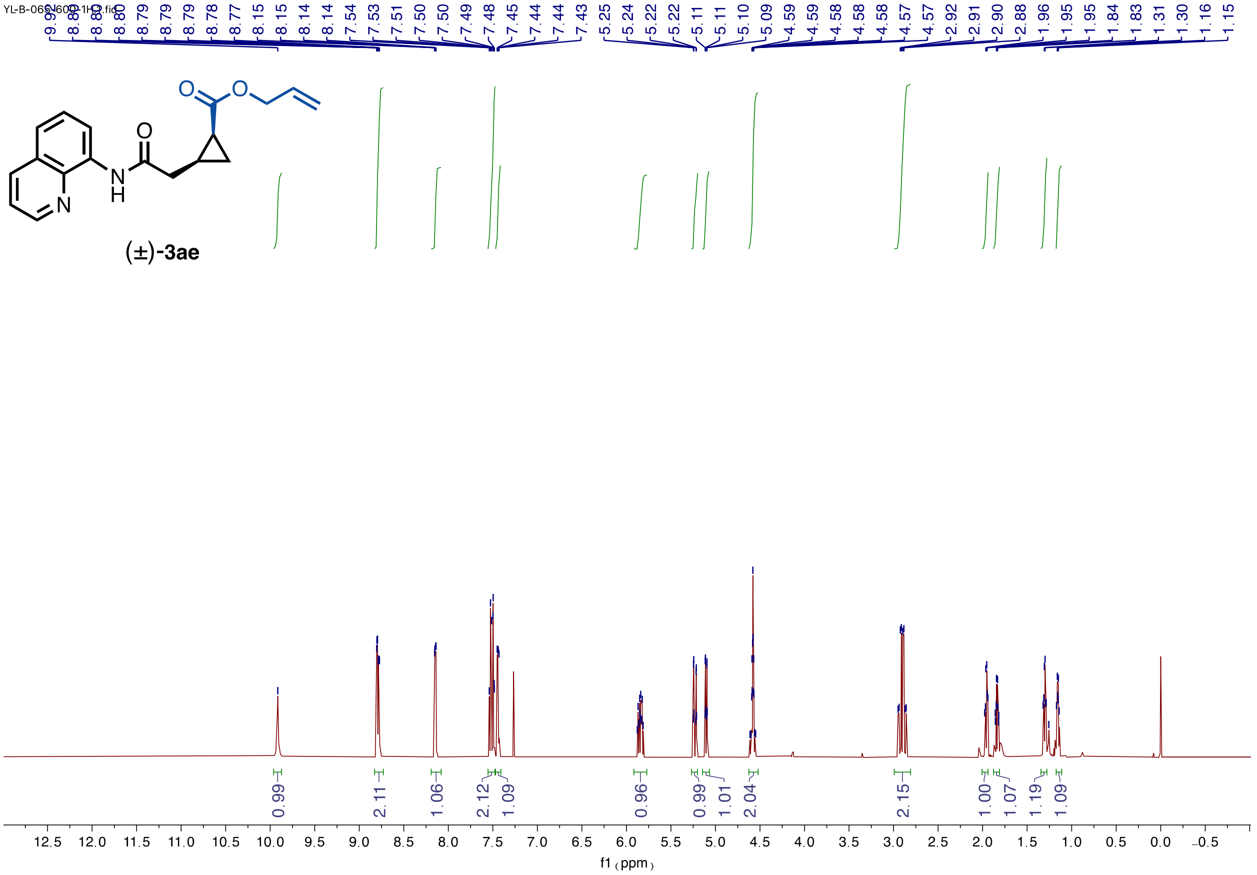
YL-B-065-600-1H-2.fid
(±)-3ae
f1 (ppm)

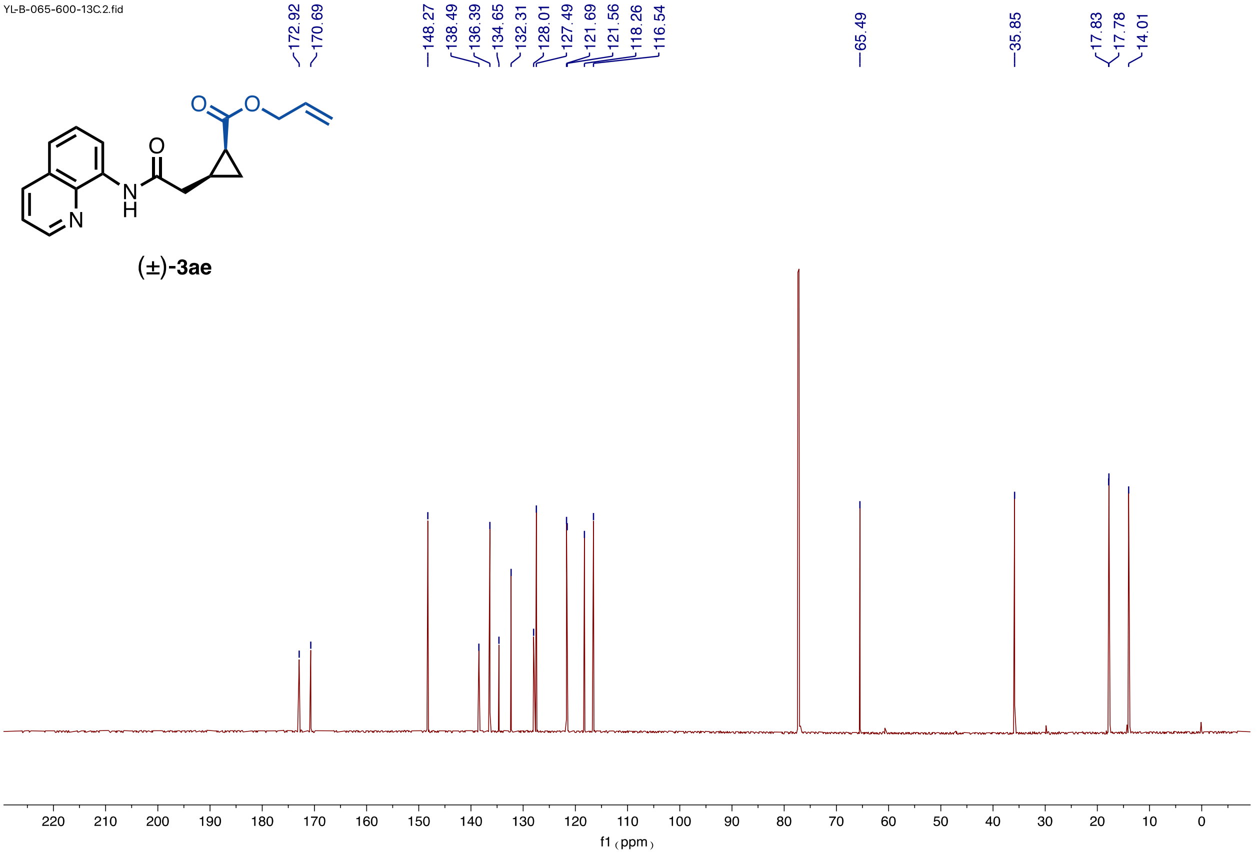
YL-B-065-600-13C.2.fid
172.92
170.69
148.27
138.49
136.39
134.65
132.31
128.01
127.49
121.69
121.56
118.26
116.54
65.49
35.85
17.83
17.78
14.01
(±)-3ae
f1 (ppm)

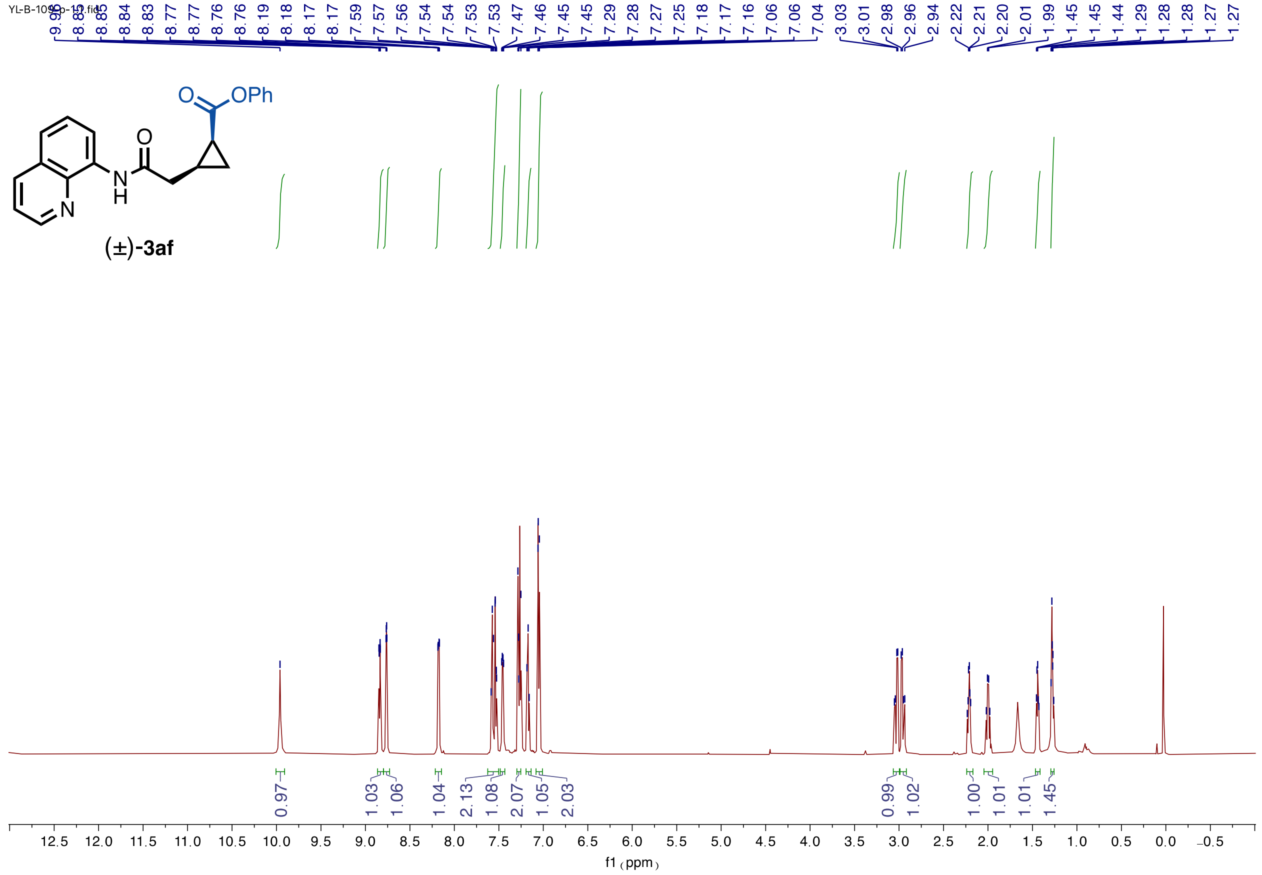
YL-B-109-p-1-1H.fid
9.96 8.85 8.85 8.84 8.83 8.77 8.77 8.76 8.76 8.19 8.18 8.17 8.17 7.59 7.57 7.56 7.54 7.54 7.53 7.53 7.47 7.46 7.45 7.45 7.29 7.28 7.27 7.25 7.18 7.17 7.16 7.06 7.06 7.04 3.03 3.01 2.98 2.96 2.94 2.22 2.21 2.20 2.01 1.99 1.45 1.45 1.44 1.29 1.28 1.27 1.27
O
OPh
O
N
H
N
(±)-3af
0.97 1.03 1.06 1.04 2.13 1.08 2.07 1.05 2.03 0.99 1.02 1.00 1.01 1.01 1.45
f1 (ppm)

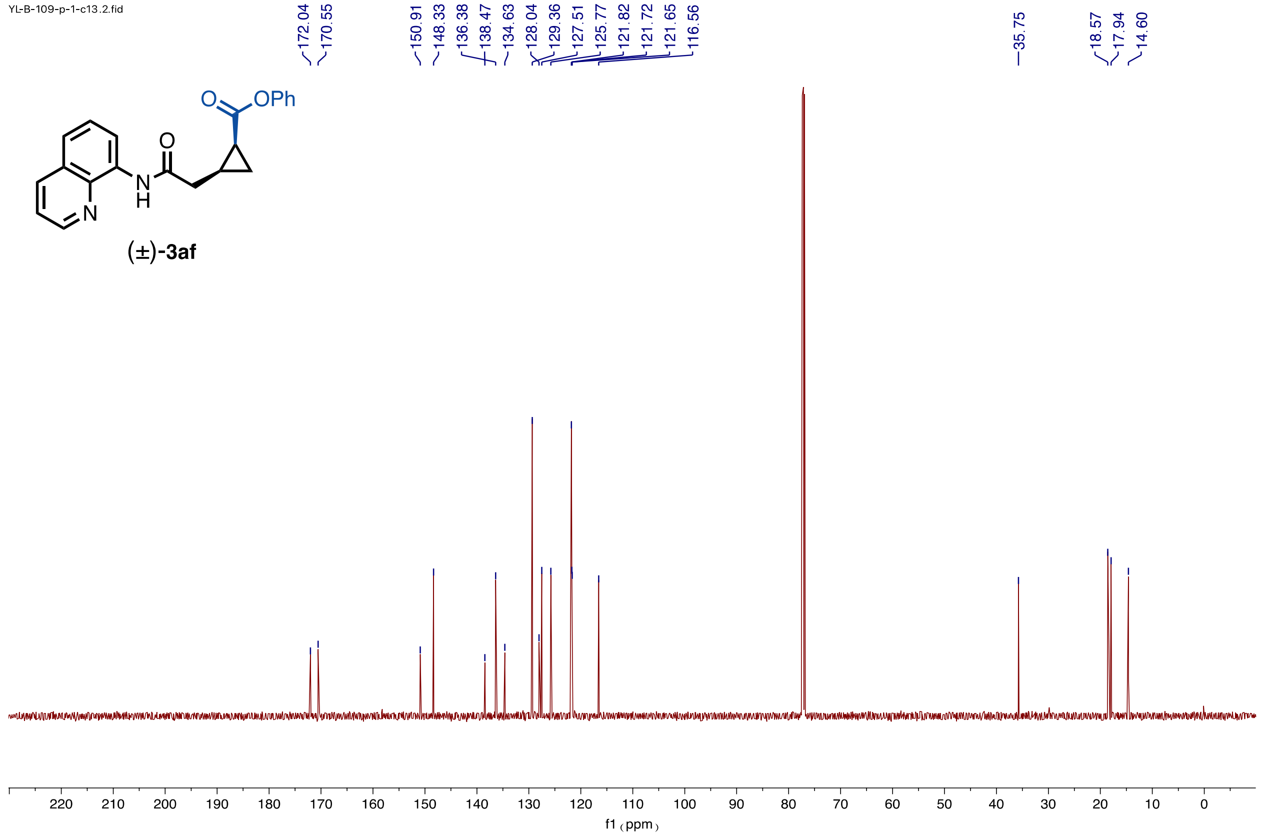
YL-B-109-p-1-c13.2.fid
172.04 170.55 150.91 148.33 136.38 138.47 134.63 128.04 129.36 127.51 125.77 121.82 121.72 121.65 116.56 35.75 18.57 17.94 14.60
O
OPh
O
N
H
N
(±)-3af
f1 (ppm)

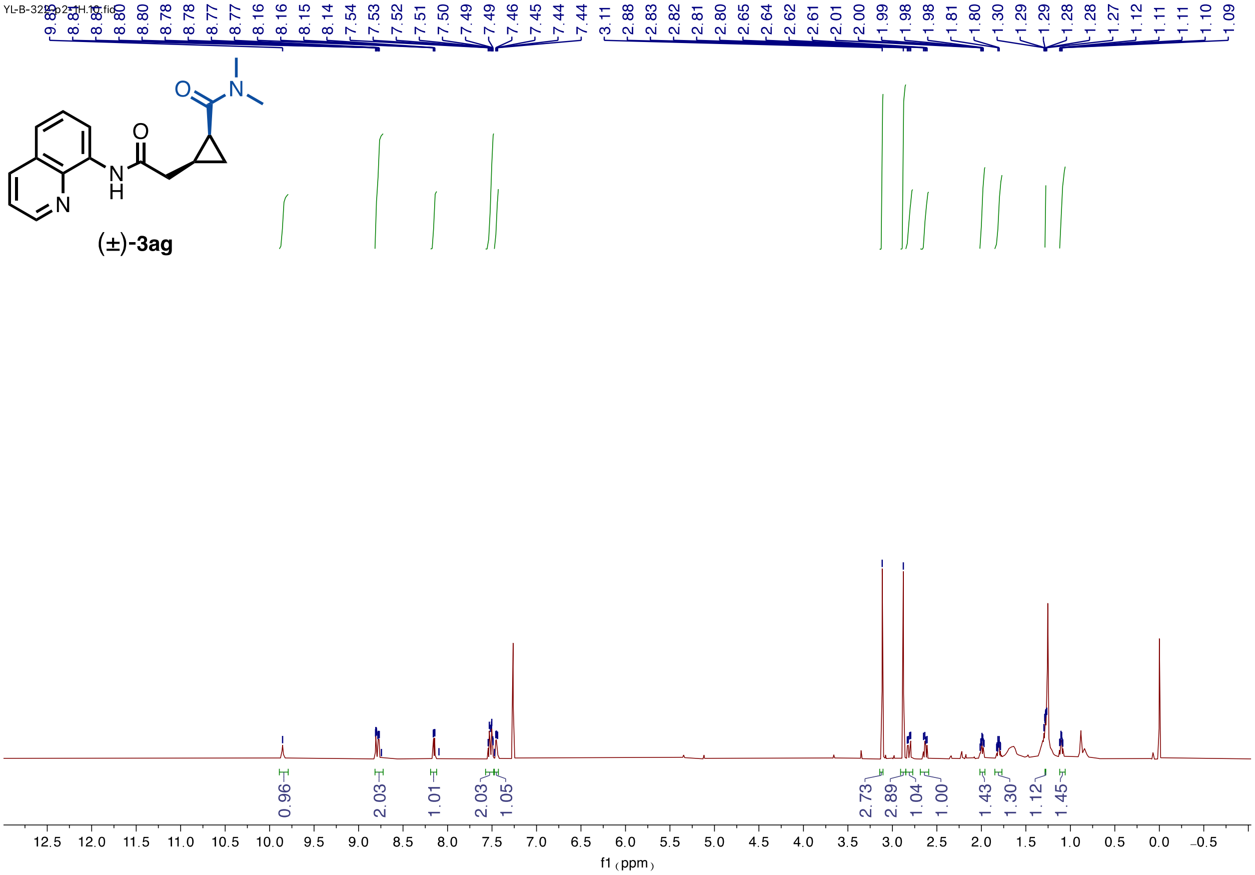
YL-B-322-p2-1H.10.fid
9.83 8.81 8.80 8.80 8.80 8.78 8.78 8.77 8.77 8.16 8.16 8.15 8.14 7.54 7.53 7.52 7.51 7.50 7.49 7.49 7.46 7.45 7.44 7.44 3.11 2.88 2.83 2.82 2.81 2.80 2.65 2.64 2.62 2.61 2.01 2.00 1.99 1.98 1.98 1.81 1.80 1.30 1.29 1.29 1.28 1.28 1.27 1.12 1.11 1.11 1.10 1.09
O
N
O
N
H
N
(±)-3ag
0.96 2.03 1.01 2.03 1.05 2.73 2.89 1.04 1.00 1.43 1.30 1.12 1.45
12.5 12.0 11.5 11.0 10.5 10.0 9.5 9.0 8.5 8.0 7.5 7.0 6.5 6.0 5.5 5.0 4.5 4.0 3.5 3.0 2.5 2.0 1.5 1.0 0.5 0.0 -0.5
f1 (ppm)

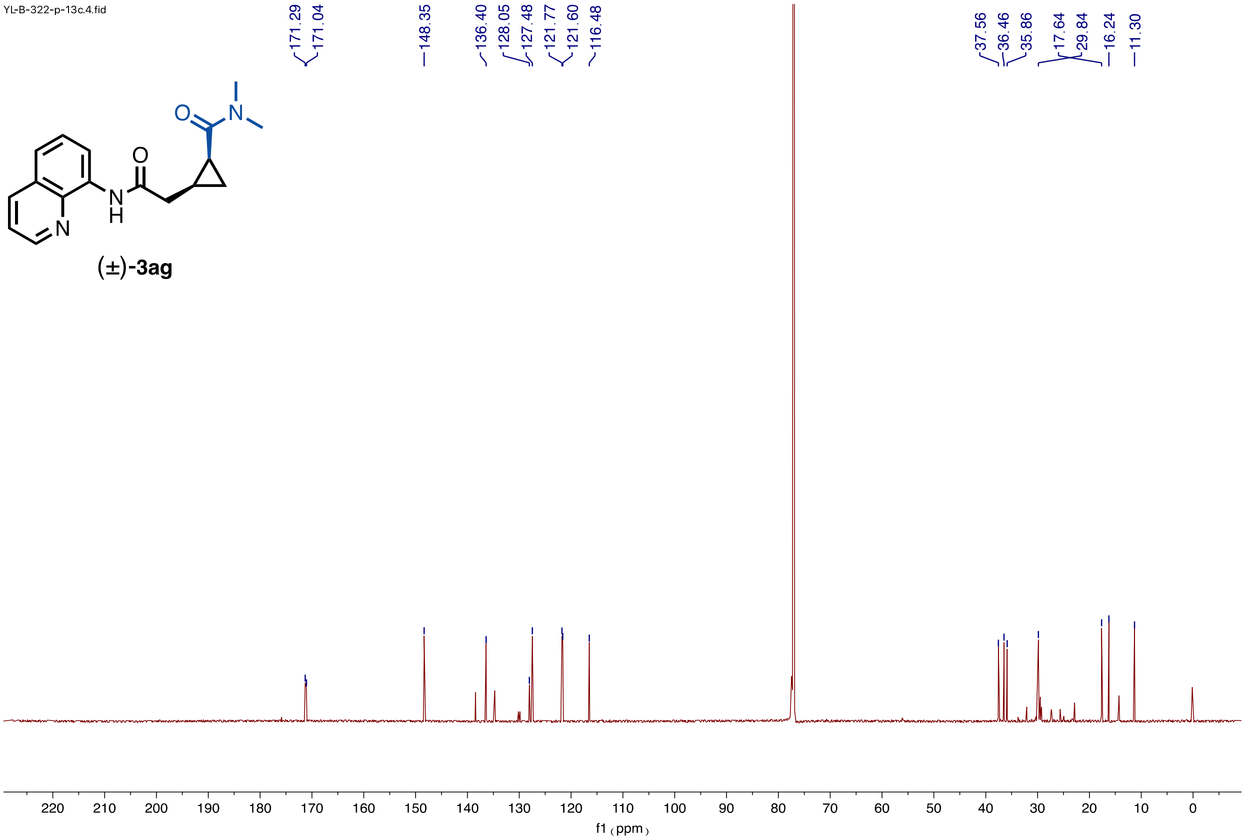
YL-B-322-p-13c.4.fid
171.29 171.04 148.35 136.40 128.05 127.48 121.77 121.60 116.48 37.56 36.46 35.86 17.64 29.84 16.24 11.30
O
N
O
N
H
N
(±)-3ag
220 210 200 190 180 170 160 150 140 130 120 110 100 90 80 70 60 50 40 30 20 10 0
f1 (ppm)

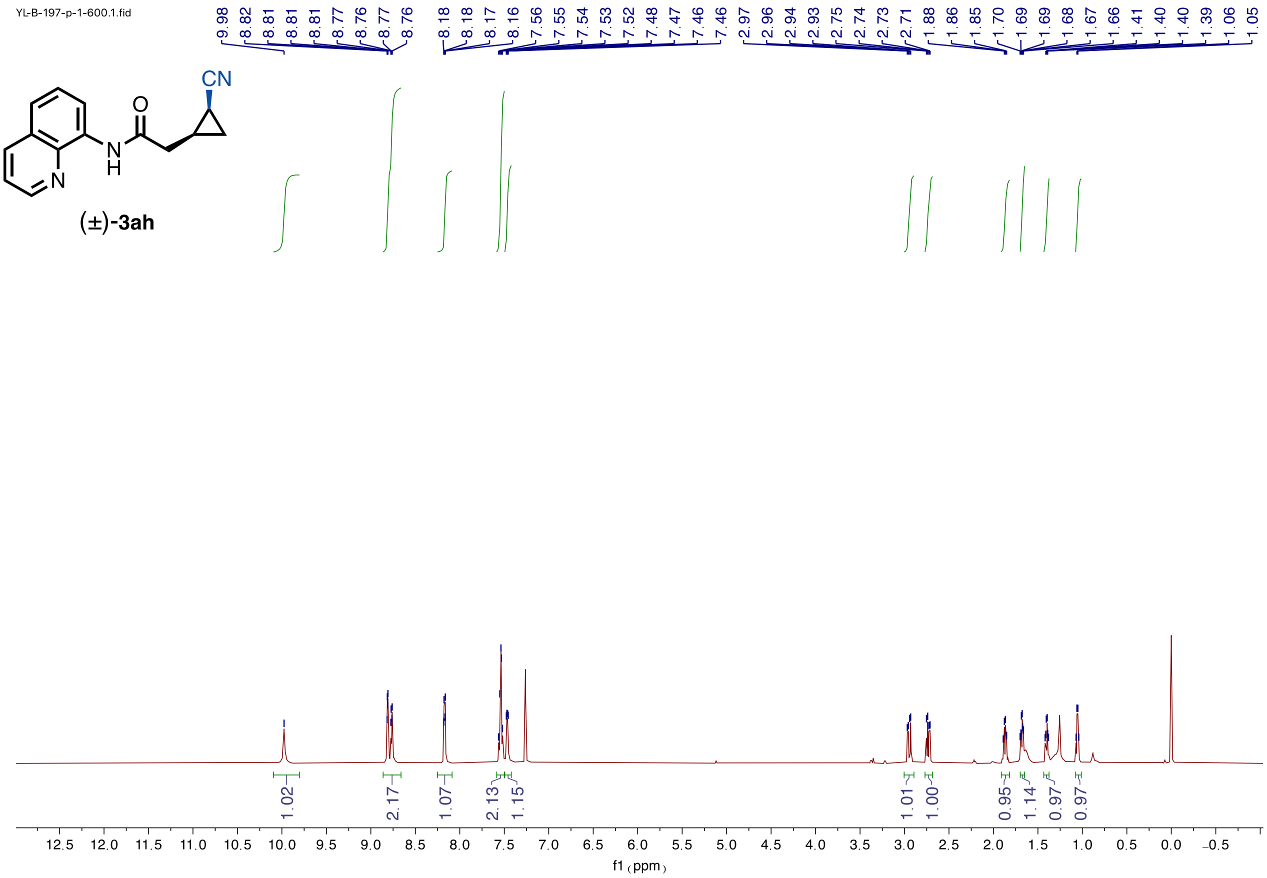
YL-B-197-p-1-600.1.fid
9.98 8.82 8.81 8.81 8.81 8.77 8.76 8.77 8.76 8.18 8.18 8.17 8.16 7.56 7.55 7.54 7.53 7.52 7.48 7.47 7.46 7.46 2.97 2.96 2.94 2.93 2.75 2.74 2.73 2.71 1.88 1.86 1.85 1.70 1.69 1.69 1.68 1.67 1.66 1.41 1.40 1.40 1.39 1.06 1.05
CN
O
N
H
N
(±)-3ah
1.02 2.17 1.07 2.13 1.15 1.01 1.00 0.95 1.14 0.97 0.97
12.5 12.0 11.5 11.0 10.5 10.0 9.5 9.0 8.5 8.0 7.5 7.0 6.5 6.0 5.5 5.0 4.5 4.0 3.5 3.0 2.5 2.0 1.5 1.0 0.5 0.0 -0.5
f1 (ppm)

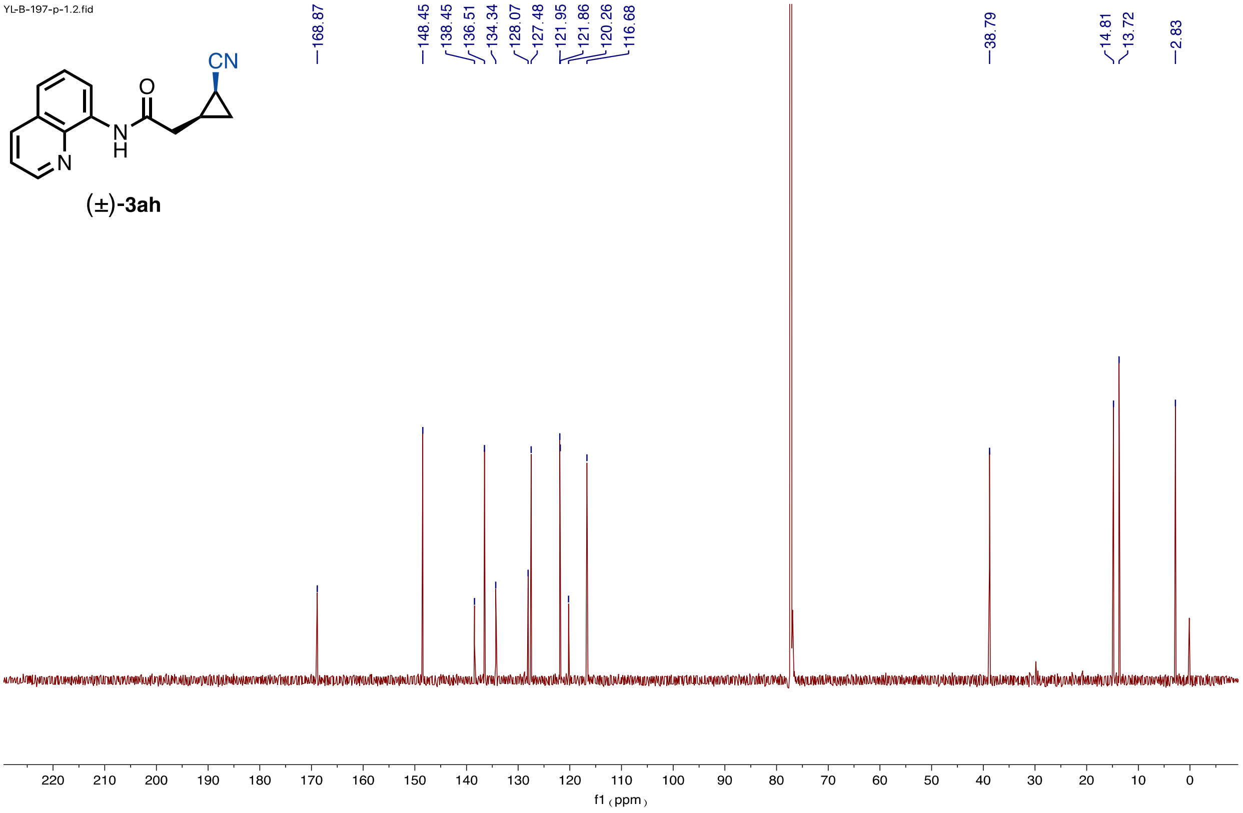
YL-B-197-p-1.2.fid
168.87 148.45 138.45 136.51 134.34 128.07 127.48 121.95 121.86 120.26 116.68 38.79 14.81 13.72 2.83
CN
O
N
H
N
(±)-3ah
220 210 200 190 180 170 160 150 140 130 120 110 100 90 80 70 60 50 40 30 20 10 0
f1 (ppm)

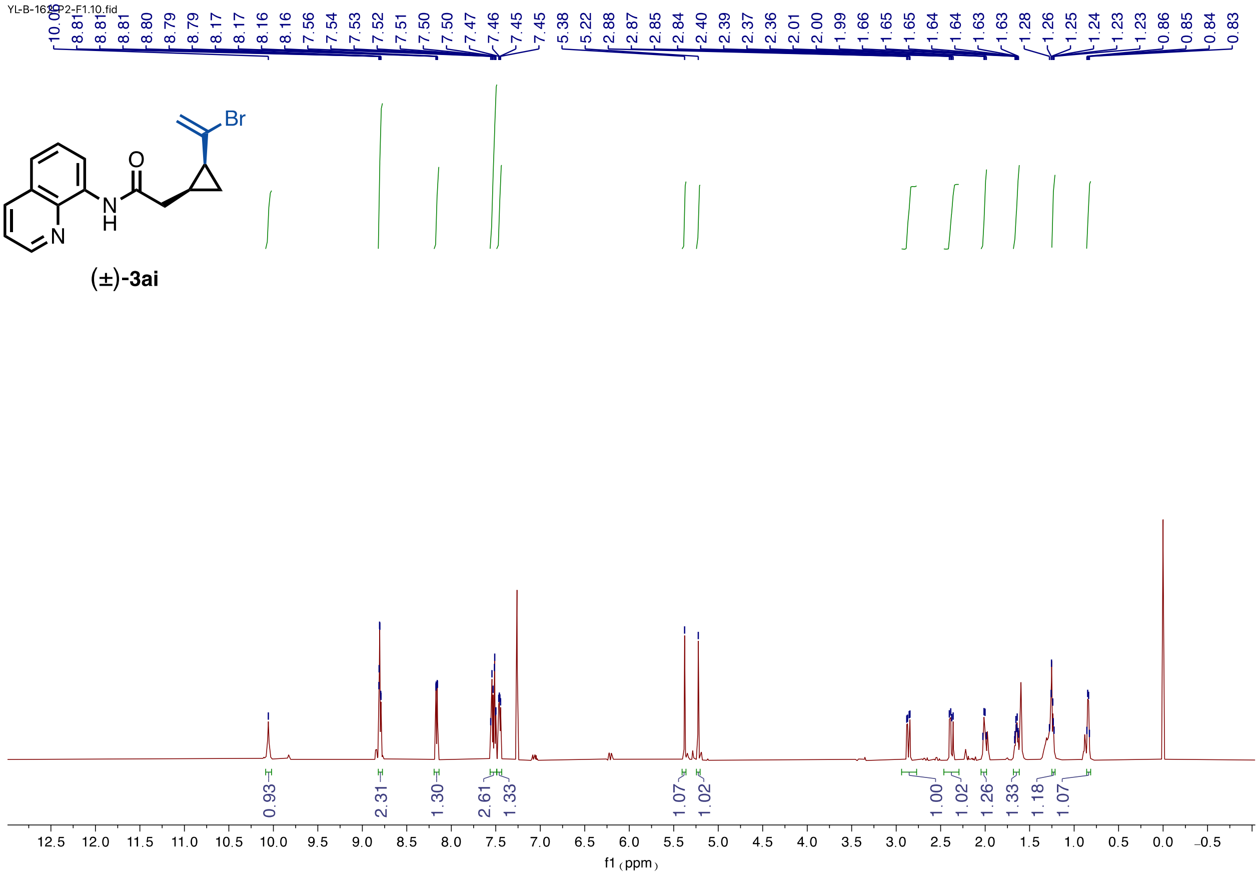
YL-B-162-P2-F1.10.fid
10.06 8.81 8.81 8.81 8.80 8.79 8.79 8.17 8.17 8.16 8.16 7.56 7.54 7.53 7.52 7.51 7.50 7.50 7.47 7.46 7.45 7.45 5.38 5.22 2.88 2.87 2.85 2.84 2.40 2.39 2.37 2.36 2.01 2.00 1.99 1.66 1.65 1.65 1.64 1.64 1.63 1.63 1.28 1.26 1.25 1.24 1.23 0.86 0.85 0.84 0.83
Br
O
N
H
N
0.93 2.31 1.30 2.61 1.33 1.07 1.02 1.00 1.02 1.26 1.33 1.18 1.07
f1 (ppm)

(±)-3ai

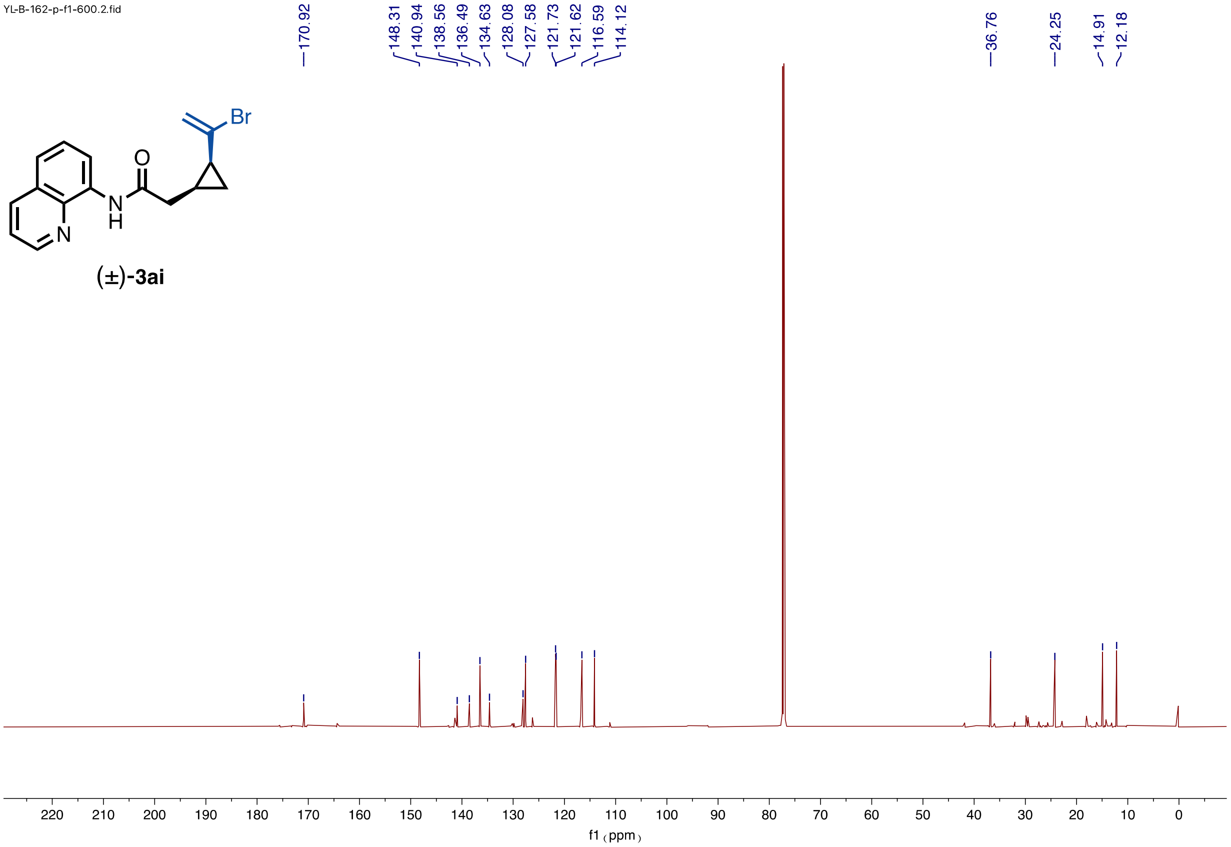
YL-B-162-p-f1-600.2.fid
170.92
148.31 140.94 138.56 136.49 134.63 128.08 127.58 121.73 121.62 116.59 114.12
36.76
24.25
14.91 12.18
Br
O
N
H
N
f1 (ppm)

(±)-3ai

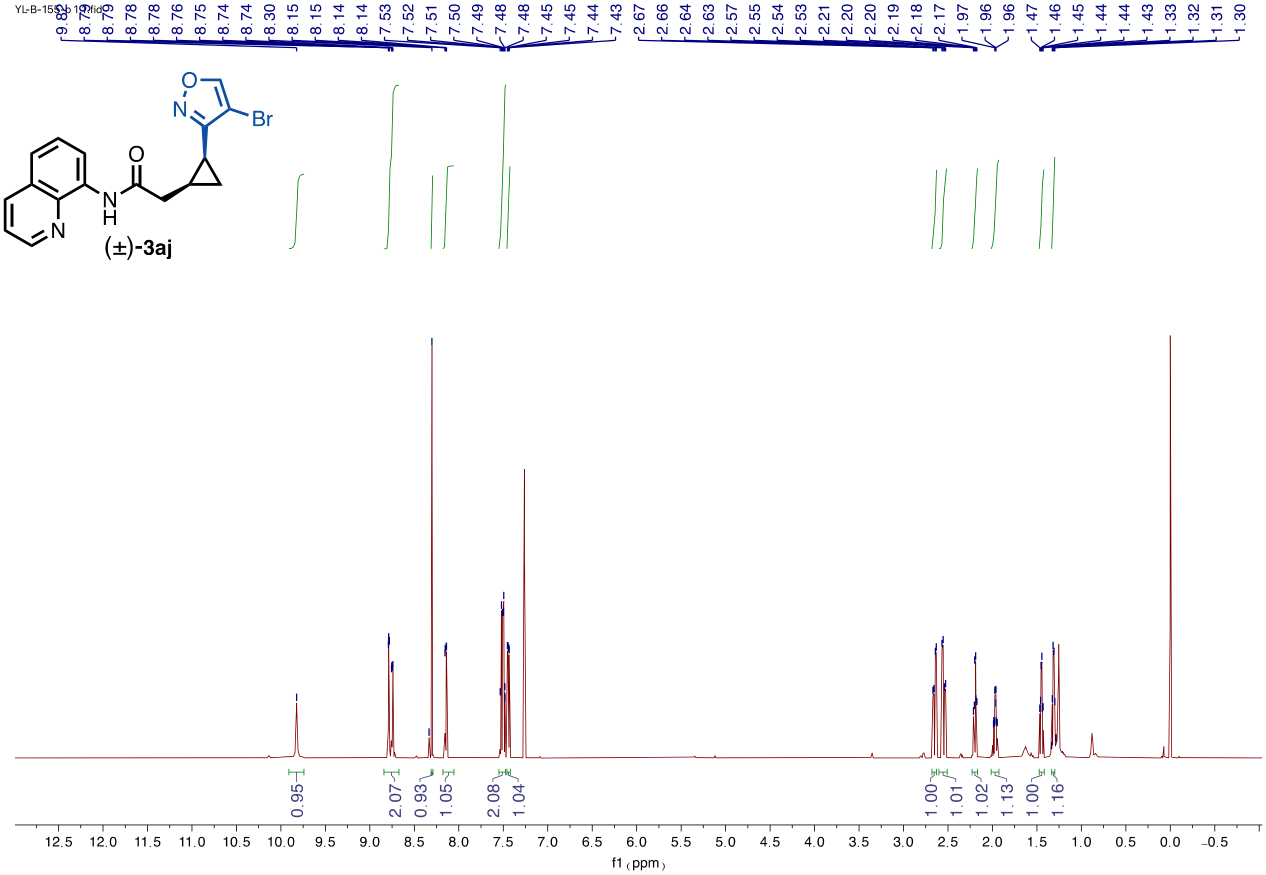
8.79
8.79
8.78
8.78
8.76
8.75
8.74
8.74
8.30
8.15
8.15
8.14
8.14
7.53
7.52
7.51
7.50
7.49
7.48
7.48
7.45
7.45
7.44
7.43
2.67
2.66
2.64
2.63
2.57
2.55
2.54
2.53
2.21
2.20
2.20
2.19
2.18
2.17
1.97
1.96
1.96
1.47
1.46
1.45
1.44
1.44
1.43
1.33
1.32
1.31
1.30
Br
N
O
N
H
(±)-3aj
0.95
2.07
0.93
1.05
2.08
1.04
1.00
1.01
1.02
1.13
1.00
1.16
f1 (ppm)

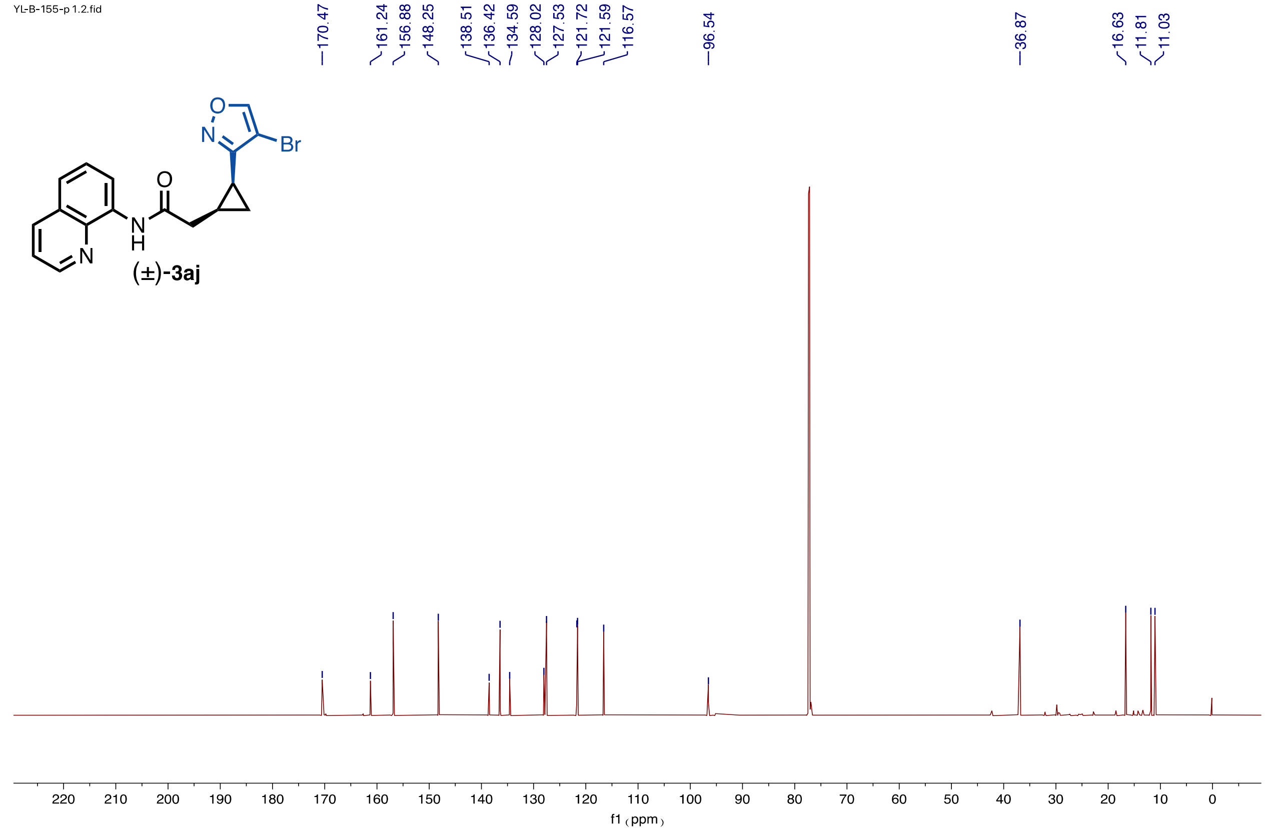
YL-B-155-p 1.2.fid
170.47
161.24
156.88
148.25
138.51
136.42
134.59
128.02
127.53
121.72
121.59
116.57
96.54
36.87
16.63
11.81
11.03
Br
N
O
N
H
(±)-3aj
f1 (ppm)

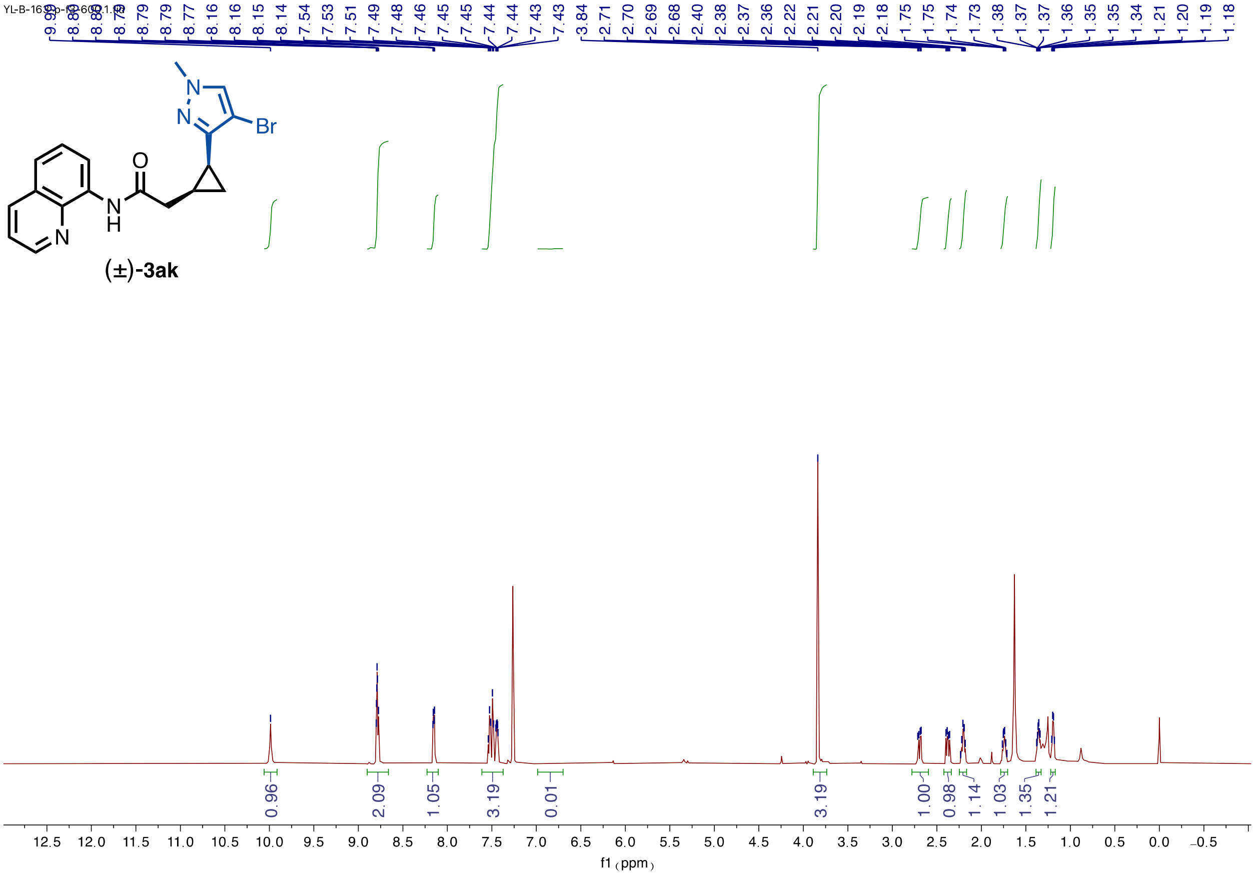
YL-B-163-p-H-600-1.fid
9.99 8.80 8.80 8.79 8.79 8.79 8.77 8.16 8.16 8.15 8.14 7.54 7.53 7.51 7.49 7.48 7.46 7.45 7.45 7.44 7.44 7.43 7.43 3.84 2.71 2.70 2.69 2.68 2.40 2.38 2.37 2.36 2.22 2.21 2.20 2.19 2.18 1.75 1.75 1.74 1.73 1.38 1.37 1.37 1.36 1.35 1.35 1.34 1.21 1.20 1.19 1.18
N
N
Br
O
N
H
N
(±)-3ak
0.96 2.09 1.05 3.19 0.01 3.19 1.00 0.98 1.14 1.03 1.35 1.21
12.5 12.0 11.5 11.0 10.5 10.0 9.5 9.0 8.5 8.0 7.5 7.0 6.5 6.0 5.5 5.0 4.5 4.0 3.5 3.0 2.5 2.0 1.5 1.0 0.5 0.0 -0.5
f1 (ppm)

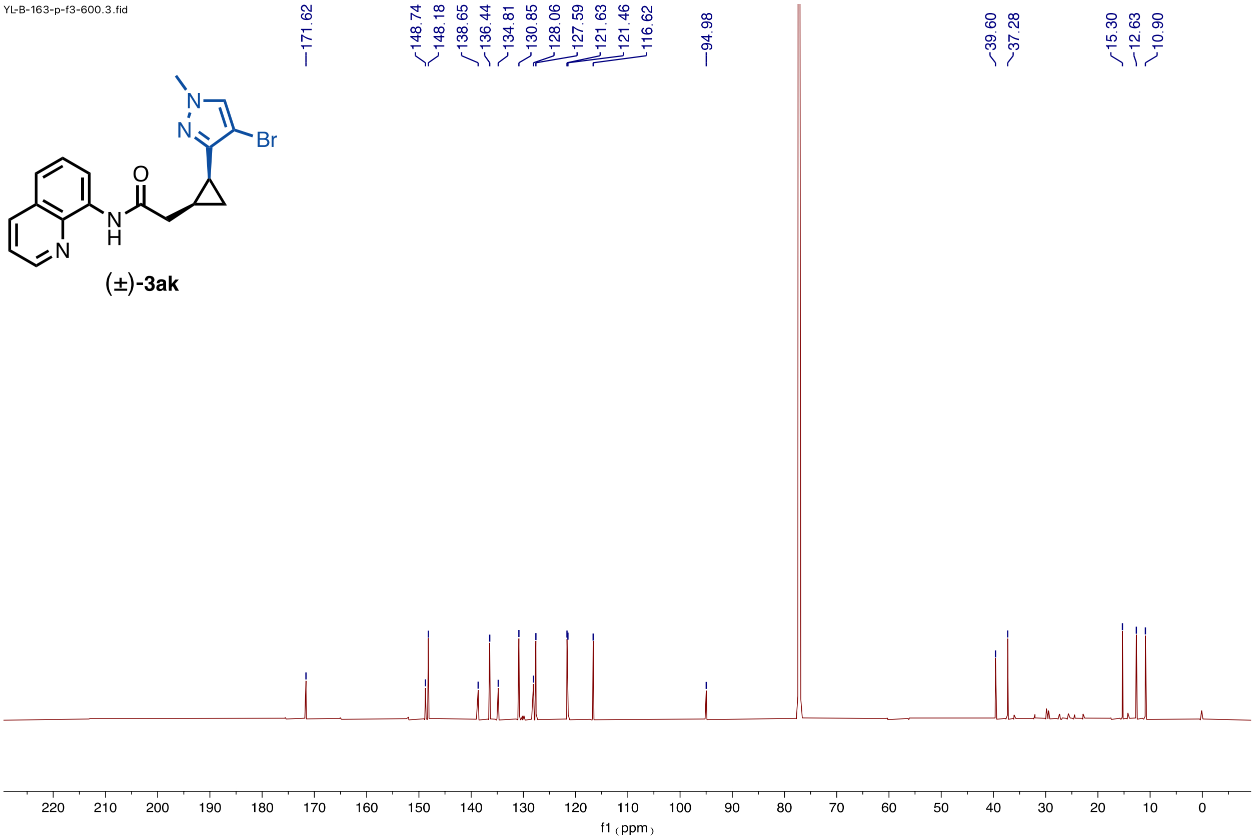
YL-B-163-p-f3-600.3.fid
171.62
148.74 148.18 138.65 136.44 134.81 130.85 128.06 127.59 121.63 121.46 116.62
94.98
39.60 37.28
15.30 12.63 10.90
N
N
Br
O
N
H
N
(±)-3ak
220 210 200 190 180 170 160 150 140 130 120 110 100 90 80 70 60 50 40 30 20 10 0
f1 (ppm)

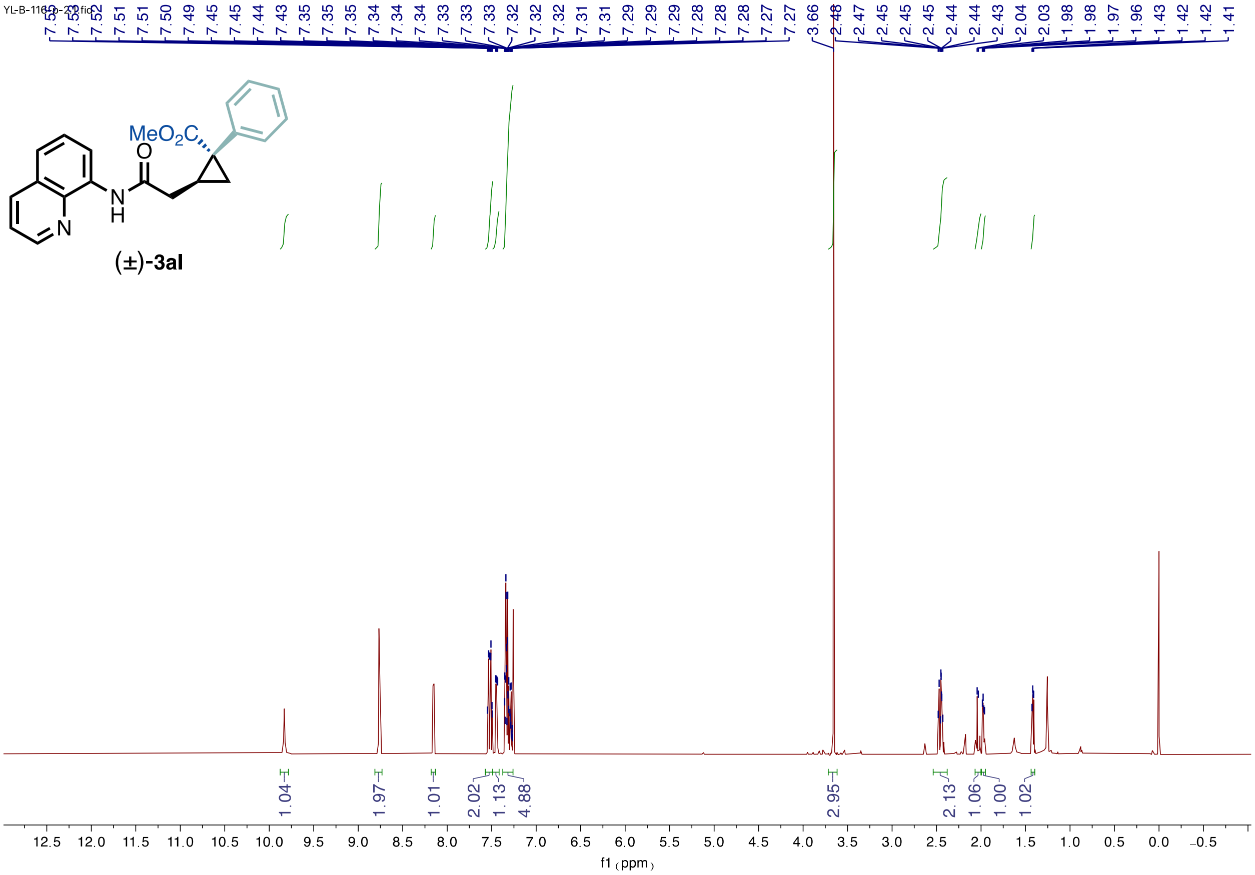
YL-B-116-p-2.1.fid
7.51 7.51 7.50 7.49 7.45 7.45 7.44 7.43 7.35 7.35 7.35 7.34 7.34 7.34 7.33 7.33 7.33 7.32 7.32 7.31 7.31 7.29 7.29 7.29 7.28 7.28 7.27 7.27 3.66 2.48 2.47 2.45 2.45 2.45 2.44 2.44 2.43 2.04 2.03 1.98 1.98 1.97 1.96 1.43 1.42 1.42 1.41
MeO2C
O
N
H
N
(±)-3al
1.04 1.97 1.01 2.02 1.13 4.88 2.95 2.13 1.06 1.00 1.02
f1 (ppm)

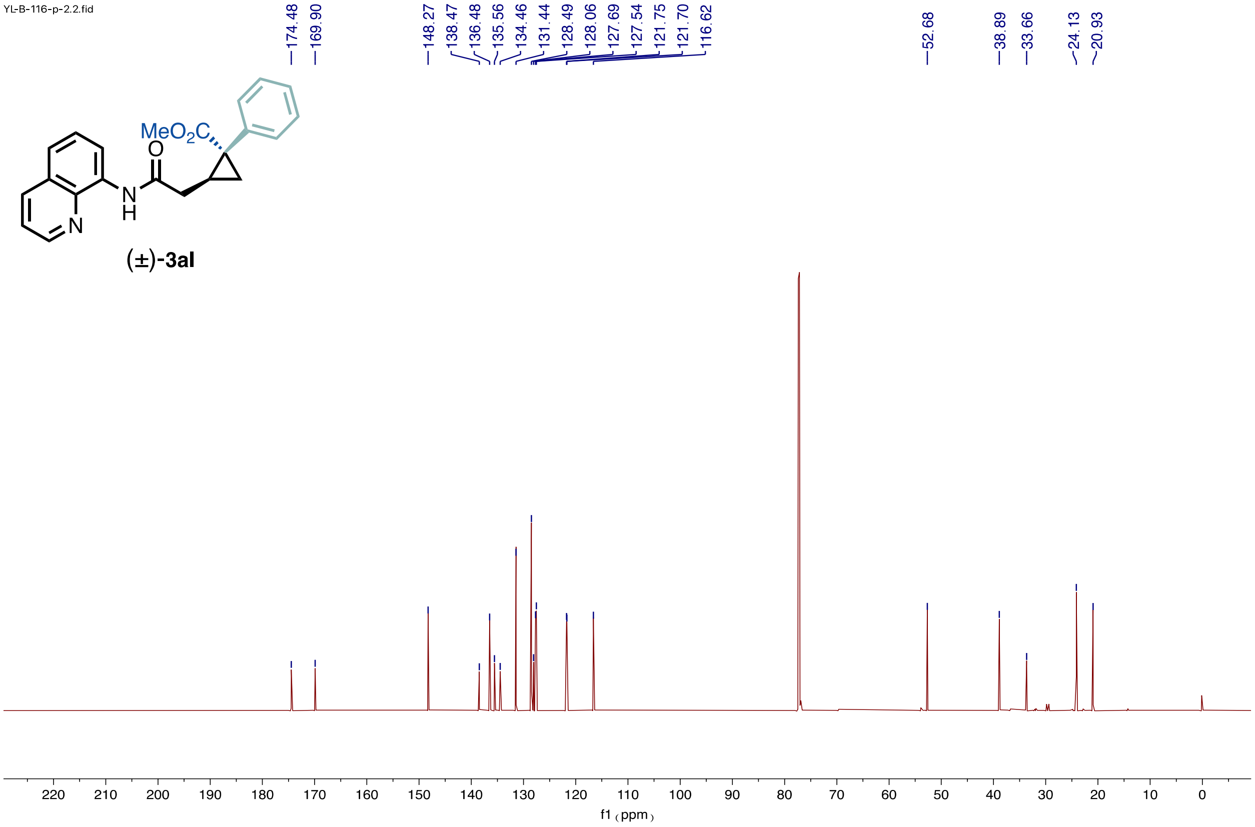
YL-B-116-p-2.2.fid
174.48 169.90 148.27 138.47 136.48 135.56 134.46 131.44 128.49 128.06 127.69 127.54 121.75 121.70 116.62 52.68 38.89 33.66 24.13 20.93
MeO2C
O
N
H
N
(±)-3al
f1 (ppm)

YL-B-116-p-1.1.fid

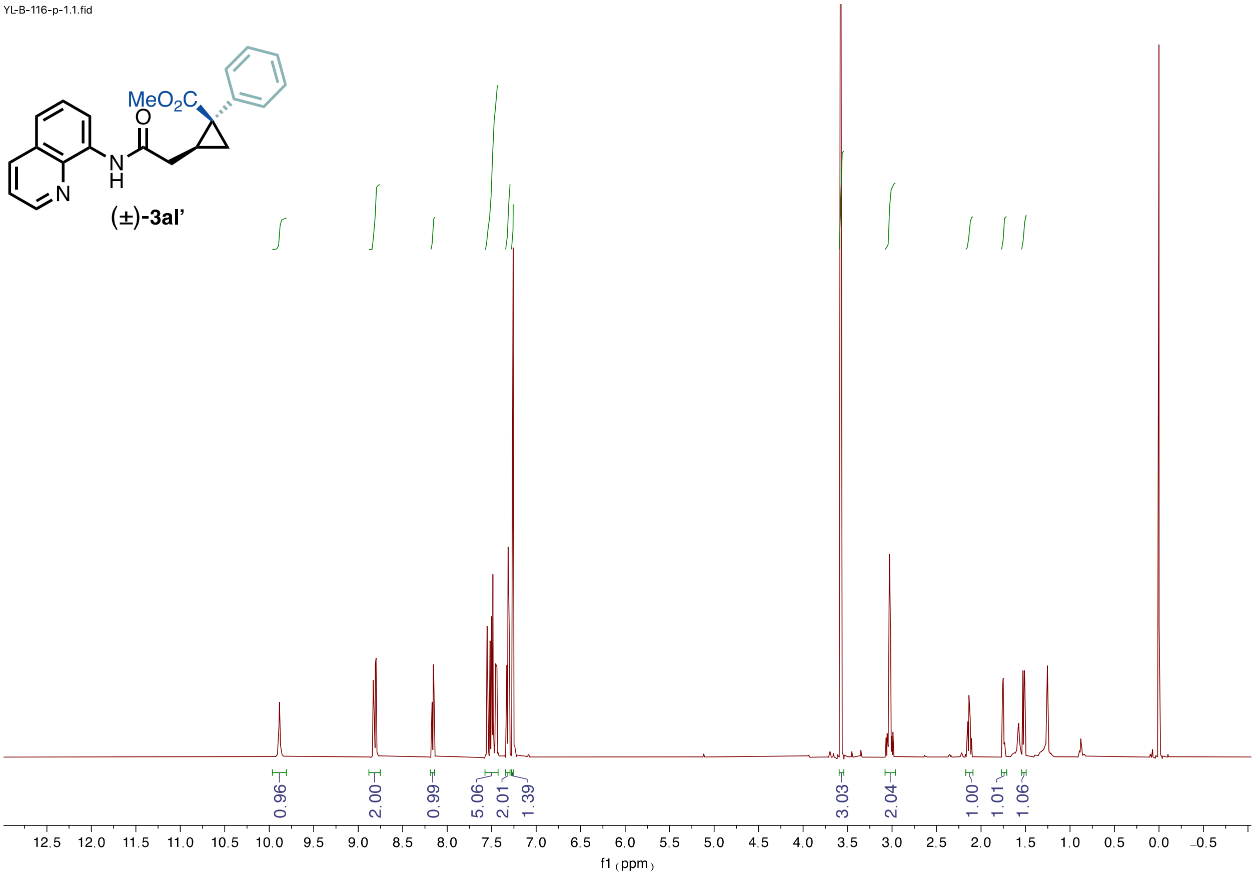

YL-B-116-p-1.2.fid

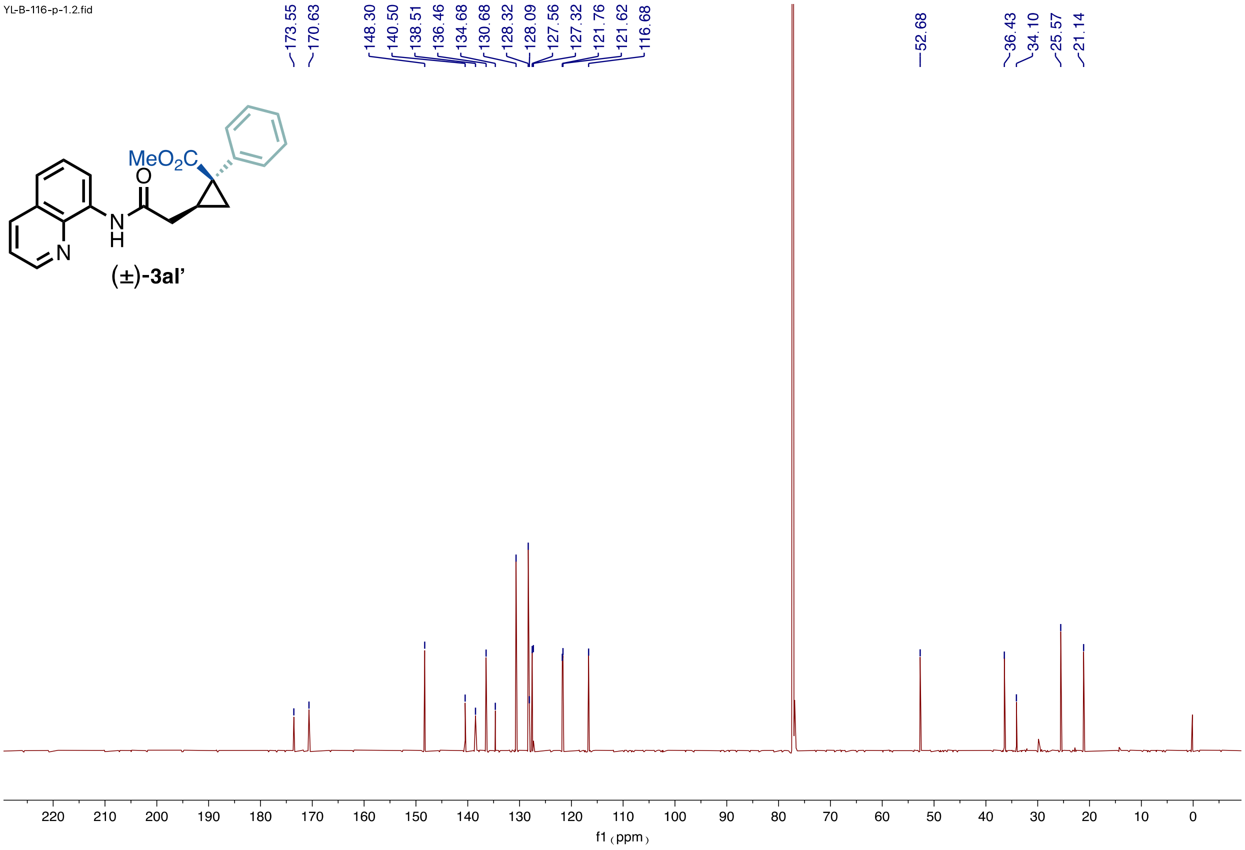

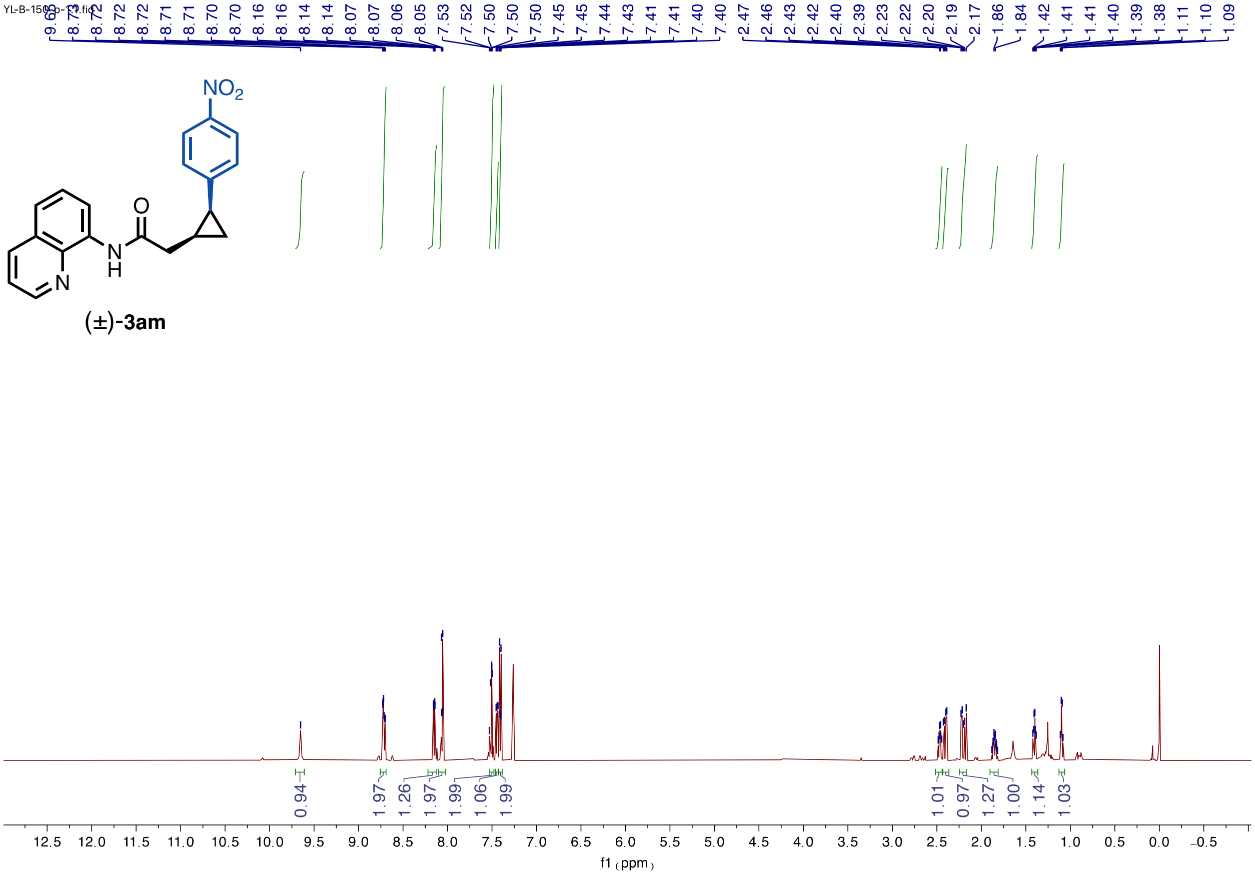
NO2
O
N
H
N
(±)-3am
0.94
1.97
1.26
1.97
1.99
1.06
1.99
1.01
0.97
1.27
1.00
1.14
1.03
f1 (ppm)

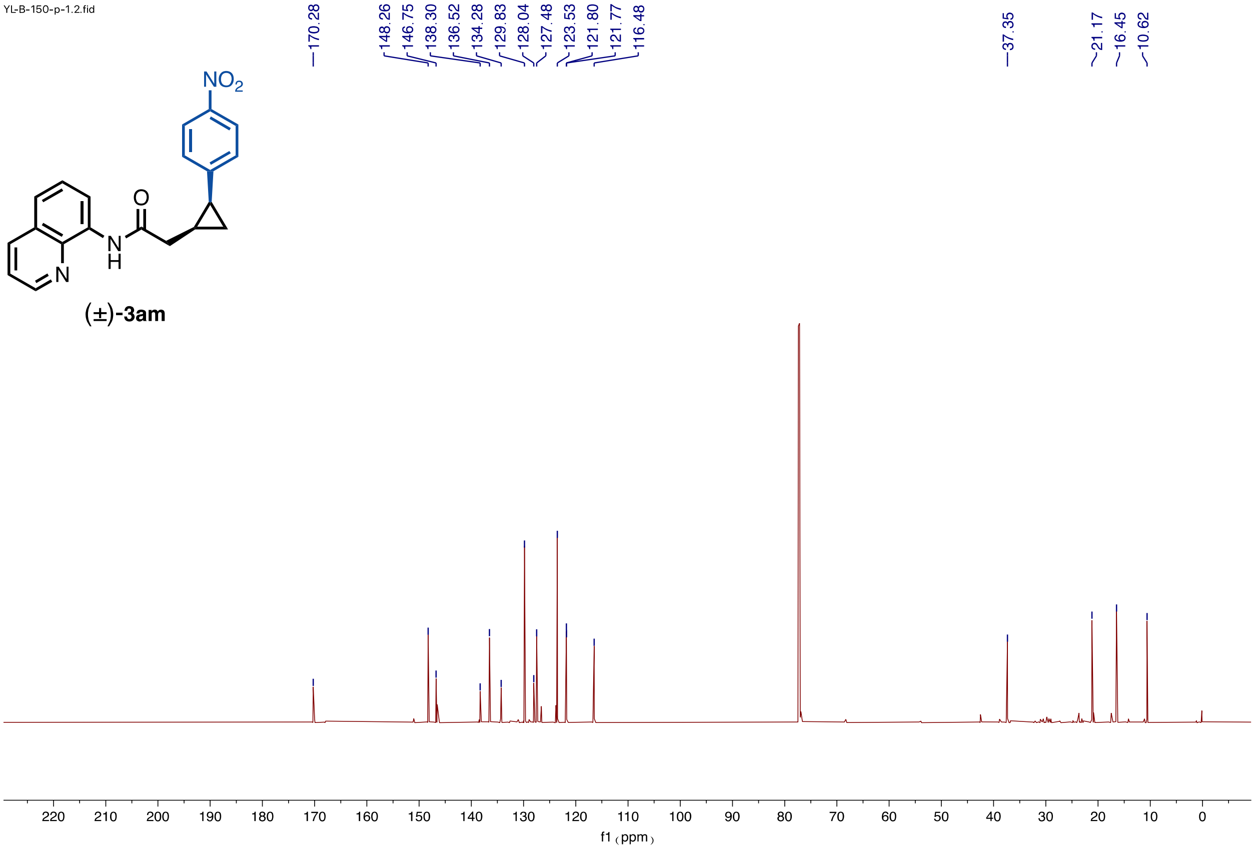
YL-B-150-p-1.2.fid
170.28
148.26
146.75
138.30
136.52
134.28
129.83
128.04
127.48
123.53
121.80
121.77
116.48
37.35
21.17
16.45
10.62
NO2
O
N
H
N
(±)-3am
f1 (ppm)

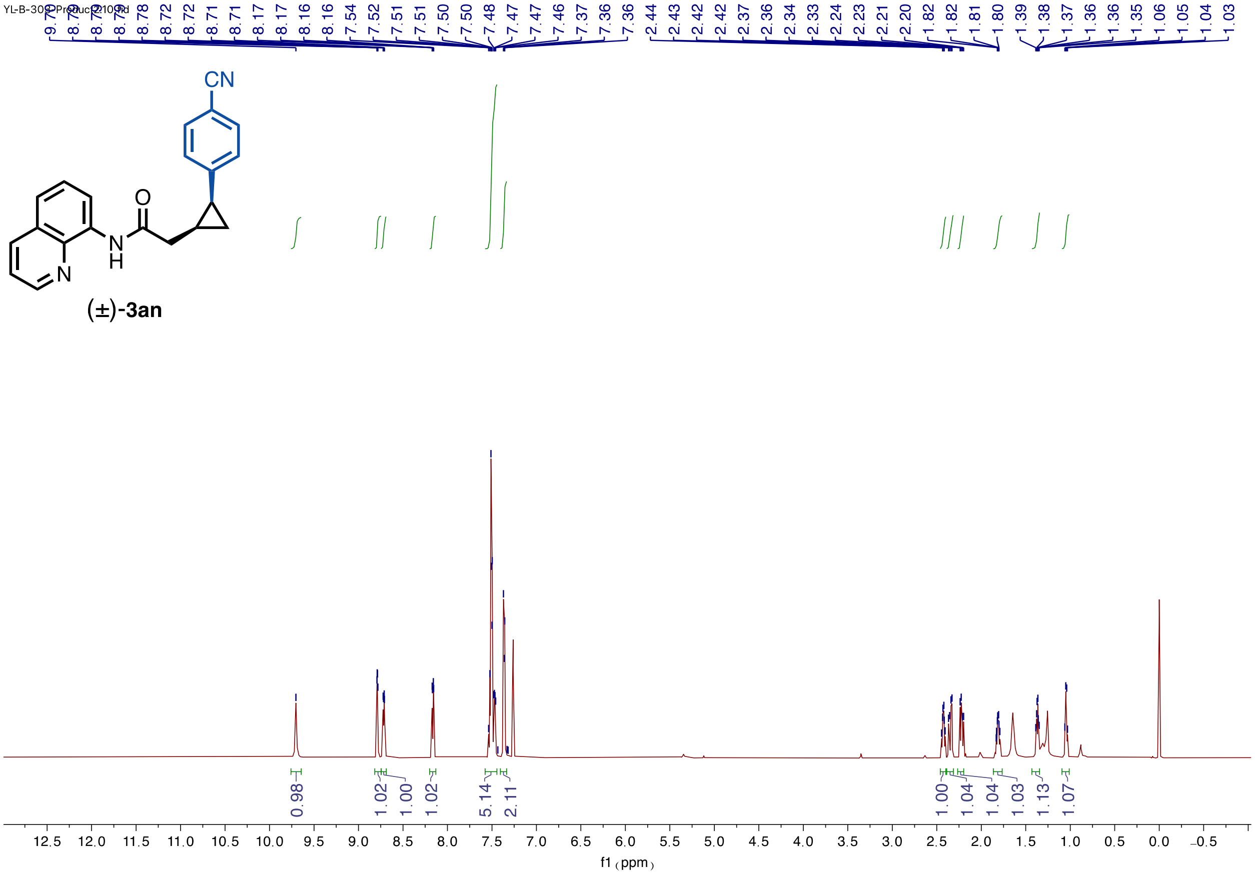
9.70 8.79 8.79 8.78 8.78 8.72 8.72 8.71 8.71 8.17 8.17 8.16 8.16 7.54 7.52 7.51 7.51 7.50 7.50 7.48 7.47 7.47 7.46 7.37 7.36 7.36 2.44 2.43 2.42 2.42 2.37 2.36 2.34 2.33 2.24 2.23 2.21 2.20 1.82 1.82 1.81 1.80 1.39 1.38 1.37 1.36 1.36 1.35 1.06 1.05 1.04 1.03
CN
O
N
H
N
(±)-3an
0.98 1.02 1.00 1.02 5.14 2.11 1.00 1.04 1.04 1.03 1.13 1.07
f1 (ppm)

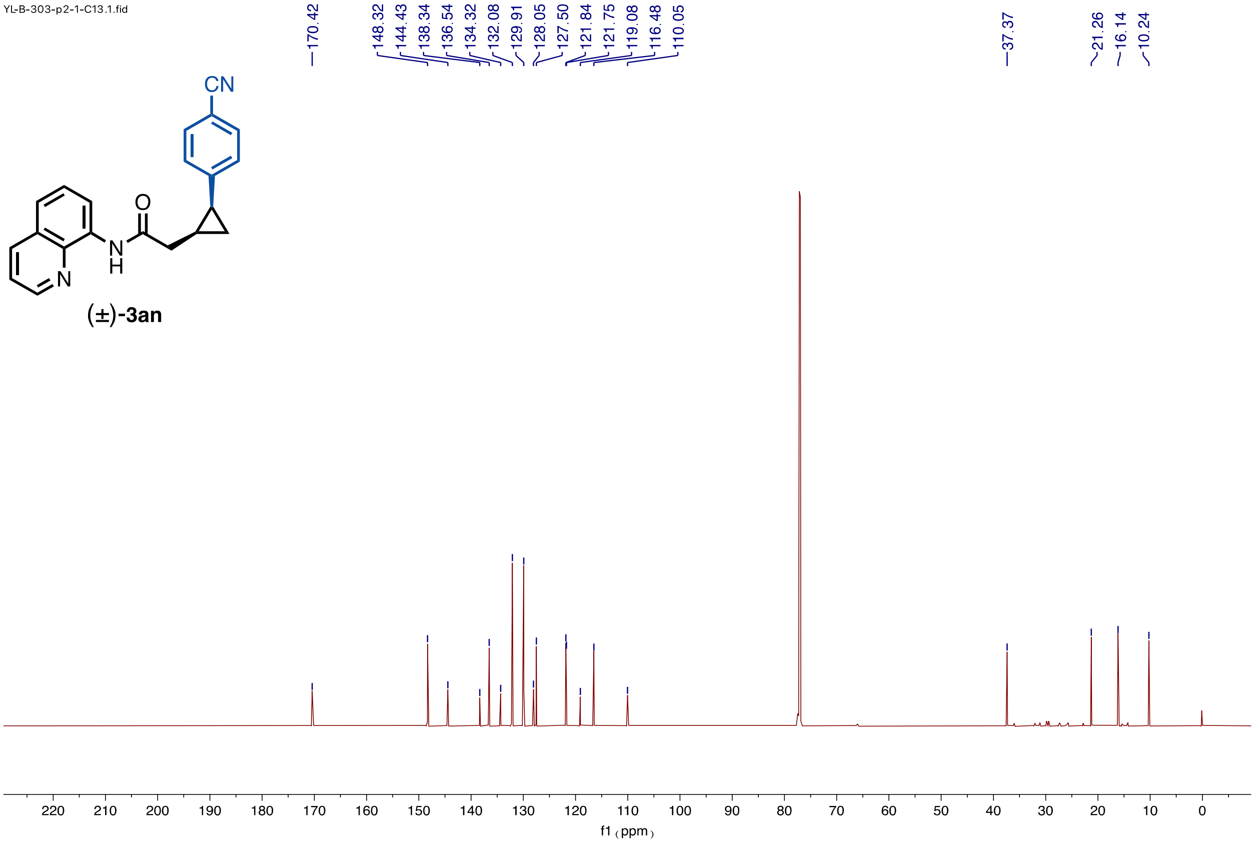
YL-B-303-p2-1-C13.1.fid
170.42 148.32 144.43 138.34 136.54 134.32 132.08 129.91 128.05 127.50 121.84 121.75 119.08 116.48 110.05 37.37 21.26 16.14 10.24
CN
O
N
H
N
(±)-3an
f1 (ppm)

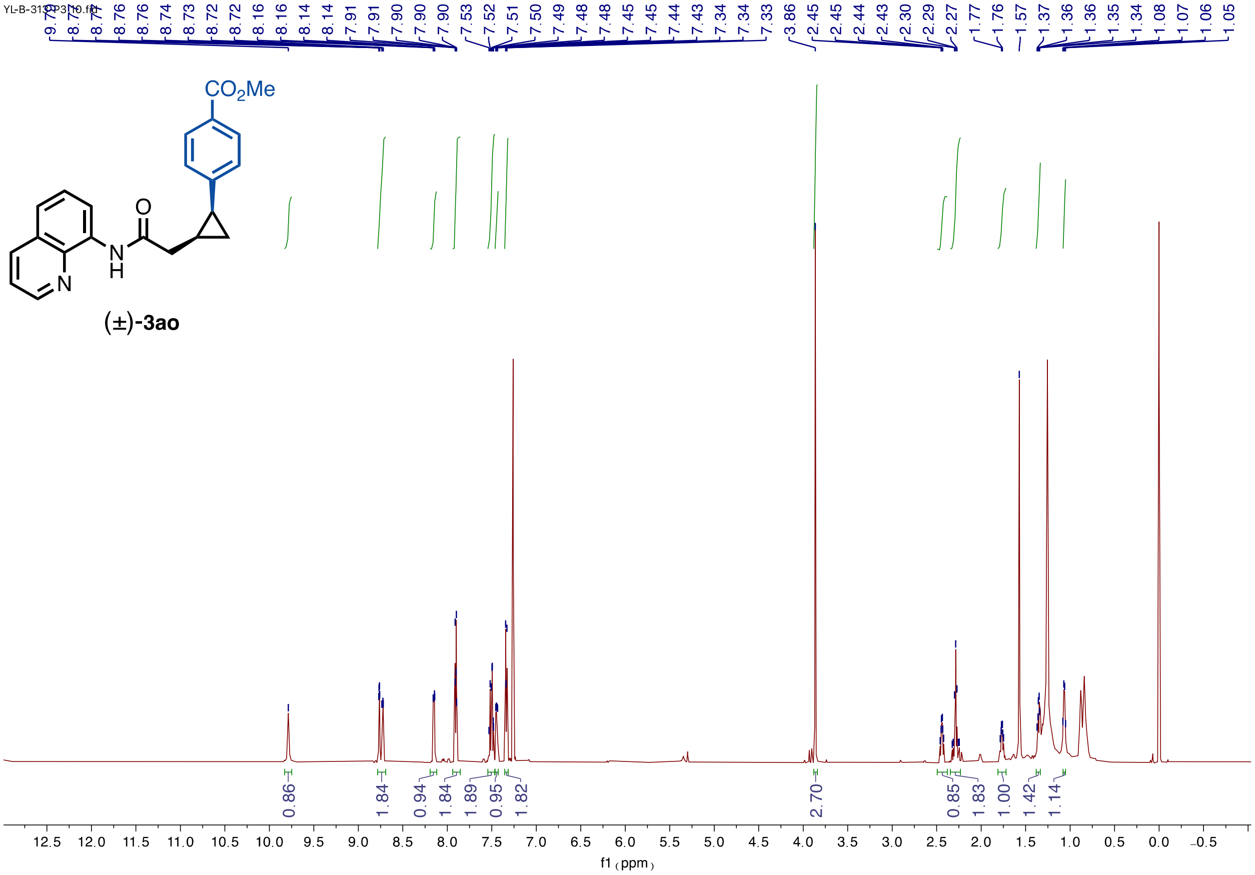
8.76
8.76
8.74
8.73
8.72
8.72
8.16
8.16
8.14
8.14
7.91
7.91
7.90
7.90
7.90
7.53
7.52
7.51
7.50
7.49
7.48
7.45
7.45
7.44
7.43
7.34
7.34
7.33
3.86
2.45
2.45
2.44
2.43
2.30
2.29
2.27
1.77
1.76
1.57
1.37
1.36
1.36
1.35
1.34
1.08
1.07
1.06
1.05
CO_2Me
O
N
H
N
(±)-3ao
0.86
1.84
0.94
1.84
1.89
0.95
1.82
2.70
0.85
1.83
1.00
1.42
1.14
12.5 12.0 11.5 11.0 10.5 10.0 9.5 9.0 8.5 8.0 7.5 7.0 6.5 6.0 5.5 5.0 4.5 4.0 3.5 3.0 2.5 2.0 1.5 1.0 0.5 0.0 -0.5
f1 (ppm)

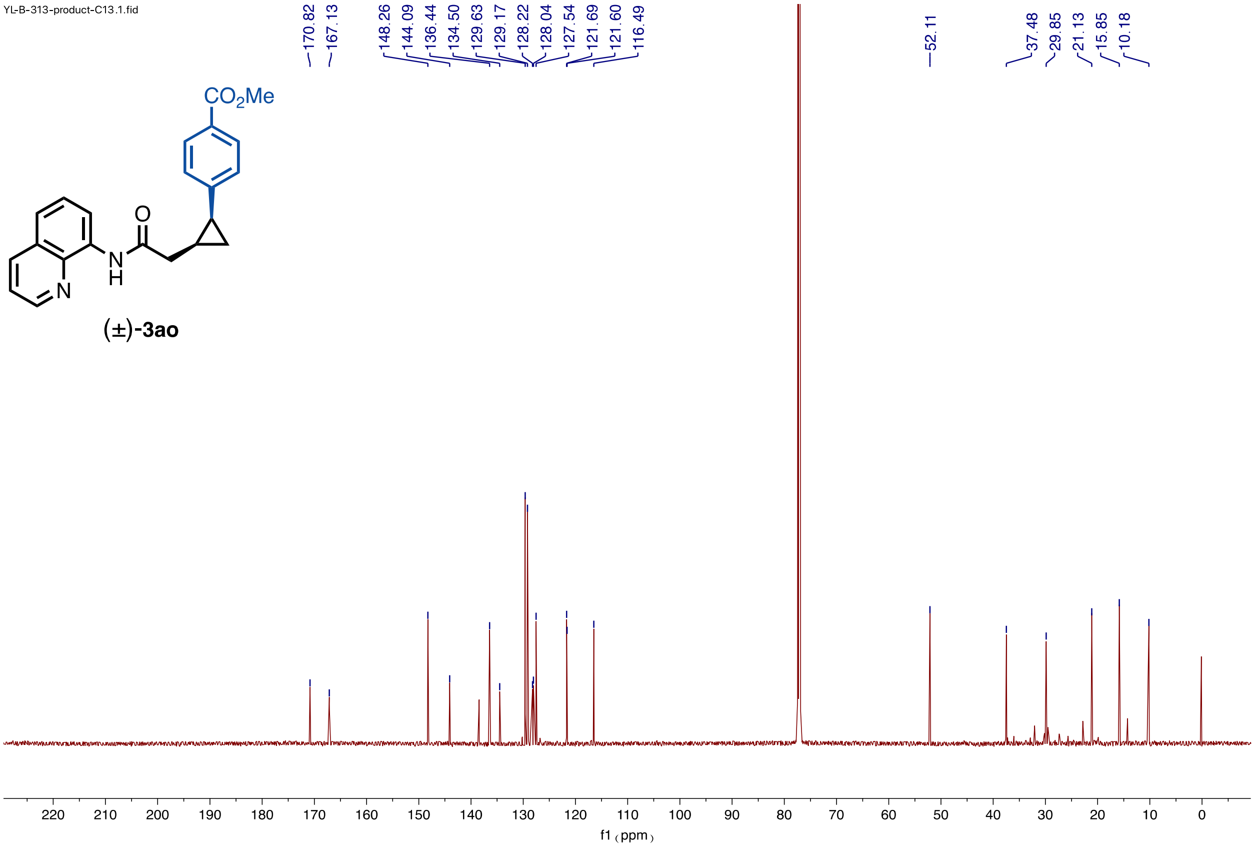
YL-B-313-product-C13.1.fid
170.82
167.13
148.26
144.09
136.44
134.50
129.63
129.17
128.22
128.04
127.54
121.69
121.60
116.49
52.11
37.48
29.85
21.13
15.85
10.18
CO_2Me
O
N
H
N
(±)-3ao
220 210 200 190 180 170 160 150 140 130 120 110 100 90 80 70 60 50 40 30 20 10 0
f1 (ppm)

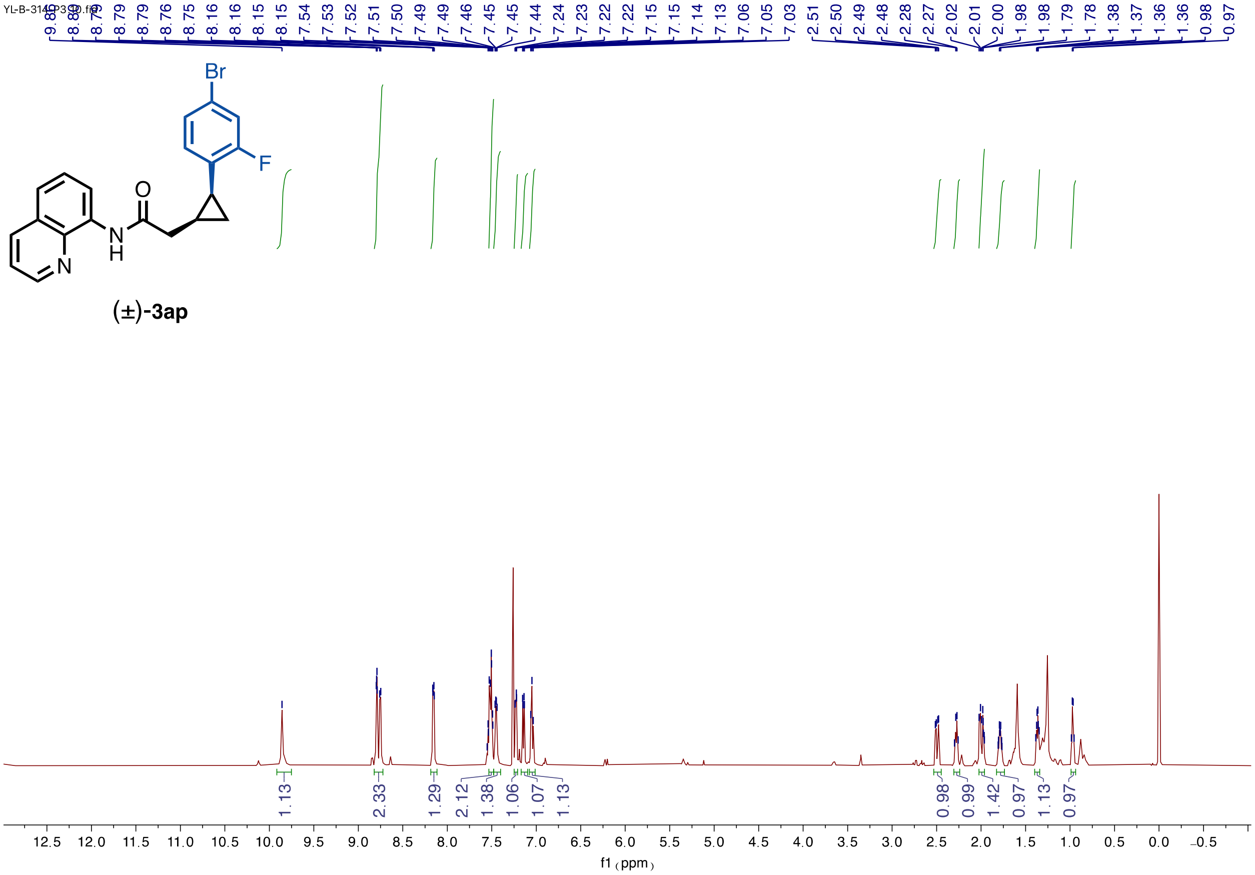
YL-B-314-P3-1
8.79
8.79
8.76
8.75
8.16
8.16
8.15
8.15
7.54
7.53
7.52
7.51
7.50
7.49
7.49
7.46
7.45
7.45
7.44
7.24
7.23
7.22
7.22
7.15
7.15
7.14
7.13
7.06
7.05
7.03
2.51
2.50
2.49
2.48
2.28
2.27
2.02
2.01
2.00
1.98
1.98
1.79
1.78
1.38
1.37
1.36
1.36
0.98
0.97
Br
F
O
N
H
N
(±)-3ap
1.13
2.33
1.29
2.12
1.38
1.06
1.07
1.13
0.98
0.99
1.42
0.97
1.13
0.97
f1 (ppm)

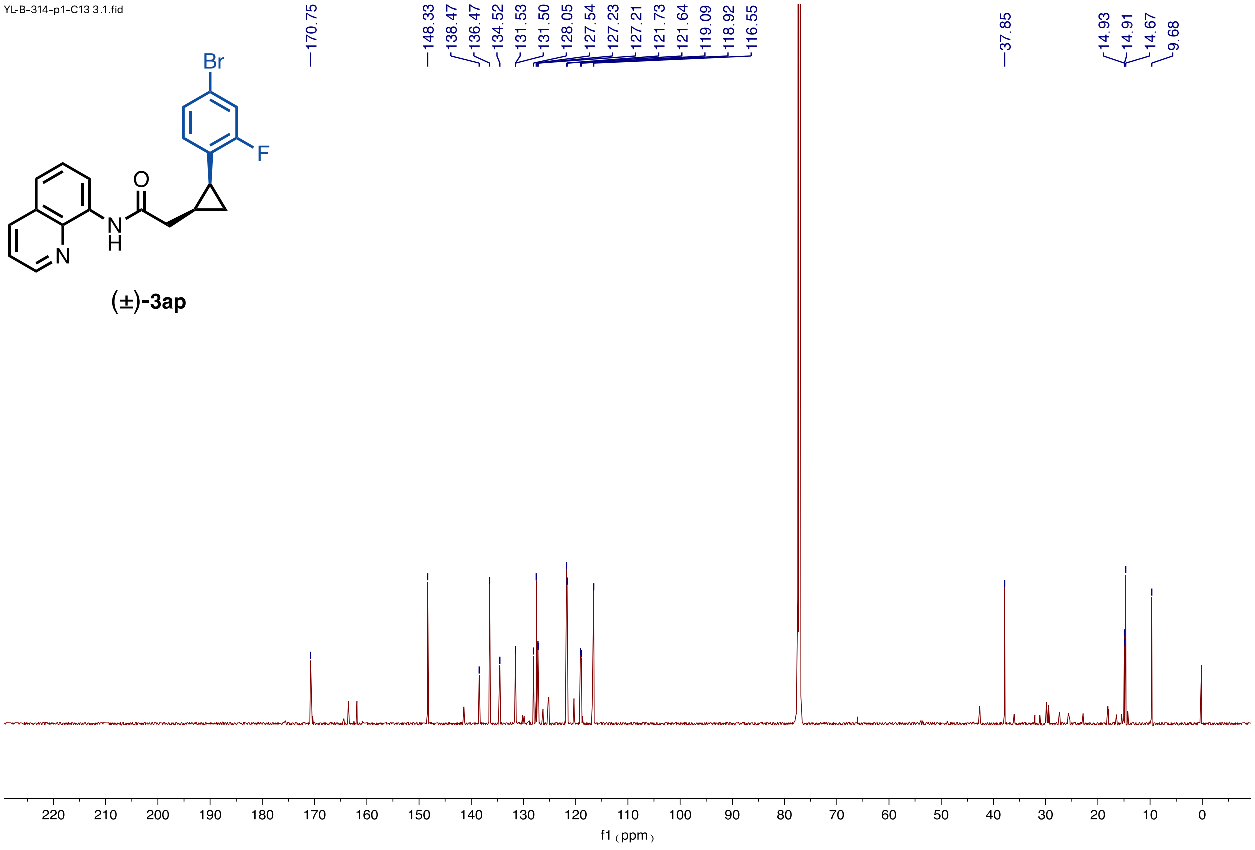
YL-B-314-p1-C13 3.1.fid
170.75
148.33
138.47
136.47
134.52
131.53
131.50
128.05
127.54
127.23
127.21
121.73
121.64
119.09
118.92
116.55
37.85
14.93
14.91
14.67
9.68
Br
F
O
N
H
N
(±)-3ap
f1 (ppm)

YL-B-314-19F 1.10.fid

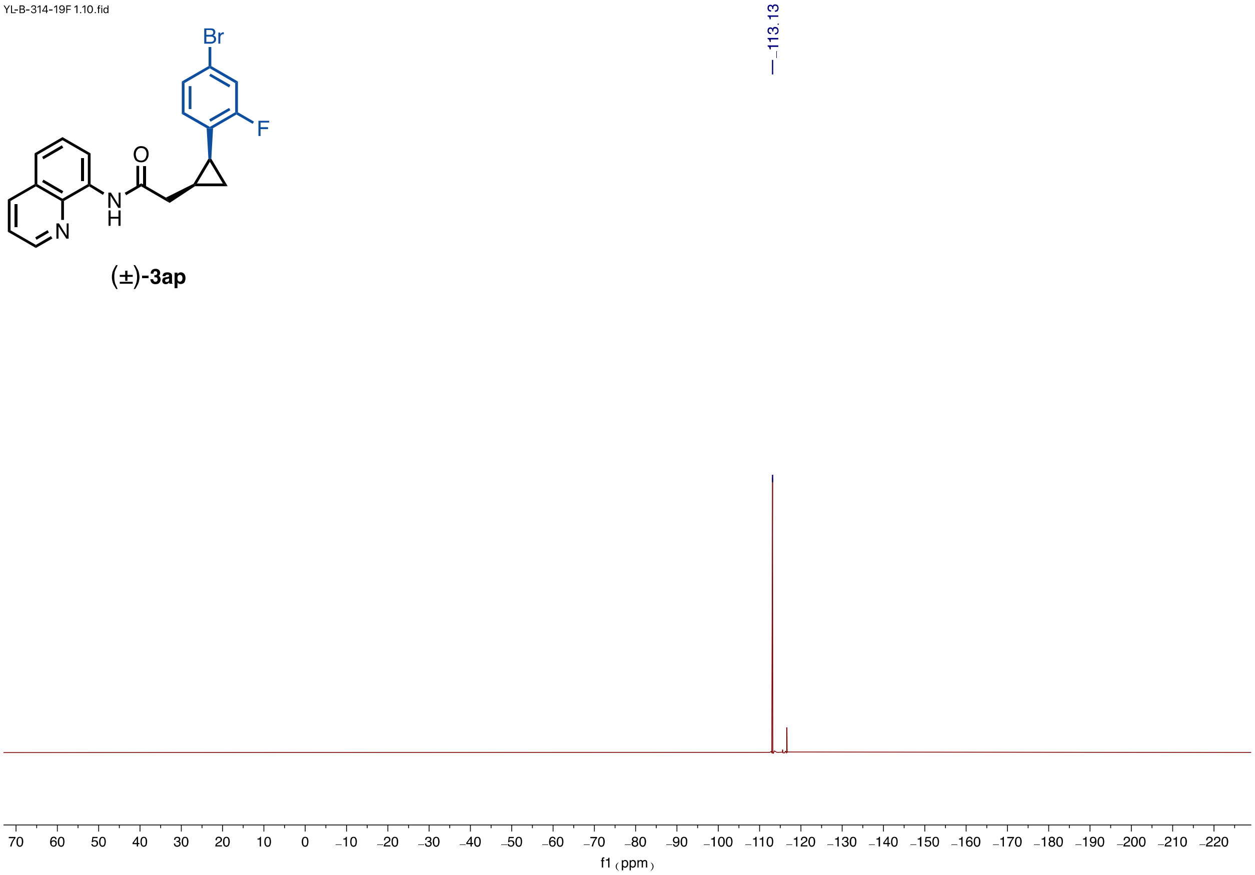

(±)-**3ap**

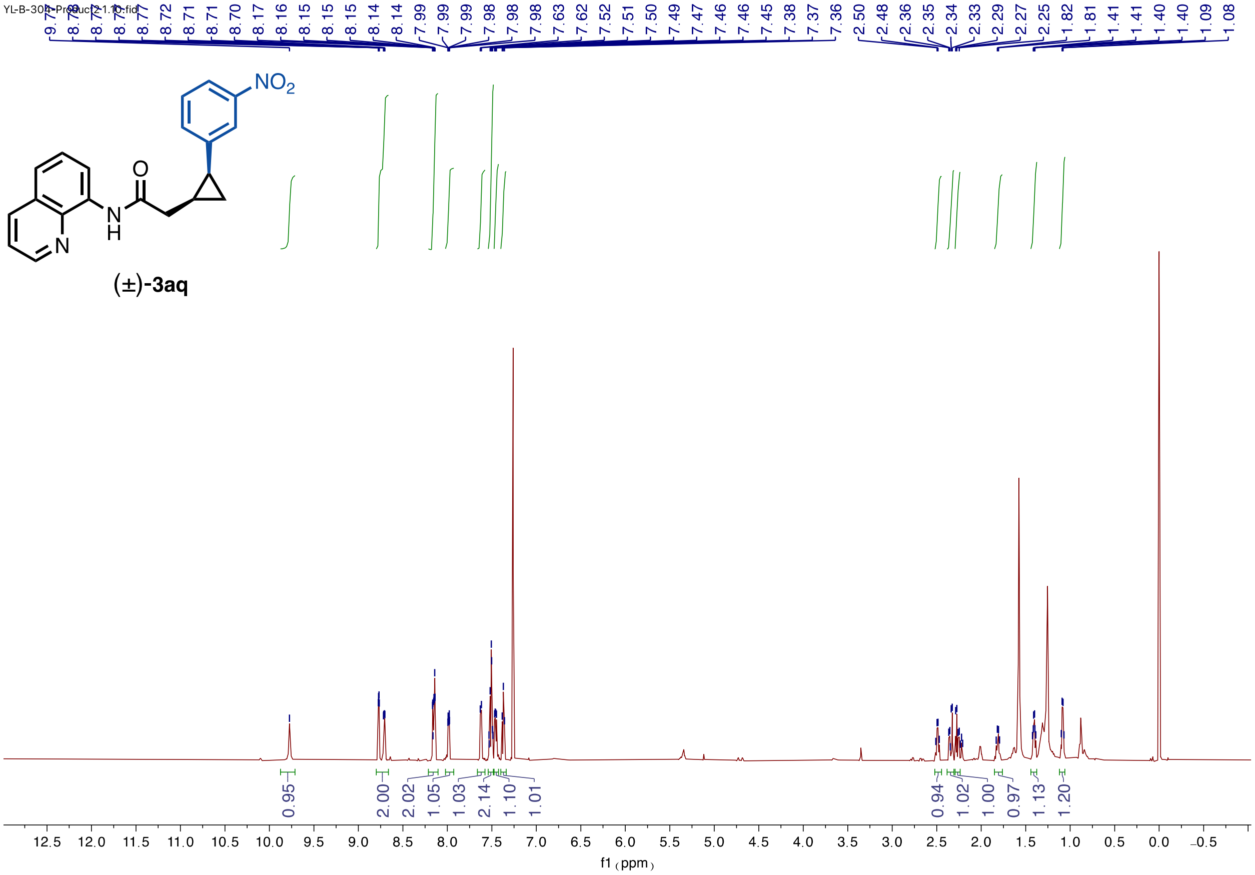
YL-B-304-Product-1.10.fid
9.77
8.79
8.72
8.71
8.70
8.17
8.16
8.15
8.15
8.14
8.14
7.99
7.99
7.98
7.98
7.63
7.62
7.52
7.51
7.50
7.49
7.47
7.46
7.46
7.45
7.38
7.37
7.36
2.50
2.48
2.36
2.35
2.34
2.33
2.29
2.27
2.25
1.82
1.81
1.41
1.41
1.40
1.09
1.08
NO_2
O
N
H
N
(±)-3aq
0.95
2.00
2.02
1.05
1.03
2.14
1.10
1.01
0.94
1.02
1.00
0.97
1.13
1.20
f1 (ppm)

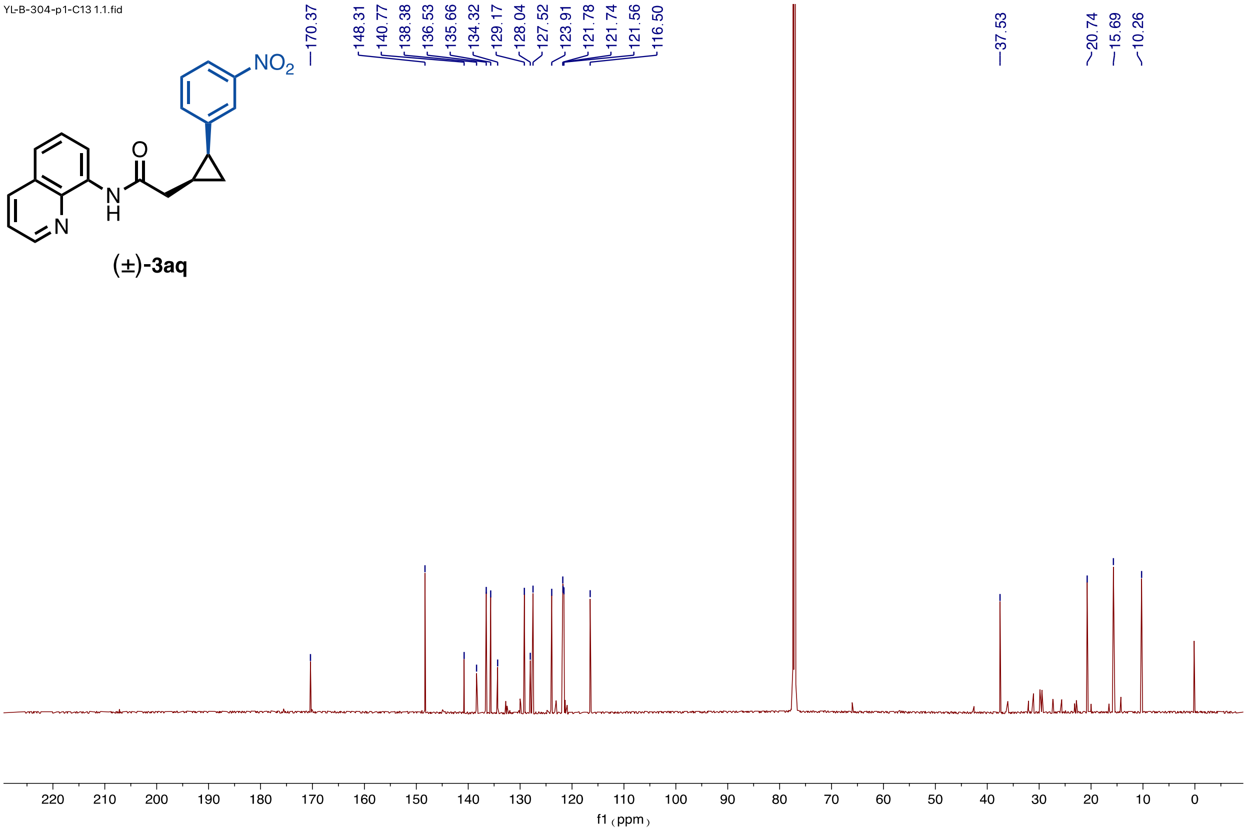
YL-B-304-p1-C13 1.1.fid
170.37
148.31
140.77
138.38
136.53
135.66
134.32
129.17
128.04
127.52
123.91
121.78
121.74
121.56
116.50
37.53
20.74
15.69
10.26
NO_2
O
N
H
N
(±)-3aq
f1 (ppm)

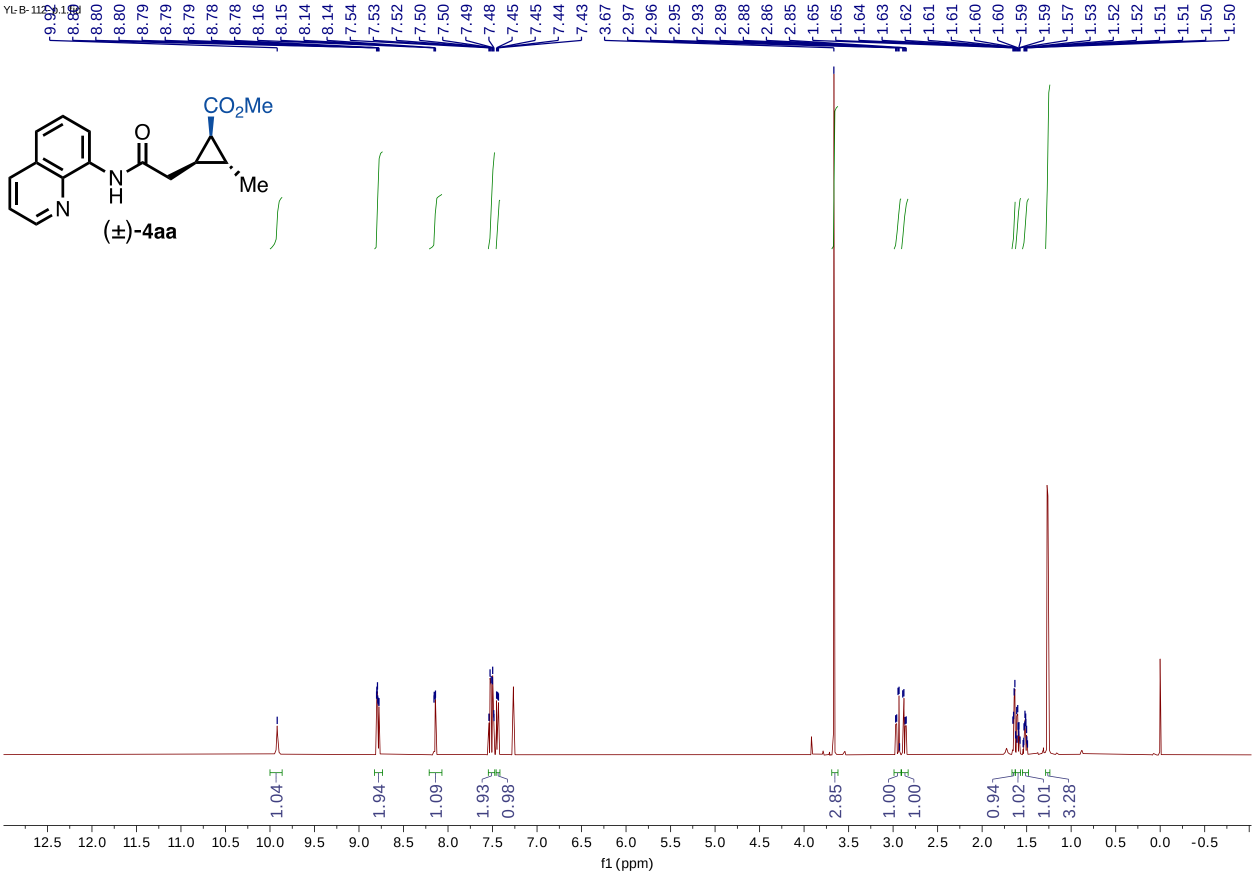
YL-B-112-p.1.fid
8.80 8.80 8.79 8.79 8.79 8.78 8.78 8.16 8.15 8.14 8.14 7.54 7.53 7.52 7.50 7.50 7.49 7.48 7.45 7.45 7.44 7.43 3.67 2.97 2.96 2.95 2.93 2.89 2.88 2.86 2.85 1.65 1.65 1.64 1.63 1.62 1.61 1.61 1.60 1.60 1.59 1.59 1.57 1.53 1.52 1.52 1.51 1.51 1.50 1.50
CO2Me
O
N
H
N
Me
(±)-4aa
1.04
1.94
1.09
1.93
0.98
2.85
1.00
1.00
0.94
1.02
1.01
3.28
f1 (ppm)

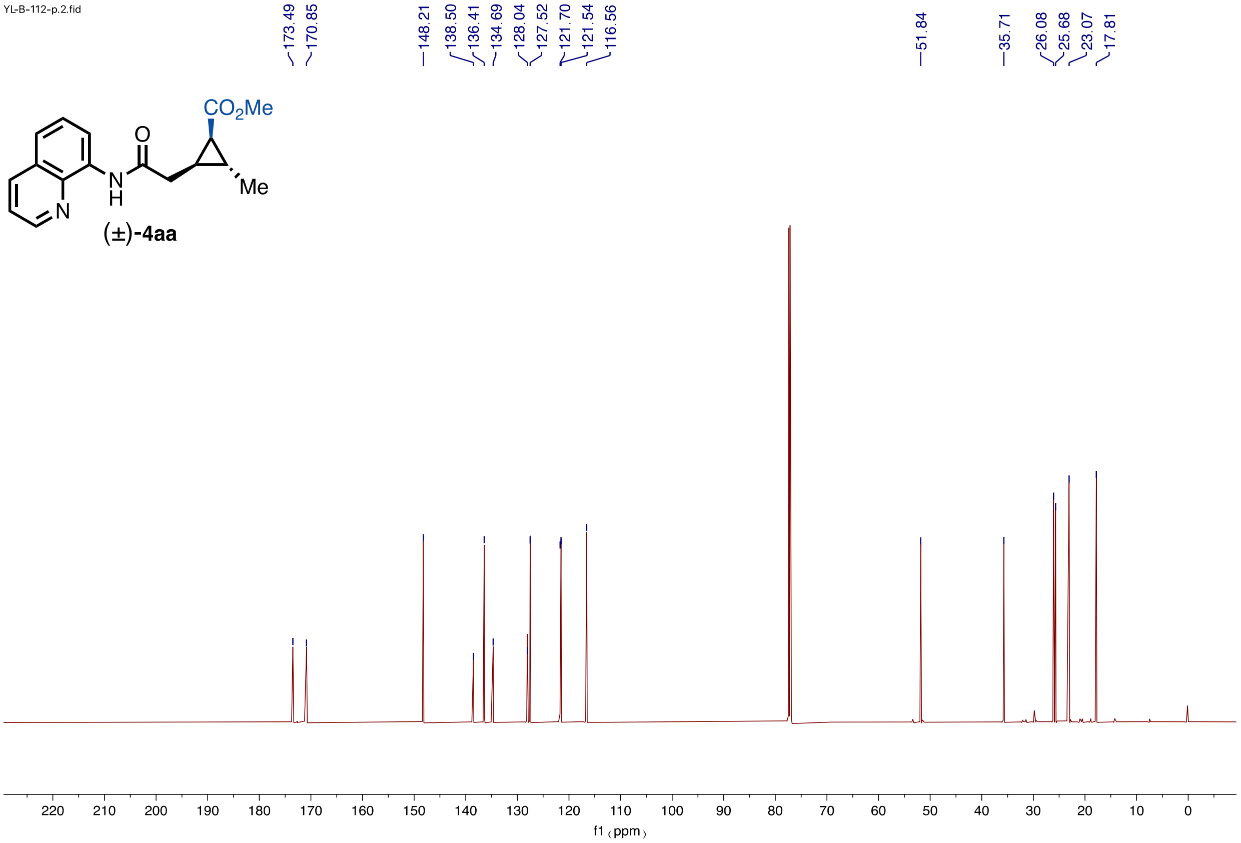
YL-B-112-p.2.fid
173.49 170.85
148.21 138.50 136.41 134.69 128.04 127.52 121.70 121.54 116.56
51.84
35.71 26.08 25.68 23.07 17.81
CO2Me
O
N
H
N
Me
(±)-4aa
f1 (ppm)

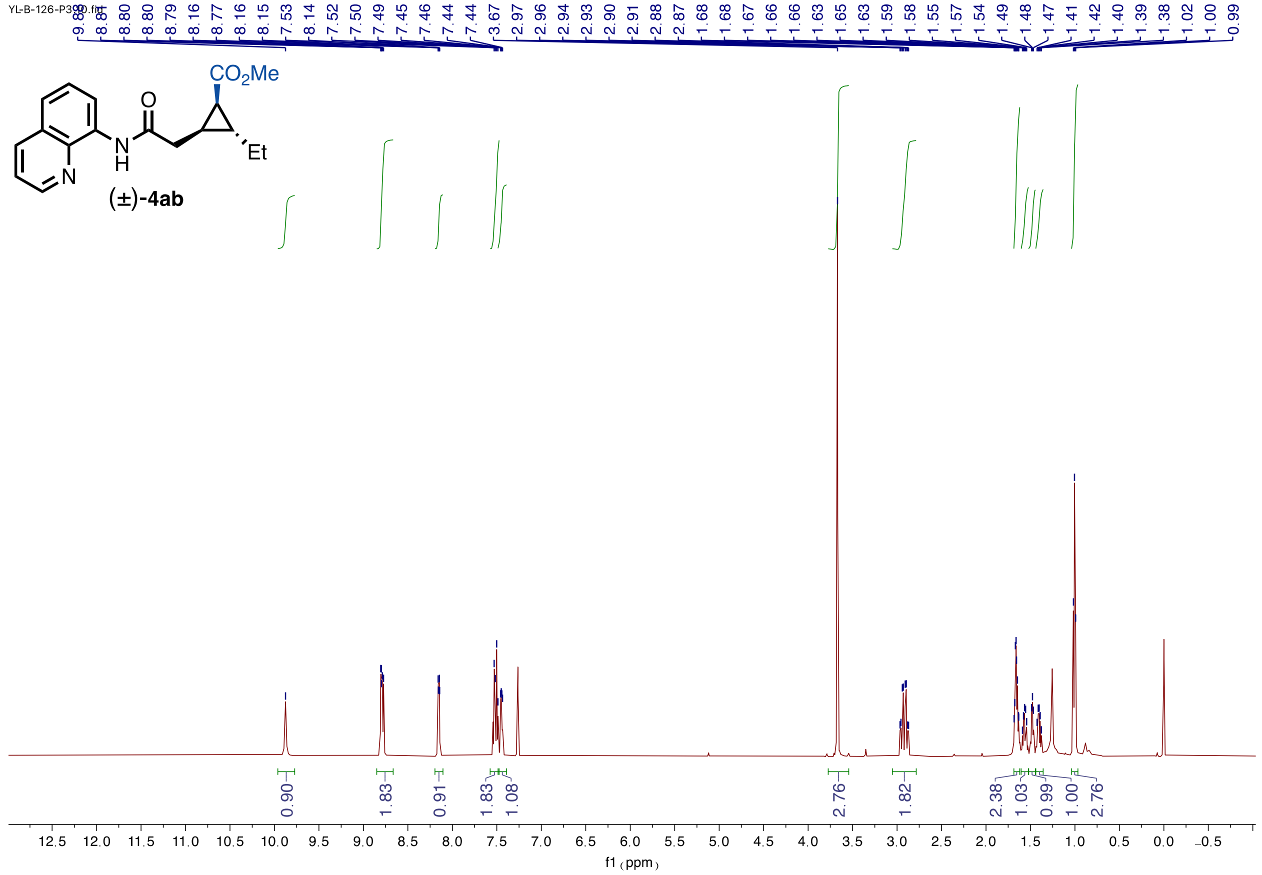
YL-B-126-P300.fid
CO2Me
O
N
H
N
Et
(±)-4ab
0.90
1.83
0.91
1.83
1.08
2.76
1.82
2.38
1.03
0.99
1.00
2.76
f1 (ppm)

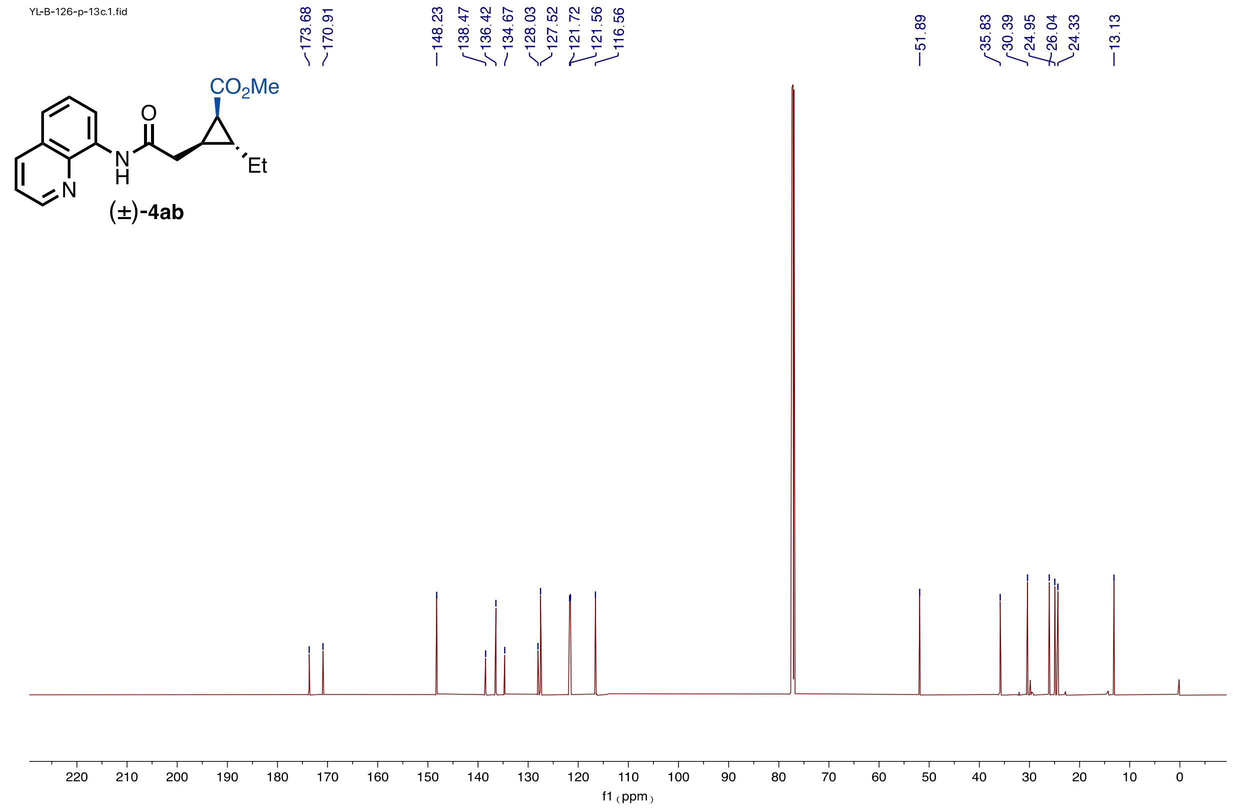
YL-B-126-p-13c.1.fid
173.68
170.91
148.23
138.47
136.42
134.67
128.03
127.52
121.72
121.56
116.56
51.89
35.83
30.39
24.95
26.04
24.33
13.13
CO2Me
O
N
H
N
Et
(±)-4ab
f1 (ppm)

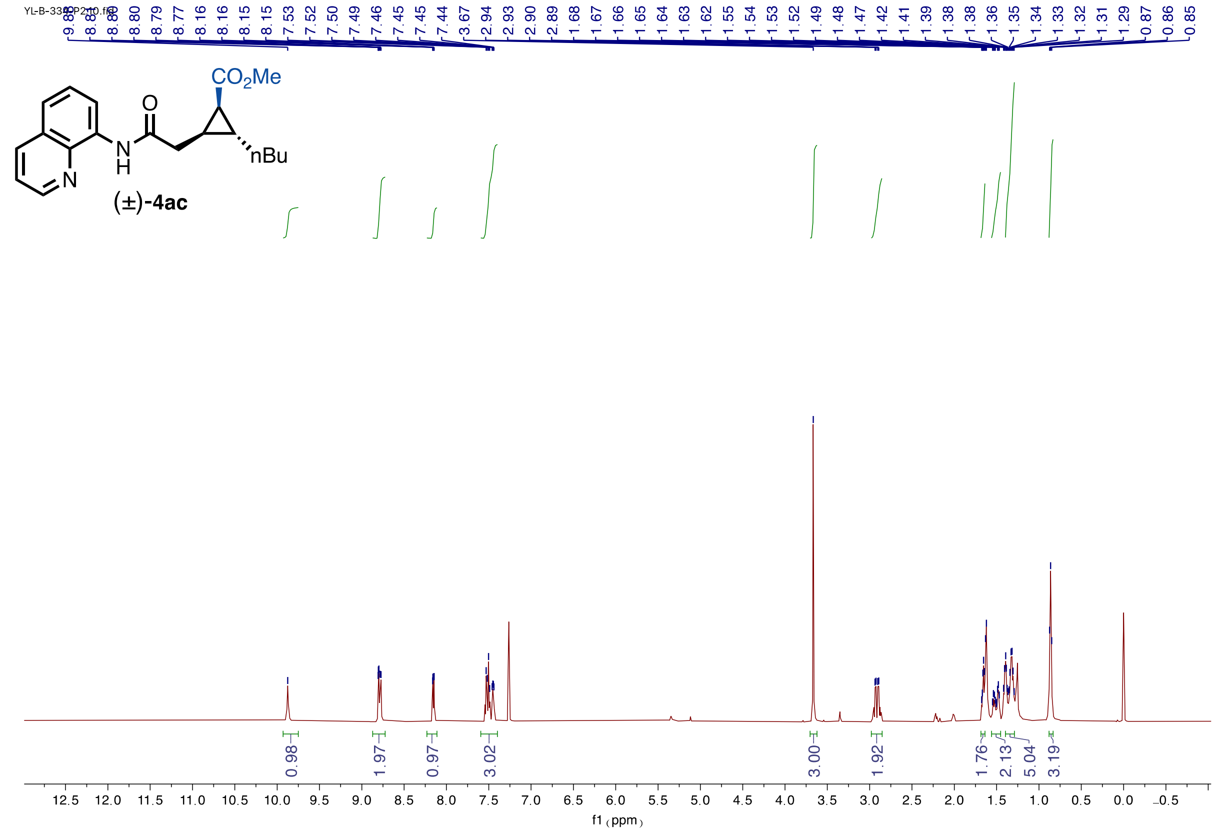

CO2Me
O
N
H
N
nBu
(±)-4ac
8.80
8.79
8.77
8.16
8.16
8.15
7.53
7.52
7.50
7.49
7.46
7.45
7.45
7.44
3.67
2.94
2.93
2.90
2.89
1.68
1.67
1.66
1.65
1.64
1.63
1.62
1.55
1.54
1.53
1.52
1.49
1.48
1.47
1.42
1.41
1.39
1.38
1.38
1.36
1.35
1.34
1.33
1.32
1.31
1.29
0.87
0.86
0.85
0.98
1.97
0.97
3.02
3.00
1.92
1.76
2.13
5.04
3.19
f1 (ppm)

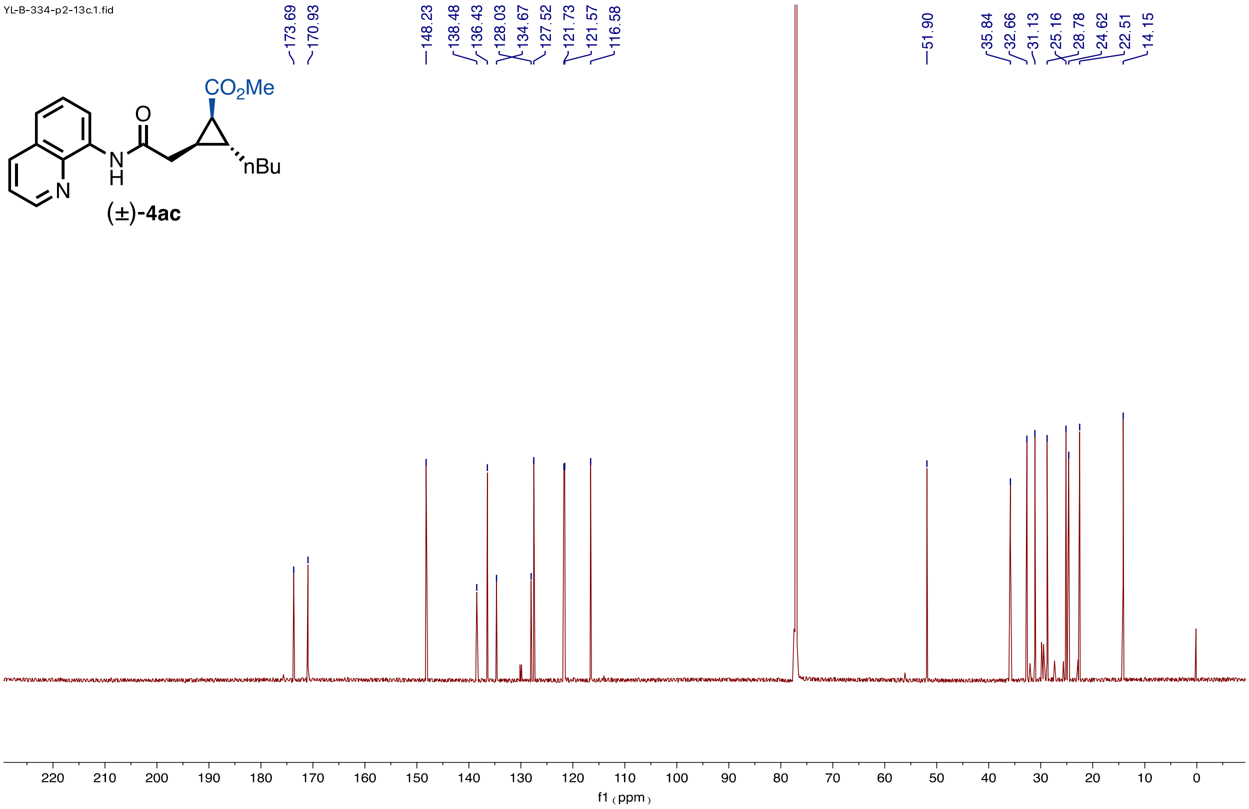

YL-B-334-p2-13c.1.fid
CO2Me
O
N
H
N
nBu
(±)-4ac
173.69
170.93
148.23
138.48
136.43
128.03
134.67
127.52
121.73
121.57
116.58
51.90
35.84
32.66
31.13
25.16
28.78
24.62
22.51
14.15
f1 (ppm)

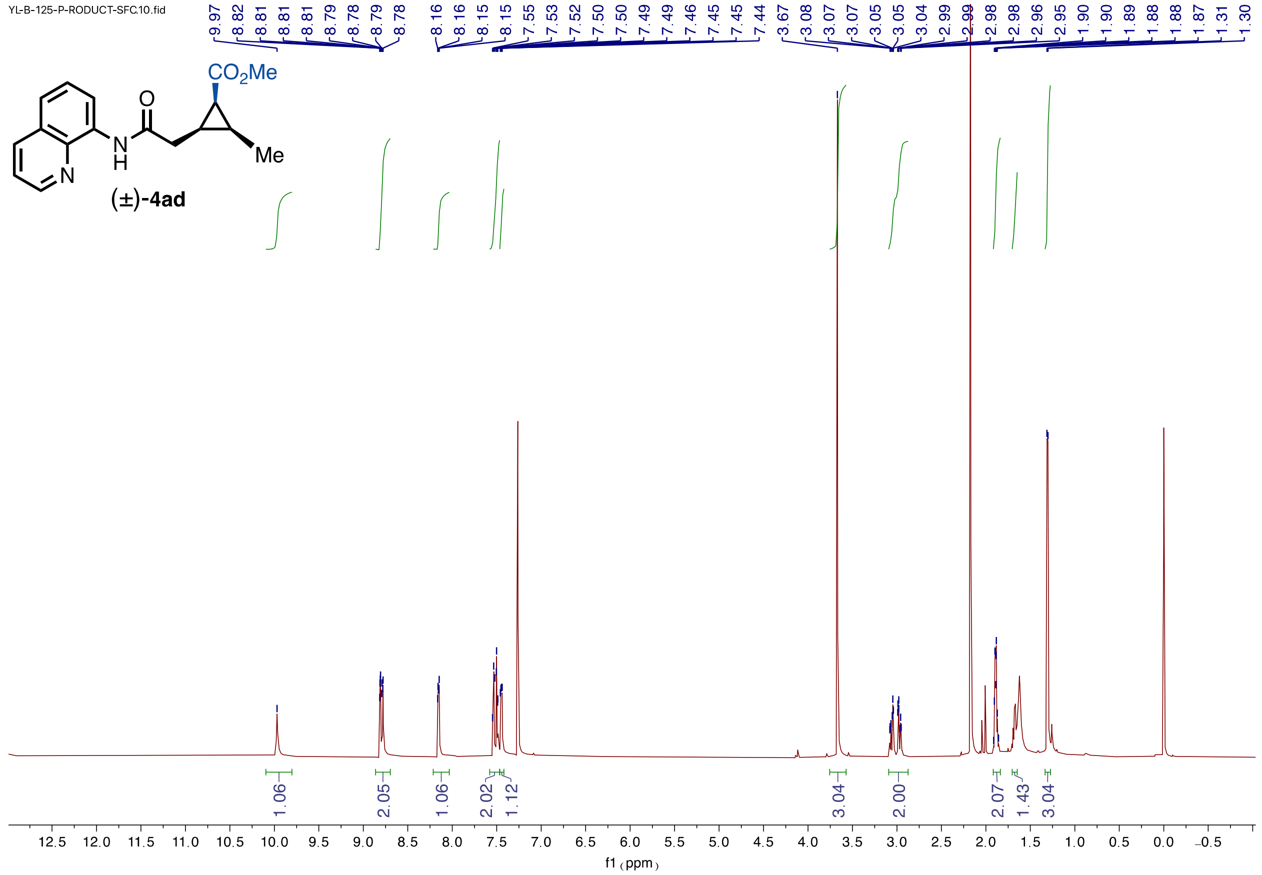
YL-B-125-P-RODUCT-SFC.10.fid
9.97 8.82 8.81 8.81 8.81 8.79 8.78 8.79 8.78 8.16 8.16 8.15 8.15 7.55 7.53 7.52 7.50 7.50 7.49 7.49 7.46 7.45 7.45 7.44 3.67 3.08 3.07 3.07 3.05 3.05 3.04 2.99 2.99 2.98 2.98 2.96 2.95 1.90 1.90 1.89 1.88 1.88 1.87 1.31 1.30
CO2Me
O
N
H
Me
N
(±)-4ad
1.06 2.05 1.06 2.02 1.12 3.04 2.00 2.07 1.43 3.04
f1 (ppm)

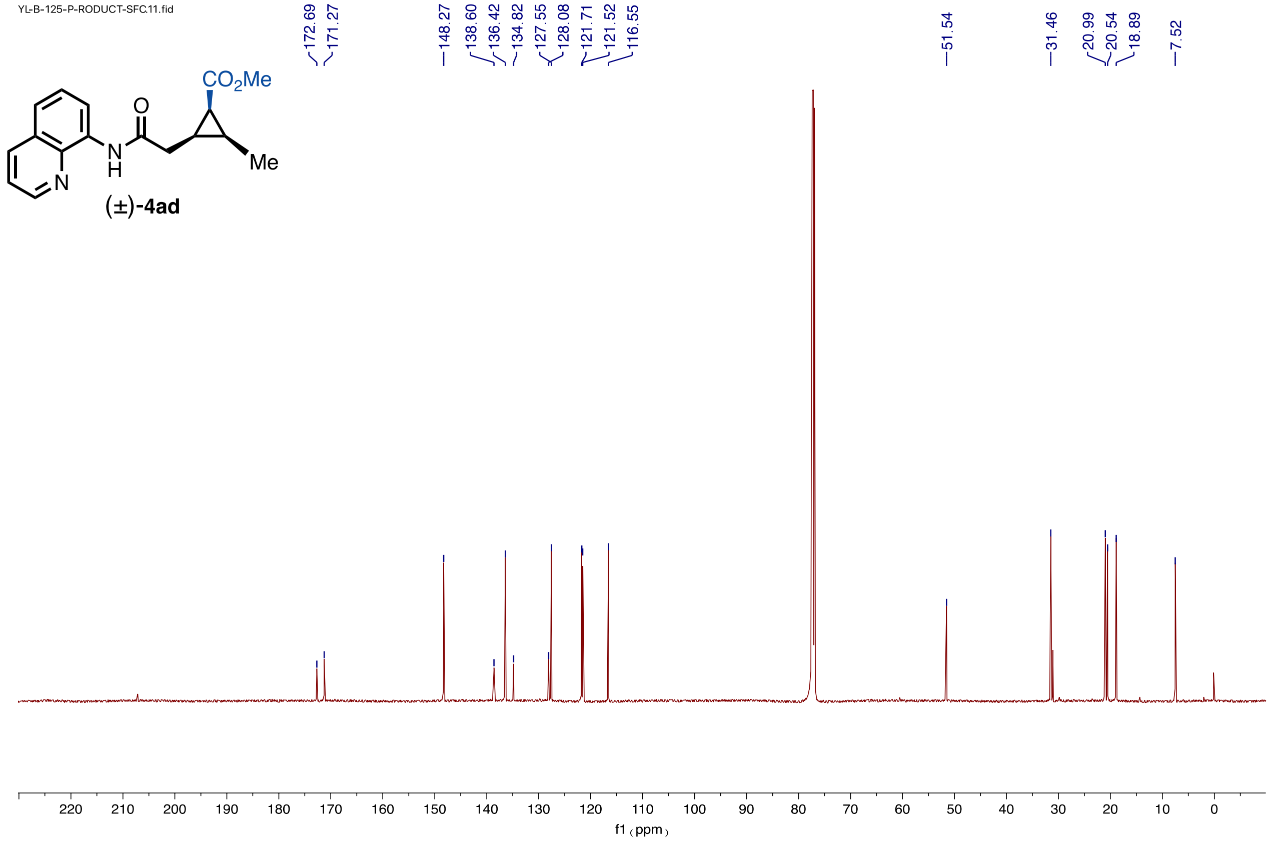
YL-B-125-P-RODUCT-SFC.11.fid
172.69 171.27 148.27 138.60 136.42 134.82 127.55 128.08 121.71 121.52 116.55 51.54 31.46 20.99 20.54 18.89 7.52
CO2Me
O
N
H
Me
N
(±)-4ad
f1 (ppm)

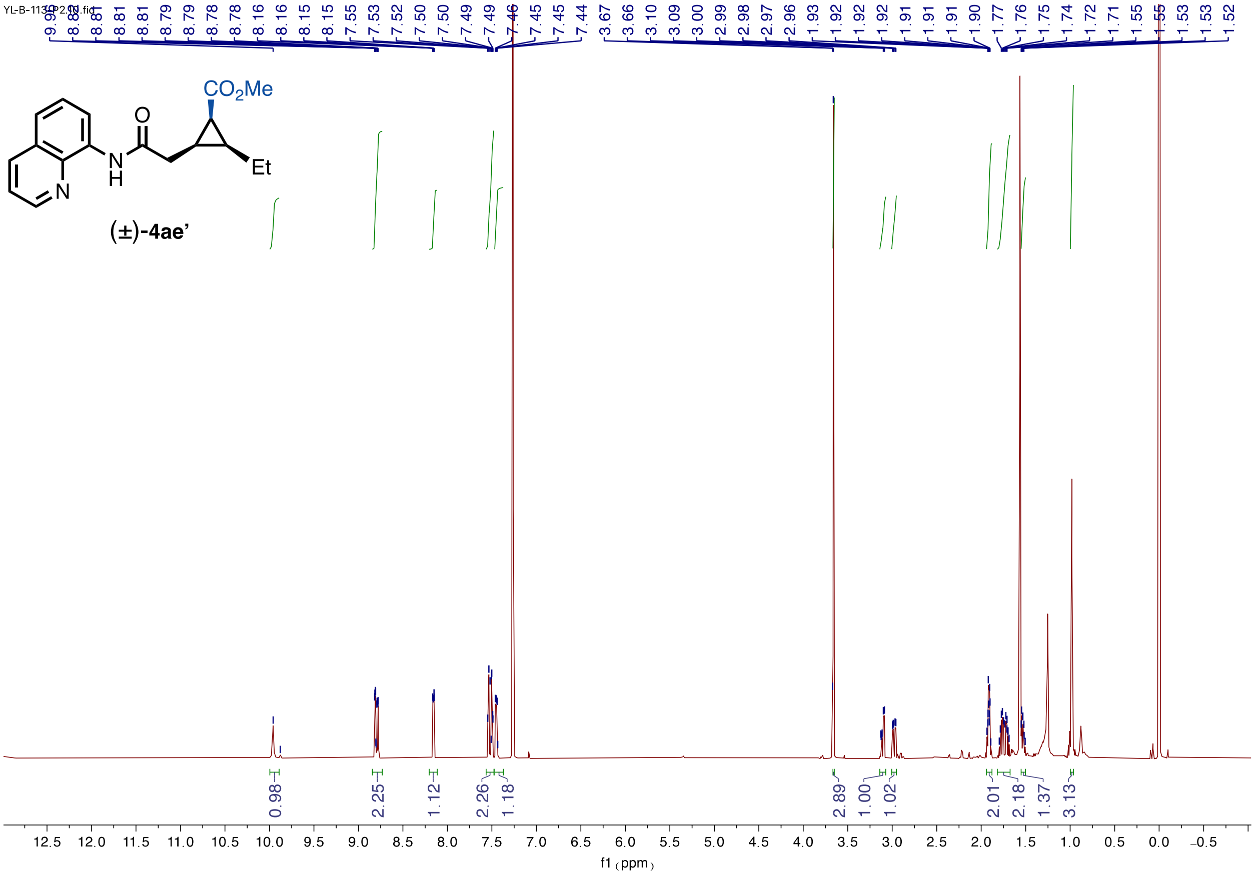
9.96 8.81 8.81 8.81 8.79 8.79 8.78 8.78 8.16 8.16 8.15 8.15 7.55 7.53 7.52 7.50 7.50 7.49 7.49 7.46 7.45 7.45 7.44 3.67 3.66 3.10 3.09 3.00 2.99 2.98 2.97 2.96 1.93 1.92 1.92 1.92 1.91 1.91 1.91 1.90 1.77 1.76 1.75 1.74 1.72 1.71 1.55 1.55 1.53 1.53 1.52
CO2Me
O
N
H
N
Et
(±)-4ae'
0.98 2.25 1.12 2.26 1.18 2.89 1.00 1.02 2.01 2.18 1.37 3.13
f1 (ppm)

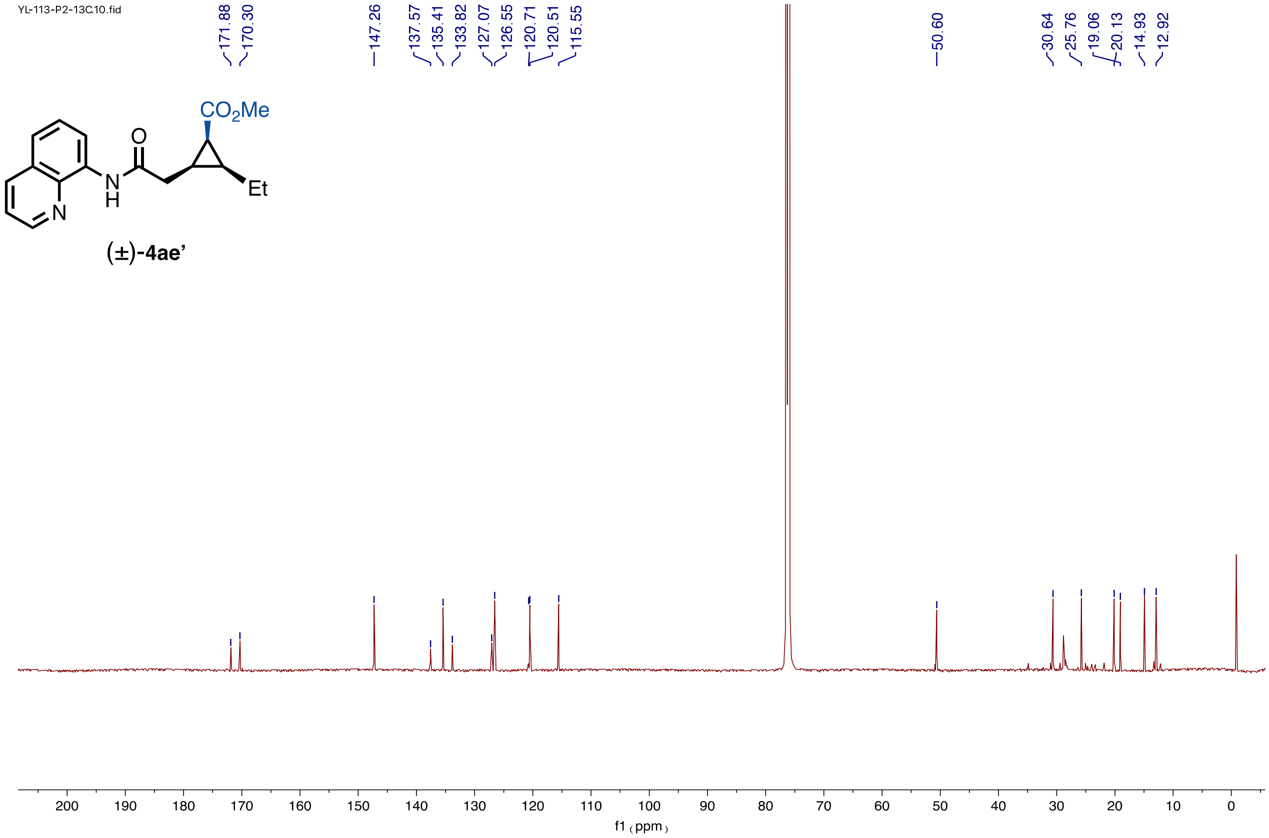
YL-113-P2-13C10.fid
171.88 170.30 147.26 137.57 135.41 133.82 127.07 126.55 120.71 120.51 115.55 50.60 30.64 25.76 19.06 20.13 14.93 12.92
CO2Me
O
N
H
N
Et
(±)-4ae'
f1 (ppm)

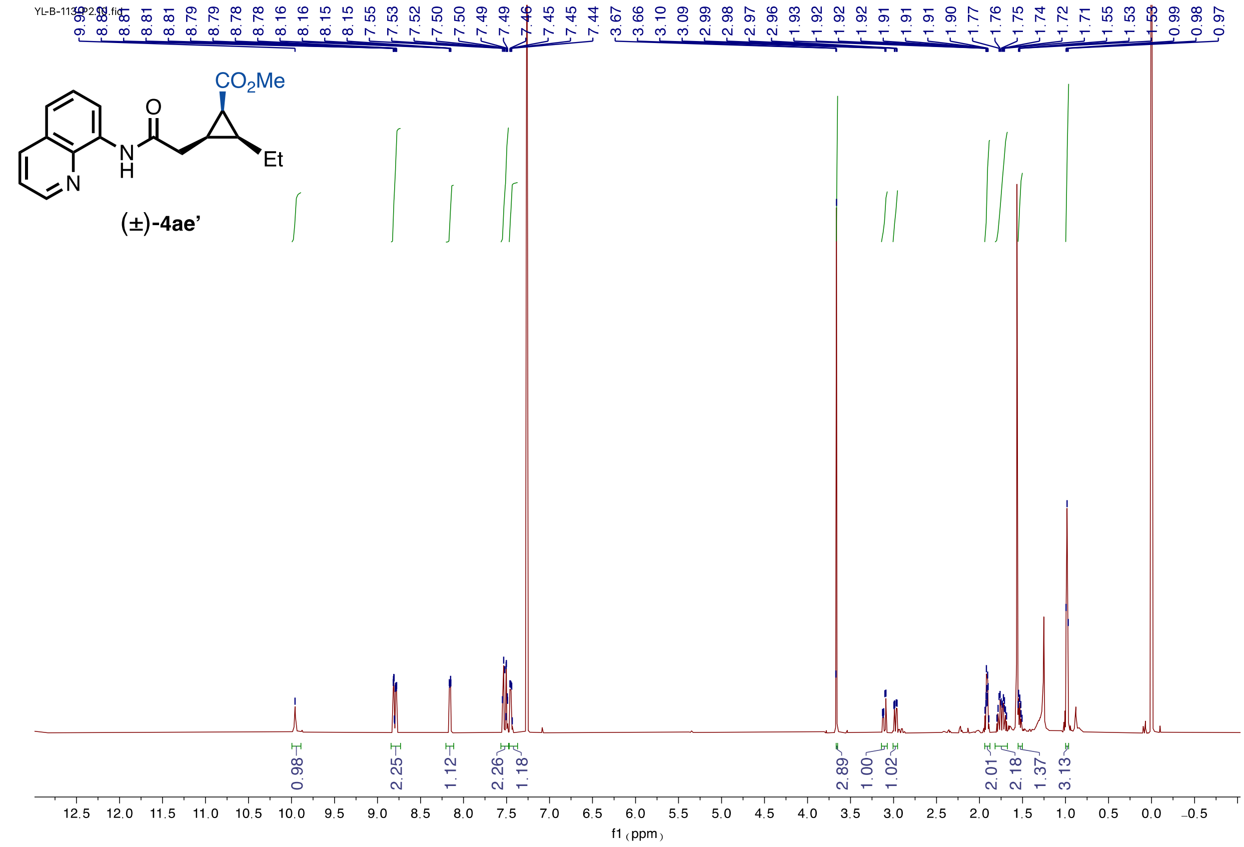
YL-B-113-P2.10.fid
CO2Me
O
N
H
N
Et
(±)-4ae'
9.97 8.82 8.81 8.81 8.79 8.79 8.78 8.78 8.16 8.15 8.15 7.55 7.53 7.52 7.50 7.50 7.49 7.49 7.46 7.45 7.45 7.44 3.67 3.66 3.10 3.09 2.99 2.98 2.97 2.96 1.93 1.92 1.92 1.92 1.91 1.91 1.91 1.90 1.77 1.76 1.75 1.74 1.72 1.71 1.55 1.53 1.53 0.99 0.98 0.97
0.98 2.25 1.12 2.26 1.18 2.89 1.00 1.02 2.01 2.18 1.37 3.13
12.5 12.0 11.5 11.0 10.5 10.0 9.5 9.0 8.5 8.0 7.5 7.0 6.5 6.0 5.5 5.0 4.5 4.0 3.5 3.0 2.5 2.0 1.5 1.0 0.5 0.0 -0.5
f1 (ppm)

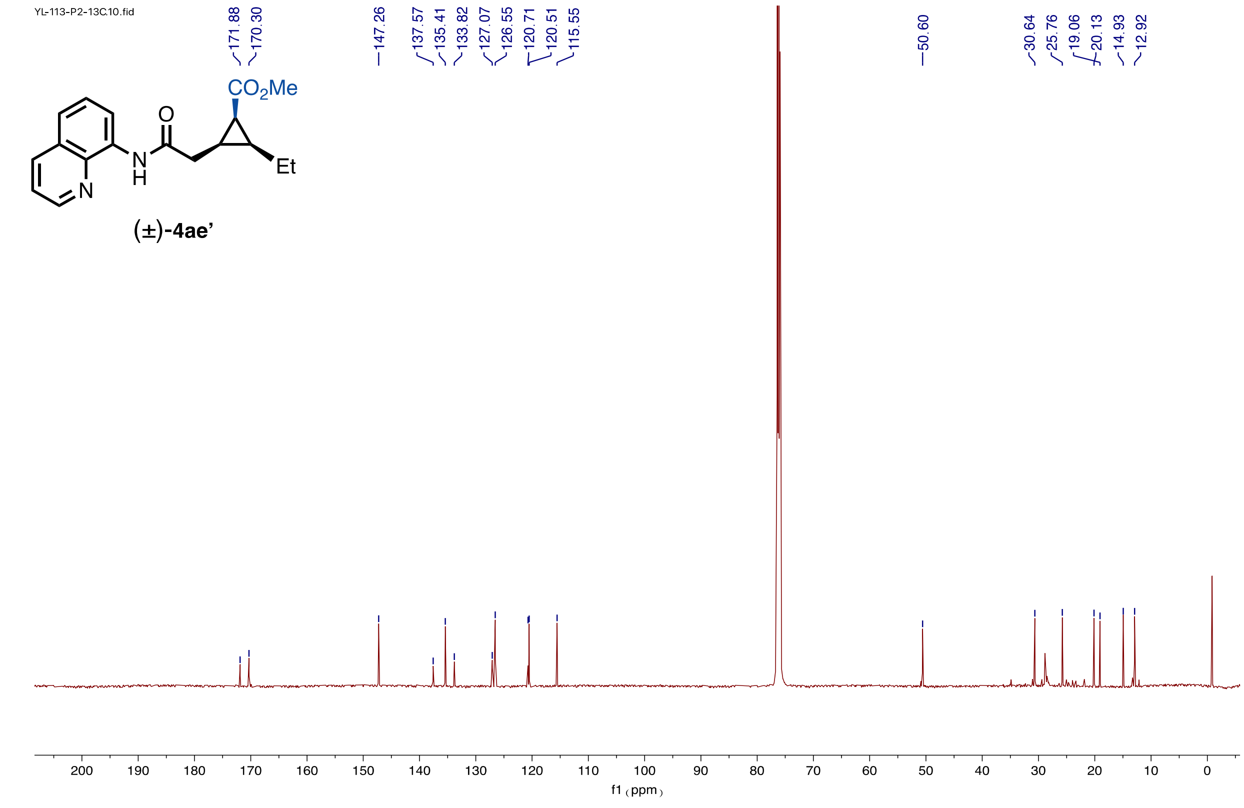
YL-113-P2-13C.10.fid
171.88 170.30 147.26 137.57 135.41 133.82 127.07 126.55 120.71 120.51 115.55 50.60 30.64 25.76 19.06 20.13 14.93 12.92
CO2Me
O
N
H
N
Et
(±)-4ae'
200 190 180 170 160 150 140 130 120 110 100 90 80 70 60 50 40 30 20 10 0
f1 (ppm)

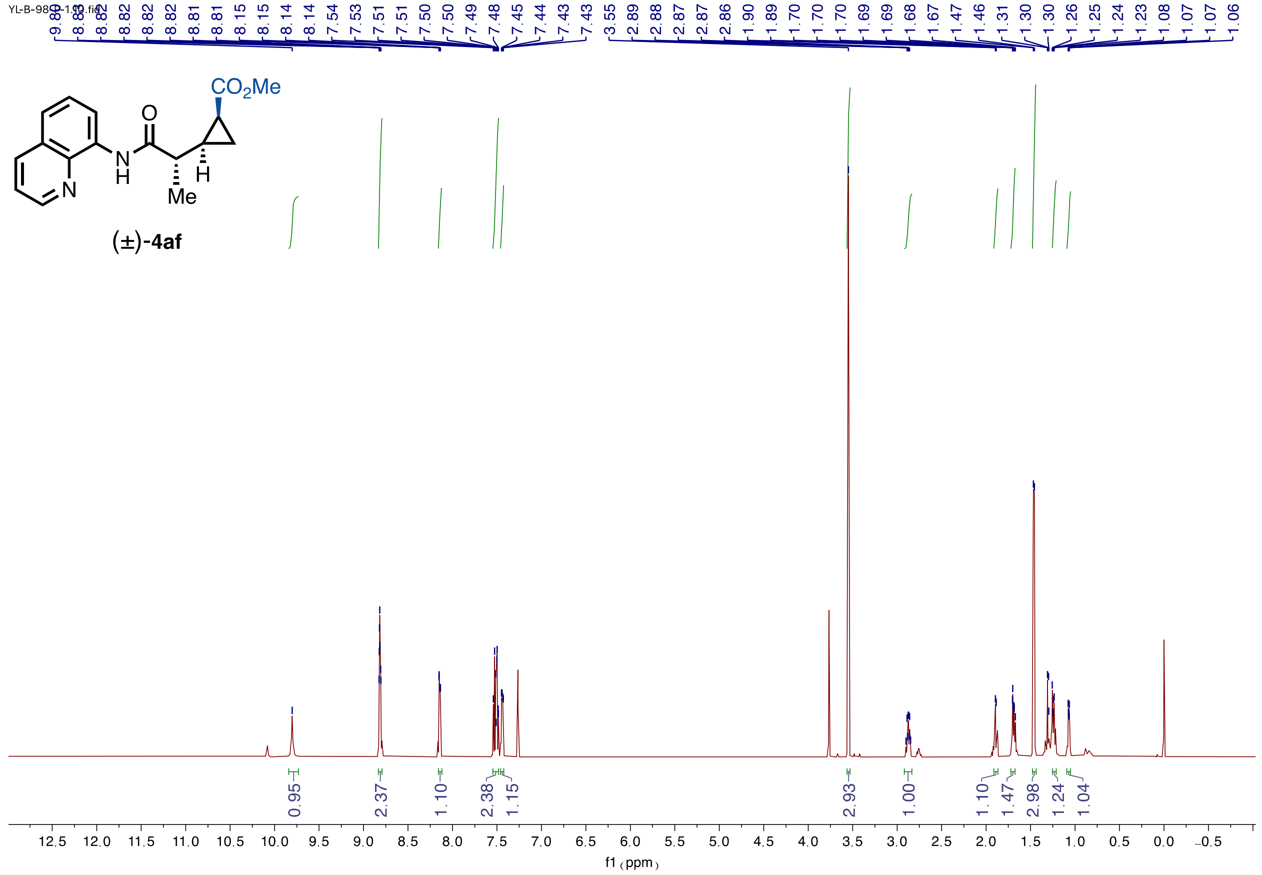
8.83
8.82
8.82
8.82
8.82
8.81
8.81
8.15
8.15
8.14
8.14
7.54
7.53
7.51
7.51
7.50
7.50
7.49
7.48
7.45
7.44
7.43
7.43
3.55
2.89
2.88
2.87
2.87
2.86
1.90
1.89
1.70
1.70
1.70
1.69
1.69
1.68
1.67
1.47
1.46
1.31
1.30
1.30
1.26
1.25
1.24
1.23
1.08
1.07
1.07
1.06
CO2Me
O
N
H
H
N
Me
(±)-4af
0.95
2.37
1.10
2.38
1.15
2.93
1.00
1.10
1.47
2.98
1.24
1.04
12.5 12.0 11.5 11.0 10.5 10.0 9.5 9.0 8.5 8.0 7.5 7.0 6.5 6.0 5.5 5.0 4.5 4.0 3.5 3.0 2.5 2.0 1.5 1.0 0.5 0.0 -0.5
f1 (ppm)

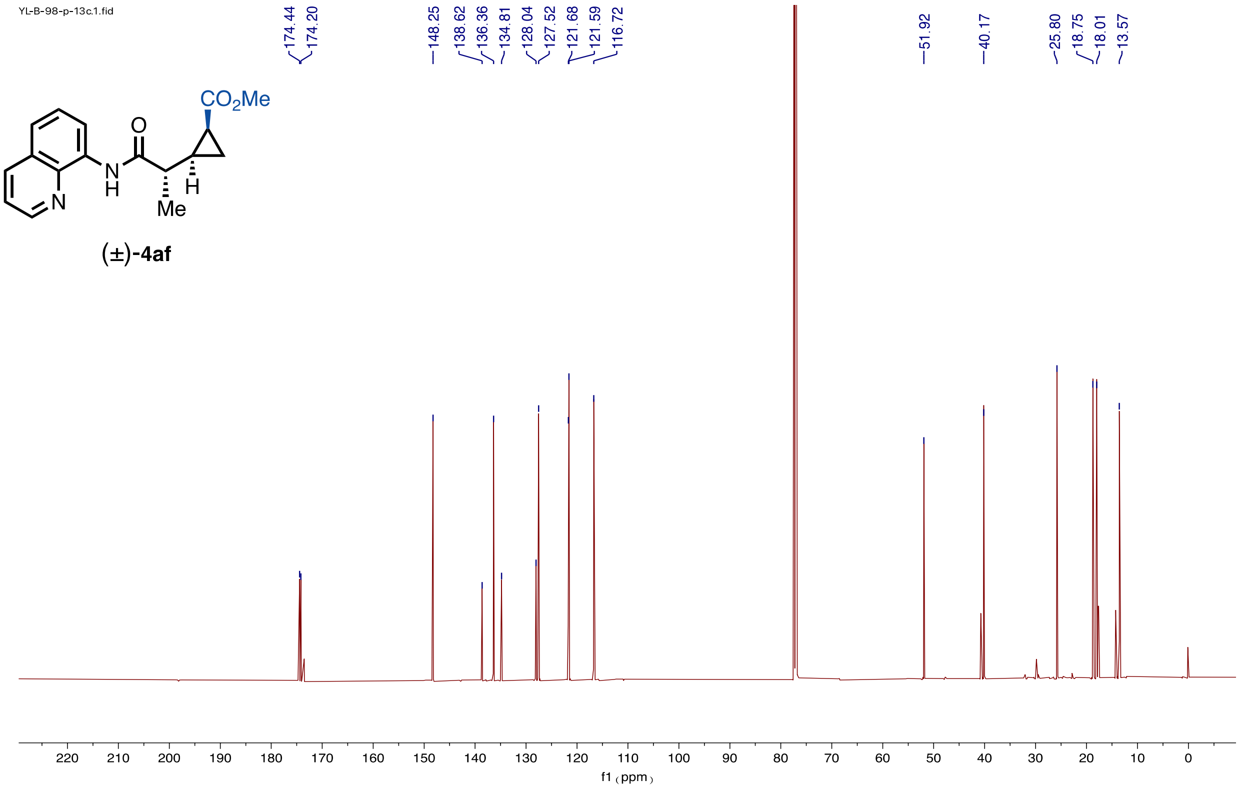
YL-B-98-p-13c.1.fid
174.44
174.20
148.25
138.62
136.36
134.81
128.04
127.52
121.68
121.59
116.72
51.92
40.17
25.80
18.75
18.01
13.57
CO2Me
O
N
H
H
N
Me
(±)-4af
220 210 200 190 180 170 160 150 140 130 120 110 100 90 80 70 60 50 40 30 20 10 0
f1 (ppm)

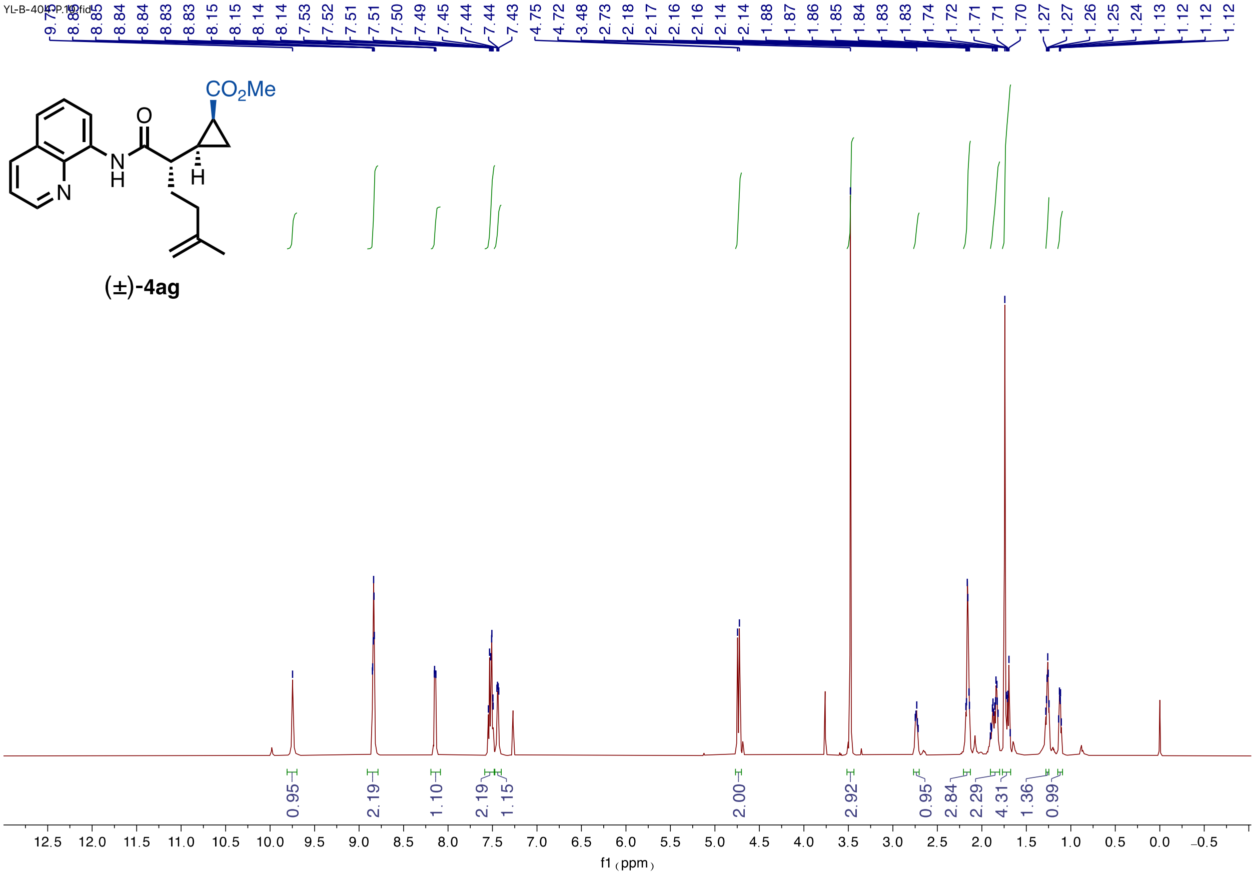

8.85
8.84
8.84
8.83
8.83
8.15
8.15
8.14
8.14
7.53
7.52
7.51
7.51
7.50
7.49
7.45
7.44
7.44
7.43
4.75
4.72
3.48
2.73
2.18
2.17
2.16
2.16
2.14
2.14
1.88
1.87
1.86
1.85
1.84
1.83
1.83
1.74
1.72
1.71
1.71
1.70
1.27
1.27
1.26
1.25
1.24
1.13
1.12
1.12
1.12
CO2Me
O
N
H
N
H
(±)-4ag
0.95
2.19
1.10
2.19
1.15
2.00
2.92
0.95
2.84
2.29
4.31
1.36
0.99
f1 (ppm)

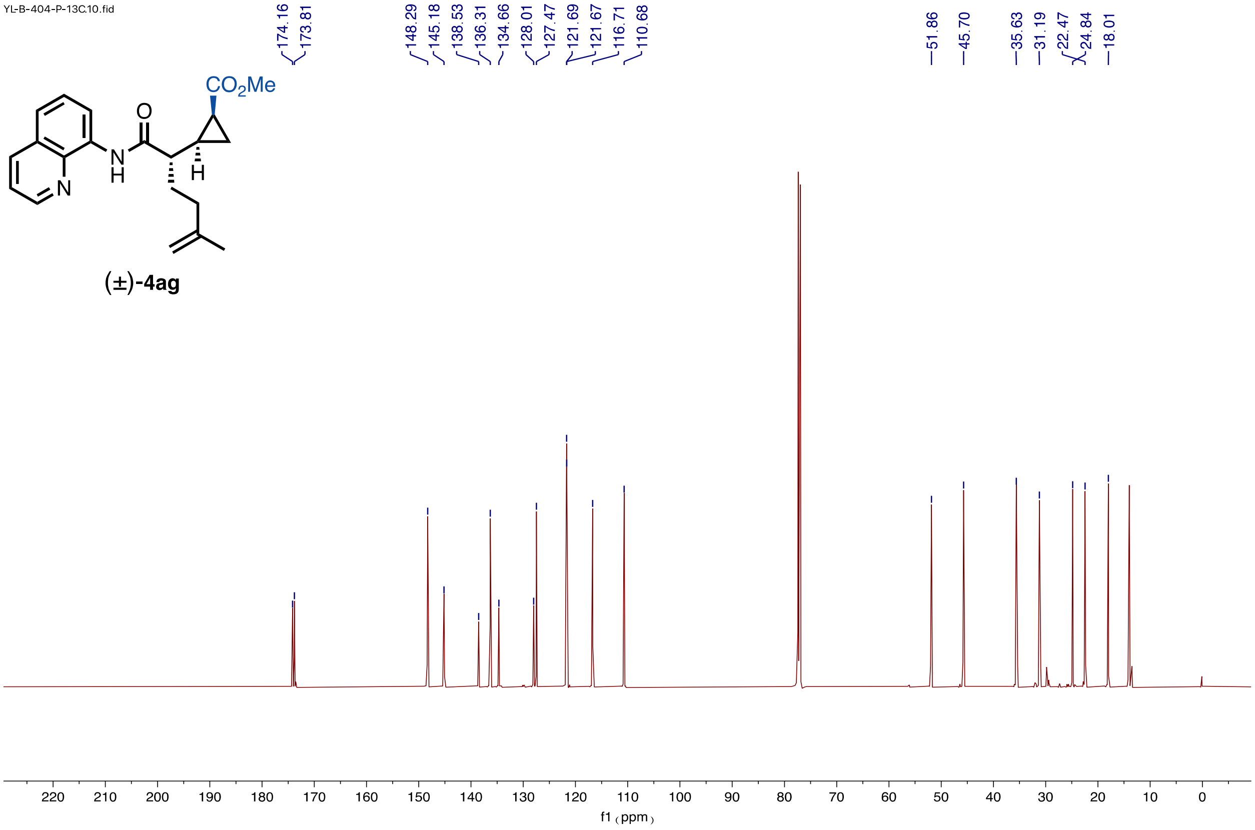

YL-B-404-P-13C.10.fid
174.16
173.81
148.29
145.18
138.53
136.31
134.66
128.01
127.47
121.69
121.67
116.71
110.68
51.86
45.70
35.63
31.19
22.47
24.84
18.01
CO2Me
O
N
H
N
H
(±)-4ag
f1 (ppm)

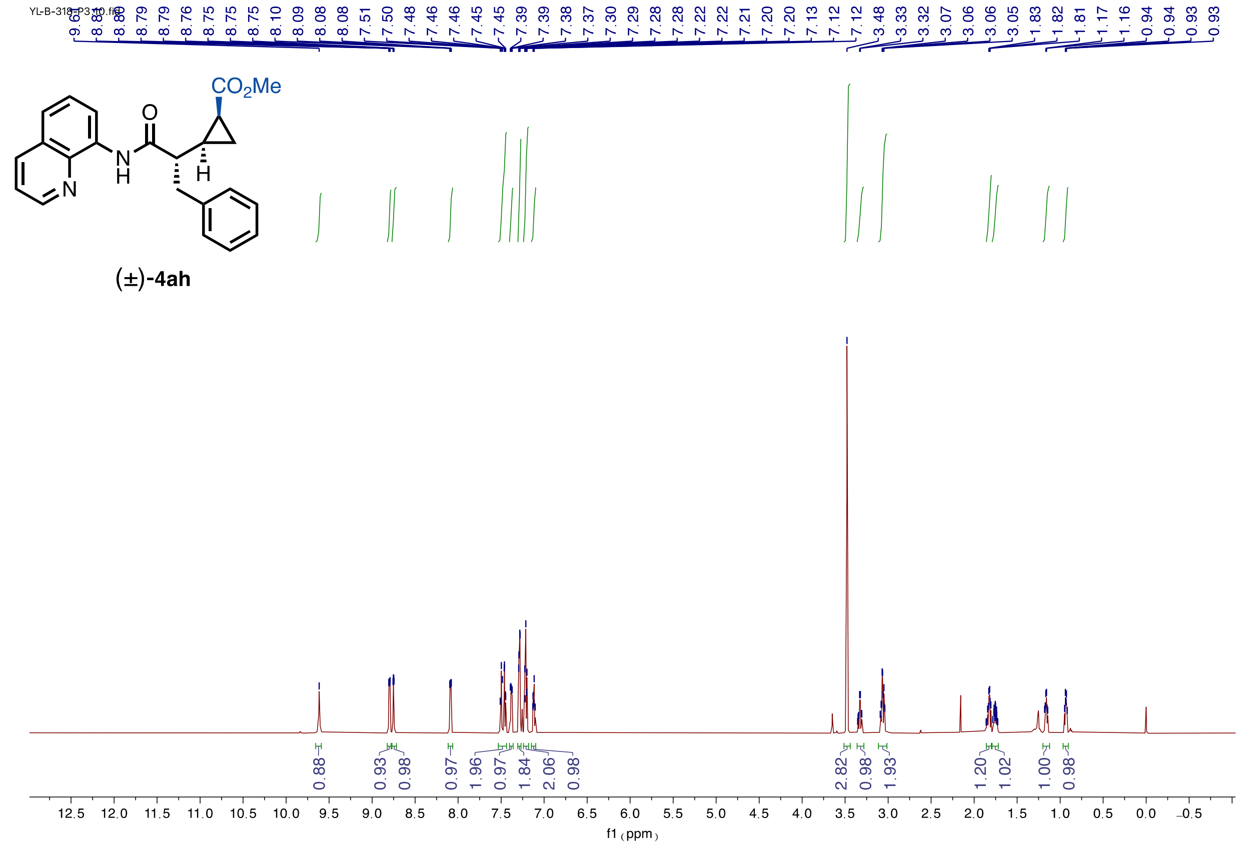

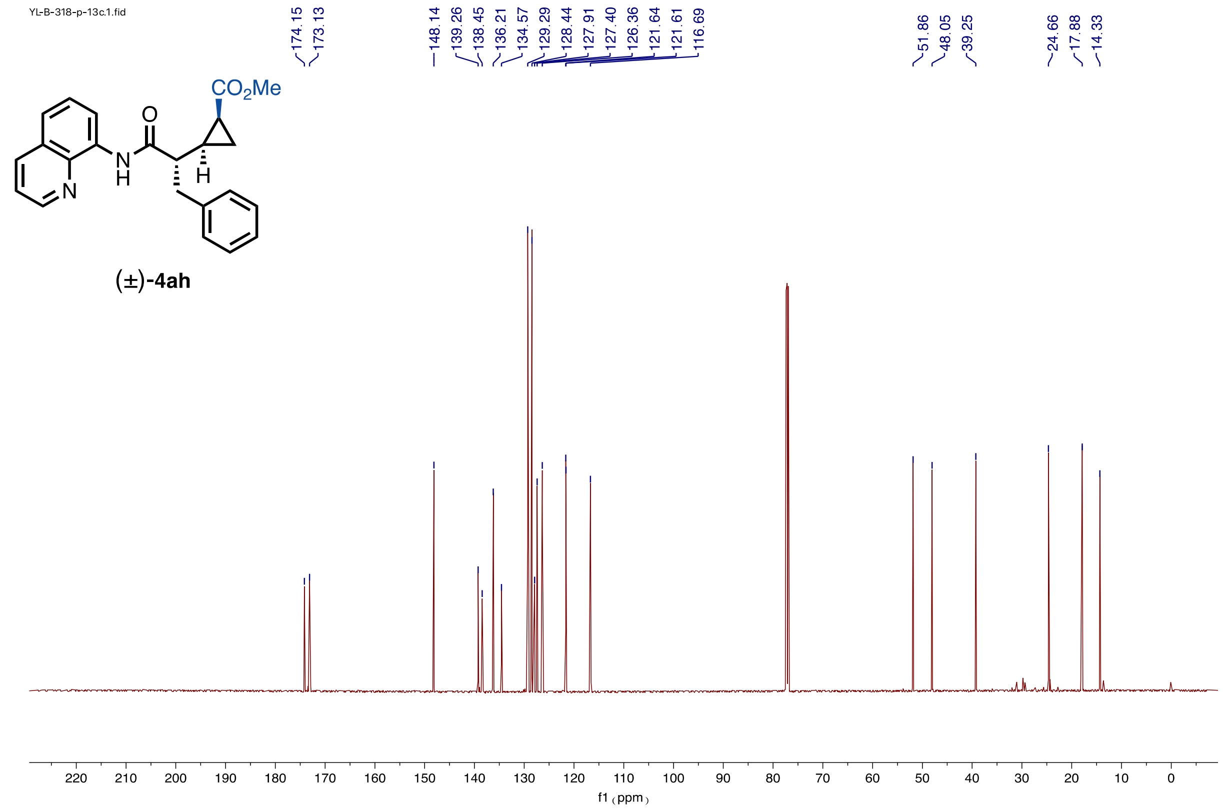

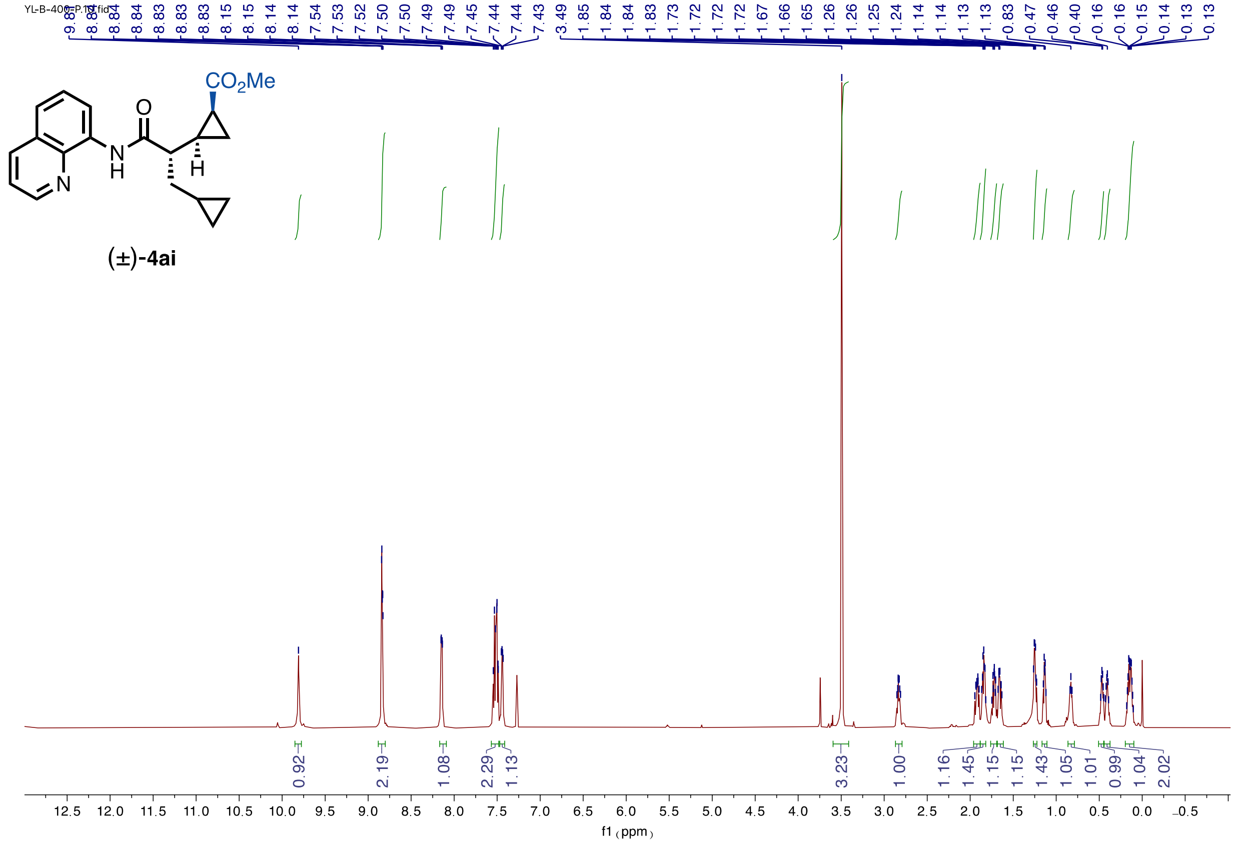
YL-B-400-P.10.fid
9.81 8.85 8.84 8.84 8.83 8.83 8.83 8.15 8.15 8.14 8.14 7.54 7.53 7.52 7.50 7.50 7.49 7.49 7.45 7.44 7.44 7.43 3.49 1.85 1.84 1.84 1.83 1.73 1.72 1.72 1.72 1.67 1.66 1.65 1.26 1.26 1.25 1.24 1.14 1.14 1.13 1.13 0.83 0.47 0.46 0.40 0.16 0.16 0.15 0.14 0.13 0.13
CO2Me
O
N
H
N
H
(±)-4ai
0.92 2.19 1.08 2.29 1.13 3.23 1.00 1.16 1.45 1.15 1.15 1.43 1.05 1.01 0.99 1.04 2.02
f1 (ppm)

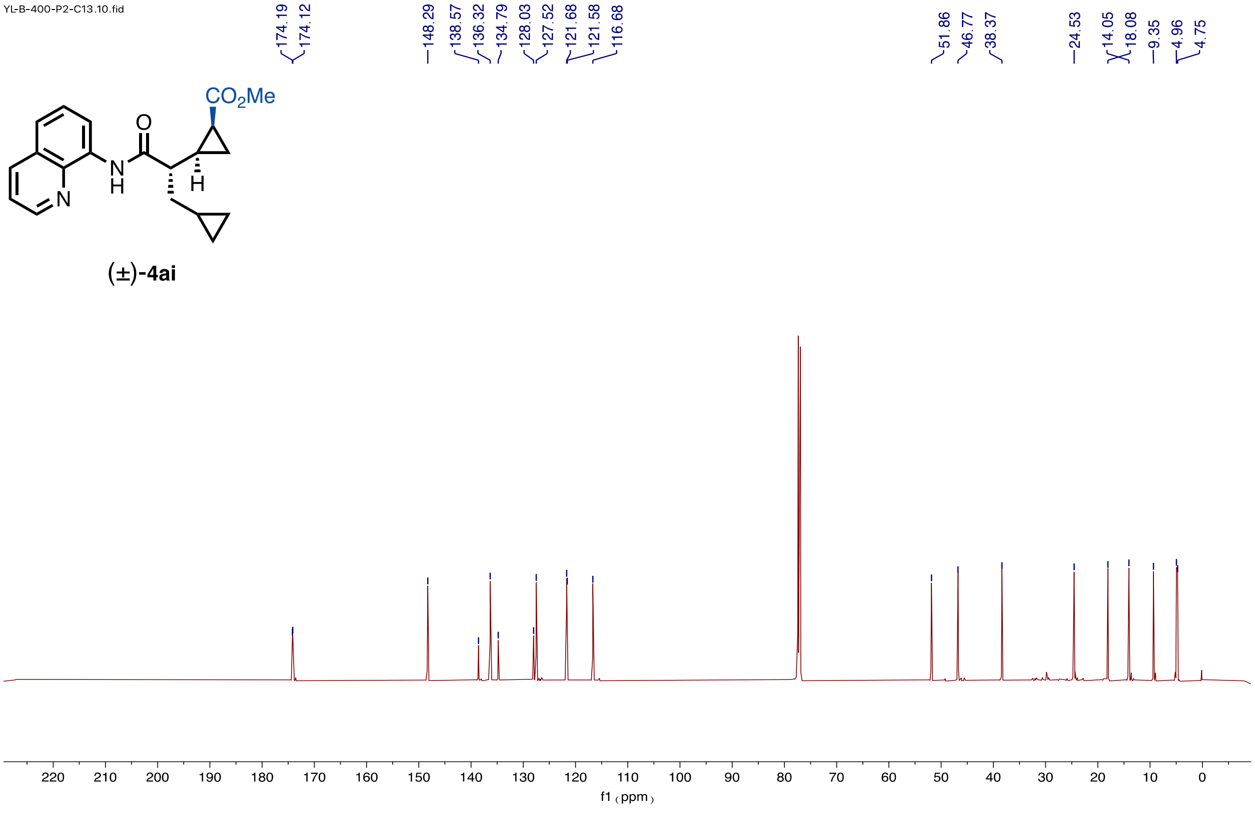
YL-B-400-P2-C13.10.fid
174.19 174.12 148.29 138.57 136.32 134.79 128.03 127.52 121.68 121.58 116.68 51.86 46.77 38.37 24.53 14.05 18.08 9.35 4.96 4.75
CO2Me
O
N
H
N
H
(±)-4ai
f1 (ppm)

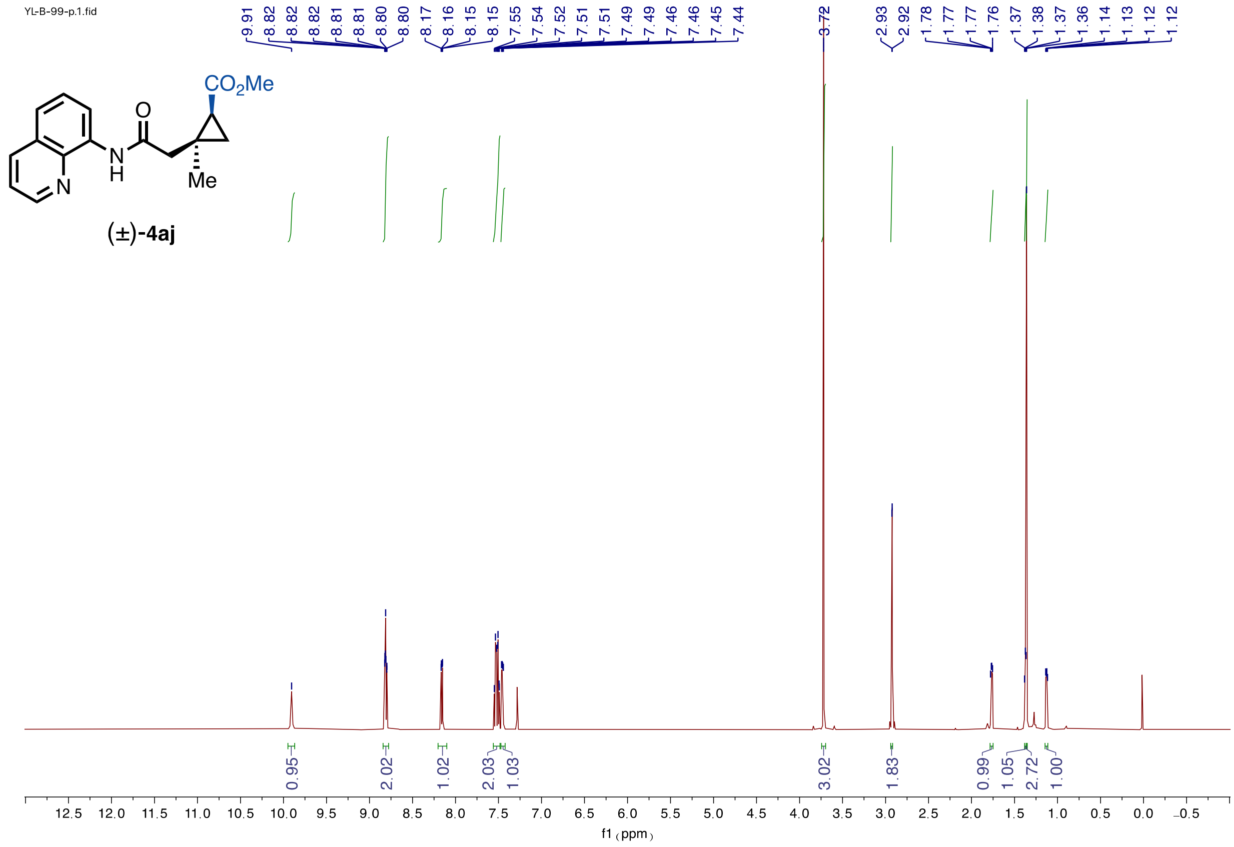
YL-B-99-p.1.fid
9.91 8.82 8.82 8.82 8.81 8.81 8.80 8.80 8.17 8.16 8.15 8.15 7.55 7.54 7.52 7.51 7.51 7.49 7.49 7.46 7.46 7.45 7.44
3.72
2.93 2.92 1.78 1.77 1.77 1.76 1.37 1.38 1.37 1.36 1.14 1.13 1.12 1.12
CO2Me
O
N
H
N
Me
(±)-4aj
0.95 2.02 1.02 2.03 1.03 3.02 1.83 0.99 1.05 2.72 1.00
12.5 12.0 11.5 11.0 10.5 10.0 9.5 9.0 8.5 8.0 7.5 7.0 6.5 6.0 5.5 5.0 4.5 4.0 3.5 3.0 2.5 2.0 1.5 1.0 0.5 0.0 -0.5
f1 (ppm)

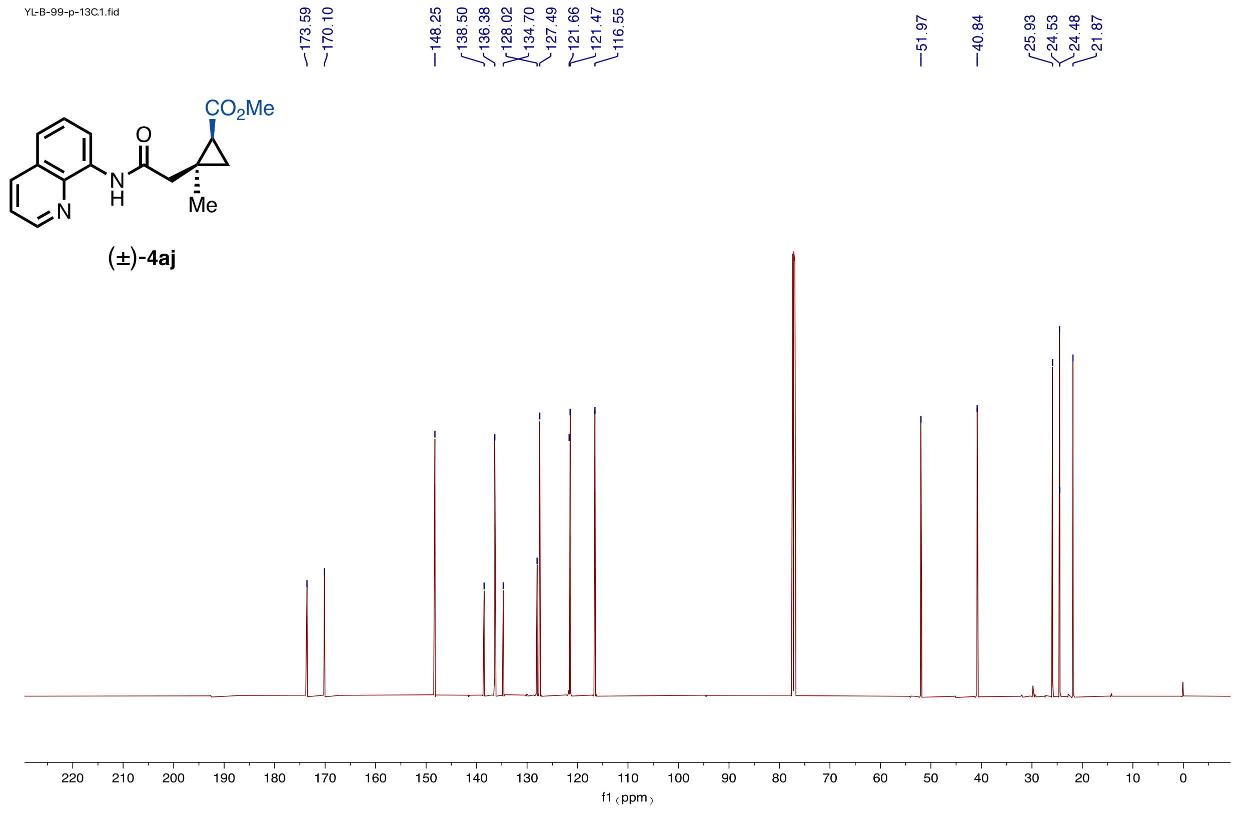
YL-B-99-p-13C1.fid
173.59 170.10
148.25 138.50 136.38 128.02 134.70 127.49 121.66 121.47 116.55
51.97
40.84
25.93 24.53 24.48 21.87
CO2Me
O
N
H
N
Me
(±)-4aj
220 210 200 190 180 170 160 150 140 130 120 110 100 90 80 70 60 50 40 30 20 10 0
f1 (ppm)

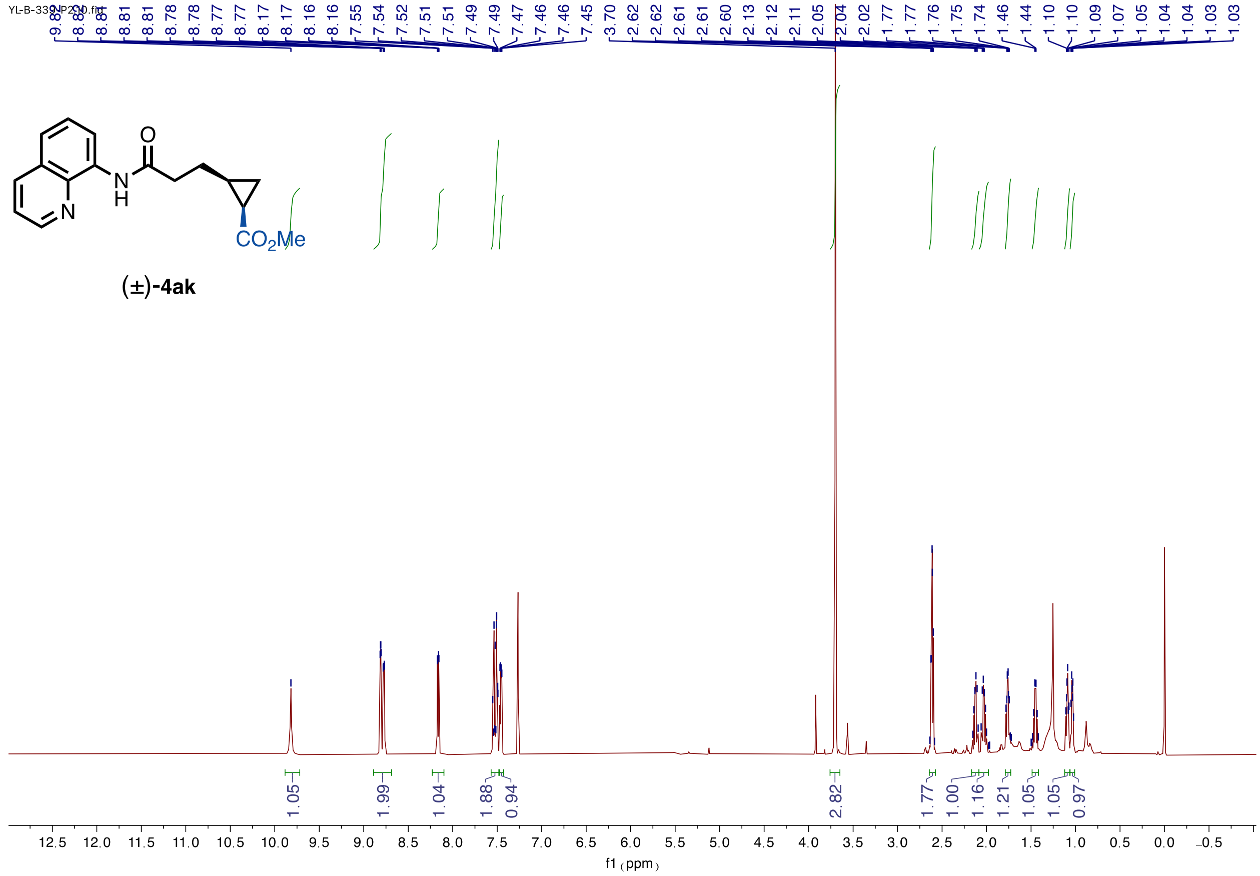
YL-B-339-P2-H.10.fid
CO2Me
(±)-4ak
1.05
1.99
1.04
1.88
0.94
2.82
1.77
1.00
1.16
1.21
1.05
1.05
0.97
f1 (ppm)

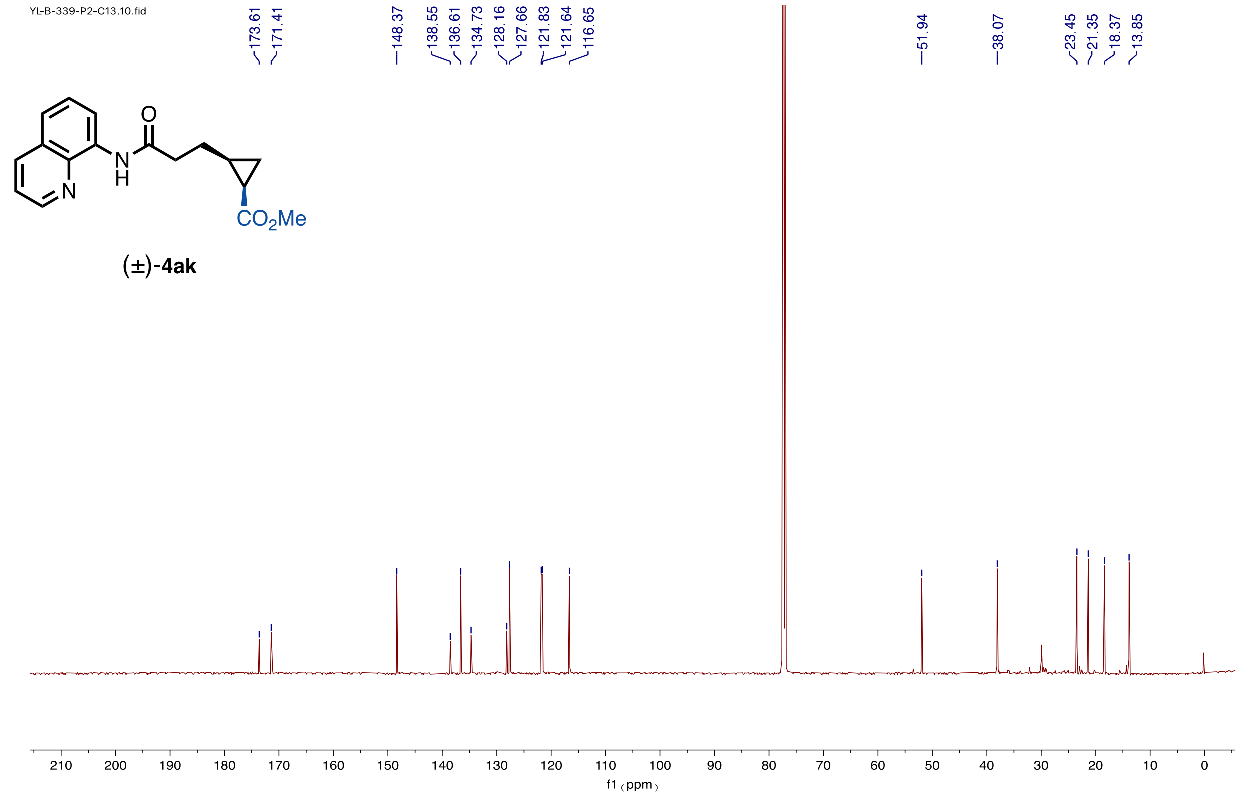
YL-B-339-P2-C13.10.fid
173.61
171.41
148.37
138.55
136.61
134.73
128.16
127.66
121.83
121.64
116.65
51.94
38.07
23.45
21.35
18.37
13.85
CO2Me
(±)-4ak
f1 (ppm)

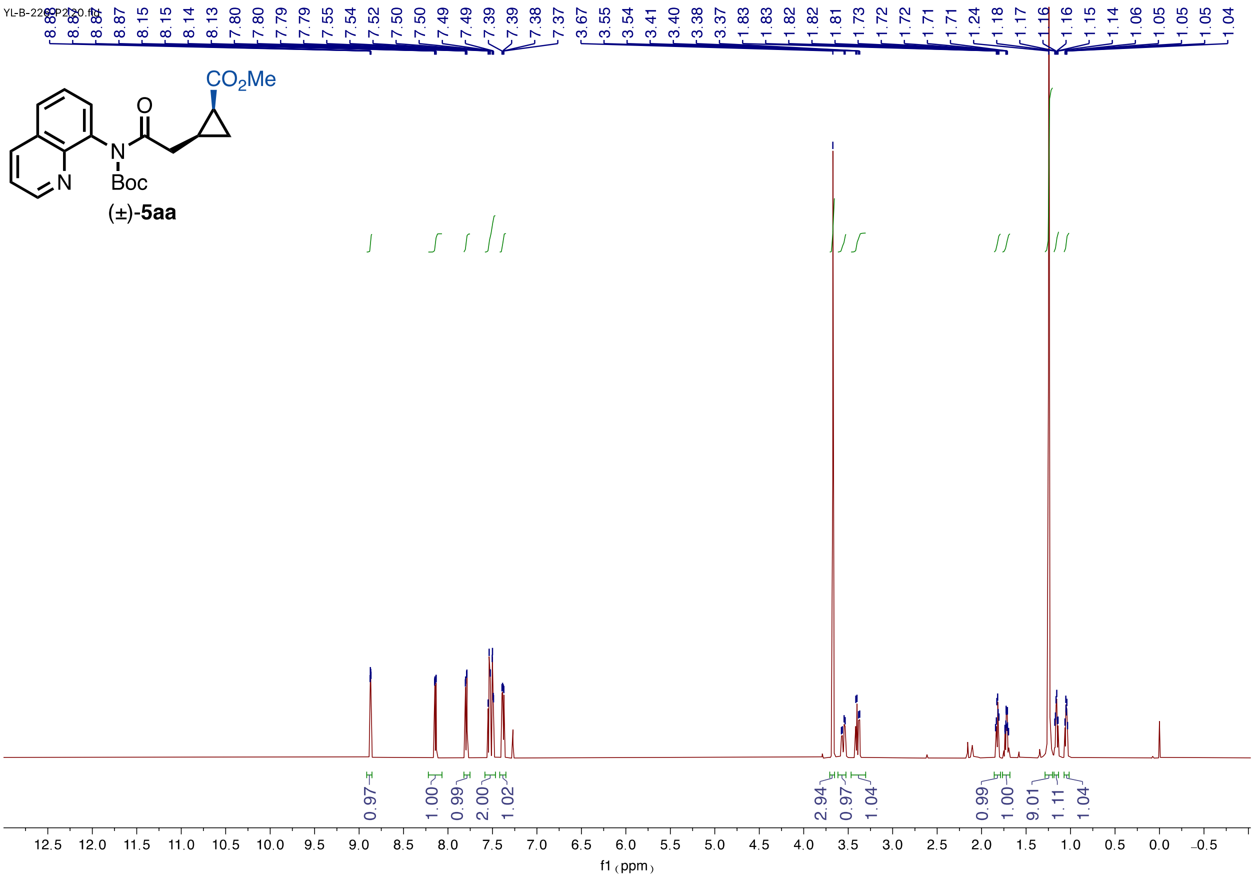
YL-B-226-P2.20.fid
8.87
8.87
8.15
8.15
8.14
8.13
7.80
7.80
7.79
7.79
7.55
7.54
7.52
7.50
7.50
7.49
7.49
7.39
7.39
7.38
7.37
3.67
3.55
3.54
3.41
3.40
3.38
3.37
1.83
1.83
1.82
1.82
1.81
1.73
1.72
1.72
1.71
1.71
1.24
1.18
1.17
1.16
1.16
1.15
1.14
1.06
1.05
1.05
1.05
1.04
CO2Me
O
N
N
Boc
(±)-5aa
0.97
1.00
0.99
2.00
1.02
2.94
0.97
1.04
0.99
1.00
9.01
1.11
1.04
12.5 12.0 11.5 11.0 10.5 10.0 9.5 9.0 8.5 8.0 7.5 7.0 6.5 6.0 5.5 5.0 4.5 4.0 3.5 3.0 2.5 2.0 1.5 1.0 0.5 0.0 -0.5
f1 (ppm)

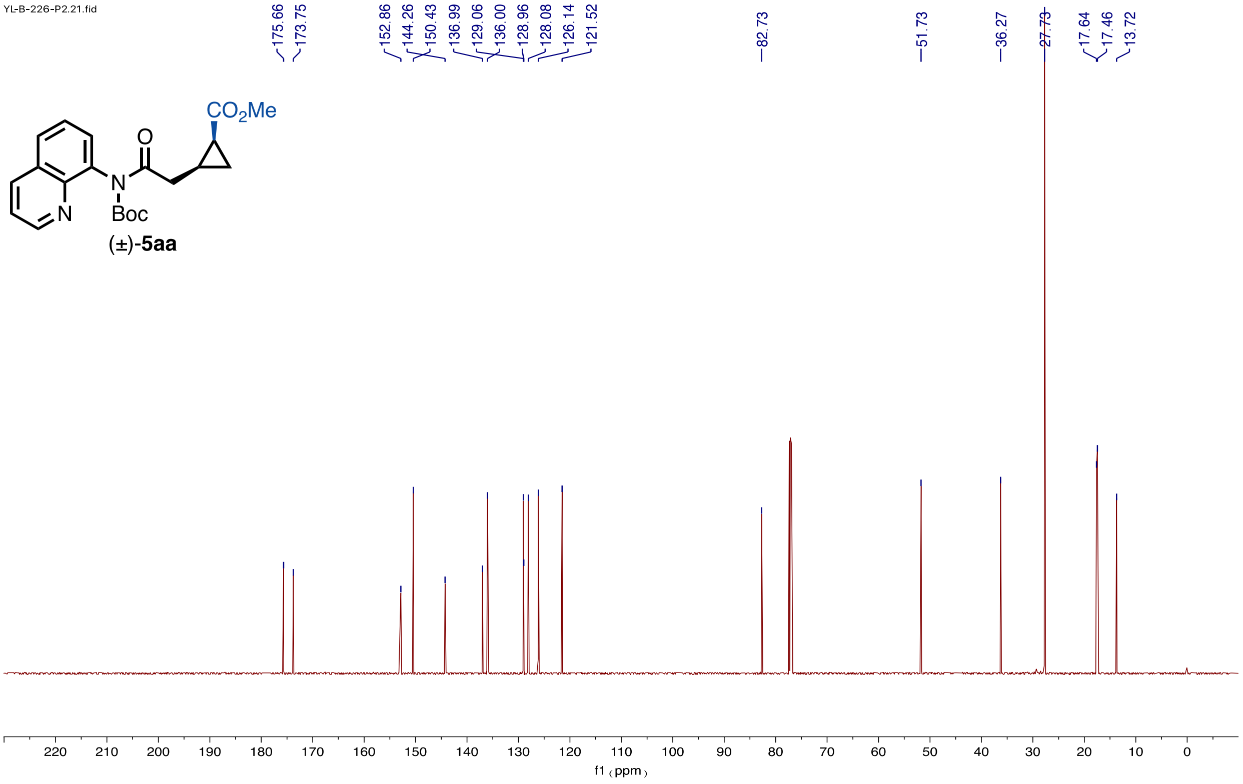
YL-B-226-P2.21.fid
175.66
173.75
152.86
144.26
150.43
136.99
129.06
136.00
128.96
128.08
126.14
121.52
82.73
51.73
36.27
27.73
17.64
17.46
13.72
CO2Me
O
N
N
Boc
(±)-5aa
220 210 200 190 180 170 160 150 140 130 120 110 100 90 80 70 60 50 40 30 20 10 0
f1 (ppm)

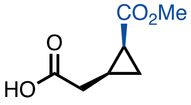

(±)-**6aa**

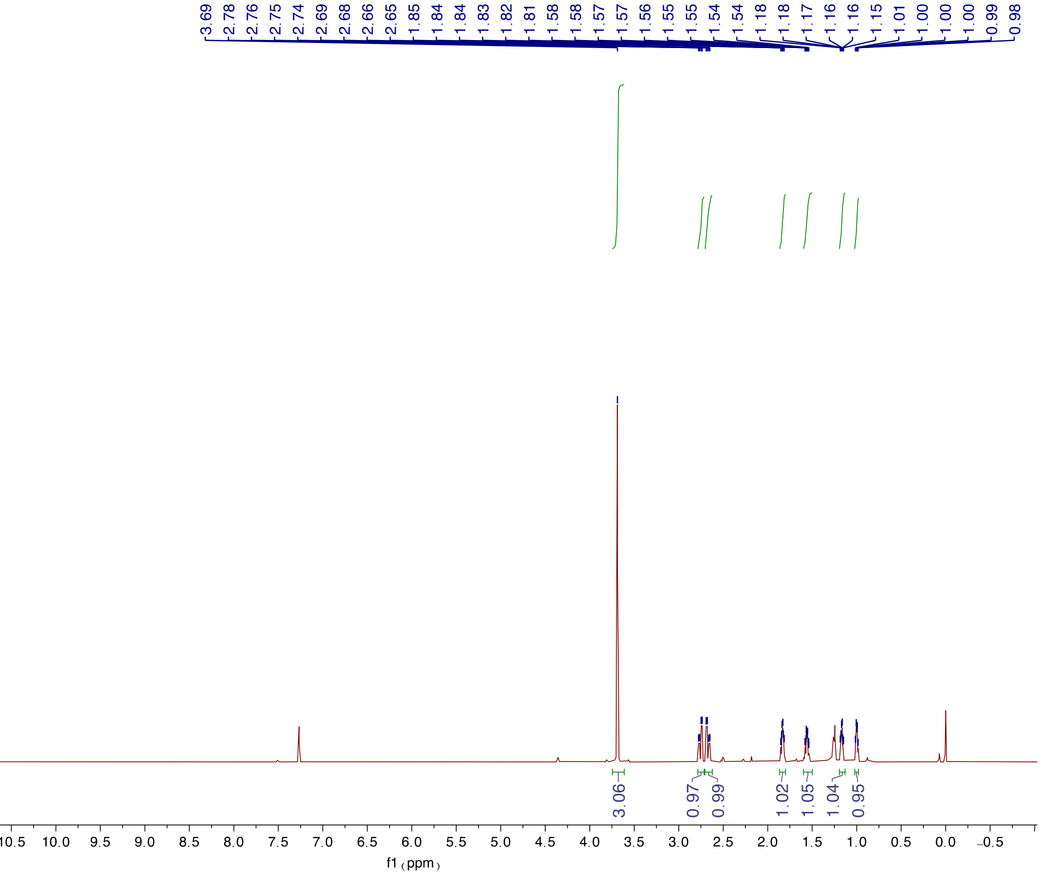

YL-B-227-P-600-1H.10.fid
3.69 2.78 2.76 2.75 2.74 2.69 2.68 2.66 2.65 1.85 1.84 1.84 1.83 1.82 1.81 1.58 1.58 1.57 1.57 1.56 1.55 1.55 1.54 1.54 1.18 1.17 1.16 1.16 1.15 1.01 1.00 1.00 0.99 0.98
3.06 0.97 0.99 1.02 1.05 1.04 0.95
12.5 12.0 11.5 11.0 10.5 10.0 9.5 9.0 8.5 8.0 7.5 7.0 6.5 6.0 5.5 5.0 4.5 4.0 3.5 3.0 2.5 2.0 1.5 1.0 0.5 0.0 -0.5
f1 (ppm)

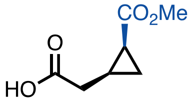

(±)-**6aa**

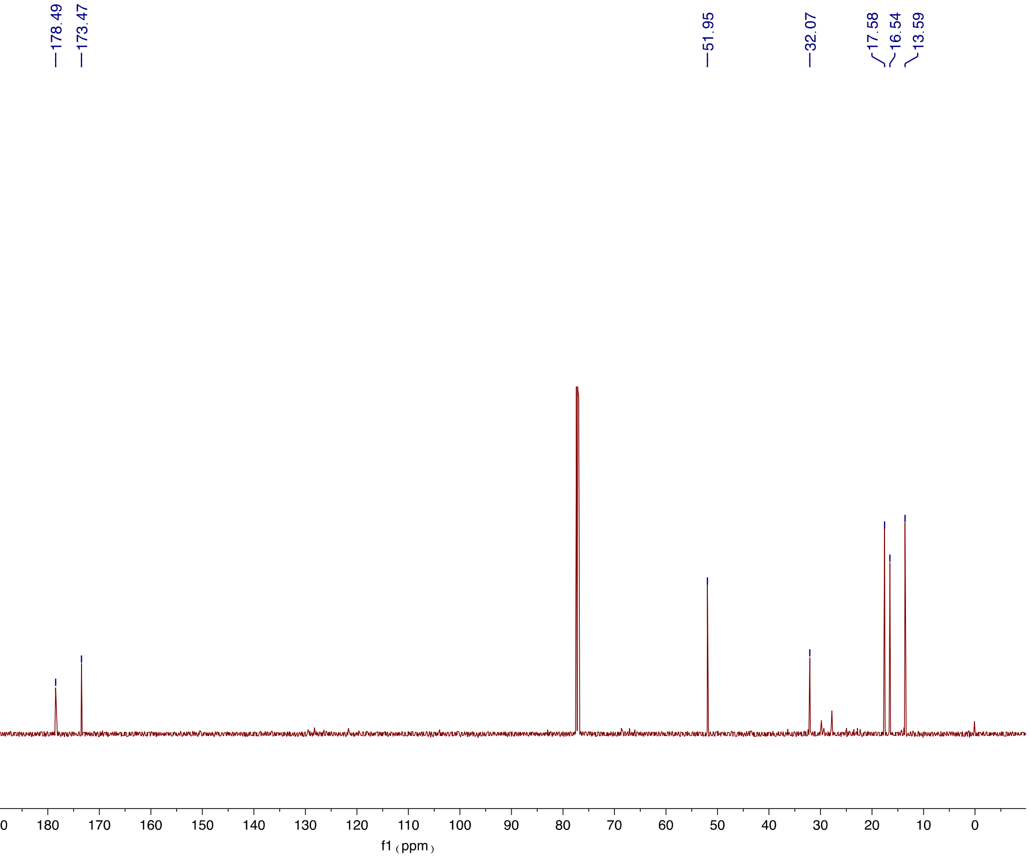

YL-B-227-P-600-13C.12.fid
178.49 173.47 51.95 32.07 17.58 16.54 13.59
220 210 200 190 180 170 160 150 140 130 120 110 100 90 80 70 60 50 40 30 20 10 0
f1 (ppm)

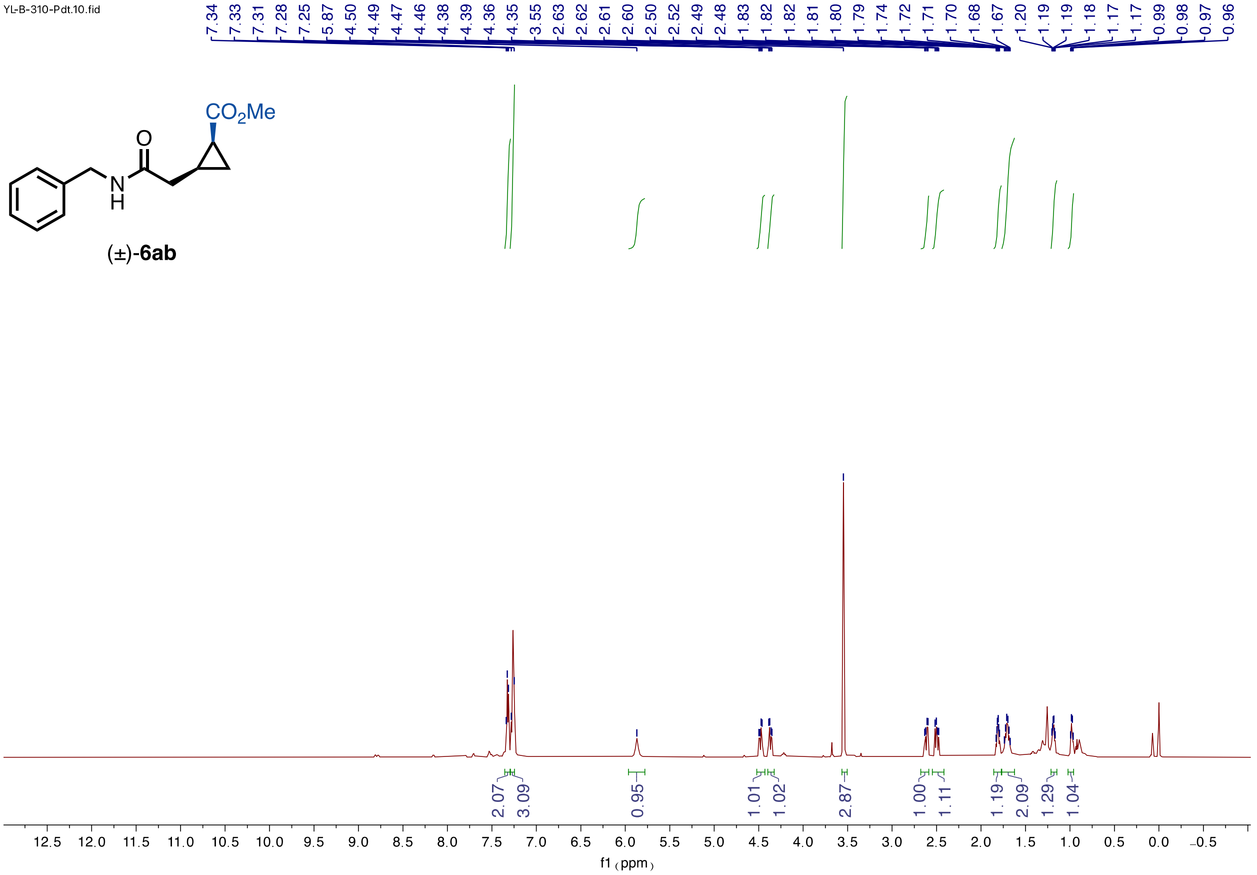
YL-B-310-Pdt.10.fid
7.34 7.33 7.31 7.28 7.25 5.87 4.50 4.49 4.47 4.46 4.38 4.39 4.36 4.35 3.55 2.63 2.62 2.61 2.60 2.50 2.52 2.49 2.48 1.83 1.82 1.82 1.81 1.80 1.79 1.74 1.72 1.71 1.70 1.68 1.67 1.20 1.19 1.19 1.18 1.17 1.17 0.99 0.98 0.97 0.96
CO_2Me
N
H
O
(±)-6ab
2.07 3.09 0.95 1.01 1.02 2.87 1.00 1.11 1.19 2.09 1.29 1.04
12.5 12.0 11.5 11.0 10.5 10.0 9.5 9.0 8.5 8.0 7.5 7.0 6.5 6.0 5.5 5.0 4.5 4.0 3.5 3.0 2.5 2.0 1.5 1.0 0.5 0.0 -0.5
f1 (ppm)

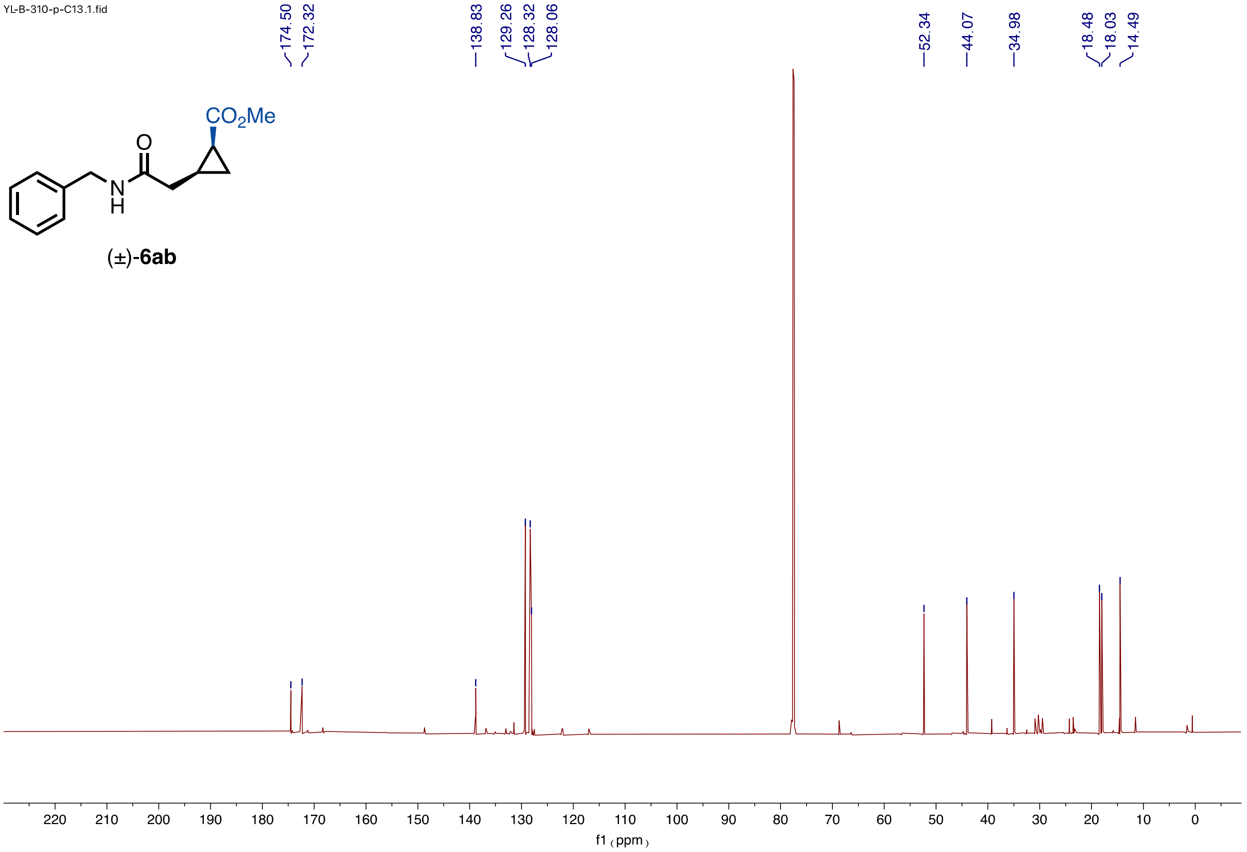
YL-B-310-p-C13.1.fid
174.50 172.32 138.83 129.26 128.32 128.06 52.34 44.07 34.98 18.48 18.03 14.49
CO_2Me
N
H
O
(±)-6ab
220 210 200 190 180 170 160 150 140 130 120 110 100 90 80 70 60 50 40 30 20 10 0
f1 (ppm)
